# Supplementary material for: pQEB1: a hospital outbreak plasmid lineage carrying blaKPC-2
Source: Microb Genom. 2024 Sep 2;10(9):001291. doi: 10.1099/mgen.0.001291 (PMC11368168; doi:10.1099/mgen.0.001291)
Supplement: Uncited Supplementary Material 1. [file mgen-10-01291-s001.pdf]

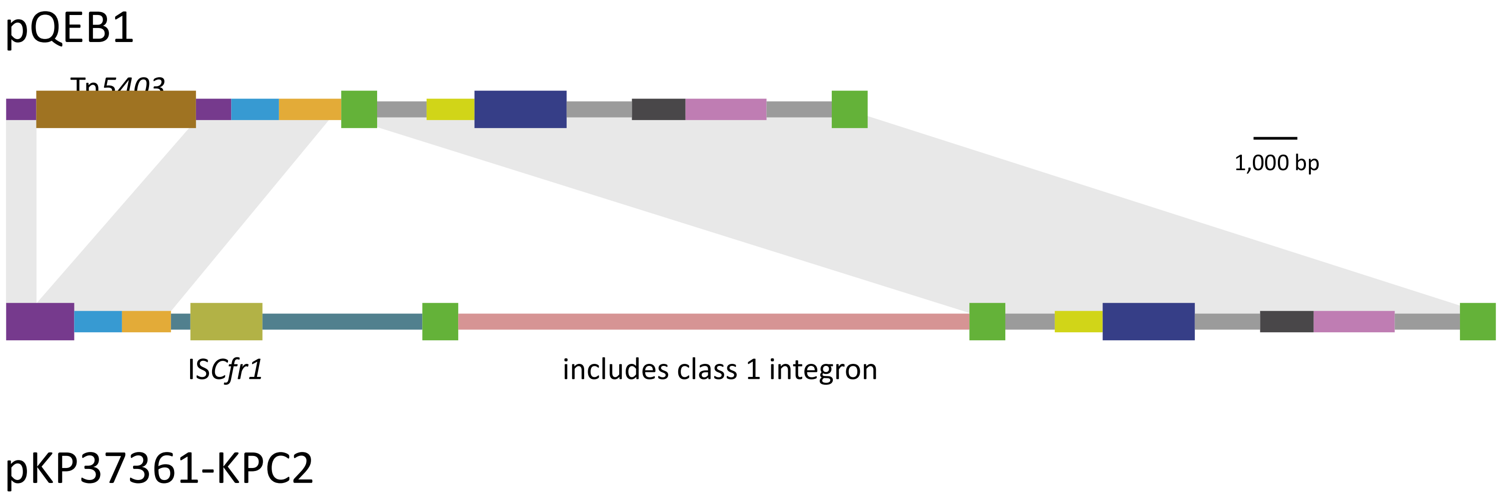

Figure S1: Structural variation in the resistance regions of pQEB1 and pKP37361-KPC2 (GenBank accession CP014944). Shared sequences (>99.5% identity) are indicated by grey shading linking the lines that represent each resistance region. Elements unique to each region are labelled. Colours here correspond to those used in Figure 1.

Table S1: pQEB1 variant sequences in the GenBank non-redundant nucleotide database

pQEB1 variant	GenBank accession	Note(s)
pQEB1	PQ162285	Baseline structure reference.
pQEB1_IS*Kpn26*	PQ162286	Acquired IS*Kpn26* and 4 bp TSD.
pQEB1_merTn::IS*4321*	PQ162287	Acquired putative mercury resistance transposon and 5 bp TSD. A copy of IS*4321* is inserted in the terminal inverted repeat of the transposon.
pQEB1_Tn::IS*5*	PQ162288	Acquired an unnamed transposon and 5 bp TSD. The transposon is itself interrupted by IS*5* and a 4 bp TSD.
pQEB1_inv1	PQ162289	IS*26*-mediated inversion event 1.
pQEB1_inv1_IS*5708*	PQ162290	Acquired IS*5708* after IS*26*-mediated inversion event 1.
pQEB1_inv1_arsTn	PQ162291	Acquired putative arsenic resistance transposon and 5 bp TSD after IS*26*-mediated inversion event 1.
pQEB1_inv1_inv2	PQ162292	IS*26*-mediated inversion event 2, after IS*26*-mediated inversion event 1.
pQEB1_inv3_Tn*5403*	PQ162293	IS*26*-mediated inversion event 3, and acquired a second copy of Tn*5403*, which has interrupted the backbone conjugative transfer gene *traN*.

*TSD = target site duplication

Supplementary File 1:

>pQEB1

GGAATTGGCCCACCTTAGACGAAACTGTAAAAAATGTATTTACTTGTTTGAACTTTGTGGTAGTGTGGAGAGTAATTTTTAACC
CACAAAGGCAAGGCGCATGGATAAGTTGCTGAACAAAAAGATAAAAGTTAAGCAGTCTAACGAGCTTACCGAAGCTGCTTAC
TACCTCTCGCTAAAAGCAAAGCGCGTTCTCTGGTTATGTCTTATGCAGACGTATTTCACAGCTTCAGTAAGCGAAGATGATGAT
GAGATGGCTGTACTCGGTGACTCTACTTTCAAAGTAAAGGTGGCTGACTATCAGCAAATTTTTCAGGTAAGCCGTAACCAGGC
TATCAAGGATGTTAAAGAAGGCGTGTTTGAGTTAAGCCGTTCTGCGGTAATCTTTTACCCGAAAGAGGGGCGTTTTGACTGC
GTCGCGCGCCCCTGGCTAACAGAGGCTGGCAGCCGATCAGCTCGTGGTATCTGGGAAATCGAATTTAACCATAAACTCCTGC
GGTACATTTACGGCCTGACGAACCAGTTCACCACCTACTCGCTCCGCGATTGTGGCAGTCTTCGAAATCCCCGGACGATCCGC
CTTTATGAAAGTCTTGCTCAATTCAAATCTTCAGGCTTATGGGTTACTACTCATGCTTGGTTAAATGACCGTTTCCTTTTGCCGG
AATCCCAACAGAAGAACTTGGCAGAGTTGAAACGATCTTTCCTTGATCCTGCACTCAAGCAGATAAATGAGAAAACACCTTTA
CTTGCTAAGTATAGTATTGATGATTCAGGAAAATTTCTGTTCTCAATAATTGATAAGCAAAATCCCGTCTGACATAAATCAGCAC
ACATGAGCCTGTCATTTGACAAATTTTTGTCATGAAGATGGGCGAATTTCCACACAGCACCGGCGCCCGGCAAGATGGGCGG
ATTCCCACACGACAGCGGCGCCCGGCAAGATGGGCGGATTTCCACACTACAGCGGCGCCCGGCAAGATGGGCGGATTTCCA
CACGGCAGCGGCGCCCGGCAAGGTGGGCGGATTTCCACACGGCAGCGGCGCCCGGCAAGGTGGGCGGATTCTCACGCGG
CAGCGGCGCCCGGCAAGATGGGCGGATTTCCACACGGCAGCGGCGCCCGGCAAGGTGGGCGGATTCTCACGCGGCAGCG
GCGCCCGGCAAGGTGGGCCGATTCCCACGCGGCAGCGGCGCCCGGCAAGGTGGGCCGATTCCCACGCGGCAGCGGCGCC
CGGCAAGGTGGGCCGATTCCCACGCGGCAGCGGCGCCCGGTAAGGTGGGCGGATTTCCACACGGCTGCCGCGCCCGGCAA
GGTGGGCGGATTTCCACACGGCAGCGGCGCCCGGCAAGGTGGGCGGATTCTCACGCGGCAGCGGCGCCCGGCAAGATGG
GCGGATTTCCACACGGCAGCGGCGCCCGGCAAGGTGGGCGGATTCTCACGCGGCAGCGGCGCCCGGCAAGATGGGCGGA
TTTCCACACGGCAGCGGCGCCCGGCAAGGTGGGCGGATTCCCACACGGCAGCCTCGCCCGGCAAGGTGGGCGGATTCCCA
CACGGCAGCCTCGCCCGGCAAGGTGGGCCGATTCCCACGCGGCAGCCTCGCCCGGCAAGGTGGGCGGATTCCCACACGGC
ACCGGCGTGCGGCAAGGTGGGCGGATTCCCACACGGCACCGGCGCGCGGCAAGGTGGGCCGATTCCCACACGGCACCGG
CGCCCGGCAAGGTGGGCCGATTCCCACACGGCAGCCGCGCCCGGCAAGGTGGGCCGATTCCCACGCGGCAGCCTCGCCCG
GCAAGGTGGGCCGATTCCCACGCGGCAGCCTCGCCCGGCAAGGTGGGCCGATTCCCACACGGCAGCGGCGCCCGGCAAGG
TGGGCGGATTTCCACACGGCAGCGGCGCGGGGCCAGTGGGATTCAGGAGAATAGGTGTTTTACCGAATGCCCTGACGAGG
CGTAAAAAAACCGCTTGGCGGCGGCCTCATAAAGCAGAAAACCCGCTCAAGGCGGGTTATCTGCTCTGTAGCCTGTGATGCT
TCGCGGGCATCCGGCATACAGCGAGGTGAAATTCTTCTTTTGGCATGTTAATTATACGTCTAACGCGGCATATGATCAAACCTG
TATTAAATAAGCCACTGTACCGTTTATAATGCTCTCAGATCAAAGAGGTAAAGCCCGTTTAGCCGCCTGTGTGATGAGCCAGTT
CAGACTCTTCAAAATCGAATTTGGTACTAAACAGGACCCGAACCGTGGGCAAGCACACGGCAACGGTATAGCCCTCTTCCGG
TTTCGCACCCGGAAGCCTGGGCGGCAGCGTGGTGAAATTCTTCTTTTGGTTAAGTGAATGGCATAACCGGATGGGCGGATTA
GAGGAAAGGGGATTGCCTAGTAACCTACGCGCCACAGAGATGGAGGTGCGGGGAATGATTGAGCTGATTATCGCTATTCTGA
CCTTAATTGCGGCTGTATTGCAGTTGATCAACTGGTTCCTTTAATGGTGCCGGAGTCTGTGAAGGTGAAAGCCTGAACGGGC
AAAACTGAAAGGTTTATAGCCGTCCTTCGGGGCGGCTTTTTTTCGGCAAAATTAGGGTTTTACCGAATAATGCAGAGTTTTAA
GGTGAGAATTTGCAGACTTGGCGTTTTACCGAACATAGATACTCCCTAGGCTGATAGGTGCATTAGTTATCACCCTACCTGAAC
ATATTGTAAAAGATGTCAGTCTCCAGTGACTTGTGTACTATCAACTGACAAGACTCTTACACGCAACGCAGGGGGATGGAGTT
TTATGCTTAGAAAAATAATCAGGGGTAGCGGATTCACTCAGTCAGAAGAAAAACTGATAGAGTTCGCTGATGATGCTTTTTTT
GGTCTTTGGTCTTATCCTAATGTTTATAGCGATGAGGGTTACTCTAAAAATAAAATTGGGAAAGAAGTTAGTGACTTATTAGTTA
TTTTTGATAAAGATATAATAATTTTTTCCGATAAAGCTATTACATACAATAAAAACAAAGATCCTAAGGTTGCATGGCAGAGATG
GTTTAAAAAATCAGTCATACAGTCTTGCACACAGTTATTTGGCGCAGAGAAGTTTATAAAAGATCATCCCGAAAGACTTTTTGT
TGACAAAGAATGCTCAGTTAACCTCCCCATTAAAATAGATAATTCTTTTAATTTTCATTTGGTGGCCGTCACTAATAATATTTCAG
ATCCGGCGATCTCGTACTTTGACAAAATAGAAAAAGGCAGCTCTGCTACTTTAGTTAACATATTTCCTTTAAACGCCCATCAATG
TCTAGAAAATCCATTTTGTGTCGGAGACGTTTATCCTGATAAGACTTTTGTCCATATACTTGATGAGACTGCCCTAAAACTACTG

TTAACCGAGTTAAACACAGCAACTGATTTCATTGGCTACCTTAACGAAAAAGAGAGGGTTGTAAGAGAAAGAACATTATTGG
TCAGCGCTGGGGAAGAAGAGACTCTTGCTGCTTACATTATGGGTGATAAAACCATAATATCAAAAGAAATTATTGGAAACGAT
CAAGGGATGACCATACCGGAAGGTGAATGGAAAAACTATAAAACCACTTTCAATTATCAATATCAGCTCTCAATGAAAAAGGG
TAGCGTTTTCTGGGATAACCTAATCCACAACTTCTCGACAAGTATATTGTCAGCTAACGTTGGTTTTTTTAGTGAAATTGAATTT
TCTACACATGAATTAGGTGTTAGAGAATTAGCCAAAGAAAGTAGGCAATCTAGATATTACCTTTCAAAGAACTTTAAAGAGAA
ATTAAAAACAACTCAGCCTCATCTAAGAACGTCAAGAATGGTCGAATCAATCGATGAGCCTGGAAAGTTTTACTTATTCCTTTT
TTTTCCTAACGATAGCAAGTTGAGTTACTCTGATTACAGAATTCAACGTATATCTTATATAAATGCTTATGCTGAGGTTGCCTTTA
ATAAATACAGACATATTAAAAAATTAATTACTATTGCAACAGAGCCGCAAAATACAGAAGGAAGATCTGAAGACCTAATATATA
GCATATCCCCAGAGAAATTTACCAAAGAGCAAAATGAAAAAGCCAAAAGATTATCAAGAGAATACAAAATACTAAGTGATTTT
TTACCTACTAAAACGACAAAGAGCGATAACTTTAAATCAGTTATATCAAAAGGTGAAAAAATAGGGCGGAATACACCTTGTCC
ATGTGGCTCCGGTGTTAAATTTAAAAAGTGCCATGGTGCGAATAATTAGCATTATTGTATGTATAACGGTAATGGCGCGGCAGA
GAAACCGGCGCGTTCTGCCCTAGTGTTGGCCTGCGGGTTCCCCCGCACCCGCTGTATGTAGTATCGGCAGCATCTGAGAAAA
CCACTACATGTAGTTATCAGCGCCACAACGGCGCGGGGACGAGTGCGGTTCGGAAAAATTGGGGTTTTACCGAATCCGGCA
AAAGATTGCTTCCTATAACGTCCGCTTCTGGCACACAGCAGCCGTTAAGATGTAAGGCCTTACGCCAACTAAATCTAATGGGA
CAGATTTAGTTGGTGATGGTCAAGTAATCTGCAAACGGTCACCAAGTAAAATGCAAATGGGTAGTCAAGTCCGATGCAATTAC
GCACCCGGCAAGGTGGGCCGATTCCCACACGACAGCAGCGCCCGGCAAGGTGGGCGGATTTCCACACGGCAGCGGCGCCC
GGCAAGGTGGGCCTATTCCCACACGGCAGCGGCGCCCGGCAAGGAGGGCCGATTCCCACACAGCACCGGCGCGCGGCAA
GGTGGGCCGATTCCCACACGACAGCAGCGCCCGGCAAGGTGGGCGGATTTCCACACAGCACCGGCGCCCGGCAAGGTGG
GCCGATTCCCACACGGCAGCGGCGCCCGGCAAGGTGGGCCGATTCCCACACGGCAGCGGCGCCCGGCAAGATGGGCGGAT
TTCCACACAGCACCGGCGCCCGGCAAGGTGGGCCGATTCCCACACGGCAGCGGCGCCCGGCAAGGTGGGCGGATTCCCAC
ACGGCAGCGGCGCCCGGCAAGATGGGCGGATTCCCACACGGCAGCGGCGCCCGGCAAGATGGGCGGATTTCCACACGGCA
GCGGCGCCCGGCAAGATGGGCGGATTCCCACACGGCAGCGGCGCCCGGCAAGATGGGCGGATTTCCACACGGCAGCGGC
GCCCGGCAAGGTGGGCCGATTCCCACACGACAGCGGCGCCCGGCAAGGTGGGCCGATTCCCACACGACAGCGGCGCCCGG
CAAGATGGGCGGATTCCCATATCGACATGTATGTAGCTTGTGTTATCCGTGGATTGTGCAGCTCAGCGGGTCGCTTGTCGTATG
GCGTAGTGTCCCCCGTAACCGGCCGCGTGCGGCCGCTAACGCGCAGTACGGCGCCGCGACCCGAAGGCGGGCCGCCGTTCC
CGCGCGCAGGCGCGCGGCGCCCACTGCGCACCCCCGTGGGGGACGTGCGGCAGCTGTGTGGCGGTGAGCGGGATTAGGG
CTTTGCAGGGAGGGGGCTGGGTCGGGCGATACGTTCAGCATTGCGGTTTCCGGCGATTTGCGGCCGGTGCCCGTTTAACTC
CGGCGTGGTCGCCTTCCATGCCCTGACGGCATAAGAAAATAAAACCGCCATGCTGCGGTCATTCATGATTTTGTGGTGTAGCG
ATAAATAGTCATGCGAGAAACGTTGAAGCGCTTAGCAACTGCACCAACTGTCATTTCAGGATCAGCAAGTAAGATTCTAATTT
GTTTAACATCTTCTTCAGAAAGTGACGGTTTTCTCCCTCCCACACGGCCCCTTGCGCGTGCAGCTGCAAGGCCTGAGCGCGTT
CTTTCAATATTGCGGTTGCGTTCAAAGCTAGAGAATATCGCCATCAGATGAGTATAGATTTCCCCTATAACTGGCGCATTTGTGT
CTATTCTGTCCTTGATGGCTATGAAAGTTATTCCGCGTTTCTTCAGGTCGTCGAGTAAAGTAATGACTTGACCCAATGAACCAC
CGAGCCGATCTAGTGCCCAAACTACTAGGGTATCTCCCTCGCGCAATGCTTTCAGGCAGTTCTCCAGTTCCAGCGCACCTTTTT
TGTCGCGCTTTGGGCCGCTACGTGAGGTCTGATCCTGATAGATTTGCTCACATCCAGCTTTTGTTAGTTCGTCAACCTGGTGCG
CCACATCCTGAAGATGCGTAGATTTACGTGCATAGCCGATTTTCATTCTTTTCTCGCTAATTAGTTATGGGGTTATTGTTATGTTG
ATACAGTAACGAGTTTTGTTACATGAGGGGAGTCATTTTTCGGGAGAAGTCAGGACTTTTCAAGACTGTCACAAAAACCATC
GTTTTTGATACATTAATTTAACCAATAGGTTGCAGATCAAATCGTCTGTAACAGCCTTTCTGGCTGTTTGTATATAATCATGAAAA
AATGGTGAGTAGAGTTTCAGGGTAACAGGGGATGCTTATGTCGGTTTTCCACAACTGGCTACTTGAGATCGCATGTGAGAATT
ACTTCGTCTACATCAAACGCCTTTCCGCCAACGATACCGGCGCAACAGGTGGTCACCAGGTAGGGCTTTATATCCCTTCAGGT
ATCGTTGAAAAACTCTTTCCGTCTATCAACCATACCCGTGAACTGAACCCTTCGGTTTTTCTCACCGCACATGTGTCATCGCATG
ATTGCCCTGACAGCGAAGCCCGGGCAATTTATTATAACAGCCGTCATTTTGGTAAAACCCGGAATGAAAAAAGGATTACCCGC
TGGGGTAGAGGCAGCCCACTTCAGAATCCTGAAAATACAGGGGCTCTGACGCTCCTGGCTTTCAAGCTTGATGAGCAAGGG
GGGGACTGTAAGGAAGTAAATATTTGGGTATGCGCCAGCACTGATGAAGAGGACGTCATTGAGACCGCTATTGGTGAAGTTA
TACCCGGAGCGCTTATATCCGGCCCCGCAGGACAGATTCTAGGCGGACTATCTCTACAGCAAGCGCCAGTAAATCATAAATATA
TTCTACCTGAAGACTGGCACCTGCGCTTTCCGTCGGGAAGTGAAATTATTCAGTATGCAGCCAGCCATTATGTGAAAAATTCCC
TTGATCCGGATGAGCAACTTCTTGACCGCCGGCGCGTGGAGTACGACATATTTCTATTGGTTGAGGAACTGCATGTTCTGGAT

ATCATCCGGAAAGGATTTGGCTCTGTGGATGAATTTATTGCGCTGGCCAATTCTGTCAGCAATCGCCGTAAATCCAGAGCCGG
GAAGTCTCTGGAACTGCACCTGGAGCATCTATTCATTGAGCACGGCCTGCGACACTTTGCGACGCAGGCCATCACAGAAGGT
AATAAAAAACCCGATTTCCTTTTCCCTTCCGCAGGGGCTTACCACGATACTGAGTTTCCCGTAGAAAATCTGCGCATGCTGGCA
GTCAAGACTACCTGTAAGGATCGCTGGCGTCAGATACTGAATGAGGCCGATAAAATTCATCAGGTGCATCTGTTTACACTCCA
AGAGGGAGTTTCTCTGGCTCAATATCGGGAGATGCGGGAGTCGGGTGTCAGATTGGTCGTGCCATCATCGCTGCACAAAAAA
TACCCGGAGGCGGTGAGAGCTGAGCTAATGACGCTAGGTGCGTTTATTGCTGAGCTGACAGGGCTTTACGCAGATATTCCAT
AGATTATCTCCCGGCATAAATACCGGGAGGAGCGATCAGATTCGTTCAACCTTGCACGAATCGGCATTAACCGCTTTCAGGAT
ATAAGGTTCAAGCAGTTTGGCTACGGCTTCAAACACGGGCACCACTACGGAGTTACCGAACTGCCTGTACGACTGAGTGTCT
GACACAGGAATGCGAAAAGGCCTGCCATCTACTTTTTCAAAACCCATAAGGCGCGCGCACTCTCGTGGAGTCAGCCTGCGGG
GCCGATGCGCCTGATTTTCTTCGTTCGCGAAGTCTGTTTCACCTGTGGCCATATCCCAGCCACGGTCTATCAGAATTTCAGACC
CGTCTTTGTGATAGCGAGCAGAAAGCGTACGGGCAATGCTTTCTTTATTTTCAGGATTAACGAGGCCAAAACCGAATCCGTTA
CCCTTAGCTGCGTGCTTTTTGGCGTAGTTATAGAGATACTCCCAGAGTTTCGGCGTCAGTATATATTTGCTGTCAACCACGGGT
TCCAGCAGTTCGCCAAATGACGGACGCTGTTCCGGATAAAAACGACTAATATCGCGCAGGGTAAAGCCCTGGTGAATGTTCA
GATCACGACGGAAACCGACCAAAACGATACGTTCTCGGTGCTGAGGTAAAAAGTGCTTTCCGTCGATAACTTTAGGATCGTTT
TTGCCCATCTCAGCTGCATCCGCAACTTCATAGCCCAGTTCGTCGAGGGTATCCATGATGACTTTAAAGGTTTTACCCTTGTCAT
GGCTCTTCAGGTTTTTAACGTTTTCAAGAACAAAGATGGCAGGTTTTTTTGCGCGTATAATACGCGCCACATCGAAGAAAAGC
GTTCCCTGAGCCTCACATTCGAAACCATGCGCGCGCCCGAGCGAGTTTTTCTTGCTTACGCCCGCAAGGCTGAACGGTTGAC
AGGGGAAACCTGCTAGAAGTACATCATGATCCGGCACATGCTCATTAATGTAAGCATAGGCATCGTTTTCAGGTACTTCAGGTT
TATCACTGAGCGTGACTTCCCGAATATCGAGATTGAAAGTGTGTTCCTGAGCATCGTTAAACCAGTTAGCTTTATATGTGCGCA
CAGCCTCTTTATTCCATTCACTGGTAAAAACGCACTGGCCACCGATGGTTTCGAAGCCCTTCCGTATACCTCCAATCCCAGCAA
ACAGGTCAATAAACCGGAAGGCATAGTCAGGGTGATGTGCAGGCGCTTCCGGAAGCATTTTTCGTAGAAGTTCCTCTTCGGC
TAACGTCAGCGTCTTAGGTGAGCACTTACCATTAATCCAGCGATTAAGAGTCTCGCGACTCCACTCATTTTTACCAACTTTTCTA
AGCAGTTCAGCCACGTACTTCTGGTCATAGATTTCCAGCACCTGCCCGAGCAGCTTTTTATAATTTTCCTGTCGCAGTTGTTCTT
CCGCTTCTGCTTTCTCAAGCAGATCCTGCGCCAGTAATTCAAATTCAGACATATTGCCTCCATTGGGTCTTATGGGTGAAACTG
TATCACTCATTTGACCCAGATTGAATGTTTTTATCTGGATATTTAAACAGGTTTATTGTTAGGTAACGCACGTTGGCCACGCTGG
AGCGTCTTCTGGGCCTGCTGTCGGCCTTTGAGGTCGTGGTATGGATGACGGATGGCTGGCCGCTGTATGAATCCCGCCTGAA
GGGAAAGCTGCACGTTATCAGCAAGCGTTACACTCAGCGCATTGAGCGACATAACCTGAATCTGAGACAACATCTGGCAAGG
CTGGGACGGAAGTCACTGTCGTTCTCAAAATCGGTGGAGCTGCATGACAAGGTCATCGGGCATTATCTGAACATAAAACACTA
TCAGTAAGTTGGAGTCATTACCGGTTCTCTTTGTCTTTTAGTGATTCTATAAACCTCATTACGTCTGAATATAAAAATCTATTATTT
GATTTATGTGGCTCATGAGGTTGTGGGATGGTCTTGTTTTTGAATGTGCCAGTTTTCTTAATGGCAAAGATTAATTCACCTTCT
GTTATTCCTAACATTTCAGCAAATGTTTTTGCTTCTATAGTTACTGACTTCATTTAATTAACTCTCATGGTATCGATTTTCTTTACC
GGCATCTTTAACAATGGTGCTCGTTTCTAGTGTTGCTGCGGTACGCTTCATCATCGTCTGCGGGGCGGTTGCGATAGTGAAGG
AGCTGCCGGGCGTGAGCAAATCTATCAGGCGCTGGCCGCTGATAATCTCCATCCGTTCACTGGCAATACTGACAGATTTTGAA
CCTGCGCCGGTTTTCCCGGTATGGCAAAACAGACCGCGACAGTTATGACGTTTAAGCAACTTCTCGAACTCCTGTACGTGCTG
TAAAGCAATATGGCCGCGATAGCGTTTAGCCTGAATAAGATAGCGATATTTTCCTATTATTACCTGGCCGTCAATGCCTCCATCG
CCGGTATAGCGTTTGTTTCTGATGGTTCTGAAGCCATGCGCGGCTTTGTTGAATAAATCGAACTTTTGCTGAGTTGAAGGATC
AGATCACGCATCTTCCCGACAACGCAGACCGTTCCGTGGCAAAGCAAAAGTTCAAAATCACCAACTGGCCCACCTACAATAA
AGCCCTCATCAACCGTGGCTCCATAACTTTCTGGCTGGATGATGAAGCTATTCAGGCCTGGTATGAGTCGGCAACGCCTTCATC
ACGGGGAAGACCTCAGCGCTATTCTGATCTCGCCATCACCACCGTTCTGGTCATTAAACGCGTGTTCAGGCTGACCCTGCGGG
CTGCACAGGGTTTTATTGATTCCATTTTTACACTGATGAATGTTCCGTTGCGCTGCCCGGATTACACCAGTGTCAGCAAGCGCG
CAAAGTCGGTTAATGTCAGTTTCAAAACGTTCACCCGGGGTGAAATCGCGCATCTGGTGATTGATTCCACCGGGCTGAAGGT
CTTTGGTGAAGGCGAATGGAAAGTCAAAAAACACGGCAAAGAACGCCGTCGTATATGGCGAAAGTTGCATCTGGCCGTTGA
CAGCAACACACATGAAATCATCTGTGCAGACCTGTCGCTGAACAATGTGACGGACTCAGAAGCCTTCCCGGGTCTTATCCGGC
AGACTCACAGAAAAATCAGGGCAGCATCGGCAGACGGCGCTTACGACACCCGGCTCTGTCACGATGAACTGCGGCGTAAGA
AAATCAGCGCGCTTATCCCTCCCCGAAAAGGTGCGGGTTACTGGCCCGGTGAATATGCAGACCGTAACCGTGCAGTGGCTAA
TCAGCGAATGACCGGGAGTAATGCGCGGTGGAAATGGACAACAGATTACAACCGTCGCTCGATAGCGGAAACGGCGATGTA

CCGGGTAAAACAGCTGTTCGGGGGTTCACTGACGCTGCGTGACTACGATGGTCAGGTTGCGGAGGCTATGGCCCTGGTACG
AGCGCTGAACAAAATGACGAAAGCAGGTATGCCTGAAAGCGTGCGTATTGCCTGAAAACACAACCCGCTACGGGGGAGACT
TACCCGAAATCTGATTTATTCAACAAAGCCGCCATGCGCTTCAAATCCTTCCAGCAACAGTTCTTCAAACACAAAAGGATCAAT
TTTCCTCAGGTAGTTAATTTTTTGTGGGAAGCCCGGCAACGTCTTTATGCGCTCCAGCACCCGCCGCGCACTTTGCTGCTTCCT
TTTGTGTCGTCGGTTGCGTACTGAACGCCGGAAGAATACAACGGCAAACAGTGCGATGGCGCTGCAAGCCCATAGAATAAGG
TTTTCTGTAGTGGGGAAGGGGAACATGGTGATAGTGTGCTTTCTGTGGGTAAAGAAAAGGGCGGTTAAACCGCCCTGGTGT
TTAGCGACGGCTGTAAACCTGCCACGAAGCGCTGCCTGACTGATTTTGGCAAATCCGCCCGTAAAGTACGGTGCCGGTCGAG
TAGCGGGCGCCATTCAGATAGCAATAGCCGCTGGATTGCAGCAGTTCGTTGCGCAGCTTTTCTTCCTGCTTTGATAGCCGGGA
CTCCGCAGACTGCAAACGAACGGATAATTCGTTAATCTGGCGTTGCTGGTTATTCATCTGGCTCTGCATCGCATTACCTTTATCT
TGGCTGACGCAACCAGTTAAGAGAGCTGTACAGGCTAATGCACTTAATATAATTTTTTTCACGTTGGCTCCTTAAATTGAGATT
ATTCCTAGCCCGCCTATAAGCGAACTTTCCCGTATTTACTTATGATCTGGCTTATCATCGACTGGTTACTTCCACCTTCGCCATTAT
CCGGGCATTCATTAAGAAAAGCCTTCCTGGCATCCCTCGTGTGGTTGGGTAAAAAGCCGTGCTTGTTCTTTTTAACGATATTGA
AGAAAGCAGCTTCAGCACTGTTACACTCGCTTCCGCCGCTATCGCCGGTGAGCTTGCCCGCCATGCACATAATAACTTTGCAG
GGATCTTCAGCATGGCTGGCAGGAAGATAAAGCAGACTACCAGCTGCTATCAGAGGGATTAAGAGTTTCTTCATTGTTTTGTC
CTTAACAGTTTGTTCAGATATACACCCGCCAGAATGTTGATAACGGTAAGTAATATTAATAATAACGCAGAGTTATAAATAGATTT
GTAACCTATATCGCCTGCGATATATTCGACAATAAAAACGAAAATCGTTAACATTGCGAACAACTTAATTAACTTTCCTGTAATTT
TCTTAACGATATAAGCAACTGAATAAGAGCCGGATTTGATGATGCTGGCGAGTAACCTGATAGCATGGACTCGGAATTTTCTTA
AAATGGGCGGAATGGAAAATGCCTTTTTCTTCCTTCTGCCTTTTTGCTTTTCCGCAAAATCAATAACATCGCCCATTTTTATACT
CTCCGGTTATATCTTTAGATCATCAAGTGATAAACCACTATCAAGCAGCTCTTTGAGCCATGTTGGTCGGCGGCCAGTCCCGGA
CCAGGTATTTTCTGCGTTCCTGGGGTCGCGGTATTTTACTTGTCTATGTTCTCTGGGGTTGGCAACGCCCTGCATTTTCATGGC
TTCCAGGCTAATGCCAGCGTCCAATTTTGCAGTTAGCCAGGCCGGGCGCTTCCCTATACCAGTCCACGTATTAAAAGGGTTATC
CGGGTCACGATACATGGGTTCACCTTTGGGGCGTTTTTCTGATTTCAGGGGAACCAGCATTACCTTCCTTCCTTCAGAGGTTT
TCTTTTCGTTTCTTCTTGACACGATCTCATTAGCCCTTATCATAAACATAAGTCGTCGAAATTACAATTACGCGGTGAAACGTAA
ATGAGTAAACATCCAAAACTTCTGGTTCTCGCTCTGGCCTGCCTTGCTTGTGCTGGCCGTGCCAGTGCTGCGCCTGCCTCAGA
TGAAGTTGCCAGGCTTGCGCAGAGATGTGCGCCTGATGTTTCACCCTTAACAATGGCGTACATCGTCGGCCATGAGTCCTCAA
ATGGGCCGTACAGGATCAATATTAACGGTGGTAGTACCCAGTTAAAACAGCAACCACGTACTGAAGCTGAGGCCGTCAGCGT
TTCGAAAGTTCTGCTGAAGGATAATAAAAGTTTTGATATGGGCCTTGCACAAATTAACTCAAATAATTTAGTGGGCCTGGGTCT
TTCGGTTGACGATATTTTCAAGCCCTGCATCAACCTGCGGGCGAGCCAGACCATCCTTAAAGCCTGTTATGATAGCGCCCTGA
AATCCTATCCAGCCGGGCAGGTTGCGCTGAGACACGCGCTTTCCTGCTACAACACCGGCTCACTCATAAACGGGATTTCTAAC
GGGTATGTCACGAAAGTTATCAACGTGGCGCGTCAATCAACTGATTTGAAAATCCCTACGCTGCTACCTGATGGCCAGACCAG
TGAGGACAGCACCGCGACTGAGCCTCAGCAGGCAAAAAGTACGGCCACGCAGTATGACGGTGAACAAGATGTTTTTGGTTC
GGGTGATGGCGATGCCTTCAGCCGAAATAATACGGATGCCTTTTTAACCAAACAGGAAACAGCGAAGGGGGAGTGAGGTTA
TGGATGGAACGTTTGTACCTTGTATTGCAATCACAGATCCTGGAGCTATTTGAAACAGGAAAAGTGAAGGAGGTAACGATAA
AACGGGTTTCATTAAAGACGTGGTATCCCGTTTTTCAGATAGACGATGAACAGTTGGGCCAGATCGCATGTTCCATTCGGGTT
AACAAAGAGCATGAACTACGAACCTGGGCTGATTTAAGGCTACTGGCAGAGTTTTTGAAAGATAAGTGTGGCGTTGAAGAAT
GCCGGTTAAATCTGCAATCAACAGAAGATAGTGAGTAAGGAGAAAGTATGACCACGTTGTTTAAGAAGTATGGCCCTGCGGT
AGTTATGGGCGTTTTGTCCATTGCCCTGCCGCAAATTGCGCTGGCCGCTGGCACCGATACTGGTGAATCAACCGCTACATCAA
TCCAGACGTGGTTGAGCACATGGATTCCAATTGGTTGTGCTATTGCGATCATGGTTAGTTGCTTTATGTGGATGCTTCACGTAA
TCCCAGCCAGCTTTATTCCTCGTATCGTAATCTCGCTGATTGGTATTGGTTCTGCATCATTTCTGGTTTCCCTGACGGGCGTAGG
AAGCTGAACAACGCGAAAAGGGGGGACTTTTGTCCCCCAAAGTGAGGACTACAAAGATGTTCGTTGACGGGAAAAGACCG
CTTTTCAAAGGTGCGACTCGCTTACCTCGCGCGCTGGGTGTACCACGTAATGTAGCTATGATGATATTCATGATTTCTGCCTCGC
TTTTTATGATTATTCATATGTGGGCGATCCTGGTGTTCGTCTTTTTGTGGATTCCTTCAGCTGCATTAACAAAATATGACGACCGC
ATGTTTCGAATTATGGGCCTGTGGTTGAAAACCAAATTCAGTAATTGGTTTGATTCTCCGTTTAAGCAGTGGGGAGGATCGTC
TTATTCCTCTGTTGACTACAAACGTAAGGGTTTAAAATAATGAGAGCTGCCACCGCTACGAAGCCAAAAAAAAATTGATGCCTA
CCGTAAGGAGCCATCAGTAAATAAAAAGTATTTGCCCTATTCTTATCACCTCAATGATTACGTGATTTCGATGGAAAACGGCGA
TCTGATGGCTTTTTTCAAGCTGGATGGCCGCACACATGACTGCGCATCAGATCGGGAACTGGTCACCTGGCATAAAGACCTTA

ATACGCTGGTCAAGAGCTTCGGAACAGACCATGTAGAGCTGTGGACGCATGAATATCACCATGAGGCTAAAGAGTACCCGGA
TGGTGAGTATGACCATTTTTTCCCTGCTTATGTTGATCAATATAACCGTAAGCTGCACGGTGATTCCAAGCAGCTGATTAATGAC
CTTTATCTGACCGTTATTTACAAACAGGTAGGGGATAAAACACAGAAGTTTCTGGCGAAATTTGAAAAGCCGACTCGTGACG
AAATTCAGCGAATGCAGAATGAGGCGCTTGAAGGTCTGGAAGATATTTCTGAACAAATCCTGGAAGCAATGAAGCCGTATGG
CATTCAGCAGTTGGGTATCTATTATCGTGACAAACGCGGTGTTGAAATTCCTGCGCCTGATAAAAAAGAACGTGAAGAACTTG
CTGAAGTCGATGAATCAGACATTTTTGACGAAGCCATTGTTATCGAACGCAACGAGCCTGAACCTTCGCAGGCTCACGCTTAT
TCAAAAGCGCTGGAGTTCCTTTATTTCCTCGCAAATATGGAATGGGCCATCGTGCCTGTTTGCCGTGATCGTATCCGTGAGTAC
ATCATGGACAACCGCCCTGTTAGCTCACTGTGGGGGGATGTTGTCCAGATCAGAACGGTAGATCACAACTTCTATACCACCGG
CATTGAATTTCGTGAATACGAAGAAGATACAGAGCCAGGCCAGCTTAACATGCTTAAAGAAGCCGATTTTGAATACCTTCTGA
CGCAGAGTTTTTCTTGCCTCTCTGAATCTTCAGCTAAAACGTTTCTGACGCATCAGGAAAAAATCTTTGCAGGAAACGCGCGAC
CGTGCGCAAAGCCAGCTGGCACAGCTTGGTACCGCGCTCGATATGCTGACGTCCAGAGAGTTCGTGATGGGCTACCATCATG
GAACCGTGCATGTCTGGGATAATGACCAAAACGCGGTACAGCGCAAAGCGCGTCGTGTGAAGGTTATGCTAACCGGCTGTG
GCGTGGTTGGCGGGACTCTCAGCCTGGCCTCTGAGGCTGCATATTATGCGAGACTGCCTGGCAACCAGAAATGGGCGCCGC
GCCCGGTTCCGATAAACTCATGGAACTTCCTGCACTTCAGCCCGTTCCACAATTTTATGCGTGGCAAGCCTGACAATAACCCGT
GGGGGCCAGCGCTGACCATGTTCCGCACGATCAGCGGTACGCCACTCTATTTTAATTTCCATGTGACCCCGCTTGAAGAACTT
TCCTACGGTAAACGCCCGCTGGGCCATGCGTTAATAACGGGTATGTCGGGGGAAGGTAAAACCACGCTGCTTAACTTCCTGCT
GGCGCAGTCAATGAAGTACAACCCGCGGCTTTTTGTTTATGACCGTGACCGCGGTATGGAGCCGTTCATTCGAAGCGTTGGT
GGCTACTATAAAGTTCTGCAACAGGGTATGCCGTCCGGGTTTGCCCCGCTTCAGATTGAACCGACCAAACGCAATATTGCCCT
CATTAAAAACCTGTTCCGCATTTGTGTGGAAACCACCAATAACGGGCCTATCAGCGCAACGATGGCTACCGAACTGGCTGAAG
GCGTTGATGCGGTTATGGGGGAAGGCTCACTTATTCCACGCGAGGCGCGCACCGTTACTATCCTGGACGGGTACGTGAATGA
AGTTGTGGAAAATGGCGTATCACTGAAAGGGCTGCTGCGCGAATGGACGCGCGAAGGCCAGTATGGCTGGCTGTTTGACAA
TGATAAAGACAGCCTGGATCTCAGCGCGAATGATATTTTTGGCTTCGATTTATCCGAGTTTATCGCAGCCAAAGAGGAAGTATC
CAGCCCGGCCCGTACTCCGCTCATGATGTACCTTCTGTACCGGGTACGTGACTCCATCGACGGCAAACGCCGCGTCATTCAGT
GCTTTGACGAGTTCCACGCCTACCTTGACGATCCGGTTATCGAGCGTGAAGTTAAGCGTGGTATCAAAACTGACCGTAAGAA
AGACGCTATCTATGTGTTTGCCACGCAGGAGCCGAACGATGCGCTGTCCAGCCGTATTGGCCGCACGATCATGTCGCAGACCG
TCACAAAAATCTGCCTGCGCGATCCGGAAGCTATCCGAGAGGATTATGCCTTCCTTACTGATGCTGAATACGACGCGCTGATGT
CGATTACCGAACACTCCAGACAGTTCCTGGTTAAACAAGGGCAACAGTCTGCGATTGCTTCTTTCAATCTCTACCCTCGCAAC
AGCGACGATATTGATGCAGATATTAAGACAATGGACAACGTTCTTAGCGTGTTGTCCGGTGAACCACAAAACGCCGAAATTGC
GCATGAGCTGGTTGAACGGCTCGGTAATGACCCTGAAGTATGGCTCAAAGAATACTGGCGCCTGACGGCTTAACAACGAGGC
AAAACACCATGAAAAAAACACTGACGGCAGTATTGCTGACCACCGGCCTGATACTGGGAGGCGCGCAAAGCGCTTCCGCAG
GCATCATCGTGACCAACCCTACTGAGCTGGCTAAACAGGTCGAGCAGCTTCAGCAAATGGCGCAGCAGCTGGAGCAGCTTA
AAAGCCAGCTGCAAACGCAGAAAAATATGTATGAGTCGATGGCAAAGACAACCAACCTGGGCGATCTGCTGGGGACGTCTA
CCAGCACGCTGGCAAATAATTTGCCGGACAACTGGAAGGAGATCTACAGCGACGCCATGAACTCCAGTTCTTCCGTCACGCC
TTCAGTTAACAGCATGATGGGCCAGTTTAATGCGGAAGTTGACGACATGACGCCCAGCGAAGCAATTACCTACATGAACAAA
AAGCTGGCTGAAAAAGGCGCTTATGACCGTGTTATGGCAGAAAAAGCCTACAACAACCAGATGCAGGAACTAACCGATATGC
AGGAGCTGACGGAGCAGATTAAAACGACTCCAGACCTGAAATCGATTGCTGACTTACAGGCCCGTATCCAGACGTCACAGG
GTGCTATTCAGGGTGAGCAGGCGAAGCTGAATCTGATGAACATGTTGCAGCAGTCACAGGACAAGCTATTACGTGCGCAGAA
AGAACGTGCCACCCACAATTTTGTTTTTGGAACCGGCGGGGACGTTACCGCGTCACCTTCAATTAACTGAGGTAATTATGAAA
AAACTACTGCTTGTTATCCCTTTCCTCCTAGTGGCCTGCGATGCCTCGCATGACGTGGAGTGGTACAAAAAACATGAGAAAGA
GCGCAAGGCAACAATTCAGGAATGCAAGAAAGACGCGGATGAACTTCAGAAACCTGATTGCAAAAACGCGCGCGAAGCCG
ATCGTCAGCTGTTTGTGTTCGGCAAAAAAGACGGCGAAATCAATTCACCGAAAATTTAGGAGTAAGGAGGCAATATGGCATT
CACCCTAGTCGCAGACATTTTCGCAAAAGTAGACGGGGCGATTACGTCAATGGTGAGCGCCAATGTTGCCACCATTATCTCTG
ATGTAACGCCTCTGATTGCCACCTGTCTGACAATCAAGCTGATGGTTCAGGGGATGTACTCAGCGTTTAATCCGGGGGCGGGC
GACAGCCTGAGTTCGCTGATTAAAGAGTATCTTTCCATAGCCCTTATCCTGAGCTTTGCAACGGCGGGCGGCTGGTATCAACA
GGAACTGGTCAACGTGGCGCTTCACCTGCCGGATGATTTTGCCGGGATACTGTCTGCCCCTAATAAAGTCGGTGCAAGTGGC
GTACCGGCGATTATTGATAGCGGTATTGAAAAAGGTATCAAGATCGTCAACACCGCATGGGAAGCCGCAGACGTGTTTTCATC

GAGCGGCCTGGCCGCGTATGCCATTGGCGGCATTATGATGATTGCTACCGTTGTGCTGGGCGGCCTCGGTGCGGGCTTTGTG
ATCATGGCTAAGATCCTTCTGGCCGTTACGCTTTGTTTTGGCCCGATTGCAATCTTCTGCCTGCTGTGGGGAGCGACAAAAAA
CATCTTTGCTCGCTGGCTGGCGTCGGTCATTAACTATGGCCTTGTCGTCGTCATTCTTGCGCTCGTGTTTGGTTTCATCATGCAG
ATGTTCGACAACCTCCTGTCCTCGATGAACTCTGATGCCGCTTACTCATCAATCACTGGTTCTATCTCCGCCTTATTACTGACGGT
CATTTCCGTTTTCGTTCTGTTCCAGATTCCGCAAATTGCCGCCAGCTGGGGTAGCGGTATCAGCGCCGGAGTTGCTGACGCCG
CACGCTCTACGGGTTCTTCCATGCAGGCGCTTGGCAATATGGGCAGCCACGGCATGTTTGGCGGTAATGCGTTCAGAGGCGG
TAACAGTGGCGGCGGCCAGCAATCGGCAGGTGGAGGAAGTGGCAGCAACAGCGGAGGAAGCAGTGGTTCTAATTTAAGTG
GTAAGGCAAGGGGCAGTCGCGGGAAGAAGGCTGCATAAAATTAAAACAAAAGTCGTTGAAATTGCAATTTCGACGACTTAT
TATAATTAGTACGTTCAACAACCGATAATGGATGCCGTAATGCGCAGCTTATTGCTTATGGGAGTTCTTCTGATTAGCGCCTGTT
CCAGCGGGCATAAACCGCCACCGGAGCCGGACTGGAGCAACACCGTTCCAGTAAACAAAACAATCCCGGTTGATACGCAAG
GTGGTGCAAATGAAAGCTAATAAAAAAACAGGGCTTACACGTGAAGCCATTAAAGAGTTCAACGAAAGCCGTAAAGGGCTT
GAAGTTGATCTGATGGATGAAGTGCTGAAGTCCCGGCGTACCGCCTGGATGGTTGCCACCGGTTCAGCGGTGGTAACTGTTT
TTGCACTCTCTTTAGTTGGTTACGTGGTGCATAAGTACAGCCAGCCAATCCCCGCACATCTGCTAACGCTCAACGAGGCCACTC
ACGAAGTACAGCAGGTCAAGCTGACCCGCGACCAGACCTCTTATGGTGACGAAATTGATAAGTTCTGGCTGACACAATATGTC
ATTCACCGTGAGAGCTATGACTTCTATTCAGTTCAGGTCGACTATACGGCCGTTGGCTTAATGTCCACGCCGAACGTGGCAGA
GTCTTACCAGAGCAAGTTCAAGGGCCGCAACGGTCTTGATAAGGTTCTGGGCGACAGTGAAACGACCCGCGTGAAGATTAA
CTCTGTGATCCTCGATAAACCGCACGGCGTAGCAACGATACGCTTTACTACGGTTCGCCGCGTGCGCAGCAATCCCGTTGATG
ATCAGCCGCAGCGCTGGATTGCCATTATGGGGTATGAATATAAATCGCTGGCGATGAATGCTGAGCAGCGTTATGTCAACCCG
CTGGGTTTCCGCGTGACGAGTTATCGCGTCAACCCTGAAGTTAACTGAGGGCTGCCCCATGAAAAAACTACTTCTTTCAGCAG
TCGTTTTGTCAGTCCTGGGAGGCGCGGCCACTAACGTTATGGCGCTTGAGGTTGGCCGCAATTCTCCTTATGACTATCGCATTA
AAAGCGTTGTTTATAACCCTGTTAATGTGGTCAAAATTGACGCTATCGCCGGTGTGGCTACCCACATTGTTGTCGCGCCTGACG
AAACCTATATCACTCATGCTTTTGGCGATTCTGAAAGCTGGACGTTTGCGCACAAAATGAACCATTTTTTTGTGAAGCCGAAA
CAGGCCATGAGTGATACCAACCTGGTGATCGTCACCGATAAGCGCACCTATAACATCGTCCTCCATTTCATCGGTGAAGAAACG
AAGAAAAATGCAGACGGTACGGTATCAAAATCCTTTATTGAAACGCCGTGGGCTGTGCGCCAGGCCGTTCTTCAGCTGACCT
ATGAATATCCGTTTGAGCAGCAGGAAAAAGCCAAAAGCGCGGCTGATAAAAAACGCATTACGCAGAAGCTGAAGCAGACGG
CTTTTGCGGGGGCGAAGAACTATCAGTACGTAATGAGCGAACAGCCTGAAATGCGCAGCATCCAGCCGGTTCACGTCTGGG
ATAACTACCGCTTTACCCGGTTTGAGTTTCCGGCCAATGCGGAGTTACCGCAGGTCTACATGATTTCGGCCAGTGGCAAAGAA
ACGCTGCCTAACTCTCATGTTGTGGGTGAGAACCGCAACATCATCGAGGTGGAAACCGTCGCTAAAGAGTGGCGTATTCGTCT
GGGCGATAAAGTCGTTGGCGTTCGTAATAATAATTTCGCGCCGGGCGCCGGTGCGGTAGCAACCGGTACGGCTTCCCCGGAT
GTGCGCAGGGTTCAAATTGGGGAGGATAACTGATGGCCCGTAAAAGTGTCGATGTAGATCAGGAACTCGATGAAAACACCG
GAGACGGTGAATTCGAAAGCGAGCGTGGCGGATTTAAAGGCAGTAACCGCCGTTCGGCTCCTGGTATGAAAGCCTTTGTCAT
ACTGATGGCGCTGCTTGCTTTGGTATTCATCGGGATTACGGTCATGGGTAAAATTCGCACCCCGGCTAAAGCTGAAGCTGATA
AAGACGGTGGTAAAGCGCAACAGGCCAATACACTGCCAAACTACAGCTTTAACAGCGATCCTGATGTTAATAAACCTGCAACT
GCGCAGAATAGCGCCACTGATGCCCGTGCTGTGCAGGCTGCCGCACAGGCAGATGCAGATGCGGGCAGCAGCAATACCGCC
GCGCGTACCTCTAATAAGCGTAAAGAACCTTCGCCTGAAGAACTGGCTATGCAGCGTCGTCTGGGCGGCGAGCTGGCCCAGA
CTAATCAGGCGGCTACAAGCAATAGTCCCGGAGTGCAGCCCCAGGACAACGAAACAAGCGAAGGTAGTTCAGCACTCGCTA
AAAACCTGACTCCTGCAAGGCTGAAGGCTAGCCGCGCTGGAGTCATGGCTAATCCCAGCCTGACTGTTCCGAAAGGCAAAAT
GATCCCCTGTGGTACCGGCACCGAGCTGGATACCACTGTTCCGGGTCAGGTTTCCTGCCGGGTTTCACAGGACGTTTACTCA
GCTGATGGACTCGTTAGGCTGATTGATAAAGGCTCATGGGTTGACGGGCAGATTACCGGTGGTATCAAAGACGGCCAGGCG
CGCGTGTTTGTTCTCTGGGAGCGTATCCGCAATGACCAGGACGGGACAATCGTTAATATTGACAGTGCCGGAACGAACTCAC
TCGGCAGCGCGGGGATTCCGGGCCAGGTGGATACCCATATGTGGGAGCGTCTGCGTGGTGCGATCATGATTTCGTTGTTCTC
TGACACCTTAACGGCGCTGGTTAACCAGACGCAGAGTAATAACATTCAGTACAACAGCACAGAAAACAGCGGTGAGCAGCT
GGCGTCTGAAGCACTCCGCTCTTACATGTCTATCCCCCCTACCCTCTACGATCAGCAGGGTGATGCGGTGAGCATTTTTGTTGC
CCGCGACCTCGATTTCAGCGGCGTTTATACGCTCGCAGACAACTAAAAAAGTGGGCGCTTAGCGCCCGCTTTTCTTCAGGAG
TAATCATGACTGATGCAGCTTTCTATCAACTTGGCCCACTGCGCGAGTATTTAGAAGATCCTACTGTTTTTGAAATTCGCATTAA
CTGCTTTCAGGAAGTTATCTGTGATACGTTCAGCGGCCGCAGGGTTGTGCAGAACGCGGCAATTACGGCAGATTTTATTAGG

AACCTTGCTAAATCGTTGGTGAGCAGCAACAAGCTGACCATGCAGGCCATTAATGACGTGATCCTGCCTGGCGGGATCAGGG
GCGTTATCTGTCTGCCCCCTGCGGTGATTGACGGTACAACGGCCGTAGCGTTTCGTAAGGATTTGGCGGCCGATAAAAATCTG
GAGCAGCTGACCCGCGAGGGGATTTTCAGTGACTGCCGGAAGATTACCGGCAGCAAGCAAAGCCTAACGGATGATGATTTT
TTCCTTAAAGAGCTGCACAGCAGCGAAAAATGGCCCGCATTCCTGCAAACCGCCGTTGAGAAGAAACGCACTATCGTGATCT
GCGGTGAAACCGGGTCGGGGAAAACGGTACTCACGCGCGCGCTGTTAAAATCGCTACATAAAGACGAGCGTGTAATTATTTT
AGAGGACGTTCACGAAGTCACGGTCGATCACGTTGTAGAAGCCGTTTATATGATGTACGGCGATGCAGGAAAGATCGGCCGC
GTCAGCGCCACTGATGCCCTGCGAGCCTGTATGCGTCTGACACCGGGCCGTATCATCATGACTGAGCTTAGGGATGATGCTGC
GTGGGATTATCTTAAAGCACTTAATACCGGCCATCCAGGCGGTGTTATGTCAACGCACGCTAACTCTGCGCGCGATGCCTTTAA
CCGTATTGGGCTGCTTATCAAGGCGACCCCTATCGGCCGTATGCTCGATATGAGCGATATTATGCGAATGCTCTACTCCACCATT
GACGTTGTGGTGCATATGGAAAAGCGGAAAATCAAAGAAATTTATTTTGACCCTGAATATAAAATGCAGTGTGTGAACGGGA
GCCTGTAATGAAAAACTTAGCAACCTGGCTTCTGGCCGCAGCATTTACGACAGCCGCCCTGCCCGCCTTTGCGGTGGAACCA
TCCGTTCAGGTTGGCTACTCGCCTGAAGGGGGGGTAAGCGGGAACCCCAGAAAATTCCGCCATTCCGCATTGTGGAATTTTT
TGGGGGGTGGTCCGCGGCATGACGACCCGCCAAAGGCTACCGCATCTATCTGAGGCGCTGTGGCGCGACGCTGAAACCCCG
GGGAATTCCGCTACTCCAGTTCCAGGTCCGCAATAGTTCGTCCAGGTCGGGTGGCGTACGCCGGAAACGCCGATTTTTCCGC
AACCGTTTCTGCGTTGTCGGCAAGGTGGTCTCAGAGAGCGCTCAAGAAGCGGTTTTTCTCGTATGTTTAGCTACGGTGGCCT
AGGAGCCTGCGCGGCAGAAGCCAAATCGGCGGAAGATCGAGGACGATACGGGGATGGCTGCCAGTTACCTTCCTTACCGAC
CCGACCAATCCTATCTGCTGCCCCCTTCTCTGGGAGAGTGGCTACCTGAAGGGCATCTTGCCTACTTCATCAGCGAGACTGTC
GATACGCTGGACTTGAGCGCATTCCACGCCCGGTATGCCGGCGGTGGTCCGGGCAATCAGCCGTTTCATCCGGCGATGATGG
TCAAAGTGCTGATCTATGGTTACGCGAGCGGCGTCTTCTCTTCGCGCAAACTAGCCAGGAAGCTGTACGAGGATGTCGCGTT
GCGTGTGCTGGCCGCTGGAAACTTCCCGGCCCACCGCACGCTGAGTGACTTCCGTGCCCTACACCTGACCGAGCTTGAGAAT
TTGTTCGTTCAGGTGGTGCAACTGGCGCGCGAATGTGGGCTGGTGAAGCTTGGCACGATCGCGGTCGACGGCACCAAGGTA
AAAGCCAACGCCAGCCGCCACAAGGCGATGAGCTATAAGCGCATGAAGCCGGCCGAGGACGAATTGCATTGCGAGATCAAG
GCGCTGCTTGATCGCGCCAAGGCTACCGACGACCAGGAGCGTAACGAGCCGGAGCTGGACATTCCTGCCGAGATTTCTCGC
CGCGAGAAGCGCCTGGAGGCGATCCAGGCGGCAAAGGCGCGCCTGGAAGGGGTCGGTTCCGGCTGAGGGCGAAATGACA
CCCTAAGCTTTCGGTTCCTTGGGCCAAAGATATTCGCCAGTCAGTAGAATGTGCGCCCAGCCCAATGGGGATATGTGGGGAA
GAAATTCAGGGGGAACATCCAACCCTTCGTTCCGCCGCTCCGTGACGGCATGACCAAGATGGACGGTATTCCAGTAAATGAT
CACCGCAGTCAATAAATTGAGCCCAGCGATTCGGTAGTGCTGCCCCTCTGTCGTGCGATCGCGAATTTCCCCCTGCCTCCCGAT
ACGGAGCGCATTTTTGAGCGCATGGTGGGCCTCTCCCTTGTTAAGACCGATCTGAGCACGCCGCTGCATGTCCGTATCCAGGA
TCCACTCAATAATGAAAAGGGTCCGTTCAATACGACCAACTTCACGAAGCGCAACTGCAAGGTTGTTTTGTCGTGGGTAAGA
AGCGAGCTTGCGCAGGAGTTGGCTGGGCCTGATTTTGCCAGCGGTCATCGTCGCGGCACAACGGAAAATATCAGGCCAGTT
CGCAACGATAAGATCCTCCCGGGCTTTTCCACCTACCAACTTGCGTAACTCCCTGGGGGTCGTATCGGGATTAAATACGTACAA
CCGCTTCGATGGCAGATCCCTGATTCGCAGAACGAGATTGTAGCCGAGCAGGCTACTGGCTCCGAACAAATGGTCGGTGAAT
CCTGCTGTATCGGCATACTGTTCGCGAACATGGCGACCGACCTCGTTCATCAGTAGTCCATCGAGAATATACGGTGCCTCGCTC
ACGGTCGCCGGGATCGACTGACAAGCGAATGGCGCGAACTGGTCGCTTACGTGAGTATACGCTTTGAGGCCGGGAACAGAA
CCATATTTGGCATTGACCATGTTCATGGCTTCGCCATGCCGCGCTGTCGGGAAAAACTGACCATCGCTCGATGCTGACGTGCC
CATCCCCCAGACGCGTGACATCGGCAGTTTACCCTGCGCGGCCACCACAATTGCCAATGCCTGGTTCATGGCTTCGCTTTCAA
CATGCCAGCGGGCAAGGCGTGAGAGCTGCCAGTAATCATGCGTGTTTGTAGCTTCCGCCATCTTACGCAGGCCCAGATTGAG
CCCTTCAGCGAGCAGGACGTTGAGCAGACCGATCCGGTCGCGACATGGAGCCCCGGTTCTCAGATGGGTAAACGCATCTGT
GAAACCAAGGGCTGCATCAACTTCAAGCAGCATGTCGGTAATCCGAACGGACGGCATTCGGCGATACAGATCCAGTATGAGT
GCCTCGGCACCATCCGGCACGTCTGCTGTCAACCTGTCGATCCGCAACGTTCCATCTTCTATGCTACCGTGCGGAATAGTGCCG
TTACGGGCAGCCCGGGCCAGCCGCTTAAGAGCGATCGTGAGTCGCGCCTTTCTGTCTGCCAGCCAATCCTGTGGGTTGGAAG
GCACGGCCAGTTTTGCATTTTCCTGCGCCGCGATCATCGGCACCAGTACCTGCTTGAGGTCACCATAGCGGCGCGAATGAGC
GAGCCAGACATCTCCGGAACGAAAAGCATCCCGGAGGTGAAAGAGTACCGCCACTTCCCAAAGACGGGTATCTCCTTTTTCC
TGAGCTCGTAAATGACGGTTCCATTTGGAGCTGGGCCGCAGGAAACGCCTTTCTGGCGATGCAACACCTTTCATCTCTCCGAT
CGACAAAGCTGCTGCTACCAATGGTCCGGCGACCGGCGCGGCTTCGAGCTTCAGACAGCGCAACATGCGGGGCGCATAACG
ACGAAAGCGATGGTATCCCTGCCCGACATATGCAAGAGGCTCATCGGCTAGCGTGTTGCTGAGTTGAGTCCCTGTCGCTACCA

GTTGAGCGAGCCGGTCCCATGCAACCGAACTGGCGACAGCCATCTCCAGCGGGGTTCCGTCACTGCGGGCCTCAAGCAACG
AAGCTCCCAGCGCGGTGAAGGTACGGATCGTATCCGTGAGTGTGGCTTTAGAGCCGGAAATTGTTTCGTCATGCTGGCGCTT
CGCTTCCCGCCAGGTTTTTCCTACGATCCTGTCATGGGTTTCGACTATGGCATCAGCAATCGCCGCTTCCCACTCCACAACACA
GACGGCAAGGATCGCCCAGCGGCGGTCCGAAGTGATGTCACGCAAACCGTCGGTGAAGTAGCGTTCACCCTGCCGACGCA
GCCGGGCAATGCGATGGGCAGGTATGCTGGCCAAAGCACTATGATTGATATTCAGGGTACGCAGAAATTCGAGCCTGTCGAG
CAAACGGTTAGCAGCAGCCGAGTTGTTACCAACCTCGAAGTTGCGAAGCCAGATGAAACGACTGATATTGCCGGCGAGCATT
TCACTCAGAAGTTTGTCCAGGTGATCGCGAACATCCGCTGTTAAATTTTCCACAATCCGCGTTTCAATCCGCCGCTCAGCGGC
GACCAGAGCATCCGCGCACAAGCGCTCGATTGTCGATACTGCGGGCAGAATGGTGGAAGTTTCCCGACACCGCACAATAAA
ACGATGAGCAAGATCCTCGTTTGATCTGGCATCTTCGGCCTGGCCGAAAGTCCACTCCCGCAGATCACGGGCACCACGGCCC
GTGAAGGTCTTGTAGCCGTAAATTTCGCGCAGCGTGTCCATGTGCTGCTGACGGGTTTGGCGCCGTGTGGCATAAGTGAGAA
GCGCATCAGCCGGAACTCCAAGCTGAGCACCGACGAAGGAAAGGACTTCACGCGGGATCATCTCACCAGGAGCCAGTGCA
CGGCCCGGATATCGTAAGGCACAAAGTTGCAGGGCAAAGCCAATCCTGTTTTCCGGTCTGCGGCGCTGCCTAATGTTTTCCA
GGTCATCATCGCCCAGCGTGTAGAACTTCAGTAGCGACAGTTCGTCCGTGGGCAGATCGAACAGCGCTGCTCGCTGCCGTTC
GGTGAAAATATGGCGTCGTGACATACAAATTCGTCCCTTTTGAAGTATAGTCTGTTTTGGACAACAGCCAGCCCATATAAATCA
GGGCGTTCCGATACAAAAATCCAGGAGGGTTCAATTGGGACATCGTGCCGCCATTTACTGCCGGGTTTCAACAGCGGATCAG
TCTTGTGAACGCCAGGAATTTGATCTGCGAGCCTTCGCCGGCCGTGCCGGCTACGACGTGGTGGGAATATTTAAGGAAACAG
GTTCAGGAACTAAACTCGACCGGGCCGAGCGAAAGAAAGTCCTGGCGCTTGCCCAGTCCAGACAAATTGATGCAATCCTGG
TCACTGAGCTTTCCCGGTGGGGGCGCTCGACGCTCGATCTGCTCAATACGCTACGTGAACTGGAGAACTGGAAGGTTTCCGT
GATAGCCATGAATGGAATGGCGTTCGATCTTTCGTCGCCGTATGGACGAATGCTGGCGACGTTTCTTTCCGGCATTGCGGAGT
TTGAGCGGGATCTCATCAGCGAGCGGGTCAAGTCAGGCCTTGCTGTTGCGAAGGCACGTGGTAAGAGGCTTGGTCGTCAGG
CCGGAGTGCGACCAAAATCAGACCGACTTTTGCCTAAGGTGGTTGCGATGAGGGCCGAGGGACGCAGCTATCGCTGGATCG
CACGCGAGCTCGGTATCAGCAAGAATACCGTCGCTGACATCGTGCAACGACACAGAGCTAACGCTTAGGGTGTCATTTCGCC
CTCAGCCGGAACCGACCCCTGGAAGCGCGCCAGCGTGAAGCGGACCAGGCCCGGGGGCGCAGCGAAGACGATGGCCGCC
GGCCTCGCCATCCGGATGGCTCGGACAAGGGCGGTGGCTCGTACAAACGCGAGTTTGGTGTGCCGGATGACCGTGATCAGG
AAAGCTTCACCGATCCGGACAGCCGGATCATGAAACACGCCGGTGGTGGCTCCGAGCAGAGCTACAACGGGTACACAGCGG
TCGATGCCGAGCACCAGATCATCGTGGCGGCGGAGTTGACCAACTGCGCCGCGGACAGTCAGGCGCTGCTGGGCATGCTGG
CAGCAGTTCAGGCCAACACCGGAGAAATGCCGGCCCAGACGCTGGCGGATGCGGGATTCCGTAGTGAGGCTGTTCTGGCA
AAGGTCGCCGATCACCACGGCGATGTCATCGTTGCCCTCGGCCGCGAGGGACGTGAAGATGCCAAGGTCAATGCCAAGACC
CATCCGCATACGGCGGCGATTGCGGCGAAATTGAAAACGGAGCAAGGCGATGCAGCTTACCGCCGGCGCAAGTCGATCGTG
GAGGCTCCGAATGGTTGGATCAAGGCGGTGATGGGATTGCGTCAGTTCAGCATGAGGGGCCTGGACAAGGTGCAAGCCGA
GTGGAAGCTCGTCTGCATGGCGCTAAATCTGAGGCGAATGGCGTATCTGTGAGGGCGAAGGTTAAATGGGGCGGCTCAAAT
GCACCCCAGTCGTCATAACACGCCGCGCGCCGCAAGATTGGTATCCTTGGCGCCGACGCCTTGCCAATTGCAGAGAGCGCCG
CCGCCATCGTCAGTGCTCTACAGAAAACCGGTCACACGGCTCTGCCGCGCAGACTCCTAGCCTAAATGTGACAGTGGTTGGT
AATCCATGCCGCCCGCAAGGGCGGCGGTGGTGGGCCAATAGATGATTTTCAGAGCCTTACTGCCCGTTGACGCCCAATCCCT
CGAGCGCGAGTCTAGCCGCAGCGGCGATGACGGCCTCGCTGTGCTTGTCATCCTTGTTAGGCGCCCGGGTGTAGACGGCCA
ACACAATAGGTGCGCGCCCAGTGGGCCAGACGACGGCATAGTCATTTGCCGTGCCATACACTCCGCAGGTTCCGGTTTTGTC
TCCGACTGCCCAGTCTGCCGGCACCGCCGCGCGGATGCGGTGGTTGCCGGTCGTGTTTCCCTTTAGCCAATCAACAAACTGC
TGCCGCTGCGGCGCAGCCAGTGCAGAGCCCAGTGTCAGTTTTTGTAAGCTTTCCGTCACGGCGCGCGGCGATGAGGTATCG
CGCGCATCGCCTGGGATGGCGGAGTTCAGCTCCAGCTCCCAGCGGTCCAGACGGAACGTGGTATCGCCGATAGAGCGCATG
AAGGCCGTCAGCCCGGCCGGGCCGCCCAACTCCTTCAGCAACAAATTGGCGGCGGCGTTATCACTGTATTGCACGGCGGCC
GCGGACAGCTCCGCCACCGTCATGCCTGTTGTCAGATATTTTTCCGAGATGGGTGACCACGGAACCAGCGCATTTTTGCCGTA
ACGGATGGGTGTGTCCAGCAAGCCGGCCTGCTGCTGGCTGCGAGCCAGCACAGCGGCAGCAAGAAAGCCCTTGAATGAGC
TGCACAGTGGGAAGCGCTCCTCAGCGCGGTAACTTACAGTTGCGCCTGAGCCGGTATCCATCGCGTACACACCGATGGAGCC
GCCAAAGTCCTGTTCGAGTTTAGCGAATGGTTCCGCGACGAGGTTGGTCAGCGCGGTGGCAGAAAAGCCAGCCAGCGGCC
ATGAGAGACAAGACAGCAGAACTAGACGGCGATACAGTGACATCAACGATATTCCTTGTTTGAAGGTGGAGTTACGGACGG
CCTCAGGAAGTCCTGGCCAAGCCCCGACTATTGGGGCGCGAAGATAGCACCGAGGGTACGCCGGTGTCAACACGGGGTCTG

ACGCTCAGTGGAACGAAAACTCACGTTAAGGGATTTTGGTCATGAGATTATCAAAAAGGATCTTCACCTAGATCCTTTTAAATT
AAAAATGAAGTTTTAAATCAATCTAAAGTATATATGAGTAAACTTGGTCTGACAGTTACCAATGCTTAATCAGTGAGGCACCTAT
CTCAGCGATCTGTCTATTTCGTTCATCCATAGTTGCCTGACTCCCCGTCGTGTAGATAACTACGATACGGGAGGGCTTACCATCT
GGCCCCAGTGCTGCAATGATACCGCGAGACCCACGCTCACCGGCTCCAGATTTATCAGCAATAAACCAGCCAGCCGGAAGGG
CCGAGCGCAGAAGTGGTCCTGCAACTTTATCCGCCTCCATCCAGTCTATTAATTGTTGCCGGGAAGCTAGAGTAAGTAGTTCG
CCAGTTAATAGTTTGCGCAACGTTGTTGCCATTGCTGCAGGCATCGTGGTGTCACGCTCGTCGTTTGGTATGGCTTCATTCAGC
TCCGGTTCCCAACGATCAAGGCGAGTTACATGATCCCCCATGTTGTGCAAAAAAGCGGTTAGCTCCTTCGGTCCTCCGATCGT
TGTCAGAAGTAAGTTGGCAGCAGTGTTATCACTCATGGTTATGGCAGCACTGCATAATTCTCTTACTGTCATGCCATCCGTAAG
ATGCTTTTCTGTGACTGGTGAGTACTCAACCAAGTCATTCTGAGAATAGTGTATGCGGCGACCGAGTTGCTCTTGCCCGGCGT
CAACACGGGATAATACCGCACCACATAGCAGAACTTTAAAAGTGCTCATCATTGGAAAACGTTCTTCGGGGCGAAAACTCTCA
AGGATCTTACCGCTGTTGAGATCCAGTTCGATGTAACCCACTCGTGCACCCAACTGATCTTCAGCATCTTTTACTTTCACCAGC
GTTTCTGGGTGAGCAAAAACAGGAAGGCAAAATGCCGCAAAAAAGGGAATAAGGGCGACACGAAAATGTTGAATACTCATA
CTCTTCCTTTTTCAATATTATTGAAGCATTTACCAGGGTTATTGTCTCATGAGCGGATACATATTTGAATGTATTTAGAAAAATAA
ACAAATAGGGGTTCCGCGCACATTTCCCCGAAAAGTGCCACCTGACGTCTAAGAAACCATTATTATCATGACATTAACCTATAA
AAATAGGCGTATCACGAGGCCCTTTCGTCTTCAAGAATTTATAAACCGTGGAGCGGGCAATACTGAGCTGATGAGCAATTTC
CGTTGCACCAGTGCCCTTCTGATGAAGCGTCAGCACGACGTTCCTGTCCACGGTACGCCTGCGGCCAAATTTGATTCCTTTCA
GCTTTGCTTCCTGTCGGCCCTCATTCGTGCGTTCTAGGATCCTCCGGCGTTCAGCCTGTGCCACAGCCGACAGGATGGTGACC
ACCATTTGCCCCATATCACCGTCGGTACGGCACTGTTGCAAAGTTAGCGATGAGGCAGCCTTTTGTCTTATTCAAAGGCCTTAC
ATTTCAAAAACTCTGCTTACCAGGCGCATTTCGCCCAGGGGATCACCATAATAAAATGCTGAGGCCTGGCCTTTGCGTAGTGC
ACGCATCACCTCAATACCTTTGATGGTGGCGTAAGCCGTCTTCATGGATTTAAATCCCAGCGTGGCGCCGATTATCCGTTTCAG
TTTGCCATGATCGCATTCAATCACGTTGTTCCGGTACTTAATCTGTCGGTGTTCAACGTCAGACGGGCACCGGCCTTCGCGTTT
GAGCAGAGCAAGCGCGCGACCATAGGCGGGCGCTTTATCCGTGTTGATGAATCGCGGGATCTGCCACTTCTTCACGTTGTTG
AGGATTTTACCCAGAAACCGGTATGCAGCTTTGCTGTTACGACGGGAGGAGAGATAAAAATCGACAGTGCGGCCCCGGCTG
TCGACGGCCCGGTACAGATACGCCCAGCGGCCATTGACCTTCACGTAGGTTTCATCCATGTGCCACGGGCAAAGATCGGAAG
GGTTACGCCAGTACCAGCGCAGCCGTTTTTCCATTTCAGGCGCATAACGCTGAACCCAGCGGTAAATCGTGGAGTGATCGAC
ATTCACTCCGCGTTCAGCCAGCATCTCCTGCAGCTCACGGTAACTGATGCCGTATTTGCAGTACCAGCGTACGGCCCACAGAA
TGATGTCACGCTGAAAATGCCGGCCTTTGAATGGGTTCATGTGCAGCTCCATCAGCAAAAGGGGATGATAAGTTTATCACCAC
CGACTATTTGCAACAGTGCCTTATTTCGAGCATTTTCTTGCGAAAAAGCCGACCGGGAAAATTGACATGCGTACCATTCGCCT
GGGGTGTAATAAGGCGATAGACACCGGTACCTACACATTTACCTTCGCGGACAAATCAACGGTTTCAGCCCGCTACACATTCA
CTTATGCATGGGACGGTAAAGAGTGGAAAATTTCCACACACCACTCTTCAGCGATGCCTGAAGGGTAAAAAGCTGTGCAGGG
GGCGGAATATTCACCGCTCCCTGAAGACTAGCCAATAATCGCGATGCCAAGACGTTCCATGAGCAACGATGCCTGGTAGTTGT
CCAACTTAACGCCTTGTAAATCAACGCGCCGAATATCTAAGTCACCCAACTCCGAATTGGTCAGATCGCAATGTGTGAAGTTTG
CTGCTCGCCAGTCGAAAGTCGAAAACTCGCCGCCGGAGAGATCTGAACCACTGAACGTCGCGCCCAGTACCTGGGCCCCCAT
CCAACGGTTTTCCCACAGCTCACACTTTTCCAACACGACTTTCGAAAAATTGGCGTAGCTTAGATTTGTGTTAGTGATATATGC
ACTGCAAAACCAGGTGCGAGTAGTGATCATATTCATAAAACTTGCGCCGCGAAAATCTGCGCCTTGCGCACGACAGTGGCGA
ATTTCAATGCCAAGCGCACTGGCATTGCGAAAATCCGCCATGGATAAATCACAGCTTTTAAAAATGGCATCTTTCAGCATCGCA
CGACTAAAATTGCACCCTTTCTGGCTTTCACGATCATAGAACTGACAGCCGATAAATTCAGTGCCGCTCAGGTCGGCACCTGA
AAAATCACAGTTAAAAAATGTACTATTTTCAATTTTCTCACCGGTGAAGCGGTTTCTGTTAATTTTTTCGCCAACGAGTGCCAG
AGCCATATTTTGTGCCTGTTTTTTTATACAGTAATGGCGTCATGGTAAACCCTGATGAGGTTATGCGTCAAATCCGCCAATATAA
CATCTGCAAATGTGCGTTAAATCTGGTGTTTTTTCAGCAAAGCGCGAAGCTGATGGTAAGTCAGACCCAGTAATTCAGCGGCT
TTTTTCTGGTTAAATTTTGCCTGCTGTAAGCTGGTTTGTAGAAAGTCTTTCTCTTGCTGCTGCTGGAATTCACGCAGATCCAGC
GGTAACCCTACAGACATCGGTTTAGTTTCCGGCGCCTGCGGCTGCGTCTGGTTCTGAAATCCATCCCTGTCGGTGTTGCTTATG
CAGTCTGGTCGGGACTCGGCGTCGTCATAATTACAGCCATTGCCTGGTTGCTTCATGGGCAAAAGCTTGATGCGTGGGGCTTT
GTAGGTATGGGGCTCATAATTGCTGCCTTTTTGCTCGCCCGATCCCCATCGTGGAAGTCGCTGCGGAGGCCGACGCCATGGTG
ACGGTGTTCGGCATTCTGAATCTCACCGAGGACTCCTTCTTCGATGAGAGCCGGCGGCTAGACCCCGCCGGCGCTGTCACCG
CGGCGATCGAAATGCTGCGAGTCGGATCAGACGTCGTGGATGTCGGACCGGCCGCCAGCCATCCGGACGCGAGGCCTGTAT

CGCCGGCCGATGAGATCAGACGTATTGCGCCGCTCTTAGACGCCCTGTCCGATCAGATGCACCGTGTTTCAATCGACAGCTTC
CAACCGGAAACCCAGCGCTATGCGCTCAAGCGCGGCGTGGGCTACCTGAACGATATCCAAGGATTTCCTGACCCTGCGCTCT
ATCCCGATATTGCTGAGGCGGACTGCAGGCTGGTGGTTATGCACTCAGCGCAGCGGGATGGCATCGCCACCCGCACCGGTCA
CCTTCGACCCGAAGACGCGCTCGACGAGATTGTGCGGTTCTTCGAGGCGCGGGTTTCCGCCTTGCGACGGAGCGGGGTCGC
TGCCGACCGGCTCATCCTCGATCCGGGGATGGGATTTTTCTTGAGCCCCGCACCGGAAACATCGCTGCACGTGCTGTCGAAC
CTTCAAAAGCTGAAGTCGGCGTTGGGGCTTCCGCTATTGGTCTCGGTGTCGCGGAAATCCTTCTTGGGCGCCACCGTTGGCC
TTCCTGTAAAGGATCTGGGTCCAGCGAGCCTTGCGGCGGAACTTCACGCGATCGGCAATGGCGCTGACTACGTCCGCACCCA
CGCGCCTGGAGATCTGCGAAGCGCAATCACCTTCTCGGAAACCCTCGCGAAATTTCGCAGTCGCGACGCCAGAGACCGAGG
GTTAGATCATGCCTAGCATTCACCTTCCGGCCGCCCGCTAAATATCTCCTTTTGGGTTGTTAATAAAACATCCAATAAGTTGACT
GTGCGTGAAAAAGAAAGTTTTGTGTGATGGCGTTGAAGATCGCACCGTTAAGCTCTTATGTGGGATGGTGCAGAGCTCGACG
ACTACCGATAAAACGCAACCGCCGCAAACAGACAAGAAAAAGCCCCAACTGATAACAGTTGGGGCTTCAGTATTGTGATTGG
TGGAGCAATAGCACCCTGAACCCAAAACCTTCTCGCTCAACCGGTAGTGGCTGATAACAACTCGTGAGGGCTATTGCGGGTT
AAGCATTTAGCGATGTCTAGGGCCAGACTGGACGTCTGAACGCAAGCCGCTGATACTGTACATAACCACAGTATCAGCGGAG
GATACCCATGTCGCTGGCAAGGAACGCCACGGCGAGTCAATCGCCCACTCAAACAAACGGTTACGAACGCCACCAACCCGA
CCAGACGCTGCTCTACCAGCTGGTTGAGCAGCACTACCCAGCCTTCAAAGCCTCACTCGAAGCCCAAGGTCAACACCTGCCT
CGCTACATCCAACAAGAATTCAACGACCTCCTCCAATGTGGCCGTCTGGAGTATGGTTTCATGCGGGTTCGCTGCGAGGATTG
TCATCACGAGCGTCTGGTCGCCTTCAGCTGTAAACGACGCGGCTTTTGCCCTAGCTGCGGTGCCCGCCGGATGGCCGAGAGT
GCGGCGCTGCTGATAGACGAAGTCTTCCCCAAGGAGCCCATTCGCCAGTGGGTGCTCAGCTTTCCTTTCCAGCTACGCTTTTT
GCTGGCTCGCCATCCCCAGCTGATGGGCCAGGTCTTGAGTATCGTCTATCGTACACTCTCAACTCATCTGATCAAAAAAGCCGG
TTACACCAAAGCCTCTGCACAAACTGGCTCAGTGACTCTTATCCAACGCTTTGGCTCCGCGCTAAATCTCAATGTCCACTACCA
CATGCTGTTTCTCGATGGTGTCTATGCCGAAGATGACTATGGCAAGCAACGCTTCCATCGTGTCAAGGCACCCACTTACGATGA
GCTGAATACGCTCGCTCACACCCTCAGCCATCGCATCGCTCGCTGCATGGAAAAGCGTGGGATTTTGGAGCGTGATGCCGAG
AATACGTGGTTGACACTGGAAGAGGGCGAAGACGATACGCTGACTCAATTACATGGTGCTTCGGTTACGTATCGCATTGCCGT
CGGCCCCCAGCAAGGGCGCAAAGTCTTCACCCTGCAAACCTTGCCAGGGCGTGAGGATAAAGCCGACTCAAGCAGTCGAGT
AGCCAACCATGCTGGTTTCTCGCTACACGCCGGTGTGATGGCCGAAGCGCATCAGCGGGATAAGCTTGAGCGCTTGTGTCGC
TACATTAGTCGGCCAGCGGTTTCAGAAAAACGTCTGGCATTAACCGCCAATGGGCAGGTGCGTTACGAGCTCAAAACTCCGT
ACCGCAATGGCACCACCCATGTGATCTTCGAGCCGCTGGACTTCATCGCCAAACTCGCTGCGTTGGTACCTAAGCCGCGAGTC
AACCTCACACGCTTCCACGGCGTCTTTGCACCGAACAGCAAACACCGAGTTCAAGTAACACCCGCCAAGCGGGGCAAGAAG
CCCGACAAATCGGAAGGTCTCGATACTAACTGGCGTGACAAGAGTCCTGCAGAGCGCCACCGCGCCATGACCTGGATGCAAC
GCCTCAAGCGAGTCTTCAATATTGATATTGAAGTCTGCGAACACTGCGGCGGTCACGTCAAAGTGATTGCCAGCATCGAAGAT
CCGAAGGTCATTGAGCAGATTCTCAAGCATCTGAAACAGAAAACAGCCAAGGCGAATGCCGCCAAGCAGCGTGAGCTGCCA
CCAGAACGAGCGCCGCCACTGACTCCCAGCCTGTTCGATCCATCACAGAGTCGTCTCTTTGACTGACGACCCCAAATCCAACA
CTGCTCAACACTGCCAACTTTTAAACGGGGCGGTGGGGCAGTTTGTATCTCTCGAGCTATCAGGCTAGAGATTTTACCGCCAA
ATCGAACCTTATTAGAGCGGTTTAGGCTGGACCGGCAGTTAAAATTGGGGCTTGAGCGGTAAACGAGTGAGGGAATTTCAG
GTAAGATACTTCGGATGAGGAGCAAAAAGGTGGTTTATACTTCCTATACCCAGTAGTGTTCCCCTGTCGGTTTGGTTGGGGTC
GATGCCCCACTTTGGGCGCGGCGTTGGGCGCCGCAAGATGAAAGCTCTGTGTGAAGGCTTGGATATTAAGACCTTGGATGAT
TTCAATGCCATCACCGAAGAGCAGATCGTTAGCGTCGACAAATTCAGCTACAAAACTGCCGCAAAAGTGCTTGCAGGTATGG
CTGAACATGCCGAGCTATTCAACAAGATTCAATCAATCATCGGGTTCAAACAGCAGGTCACTTCGGGGGATCGTTTTACCGGT
GAGAAGATCGTGATCACCGGTTTTCGTGACGCGGCCTTGGAGAAGCTGATTGAAGCTGAGGGTGGTGAAGTGCAATCATCA
GTCTCGTCGAAGACCACCATGGTGATCGCGGCATCCACTTCCGGATCTTCCGGCAAATTGAAGAAAGTGCACGATCTCAACA
ACAGCGGAAAGGCAAATATCAAACTGATTGATCTGGCCACCTTCCGTAAGCAATATCTGGAACAACCAGCATCGACTGGTTTG
GAGTTTTAATGAGTCACCCACAACTTGAGCTAATAGTCGCTGTGGATTCTAAGTTGGGATTCGGGAAAGGCGGCAAGATTCC
ATGGAAATGCAAAGAAGACATGGCGCGATTTACGCGGATTTCTAAAGAGATCCGCGTGTGCGTTATGGGGAAACACACGTAT
ACTGACATGCGTGACATGCAGTTAGAAAAGGATGGCGCCGAGGAGCGAATCAAGGAGAAAGGAATTCTCCCCGAACGCGA
ATCGTTCGTGATCTCCTCGACGTTAAAACAAGAAGATGTCATAGGCGCTACTGTCGTTCCTGATCTTCGTGCTGTGATCAACCT
GTATGAGAATACCGATCAACGCATTGCTGTCATTGGTGGGGAGAAGTTGTACATTCAAGCTCTTTCATCAGCAACGAAACTGC

ACATGACCATAATTCCAAGAGAGTTCGACTGTGATCGATTTATTCCTGTTGATCCGATCCAGAACAATTTTCACATTGATTCCAG
TGCCAGCGAGACTGTGGAGGCAACCGTTGATGAGACTCAAGAGCGCATTCACTTTGCTACTTACGTGCGTAACAATCAGTAA
CGCGCTGGCAGTGGAAGAACAAGAACCAGAAACGAAGCAGACGGTGTTGGCCGTCTGCTTCGAAACATCATTATTGGCTCG
AGAACTGATCGAGAATACCATTCTTGCGGACGACATCCAAAACAATACAGAAAAGATAGCTTTGCAGTTGTCGGAGCCCCTCC
CCCCAACTTATAATGGGATTATTGCTGGGCTAAACAATGCTGTCGCCATGCGTGGGACAGCAAACGTTGAATCGCCATCAAGA
GCATCCACAGATTTACGTGCAGCCGTCATCGGTTTATGGTGTATAAATGACACCATAGCCGCCCTGAATCAGACTGATAATGCA
GTCTTACAGGACGGCGAGTTGCAACAAACACTTGTCGATTATTACAACAACTTATTGGCTGACATCGTTTGTCGTTTTCAGAA
GACGGCTGCACTGAACGTCAGAAGCCGACTGCACTATAGCAGCGGAGGGGTTGGATCCATCAGGCAACGACGGGCTGCTG
CCGGCCATCAGCGGACGCAGGGAGGACTTTCCGCAACCGGCCGTTCGATGCGGCACCGATGGCCTTCGCGCAGGGGTAGT
GAATCCGCCAGGATTGACTTGCGCTGCCCTACCTCTCACTAGTGAGGGGCGGCAGCGCATCAAGCGGTGAGCGCACTCCGG
CACCGCCAACTTTCAGCACATGCGTGTAAATCATCGTCGTAGAGACGTCGGAATGGCCGAGCAGATCCTGCACGGTTCGAAT
GTCGTAACCGCTGCGGAGCAAGGCCGTCGCGAACGAGTGGCGGAGGGTGTGCGGTGTGGCGGGCTTCGTGATGCCTGCTT
GTTCTACGGCACGTTTGAAGGCGCGCTGAAAGGTCTGGTCATACATGTGATGGCGACGCACGACACCGCTCCGTGGATCGGT
CGAATGCGTGTGCTGCGCAAAAACCCAGAACCACGGCCAGGAATGCCCGGCGCGCGGATACTTCCGCTCAAGGGCGTCGG
GAAGCGCAACGCCGCTGCGGCCCTCGGCCTGGTCCTTCAGCCACCATGCCCGTGCACGCGACAGCTGCTCGCGCAGGCTGG
GTGCCAAGCTCTCGGGTAACATCAAGGCCCGATCCTTGGAGCCCTTGCCCTCCCGCACGATGATCGTGCCGTGATCGAAATCC
AGATCCTTGACCCGCAGTTGCAAACCCTCACTGATCCGCATGCCCGTTCCATACAGAAGCTGGGCGAACAAACGATGCTCGCC
TTCCAGAAAACCGAGGATGCGAACCACTTCATCCGGGGTCAGCACCACCGGCAAGCGCCGCGACGGCCGAGGTCTTCCGAT
CTCCTGAAGCCAGGGCAGATCCGTGCACAGCACCTTGCCGTAGAAGAACAGCAAGGCCGCCAATGCCTGACGATGCGTGGA
GACCGAAACCTTGCGCTCGTTCGCCAGCCAGGACAGAAATGCCTCGACTTCGCTGCTGCCCAAGGTTGCCGGGTGACGCAC
ACCGTGGAAACGGATGAAGGCACGAACCCAGTGGACATAAGCCTGTTCGGTTCGTAAGCTGTAATGCAAGTAGCGTATGCGC
TCACGCAACTGGTCCAGAACCTTGACCGAACGCAGCGGTGGTAACGGCGCAGTGGCGGTTTTCATGGCTTGTTATGACTGTT
TTTTTGTACAGTCTATGCCTCGGGCATCAAGGCGAGCTCAGAGACCATGGAAAGCATGTTCTCGGACTTACGTAGCAACTCGT
TTCTTTTCGCAGGTTGAGCCACCTCCGCGCTTCATCAGAAAACTGAAGGAACCTCCATTGAATCGAACTAATATTTTTTTTGGT
GAATCGCATTCTGACTGGTTGCCTGTCAGAGGCGGAGAATCTGGTGATTTTGTTTTTCGACGTGGTGACGGGCATGCCTTCG
CGAAAATCGCACCTGCTTCCCGCCGCGGTGAGCTCGCTGGAGAGCGTGACCGCCTCATTTGGCTCAAAGGTCGAGGTGTGG
CTTGCCCCGAGGTGATCAACTGGCAGGAGGAACAGGAGGGTGCATGCTTGGTGATAACGGCAATTCCGGGAGTACCGGCG
GCTGATCTGTCTGGAGCGGATTTGCTCAAAGCGTGGCCGTCAATGGGGCAGCAACTTGGCGCTGTTCACAGCCTATCGGTTG
ATCAATGTCCGTTTGAGCGCAGGCTGTCGCGAATGTTCGGACGCGCCGTTGATGTGGTGTCCCGCAATGCCGTCAATCCCGAC
TTCTTACCGGACGAGGACAAGAGTACGCCGCAGCTCGATCTTTTGGCTCGTGTCGAACGAGAGCTACCGGTGCGGCTCGACC
AAGAGCGCACCGATATGGTTGTTTGCCATGGTGATCCCTGCATGCCGAACTTCATGGTGGACCCTAAAACTCTTCAATGCACG
GGTCTGATCGACCTTGGGCGGCTCGGAACAGCAGATCGCTATGCCGATTTGGCACTCATGATTGCTAACGCCGAAGAGAACT
GGGCAGCGCCAGATGAAGCAGAGCGCGCCTTCGCTGTCCTATTCAATGTATTGGGGATCGAAGCCCCCGACCGCGAACGCCT
TGCCTTCTATCTGCGATTGGACCCTCTGACTTGGGGTTGATGTTCATGCCGCCTGTTTTTCCTGCTCATTGGCACGTTTCGCAA
CCTGTTCTCATTGCGGACACCTTTTCCAGCCTCGTTTGGAAAGTTTCATTGCCAGACGGGACTCCTGCAATCGTCAAGGGATT
GAAACCTATAGAAGACATTGCTGATGAACTGCGCGGGGCCGACTATCTGGTATGGCGCAATGGGAGGGGAGCAGTCCGGTT
GCTCGGTCGTGAGAACAATCTGATGTTGCTCGAATATGCCGGGGAGCGAATGCTCTCTCACATCGTTGCCGAGCACGGCGAC
TACCAGGCGACCGAAATTGCAGCGGAACTAATGGCGAAGCTGTATGCCGCATCTGAGGAACCCCTGCCTTCTGCCCTTCTCCC
GATCCGGGATCGCTTTGCAGCTTTGTTTCAGCGGGCGCGCGATGATCAAAACGCAGGTTGTCAAACTGACTACGTCCACGCG
GCGATTATAGCCGATCAAATGATGAGCAATGCCTCGGAACTGCGTGGGCTACATGGCGATCTGCATCATGAAAACATCATGTTC
TCCAGTCGCGGCTGGCTGGTGATAGATCCCGTCGGTCTGGTCGGTGAAGTGGGCTTTGGCGCCGCCAATATGTTCTACGATCC
GGCTGACAGAGACGACCTTTGTCTCGATCCTAGACGCATTGCACAGATGGCGGACGCATTCTCTCGTGCGCTGGACGTCGAT
CCGCGTCGCCTGCTCGACCAGGCGTACGCTTATGGGTGCCTTTCCGCAGCTTGGAACGCGGATGGAGAAGAGGAGCAACGC
GATCTAGCTATCGCGGCCGCGATCAAGCAGGTGCGACAGACGTCATACTAGATATCAAGCGACTTCTCCTATCCCCTGGGAAC
ACATCAATCTTACCGGAGAATATCGTTGGCCAAAGCCTTAGCGTAGGATTTCGCCCTCTCCCGCAAACGACCCCTAAAAGCCG
TTTCCTCTGTATAAAAGATCAGCTAAATTATGTGTATTGCACAATACATATATGTGAGGTTAGCAGTGAATTTGCCTACGCCCGA

AACCTACGATGAACTTCAGAGAGCCTACGATTTTTTCAATGAGAAGCTATTCAGCAACGAGCTGCCGCCATGCCTGATAACGT
TGCAGCGTGAGAAGCGAACGTATGGCTATTGTTCCTTTAAGCGTTTCGTCGGCCGTGAGAGTGGGTACACGGTAGACGAGAT
CGCTATGAATCCGGTGTATTTCTCGATCAGAACCATAAAGGCCACGCTTTCAACACTGGTGCATGAGATGGTTCATCAGTGGC
AATTCCATTTTGGCGAGCCTGGCCGCCGTGGCTATCACAACAAACAGTGGGCGGCCCGGATGGAACGGGTAGGACTAATGC
CTTCTGATACCGGCGAACCGGGAGGCAGGAAAGTGGGCCAGAGCATGACCCATTATATTATTGCCGGTGGCCCTTTCGATATG
GCCTGTGATGAACTGCTGACAGGCCATTTCCAGCTTTCCTGGATGGACAGGTTTCCGCCTTACCAGCCTAAGCCTGGCGCTGT
GCTAAGCCCTACAGGAAAAGGCTATATTGACGACGAGGAAGATGATAGCGAACACGAACAGGAGGTGGAGGAAGGGCGCG
ACCCGGTTGAACTCGACGACGAGATCATAGAGGCCATGCGATTTGTAACCCCACCGCCTGAAGCACCGGTGAACAAAACAAA
CCGGGAAAAGTACAGCTGCCCGGTGTGTCATATCAATCTCTGGGGTAAACCGGGGATAGTGGTTTACTGTGGTGGCGAGCAC
TGTAATAAAGCCGCGTTAGTAGTCTTAAAATAAAGTCCTTTCGGACTTTATTTTTTTTCCATTTCCGAGGTCGTGATGTTATTAAT
GCTGTACTTCGCGGCTTCTTTTAAAACAGTTTCAGCAAGGCTTGCTGGTATCCAGACCTGAACTAATTTTAATGGTTCGCCGTT
CTCGGCTTTAAGAGTGGTGTTCTGGTACAAATCCCAGATTCGCTTAACGGTGCTGGAAATGTTTTGCTTGGAACGGCCTACTC
GCGTGGCTACGTCTGATGATTTCTCACCTTTGACAAGCACGGAATAGCCAATATCTGTTGTGATGTGTGCAAAGGAAGCCATT
TGCGGCAGCAGCTGTTTCCATTCTGTTTCTGAAATTCTGTTTTTCTGAGCCATCTGTGGCGCCTCCGTAGTTTTGGTTACAGAA
AGGATATACTCAGAATAAACAGGGGTCAATACAAGTACGATTTTTATAAACTTTATTTTATTTGAGGGTGAGGCCCGGTGCGGC
AGCAGCGCGGGCCTCGATGGTGCCGCGAAGGTGCTGGCGCCATGCTCGGATTAAAACATGAACCGTGAAGAACTGCGAAAC
TTGTTTTCGCGGTTCTGAGGGGTTGACCGAGCCGCGAAGCGGCGCTGGTAAGCGATGATATGCACATATCCACAGGCATATTT
TTAAAAGGGGCACTGTTGCAAAGTTAGCGATGAGGCAGCCTTTTGTCTTATTCAAAGGCCTTACATTTCAAAAACTCTGCTTA
CCAGGCGCATTTCGCCCAGGGGATCACCATAATAAAATGCTGAGGCCTGGCCTTTGCGTAGTGCACGCATCACCTCAATACCT
TTGATGGTGGCGTAAGCCGTCTTCATGGATTTAAATCCCAGCGTGGCGCCGATTATCCGTTTCAGTTTGCCATGATCGCATTCA
ATCACGTTGTTCCGGTACTTAATCTGTCGGTGTTCAACGTCAGACGGGCACCGGCCTTCGCGTTTGAGCAGAGCAAGCGCGC
GACCATAGGCGGGCGCTTTATCCGTGTTGATGAATCGCGGGATCTGCCACTTCTTCACGTTGTTGAGGATTTTACCCAGAAAC
CGGTATGCAGCTTTGCTGTTACGACGGGAGGAGAGATAAAAATCGACAGTGCGGCCCCGGCTGTCGACGGCCCGGTACAGA
TACGCCCAGCGGCCATTGACCTTCACGTAGGTTTCATCCATGTGCCACGGGCAAAGATCGGAAGGGTTACGCCAGTACCAGC
GCAGCCGTTTTTCCATTTCAGGCGCATAACGCTGAACCCAGCGGTAAATCGTGGAGTGATCGACATTCACTCCGCGTTCAGCC
AGCATCTCCTGCAGCTCACGGTAACTGATGCCGTATTTGCAGTACCAGCGTACGGCCCACAGAATGATGTCACGCTGAAAATG
CCGGCCTTTGAATGGGTTCATGTGCAGCTCCATCAGCAAAAGGGGATGATAAGTTTATCACCACCGACTATTTGCAACAGTGC
CGGTCGCCGGGAGTCAGCAGATCGACGTCAACGCCGAGCAGCGATTTCAGTTCTTCTTCCAAATCGCCCAAGTCCAACAACG
TGGCACCGGGCAGCGCATCGACCAACAGGTCGAGGTCGCTGCCATCCCGGTCGGTGCCATGCAGCACCGAGCCGAAGACG
CGCGGGTTCGCGGCGCGAAAGCGGCCTACCGCTTCACGCACTGCGCTTCGCTTCATGTCAAGCACAACAGACGGTCGCATG
CGCATCCTTTCTTATCGAAACTCGTTGAGATGATATGCAATCAAGAATAGAATTTCAAGAACTCACAAAGTAACGCGGTGGTTA
ATATCCTGTACCCACGGATTGCCCTTAGCGCTGCCTATATCGGCTAAAGCACTCCGGTAGCTTGATTCACCCCACGGCCACGGC
AGGATCTTGGCCGTCGCAAGCGCCAGGGGAAAATCTTCAGCTGCAAGCCTGAGTGATTTCATGTGCGTGTAATCCATCGCCC
AGATGATTTTTGTGAAGAAGAACTCGCGCTCGTTCATGTCCGGGCGCGCGTCCTGGCCCTCCGTGCCAACGGCCAGCAAGTA
ATCGGCCTGAATTGGCAGTATCAGCGCGCGTAGTAAGTCATGGATCGCCGGTTGCGGCAGTCTGCGCGCCAGATTAATTACCC
GGTCGAACTTCAGTTTATCCTGCTTGCGCTTGTCGGTCTGCTCCATCAGATTTCATGGCCCCCTTCTTCATGCTCATGCTCATGG
GTGTGTTCTTTTCCGGTATGGCTCTGTTCCGCCTGAGACGTCTGCGGCATGGCGTAATCGTCGTAAATGCTGCTGTCAAAGTCG
TAGTCTGATGCTTCGGCATAATGCTCGTAATCGGCATACTCCTGCGCGCTCCACTGCTGATCGTCGGCAGCAGCATAATCATGG
GCCAGCTCTGCATCATTTTGCTGTGCTTCATGACGCCGCAGGCCAACGGAATCATCCATAGGGTTCTGCTTAAGATGAAAGGC
GTCCTCTGCGTTGCTCACCGGCTGATAATCAGTGCCGGTTGTCATGTTATGTTCATCGGGTTTCTGGTTAAACGCCATGCTTTCC
CCCGTGGCTTCTGGCAGACCTTTTTCAGCTGATCGGGTTTCTAAACTGGTATCGCGGCCAATATCCTTAAACCTGGCCTCAAGC
CCAAAGAAACGGTCAATTTCTGCGGCCGTGGTTTTCGGGCTGTCGCGGCTCACGCTCGATGCCAAAGATTTTTTATCGTCGGT
AAAAATTTCCACCTCATGACGCGCACGCGAAATACCAACATAAAAAACGTCCTTAGAAGTGGTAAGCGATTTGGTATCTATGTT
GAACAACACGCGATCACAGGTAAGCCCTTGGGATTTGTGGACGGTGGTTGCATAAGCATAGGAAAGATAAGAAGCCTGTTTT
TTGTCCAGCTCAACCGTGCGCCCTTTTTTGTCCTCAAGCGTCAGTTTTTCACCCTCCACGGTTTTCACCGTGAAGCGGTCGCC
GTTGGCAACGTCCAGCGTTTTATCGTTACGCGTTACCATAACCTTATCGCCCGGCGCCAGTTCGGCGCTGACTGCCTGGTATAC

AGACAGCTTGGTGTGTGTACGCGGGCTGAAAGCGATCTGCTCACCGCTGCTGCTTTCAACCGTCAATTTGTTGCCCGGCCCG
GTATCAAGAACCTGGTAAGACTCGCCCCGCTTCATACCATTTTTGTAATCCTGTTCGGGGATAATGATTTGCCCTTTACTGAAAT
AACGGCTGTCGCGGCGTTCCGCCTGTGTCGAATCCACGCGGTCAAGTAGCGTGAACGTTTCGCCGGTTCCGGCAAGCCCCA
GATTGCCCCGGATGTAGTCATTGAGGGTTTTGCGTGAGGCGTTCGTACCAGAGATTATCAGGGTGGCATCCTGTTGTTCTGAG
GACAGAGACAGGTAGCGATCGGCAAGTTGAGCGAGTCGGGGCGCTTCTTCCTTCAGTTCGTTCACGCCGGTGATATTTTTCA
GGGCGCGCGCGGCATTACCTTCAGCGGCATACTTAACCGCCTCAAGCAAAACTTCATTCTTCTGTCGCTGAATGTCTTTCATGT
AGCTGGTCTGCATATCTGCTTTAATCAGCTGCTCAAAAGGCTTACCGGCTTCTACCGCTTTCGTCTGTGACGTATCCCCCAGGA
ATACCGCGCGAGCGTTATGCTTCTCGATCACCTCCATCAGCTGTTTCATCTGTCGGGCGGGTATAACCCCGGCTTCATCAATGA
ATACGACTGATTTTTCATCCAGCTTTTTATCCTTCGCTTTGAGGAAAGCGGCAACGGTGCGGGCCGGTAATCCATCATCTTCAA
GCGCTTTTTTCTGTGTCCCATAGGGGGCCAGCGCCGTGACCTTCAGCCCTTGTGACTCCAGCAGCTCTTTAGCGGCCATCGTC
ATATAGCTTTTACCGGTACCGGCGTAACCATGTGCGGCCACAAACCGATCTTTGCTCGTCACAATTTCTGTAACCGCGCGCATC
TGCTCCTTCTTGAGGGTTTTCCCGGCAAGCAGCTGGCCTGCAATCTCTGCGGTCAGCTGTCGCGGCATCTGCCCCCGGCCGC
GTGATTCGATAGTCAGAATGGAACGCTCAAGGCGAATACCCTCCACGGTAGTGACGCGGTGGCTGGTCTTTTTAAGCCTGCC
GTTTTTAATACCATCATCTACCGCAAAACGGGCTTTATCCGCACGCATCCCGCTATTCGTCAGCGAGTCGATCCACTCTTTGCGC
GTCAGAGTTTCGGCCATAACTGAAGCACCGACCTTCAGAGTTGATTGATACCGGGCTTCGCCCTCGATGATGGCGCCCTTCTG
TACCGCCTTCAGGTACGCTTTTTCAACATCGGCTATTGTGGCATGGCCCAGCACCTGCTTATTAGCGATTTGAATCAGCTTCTG
GCGTTCAAAGCTGGCATCGCGCTCTGACAGCGACTTAACTGCAAACTGGATAGCCCGGTCAGCTTTAACCTCCGGGCTGGTA
AAATCCGGGGCCATGTTGCGCGCTATATCAGCCTCCAGAGGTTTACCGTGTCCCTGCCATTCACGGTTATCAAAATCAATGCCG
AGCGTTTTGGCGCGGCTGGCCCATTCCTGGTGAATTTCTTCACGGGAATGCTCTGTTTTCTTTTCACGCGTAGCCATCGAGAC
GCGGCTTTTCGTCTGAGCATCGGCGGTTTCCCGCGTCAGACCCATTGCAGCGAGTCCCTTTTCAATTTGCTCCGACCGGCGG
GAAAAAGCGCGAATCTGTTCATCTGAAAAATGGGCCATATCGAACGTGTTATTTTTGCTGTTGTAACGCAGCTCATAACCGGCT
TTGGTCAACTCCAACGCCAGCTCCTGTTTGTAAACATCGCCCAGGTGCATTTTGTTACGCATCAGCTCATCATTTTTGAGCGCG
CGCCACTGGCCGTCCTCGCGCTGGGTCATGTTCATGACAAAAGCGTGTGTGTGCAAATCAGGATCTAGCGCCCTGGAAGTTT
CGTGGCGGAAAGTAGCGACGACAAGGTTATTGGTATTCTGGGTTACTGATTTCCCCTGGCGAGTCGTCCGGGCCTGCGCGAG
TTTTTCAGCTTCACGCACAGCAGCGGCAACAGCTTTTTCATGAGCCTCGATAATGGTTTTATCGCCGTGTATCAGCGCCTGCAT
GGATACCCCTTTAGGCGCTGAAAACGTCAGGTCGTAGCCCAGACGCTCTTTTTTGGCATCACCCACGTGTCGCTGCATATGCG
TGAAGGTATCTATCTCTCCGACAAGCAGCTCTTTAAACCGGGCTGATTCAACGTCCCCGGATAAGCCGAGGGCTTCAGCTCCG
GTTCCCTGCCAGGACGTGAATGATGAATCCTTACTGTAGTAATCATCCTTTGCATCAGAGTAGTAGCCCACAACGCTAGTGACG
TTCTGGCGGGTAATCGTGGTTATATCAAGCATCAGATCTCCCTCAGTTCAATGCCAGGAACAGGGTTTTTGCGATGGTATTTAA
CGTGTTTAGCCTTGAACTTAGCGACGGGCATATCACCAGGCAACGCCAGATAGCCGGTGAGGTTTGGCAACATTGATATTTCG
GTAGGCGTTACGGCACGAACAACTTTAACGTCGCGGCGTTTACGGACAATCCAGGGCTTCTGAGGATCGGATTCTTTACGCT
CAACTTCGCCTTCTATCTCACCGAGTGAGCGCGACATTTGATCCAACGTTTCATCACCGAGACGGCTGCCGCCCAGCACGATG
TTAGAACGCATGTTAGCCAGAATTGTCTGAGCCATATCCCGACCATAAACCTTAACCAGCTGAGAATAGGTTTGATAGCCAGCA
TAAACACACAGACCGCTTTTACGCCCTTTGGTCAGTGCATCGTTGAGGTTTGGCAGAAACTGGAGTGATTCCAGCTCGTCAAT
AAATACATTAATGCGGCTTTCTTTTTCACCCATACCCAGCACGATAGAAAAAATCGAATCCAGCCAGCAGGAAATTAGCGGATT
AAGTGACCTTTTCATTTCTTCCTGCCAGGTGATAAACAGGGTTCCCGGCTTTCCATCATCAAGCCAGTCACGCAGGGAAAAAT
TACCTTCCGGCATTTTCAAATGTGGGGCAAGATTCTTACTGAGAACAAATCGCGCGCTTCCAACTGCTTTTTCAGACCCGGAA
AAAATAGCTTCGGCAGGCGTCCCCATTAAAAATTCTTTTAATTTTTTCTGGTCAACGTTACAGGCCCAGTGAATAACTTCTTCC
ATAGTTACTGTGCTGTATAGGCTGTGAAGTTTTTTCGAAACTTCACTAAAAATAAGACGGCCATAGCCGAACCATTCTTCAGTA
GCCATATCAGGGCTTTCCTGAACAATAGAGTTCACTAAACGCTCGTAATCATATGAACGGCGAATTTCATTGAAAAACACCCAG
CCTTCAGTGCGTTTATCATAGGCGTTTAAAATAACATCGCCGGGACGATAGAAATTCTTTAAGAACCCCCCATTTGGATCTAAA
GCAATATTTTTGCCGCCTCTAATGATGCTCTTAAATAACAGTTCATTGAAAATTGTGGTTTTACCAGTACCGGTTGTACCGGCAA
TCGAAAAATGCAAGTTCTCAGCGTATGTAGGTATGGGGATATTAGCCACGGTTAACTGGTTGACACCTCTTTCGCGTGTTTTAT
CAGCGAGTGTTCTGGCGCGAACAAGCTCTGTACCACGATAAATCTTTTTGAATCTTTCGCCTTTAAACACGCGTGATTTATCAT
AAATGATAAAAGCGATCAGACCGCCAACACCAATAAACCAGCCAGCAATTAAAGCTGACCATAAAGGCCATAGCGAAAAAGT
ATTCTTAACCAGATACGGAATCAGGTATTTAGCCGTGGATGGATCAATACCGTAGGTAAATTTTGCAACTAGAAACCATACCATC

ACTGGAGGCAAAGTAATTGCAAATAAAAATGCTAAGCCTCTTTCTCTATCGTCCATTTCAGCGCTCCTTTTTTGGTTCCCAGAC
TTTGTAGCCGTTACGTTCAACCTCTGCTTTTGCCGCTTTGGTTTTGCCCGGTTCTGCTATCGAGCGCAGGAGGATTAGCGTTTC
AATAGCGAGTGATTCATGCAACATCATCTGTCCTGCCGGTGGGAATTTTACGCCAGATAGCGTTTCGGTTATTGCCTTCAGCTC
ATCGCGCAGTGGGCCAAAATCCGCATCTGAAGCACGGTCAAAAAGATAATCCAGTTTGCGATTTACGTCGCTCAGCCGGTCG
GCAACTATTTTCAACCCGGACTCCCGATCACCTGGGCCAGCTTCAATGCAGCGCCGCAGATAATCTGACCGATTACCTCCTGA
AACCAGGTCTATATAGGCCAAAAGTTCATCTGATACTTTTGCGGTTATTATTGGCATTCAGTCCTCACATTGTGCATTTCTTAAAC
AAAAAATTGGGATCTAACAAGCTGAAATCTTAGTATTACCAAAGTAATAAAGCAAACTCATTATAAAACAATGGGTTATTGGGT
GTTTTTAATACCTAATTATTACCGAATATTGACGCTATTTATTTTTTTATCTTTTAAATCAGTACGATAGCGTGATTTATCGCGCTGC
GTTAGGTGTATAGCAGGTTAAGGAAAAAAAATCATCTTTTTTGGTAGGAGCGACCTCCGTAGGTTAAGGGTCATTTGGCTAAA
AAGCGTCCTATTCTTTGATGGTCATGCTTGCATGACCATCTGAGCAACCAAAAACTACAGATAAACTACAGAGAAACTACAGAT
AAACTACAAAAAACGATTTACCTTAGCGTTGTCAGACTACTAATAGACTACAAGGAAACTACAAAGAAACTACAAAGAAACTA
CAAAGAAACTACAAATAGACTACTAAAACCGTGGCAGACTACTAATAGACTACAAGAAAACTACAAATAAACTACAAAACTGG
ATTGACCCCTTCTTACGAGTGTTGTAGAGTCATCTTCATACAACGGAGGGGGTTATGAATAAACCAGCAGATCTGAAACCCCG
CAACTTATCGGCTGCTGTCAGATTGCGCCTAAATGAAATCGAGAACTGGCTGGACAGAGGGCTAACGCGGCATGAAATTGCT
GAAATCCTCGACAGCGAATACAGCTTTTCGGTAACAGCCAAAGGGCTTGAGATGGCACTGTATAGAACGCGGCAAAACCGA
AAAAATGTATTGCACAATACACATGATAAGAGTAGCGCGAAGGGTGCAGCGGAAAGTGTATTGCACAATACACAACCGTCTG
AGCCTGAAGCGCAGGAAAGTGAAAAAGCAGAGAGTCCCGGCATTATTGATAAAGAGTTCTTCAATAAAATCGGTGAGGATTT
CAACCCTAAGAAGTTCAACAAAAAATTCTGAGGTGATTTATGAAAGTAGCGGTAATTAATTACAGTGGCAGTGTTGGTAAAAC
ATTAATTTCATCTTACCTGTTAGCCCCGCGCCTGACTGGTGCAAAGTTCTATGCGGTAGAGACTATCAACCAGTCTGCTTCCGAT
CTGGGTATTGAAAATGTGACCAGTTTTAAAGGTGACGACTTCTCACGTTTGATTGAGGATATTGTTTTTGAAGATGCAGGCAT
TATTGATATTGGCGCGTCAAACGTTGAAGCGTTCCTGATGGCTATGTCTCGCTTTGACAGTGGCGCGAACGAATTTGATAAATA
TGTAATCCCGGTGACGCCGGATAATAAGGCGATTGATGAAAGCCTGAAAACGGCACACACGTTAAGTAAAGCGGGCGTGAG
CAGCAAGAAAATTATCTTTGTTCCAAACCGTATTAGTCCAGACAGTGAAGTAGAAGATGTGCTGGCGCCGGTGTTTGAGTTTG
TCAAAGAAACGAAGATTGGCAAAATAAGCAAGAAGGCTGTTATTTATAACAGTGAGGTTTTCGAATATCTGGCGTTTCACCGT
ATCTCATTCGAAGTATTGACCGCTGAAGATCCAGAAGAATTCAAATCCCGTGCAAAACAAACAACCGATGCTGACGAGCGCA
AAAAACTGGCACGCCGTTATACATACATGAAACAGGCGATTCCGGTAAAAGCTAATCTCGATAAAGCATATGCGGCTTTAATGG
GAGAATAAAATGGAAAAGCAGCCGGATAAATTAGAAGTTCTGATGGACTGGTTTTTAGGTGACGCGAAGGAAATCACCGCA
ACTCAGAAAGAAATGACGCAGAAACTTTCTGAGCTTTCGGAAAAGCTGGCAAAAGACACCGAAAGTTTAGGAGAGACGGC
AGACTCTTTTAAACGGGCTTTAGTAGAAAACCAGCGTTCAATTAGCCTGGCAATTAGTGATGATGCTAAGGCGCGCGAGGAA
TTTCTAACTAAATTCCGCCGCGCGCAGGCGTCCAGTGCTGAGACGTTTACCCGTCAGATCCTTTTTATTACAGCTGGCTGCACC
ATCGTGGGCGCCGCAGTAGGCGCCGCGATAGCGATACTTTTACTGAGATAAAGCAAACCGGGCGTGTCCCGGTTTTTTTGTC
AAGCGGAGCGCGGAGGCCGAAGGCCGGAGGCATTAGTGGCCGCCGCCCGCGTAAGCGGGGCGAGACGGAACCGGCTC
GAAGCGCAGCACGGCAGAACGGCCCCGCAGGGGCAATGCCCGTTTTAATTCATCGTGACAGTCGCGCGTGACCATCACGGG
GAGAAAAATAATGAATGACCGACAGCGAGAACTGGCCCGTATACGCCAGGCCCGCCGCCGCGCGCGGCTCAAGGAAGAAG
GCACAAGCGTGACAGTCACGCTAACAAAACAGGAAGAAGCAATGTTGCAGGAGCTGTGCCGGGTTCGCCGTCCTGGACGA
ACGCCTTATTCAACGAACGAATTTTTCCAGCTGCTGCTTATCCGCAACTGGCAGCAGTGGCAGGAGCAGAAGGCACAGCTGG
GAAAATGCCAGGCTTGCGGAAAGCTGAAAGCGGAGGGGGGGTGCGAGGGTGAACGGAAAGGCGAAACCTTTAACTGCTG
GCTTGCCGTCGAAGCCAATGAACTAAATTTGTAGTGTATTGTGCAATACACATTTACACAGAAACAAAAACCACCGGCAATTC
CTGGAACCGGATACCTACGGCTATTCCTGGGTGAACGGTACTTTTTGCACCTGGGTGCGCTGAAAAAGCTGAATATGCAGGG
TGACGTTCGCGTGCTGTTCTGCTTTGTAGACTGAATGCGCCAGCTATACGCCTGACTGCTTAAACCTGGTAAAGTTCTGCAACC
GGCACTGACCGGAAAGCAAGGCAGGGAAGACCTAAGCCAGAAACCTTGACTGCTCCCCGCCCTTCAGGGCGGGGATTGCG
GATCATGTTCTTCTCTTTCAGGGATTCAACGCAGACAAGAAAGGCTTTCAATTTCCTATACGTGAACGGCCGCGCAGCGGAAA
GAAACAAGCCCGGTCAATCCGGGCTTGTTTCTTTAGGCGGCTCAGAAATCGCCTAAAGGCCCGGCTTGGCCGGGCAGTCAG
TGCTATTTAGTTTGTTGCAGCAGCTGGCTTAATTTTGCCGCCAGTGCATACGTCGATTGGAAAGCGCCTTGCAGGCGCTGATT
AGGCAGCTGGTTGAACGCTTCGAGGCAGGCGCGCAGCAATAATTCTTTCTGAGATTCGACTTCTTTTTTCAGTTGGGAAGGG
GTGGTAACTGTAGTCATGCTTGCTCCTTAGTGAGCCGATATCGGCAATTTTTCGGGTGGCGGTGTTGCCTCCCGATGATTTAAT

TATCGGTGATTATGCTTTTAAAGTCAATACAGGTACGGAATTTATTTACCTGTTTTTATGCCCGTCAGGGCATGGAAGGCGACC
GCGCCGGACTCCACCGGACACCGGCCGCAAATCGCCGGAAACTGCGGGACTGACCGGAGCAACAGGCCAACCCCCCTCCC
TGCTAAGCCATAACCCAGCCCGCCGCCACGCAGCTGCCGCACGTCCCCCACGGGGGTGCGCAGTGGGCGCCGCGCGCCTGC
GCGCGGGTACGGCGGCCCGCCTGCGGGTCGCGGCGCCGTACTGCGAGTTAGCGGCCGCCGCGCGGCCGGTTACGGGGGA
CACCGCACAGTCACGGCCAGTGCCCCGCTGAGCTGCACAATCCACGGATAACACAATAGCGCACTGGCAAAGGATGCCGAC
GCCTGAAGGGCGTGGGCACCCCGAAGGGGCGGGGCGGCCGCTTGCGGCCGGGCGAGTCCGGCGCAGGGTGTGGCCTGC
CAAGCGGAGCGCGGAGGCCGAAGGCCGGAGGCGTTAGCGGCCGCTGCCCGCGTAAGCGGGGCGAGACGGGAACCGGCT
CGATGCGCAGCACAGCAGAGCGACCCCGAAGGGGTAACGCCCGGTGTGGCATCAGGATTTAGTGCAATGGCAGAACATGA
GCTGGAGAGATCACCGGCAAGCAGCAGCAAAGGGGCGGCACAGCCGCCCCGATGGCTGTTTGCCGATACCGGCGATTAATT
AGAGCGGTGTTTAATATCCCCCGCGTTGCGGGGGACTAGGTTTCAGCAAGTCATGTTAAATACGTGTCCATCATGTAAACTGA
AATCCCCAATAAACAGATCCCGCGCATAGGCTACGATGTCAAAATATCGGGCTACGGATTCCGGAATATCATTCAGTAGACCGC
TATCATTCAGGTATTCCTCTGCAAAAGTTTCTTCGTCCTTAGCTTCGCCCATATAGGCATCTCTAAACAGGTCGAAATCAGTGCT
ATTAAACAGATCAACAAAGGCCACAAACGCCGCTTCGTTACCTTCCTCGCGGGCTTGTTTAAAGCCGTTAATAAAATCCCAGT
TGATATGGCACTCTGACGCCATACCAGACGGAATACCCTCCCAATCTTGGAACATAAATTCTGGATCAGCCTCATTTGCGTGTA
ACTCGCGGCAGCGCTCGTAAAACTCCTCTGAGCTATCAAAATCGGTCAGATCGAGCCAGGCTCCCGCAATGCTTCCGCAGTTG
TATTTATGGTAAGTGCCAACATAAACAGAAGGGGTCGTAATATCAGTCATGGTGTACTCCTTAAAGCGCCGATACCGGCAATTT
TTCGGGCGGCGGTATTGCCTCCCGATGATTTAATTATCGTTGATTATGCTTTTAAAGTCAATACAGATACGGAATTTATTTACCTG
TTTTTATGCCCGTCAGGGCATGGAAGGCGACCGCGCCGGACTCCACCGGACACCGGCCGCAAATCGCCGGAAACTGCGGGA
CTGACCGGAGCAACAGGCCAACCCCCCTCCCTGCTAAGCCATAACCCAGCCCGCCGCCACGCAGCTGCCGCACGTCCCCCAC
GGGGGTGCGCAGTGGGCGCCGCGCGCCTGCGCGCGGGTACGGCGGCCCGCCTGCGGGTCGCGGCGCCGTACTGCGAGTT
AGCGGCCGCCGCGCGGCCGGTTACGGGGGACACCGCACCGTCACGGCCAGCGCCCCGCTGAGCTGCACAATCCACGGATA
ACACAATAGCGCACTGGCAAAGGATGCCGACGCCTGAAGGGCGTTGGCACCCCGAAGGGGCGGGGCGGCCGCTTGCGGC
CGGGCGAGTCCGGCGCAGGGTGTGGCCTGCCAAGCGGAGCGCGGAGGCCGAAGGCCGGAGGCGTTAGCGGCCGCTGCC
CGCGTAAGCGGGGCGAGACGGGAACCGGCTCGATGCGCAGCACAGCAGAGCGGCCCCGAAGGGGTAACGCCCTGTGTGG
CATCAGGATTTAGCACAATGTCAGAACATAAACTGGAGAGATCACCGGCAAGCAGCAGCAAAGGGGCGGCACAGCCGCCCC
GATGGCTGTTACTTGTCTTTGTCGCGTAGCACTTTGATTAGGCCGGTTACGGCCGTAATCAGAGCGGCCAGCGAGGTGATGAT
TTGCGGTAGGTTTTCGAGGATGGTAGAGGTCATATAGCACCTGTAGAGAAGTTGGCGGGGTGTCGTTTCCGACGGCCGCACT
GTAACCGGGCGAATAAGGCAGGTTGTCAACAGCTTGAGCGAAGCGTCTGTTGACAACCTGCCGCGCCCGGTTTCACTGCGG
TCATAGGCGGAACGACCCACGCCAACGGAACGGCTTTATGACCGGGCAGCTGAGATACCGGCGAACCTGGCTGGCGGCTGA
CGCCAGCCGCCAAGCGCCAGCGCGGAGGGCAAAGCCCGGAGGCCAAGCGGAGCGCGGAGGCCGAAGGCCGGAGGCCG
GAGGCGTTAGCGGCCGCTGCCCGCGTAAGCGGGGCGAGACGGGAACCGGCTCGATGCGCAGCACAGCAGAGCGGCCCCG
AAGGGGTAACGCCCGGAGTCTGCCGCTGTTTATCTCTCGTTCCATCTGAAATCGGCGGTAAGGCCATTAAAAGGGTCAGTTTA
TCAGGGAGGCGTTAGCCCCCCATGTTGTTAATCATCAGGCAATATCGTCTTTGTAGCAGGCATAACCGAAGCTAAGCTCTGTTT
TCATATAGTGGCGGGCAAAGTCCCAGGCATCGTGGCCGAAGTCCTCATAATCTGCCAGGACGATTTCGCGGGCTTTGTGCCAT
TGCTGTACGGAAGAAAGCGGCAGAACAGGGCAGGGGTGCGACCAGTCAGTGACGGTGTTGCAGATCATATCTGCCAGACG
CTCCAGGGAGCCGTAAACCAGCTCTGTGCGCAGATCCGCCACCATGCGTTGTTTACGCAGTGATGCCAGATAATCAATCTCTT
TGGTTATATCAGAATTTAAGCGGGTCTGGTAATCCATGATGTACTCCTTTGCGCGCCGATACCGGCAATTTTGCGGGCGACGGT
GTTGCCTCCCGATGATTTAATTATCGGTGATTATGCCCTCAAAGTCAATATAAGTACGGAATATGCATGCATAATTTTATATCTTGC
AAAGCGTTCATAGAGTGCCTGAATCGCTTTCTGACAGCCTCAATAAAAAAAGGCGGGGATTCCCGCCTTTTTTCTTACAGCTG
CTTACGTGGCTTTTTACGCGTCATATACAACGGTATCGCGCAGTCTACCGCGTACAAAAAGCACGCCAGCGCGCCGCAACCGT
ACAGAAACGCAAGCGGCTTATTATCGAAGTAGCTGAAAACCCCTGTCGCCGCACACAGGCCCAGAACAGAGACGCAGGCGC
AGGTGATCTGCACCAGATCCCTGTATCCCGCACGAACGATAAACCAGGGCAGGGCAAGCGCAGCGGCGCTAATAATTAATGC
GAGAGGGACAAAAACGAGATAGTGATACATGTGAACTCCTTGATGGTTGCCGATACCGGCGATTGTTCGGGCGGCGGTATTG
CCACCCGATGATTTAATTAGAGGTTTTGCGCGTCCAGGAGATTGACCTGAGCCGGGGTAACGTGAAACTTTTCCCCTTTATGG
ATCACGTTATGCGGGGCGCTAATTTCATCACTGATAAAGCTAACCGGGTAACGTTTTTTACCGCAAATCCGCTCGCTAAACCAT
GCCACTTTTGCCGCTGGCCGATCCACTGGATGAACAATCACACCGGCCATGCTGCAACCCGTTGCGGGTTCGTCCAGCGTAAT

GCTTACCGGGACTGTATCCCGGTAAAAAAACTTAACCGGCGGCACACCTGCCTGCGTAGCGGCTGCGACAACTGCAAGCCCG
ATAACCGCTATCCGATTAATAAGCATTTATTCCCCTTACTCATGCTGATATCACCTTGCCAGCTGTTACCAGTTTACGAAATTCA
CTTTCATGAATTTCACGCCCATGCTCAAGCGTTGAATACACGTTGCCGATAAGCCAGTTACCAGCCGTTTTTGTCTCGGTATACC
ACCATGCTTCTGTCAGAGTAAGCAGCGGTTCGCTATCCTCGCCCTTCTCATAGATCCAGATTTTAGTGACGTCTTTACCCGTTTC
GTTGCTGCCCTGAATGGTAGGGTCAAAGGTATGTTCAATCTCTATATCGAAGTAACGCTGAAGGAAGTTAGTAAAGTGCATGA
CGACTCCTGTAAGCGCCGATACCGGCAATTTTTCGGGTGGCGGTGTCGCCTCCCGATGATTTAATTATCGTTGATTATGCTTTTA
AAGTCAATACAGGTACGGAATTTATTTACCTGTTTTTATGCCCGTCAGGGCATGGAAGGCGACCGCGCCGGACTCCACCGGAC
ACCGGCCGCAAATCGCCGGAAACTGCGGGACTGACCGGAGCAACAGGCCAACCCCCCTCCCTGCTAAGCCATAACCCAGCC
CGCCGCCACGCAGCTGCCGCACGTCCCCCACGGGGGTGCGCAGTGGGCGCCGCGCGCCTGCGCGCGGGTACGGCGGCCCG
CCTGCGGGTCGCGGCGCCGTACTGCGAGTTAGCGGCCGCCGCGCGGCCGGTTACGGGGGACACCGCACCGTCACGGCCAG
CGCCCCACTGAGCTGCACAATCCACGGATAATGCAGGAGACGAATCATGATAGGAGGCTGAAGGGGAAATGAGCGGCAGCA
GGGGAAGGGGTTGCCAAGCGGAGCGCGGAGGCCGCAGGCCGGAGGCGTCAGTGGCAGCTGCCCGCGTGAGCGGGGCGA
GACGCGTAGCGGCTCGATGCGCAGCACAGCAGAACGGCCCCGGAGGGGTGACGTCCGGGGGTTCGCTTTTAAAGATTTTC
GACCACATCAGTAAATCGTAGTGACACCATGAAGCAAAAGTATCGTGCAGACCAGAATCAACAACAGTAACAGCAGACCTTT
TTTTCGACGTAATAAAACCGGCCCCAAAGCGCCCGCAAGAAGCACACTCAACACGATTTCCGTTATACTAATCGTATTCATAAT
CTCTCACGTTTCCCTTTTTAGAACTCTGCCACACACAGATAAAACCTTATAACAAGCTACAAAACCCGTTATTCAGACGCGGTA
ATGCCTAGTTTTTTTGCCAGTATTTCAGATGACAAAGAAACCCTTATCCACTTCCCGGTTACAAGGTGAATGATTGTGGCTTCA
TGCCCTTTAACAGAGTGGGATATTAACTCACGCTGAATGATATGCGTCTCTCCATCATTTCCCGTCACGGCAATTAGGCCCTCTG
TAGAACAGGGGTTGATACTACTAATGTTGGTGTTGTTTTCGGTTTTGTTCAGCATCGCTGATCCTCAAATATCGGTTTGTGTTAC
GTCTGCCGCTTTGCGCTGGATAAGCGACTTAAAGAAATCCGACGCCTTCAGAATATCGCTATCCTGGAAGTCGGGAAATGTCG
CTTCTGTCAGCTCCTGCGCCGTACCTGCCCGTATAACGGTTTCATCGCGGATCATCGTTCGCTTAACGCCATCAGCCTGTTTTTT
GCTGAAGGAGTGCCGGAAAATAATCGCGTATTGGCCGCCGCTATCCTGATGAACAGAAGTATCCCAGCGATGATTACCCTGTT
TACGATAGTGGTGATAGATTTGCTGGCCGTAGCGCTCCTGATCGGAATTACGGTAATCGAACATTAACAGTTCCAAAAGCATTA
ACCGATCTGGCAATTGCCAGGCGGTAGGGTGTTGAAGTGTCGCTAACATAGTTTCCCCTGAGCGTGACAGTCACGATAAGGC
GGGCTTTGCCCGCCTGGTTATCAGTTAATCAATGGCACGATAAATACGATTCTGGCTTTCGTTCTCCAGCGTATTAACGTACTCC
CGCAACAGGTGATACCGGTTAGCCATCGTTTCGTTCAGTTCGGCTTTGCCTTCTTCATATGCCAGGCCGCAAAAGTAACTGTA
GGCATACAGACAAACAATAATCCCTACTTCGCGCGCGCTGCATTCACCTTCAAAATAGTTAGGTAACGAGAGCCAAAGAGGTT
GGGGCGCTTCCATAAAAAACGCGCCATTGCTGGCCTGAAGGTATTCCCAATACCCTCCCTGGTAGTCTTTAGCGTAACGATTC
AGAAAGGACTGAATGAAGTGATCTGCGCTGAAGAAAGCGCCACGAAATGCCGCAGGCATGAAGTTCATGCGGGCGTTTTCA
GAAATGTAGCGGGCGGTGATTTCGATAGTTTCCATGATACTTCCTCTTAAAGCCGATACCGGCGATGGTTAAGCGGCAGGCAC
ATCACCTGCCACTTTTTAATTATCGTACAATGGGGCGTTAAAGTCAATATAAGTACGGATTATATTTACCTAATTTTATGCCCGTC
AGAGCATGGAAGGCGACCTCGCCGGACTCCACCGGACACCGGGGGCAAATCGCCGGAAACTGCGGGACTGACCGGAGCG
ACAGGCCACCCCCCCTCCCTGCTAGCCCGCCGCCACGCGGCCGGTTACAGGGGACACTGAGAAAACAGAAAGCCAACAAAC
ACTATATATAGCGTTCGTTGGCAGCTGAAGCAGCACTACATATAGTAGAGTACCTGTAAAACTTGCCAACCTGACCATAACAGC
GATACTGTATAAGTAAACAGTGATTTGGAAGATCGCTATGAAGGTCGATATTTTTGAAAGCTCCGGCGCCAGCCGGGTACACA
GCATCCCTTTTTATCTGCAAAGAATTTCTGCGGGGTTCCCCAGCCCGGCCCAGGGCTATGAAAAGCAGGAGTTAAACCTGCAT
GAGTATTGTGTTCGTCACCCTTCAGCAACTTACTTCCTACGGGTTTCTGGCTCGTCAATGGAAGATGGCCGCATCCATGATGGT
GACGTACTGGTTGTGGATCGCTCGCTGACGGCCAGCCACGGCTCAATCGTAGTCGCCTGCATCCATAATGAATTTACCGTGAA
GCGGCTACTGCTGAGGCCCAGACCCTGCCTGATGCCGATGAACAAAGATTTTCCTGTGTACTACATTGACCCGGATAATGAGA
GCGTTGAAATCTGGGGAGTGGTTACGCATTCCCTTATCGAGCATCCGGTATGTTTGCGCTGATTGATGTCAATGGCATGTACGC
CAGCTGTGAGCAGGCATTTAGGCCAGATCTGGCAAACCGAGCAGTGGCCGTTTTATCCAACAATGACGGCAACATTGTGGCC
CGTAATTACCTGGCGAAGAAAGCGGGCCTGAAAATGGGCGATCCGTACTTCAAAGTCAGACCCATAATCGAGCGTCATAACAT
CGCTATTTTTAGCTCTAATTACACTCTCTATGCCTCCATGTCGGCCCGGTTCGCGGCCGTAGTTGAGTCCCTTGCAAGCCACGTC
GAACAGTATTCAATCGACGAGCTTTTTGTTGACTGCAAAGGGATAACGGCCGCCATGAGCCTTGACGCTTTCGGGCGCCAAC
TGCGCGAGGAAGTCAGGCGACACACAACGCTGGTATGCGGGGTCGGTATTGCCCGTACTAAGACGCTGGCGAAGCTGTGTA
ACCACGCTGCAAAAACATGGCCCGCTACTGGCGGGGTGGTTGCTCTGGACGATGGCGCCAGACTGAAGAAATTAATGAGCA

TCCTGCCGGTTGCGGAAGTCTGGGGCGTCGGCCATCGTACAGAGAAAGCACTCGCCACAATGGGGATCAAAACGGTGCTGG
ATTTAGCCAGGGCAGATACGCGCCTAATCCGTAAAACATTCGGCGTTGTGCTTGAAAGAACGGTACGGGAGTTGCGCGGCGA
GGCTTGCTTCAGCCTGGAAGAAAACCCTCCTGCGAAGCAGCAGATTGTTGTGTCGCGCTCATTCGGCCAACGCGTAGAAACC
CTGACGGACATGCAGCAGGCTGTCACCGGATTTGCAGCGCGCGCAGCTGAAAAACTGCGTAATGAGAGGCAATACTGCCGC
GTCATAAGCGTCTTTATCCGTACCAGTCCTTATTCAGTGCGTGATACACAGTATGCCAATCAGGCAACCGAAAAACTGACGGTG
GCAACCCAGGACAGCCGCACGATAATTCAGGCAGCACAAGCCGCGCTGGCGCGGATCTGGCGGGAAGATATTGCGTATGCA
AAAGCAGGGGTCATGCTGGCAGATTTTAGCGGGAAGGAGGCCCAGCTTGATTTATTCGACTCTGCTACGCCTTCAGCTGGCA
GCGAGGCTTTAATGGCTGTTCTTGATGGTATAAACCGGCGTGGAAAGAGCCAGCTTTTTTTTGCAGGCCAGGGCATCGATAA
CTCCTTTGCCATGCGTCGTCAGATGTTGTCACCTGATTACACGACAGACTGGCGCTCAATACCAATAGCCACCATCAAATAATTA
CCGGCGCCGTACACGGGCCGGTTAACCCCTCAACCGGCCGAAACAAGTTTCGGCACGGTTTCGCGGTTTTCGGTAAAAGCC
GTTTCCTCTGTATAAAAGATCAGCTAAATTATGTGTATTGCACAATACATATATGTGAGGTTAGCAGTGAATTTGCCTACGCCCG
AAACCTACGATGAACTTCAGAGAGCCTACGATTTTTTCAATGATAAGCTATTCAGCAACGAGCTGCCGCCATGCCTGATAACGT
TGCAGCGTGAGAAGCGAACGTATGGCTATTGTTCCTTTAAGCGTTTCGTCGGCCGTGAGAGTGGGTACACGGTAGACGAGAT
CGCTATGAATCCGGTGTATTTCTCGATCAGAACCATAAAGGCCACGCTTTCAACACTGGTGCATGAGATGGTTCATCAGTGGC
AATTCCATTTTGGCGAGCCTGGCCGCCGTGGCTATCACAACAAACAGTGGGCGGCCCGGATGGAACGGGTAGGACTAATGC
CTTCTGATACCGGCGAACCGGGAGGCAGGAAAGTGGGCCAGAGCATGACCCATTATATTATTGCCGGTGGCCCTTTCGATATG
GCCTGTGATGAACTGCTGACAGGCCATTTCCGGCTTTCCTGGATGGACAGGTTTCCGCCTTACCAGCCTAAGCCTGGCGCTGT
GCTAAGCCCTACAGGAAAAGGCTATATTGACGACGAGGAAGATGATAGCGAACACGAACAGGAGGTGGAGGAAGGGCGCG
ACCCGGTTGAACTCGACGACGAGATCATAGAGGCCATGCGATTTGTAACCCCACCGCCTGAAGCACCGGTGAACAAAACAAA
CCGGGAAAAGTACAGCTGCCCGGTGTGTCATATCAATCTCTGGGGTAAACCGGGGATAGTGGTTTACTGTGGTGGCGAGCAC
TGTAATAAAGCCGCGTTAGTAGTCTTAAAATAAAGTCCTTTCGGACTTTATTTTTTTTCCATTTCCGAGGTCGTGATGTTATTAAT
GCTGTACTTCGCGGCTTCTTTTAAAACAGTTTCAGCAAGGCTTGCTGGTATCCAGACCTGAACTAATTTTAATGGTTCGCCGTT
CTCGGCTTTAAGAGTGGTGTTCTGGTACAAATCCCAGATTCGCTTAACGGTGCTGGAAATGTTTTGCTTGGAACGGCCTACTC
GCATGGCTACGTCTGATGATTTCTCACCTTTGACAAGCACGGAATAGCCAATATCTGTTGTGATGTGTGCAAAGGAAGCCATTT
GCGGCAGCAGCTGTTTCCATTCTGTTTCTGAAATTCTGTTTTTCTGAGCCATCTGTGGCGCCTCCGTAGTTTTGGTTACAGAAA
GGATATACTCAGAATAAACAGGGGTCAATACAAGTACGATTTTTATAAACTTTATTTTATTTGAGGGTGAGGCCCGGTGCGGCA
GCAGCGCGGGCCTCGATGGTGCCGCGAAGGTGCTGGCGCCATGCTCGGATTAAAACATGAACCGTGAAGAACTGCGAAACT
TGTTTTCGCGGTTCTGAGGGGTTGACCGAGCCGCGAAGCGGCGCTGGTAAGCGATGATATGCACATATCCACAGGCATATTTT
TAAAAGGTATTTTATAGATTTTTTATCTTTTTAAAGTCTTTTAGAGCTATATAACTCATTGATTTAAAATCATAAATAAGTGTTATCT
CTGGGAATCCGCCCACCTTGTTATGGGAATTGGCCCACCTTACTATGGGAAACAGCCCACCTTACTATGGGAATTAGCCCACCT
TGTTATG

>pQEB1_ISKpn26

GGAATTGGCCCACCTTAGACGAAACTGTAAAAAATGTATTTACTTGTTTGAACTTTGTGGTAGTGTGGAGAGTAATTTTTAACC
CACAAAGGCAAGGCGCATGGATAAGTTGCTGAACAAAAAGATAAAAGTTAAGCAGTCTAACGAGCTTACCGAAGCTGCTTAC
TACCTCTCGCTAAAAGCAAAGCGCGTTCTCTGGTTATGTCTTATGCAGACGTATTTCACAGCTTCAGTAAGCGAAGATGATGAT
GAGATGGCTGTACTCGGTGACTCTACTTTCAAAGTAAAGGTGGCTGACTATCAGCAAATTTTTCAGGTAAGCCGTAACCAGGC
TATCAAGGATGTTAAAGAAGGCGTGTTTGAGTTAAGCCGTTCTGCGGTAATCTTTTACCCGAAAGAGGGGCGTTTTGACTGC
GTCGCGCGCCCCTGGCTAACAGAGGCTGGCAGCCGATCAGCTCGTGGTATCTGGGAAATCGAATTTAACCATAAACTCCTGC
GGTACATTTACGGCCTGACGAACCAGTTCACCACCTACTCGCTCCGCGATTGTGGCAGTCTTCGAAATCCCCGGACGATCCGC
CTTTATGAAAGTCTTGCTCAATTCAAATCTTCAGGCTTATGGGTTACTACTCATGCTTGGTTAAATGACCGTTTCCTTTTGCCGG
AATCCCAACAGAAGAACTTGGCAGAGTTGAAACGATCTTTCCTTGATCCTGCACTCAAGCAGATAAATGAGAAAACACCTTTA

CTTGCTAAGTATAGTATTGATGATTCAGGAAAATTTCTGTTCTCAATAATTGATAAGCAAAATCCCGTCTGACATAAATCAGCAC
ACATGAGCCTGTCATTTGACAAATTTTTGTCATGAAGATGGGCGAATTTCCACACAGCACCGGCGCCCGGCAAGATGGGCGG
ATTCCCACACGACAGCGGCGCCCGGCAAGATGGGCGGATTTCCACACTACAGCGGCGCCCGGCAAGATGGGCGGATTTCCA
CACGGCAGCGGCGCCCGGCAAGGTGGGCGGATTTCCACACGGCAGCGGCGCCCGGCAAGGTGGGCGGATTCTCACGCGG
CAGCGGCGCCCGGCAAGATGGGCGGATTTCCACACGGCAGCGGCGCCCGGCAAGGTGGGCGGATTCTCACGCGGCAGCG
GCGCCCGGCAAGGTGGGCCGATTCCCACGCGGCAGCGGCGCCCGGCAAGGTGGGCCGATTCCCACGCGGCAGCGGCGCC
CGGCAAGGTGGGCCGATTCCCACGCGGCAGCGGCGCCCGGTAAGGTGGGCGGATTTCCACACGGCTGCCGCGCCCGGCAA
GGTGGGCGGATTTCCACACGGCAGCGGCGCCCGGCAAGGTGGGCGGATTCTCACGCGGCAGCGGCGCCCGGCAAGATGG
GCGGATTTCCACACGGCAGCGGCGCCCGGCAAGGTGGGCGGATTCTCACGCGGCAGCGGCGCCCGGCAAGATGGGCGGA
TTTCCACACGGCAGCGGCGCCCGGCAAGGTGGGCGGATTCCCACACGGCAGCCTCGCCCGGCAAGGTGGGCGGATTCCCA
CACGGCAGCCTCGCCCGGCAAGGTGGGCCGATTCCCACGCGGCAGCCTCGCCCGGCAAGGTGGGCGGATTCCCACACGGC
ACCGGCGTGCGGCAAGGTGGGCGGATTCCCACACGGCACCGGCGCGCGGCAAGGTGGGCCGATTCCCACACGGCACCGG
CGCCCGGCAAGGTGGGCCGATTCCCACACGGCAGCCGCGCCCGGCAAGGTGGGCCGATTCCCACGCGGCAGCCTCGCCCG
GCAAGGTGGGCCGATTCCCACGCGGCAGCCTCGCCCGGCAAGGTGGGCCGATTCCCACACGGCAGCGGCGCCCGGCAAGG
TGGGCGGATTTCCACACGGCAGCGGCGCGGGGCCAGTGGGATTCAGGAGAATAGGTGTTTTACCGAATGCCCTGACGAGG
CGTAAAAAAACCGCTTGGCGGCGGCCTCATAAAGCAGAAAACCCGCTCAAGGCGGGTTATCTGCTCTGTAGCCTGTGATGCT
TCGCGGGCATCCGGCATACAGCGAGGTGAAATTCTTCTTTTGGCATGTTAATTATACGTCTAACGCGGCATATGATCAAACCTG
TATTAAATAAGCCACTGTACCGTTTATAATGCTCTCAGATCAAAGAGGTAAAGCCCGTTTAGCCGCCTGTGTGATGAGCCAGTT
CAGACTCTTCAAAATCGAATTTGGTACTAAACAGGACCCGAACCGTGGGCAAGCACACGGCAACGGTATAGCCCTCTTCCGG
TTTCGCACCCGGAAGCCTGGGCGGCAGCGTGGTGAAATTCTTCTTTTGGTTAAGTGAATGGCATAACCGGATGGGCGGATTA
GAGGAAAGGGGATTGCCTAGTAACCTACGCGCCACAGAGATGGAGGTGCGGGGAATGATTGAGCTGATTATCGCTATTCTGA
CCTTAATTGCGGCTGTATTGCAGTTGATCAACTGGTTCCTTTAATGGTGCCGGAGTCTGTGAAGGTGAAAGCCTGAACGGGC
AAAACTGAAAGGTTTATAGCCGTCCTTCGGGGCGGCTTTTTTTCGGCAAAATTAGGGTTTTACCGAATAATGCAGAGTTTTAA
GGTGAGAATTTGCAGACTTGGCGTTTTACCGAACATAGATACTCCCTAGGCTGATAGGTGCATTAGTTATCACCCTACCTGAAC
ATATTGTAAAAGATGTCAGTCTCCAGTGACTTGTGTACTATCAACTGACAAGACTCTTACACGCAACGCAGGGGGATGGAGTT
TTATGCTTAGAAAAATAATCAGGGGTAGCGGATTCACTCAGTCAGAAGAAAAACTGATAGAGTTCGCTGATGATGCTTTTTTT
GGTCTTTGGTCTTATCCTAATGTTTATAGCGATGAGGGTTACTCTAAAAATAAAATTGGGAAAGAAGTTAGTGACTTATTAGTTA
TTTTTGATAAAGATATAATAATTTTTTCCGATAAAGCTATTACATACAATAAAAACAAAGATCCTAAGGTTGCATGGCAGAGATG
GTTTAAAAAATCAGTCATACAGTCTTGCACACAGTTATTTGGCGCAGAGAAGTTTATAAAAGATCATCCCGAAAGACTTTTTGT
TGACAAAGAATGCTCAGTTAACCTCCCCATTAAAATAGATAATTCTTTTAATTTTCATTTGGTGGCCGTCACTAATAATATTTCAG
ATCCGGCGATCTCGTACTTTGACAAAATAGAAAAAGGCAGCTCTGCTACTTTAGTTAACATATTTCCTTTAAACGCCCATCAATG
TCTAGAAAATCCATTTTGTGTCGGAGACGTTTATCCTGATAAGACTTTTGTCCATATACTTGATGAGACTGCCCTAAAACTACTG
TTAACCGAGTTAAACACAGCAACTGATTTCATTGGCTACCTTAACGAAAAAGAGAGGGTTGTAAGAGAAAGAACATTATTGG
TCAGCGCTGGGGAAGAAGAGACTCTTGCTGCTTACATTATGGGTGATAAAACCATAATATCAAAAGAAATTATTGGAAACGAT
CAAGGGATGACCATACCGGAAGGTGAATGGAAAAACTATAAAACCACTTTCAATTATCAATATCAGCTCTCAATGAAAAAGGG
TAGCGTTTTCTGGGATAACCTAATCCACAACTTCTCGACAAGTATATTGTCAGCTAACGTTGGTTTTTTTAGTGAAATTGAATTT
TCTACACATGAATTAGGTGTTAGAGAATTAGCCAAAGAAAGTAGGCAATCTAGATATTACCTTTCAAAGAACTTTAAAGAGAA
ATTAAAAACAACTCAGCCTCATCTAAGAACGTCAAGAATGGTCGAATCAATCGATGAGCCTGGAAAGTTTTACTTATTCCTTTT
TTTTCCTAACGATAGCAAGTTGAGTTACTCTGATTACAGAATTCAACGTATATCTTATATAAATGCTTATGCTGAGGTTGCCTTTA
ATAAATACAGACATATTAAAAAATTAATTACTATTGCAACAGAGCCGCAAAATACAGAAGGAAGATCTGAAGACCTAATATATA
GCATATCCCCAGAGAAATTTACCAAAGAGCAAAATGAAAAAGCCAAAAGATTATCAAGAGAATACAAAATACTAAGTGATTTT
TTACCTACTAAAACGACAAAGAGCGATAACTTTAAATCAGTTATATCAAAAGGTGAAAAAATAGGGCGGAATACACCTTGTCC
ATGTGGCTCCGGTGTTAAATTTAAAAAGTGCCATGGTGCGAATAATTAGCATTATTGTATGTATAACGGTAATGGCGCGGCAGA
GAAACCGGCGCGTTCTGCCCTAGTGTTGGCCTGCGGGTTCCCCCGCACCCGCTGTATGTAGTATCGGCAGCATCTGAGAAAA
CCACTACATGTAGTTATCAGCGCCACAACGGCGCGGGGACGAGTGCGGTTCGGAAAAATTGGGGTTTTACCGAATCCGGCA
AAAGATTGCTTCCTATAACGTCCGCTTCTGGCACACAGCAGCCGTTAAGATGTAAGGCCTTACGCCAACTAAATCTAATGGGA

CAGATTTAGTTGGTGATGGTCAAGTAATCTGCAAACGGTCACCAAGTAAAATGCAAATGGGTAGTCAAGTCCGATGCAATTAC
GCACCCGGCAAGGTGGGCCGATTCCCACACGACAGCAGCGCCCGGCAAGGTGGGCGGATTTCCACACGGCAGCGGCGCCC
GGCAAGGTGGGCCTATTCCCACACGGCAGCGGCGCCCGGCAAGGAGGGCCGATTCCCACACAGCACCGGCGCGCGGCAA
GGTGGGCCGATTCCCACACGACAGCAGCGCCCGGCAAGGTGGGCGGATTTCCACACAGCACCGGCGCCCGGCAAGGTGG
GCCGATTCCCACACGGCAGCGGCGCCCGGCAAGGTGGGCCGATTCCCACACGGCAGCGGCGCCCGGCAAGATGGGCGGAT
TTCCACACAGCACCGGCGCCCGGCAAGGTGGGCCGATTCCCACACGGCAGCGGCGCCCGGCAAGGTGGGCGGATTCCCAC
ACGGCAGCGGCGCCCGGCAAGATGGGCGGATTCCCACACGGCAGCGGCGCCCGGCAAGATGGGCGGATTTCCACACGGCA
GCGGCGCCCGGCAAGATGGGCGGATTCCCACACGGCAGCGGCGCCCGGCAAGATGGGCGGATTTCCACACGGCAGCGGC
GCCCGGCAAGGTGGGCCGATTCCCACACGACAGCGGCGCCCGGCAAGGTGGGCCGATTCCCACACGACAGCGGCGCCCGG
CAAGATGGGCGGATTCCCATATCGACATGTATGTAGCTTGTGTTATCCGTGGATTGTGCAGCTCAGCGGGTCGCTTGTCGTATG
GCGTAGTGTCCCCCGTAACCGGCCGCGTGCGGCCGCTAACGCGCAGTACGGCGCCGCGACCCGAAGGCGGGCCGCCGTTCC
CGCGCGCAGGCGCGCGGCGCCCACTGCGCACCCCCGTGGGGGACGTGCGGCAGCTGTGTGGCGGTGAGCGGGATTAGGG
CTTTGCAGGGAGGGGGCTGGGTCGGGCGATACGTTCAGCATTGCGGTTTCCGGCGATTTGCGGCCGGTGCCCGTTTAACTC
CGGCGTGGTCGCCTTCCATGCCCTGACGGCATAAGAAAATAAAACCGCCATGCTGCGGTCATTCATGATTTTGTGGTGTAGCG
ATAAATAGTCATGCGAGAAACGTTGAAGCGCTTAGCAACTGCACCAACTGTCATTTCAGGATCAGCAAGTAAGATTCTAATTT
GTTTAACATCTTCTTCAGAAAGTGACGGTTTTCTCCCTCCCACACGGCCCCTTGCGCGTGCAGCTGCAAGGCCTGAGCGCGTT
CTTTCAATATTGCGGTTGCGTTCAAAGCTAGAGAATATCGCCATCAGATGAGTATAGATTTCCCCTATAACTGGCGCATTTGTGT
CTATTCTGTCCTTGATGGCTATGAAAGTTATTCCGCGTTTCTTCAGGTCGTCGAGTAAAGTAATGACTTGACCCAATGAACCAC
CGAGCCGATCTAGTGCCCAAACTACTAGGGTATCTCCCTCGCGCAATGCTTTCAGGCAGTTCTCCAGTTCCAGCGCACCTTTTT
TGTCGCGCTTTGGGCCGCTACGTGAGGTCTGATCCTGATAGATTTGCTCACATCCAGCTTTTGTTAGTTCGTCAACCTGGTGCG
CCACATCCTGAAGATGCGTAGATTTACGTGCATAGCCGATTTTCATTCTTTTCTCGCTAATTAGTTATGGGGTTATTGTTATGTTG
ATACAGTAACGAGTTTTGTTACATGAGGGGAGTCATTTTTCGGGAGAAGTCAGGACTTTTCAAGACTGTCACAAAAACCATC
GTTTTTGATACATTAATTTAACCAATAGGTTGCAGATCAAATCGTCTGTAACAGCCTTTCTGGCTGTTTGTATATAATCATGAAAA
AATGGTGAGTAGAGTTTCAGGGTAACAGGGGATGCTTATGTCGGTTTTCCACAACTGGCTACTTGAGATCGCATGTGAGAATT
ACTTCGTCTACATCAAACGCCTTTCCGCCAACGATACCGGCGCAACAGGTGGTCACCAGGTAGGGCTTTATATCCCTTCAGGT
ATCGTTGAAAAACTCTTTCCGTCTATCAACCATACCCGTGAACTGAACCCTTCGGTTTTTCTCACCGCACATGTGTCATCGCATG
ATTGCCCTGACAGCGAAGCCCGGGCAATTTATTATAACAGCCGTCATTTTGGTAAAACCCGGAATGAAAAAAGGATTACCCGC
TGGGGTAGAGGCAGCCCACTTCAGAATCCTGAAAATACAGGGGCTCTGACGCTCCTGGCTTTCAAGCTTGATGAGCAAGGG
GGGGACTGTAAGGAAGTAAATATTTGGGTATGCGCCAGCACTGATGAAGAGGACGTCATTGAGACCGCTATTGGTGAAGTTA
TACCCGGAGCGCTTATATCCGGCCCCGCAGGACAGATTCTAGGCGGACTATCTCTACAGCAAGCGCCAGTAAATCATAAATATA
TTCTACCTGAAGACTGGCACCTGCGCTTTCCGTCGGGAAGTGAAATTATTCAGTATGCAGCCAGCCATTATGTGAAAAATTCCC
TTGATCCGGATGAGCAACTTCTTGACCGCCGGCGCGTGGAGTACGACATATTTCTATTGGTTGAGGAACTGCATGTTCTGGAT
ATCATCCGGAAAGGATTTGGCTCTGTGGATGAATTTATTGCGCTGGCCAATTCTGTCAGCAATCGCCGTAAATCCAGAGCCGG
GAAGTCTCTGGAACTGCACCTGGAGCATCTATTCATTGAGCACGGCCTGCGACACTTTGCGACGCAGGCCATCACAGAAGGT
AATAAAAAACCCGATTTCCTTTTCCCTTCCGCAGGGGCTTACCACGATACTGAGTTTCCCGTAGAAAATCTGCGCATGCTGGCA
GTCAAGACTACCTGTAAGGATCGCTGGCGTCAGATACTGAATGAGGCCGATAAAATTCATCAGGTGCATCTGTTTACACTCCA
AGAGGGAGTTTCTCTGGCTCAATATCGGGAGATGCGGGAGTCGGGTGTCAGATTGGTCGTGCCATCATCGCTGCACAAAAAA
TACCCGGAGGCGGTGAGAGCTGAGCTAATGACGCTAGGTGCGTTTATTGCTGAGCTGACAGGGCTTTACGCAGATATTCCAT
AGATTATCTCCCGGCATAAATACCGGGAGGAGCGATCAGATTCGTTCAACCTTGCACGAATCGGCATTAACCGCTTTCAGGAT
ATAAGGTTCAAGCAGTTTGGCTACGGCTTCAAACACGGGCACCACTACGGAGTTACCGAACTGCCTGTACGACTGAGTGTCT
GACACAGGAATGCGAAAAGGCCTGCCATCTACTTTTTCAAAACCCATAAGGCGCGCGCACTCTCGTGGAGTCAGCCTGCGGG
GCCGATGCGCCTGATTTTCTTCGTTCGCGAAGTCTGTTTCACCTGTGGCCATATCCCAGCCACGGTCTATCAGAATTTCAGACC
CGTCTTTGTGATAGCGAGCAGAAAGCGTACGGGCAATGCTTTCTTTATTTTCAGGATTAACGAGGCCAAAACCGAATCCGTTA
CCCTTAGCTGCGTGCTTTTTGGCGTAGTTATAGAGATACTCCCAGAGTTTCGGCGTCAGTATATATTTGCTGTCAACCACGGGT
TCCAGCAGTTCGCCAAATGACGGACGCTGTTCCGGATAAAAACGACTAATATCGCGCAGGGTAAAGCCCTGGTGAATGTTCA
GATCACGACGGAAACCGACCAAAACGATACGTTCTCGGTGCTGAGGTAAAAAGTGCTTTCCGTCGATAACTTTAGGATCGTTT

TTGCCCATCTCAGCTGCATCCGCAACTTCATAGCCCAGTTCGTCGAGGGTATCCATGATGACTTTAAAGGTTTTACCCTTGTCAT
GGCTCTTCAGGTTTTTAACGTTTTCAAGAACAAAGATGGCAGGTTTTTTTGCGCGTATAATACGCGCCACATCGAAGAAAAGC
GTTCCCTGAGCCTCACATTCGAAACCATGCGCGCGCCCGAGCGAGTTTTTCTTGCTTACGCCCGCAAGGCTGAACGGTTGAC
AGGGGAAACCTGCTAGAAGTACATCATGATCCGGCACATGCTCATTAATGTAAGCATAGGCATCGTTTTCAGGTACTTCAGGTT
TATCACTGAGCGTGACTTCCCGAATATCGAGATTGAAAGTGTGTTCCTGAGCATCGTTAAACCAGTTAGCTTTATATGTGCGCA
CAGCCTCTTTATTCCATTCACTGGTAAAAACGCACTGGCCACCGATGGTTTCGAAGCCCTTCCGTATACCTCCAATCCCAGCAA
ACAGGTCAATAAACCGGAAGGCATAGTCAGGGTGATGTGCAGGCGCTTCCGGAAGCATTTTTCGTAGAAGTTCCTCTTCGGC
TAACGTCAGCGTCTTAGGTGAGCACTTACCATTAATCCAGCGATTAAGAGTCTCGCGACTCCACTCATTTTTACCAACTTTTCTA
AGCAGTTCAGCCACGTACTTCTGGTCATAGATTTCCAGCACCTGCCCGAGCAGCTTTTTATAATTTTCCTGTCGCAGTTGTTCTT
CCGCTTCTGCTTTCTCAAGCAGATCCTGCGCCAGTAATTCAAATTCAGACATATTGCCTCCATTGGGTCTTATGGGTGAAACTG
TATCACTCATTTGACCCAGATTGAATGTTTTTATCTGGATATTTAAACAGGTTTATTGTTAGGTAACGCACGTTGGCCACGCTGG
AGCGTCTTCTGGGCCTGCTGTCGGCCTTTGAGGTCGTGGTATGGATGACGGATGGCTGGCCGCTGTATGAATCCCGCCTGAA
GGGAAAGCTGCACGTTATCAGCAAGCGTTACACTCAGCGCATTGAGCGACATAACCTGAATCTGAGACAACATCTGGCAAGG
CTGGGACGGAAGTCACTGTCGTTCTCAAAATCGGTGGAGCTGCATGACAAGGTCATCGGGCATTATCTGAACATAAAACACTA
TCAGTAAGTTGGAGTCATTACCGGTTCTCTTTGTCTTTTAGTGATTCTATAAACCTCATTACGTCTGAATATAAAAATCTATTATTT
GATTTATGTGGCTCATGAGGTTGTGGGATGGTCTTGTTTTTGAATGTGCCAGTTTTCTTAATGGCAAAGATTAATTCACCTTCT
GTTATTCCTAACATTTCAGCAAATGTTTTTGCTTCTATAGTTACTGACTTCATTTAATTAACTCTCATGGTATCGATTTTCTTTACC
GGCATCTTTAACAATGGTGCTCGTTTCTAGTGTTGCTGCGGTACGCTTCATCATCGTCTGCGGGGCGGTTGCGATAGTGAAGG
AGCTGCCGGGCGTGAGCAAATCTATCAGGCGCTGGCCGCTGATAATCTCCATCCGTTCACTGGCAATACTGACAGATTTTGAA
CCTGCGCCGGTTTTCCCGGTATGGCAAAACAGACCGCGACAGTTATGACGTTTAAGCAACTTCTCGAACTCCTGTACGTGCTG
TAAAGCAATATGGCCGCGATAGCGTTTAGCCTGAATAAGATAGCGATATTTTCCTATTATTACCTGGCCGTCAATGCCTCCATCG
CCGGTATAGCGTTTGTTTCTGATGGTTCTGAAGCCATGCGCGGCTTTGTTGAATAAATCGAACTTTTGCTGAGTTGAAGGATC
AGATCACGCATCTTCCCGACAACGCAGACCGTTCCGTGGCAAAGCAAAAGTTCAAAATCACCAACTGGCCCACCTACAATAA
AGCCCTCATCAACCGTGGCTCCATAACTTTCTGGCTGGATGATGAAGCTATTCAGGCCTGGTATGAGTCGGCAACGCCTTCATC
ACGGGGAAGACCTCAGCGCTATTCTGATCTCGCCATCACCACCGTTCTGGTCATTAAACGCGTGTTCAGGCTGACCCTGCGGG
CTGCACAGGGTTTTATTGATTCCATTTTTACACTGATGAATGTTCCGTTGCGCTGCCCGGATTACACCAGTGTCAGCAAGCGCG
CAAAGTCGGTTAATGTCAGTTTCAAAACGTTCACCCGGGGTGAAATCGCGCATCTGGTGATTGATTCCACCGGGCTGAAGGT
CTTTGGTGAAGGCGAATGGAAAGTCAAAAAACACGGCAAAGAACGCCGTCGTATATGGCGAAAGTTGCATCTGGCCGTTGA
CAGCAACACACATGAAATCATCTGTGCAGACCTGTCGCTGAACAATGTGACGGACTCAGAAGCCTTCCCGGGTCTTATCCGGC
AGACTCACAGAAAAATCAGGGCAGCATCGGCAGACGGCGCTTACGACACCCGGCTCTGTCACGATGAACTGCGGCGTAAGA
AAATCAGCGCGCTTATCCCGCCCCGAAAAGGCGCGGGTTACTGGCCCGGTGAATATGCAGACCGTAACCGTGCTGTTGCGAA
TCAGCGGCTGACCGGGAGTAATGCGCGGTGGAAATGGACAACAGATTATAACCGTCGCTCGATAGCGGAAACGGCGATGTA
CCGGGTAAAACAGCTGTTCGGAGGTTCACTGACACTGCGTGACTACGATGGTCAGGTTGCAGAGGCTATGGCCCTGGTACG
AGCGCTGAACAAAATGACGAAAGCAGGTATGCCTGAAAGCGTGCGTATTGCCTGAAAACACAACCCGCTACGGGGGAGACT
TACCCGAAATCTGATTTATTCAACAAAGCCGCCATGCGCTTCAAATCCTTCCAGCAACAGTTCTTCAAACACAAAAGGATCAAT
TTTCCTCAGGTAGTTAATTTTTTGTGGGAAGCCCGGCAACGTCTTTATGCGCTCCAGCACCCGCCGCGCACTTTGCTGCTTCCT
TTTGTGTCGTCGGTTGCGTACTGAACGCCGGAAGAATACAACGGCAAACAGTGCGATGGCGCTGCAAGCCCATAGAATAAGG
TTTTCTGTAGTGGGGAAGGGGAACATGGTGATAGTGTGCTTTCTGTGGGTAAAGAAAAGGGCGGTTAAACCGCCCTGGTGT
TTAGCGACGGCTGTAAACCTGCCACGAAGCGCTGCCTGACTGATTTTGGCAAATCCGCCCGTAAAGTACGGTGCCGGTCGAG
TAGCGGGCGCCATTCAGATAGCAATAGCCGCTGGATTGCAGCAGTTCGTTGCGCAGCTTTTCTTCCTGCTTTGATAGCCGGGA
CTCCGCAGACTGCAAACGAACGGATAATTCGTTAATCTGGCGTTGCTGGTTATTCATCTGGCTCTGCATCGCATTACCTTTATCT
TGGCTGACGCAACCAGTTAAGAGAGCTGTACAGGCTAATGCACTTAATATAATTTTTTTCACGTTGGCTCCTTAAATTGAGATT
ATTCCTAGCCCGCCTATAAGCGAACTTTCCCGTATTTACTTATGATCTGGCTTATCATCGACTGGTTACTTCCACCTTCGCCATTAT
CCGGGCATTCATTAAGAAAAGCCTTCCTGGCATCCCTCGTGTGGTTGGGTAAAAAGCCGTGCTTGTTCTTTTTAACGATATTGA
AGAAAGCAGCTTCAGCACTGTTACACTCGCTTCCGCCGCTATCGCCGGTGAGCTTGCCCGCCATGCACATAATAACTTTGCAG
GGATCTTCAGCATGGCTGGCAGGAAGATAAAGCAGACTACCAGCTGCTATCAGAGGGATTAAGAGTTTCTTCATTGTTTGTC

CTTAACAGTTTGTTCAGATATACACCCGCCAGAATGTTGATAACGGTAAGTAATATTAATAATAACGCAGAGTTATAAATAGATTT
GTAACCTATATCGCCTGCGATATATTCGACAATAAAAACGAAAATCGTTAACATTGCGAACAACTTAATTAACTTTCCTGTAATTT
TCTTAACGATATAAGCAACTGAATAAGAGCCGGATTTGATGATGCTGGCGAGTAACCTGATAGCATGGACTCGGAATTTTCTTA
AAATGGGCGGAATGGAAAATGCCTTTTTCTTCCTTCTGCCTTTTTGCTTTTCCGCAAAATCAATAACATCGCCCATTTTTATACT
CTCCGGTTATATCTTTAGATCATCAAGTGATAAACCACTATCAAGCAGCTCTTTGAGCCATGTTGGTCGGCGGCCAGTCCCGGA
CCAGGTATTTTCTGCGTTCCTGGGGTCGCGGTATTTTACTTGTCTATGTTCTCTGGGGTTGGCAACGCCCTGCATTTTCATGGC
TTCCAGGCTAATGCCAGCGTCCAATTTTGCAGTTAGCCAGGCCGGGCGCTTCCCTATACCAGTCCACGTATTAAAAGGGTTATC
CGGGTCACGATACATGGGTTCACCTTTGGGGCGTTTTTCTGATTTCAGGGGAACCAGCATTACCTTCCTTCCTTCAGAGGTTT
TCTTTTCGTTTCTTCTTGACACGATCTCATTAGCCCTTATCATAAACATAAGTCGTCGAAATTACAATTACGCGGTGAAACGTAA
ATGAGTAAACATCCAAAACTTCTGGTTCTCGCTCTGGCCTGCCTTGCTTGTGCTGGCCGTGCCAGTGCTGCGCCTGCCTCAGA
TGAAGTTGCCAGGCTTGCGCAGAGATGTGCGCCTGATGTTTCACCCTTAACAATGGCGTACATCGTCGGCCATGAGTCCTCAA
ATGGGCCGTACAGGATCAATATTAACGGTGGTAGTACCCAGTTAAAACAGCAACCACGTACTGAAGCTGAGGCCGTCAGCGT
TTCGAAAGTTCTGCTGAAGGATAATAAAAGTTTTGATATGGGCCTTGCACAAATTAACTCAAATAATTTAGTGGGCCTGGGTCT
TTCGGTTGACGATATTTTCAAGCCCTGCATCAACCTGCGGGCGAGCCAGACCATCCTTAAAGCCTGTTATGATAGCGCCCTGA
AATCCTATCCAGCCGGGCAGGTTGCGCTGAGACACGCGCTTTCCTGCTACAACACCGGCTCACTCATAAACGGGATTTCTAAC
GGGTATGTCACGAAAGTTATCAACGTGGCGCGTCAATCAACTGATTTGAAAATCCCTACGCTGCTACCTGATGGCCAGACCAG
TGAGGACAGCACCGCGACTGAGCCTCAGCAGGCAAAAAGTACGGCCACGCAGTATGACGGTGAACAAGATGTTTTTGGTTC
GGGTGATGGCGATGCCTTCAGCCGAAATAATACGGATGCCTTTTAACCAAACAGGAAACAGCGAAGGGGGAGTGAGGTTA
TGGATGGAACGTTTGTACCTTGTATTGCAATCACAGATCCTGGAGCTATTTGAAACAGGAAAAGTGAAGGAGGTAACGATAA
AACGGGTTTCATTAAAGACGTGGTATCCCGTTTTTCAGATAGACGATGAACAGTTGGGCCAGATCGCATGTTCCATTCGGGTT
AACAAAGAGCATGAACTACGAACCTGGGCTGATTTAAGGCTACTGGCAGAGTTTTTGAAAGATAAGTGTGGCGTTGAAGAAT
GCCGGTTAAATCTGCAATCAACAGAAGATAGTGAGTAAGGAGAAAGTATGACCACGTTGTTTAAGAAGTATGGCCCTGCGGT
AGTTATGGGCGTTTTGTCCATTGCCCTGCCGCAAATTGCGCTGGCCGCTGGCACCGATACTGGTGAATCAACCGCTACATCAA
TCCAGACGTGGTTGAGCACATGGATTCCAATTGGTTGTGCTATTGCGATCATGGTTAGTTGCTTTATGTGGATGCTTCACGTAA
TCCCAGCCAGCTTTATTCCTCGTATCGTAATCTCGCTGATTGGTATTGGTTCTGCATCATTTCTGGTTTCCCTGACGGGCGTAGG
AAGCTGAACAACGCGAAAAGGGGGGGACTTTTGTCCCCCAAAGTGAGGACTACAAAGATGTTCGTTGACGGGAAAAGACC
GCTTTTCAAAGGTGCGACTCGCTTACCTCGCGCGCTGGGTGTACCACGTAATGTAGCTATGATGATATTCATGATTTCTGCCTC
GCTTTTTATGATTATTCATATGTGGGCGATCCTGGTGTTCGTCTTTTTGTGGATTCCTTCAGCTGCATTAACAAAATATGACGACC
GCATGTTTCGAATTATGGGCCTGTGGTTGAAAACCAAATTCAGTAATTGGTTTGATTCTCCGTTTAAGCAGTGGGGAGGATCG
TCTTATTCCTCTGTTGACTACAAACGTAAGGGTTTAAAATAATGAGAGCTGCCACCGCTACGAAGCCAAAAAAAATTGATGCCT
ACCGTAAGGAGCCATCAGTAAATAAAAAGTATTTGCCCTATTCTTATCACCTCAATGATTACGTGATTTCGATGGAAAACGGCG
ATCTGATGGCTTTTTTCAAGCTGGATGGCCGCACACATGACTGCGCATCAGATCGGGAACTGGTCACCTGGCATAAAGACCTT
AATACGCTGGTCAAGAGCTTCGGAACAGACCATGTAGAGCTGTGGACGCATGAATATCACCATGAGGCTAAAGAGTACCCGG
ATGGTGAGTATGACCATTTTTTCCCTGCTTATGTTGATCAATATAACCGTAAGCTGCACGGTGATTCCAAGCAGCTGATTAATGA
CCTTTATCTGACCGTTATTTACAAACAGGTAGGGGATAAAACACAGAAGTTTCTGGCGAAATTTGAAAAGCCGACTCGTGACG
AAATTCAGCGAATGCAGAATGAGGCGCTTGAAGGTCTGGAAGATATTTCTGAACAAATCCTGGAAGCAATGAAGCCGTATGG
CATTCAGCAGTTGGGTATCTATTATCGTGACAAACGCGGTGTTGAAATTCCTGCGCCTGATAAAAAAGAACGTGAAGAACTTG
CTGAAGTCGATGAATCAGACATTTTTGACGAAGCCATTGTTATCGAACGCAACGAGCCTGAACCTTCGCAGGCTCACGCTTAT
TCAAAAGCGCTGGAGTTCCTTTATTTCCTCGCAAATATGGAATGGGCCATCGTGCCTGTTTGCCGTGATCGTATCCGTGAGTAC
ATCATGGACAACCGCCCTGTTAGCTCACTGTGGGGGGATGTTGTCCAGATCAGAACGGTAGATCACAACTTCTATACCACCGG
CATTGAATTTCGTGAATACGAAGAAGATACAGAGCCAGGCCAGCTTAACATGCTTAAAGAAGCCGATTTTGAATACCTTCTGA
CGCAGAGTTTTTCTTGCCTCTCTGAATCTTCAGCTAAAACGTTTCTGACGCATCAGGAAAAATCTTTGCAGGAAACGCGCGAC
CGTGCGCAAAGCCAGCTGGCACAGCTTGGTACCGCGCTCGATATGCTGACGTCCAGAGAGTTCGTGATGGGCTACCATCATG
GAACCGTGCATGTCTGGGATAATGACCAAAACGCGGTACAGCGCAAAGCGCGTCGTGTGAAGGTTATGCTAACCGGCTGTG
GCGTGGTTGGCGGGACTCTCAGCCTGGCCTCTGAGGCTGCATATTATGCGAGACTGCCTGGCAACCAGAAATGGGCGCCGC
GCCCGGTTCCGATAAACTCATGGAACTTCCTGCACTTCAGCCCGTTCCACAATTTTATGCGTGGCAAGCCTGACAATAACCCGT

GGGGGCCAGCGCTGACCATGTTCCGCACGATCAGCGGTACGCCACTCTATTTTAATTTCCATGTGACCCCGCTTGAAGAACTT
TCCTACGGTAAACGCCCGCTGGGCCATGCGTTAATAACGGGTATGTCGGGGGAAGGTAAAACCACGCTGCTTAACTTCCTGCT
GGCGCAGTCAATGAAGTACAACCCGCGGCTTTTTGTTTATGACCGTGACCGCGGTATGGAGCCGTTCATTCGAAGCGTTGGT
GGCTACTATAAAGTTCTGCAACAGGGTATGCCGTCCGGGTTTGCCCCGCTTCAGATTGAACCGACCAAACGCAATATTGCCCT
CATTAAAAACCTGTTCCGCATTTGTGTGGAAACCACCAATAACGGGCCTATCAGCGCAACGATGGCTACCGAACTGGCTGAAG
GCGTTGATGCGGTTATGGGGGAAGGCTCACTTATTCCACGCGAGGCGCGCACCGTTACTATCCTGGACGGGTACGTGAATGA
AGTTGTGGAAAATGGCGTATCACTGAAAGGGCTGCTGCGCGAATGGACGCGCGAAGGCCAGTATGGCTGGCTGTTTGACAA
TGATAAAGACAGCCTGGATCTCAGCGCGAATGATATTTTTGGCTTCGATTTATCCGAGTTTATCGCAGCCAAAGAGGAAGTATC
CAGCCCGGCCCGTACTCCGCTCATGATGTACCTTCTGTACCGGGTACGTGACTCCATCGACGGCAAACGCCGCGTCATTCAGT
GCTTTGACGAGTTCCACGCCTACCTTGACGATCCGGTTATCGAGCGTGAAGTTAAGCGTGGTATCAAAACTGACCGTAAGAA
AGACGCTATCTATGTGTTTGCCACGCAGGAGCCGAACGATGCGCTGTCCAGCCGTATTGGCCGCACGATCATGTCGCAGACCG
TCACAAAAATCTGCCTGCGCGATCCGGAAGCTATCCGAGAGGATTATGCCTTCCTTACTGATGCTGAATACGACGCGCTGATGT
CGATTACCGAACACTCCAGACAGTTCCTGGTTAAACAAGGGCAACAGTCTGCGATTGCTTCTTTCAATCTCTACCCTCGCAAC
AGCGACGATATTGATGCAGATATTAAGACAATGGACAACGTTCTTAGCGTGTTGTCCGGTGAACCACAAAACGCCGAAATTGC
GCATGAGCTGGTTGAACGGCTCGGTAATGACCCTGAAGTATGGCTCAAAGAATACTGGCGCCTGACGGCTTAACAACGAGGC
AAAACACCATGAAAAAAACACTGACGGCAGTATTGCTGACCACCGGCCTGATACTGGGAGGCGCGCAAAGCGCTTCCGCAG
GCATCATCGTGACCAACCCTACTGAGCTGGCTAAACAGGTCGAGCAGCTTCAGCAAATGGCGCAGCAGCTGGAGCAGCTTA
AAAGCCAGCTGCAAACGCAGAAAAATATGTATGAGTCGATGGCAAAGACAACCAACCTGGGCGATCTGCTGGGGACGTCTA
CCAGCACGCTGGCAAATAATTTGCCGGACAACTGGAAGGAGATCTACAGCGACGCCATGAACTCCAGTTCTTCCGTCACGCC
TTCAGTTAACAGCATGATGGGCCAGTTTAATGCGGAAGTTGACGACATGACGCCCAGCGAAGCAATTACCTACATGAACAAA
AAGCTGGCTGAAAAAGGCGCTTATGACCGTGTTATGGCAGAAAAAGCCTACAACAACCAGATGCAGGAACTAACCGATATGC
AGGAGCTGACGGAGCAGATTAAAACGACTCCAGACCTGAAATCGATTGCTGACTTACAGGCCCGTATCCAGACGTCACAGG
GTGCTATTCAGGGTGAGCAGGCGAAGCTGAATCTGATGAACATGTTGCAGCAGTCACAGGACAAGCTATTACGTGCGCAGAA
AGAACGTGCCACCCACAATTTTGTTTTTGGAACCGGCGGGGACGTTACCGCGTCACCTTCAATTAACTGAGGTAATTATGAAA
AAACTACTGCTTGTTATCCCTTTCCTCCTAGTGGCCTGCGATGCCTCGCATGACGTGGAGTGGTACAAAAAACATGAGAAAGA
GCGCAAGGCAACAATTCAGGAATGCAAGAAAGACGCGGATGAACTTCAGAAACCTGATTGCAAAAACGCGCGCGAAGCCG
ATCGTCAGCTGTTTGTGTTCGGCAAAAAAGACGGCGAAATCAATTCACCGAAAATTTAGGAGTAAGGAGGCAATATGGCATT
CACCCTAGTCGCAGACATTTTCGCAAAAGTAGACGGGGCGATTACGTCAATGGTGAGCGCCAATGTTGCCACCATTATCTCTG
ATGTAACGCCTCTGATTGCCACCTGTCTGACAATCAAGCTGATGGTTCAGGGGATGTACTCAGCGTTTAATCCGGGGGCGGGC
GACAGCCTGAGTTCGCTGATTAAAGAGTATCTTTCCATAGCCCTTATCCTGAGCTTTGCAACGGCGGGCGGCTGGTATCAACA
GGAACTGGTCAACGTGGCGCTTCACCTGCCGGATGATTTTGCCGGGATACTGTCTGCCCCTAATAAAGTCGGTGCAAGTGGC
GTACCGGCGATTATTGATAGCGGTATTGAAAAAGGTATCAAGATCGTCAACACCGCATGGGAAGCCGCAGACGTGTTTTCATC
GAGCGGCCTGGCCGCGTATGCCATTGGCGGCATTATGATGATTGCTACCGTTGTGCTGGGCGGCCTCGGTGCGGGCTTTGTG
ATCATGGCTAAGATCCTTCTGGCCGTTACGCTTTGTTTTGGCCCGATTGCAATCTTCTGCCTGCTGTGGGGAGCGACAAAAAA
CATCTTTGCTCGCTGGCTGGCGTCGGTCATTAACTATGGCCTTGTCGTCGTCATTCTTGCGCTCGTGTTTGGTTTCATCATGCAG
ATGTTCGACAACCTCCTGTCCTCGATGAACTCTGATGCCGCTTACTCATCAATCACTGGTTCTATCTCCGCCTTATTACTGACGGT
CATTTCCGTTTTCGTTCTGTTCCAGATTCCGCAAATTGCCGCCAGCTGGGGTAGCGGTATCAGCGCCGGAGTTGCTGACGCCG
CACGCTCTACGGGTTCTTCCATGCAGGCGCTTGGCAATATGGGCAGCCACGGCATGTTTGGCGGTAATGCGTTCAGAGGCGG
TAACAGTGGCGGCGGCCAGCAATCGGCAGGTGGAGGAAGTGGCAGCAACAGCGGAGGAAGCAGTGGTTCTAATTTAAGTG
GTAAGGCAAGGGGCAGTCGCGGGAAGAAGGCTGCATAAAATTAAAACAAAAGTCGTTGAAATTGCAATTTCGACGACTTAT
TATAATTAGTACGTTCAACAACCGATAATGGATGCCGTAATGCGCAGCTTATTGCTTATGGGAGTTCTTCTGATTAGCGCCTGTT
CCAGCGGGCATAAACCGCCACCGGAGCCGGACTGGAGCAACACCGTTCCAGTAAACAAAACAATCCCGGTTGATACGCAAG
GTGGTGCAAATGAAAGCTAATAAAAAAACAGGGCTTACACGTGAAGCCATTAAAGAGTTCAACGAAAGCCGTAAAGGGCTT
GAAGTTGATCTGATGGATGAAGTGCTGAAGTCCCGGCGTACCGCCTGGATGGTTGCCACCGGTTCAGCGGTGGTAACTGTTT
TTGCACTCTCTTTAGTTGGTTACGTGGTGCATAAGTACAGCCAGCCAATCCCCGCACATCTGCTAACGCTCAACGAGGCCACTC
ACGAAGTACAGCAGGTCAAGCTGACCCGCGACCAGACCTCTTATGGTGACGAAATTGATAAGTTCTGGCTGACACAATATGTC

ATTCACCGTGAGAGCTATGACTTCTATTCAGTTCAGGTCGACTATACGGCCGTTGGCTTAATGTCCACGCCGAACGTGGCAGA
GTCTTACCAGAGCAAGTTCAAGGGCCGCAACGGTCTTGATAAGGTTCTGGGCGACAGTGAAACGACCCGCGTGAAGATTAA
CTCTGTGATCCTCGATAAACCGCACGGCGTAGCAACGATACGCTTTACTACGGTTCGCCGCGTGCGCAGCAATCCCGTTGATG
ATCAGCCGCAGCGCTGGATTGCCATTATGGGGTATGAATATAAATCGCTGGCGATGAATGCTGAGCAGCGTTATGTCAACCCG
CTGGGTTTCCGCGTGACGAGTTATCGCGTCAACCCTGAAGTTAACTGAGGGCTGCCCCATGAAAAAACTACTTCTTTCAGCAG
TCGTTTTGTCAGTCCTGGGAGGCGCGGCCACTAACGTTATGGCGCTTGAGGTTGGCCGCAATTCTCCTTATGACTATCGCATTA
AAAGCGTTGTTTATAACCCTGTTAATGTGGTCAAAATTGACGCTATCGCCGGTGTGGCTACCCACATTGTTGTCGCGCCTGACG
AAACCTATATCACTCATGCTTTTGGCGATTCTGAAAGCTGGACGTTTGCGCACAAAATGAACCATTTTTTTGTGAAGCCGAAA
CAGGCCATGAGTGATACCAACCTGGTGATCGTCACCGATAAGCGCACCTATAACATCGTCCTCCATTTCATCGGTGAAGAAACG
AAGAAAAATGCAGACGGTACGGTATCAAAATCCTTTATTGAAACGCCGTGGGCTGTGCGCCAGGCCGTTCTTCAGCTGACCT
ATGAATATCCGTTTGAGCAGCAGGAAAAAGCCAAAAGCGCGGCTGATAAAAAACGCATTACGCAGAAGCTGAAGCAGACGG
CTTTTGCGGGGGCGAAGAACTATCAGTACGTAATGAGCGAACAGCCTGAAATGCGCAGCATCCAGCCGGTTCACGTCTGGG
ATAACTACCGCTTTACCCGGTTTGAGTTTCCGGCCAATGCGGAGTTACCGCAGGTCTACATGATTTCGGCCAGTGGCAAAGAA
ACGCTGCCTAACTCTCATGTTGTGGGTGAGAACCGCAACATCATCGAGGTGGAAACCGTCGCTAAAGAGTGGCGTATTCGTCT
GGGCGATAAAGTCGTTGGCGTTCGTAATAATAATTTCGCGCCGGGCGCCGGTGCGGTAGCAACCGGTACGGCTTCCCCGGAT
GTGCGCAGGGTTCAAATTGGGGAGGATAACTGATGGCCCGTAAAAGTGTCGATGTAGATCAGGAACTCGATGAAAACACCG
GAGACGGTGAATTCGAAAGCGAGCGTGGCGGATTTAAAGGCAGTAACCGCCGTTCGGCTCCTGGTATGAAAGCCTTTGTCAT
ACTGATGGCGCTGCTTGCTTTGGTATTCATCGGGATTACGGTCATGGGTAAAATTCGCACCCCGGCTAAAGCTGAAGCTGATA
AAGACGGTGGTAAAGCGCAACAGGCCAATACACTGCCAAACTACAGCTTTAACAGCGATCCTGATGTTAATAAACCTGCAACT
GCGCAGAATAGCGCCACTGATGCCCGTGCTGTGCAGGCTGCCGCACAGGCAGATGCAGATGCGGGCAGCAGCAATACCGCC
GCGCGTACCTCTAATAAGCGTAAAGAACCTTCGCCTGAAGAACTGGCTATGCAGCGTCGTCTGGGCGGCGAGCTGGCCCAGA
CTAATCAGGCGGCTACAAGCAATAGTCCCGGAGTGCAGCCCCAGGACAACGAAACAAGCGAAGGTAGTTCAGCACTCGCTA
AAAACCTGACTCCTGCAAGGCTGAAGGCTAGCCGCGCTGGAGTCATGGCTAATCCCAGCCTGACTGTTCCGAAAGGCAAAAT
GATCCCCTGTGGTACCGGCACCGAGCTGGATACCACTGTTCCGGGTCAGGTTTCCTGCCGGGTTTCACAGGACGTTTACTCA
GCTGATGGACTCGTTAGGCTGATTGATAAAGGCTCATGGGTTGACGGGCAGATTACCGGTGGTATCAAAGACGGCCAGGCG
CGCGTGTTTGTTCTCTGGGAGCGTATCCGCAATGACCAGGACGGGACAATCGTTAATATTGACAGTGCCGGAACGAACTCAC
TCGGCAGCGCGGGGATTCCGGGCCAGGTGGATACCCATATGTGGGAGCGTCTGCGTGGTGCGATCATGATTTCGTTGTTCTC
TGACACCTTAACGGCGCTGGTTAACCAGACGCAGAGTAATAACATTCAGTACAACAGCACAGAAAACAGCGGTGAGCAGCT
GGCGTCTGAAGCACTCCGCTCTTACATGTCTATCCCCCCTACCCTCTACGATCAGCAGGGTGATGCGGTGAGCATTTTTGTTGC
CCGCGACCTCGATTTCAGCGGCGTTTATACGCTCGCAGACAACTAAAAAAGTGGGCGCTTAGCGCCCGCTTTTCTTCAGGAG
TAATCATGACTGATGCAGCTTTCTATCAACTTGGCCCACTGCGCGAGTATTTAGAAGATCCTACTGTTTTTGAAATTCGCATTAA
CTGCTTTCAGGAAGTTATCTGTGATACGTTCAGCGGCCGCAGGGTTGTGCAGAACGCGGCAATTACGGCAGATTTTATTAGG
AACCTTGCTAAATCGTTGGTGAGCAGCAACAAGCTGACCATGCAGGCCATTAATGACGTGATCCTGCCTGGCGGGATCAGGG
GCGTTATCTGTCTGCCCCCTGCGGTGATTGACGGTACAACGGCCGTAGCGTTTCGTAAGGATTTGGCGGCCGATAAAAATCTG
GAGCAGCTGACCCGCGAGGGGATTTTCAGTGACTGCCGGAAGATTACCGGCAGCAAGCAAAGCCTAACGGATGATGATTTT
TTCCTTAAAGAGCTGCACAGCAGCGAAAAATGGCCCGCATTCCTGCAAACCGCCGTTGAGAAGAAACGCACTATCGTGATCT
GCGGTGAAACCGGGTCGGGGAAAACGGTACTCACGCGCGCGCTGTTAAAATCGCTACATAAAGACGAGCGTGTAATTATTTT
AGAGGACGTTCACGAAGTCACGGTCGATCACGTTGTAGAAGCCGTTTATATGATGTACGGCGATGCAGGAAAGATCGGCCGC
GTCAGCGCCACTGATGCCCTGCGAGCCTGTATGCGTCTGACACCGGGCCGTATCATCATGACTGAGCTTAGGGATGATGCTGC
GTGGGATTATCTTAAAGCACTTAATACCGGCCATCCAGGCGGTGTTATGTCAACGCACGCTAACTCTGCGCGCGATGCCTTTAA
CCGTATTGGGCTGCTTATCAAGGCGACCCCTATCGGCCGTATGCTCGATATGAGCGATATTATGCGAATGCTCTACTCCACCATT
GACGTTGTGGTGCATATGGAAAAGCGGAAAATCAAAGAAATTTATTTTGACCCTGAATATAAAATGCAGTGTGTGAACGGGA
GCCTGTAATGAAAAACTTAGCAACCTGGCTTCTGGCCGCAGCATTTACGACAGCCGCCCTGCCCGCCTTTGCGGTGGAACCA
TCCGTTCAGGTTGGCTACTCGCCTGAAGGGGGGGTAAGCGGGAACCCCAGAAAATTCCGCCATTCCGCATTGTGGAATTTTT
TGGGGGGGTGGTCCGCGGCATGACGACCCGCCAAAGGCTACCGCATCTATCTGAGGCGCTGTGGCGCGACGCTGAAACCCC
GGGGAATTCCGCTACTCCAGTTCCAGGTCCGCAATAGTTCGTCCAGGTCGGGTGGCGTACGCCGGAAACGCCGATTTTTCCG

CAACCGTTTCTGCGTTGTCGGCAAGGTGGTCTCAGAGAGCGCTCAAGAAGCGGTTTTTCTCGTATGTTTAGCTACGGTGGCC
TAGGAGCCTGCGCGGCAGAAGCCAAATCGGCGGAAGATCGAGGACGATACGGGGATGGCTGCCAGTTACCTTCCTTACCGA
CCCGACCAATCCTATCTGCTGCCCCCTTCTCTGGGAGAGTGGCTACCTGAAGGGCATCTTGCCTACTTCATCAGCGAGACTGTC
GATACGCTGGACTTGAGCGCATTCCACGCCCGGTATGCCGGCGGTGGTCCGGGCAATCAGCCGTTTCATCCGGCGATGATGG
TCAAAGTGCTGATCTATGGTTACGCGAGCGGCGTCTTCTCTTCGCGCAAACTAGCCAGGAAGCTGTACGAGGATGTCGCGTT
GCGTGTGCTGGCCGCTGGAAACTTCCCGGCCCACCGCACGCTGAGTGACTTCCGTGCCCTACACCTGACCGAGCTTGAGAAT
TTGTTCGTTCAGGTGGTGCAACTGGCGCGCGAATGTGGGCTGGTGAAGCTTGGCACGATCGCGGTCGACGGCACCAAGGTA
AAAGCCAACGCCAGCCGCCACAAGGCGATGAGCTATAAGCGCATGAAGCCGGCCGAGGACGAATTGCATTGCGAGATCAAG
GCGCTGCTTGATCGCGCCAAGGCTACCGACGACCAGGAGCGTAACGAGCCGGAGCTGGACATTCCTGCCGAGATTTCTCGC
CGCGAGAAGCGCCTGGAGGCGATCCAGGCGGCAAAGGCGCGCCTGGAAGGGGTCGGTTCCGGCTGAGGGCGAAATGACA
CCCTAAGCTTTCGGTTCCTTGGGCCAAAGATATTCGCCAGTCAGTAGAATGTGCGCCCAGCCCAATGGGGATATGTGGGGAA
GAAATTCAGGGGGAACATCCAACCCTTCGTTCCGCCGCTCCGTGACGGCATGACCAAGATGGACGGTATTCCAGTAAATGAT
CACCGCAGTCAATAAATTGAGCCCAGCGATTCGGTAGTGCTGCCCCTCTGTCGTGCGATCGCGAATTTCCCCCTGCCTCCCGAT
ACGGAGCGCATTTTTGAGCGCATGGTGGGCCTCTCCCTTGTTAAGACCGATCTGAGCACGCCGCTGCATGTCCGTATCCAGGA
TCCACTCAATAATGAAAAGGGTCCGTTCAATACGACCAACTTCACGAAGCGCAACTGCAAGGTTGTTTTGTCGTGGGTAAGA
AGCGAGCTTGCGCAGGAGTTGGCTGGGCCTGATTTTGCCAGCGGTCATCGTCGCGGCACAACGGAAAATATCAGGCCAGTT
CGCAACGATAAGATCCTCCCGGGCTTTTCCACCTACCAACTTGCGTAACTCCCTGGGGGTCGTATCGGGATTAAATACGTACAA
CCGCTTCGATGGCAGATCCCTGATTCGCAGAACGAGATTGTAGCCGAGCAGGCTACTGGCTCCGAACAAATGGTCGGTGAAT
CCTGCTGTATCGGCATACTGTTCGCGAACATGGCGACCGACCTCGTTCATCAGTAGTCCATCGAGAATATACGGTGCCTCGCTC
ACGGTCGCCGGGATCGACTGACAAGCGAATGGCGCGAACTGGTCGCTTACGTGAGTATACGCTTTGAGGCCGGGAACAGAA
CCATATTTGGCATTGACCATGTTCATGGCTTCGCCATGCCGCGCTGTCGGGAAAAACTGACCATCGCTCGATGCTGACGTGCC
CATCCCCCAGACGCGTGACATCGGCAGTTTACCCTGCGCGGCCACCACAATTGCCAATGCCTGGTTCATGGCTTCGCTTTCAA
CATGCCAGCGGGCAAGGCGTGAGAGCTGCCAGTAATCATGCGTGTTTGTAGCTTCCGCCATCTTACGCAGGCCCAGATTGAG
CCCTTCAGCGAGCAGGACGTTGAGCAGACCGATCCGGTCGCGACATGGAGCCCCGGTTCTCAGATGGGTAAACGCATCTGT
GAAACCAAGGGCTGCATCAACTTCAAGCAGCATGTCGGTAATCCGAACGGACGGCATTCGGCGATACAGATCCAGTATGAGT
GCCTCGGCACCATCCGGCACGTCTGCTGTCAACCTGTCGATCCGCAACGTTCCATCTTCTATGCTACCGTGCGGAATAGTGCCG
TTACGGGCAGCCCGGGCCAGCCGCTTAAGAGCGATCGTGAGTCGCGCCTTTCTGTCTGCCAGCCAATCCTGTGGGTTGGAAG
GCACGGCCAGTTTTGCATTTTCCTGCGCCGCGATCATCGGCACCAGTACCTGCTTGAGGTCACCATAGCGGCGCGAATGAGC
GAGCCAGACATCTCCGGAACGAAAAGCATCCCGGAGGTGAAAGAGTACCGCCACTTCCCAAAGACGGGTATCTCCTTTTTCC
TGAGCTCGTAAATGACGGTTCCATTTGGAGCTGGGCCGCAGGAAACGCCTTTCTGGCGATGCAACACCTTTCATCTCTCCGAT
CGACAAAGCTGCTGCTACCAATGGTCCGGCGACCGGCGCGGCTTCGAGCTTCAGACAGCGCAACATGCGGGGCGCATAACG
ACGAAAGCGATGGTATCCCTGCCCGACATATGCAAGAGGCTCATCGGCTAGCGTGTTGCTGAGTTGAGTCCCTGTCGCTACCA
GTTGAGCGAGCCGGTCCCATGCAACCGAACTGGCGACAGCCATCTCCAGCGGGGTTCCGTCACTGCGGGCCTCAAGCAACG
AAGCTCCCAGCGCGGTGAAGGTACGGATCGTATCCGTGAGTGTGGCTTTAGAGCCGGAAATTGTTTCGTCATGCTGGCGCTT
CGCTTCCCGCCAGGTTTTTCCTACGATCCTGTCATGGGTTTCGACTATGGCATCAGCAATCGCCGCTTCCCACTCCACAACACA
GACGGCAAGGATCGCCCAGCGGCGGTCCGAAGTGATGTCACGCAAACCGTCGGTGAAGTAGCGTTCACCCTGCCGACGCA
GCCGGGCAATGCGATGGGCAGGTATGCTGGCCAAAGCACTATGATTGATATTCAGGGTACGCAGAAATTCGAGCCTGTCGAG
CAAACGGTTAGCAGCAGCCGAGTTGTTACCAACCTCGAAGTTGCGAAGCCAGATGAAACGACTGATATTGCCGGCGAGCATT
TCACTCAGAAGTTTGTCCAGGTGATCGCGAACATCCGCTGTTAAATTTTCCACAATCCGCGTTTCAATCCGCCGCTCAGCGGC
GACCAGAGCATCCGCGCACAAGCGCTCGATTGTCGATACTGCGGGCAGAATGGTGGAAGTTTCCCGACACCGCACAATAAA
ACGATGAGCAAGATCCTCGTTTGATCTGGCATCTTCGGCCTGGCCGAAAGTCCACTCCCGCAGATCACGGGCACCACGGCCC
GTGAAGGTCTTGTAGCCGTAAATTTCGCGCAGCGTGTCCATGTGCTGCTGACGGGTTTGGCGCCGTGTGGCATAAGTGAGAA
GCGCATCAGCCGGAACTCCAAGCTGAGCACCGACGAAGGAAAGGACTTCACGCGGGATCATCTCACCAGGAGCCAGTGCA
CGGCCCGGATATCGTAAGGCACAAAGTTGCAGGGCAAAGCCAATCCTGTTTTCCGGTCTGCGGCGCTGCCTAATGTTTTCCA
GGTCATCATCGCCCAGCGTGTAGAACTTCAGTAGCGACAGTTCGTCCGTGGGCAGATCGAACAGCGCTGCTCGCTGCCGTTC
GGTGAAAATATGGCGTCGTGACATACAAATTCGTCCCTTTTGAAGTATAGTCTGTTTTGGACAACAGCCAGCCCATATAAATCA

GGGCGTTCCGATACAAAAATCCAGGAGGGTTCAATTGGGACATCGTGCCGCCATTTACTGCCGGGTTTCAACAGCGGATCAG
TCTTGTGAACGCCAGGAATTTGATCTGCGAGCCTTCGCCGGCCGTGCCGGCTACGACGTGGTGGGAATATTTAAGGAAACAG
GTTCAGGAACTAAACTCGACCGGGCCGAGCGAAAGAAAGTCCTGGCGCTTGCCCAGTCCAGACAAATTGATGCAATCCTGG
TCACTGAGCTTTCCCGGTGGGGGCGCTCGACGCTCGATCTGCTCAATACGCTACGTGAACTGGAGAACTGGAAGGTTTCCGT
GATAGCCATGAATGGAATGGCGTTCGATCTTTCGTCGCCGTATGGACGAATGCTGGCGACGTTTCTTTCCGGCATTGCGGAGT
TTGAGCGGGATCTCATCAGCGAGCGGGTCAAGTCAGGCCTTGCTGTTGCGAAGGCACGTGGTAAGAGGCTTGGTCGTCAGG
CCGGAGTGCGACCAAAATCAGACCGACTTTTGCCTAAGGTGGTTGCGATGAGGGCCGAGGGACGCAGCTATCGCTGGATCG
CACGCGAGCTCGGTATCAGCAAGAATACCGTCGCTGACATCGTGCAACGACACAGAGCTAACGCTTAGGGTGTCATTTCGCC
CTCAGCCGGAACCGACCCCTGGAAGCGCGCCAGCGTGAAGCGGACCAGGCCCGGGGGCGCAGCGAAGACGATGGCCGCC
GGCCTCGCCATCCGGATGGCTCGGACAAGGGCGGTGGCTCGTACAAACGCGAGTTTGGTGTGCCGGATGACCGTGATCAGG
AAAGCTTCACCGATCCGGACAGCCGGATCATGAAACACGCCGGTGGTGGCTCCGAGCAGAGCTACAACGGGTACACAGCGG
TCGATGCCGAGCACCAGATCATCGTGGCGGCGGAGTTGACCAACTGCGCCGCGGACAGTCAGGCGCTGCTGGGCATGCTGG
CAGCAGTTCAGGCCAACACCGGAGAAATGCCGGCCCAGACGCTGGCGGATGCGGGATTCCGTAGTGAGGCTGTTCTGGCA
AAGGTCGCCGATCACCACGGCGATGTCATCGTTGCCCTCGGCCGCGAGGGACGTGAAGATGCCAAGGTCAATGCCAAGACC
CATCCGCATACGGCGGCGATTGCGGCGAAATTGAAAACGGAGCAAGGCGATGCAGCTTACCGCCGGCGCAAGTCGATCGTG
GAGGCTCCGAATGGTTGGATCAAGGCGGTGATGGGATTGCGTCAGTTCAGCATGAGGGGCCTGGACAAGGTGCAAGCCGA
GTGGAAGCTCGTCTGCATGGCGCTAAATCTGAGGCGAATGGCGTATCTGTGAGGGCGAAGGTTAAATGGGGCGGCTCAAAT
GCACCCCAGTCGTCATAACACGCCGCGCGCCGCAAGATTGGTATCCTTGGCGCCGACGCCTTGCCAATTGCAGAGAGCGCCG
CCGCCATCGTCAGTGCTCTACAGAAAACCGGTCACACGGCTCTGCCGCGCAGACTCCTAGCCTAAATGTGACAGTGGTTGGT
AATCCATGCCGCCCGCAAGGGCGGCGGTGGTGGGCCAATAGATGATTTTCAGAGCCTTACTGCCCGTTGACGCCCAATCCCT
CGAGCGCGAGTCTAGCCGCAGCGGCGATGACGGCCTCGCTGTGCTTGTCATCCTTGTTAGGCGCCCGGGTGTAGACGGCCA
ACACAATAGGTGCGCGCCCAGTGGGCCAGACGACGGCATAGTCATTTGCCGTGCCATACACTCCGCAGGTTCCGGTTTTGTC
TCCGACTGCCCAGTCTGCCGGCACCGCCGCGCGGATGCGGTGGTTGCCGGTCGTGTTTCCCTTTAGCCAATCAACAAACTGC
TGCCGCTGCGGCGCAGCCAGTGCAGAGCCCAGTGTCAGTTTTTGTAAGCTTTCCGTCACGGCGCGCGGCGATGAGGTATCG
CGCGCATCGCCTGGGATGGCGGAGTTCAGCTCCAGCTCCCAGCGGTCCAGACGGAACGTGGTATCGCCGATAGAGCGCATG
AAGGCCGTCAGCCCGGCCGGGCCGCCCAACTCCTTCAGCAACAAATTGGCGGCGGCGTTATCACTGTATTGCACGGCGGCC
GCGGACAGCTCCGCCACCGTCATGCCTGTTGTCAGATATTTTTCCGAGATGGGTGACCACGGAACCAGCGCATTTTTGCCGTA
ACGGATGGGTGTGTCCAGCAAGCCGGCCTGCTGCTGGCTGCGAGCCAGCACAGCGGCAGCAAGAAAGCCCTTGAATGAGC
TGCACAGTGGGAAGCGCTCCTCAGCGCGGTAACTTACAGTTGCGCCTGAGCCGGTATCCATCGCGTACACACCGATGGAGCC
GCCAAAGTCCTGTTCGAGTTTAGCGAATGGTTCCGCGACGAGGTTGGTCAGCGCGGTGGCAGAAAAGCCAGCCAGCGGCC
ATGAGAGACAAGACAGCAGAACTAGACGGCGATACAGTGACATCAACGATATTCCTTGTTTGAAGGTGGAGTTACGGACGG
CCTCAGGAAGTCCTGGCCAAGCCCCGACTATTGGGGCGCGAAGATAGCACCGAGGGTACGCCGGTGTCAACACGGGGTCTG
ACGCTCAGTGGAACGAAAACTCACGTTAAGGGATTTTGGTCATGAGATTATCAAAAAGGATCTTCACCTAGGGAAGGTGCGA
ACAAGTTCCTGATATGAGATCATCATATTCATCCGGAGCGCATCCCAGAGGGACATCATGAGCCATCAACTCACCTTCGCCGAT
AGTGAATTCAGCACTAAGCGCCGTCAGACCCGAAAAGAGATTTTCCTCTCCCGCATGGAGCAGATTTTGCCATGGCAGAATAT
GACCGCTGTCATCGAGCCGTTTTATCCCAAGGCGGGCAATGGCCGACGGCCCTATCCGCTGGAGACCATGCTGCGTATTCACT
GCATGCAGCATTGGTACAACCTGAGCGACGGTGCCATGGAAGATGCTCTGTACGAAATCGCCTCCATGCGCCTGTTTGCCCGA
TTATCCCTGGATAGCGCCCTGCCGGATCGCACCACCATCATGAATTTCCGCCACCTGCTCGAGCAGCATCAACTGGCCCGTCAA
TTGTTCAAGACCATCAGTCGCTGGCTGGCCGAAGCAGGCGTCATGATGACCCAAGGCACTTTGGTGGATGCCACCATCATTG
AGGCACCCAGCTCTACCAAGAACAAAGAGCAGCAACGCGATCCGGAGATGCATCAGACCAAGAAAGGCAATCAGTGGCACT
TTGGCATGAAGGCCCACATTGGTGTCGATGCCAAGAGTGGCCTGACCCACAGCCTAGTCACCACCGCGGCCAACGAGCATG
ACCTCAATCAGCTGGGTAATCTGCTTCATGGAGAGGAGCAATTTGTCTCAGCCGATGCCGGCTACCAAGGAGCGCCACAGCG
CGAGGAGCTGGCCGAGGTGGATGTGGACTGGCTGATCGCCGAGCGTCCCGGCAAGGTAAAAACCTTGAAGCAGCATCCGC
GCAAGAACAAAACGGCCATCAACATCGAATACATGAAAGCCAGCATCCGTGCCAGGGTGGAGCACCCGTTTCGCATCATCAA
GCGGCAGTTCGGCTTCGTGAAAGCCAGATACAAGGGGCTGCTGAAAAACGATAACCAACTGGCGATGTTATTCACCCTGGCC
AACCTGTTTCGGGTGGACCAAATGATACGTCAGTGGGAGAGATCTCAGTAAAAACCGGAAATAACGCCAGAAATGGTGGAA

AAAATAGCCTAAATAGGCTGATTCGATGTGTTTGCGGGAAAAAAATCGGCCCAGATCCGCGAAATTTTAATCAGCGAGTCAGC
TTGGGAAGAAATGACCTGCTTATTCGCACCTTCCCTAGATCCTTTTAAATTAAAAATGAAGTTTTAAATCAATCTAAAGTATATAT
GAGTAAACTTGGTCTGACAGTTACCAATGCTTAATCAGTGAGGCACCTATCTCAGCGATCTGTCTATTTCGTTCATCCATAGTTG
CCTGACTCCCCGTCGTGTAGATAACTACGATACGGGAGGGCTTACCATCTGGCCCCAGTGCTGCAATGATACCGCGAGACCCA
CGCTCACCGGCTCCAGATTTATCAGCAATAAACCAGCCAGCCGGAAGGGCCGAGCGCAGAAGTGGTCCTGCAACTTTATCCG
CCTCCATCCAGTCTATTAATTGTTGCCGGGAAGCTAGAGTAAGTAGTTCGCCAGTTAATAGTTTGCGCAACGTTGTTGCCATTG
CTGCAGGCATCGTGGTGTCACGCTCGTCGTTTGGTATGGCTTCATTCAGCTCCGGTTCCCAACGATCAAGGCGAGTTACATGA
TCCCCCATGTTGTGCAAAAAAGCGGTTAGCTCCTTCGGTCCTCCGATCGTTGTCAGAAGTAAGTTGGCAGCAGTGTTATCACT
CATGGTTATGGCAGCACTGCATAATTCTCTTACTGTCATGCCATCCGTAAGATGCTTTTCTGTGACTGGTGAGTACTCAACCAAG
TCATTCTGAGAATAGTGTATGCGGCGACCGAGTTGCTCTTGCCCGGCGTCAACACGGGATAATACCGCACCACATAGCAGAAC
TTTAAAAGTGCTCATCATTGGAAAACGTTCTTCGGGGCGAAAACTCTCAAGGATCTTACCGCTGTTGAGATCCAGTTCGATGT
AACCCACTCGTGCACCCAACTGATCTTCAGCATCTTTTACTTTCACCAGCGTTTCTGGGTGAGCAAAAACAGGAAGGCAAAAT
GCCGCAAAAAAGGGAATAAGGGCGACACGAAAATGTTGAATACTCATACTCTTCCTTTTTCAATATTATTGAAGCATTTACCAG
GGTTATTGTCTCATGAGCGGATACATATTTGAATGTATTTAGAAAAATAAACAAATAGGGGTTCCGCGCACATTTCCCCGAAAA
GTGCCACCTGACGTCTAAGAAACCATTATTATCATGACATTAACCTATAAAAATAGGCGTATCACGAGGCCCTTTCGTCTTCAAG
AATTTTATAAACCGTGGAGCGGGCAATACTGAGCTGATGAGCAATTTCCGTTGCACCAGTGCCCTTCTGATGAAGCGTCAGCA
CGACGTTCCTGTCCACGGTACGCCTGCGGCCAAATTTGATTCCTTTCAGCTTTGCTTCCTGTCGGCCCTCATTCGTGCGTTCTA
GGATCCTCCGGCGTTCAGCCTGTGCCACAGCCGACAGGATGGTGACCACCATTTGCCCCATATCACCGTCGGTACGGCACTGT
TGCAAAGTTAGCGATGAGGCAGCCTTTTGTCTTATTCAAAGGCCTTACATTTCAAAAACTCTGCTTACCAGGCGCATTTCGCCC
AGGGGATCACCATAATAAAATGCTGAGGCCTGGCCTTTGCGTAGTGCACGCATCACCTCAATACCTTTGATGGTAGCGTAAGC
CGTCTTCATGGATTTAAATCCCAGCGTGGCGCCGATTATCCGTTTCAGTTTGCCATGATCGCATTCAATCACGTTGTTCCGGTAC
TTAATCTGTCGGTGTTCAACGTCAGACGGGCACCGGCCTTCGCGTTTGAGCAGAGCAAGCGCGCGACCATAGGCGGGCGCT
TTATCCGTGTTGATGAATCGCGGGATCTGCCACTTCTTCACGTTGTTGAGGATTTTACCCAGAAACCGGTATGCAGCTTTGCTG
TTACGACGGGAGGAGAGATAAAAATCGACAGTGCGGCCCCGGCTGTCGACGGCCCGGTACAGATACGCCCAGCGGCCATTG
ACCTTCACGTAGGTTTCATCCATGTGCCACGGGCAAAGATCGGAAGGGTTACGCCAGTACCAGCGCAGCCGTTTTTCCATTTC
AGGCGCATAACGCTGAACCCAGCGGTAAATCGTGGAGTGATCGACATTCACTCCGCGTTCAGCCAGCATCTCCTGCAGCTCAC
GGTAACTGATGCCGTATTTGCAGTACCAGCGTACGGCCCACAGAATGATGTCACGCTGAAAATGCCGGCCTTTGAATGGGTTC
ATGTGCAGCTCCATCAGCAAAAGGGGATGATAAGTTTATCACCACCGACTATTTGCAACAGTGCCTTATTTCGAGCATTTTCTT
GCGAAAAAGCCGACCGGGAAAATTGACATGCGTACCATTCGCCTGGGGTGTAATAAGGCGATAGACACCGGTACCTACACAT
TTACCTTCGCGGACAAATCAACGGTTTCAGCCCGCTACACATTCACTTATGCATGGGACGGTAAAGAGTGGAAAATTTCCACA
CACCACTCTTCAGCGATGCCTGAAGGGTAAAAAGCTGTGCAGGGGGGCGGAATATTCACCGCTCCCTGAAGACTAGCCAATAA
TCGCGATGCCAAGACGTTCCATGAGCAACGATGCCTGGTAGTTGTCCAACTTAACGCCTTGTAAATCAACGCGCCGAATATCTA
AGTCACCCAACTCCGAATTGGTCAGATCGCAATGTGTGAAGTTTGCTGCTCGCCAGTCGAAAGTCGAAAACTCGCCGCCGGA
GAGATCTGAACCACTGAACGTCGCGCCCAGTACCTGGGCCCCCATCCAACGGTTTTCCCACAGCTCACACTTTTCCAACACGA
CTTTCGAAAAATTGGCGTAGCTTAGATTTGTGTTAGTGATATATGCACTGCAAAACCAGGTGCGAGTAGTGATCATATTCATAA
AACTTGCGCCGCGAAAATCTGCGCCTTGCGCACGACAGTGGCGAATTTCAATGCCAAGCGCACTGGCATTGCGAAAATCCGC
CATGGATAAATCACAGCTTTTAAAAATGGCATCTTTCAGCATCGCACGACTAAAATTGCACCCTTTCTGGCTTTCACGATCATAG
AACTGACAGCCGATAAATTCAGTGCCGCTCAGGTCGGCACCTGAAAAATCACAGTTAAAAAATGTACTATTTTCAATTTTCTCA
CCGGTGAAGCGGTTTCTGTTAATTTTTTCGCCAACGAGTGCCAGAGCCATATTTGTGCCTGTTTTTTTATACAGTAATGGCGT
CATGGTAAACCCTGATGAGGTTATGCGTCAAATCCGCCAATATAACATCTGCAAATGTGCGTTAAATCTGGTGTTTTTTCAGCAA
AGCGCGAAGCTGATGGTAAGTCAGACCCAGTAATTCAGCGGCTTTTTTCTGGTTAAATTTTGCCTGCTGTAAGCTGGTTTGTA
GAAAGTCTTTCTCTTGCTGCTGCTGGAATTCACGCAGATCCAGCGGTAACCCTACAGACATCGGTTTAGTTTCCGGCGCCTGC
GGCTGCGTCTGGTTCTGAAATCCATCCCTGTCGGTGTTGCTTATGCAGTCTGGTCGGGACTCGGCGTCGTCATAATTACAGCCA
TTGCCTGGTTGCTTCATGGGCAAAAGCTTGATGCGTGGGGCTTTGTAGGTATGGGGCTCATAATTGCTGCCTTTTTGCTCGCC
CGATCCCCATCGTGGAAGTCGCTGCGGAGGCCGACGCCATGGTGACGGTGTTCGGCATTCTGAATCTCACCGAGGACTCCTT
CTTCGATGAGAGCCGGCGGCTAGACCCCGCCGGCGCTGTCACCGCGGCGATCGAAATGCTGCGAGTCGGATCAGACGTCGT

GGATGTCGGACCGGCCGCCAGCCATCCGGACGCGAGGCCTGTATCGCCGGCCGATGAGATCAGACGTATTGCGCCGCTCTTA
GACGCCCTGTCCGATCAGATGCACCGTGTTTCAATCGACAGCTTCCAACCGGAAACCCAGCGCTATGCGCTCAAGCGCGGCG
TGGGCTACCTGAACGATATCCAAGGATTTCCTGACCCTGCGCTCTATCCCGATATTGCTGAGGCGGACTGCAGGCTGGTGGTT
ATGCACTCAGCGCAGCGGGATGGCATCGCCACCCGCACCGGTCACCTTCGACCCGAAGACGCGCTCGACGAGATTGTGCGG
TTCTTCGAGGCGCGGGTTTCCGCCTTGCGACGGAGCGGGGTCGCTGCCGACCGGCTCATCCTCGATCCGGGGATGGGATTT
TTCTTGAGCCCCGCACCGGAAACATCGCTGCACGTGCTGTCGAACCTTCAAAAGCTGAAGTCGGCGTTGGGGCTTCCGCTAT
TGGTCTCGGTGTCGCGGAAATCCTTCTTGGGCGCCACCGTTGGCCTTCCTGTAAAGGATCTGGGTCCAGCGAGCCTTGCGGC
GGAACTTCACGCGATCGGCAATGGCGCTGACTACGTCCGCACCCACGCGCCTGGAGATCTGCGAAGCGCAATCACCTTCTCG
GAAACCCTCGCGAAATTTCGCAGTCGCGACGCCAGAGACCGAGGGTTAGATCATGCCTAGCATTCACCTTCCGGCCGCCCGC
TAAATATCTCCTTTTGGGTTGTTAATAAAACATCCAATAAGTTGACTGTGCGTGAAAAAGAAAGTTTTGTGTGATGGCGTTGAA
GATCGCACCGTTAAGCTCTTATGTGGGATGGTGCAGAGCTCGACGACTACCGATAAAACGCAACCGCCGCAAACAGACAAGA
AAAAGCCCCAACTGATAACAGTTGGGGCTTCAGTATTGTGATTGGTGGAGCAATAGCACCCTGAACCCAAAACCTTCTCGCTC
AACCGGTAGTGGCTGATAACAACTCGTGAGGGCTATTGCGGGTTAAGCATTTAGCGATGTCTAGGGCCAGACTGGACGTCTG
AACGCAAGCCGCTGATACTGTACATAACCACAGTATCAGCGGAGGATACCCATGTCGCTGGCAAGGAACGCCACGGCGAGTC
AATCGCCCACTCAAACAAACGGTTACGAACGCCACCAACCCGACCAGACGCTGCTCTACCAGCTGGTTGAGCAGCACTACCC
AGCCTTCAAAGCCTCACTCGAAGCCCAAGGTCAACACCTGCCTCGCTACATCCAACAAGAATTCAACGACCTCCTCCAATGTG
GCCGTCTGGAGTATGGTTTCATGCGGGTTCGCTGCGAGGATTGTCATCACGAGCGTCTGGTCGCCTTCAGCTGTAAACGACG
CGGCTTTTGCCCTAGCTGCGGTGCCCGCCGGATGGCCGAGAGTGCGGCGCTGCTGATAGACGAAGTCTTCCCCAAGGAGCC
CATTCGCCAGTGGGTGCTCAGCTTTCCTTTCCAGCTACGCTTTTTGCTGGCTCGCCATCCCCAGCTGATGGGCCAGGTCTTGA
GTATCGTCTATCGTACACTCTCAACTCATCTGATCAAAAAAGCCGGTTACACCAAAGCCTCTGCACAAACTGGCTCAGTGACTC
TTATCCAACGCTTTGGCTCCGCGCTAAATCTCAATGTCCACTACCACATGCTGTTTCTCGATGGTGTCTATGCCGAAGATGACTA
TGGCAAGCAACGCTTCCATCGTGTCAAGGCACCCACTTACGATGAGCTGAATACGCTCGCTCACACCCTCAGCCATCGCATCG
CTCGCTGCATGGAAAAGCGTGGGATTTTGGAGCGTGATGCCGAGAATACGTGGTTGACACTGGAAGAGGGCGAAGACGATA
CGCTGACTCAATTACATGGTGCTTCGGTTACGTATCGCATTGCCGTCGGCCCCCAGCAAGGGCGCAAAGTCTTCACCCTGCAA
ACCTTGCCAGGGCGTGAGGATAAAGCCGACTCAAGCAGTCGAGTAGCCAACCATGCTGGTTTCTCGCTACACGCCGGTGTGA
TGGCCGAAGCGCATCAGCGGGATAAGCTTGAGCGCTTGTGTCGCTACATTAGTCGGCCAGCGGTTTCAGAAAAACGTCTGGC
ATTAACCGCCAATGGGCAGGTGCGTTACGAGCTCAAAACTCCGTACCGCAATGGCACCACCCATGTGATCTTCGAGCCGCTG
GACTTCATCGCCAAACTCGCTGCGTTGGTACCTAAGCCGCGAGTCAACCTCACACGCTTCCACGGCGTCTTTGCACCGAACA
GCAAACACCGAGTTCAAGTAACACCCGCCAAGCGGGGCAAGAAGCCCGACAAATCGGAAGGTCTCGATACTAACTGGCGTG
ACAAGAGTCCTGCAGAGCGCCACCGCGCCATGACCTGGATGCAACGCCTCAAGCGAGTCTTCAATATTGATATTGAAGTCTGC
GAACACTGCGGCGGTCACGTCAAAGTGATTGCCAGCATCGAAGATCCGAAGGTCATTGAGCAGATTCTCAAGCATCTGAAAC
AGAAAACAGCCAAGGCGAATGCCGCCAAGCAGCGTGAGCTGCCACCAGAACGAGCGCCGCCACTGACTCCCAGCCTGTTC
GATCCATCACAGAGTCGTCTCTTTGACTGACGACCCCAAATCCAACACTGCTCAACACTGCCAACTTTTAAACGGGGCGGTGG
GGCAGTTTGTATCTCTCGAGCTATCAGGCTAGAGATTTTACCGCCAAATCGAACCTTATTAGAGCGGTTTAGGCTGGACCGGC
AGTTAAAATTGGGGCTTGAGCGGTAAACGAGTGAGGGAATTTCAGGTAAGATACTTCGGATGAGGAGCAAAAAGGTGGTTT
ATACTTCCTATACCCAGTAGTGTTCCCCTGTCGGTTTGGTTGGGGTCGATGCCCCACTTTGGGCGCGGCGTTGGGCGCCGCAA
GATGAAAGCTCTGTGTGAAGGCTTGGATATTAAGACCTTGGATGATTTCAATGCCATCACCGAAGAGCAGATCGTTAGCGTCG
ACAAATTCAGCTACAAAACTGCCGCAAAAGTGCTTGCAGGTATGGCTGAACATGCCGAGCTATTCAACAAGATTCAATCAATC
ATCGGGTTCAAACAGCAGGTCACTTCGGGGGATCGTTTTACCGGTGAGAAGATCGTGATCACCGGTTTTCGTGACGCGGCCT
TGGAGAAGCTGATTGAAGCTGAGGGTGGTGAAGTGCAATCATCAGTCTCGTCGAAGACCACCATGGTGATCGCGGCATCCAC
TTCCGGATCTTCCGGCAAATTGAAGAAAGTGCACGATCTCAACAACAGCGGAAAGGCAAATATCAAACTGATTGATCTGGCC
ACCTTCCGTAAGCAATATCTGGAACAACCAGCATCGACTGGTTTGGAGTTTTAATGAGTCACCCACAACTTGAGCTAATAGTC
GCTGTGGATTCTAAGTTGGGATTCGGGAAAGGCGGCAAGATTCCATGGAAATGCAAAGAAGACATGGCGCGATTTACGCGG
ATTTCTAAAGAGATCCGCGTGTGCGTTATGGGGAAACACACGTATACTGACATGCGTGACATGCAGTTAGAAAAGGATGGCG
CCGAGGAGCGAATCAAGGAGAAAGGAATTCTCCCCGAACGCGAATCGTTCGTGATCTCCTCGACGTTAAAACAAGAAGATG
TCATAGGCGCTACTGTCGTTCCTGATCTTCGTGCTGTGATCAACCTGTATGAGAATACCGATCAACGCATTGCTGTCATTGGTGG

GGAGAAGTTGTACATTCAAGCTCTTTCATCAGCAACGAAACTGCACATGACCATAATTCCAAGAGAGTTCGACTGTGATCGAT
TTATTCCTGTTGATCCGATCCAGAACAATTTTCACATTGATTCCAGTGCCAGCGAGACTGTGGAGGCAACCGTTGATGAGACT
CAAGAGCGCATTCACTTTGCTACTTACGTGCGTAACAATCAGTAACGCGCTGGCAGTGGAAGAACAAGAACCAGAAACGAA
GCAGACGGTGTTGGCCGTCTGCTTCGAAACATCATTATTGGCTCGAGAACTGATCGAGAATACCATTCTTGCGGACGACATCC
AAAACAATACAGAAAAGATAGCTTTGCAGTTGTCGGAGCCCCTCCCCCCAACTTATAATGGGATTATTGCTGGGCTAAACAAT
GCTGTCGCCATGCGTGGGACAGCAAACGTTGAATCGCCATCAAGAGCATCCACAGATTTACGTGCAGCCGTCATCGGTTTATG
GTGTATAAATGACACCATAGCCGCCCTGAATCAGACTGATAATGCAGTCTTACAGGACGGCGAGTTGCAACAAACACTTGTCG
ATTATTACAACAACTTATTGGCTGACATCGTTTGTCGTTTTCAGAAGACGGCTGCACTGAACGTCAGAAGCCGACTGCACTATA
GCAGCGGAGGGGTTGGATCCATCAGGCAACGACGGGCTGCTGCCGGCCATCAGCGGACGCAGGGAGGACTTTCCGCAACC
GGCCGTTCGATGCGGCACCGATGGCCTTCGCGCAGGGGTAGTGAATCCGCCAGGATTGACTTGCGCTGCCCTACCTCTCACT
AGTGAGGGGCGGCAGCGCATCAAGCGGTGAGCGCACTCCGGCACCGCCAACTTTCAGCACATGCGTGTAAATCATCGTCGT
AGAGACGTCGGAATGGCCGAGCAGATCCTGCACGGTTCGAATGTCGTAACCGCTGCGGAGCAAGGCCGTCGCGAACGAGT
GGCGGAGGGTGTGCGGTGTGGCGGGCTTCGTGATGCCTGCTTGTTCTACGGCACGTTTGAAGGCGCGCTGAAAGGTCTGGT
CATACATGTGATGGCGACGCACGACACCGCTCCGTGGATCGGTCGAATGCGTGTGCTGCGCAAAAACCCAGAACCACGGCCA
GGAATGCCCGGCGCGCGGATACTTCCGCTCAAGGGCGTCGGGAAGCGCAACGCCGCTGCGGCCCTCGGCCTGGTCCTTCAG
CCACCATGCCCGTGCACGCGACAGCTGCTCGCGCAGGCTGGGTGCCAAGCTCTCGGGTAACATCAAGGCCCGATCCTTGGA
GCCCTTGCCCTCCCGCACGATGATCGTGCCGTGATCGAAATCCAGATCCTTGACCCGCAGTTGCAAACCCTCACTGATCCGCAT
GCCCGTTCCATACAGAAGCTGGGCGAACAAACGATGCTCGCCTTCCAGAAAACCGAGGATGCGAACCACTTCATCCGGGGT
CAGCACCACCGGCAAGCGCCGCGACGGCCGAGGTCTTCCGATCTCCTGAAGCCAGGGCAGATCCGTGCACAGCACCTTGCC
GTAGAAGAACAGCAAGGCCGCCAATGCCTGACGATGCGTGGAGACCGAAACCTTGCGCTCGTTCGCCAGCCAGGACAGAA
ATGCCTCGACTTCGCTGCTGCCCAAGGTTGCCGGGTGACGCACACCGTGGAAACGGATGAAGGCACGAACCCAGTGGACAT
AAGCCTGTTCGGTTCGTAAGCTGTAATGCAAGTAGCGTATGCGCTCACGCAACTGGTCCAGAACCTTGACCGAACGCAGCGG
TGGTAACGGCGCAGTGGCGGTTTTCATGGCTTGTTATGACTGTTTTTTTGTACAGTCTATGCCTCGGGCATCAAGGCGAGCTC
AGAGACCATGGAAAGCATGTTCTCGGACTTACGTAGCAACTCGTTTCTTTTCGCAGGTTGAGCCACCTCCGCGCTTCATCAGA
AAACTGAAGGAACCTCCATTGAATCGAACTAATATTTTTTTTGGTGAATCGCATTCTGACTGGTTGCCTGTCAGAGGCGGAGA
ATCTGGTGATTTTGTTTTTCGACGTGGTGACGGGCATGCCTTCGCGAAAATCGCACCTGCTTCCCGCCGCGGTGAGCTCGCTG
GAGAGCGTGACCGCCTCATTTGGCTCAAAGGTCGAGGTGTGGCTTGCCCCGAGGTGATCAACTGGCAGGAGGAACAGGAG
GGTGCATGCTTGGTGATAACGGCAATTCCGGGAGTACCGGCGGCTGATCTGTCTGGAGCGGATTTGCTCAAAGCGTGGCCGT
CAATGGGGCAGCAACTTGGCGCTGTTCACAGCCTATCGGTTGATCAATGTCCGTTTGAGCGCAGGCTGTCGCGAATGTTCGG
ACGCGCCGTTGATGTGGTGTCCCGCAATGCCGTCAATCCCGACTTCTTACCGGACGAGGACAAGAGTACGCCGCAGCTCGAT
CTTTTGGCTCGTGTCGAACGAGAGCTACCGGTGCGGCTCGACCAAGAGCGCACCGATATGGTTGTTTGCCATGGTGATCCCT
GCATGCCGAACTTCATGGTGGACCCTAAAACTCTTCAATGCACGGGTCTGATCGACCTTGGGCGGCTCGGAACAGCAGATCG
CTATGCCGATTTGGCACTCATGATTGCTAACGCCGAAGAGAACTGGGCAGCGCCAGATGAAGCAGAGCGCGCCTTCGCTGTC
CTATTCAATGTATTGGGGATCGAAGCCCCCGACCGCGAACGCCTTGCCTTCTATCTGCGATTGGACCCTCTGACTTGGGGTTG
ATGTTCATGCCGCCTGTTTTTCCTGCTCATTGGCACGTTTCGCAACCTGTTCTCATTGCGGACACCTTTTCCAGCCTCGTTTGGA
AAGTTTCATTGCCAGACGGGACTCCTGCAATCGTCAAGGGATTGAAACCTATAGAAGACATTGCTGATGAACTGCGCGGGGC
CGACTATCTGGTATGGCGCAATGGGAGGGGAGCAGTCCGGTTGCTCGGTCGTGAGAACAATCTGATGTTGCTCGAATATGCC
GGGGAGCGAATGCTCTCTCACATCGTTGCCGAGCACGGCGACTACCAGGCGACCGAAATTGCAGCGGAACTAATGGCGAAG
CTGTATGCCGCATCTGAGGAACCCCTGCCTTCTGCCCTTCTCCCGATCCGGGATCGCTTTGCAGCTTTGTTTCAGCGGGCGCG
CGATGATCAAAACGCAGGTTGTCAAACTGACTACGTCCACGCGGCGATTATAGCCGATCAAATGATGAGCAATGCCTCGGAAC
TGCGTGGGCTACATGGCGATCTGCATCATGAAAACATCATGTTCTCCAGTCGCGGCTGGCTGGTGATAGATCCCGTCGGTCTG
GTCGGTGAAGTGGGCTTTGGCGCCGCCAATATGTTCTACGATCCGGCTGACAGAGACGACCTTTGTCTCGATCCTAGACGCAT
TGCACAGATGGCGGACGCATTCTCTCGTGCGCTGGACGTCGATCCGCGTCGCCTGCTCGACCAGGCGTACGCTTATGGGTGC
CTTTCCGCAGCTTGGAACGCGGATGGAGAAGAGGAGCAACGCGATCTAGCTATCGCGGCCGCGATCAAGCAGGTGCGACA
GACGTCATACTAGATATCAAGCGACTTCTCCTATCCCCTGGGAACACATCAATCTTACCGGAGAATATCGTTGGCCAAAGCCTT
AGCGTAGGATTTCGCCCTCTCCCGCAAACGACCCCTAAAAGCCGTTTCCTCTGTATAAAAGATCAGCTAAATTATGTGTATTGC

ACAATACATATATGTGAGGTTAGCAGTGAATTTGCCTACGCCCGAAACCTACGATGAACTTCAGAGAGCCTACGATTTTTTCAA
TGAGAAGCTATTCAGCAACGAGCTGCCGCCATGCCTGATAACGTTGCAGCGTGAGAAGCGAACGTATGGCTATTGTTCCTTTA
AGCGTTTCGTCGGCCGTGAGAGTGGGTACACGGTAGACGAGATCGCTATGAATCCGGTGTATTTCTCGATCAGAACCATAAAG
GCCACGCTTTCAACACTGGTGCATGAGATGGTTCATCAGTGGCAATTCCATTTTGGCGAGCCTGGCCGCCGTGGCTATCACAA
CAAACAGTGGGCGGCCCGGATGGAACGGGTAGGACTAATGCCTTCTGATACCGGCGAACCGGGAGGCAGGAAAGTGGGCC
AGAGCATGACCCATTATATTATTGCCGGTGGCCCTTTCGATATGGCCTGTGATGAACTGCTGACAGGCCATTTCCGGCTTTCCT
GGATGGACAGGTTTCCGCCTTACCAGCCTAAGCCTGGCGCTGTGCTAAGCCCTACAGGAAAAGGCTATATTGACGACGAGGA
AGATGATAGCGAACACGAACAGGAGGTGGAGGAAGGGCGCGACCCGGTTGAACTCGACGACGAGATCATAGAGGCCATGC
GATTTGTAACCCCACCGCCTGAAGCACCGGTGAACAAAACAAACCGGGAAAAGTACAGCTGCCCGGTGTGTCATATCAATCT
CTGGGGTAAACCGGGGATAGTGGTTTACTGTGGTGGCGAGCACTGTAATAAAGCCGCGTTAGTAGTCTTAAAATAAAGTCCTT
TCGGACTTTATTTTTTTTCCATTTCCGAGGTCGTGATGTTATTAATGCTGTACTTCGCGGCTTCTTTTAAAACAGTTTCAGCAAG
GCTTGCTGGTATCCAGACCTGAACTAATTTTAATGGTTCGCCGTTCTCGGCTTTAAGAGTGGTGTTCTGGTACAAATCCCAGAT
TCGCTTAACGGTGCTGGAAATGTTTTGCTTGGAACGGCCTACTCGCATGGCTACGTCTGATGATTTCTCACCTTTGACAAGCAC
GGAATAGCCAATATCTGTTGTGATGTGTGCAAAGGAAGCCATTTGCGGCAGCAGCTGTTTCCATTCTGTTTCTGAAATTCTGTT
TTTCTGAGCCATCTGTGGCGCCTCCGTAGTTTTGGTTACAGAAAGGATATACTCAGAATAAACAGGGGTCAATACAAGTACGA
TTTTTATAAACTTTATTTTATTTGAGGGTGAGGCCCGGTGCGGCAGCAGCGCGGGCCTCGATGGTGCCGCGAAGGTGCTGGC
GCCATGCTCGGATTAAAACATGAACCGTGAAGAACTGCGAAACTTGTTTTCGCGGTTCTGAGGGGTTGACCGAGCCGCGAA
GCGGCGCTGGTAAGCGATGATATGCACATATCCACAGGCATATTTTTAAAAGGGGCACTGTTGCAAAGTTAGCGATGAGGCA
GCCTTTTGTCTTATTCAAAGGCCTTACATTTCAAAAACTCTGCTTACCAGGCGCATTTCGCCCAGGGGATCACCATAATAAAAT
GCTGAGGCCTGGCCTTTGCGTAGTGCACGCATCACCTCAATACCTTTGATGGTAGCGTAAGCCGTCTTCATGGATTTAAATCCC
AGCGTGGCGCCGATTATCCGTTTCAGTTTGCCATGATCGCATTCAATCACGTTGTTCCGGTACTTAATCTGTCGGTGTTCAACGT
CAGACGGGCACCGGCCTTCGCGTTTGAGCAGAGCAAGCGCGCGACCATAGGCGGGCGCTTTATCCGTGTTGATGAATCGCG
GGATCTGCCACTTCTTCACGTTGTTGAGGATTTTACCCAGAAACCGGTATGCAGCTTTGCTGTTACGACGGGAGGAGAGATA
AAAATCGACAGTGCGGCCCCGGCTGTCGACGGCCCGGTACAGATACGCCCAGCGGCCATTGACCTTCACGTAGGTTTCATCC
ATGTGCCACGGGCAAAGATCGGAAGGGTTACGCCAGTACCAGCGCAGCCGTTTTTCCATTTCAGGCGCATAACGCTGAACCC
AGCGGTAAATCGTGGAGTGATCGACATTCACTCCGCGTTCAGCCAGCATCTCCTGCAGCTCACGGTAACTGATGCCGTATTTG
CAGTACCAGCGTACGGCCCACAGAATGATGTCACGCTGAAAATGCCGGCCTTTGAATGGGTTCATGTGCAGCTCCATCAGCA
AAAGGGGATGATAAGTTTATCACCACCGACTATTTGCAACAGTGCCGGTCGCCGGGAGTCAGCAGATCGACGTCAACGCCGA
GCAGCGATTTCAGTTCTTCTTCCAAATCGCCCAAGTCCAACAACGTGGCACCGGGCAGCGCATCGACCAACAGGTCGAGGTC
GCTGCCATCCCGGTCGGTGCCATGCAGCACCGAGCCGAAGACGCGCGGGTTCGCGGCGCGAAAGCGGCCTACCGCTTCAC
GCACTGCGCTTCGCTTCATGTCAAGCACAACAGACGGTCGCATGCGCATCCTTTCTTATCGAAACTCGTTGAGATGATATGCAA
TCAAGAATAGAATTTCAAGAACTCACAAAGTAACGCGGTGGTTAATATCCTGTACCCACGGATTGCCCTTAGCGCTGCCTATAT
CGGCTAAAGCACTCCGGTAGCTTGATTCACCCCACGGCCACGGCAGGATCTTGGCCGTCGCAAGCGCCAGGGGAAAATCTT
CAGCTGCAAGCCTGAGTGATTTCATGTGCGTGTAATCCATCGCCCAGATGATTTTTGTGAAGAAGAACTCGCGCTCGTTCATG
TCCGGGCGCGCGTCCTGGCCCTCCGTGCCAACGGCCAGCAAGTAATCGGCCTGAATTGGCAGTATCAGCGCGCGTAGTAAGT
CATGGATCGCCGGTTGCGGCAGTCTGCGCGCCAGATTAATTACCCGGTCGAACTTCAGTTTATCCTGCTTGCGCTTGTCGGTCT
GCTCCATCAGATTTCATGGCCCCCTTCTTCATGCTCATGCTCATGGGTGTGTTCTTTTCCGGTATGGCTCTGTTCCGCCTGAGAC
GTCTGCGGCATGGCGTAATCGTCGTAAATGCTGCTGTCAAAGTCGTAGTCTGATGCTTCGGCATAATGCTCGTAATCGGCATAC
TCCTGCGCGCTCCACTGCTGATCGTCGGCAGCAGCATAATCATGGGCCAGCTCTGCATCATTTTGCTGTGCTTCATGACGCCGC
AGGCCAACGGAATCATCCATAGGGTTCTGCTTAAGATGAAAGGCGTCCTCTGCGTTGCTCACCGGCTGATAATCAGTGCCGGT
TGTCATGTTATGTTCATCGGGTTTCTGGTTAAACGCCATGCTTTCCCCCGTGGCTTCTGGCAGACCTTTTTCAGCTGATCGGGT
TTCTAAACTGGTATCGCGGCCAATATCCTTAAACCTGGCCTCAAGCCCAAAGAAACGGTCAATTTCTGCGGCCGTGGTTTTCG
GGCTGTCGCGGCTCACGCTCGATGCCAAAGATTTTTTATCGTCGGTAAAAATTTCCACCTCATGACGCGCACGCGAAATACCA
ACATAAAAAACGTCCTTAGAAGTGGTAAGCGATTTGGTATCTATGTTGAACAACACGCGATCACAGGTAAGCCCTTGGGATTT
GTGGACGGTGGTTGCATAAGCATAGGAAAGATAAGAAGCCTGTTTTTTGTCCAGCTCAACCGTGCGCCCTTTTTTGTCCTCAA
GCGTCAGTTTTTCACCCTCCACGGTTTTCACCGTGAAGCGGTCGCCGTTGGCAACGTCCAGCGTTTTATCGTTACGCGTTACC

ATAACCTTATCGCCCGGCGCCAGTTCGGCGCTGACTGCCTGGTATACAGACAGCTTGGTGTGTGTACGCGGGCTGAAAGCGA
TCTGCTCACCGCTGCTGCTTTCAACCGTCAATTTGTTGCCCGGCCCGGTATCAAGAACCTGGTAAGACTCGCCCCGCTTCATAC
CATTTTTGTAATCCTGTTCGGGGATAATGATTTGCCCTTTACTGAAATAACGGCTGTCGCGGCGTTCCGCCTGTGTCGAATCCA
CGCGGTCAAGTAGCGTGAACGTTTCGCCGGTTCCGGCAAGCCCCAGATTGCCCCGGATGTAGTCATTGAGGGTTTTGCGTGA
GGCGTTCGTACCAGAGATTATCAGGGTGGCATCCTGTTGTTCTGAGGACAGAGACAGGTAGCGATCGGCAAGTTGAGCGAG
TCGGGGCGCTTCTTCCTTCAGTTCGTTCACGCCGGTGATATTTTTCAGGGCGCGCGCGGCATTACCTTCAGCGGCATACTTAA
CCGCCTCAAGCAAAACTTCATTCTTCTGTCGCTGAATGTCTTTCATGTAGCTGGTCTGCATATCTGCTTTAATCAGCTGCTCAAA
AGGCTTACCGGCTTCTACCGCTTTCGTCTGTGACGTATCCCCCAGGAATACCGCGCGAGCGTTATGCTTCTCGATCACCTCCAT
CAGCTGTTTCATCTGTCGGGCGGGTATAACCCCGGCTTCATCAATGAATACGACTGATTTTTCATCCAGCTTTTTATCCTTCGCT
TTGAGGAAAGCGGCAACGGTGCGGGCCGGTAATCCATCATCTTCAAGCGCTTTTTTCTGTGTCCCATAGGGGGCCAGCGCCG
TGACCTTCAGCCCTTGTGACTCCAGCAGCTCTTTAGCGGCCATCGTCATATAGCTTTTACCGGTACCGGCGTAACCATGTGCGG
CCACAAACCGATCTTTGCTCGTCACAATTTCTGTAACCGCGCGCATCTGCTCCTTCTTGAGGGTTTTCCCGGCAAGCAGCTGG
CCTGCAATCTCTGCGGTCAGCTGTCGCGGCATCTGCCCCCGGCCGCGTGATTCGATAGTCAGAATGGAACGCTCAAGGCGAA
TACCCTCCACGGTAGTGACGCGGTGGCTGGTCTTTTTAAGCCTGCCGTTTTTAATACCATCATCTACCGCAAAACGGGCTTTAT
CCGCACGCATCCCGCTATTCGTCAGCGAGTCGATCCACTCTTTGCGCGTCAGAGTTTCGGCCATAACTGAAGCACCGACCTTC
AGAGTTGATTGATACCGGGCTTCGCCCTCGATGATGGCGCCCTTCTGTACCGCCTTCAGGTACGCTTTTTCAACATCGGCTATT
GTGGCATGGCCCAGCACCTGCTTATTAGCGATTTGAATCAGCTTCTGGCGTTCAAAGCTGGCATCGCGCTCTGACAGCGACTT
AACTGCAAACTGGATAGCCCGGTCAGCTTTAACCTCCGGGCTGGTAAAATCCGGGGCCATGTTGCGCGCTATATCAGCCTCCA
GAGGTTTACCGTGTCCCTGCCATTCACGGTTATCAAAATCAATGCCGAGCGTTTTGGCGCGGCTGGCCCATTCCTGGTGAATT
TCTTCACGGGAATGCTCTGTTTTCTTTTCACGCGTAGCCATCGAGACGCGGCTTTTCGTCTGAGCATCGGCGGTTTCCCGCGT
CAGACCCATTGCAGCGAGTCCCTTTTCAATTTGCTCCGACCGGCGGGAAAAAGCGCGAATCTGTTCATCTGAAAAATGGGCC
ATATCGAACGTGTTATTTTTGCTGTTGTAACGCAGCTCATAACCGGCTTTGGTCAACTCCAACGCCAGCTCCTGTTTGTAAACAT
CGCCCAGGTGCATTTTGTTACGCATCAGCTCATCATTTTTGAGCGCGCGCCACTGGCCGTCCTCGCGCTGGGTCATGTTCATG
ACAAAAGCGTGTGTGTGCAAATCAGGATCTAGCGCCCTGGAAGTTTCGTGGCGGAAAGTAGCGACGACAAGGTTATTGGTAT
TCTGGGTTACTGATTTCCCCTGGCGAGTCGTCCGGGCCTGCGCGAGTTTTTCAGCTTCACGCACAGCAGCGGCAACAGCTTT
TTCATGAGCCTCGATAATGGTTTTATCGCCGTGTATCAGCGCCTGCATGGATACCCCTTTAGGCGCTGAAAACGTCAGGTCGTA
GCCCAGACGCTCTTTTTTGGCATCACCCACGTGTCGCTGCATATGCGTGAAGGTATCTATCTCTCCGACAAGCAGCTCTTTAAA
CCGGGCTGATTCAACGTCCCCGGATAAGCCGAGGGCTTCAGCTCCGGTTCCCTGCCAGGACGTGAATGATGAATCCTTACTGT
AGTAATCATCCTTTGCATCAGAGTAGTAGCCCACAACGCTAGTGACGTTCTGGCGGGTAATCGTGGTTATATCAAGCATCAGAT
CTCCCTCAGTTCAATGCCAGGAACAGGGTTTTTGCGATGGTATTTAACGTGTTTAGCCTTGAACTTAGCGACGGGCATATCACC
AGGCAACGCCAGATAGCCGGTGAGGTTTGGCAACATTGATATTTCGGTAGGCGTTACGGCACGAACAACTTTAACGTCGCGG
CGTTTACGGACAATCCAGGGCTTCTGAGGATCGGATTCTTTACGCTCAACTTCGCCTTCTATCTCACCGAGTGAGCGCGACATT
TGATCCAACGTTTCATCACCGAGACGGCTGCCGCCCAGCACGATGTTAGAACGCATGTTAGCCAGAATTGTCTGAGCCATATC
CCGACCATAAACCTTAACCAGCTGAGAATAGGTTTGATAGCCAGCATAAACACACAGACCGCTTTTACGCCCTTTGGTCAGTG
CATCGTTGAGGTTTGGCAGAAACTGGAGTGATTCCAGCTCGTCAATAAATACATTAATGCGGCTTTCTTTTTCACCCATACCCA
GCACGATAGAAAAAATCGAATCCAGCCAGCAGGAAATTAGCGGATTAAGTGACCTTTTCATTTCTTCCTGCCAGGTGATAAAC
AGGGTTCCCGGCTTTCCATCATCAAGCCAGTCACGCAGGGAAAAATTACCTTCCGGCATTTTCAAATGTGGGGCAAGATTCTT
ACTGAGAACAAATCGCGCGCTTCCAACTGCTTTTTCAGACCCGGAAAAAATAGCTTCGGCAGGCGTCCCCATTAAAAATTCTT
TTAATTTTTTCTGGTCAACGTTACAGGCCCAGTGAATAACTTCTTCCATAGTTACTGTGCTGTATAGGCTGTGAAGTTTTTTCGA
AACTTCACTAAAAATAAGACGGCCATAGCCGAACCATTCTTCAGTAGCCATATCAGGGCTTTCCTGAACAATAGAGTTCACTAA
ACGCTCGTAATCATATGAACGGCGAATTTCATTGAAAAACACCCAGCCTTCAGTGCGTTTATCATAGGCGTTTAAAATAACATC
GCCGGGACGATAGAAATTCTTTAAGAACCCCCCATTTGGATCTAAAGCAATATTTTTGCCGCCTCTAATGATGCTCTTAAATAAC
AGTTCATTGAAAATTGTGGTTTTACCAGTACCGGTTGTACCGGCAATCGAAAAATGCAAGTTCTCAGCGTATGTAGGTATGGG
GATATTAGCCACGGTTAACTGGTTGACACCTCTTTCGCGTGTTTTATCAGCGAGTGTTCTGGCGCGAACAAGCTCTGTACCAC
GATAAATCTTTTTGAATCTTTCGCCTTTAAACACGCGTGATTTATCATAAATGATAAAAGCGATCAGACCGCCAACACCAATAAA
CCAGCCAGCAATTAAAGCTGACCATAAAGGCCATAGCGAAAAAGTATTCTTAACCAGATACGGAATCAGGTATTTAGCCGTGG

ATGGATCAATACCGTAGGTAAATTTTGCAACTAGAAACCATACCATCACTGGAGGCAAAGTAATTGCAAATAAAAATGCTAAGC
CTCTTTCTCTATCGTCCATTTCAGCGCTCCTTTTTTGGTTCCCAGACTTTGTAGCCGTTACGTTCAACCTCTGCTTTTGCCGCTTT
GGTTTTGCCCGGTTCTGCTATCGAGCGCAGGAGGATTAGCGTTTCAATAGCGAGTGATTCATGCAACATCATCTGTCCTGCCG
GTGGGAATTTTACGCCAGATAGCGTTTCGGTTATTGCCTTCAGCTCATCGCGCAGTGGGCCAAAATCCGCATCTGAAGCACGG
TCAAAAAGATAATCCAGTTTGCGATTTACGTCGCTCAGCCGGTCGGCAACTATTTTCAACCCGGACTCCCGATCACCTGGGCC
AGCTTCAATGCAGCGCCGCAGATAATCTGACCGATTACCTCCTGAAACCAGGTCTATATAGGCCAAAAGTTCATCTGATACTTT
TGCGGTTATTATTGGCATTCAGTCCTCACATTGTGCATTTCTTAAACAAAAAATTGGGATCTAACAAGCTGAAATCTTAGTATTA
CCAAAGTAATAAAGCAAACTCATTATAAAACAATGGGTTATTGGGTGTTTTTAATACCTAATTATTACCGAATATTGACGCTATTT
ATTTTTTTATCTTTTAAATCAGTACGATAGCGTGATTTATCGCGCTGCGTTAGGTGTATAGCAGGTTAAGGAAAAAAAATCATCT
TTTTTGGTAGGAGCGACCTCCGTAGGTTAAGGGTCATTTGGCTAAAAAGCGTCCTATTCTTTGATGGTCATGCTTGCATGACCA
TCTGAGCAACCAAAAACTACAGATAAACTACAGAGAAACTACAGATAAACTACAAAAAACGATTTACCTTAGCGTTGTCAGAC
TACTAATAGACTACAAGGAAACTACAAAGAAACTACAAAGAAACTACAAAGAAACTACAAATAGACTACTAAAACCGTGGCA
GACTACTAATAGACTACAAGAAAACTACAAATAAACTACAAAACTGGATTGACCCCTTCTTACGAGTGTTGTAGAGTCATCTTC
ATACAACGGAGGGGGTTATGAATAAACCAGCAGATCTGAAACCCCGCAACTTATCGGCTGCTGTCAGATTGCGCCTAAATGAA
ATCGAGAACTGGCTGGACAGAGGGCTAACGCGGCATGAAATTGCTGAAATCCTCGACAGCGAATACAGCTTTTCGGTAACAG
CCAAAGGGCTTGAGATGGCACTGTATAGAACGCGGCAAAACCGAAAAAATGTATTGCACAATACACATGATAAGAGTAGCGC
GAAGGGTGCAGCGGAAAGTGTATTGCACAATACACAACCGTCTGAGCCTGAAGCGCAGGAAAGTGAAAAAGCAGAGAGTC
CCGGCATTATTGATAAAGAGTTCTTCAATAAAATCGGTGAGGATTTCAACCCTAAGAAGTTCAACAAAAAATTCTGAGGTGAT
TTATGAAAGTAGCGGTAATTAATTACAGTGGCAGTGTTGGTAAAACATTAATTTCATCTTACCTGTTAGCCCCGCGCCTGACTG
GTGCAAAGTTCTATGCGGTAGAGACTATCAACCAGTCTGCTTCCGATCTGGGTATTGAAAATGTGACCAGTTTTAAAGGTGAC
GACTTCTCACGTTTGATTGAGGATATTGTTTTTGAAGATGCAGGCATTATTGATATTGGCGCGTCAAACGTTGAAGCGTTCCTG
ATGGCTATGTCTCGCTTTGACAGTGGCGCGAACGAATTTGATAAATATGTAATCCCGGTGACGCCGGATAATAAGGCGATTGAT
GAAAGCCTGAAAACGGCACACACGTTAAGTAAAGCGGGCGTGAGCAGCAAGAAAATTATCTTTGTTCCAAACCGTATTAGTC
CAGACAGTGAAGTAGAAGATGTGCTGGCGCCGGTGTTTGAGTTTGTCAAAGAAACGAAGATTGGCAAAATAAGCAAGAAG
GCTGTTATTTATAACAGTGAGGTTTTCGAATATCTGGCGTTTCACCGTATCTCATTCGAAGTATTGACCGCTGAAGATCCAGAA
GAATTCAAATCCCGTGCAAAACAAACAACCGATGCTGACGAGCGCAAAAAACTGGCACGCCGTTATACATACATGAAACAGG
CGATTCCGGTAAAAGCTAATCTCGATAAAGCATATGCGGCTTTAATGGGAGAATAAAATGGAAAAGCAGCCGGATAAATTAGA
AGTTCTGATGGACTGGTTTTTAGGTGACGCGAAGGAAATCACCGCAACTCAGAAAGAAATGACGCAGAAACTTTCTGAGCTT
TCGGAAAAGCTGGCAAAAGACACCGAAAGTTTAGGAGAGACGGCAGACTCTTTTAAACGGGCTTTAGTAGAAAACCAGCG
TTCAATTAGCCTGGCAATTAGTGATGATGCTAAGGCGCGCGAGGAATTTCTAACTAAATTCCGCCGCGCGCAGGCGTCCAGTG
CTGAGACGTTTACCCGTCAGATCCTTTTTATTACAGCTGGCTGCACCATCGTGGGCGCCGCAGTAGGCGCCGCGATAGCGATA
CTTTTACTGAGATAAAGCAAACCGGGCGTGTCCCGGTTTTTTTGTCAAGCGGAGCGCGGAGGCCGAAGGCCGGAGGCATTA
GTGGCCGCCGCCCGCGTAAGCGGGGCGAGACGGGAACCGGCTCGAAGCGCAGCACGGCAGAACGGCCCCGCAGGGGCA
ATGCCCGTTTTAATTCATCGTGACAGTCGCGCGTGACCATCACGGGGAGAAAAATAATGAATGACCGACAGCGAGAACTGGC
CCGTATACGCCAGGCCCGCCGCCGCGCGCGGCTCAAGGAAGAAGGCACAAGCGTGACAGTCACGCTAACAAAACAGGAAG
AAGCAATGTTGCAGGAGCTGTGCCGGGTTCGCCGTCCTGGACGAACGCCTTATTCAACGAACGAATTTTTCCAGCTGCTGCT
TATCCGCAACTGGCAGCAGTGGCAGGAGCAGAAGGCACAGCTGGGAAAATGCCAGGCTTGCGGAAAGCTGAAAGCGGAG
GGGGGGTGCGAGGGTGAACGGAAAGGCGAAACCTTTAACTGCTGGCTTGCCGTCGAAGCCAATGAACTAAATTTGTAGTGT
ATTGTGCAATACACATTTACACAGAAACAAAAACCACCGGCAATTCCTGGAACCGGATACCTACGGCTATTCCTGGGTGAACG
GTACTTTTTGCACCTGGGTGCGCTGAAAAAGCTGAATATGCAGGGTGACGTTCGCGTGCTGTTCTGCTTTGTAGACTGAATGC
GCCAGCTATACGCCTGACTGCTTAAACCTGGTAAAGTTCTGCAACCGGCACTGACCGGAAAGCAAGGCAGGGAAGACCTAA
GCCAGAAACCTTGACTGCTCCCCGCCCTTCAGGGCGGGGATTGCGGATCATGTTCTTCTCTTTCAGGGATTCAACGCAGACA
AGAAAGGCTTTCAATTTCCTATACGTGAACGGCCGCGCAGCGGAAAGAAACAAGCCCGGTCAATCCGGGCTTGTTTCTTTAG
GCGGCTCAGAAATCGCCTAAAGGCCCGGCTTGGCCGGGCAGTCAGTGCTATTTAGTTTGTTGCAGCAGCTGGCTTAATTTTG
CCGCCAGTGCATACGTCGATTGGAAAGCGCCTTGCAGGCGCTGATTAGGCAGCTGGTTGAACGCTTCGAGGCAGGCGCGCA
GCAATAATTCTTTCTGAGATTCGACTTCTTTTTTCAGTTGGGAAGGGGTGGTAACTGTAGTCATGCTTGCTCCTTAGTGAGCCG

ATATCGGCAATTTTTCGGGTGGCGGTGTTGCCTCCCGATGATTTAATTATCGGTGATTATGCTTTTAAAGTCAATACAGGTACGG
AATTTATTTACCTGTTTTTATGCCCGTCAGGGCATGGAAGGCGACCGCGCCGGACTCCACCGGACACCGGCCGCAAATCGCC
GGAAACTGCGGGACTGACCGGAGCAACAGGCCAACCCCCCTCCCTGCTAAGCCATAACCCAGCCCGCCGCCACGCAGCTGC
CGCACGTCCCCCACGGGGGTGCGCAGTGGGCGCCGCGCGCCTGCGCGCGGGTACGGCGGCCCGCCTGCGGGTCGCGGCG
CCGTACTGCGAGTTAGCGGCCGCCGCGCGGCCGGTTACGGGGGACACCGCACAGTCACGGCCAGTGCCCCGCTGAGCTGC
ACAATCCACGGATAACACAATAGCGCACTGGCAAAGGATGCCGACGCCTGAAGGGCGTGGGCACCCCGAAGGGGCGGGGC
GGCCGCTTGCGGCCGGGCGAGTCCGGCGCAGGGTGTGGCCTGCCAAGCGGAGCGCGGAGGCCGAAGGCCGGAGGCGTT
AGCGGCCGCTGCCCGCGTAAGCGGGGCGAGACGGGAACCGGCTCGATGCGCAGCACAGCAGAGCGACCCCGAAGGGGTA
ACGCCCGGTGTGGCATCAGGATTTAGTGCAATGGCAGAACATGAGCTGGAGAGATCACCGGCAAGCAGCAGCAAAGGGGC
GGCACAGCCGCCCCGATGGCTGTTTGCCGATACCGGCGATTAATTAGAGCGGTGTTTAATATCCCCCGCGTTGCGGGGGACTA
GGTTTCAGCAAGTCATGTTAAATACGTGTCCATCATGTAAACTGAAATCCCCAATAAACAGATCCCGCGCATAGGCTACGATGT
CAAAATATCGGGCTACGGATTCCGGAATATCATTCAGTAGACCGCTATCATTCAGGTATTCCTCTGCAAAAGTTTCTTCGTCCTT
AGCTTCGCCCATATAGGCATCTCTAAACAGGTCGAAATCAGTGCTATTAAACAGATCAACAAAGGCCACAAACGCCGCTTCGT
TACCTTCCTCGCGGGCTTGTTTAAAGCCGTTAATAAAATCCCAGTTGATATGGCACTCTGACGCCATACCAGACGGAATACCCT
CCCAATCTTGGAACATAAATTCTGGATCAGCCTCATTTGCGTGTAACTCGCGGCAGCGCTCGTAAAACTCCTCTGAGCTATCAA
AATCGGTCAGATCGAGCCAGGCTCCCGCAATGCTTCCGCAGTTGTATTTATGGTAAGTGCCAACATAAACAGAAGGGGTCGTA
ATATCAGTCATGGTGTACTCCTTAAAGCGCCGATACCGGCAATTTTTCGGGCGGCGGTATTGCCTCCCGATGATTTAATTATCGT
TGATTATGCTTTTAAAGTCAATACAGATACGGAATTTATTTACCTGTTTTTATGCCCGTCAGGGCATGGAAGGCGACCGCGCCG
GACTCCACCGGACACCGGCCGCAAATCGCCGGAAACTGCGGGACTGACCGGAGCAACAGGCCAACCCCCCTCCCTGCTAAG
CCATAACCCAGCCCGCCGCCACGCAGCTGCCGCACGTCCCCCACGGGGGTGCGCAGTGGGCGCCGCGCGCCTGCGCGCGG
GTACGGCGGCCCGCCTGCGGGTCGCGGCGCCGTACTGCGAGTTAGCGGCCGCCGCGCGGCCGGTTACGGGGGACACCGCA
CCGTCACGGCCAGCGCCCCGCTGAGCTGCACAATCCACGGATAACACAATAGCGCACTGGCAAAGGATGCCGACGCCTGAA
GGGCGTTGGCACCCCGAAGGGGCGGGGCGGCCGCTTGCGGCCGGGCGAGTCCGGCGCAGGGTGTGGCCTGCCAAGCGG
AGCGCGGAGGCCGAAGGCCGGAGGCGTTAGCGGCCGCTGCCCGCGTAAGCGGGGCGAGACGGGAACCGGCTCGATGCGC
AGCACAGCAGAGCGGCCCCGAAGGGGTAACGCCCTGTGTGGCATCAGGATTTAGCACAATGTCAGAACATAAACTGGAGAG
ATCACCGGCAAGCAGCAGCAAAGGGGCGGCACAGCCGCCCCGATGGCTGTTACTTGTCTTTGTCGCGTAGCACTTTGATTAG
GCCGGTTACGGCCGTAATCAGAGCGGCCAGCGAGGTGATGATTTGCGGTAGGTTTTCGAGGATGGTAGAGGTCATATAGCAC
CTGTAGAGAAGTTGGCGGGGTGTCGTTTCCGACGGCCGCACTGTAACCGGGCGAATAAGGCAGGTTGTCAACAGCTTGAGC
GAAGCGTCTGTTGACAACCTGCCGCGCCCGGTTTCACTGCGGTCATAGGCGGAACGACCCACGCCAACGGAACGGCTTTAT
GACCGGGCAGCTGAGATACCGGCGAACCTGGCTGGCGGCTGACGCCAGCCGCCAAGCGCCAGCGCGGAGGGCAAAGCCC
GGAGGCCAAGCGGAGCGCGGAGGCCGAAGGCCGGAGGCCGGAGGCGTTAGCGGCCGCTGCCCGCGTAAGCGGGGCGAG
ACGGGAACCGGCTCGATGCGCAGCACAGCAGAGCGGCCCCGAAGGGGTAACGCCCGGAGTCTGCCGCTGTTTATCTCTCGT
TCCATCTGAAATCGGCGGTAAGGCCATTAAAAGGGTCAGTTTATCAGGGAGGCGTTAGCCCCCCATGTTGTTAATCATCAGGC
AATATCGTCTTTGTAGCAGGCATAACCGAAGCTAAGCTCTGTTTTCATATAGTGGCGGGCAAAGTCCCAGGCATCGTGGCCGA
AGTCCTCATAATCTGCCAGGACGATTTCGCGGGCTTTGTGCCATTGCTGTACGGAAGAAAGCGGCAGAACAGGGCAGGGGT
GCGACCAGTCAGTGACGGTGTTGCAGATCATATCTGCCAGACGCTCCAGGGAGCCGTAAACCAGCTCTGTGCGCAGATCCGC
CACCATGCGTTGTTTACGCAGTGATGCCAGATAATCAATCTCTTTGGTTATATCAGAATTTAAGCGGGTCTGGTAATCCATGATG
TACTCCTTTGCGCGCCGATACCGGCAATTTTGCGGGCGACGGTGTTGCCTCCCGATGATTTAATTATCGGTGATTATGCCCTCA
AAGTCAATATAAGTACGGAATATGCATGCATAATTTTATATCTTGCAAAGCGTTCATAGAGTGCCTGAATCGCTTTCTGACAGCC
TCAATAAAAAAAGGCGGGGATTCCCGCCTTTTTTCTTACAGCTGCTTACGTGGCTTTTTACGCGTCATATACAACGGTATCGCG
CAGTCTACCGCGTACAAAAAGCACGCCAGCGCGCCGCAACCGTACAGAAACGCAAGCGGCTTATTATCGAAGTAGCTGAAAA
CCCCTGTCGCCGCACACAGGCCCAGAACAGAGACGCAGGCGCAGGTGATCTGCACCAGATCCCTGTATCCCGCACGAACGAT
AAACCAGGGCAGGGCAAGCGCAGCGGCGCTAATAATTAATGCGAGAGGGACAAAAACGAGATAGTGATACATGTGAACTCC
TTGATGGTTGCCGATACCGGCGATTGTTCGGGCGGCGGTATTGCCACCCGATGATTTAATTAGAGGTTTTGCGCGTCCAGGAG
ATTGACCTGAGCCGGGGTAACGTGAAACTTTTCCCCTTTATGGATCACGTTATGCGGGGCGCTAATTTCATCACTGATAAAGCT
AACCGGGTAACGTTTTTTACCGCAAATCCGCTCGCTAAACCATGCCACTTTTGCCGCTGGCCGATCCACTGGATGAACAATCA

CACCGGCCATGCTGCAACCCGTTGCGGGTTCGTCCAGCGTAATGCTTACCGGGACTGTATCCCGGTAAAAAAACTTAACCGGC
GGCACACCTGCCTGCGTAGCGGCTGCGACAACTGCAAGCCCGATAACCGCTATCCGATTAATAAGCATTTATTCCCCTTACTC
ATGCTGATATCACCTTGCCAGCTGTTACCAGTTTACGAAATTCACTTTCATGAATTTCACGCCCATGCTCAAGCGTTGAATACAC
GTTGCCGATAAGCCAGTTACCAGCCGTTTTTGTCTCGGTATACCACCATGCTTCTGTCAGAGTAAGCAGCGGTTCGCTATCCTC
GCCCTTCTCATAGATCCAGATTTTAGTGACGTCTTTACCCGTTTCGTTGCTGCCCTGAATGGTAGGGTCAAAGGTATGTTCAATC
TCTATATCGAAGTAACGCTGAAGGAAGTTAGTAAAGTGCATGACGACTCCTGTAAGCGCCGATACCGGCAATTTTTCGGGTGG
CGGTGTCGCCTCCCGATGATTTAATTATCGTTGATTATGCTTTTAAAGTCAATACAGGTACGGAATTTATTTACCTGTTTTTATGC
CCGTCAGGGCATGGAAGGCGACCGCGCCGGACTCCACCGGACACCGGCCGCAAATCGCCGGAAACTGCGGGACTGACCGG
AGCAACAGGCCAACCCCCCTCCCTGCTAAGCCATAACCCAGCCCGCCGCCACGCAGCTGCCGCACGTCCCCCACGGGGGTG
CGCAGTGGGCGCCGCGCGCCTGCGCGCGGGTACGGCGGCCCGCCTGCGGGTCGCGGCGCCGTACTGCGAGTTAGCGGCCG
CCGCGCGGCCGGTTACGGGGGACACCGCACCGTCACGGCCAGCGCCCCACTGAGCTGCACAATCCACGGATAATGCAGGAG
ACGAATCATGATAGGAGGCTGAAGGGGAAATGAGCGGCAGCAGGGGAAGGGGTTGCCAAGCGGAGCGCGGAGGCCGCA
GGCCGGAGGCGTCAGTGGCAGCTGCCCGCGTGAGCGGGGCGAGACGCGTAGCGGCTCGATGCGCAGCACAGCAGAACGG
CCCCGGAGGGGTGACGTCCGGGGGTTCGCTTTTAAAGATTTTCGACCACATCAGTAAATCGTAGTGACACCATGAAGCAAAA
GTATCGTGCAGACCAGAATCAACAACAGTAACAGCAGACCTTTTTTTCGACGTAATAAAACCGGCCCCAAAGCGCCCGCAAG
AAGCACACTCAACACGATTTCCGTTATACTAATCGTATTCATAATCTCTCACGTTTCCCTTTTTAGAACTCTGCCACACACAGATA
AAACCTTATAACAAGCTACAAAACCCGTTATTCAGACGCGGTAATGCCTAGTTTTTTTGCCAGTATTTCAGATGACAAAGAAAC
CCTTATCCACTTCCCGGTTACAAGGTGAATGATTGTGGCTTCATGCCCTTTAACAGAGTGGGATATTAACTCACGCTGAATGAT
ATGCGTCTCTCCATCATTTCCCGTCACGGCAATTAGGCCCTCTGTAGAACAGGGGTTGATACTACTAATGTTGGTGTTGTTTTCG
GTTTTGTTCAGCATCGCTGATCCTCAAATATCGGTTTGTGTTACGTCTGCCGCTTTGCGCTGGATAAGCGACTTAAAGAAATCC
GACGCCTTCAGAATATCGCTATCCTGGAAGTCGGGAAATGTCGCTTCTGTCAGCTCCTGCGCCGTACCTGCCCGTATAACGGTT
TCATCGCGGATCATCGTTCGCTTAACGCCATCAGCCTGTTTTTTGCTGAAGGAGTGCCGGAAAATAATCGCGTATTGGCCGCC
GCTATCCTGATGAACAGAAGTATCCCAGCGATGATTACCCTGTTTACGATAGTGGTGATAGATTTGCTGGCCGTAGCGCTCCTG
ATCGGAATTACGGTAATCGAACATTAACAGTTCCAAAAGCATTAACCGATCTGGCAATTGCCAGGCGGTAGGGTGTTGAAGTG
TCGCTAACATAGTTTCCCCTGAGCGTGACAGTCACGATAAGGCGGGCTTTGCCCGCCTGGTTATCAGTTAATCAATGGCACGA
TAAATACGATTCTGGCTTTCGTTCTCCAGCGTATTAACGTACTCCCGCAACAGGTGATACCGGTTAGCCATCGTTTCGTTCAGTT
CGGCTTTGCCTTCTTCATATGCCAGGCCGCAAAAGTAACTGTAGGCATACAGACAAACAATAATCCCTACTTCGCGCGCGCTG
CATTCACCTTCAAAATAGTTAGGTAACGAGAGCCAAAGAGGTTGGGGCGCTTCCATAAAAAACGCGCCATTGCTGGCCTGAA
GGTATTCCCAATACCCTCCCTGGTAGTCTTTAGCGTAACGATTCAGAAAGGACTGAATGAAGTGATCTGCGCTGAAGAAAGCG
CCACGAAATGCCGCAGGCATGAAGTTCATGCGGGCGTTTTCAGAAATGTAGCGGGCGGTGATTTCGATAGTTTCCATGATACT
TCCTCTTAAAGCCGATACCGGCGATGGTTAAGCGGCAGGCACATCACCTGCCACTTTTTAATTATCGTACAATGGGGCGTTAAA
GTCAATATAAGTACGGATTATATTTACCTAATTTTATGCCCGTCAGAGCATGGAAGGCGACCTCGCCGGACTCCACCGGACACC
GGGGGCAAATCGCCGGAAACTGCGGGACTGACCGGAGCGACAGGCCACCCCCCCTCCCTGCTAGCCCGCCGCCACGCGGC
CGGTTACAGGGGACACTGAGAAAACAGAAAGCCAACAAACACTATATATAGCGTTCGTTGGCAGCTGAAGCAGCACTACATA
TAGTAGAGTACCTGTAAAACTTGCCAACCTGACCATAACAGCGATACTGTATAAGTAAACAGTGATTTGGAAGATCGCTATGAA
GGTCGATATTTTTGAAAGCTCCGGCGCCAGCCGGGTACACAGCATCCCTTTTTATCTGCAAAGAATTTCTGCGGGGTTCCCCA
GCCCGGCCCAGGGCTATGAAAAGCAGGAGTTAAACCTGCATGAGTATTGTGTTCGTCACCCTTCAGCAACTTACTTCCTACGG
GTTTCTGGCTCGTCAATGGAAGATGGCCGCATCCATGATGGTGACGTACTGGTTGTGGATCGCTCGCTGACGGCCAGCCACG
GCTCAATCGTAGTCGCCTGCATCCATAATGAATTTACCGTGAAGCGGCTACTGCTGAGGCCCAGACCCTGCCTGATGCCGATG
AACAAAGATTTTCCTGTGTACTACATTGACCCGGATAATGAGAGCGTTGAAATCTGGGGAGTGGTTACGCATTCCCTTATCGA
GCATCCGGTATGTTTGCGCTGATTGATGTCAATGGCATGTACGCCAGCTGTGAGCAGGCATTTAGGCCAGATCTGGCAAACCG
AGCAGTGGCCGTTTTATCCAACAATGACGGCAACATTGTGGCCCGTAATTACCTGGCGAAGAAAGCGGGCCTGAAAATGGGC
GATCCGTACTTCAAAGTCAGACCCATAATCGAGCGTCATAACATCGCTATTTTTAGCTCTAATTACACTCTCTATGCCTCCATGTC
GGCCCGGTTCGCGGCCGTAGTTGAGTCCCTTGCAAGCCACGTCGAACAGTATTCAATCGACGAGCTTTTTGTTGACTGCAAA
GGGATAACGGCCGCCATGAGCCTTGACGCTTTCGGGCGCCAACTGCGCGAGGAAGTCAGGCGACACACAACGCTGGTATGC
GGGGTCGGTATTGCCCGTACTAAGACGCTGGCGAAGCTGTGTAACCACGCTGCAAAAACATGGCCCGCTACTGGCGGGGTG

GTTGCTCTGGACGATGGCGCCAGACTGAAGAAATTAATGAGCATCCTGCCGGTTGCGGAAGTCTGGGGCGTCGGCCATCGTA
CAGAGAAAGCACTCGCCACAATGGGGATCAAAACGGTGCTGGATTTAGCCAGGGCAGATACGCGCCTAATCCGTAAAACATT
CGGCGTTGTGCTTGAAAGAACGGTACGGGAGTTGCGCGGCGAGGCTTGCTTCAGCCTGGAAGAAAACCCTCCTGCGAAGC
AGCAGATTGTTGTGTCGCGCTCATTCGGCCAACGCGTAGAAACCCTGACGGACATGCAGCAGGCTGTCACCGGATTTGCAGC
GCGCGCAGCTGAAAAACTGCGTAATGAGAGGCAATACTGCCGCGTCATAAGCGTCTTTATCCGTACCAGTCCTTATTCAGTGC
GTGATACACAGTATGCCAATCAGGCAACCGAAAAACTGACGGTGGCAACCCAGGACAGCCGCACGATAATTCAGGCAGCAC
AAGCCGCGCTGGCGCGGATCTGGCGGGAAGATATTGCGTATGCAAAAGCAGGGGTCATGCTGGCAGATTTTAGCGGGAAG
GAGGCCCAGCTTGATTTATTCGACTCTGCTACGCCTTCAGCTGGCAGCGAGGCTTTAATGGCTGTTCTTGATGGTATAAACCG
GCGTGGAAAGAGCCAGCTTTTTTTTGCAGGCCAGGGCATCGATAACTCCTTTGCCATGCGTCGTCAGATGTTGTCACCTGATT
ACACGACAGACTGGCGCTCAATACCAATAGCCACCATCAAATAATTACCGGCGCCGTACACGGGCCGGTTAACCCCTCAACCG
GCCGAAACAAGTTTCGGCACGGTTTCGCGGTTTTCGGTAAAAGCCGTTTCCTCTGTATAAAAGATCAGCTAAATTATGTGTATT
GCACAATACATATATGTGAGGTTAGCAGTGAATTTGCCTACGCCCGAAACCTACGATGAACTTCAGAGAGCCTACGATTTTTTC
AATGATAAGCTATTCAGCAACGAGCTGCCGCCATGCCTGATAACGTTGCAGCGTGAGAAGCGAACGTATGGCTATTGTTCCTT
TAAGCGTTTCGTCGGCCGTGAGAGTGGGTACACGGTAGACGAGATCGCTATGAATCCGGTGTATTTCTCGATCAGAACCATAA
AGGCCACGCTTTCAACACTGGTGCATGAGATGGTTCATCAGTGGCAATTCCATTTTGGCGAGCCTGGCCGCCGTGGCTATCAC
AACAAACAGTGGGCGGCCCGGATGGAACGGGTAGGACTAATGCCTTCTGATACCGGCGAACCGGGAGGCAGGAAAGTGGG
CCAGAGCATGACCCATTATATTATTGCCGGTGGCCCTTTCGATATGGCCTGTGATGAACTGCTGACAGGCCATTTCCGGCTTTC
CTGGATGGACAGGTTTCCGCCTTACCAGCCTAAGCCTGGCGCTGTGCTAAGCCCTACAGGAAAAGGCTATATTGACGACGAG
GAAGATGATAGCGAACACGAACAGGAGGTGGAGGAAGGGCGCGACCCGGTTGAACTCGACGACGAGATCATAGAGGCCAT
GCGATTTGTAACCCCACCGCCTGAAGCACCGGTGAACAAAACAAACCGGGAAAAGTACAGCTGCCCGGTGTGTCATATCAAT
CTCTGGGGTAAACCGGGGATAGTGGTTTACTGTGGTGGCGAGCACTGTAATAAAGCCGCGTTAGTAGTCTTAAAATAAAGTCC
TTTCGGACTTTATTTTTTTTCCATTTCCGAGGTCGTGATGTTATTAATGCTGTACTTCGCGGCTTCTTTTAAAACAGTTTCAGCA
AGGCTTGCTGGTATCCAGACCTGAACTAATTTTAATGGTTCGCCGTTCTCGGCTTTAAGAGTGGTGTTCTGGTACAAATCCCAG
ATTCGCTTAACGGTGCTGGAAATGTTTTGCTTGGAACGGCCTACTCGCATGGCTACGTCTGATGATTTCTCACCTTTGACAAGC
ACGGAATAGCCAATATCTGTTGTGATGTGTGCAAAGGAAGCCATTTGCGGCAGCAGCTGTTTCCATTCTGTTTCTGAAATTCTG
TTTTTCTGAGCCATCTGTGGCGCCTCCGTAGTTTTGGTTACAGAAAGGATATACTCAGAATAAACAGGGGTCAATACAAGTAC
GATTTTTATAAACTTTATTTTATTTGAGGGTGAGGCCCGGTGCGGCAGCAGCGCGGGCCTCGATGGTGCCGCGAAGGTGCTG
GCGCCATGCTCGGATTAAAACATGAACCGTGAAGAACTGCGAAACTTGTTTTCGCGGTTCTGAGGGGTTGACCGAGCCGCG
AAGCGGCGCTGGTAAGCGATGATATGCACATATCCACAGGCATATTTTTAAAAGGTATTTTATAGATTTTTTATCTTTTTAAAGTC
TTTTAGAGCTATATAACTCATTGATTTAAAATCATAAATAAGTGTTATCTCTGGGAATCCGCCCACCTTGTTATGGGAATTGGCCC
ACCTTACTATGGGAAACAGCCCACCTTACTATGGGAATTAGCCCACCTTGTTATG

>pQEB1_merTn-IS4321

GGAATTGGCCCACCTTAGACGAAACTGTAAAAAATGTATTTACTTGTTTGAACTTTGTGGTAGTGTGGAGAGTAATTTTTAACC
CACAAAGGCAAGGCGCATGGATAAGTTGCTGAACAAAAAGATAAAAGTTAAGCAGTCTAACGAGCTTACCGAAGCTGCTTAC
TACCTCTCGCTAAAAGCAAAGCGCGTTCTCTGGTTATGTCTTATGCAGACGTATTTCACAGCTTCAGTAAGCGAAGATGATGAT
GAGATGGCTGTACTCGGTGACTCTACTTTCAAAGTAAAGGTGGCTGACTATCAGCAAATTTTTCAGGTAAGCCGTAACCAGGC
TATCAAGGATGTTAAAGAAGGCGTGTTTGAGTTAAGCCGTTCTGCGGTAATCTTTTACCCGAAAGAGGGGCGTTTTGACTGC
GTCGCGCGCCCCTGGCTAACAGAGGCTGGCAGCCGATCAGCTCGTGGTATCTGGGAAATCGAATTTAACCATAAACTCCTGC
GGTACATTTACGGCCTGACGAACCAGTTCACCACCTACTCGCTCCGCGATTGTGGCAGTCTTCGAAATCCCCGGACGATCCGC
CTTTATGAAAGTCTTGCTCAATTCAAATCTTCAGGCTTATGGGTTACTACTCATGCTTGGTTAAATGACCGTTTCCTTTTGCCGG
AATCCCAACAGAAGAACTTGGCAGAGTTGAAACGATCTTTCCTTGATCCTGCACTCAAGCAGATAAATGAGAAAACACCTTTA

CTTGCTAAGTATAGTATTGATGATTCAGGAAAATTTCTGTTCTCAATAATTGATAAGCAAAATCCCGTCTGACATAAATCAGCAC
ACATGAGCCTGTCATTTGACAAATTTTTGTCATGAAGATGGGCGAATTTCCACACAGCACCGGCGCCCGGCAAGATGGGCGG
ATTCCACACGACAGCGGCGCCCGGCAAGATGGGCGGATTTCCACACTACAGCGGCGCCCGGCAAGATGGGCGGATTTCCAC
ACGGCAGCGGCGCCCGGCAAGGTGGGCGGATTTCCACACGGCAGCGGCGCCCGGCAAGGTGGGCGGATTCTCACGCGGC
AGCGGCGCCCGGCAAGATGGGCGGATTTCCACACGGCAGCGGCGCCCGGCAAGGTGGGCGGATTCTCACGCGGCAGCGG
CGCCGGCAAGGTGGGCCGATTCCCACGCGGCAGCGGCGCCCGGCAAGGTGGGCCGATTCCCACGCGGCAGCGGCGCCCG
GCAAGGTGGGCCGATTCCCACGCGGCAGCGGCGCCCGGTAAGGTGGGCGGATTTCCACACGGCTGCCGCGCCCGGCAAGG
TGGGCGGATTTCCACACGGCAGCGGCGCCCGGCAAGGTGGGCGGATTCTCACGCGGCAGCGGCGCCCGGCAAGATGGGC
GGATTTCCACACGGCAGCGGCGCCCGGCAAGGTGGGCGGATTCTCACGCGGCAGCGGCGCCCGGCAAGATGGGCGGATTT
CCACACGGCAGCGGCGCCCGGCAAGGTGGGCGGATTCCACACGGCAGCCTCGCCCGGCAAGGTGGGCGGATTCCCACACG
GCAGCCTCGCCCGGCAAGGTGGGCCGATTCCCACGCGGCAGCCTCGCCCGGCAAGGTGGGCGGATTCCCACACGGCACCG
GCGTGCGGCAAGGTGGGCGGATTCCCACACGGCACCGGCGCGGCAAGGTGGGCCGATTCCACACGGCACCGGCGCCC
GGCAAGGTGGGCCGATTCCCACACGGCAGCCGCGCCGGCAAGGTGGGCCGATTCCCACGCGGCAGCCTCGCCCGGCAAGG
TGGGCCGATTCCCACGCGGCAGCCTCGCCCGGCAAGGTGGGCCGATTCCCACACGGCAGCGGCGCCCGGCAAGGTGGGCG
GATTTCCACACGGCAGCGGCGCGGGGCCAGTGGGATTCAGGAGAATAGGTGTTTTACCGAATGCCCTGACGAGGCGTAAAA
AAACCGCTTGGCGGCGGCCTCATAAAGCAGAAAACCCGCTCAAGGCGGGTTATCTGCTCTGTAGCCTGTGATGCTTCGCGGG
CATCCGGCATACAGCGAGGTGAAATTCTTCTTTTGGCATGTTAATTATACGTCTAACGCGGCATATGATCAAACCTGTATTAAAT
AAGCCACTGTACCGTTTATAATGCTCTCAGATCAAAGAGGTAAAGCCCGTTTAGCCGCCTGTGTGATGAGCCAGTTCAGACTC
TTCAAAATCGAATTTGGTACTAAACAGGACCCGAACCGTGGGCAAGCACACGGCAACGGTATAGCCCTCTTCCGGTTTCGCA
CCCGGAAGCCTGGGCGGCAGCGTGGTGAAATTCTTCTTTTGGTTAAGTGAATGGCATAACCGGATGGGCGGATTAGAGGAA
AGGGGATTGCCTAGTAACCTACGCGCCACAGAGATGGAGGTGCGGGGAATGATTGAGCTGATTATCGCTATTCTGACCTTAAT
TGCGGCTGTATTGCAGTTGATCAACTGGTTCCTTTAATGGTGCCGGAGTCTGTGAAGGTGAAAGCCTGAACGGGCAAAACTG
AAAGGTTTATAGCCGTCCTTCGGGGCGGCTTTTTTCGGCAAAATTAGGGTTTTACCGAATAATGCAGAGTTTTAAGGTGAGA
ATTTGCAGACTTGGCGTTTTACCGAACATAGATACTCCCTAGGCTGATAGGTGCATTAGTTATCACCCTACCTGAACATATTGTA
AAAGATGTCAGTCTCCAGTGACTTGTGTACTATCAACTGACAAGACTCTTACACGCAACGCAGGGGGATGGAGTTTTATGCTT
AGAAAAATAATCAGGGGTAGCGGATTCACTCAGTCAGAAGAAAAACTGATAGAGTTCGCTGATGATGCTTTTTTTGGTCTTTG
GTCTTATCCTAATGTTTATAGCGATGAGGGTTACTCTAAAAATAAAATTGGGAAAGAAGTTAGTGACTTATTAGTTATTTTTGAT
AAAGATATAATAATTTTTTCCGATAAAGCTATTACATACAATAAAAACAAAGATCCTAAGGTTGCATGGCAGAGATGGTTTAAA
AAATCAGTCATACAGTCTTGCACACAGTTATTTGGCGCAGAGAAGTTTATAAAAGATCATCCCGAAAGACTTTTTGTTGACAA
AGAATGCTCAGTTAACCTCCCCATTAAAATAGATAATTCTTTTAATTTTCATTTGGTGGCCGTCACTAATAATATTTCAGATCCGG
CGATCTCGTACTTTGACAAAATAGAAAAAGGCAGCTCTGCTACTTTAGTTAACATATTTCCTTTAAACGCCCATCAATGTCTAGA
AAATCCATTTTGTGTCGGAGACGTTTATCCTGATAAGACTTTTGTCCATATACTTGATGAGACTGCCCTAAAACTACTGTTAACC
GAGTTAAACACAGCAACTGATTTCATTGGCTACCTTAACGAAAAAGAGGGTTGTAAGAGAAAGAACATTATTGGTCAGCGCT
GGGGAAGAAGAGACTCTTGCTGCTTACATTATGGGTGATAAAACCATAATATCAAAAGAAATTATTGGAAACGATCAAGGGAT
GACCATACCGGAAGGTGAATGGAAAAACTATAAAACCACTTTCAATTATCAATATCAGCTCTCAATGAAAAAGGGTAGCGTTT
TCTGGGATAACCTAATCCACAACTTCTCGACAAGTATATTGTCAGCTAACGTTGGTTTTTTTAGTGAAATTGAATTTTCTACACA
TGAATTAGGTGTTAGAGAATTAGCCAAAGAAAGTAGGCAATCTAGATATTACCTTTCAAAGAACTTTAAAGAGAAATTAAAAA
CAACTCAGCCTCATCTAAGAACGTCAAGAATGGTCGAATCAATCGATGAGCCTGGAAAGTTTTACTTATTCCTTTTTTTCCTAA
CGATAGCAAGTTGAGTTACTCTGATTACAGAATTCAACGTATATCTTATATAAATGCTTATGCTGAGGTTGCCTTTAATAAATACA
GACATATTAAAAAATTAATTACTATTGCAACAGAGCCGCAAAATACAGAAGGAAGATCTGAAGACCTAATATATAGCATATCCCC
AGAGAAATTTACCAAAGAGCAAAATGAAAAAGCCAAAAGATTATCAAGAGAATACAAAATACTAAGTGATTTTTTACCTACTA
AAACGACAAAGAGCGATAACTTTAAATCAGTTATATCAAAAGGTGAAAAAATAGGGCGGAATACACCTTGTCCATGTGGCTCC
GGTGTTAAATTTAAAAAGTGCCATGGTGCGAATAATTAGCATTATTGTATGTATAACGGTAATGGCGCGGCAGAGAAACCGGC
GCGTTCTGCCCTAGTGTTGGCCTGCGGGTTCCCCGCACCCGCTGTATGTAGTATCGGCAGCATCTGAGAAAACCACTACATGT
AGTTATCAGCGCCACAACGGCGCGGGGACGAGTGCGGTTCGGAAAAATTGGGGTTTTACCGAATCCGGCAAAAGATTGCTT
CCTATAACGTCCGCTTCTGGCACACAGCAGCCGTTAAGATGTAAGGCCTTACGCCAACTAAATCTAATGGGACAGATTTAGTTG

GTGATGGTCAAGTAATCTGCAAACGGTCACCAAGTAAAATGCAAATGGGTAGTCAAGTCCGATGCAATTACGCACCCGGCAA
GGTGGGCCGATTCCCACACGACAGCAGCGCCCGGCAAGGTGGGCGGATTTCCACACGGCAGCGGCGCCCGGCAAGGTGG
GCCTATTCCCACACGGCAGCGGCGCCCGGCAAGGAGGGCCGATTCCCACACAGCACCGGCGCGCGGCAAGGTGGGCCGAT
TCCCACACGACAGCAGCGCCCGGCAAGGTGGGCGGATTTCCACACAGCACCGGCGCCCGGCAAGGTGGGCCGATTCCCAC
ACGGCAGCGGCGCCCGGCAAGGTGGGCCGATTCCCACGGCAGCGGCGCCCGGCAAGATGGGCGGATTTCCACACAGCACC
GGCGCCCGGCAAGGTGGGCCGATTCCCCACGGCAGCGGCGCCCGGCAAGGTGGGCGGATTCCCCACGGCAGCGGCGCCC
GGCAAGATGGGCGGATTCCCACACGGCAGCGGCGCCCGGCAAGATGGGCGGATTTCCACACGGCAGCGGCGCCCGGCAA
GATGGGCGGATTCCCACACGGCAGCGGCGCCGGCAAGATGGGCGGATTTCCACACGGCAGCGGCGCCCGGCAAGGTGGG
CCGATTCCCACACGACAGCGGCGCCCGGCAAGGTGGGCCGATTCCCACACGACAGCGGCGCCCGGCAAGATGGGCGGATT
CCCATATCGACATGTATGTAGCTTGTGTTATCCGTGGATTGTGCAGCTCAGCGGGTCGCTTGTCGTATGGCGTAGTGTCCCCCG
TAACCGGCCGCGTGCGGCCGCTAACGCGCAGTACGGCGCCGCGACCCGAAGGCGGGCCGCCGTTCCCGCGCGCAGGCGCG
CGGCGCCCACTGCGCACCCCCGTGGGGGACGTGCGGCAGCTGTGTGGCGGTGAGCGGGATTAGGGCTTTGCAGGGAGGG
GCTGGGTCGGGCGATACGTTCAGCATTGCGGTTTCCGGCGATTTGCGGCCGGTGCCCGTTTAACTCCGGCGTGGTCGCCTTC
CATGCCCTGACGGCATAAGAAAATAAAACCGCCATGCTGCGGTCATTCATGATTTTGTGGTGTAGCGATAAATAGTCATGCGAG
AAACGTTGAAGCGCTTAGCAACTGCACCAACTGTCATTTCAGGATCAGCAAGTAAGATTCTAATTTGTTTAACATCTTCTTCAG
AAAGTGACGGTTTTCTCCCTCCCACACGGCCCCTTGCGCGTGCAGCTGCAAGGCCTGAGCGCGTTCTTTCAATATTGCGGTTG
CGTTCAAAGCTAGAGAATATCGCCATCAGATGAGTATAGATTTCCCCTATAACTGGCGCATTTGTGTCTATTCTGTCCTTGATGG
CTATGAAAGTTATTCCGCGTTTCTTCAGGTCGTCGAGTAAAGTAATGACTTGACCCAATGAACCACCGAGCCGATCTAGTGCCC
AAACTACTAGGGTATCTCCCTCGCGCAATGCTTTCAGGCAGTTCTCCAGTTCCAGCGCACCTTTTTTGTCGCGCTTTGGGCCG
CTACGTGAGGTCTGATCCTGATAGATTTGCTCACATCCAGCTTTTGTTAGTTCGTCAACCTGGTGCGCCACATCCTGAAGATGC
GTAGATTTACGTGCATAGCCGATTTTCATTCTTTTCTCGCTAATTAGTTATGGGGTTATTGTTATGTTGATACAGTAACGAGTTTT
GTTACATGAGGGGAGTCATTTTTCGGGAGAAGTCAGGACTTTTCAAGACTGTCACAAAAACCATCGTTTTTGATACATTAATT
TAACCAATAGGTTGCAGATCAAATCGTCTGTAACAGCCTTTCTGGCTGTTTGTATATAATCATGAAAAAATGGTGAGTAGAGTT
TCAGGGTAACAGGGGATGCTTATGTCGGTTTTCCACAACTGGCTACTTGAGATCGCATGTGAGAATTACTTCGTCTACATCAAA
CGCCTTTCCGCCAACGATACCGGCGCAACAGGTGGTCACCAGGTAGGGCTTTATATCCCTTCAGGTATCGTTGAAAAACTCTT
TCCGTCTATCAACCATACCCGTGAACTGAACCCTTCGGTTTTTCTCACCGCACATGTGTCATCGCATGATTGCCCTGACAGCGA
AGCCCGGGCAATTTATTATAACAGCCGTCATTTTGGTAAAACCCGGAATGAAAAAAGGATTACCCGCTGGGGTAGAGGCAGC
CCACTTCAGAATCCTGAAAATACAGGGGCTCTGACGCTCCTGGCTTTCAAGCTTGATGAGCAAGGGGGGACTGTAAGGAAG
TAAATATTTGGGTATGCGCCAGCACTGATGAAGAGGACGTCATTGAGACCGCTATTGGTGAAGTTATACCCGGAGCGCTTATAT
CCGGCCCCGCAGGACAGATTCTAGGCGGACTATCTCTACAGCAAGCGCCAGTAAATCATAAATATATTCTACCTGAAGACTGG
CACCTGCGCTTTCCGTCGGGAAGTGAAATTATTCAGTATGCAGCCAGCCATTATGTGAAAAATTCCCTTGATCCGGATGAGCA
ACTTCTTGACCGCCGGCGCGTGGAGTACGACATATTTCTATTGGTTGAGGAACTGCATGTTCTGGATATCATCCGGAAAGGAT
TTGGCTCTGTGGATGAATTTATTGCGCTGGCCAATTCTGTCAGCAATCGCCGTAAATCCAGAGCCGGGAAGTCTCTGGAACTG
CACCTGGAGCATCTATTCATTGAGCACGGCCTGCGACACTTTGCGACGCAGGCCATCACAGAAGGTAATAAAAAACCCGATTT
CCTTTTCCCTTCCGCAGGGGCTTACCACGATACTGAGTTTCCCGTAGAAAATCTGCGCATGCTGGCAGTCAAGACTACCTGTA
AGGATCGCTGGCGTCAGATACTGAATGAGGCCGATAAAATTCATCAGGTGCATCTGTTTACACTCCAAGAGGGAGTTTCTCTG
GCTCAATATCGGGAGATGCGGGAGTCGGGTGTCAGATTGGTCGTGCCATCATCGCTGCACAAAAAATACCCGGAGGCGGTG
AGAGCTGAGCTAATGACGCTAGGTGCGTTTATTGCTGAGCTGACAGGGCTTTACGCAGATATTCCATAGATTATCTCCCGGCAT
AAATACCGGGAGGAGCGATCAGATTCGTTCAACCTTGCACGAATCGGCATTAACCGCTTTCAGGATATAAGGTTCAAGCAGTT
TGGCTACGGCTTCAAACACGGGCACCACTACGGAGTTACCGAACTGCCTGTACGACTGAGTGTCTGACACAGGAATGCGAAA
AGGCCTGCCATCTACTTTTTCAAAACCCATAAGGCGCGCGCACTCGTGGAGTCAGCCTGCGGGGCCGATGCGCCTGATTTTCT
TCGTTCGCGAAGTCTGTTTCACCTGTGGCCATATCCCAGCCACGGTCTATCAGAATTTCAGACCCGTCTTTGTGATAGCGAGCA
GAAAGCGTACGGGCAATGCTTTCTTTATTTTCAGGATTAACGAGGCCAAAACCGAATCCGTTACCCTTAGCTGCGTGCTTTTT
GGCGTAGTTATAGAGATACTCCCAGAGTTTCGGCGTCAGTATATATTTGCTGTCAACCACGGGTTCCAGCGTTCGCCAAATGAC
GGACGCTGTTCCGGATAAAAACGACTAATATCGCGCAGGGTAAAGCCCTGGTGAATGTTCAGATCACGACGGAAACCGACCA
AAACGATACGTTCTCGGTGCTGAGGTAAAAAGTGCTTTCCGTCGATAACTTTAGGATCGTTTTTGCCCATCTCAGCTGCATCCG

CAACTTCATAGCCCAGTTCGTCGAGGGTATCCATGATGACTTTAAAGGTTTTACCCTTGTCATGGCTCTTCAGGTTTTTAACGTT
TTCAAGAACAAAGATGGCAGGTTTTTTTGCGCGTATAATACGCGCCACATCGAAGAAAAGCGTTCCCTGAGCCTCACATTCGA
AACCATGCGCGCGCCCGAGCGAGTTTTTCTTGCTTACGCCCGCAAGGCTGAACGGTTGACAGGGGAAACCTGCTAGAAGTA
CATCATGATCCGGCACATGCTCATTAATGTAAGCATAGGCATCGTTTTCAGGTACTTCAGGTTTATCACTGAGCGTGACTTCCCG
AATATCGAGATTGAAAGTGTGTTCCTGAGCATCGTTAAACCAGTTAGCTTTATATGTGCGCACAGCCTCTTTATTCCATTCACTG
GTAAAAACGCACTGGCCACCGATGGTTTCGAAGCCCTTCCGTATACCTCCAATCCCAGCAAACAGGTCAATAAACCGGAAGG
CATAGTCAGGGTGATGTGCAGGCGCTTCCGGAAGCATTTTTCGTAGAAGTTCCTCTTCGGCTAACGTCAGCGTCTTAGGTGAG
CACTTACCATTAATCCAGCGATTAAGAGTCTCGCGACTCCACTCATTTTTACCAACTTTTCTAAGCAGTTCAGCCACGTACTTCT
GGTCATAGATTTCCAGCACCTGCCCGAGCAGCTTTTTATAATTTTCCTGTCGCAGTTGTTCTTCCGCTTCTGCTTTCTCAAGCAG
ATCCTGCGCCAGTAATTCAAATTCAGACATATTGCCTCCATTGGGTCTTATGGGTGAAACTGTATCACTCATTTGACCCAGATTG
AATGTTTTTATCTGGATATTTAAACAGGTTTATTGTTAGGTAACGCACGTTGGCCACGCTGGAGCGTCTTCTGGGCCTGCTGTC
GGCCTTTGAGGTCGTGGTATGGATGACGGATGGCTGGCCGCTGTATGAATCCCGCCTGAAGGGAAAGCTGCACGTTATCAGC
AAGCGTTACACTCAGCGCATTGAGCGACATAACCTGAATCTGAGACAACATCTGGCAAGGCTGGGACGGAAGTCACTGTCGT
TCTCAAAATCGGTGGAGCTGCATGACAAGGTCATCGGGCATTATCTGAACATAAAACACTATCAGTAAGTTGGAGTCATTACC
GGTTCTCTTTGTCTTTTAGTGATTCTATAAACCTCATTACGTCTGAATATAAAAATCTATTATTTGATTTATGTGGCTCATGAGGTT
GTGGGATGGTCTTGTTTTTGAATGTGCCAGTTTTCTTAATGGCAAAGATTAATTCACCTTCTGTTATTCCTAACATTTCAGCAAA
TGTTTTTGCTTCTATAGTTACTGACTTCATTTAATTAACTCTCATGGTATCGATTTTCTTTACCGGCATCTTTAACAATGGTGCTCG
TTTCTAGTGTTGCTGCGGTACGCTTCATCATCGTCTGCGGGGCGGTTGCGATAGTGAAGGAGCTGCCGGGCGTGAGCAAATC
TATCAGGCGCTGGCCGCTGATAATCTCCATCCGTTCACTGGCAATACTGACAGATTTTGAACCTGCGCCGGTTTTCCCGGTATG
GCAAAACAGACCGCGACAGTTATGACGTTTAAGCAACTTCTCGAACTCCTGTACGTGCTGTAAAGCAATATGGCCGCGATAGC
GTTTAGCCTGAATAAGATAGCGATATTTTCCTATTATTACCTGGCCGTCAATGCCTCCATCGCCGGTATAGCGTTTGTTTCTGATG
GTTCTGAAGCCATGCGCGGCTTTGTTGAATAAATCGAACTTTTGCTGAGTTGAAGGATCAGATCACGCATCTTCCCGACAACG
CAGACCGTTCCGTGGCAAAGCAAAAGTTCAAAATCACCAACTGGCCCACCTACAATAAAGCCCTCATCAACCGTGGCTCCATA
ACTTTCTGGCTGGATGATGAAGCTATTCAGGCCTGGTATGAGTCGGCAACGCCTTCATCACGGGGAAGACCTCAGCGCTATTC
TGATCTCGCCATCACCACCGTTCTGGTCATTAAACGCGTGTTCAGGCTGACCCTGCGGGCTGCACAGGGTTTTATTGATTCCAT
TTTTACACTGATGAATGTTCCGTTGCGCTGCCCGGATTACACCAGTGTCAGCAAGCGCGCAAAGTCGGTTAATGTCAGTTTCA
AAACGTTCACCCGGGGTGAAATCGCGCATCTGGTGATTGATTCCACCGGGCTGAAGGTCTTTGGTGAAGGCGAATGGAAAG
TCAAAAAACACGGCAAAGAACGCCGTCGTATATGGCGAAAGTTGCATCTGGCCGTTGACAGCAACACACATGAAATCATCTG
TGCAGACCTGTCGCTGAACAATGTGACGGACTCAGAAGCCTTCCCGGGTCTTATCCGGCAGACTCACAGAAAAATCAGGGCA
GCATCGGCAGACGGCGCTTACGACACCCGGCTCTGTCACGATGAACTGCGGCGTAAGAAAATCAGCGCGCTTATCCCGCCCC
GAAAAGGCGCGGGTTACTGGCCCGGTGAATATGCAGACCGTAACCGTGCTGTTGCGAATCAGCGGCTGACCGGGAGTAATG
CGCGGTGGAAATGGACAACAGATTATAACCGTCGCTCGATAGCGGAAACGGCGATGTACCGGGTAAAACAGCTGTTCGGAG
GTTCACTGACACTGCGTGACTACGATGGTCAGGTTGCAGAGGCTATGGCCCTGGTACGAGCGCTGAACAAAATGACGAAAG
CAGGTATGCCTGAAAGCGTGCGTATTGCCTGAAAACACAACCCGCTACGGGGAGACTTACCCGAAATCTGATTTATTCAACAA
AGCCGCCATGCGCTTCAAATCCTTCCAGCAACAGTTCTTCAAACACAAAAGGATCAATTTTCCTCAGGTAGTTAATTTTTGTGG
GAAGCCCGGCAACGTCTTTATGCGCTCCAGCACCCGCCGCGCACTTTGCTGCTTCCTTTTGTGTCGTCGGTTGCGTACTGAAC
GCCGGAAGAATACAACGGCAAACAGTGCGATGGCGCTGCAAGCCCATAGAATAAGGTTTTCTGTAGTGGGGAAGGGGAAC
ATGGTGATAGTGTGCTTTCTGTGGGTAAAGAAAAGGGCGGTTAAACCGCCCTGGTGTTTAGCGACGGCTGTAAACCTGCCAC
GAAGCGCTGCCTGACTGATTTTGGCAAATCCGCCCGTAAAGTACGGTGCCGGTCGAGTAGCGGGCGCCATTCAGATAGCAAT
AGCCGCTGGATTGCAGCAGTTCGTTGCGCAGCTTTTCTTCCTGCTTTGATAGCCGGGACTCCGCAGACTGCAAACGAACGGA
TAATTCGTTAATCTGGCGTTGCTGGTTATTCATCTGGCTCTGCATCGCATTACCTTTATCTTGGCTGACGCAACCAGTTAAGAGA
GCTGTACAGGCTAATGCACTTAATATAATTTTTTCACGTTGGCTCCTTAAATTGAGATTATTCCTAGCCCGCCTATAAGCGAACTT
TCCCGTATTTACTTATGATCTGGCTTATCATCGACTGGTTACTTCCACCTTCGCCATTATCCGGGCATTCATTAAGAAAAGCCTTC
CTGGCATCCCTCGTGTGGTTGGGTAAAAAGCCGTGCTTGTTCTTTTTAACGATATTGAAGAAAGCAGCTTCAGCACTGTTACA
CTCGCTTCCGCCGCTATCGCCGGTGAGCTTGCCCGCCATGCACATAATAACTTTGCAGGGATCTTCAGCATGGCTGGCAGGAA
GATAAAGCAGACTACCAGCTGCTATCAGAGGGATTAAGAGTTTCTTCATTGTTTTGTCCTTAACAGTTTGTTCAGATATACACC

CGCCAGAATGTTGATAACGGTAAGTAATATTAATAATAACGCAGAGTTATAAATAGATTTGTAACCTATATCGCCTGCGATATATT
CGACAATAAAAACGAAAATCGTTAACATTGCGAACAACTTAATTAACTTTCCTGTAATTTTCTTAACGATATAAGCAACTGAATA
AGAGCCGGATTTGATGATGCTGGCGAGTAACCTGATAGCATGGACTCGGAATTTTCTTAAAATGGGCGGAATGGAAAATGCC
TTTTTCTTCCTTCTGCCTTTTTGCTTTTCCGCAAAATCAATAACATCGCCCATTTTTATACTCTCCGGTTATATCTTTAGATCATCAA
GTGATAAACCACTATCAAGCAGCTCTTTGAGCCATGTTGGTCGGCGGCCAGTCCCGGACCAGGTATTTTCTGCGTTCCTGGGG
TCGCGGTATTTTACTTGTCTATGTTCTCTGGGGTTGGCAACGCCCTGCATTTTCATGGCTTCCAGGCTAATGCCAGCGTCCAATT
TTGCAGTTAGCCAGGCCGGGCGCTTCCCTATACCAGTCCACGTATTAAAAGGGTTATCCGGGTCACGATACATGGGTTCACCT
TTGGGGCGTTTTTCTGATTTCAGGGGAACCAGCATTACCTTCCTTCCTTCAGAGGTTTTCTTTTCGTTTCTTCTTGACACGATC
TCATTAGCCCTTATCATAAACATAAGTCGTCGAAATTACAATTACGCGGTGAAACGTAAATGAGTAAACATCCAAAACTTCTGGT
TCTCGCTCTGGCCTGCCTTGCTTGTGCTGGCCGTGCCAGTGCTGCGCCTGCCTCAGATGAAGTTGCCAGGCTTGCGCAGAGA
TGTGCGCCTGATGTTTCACCCTTAACAATGGCGTACATCGTCGGCCATGAGTCCTCAAATGGGCCGTACAGGATCAATATTAAC
GGTGGTAGTACCCAGTTAAAACAGCAACCACGTACTGAAGCTGAGGCCGTCAGCGTTTCGAAAGTTCTGCTGAAGGATAATA
AAAGTTTTGATATGGGCCTTGCACAAATTAACTCAAATAATTTAGTGGGCCTGGGTCTTTCGGTTGACGATATTTTCAAGCCCT
GCATCAACCTGCGGGCGAGCCAGACCATCCTTAAAGCCTGTTATGATAGCGCCCTGAAATCCTATCCAGCCGGGCAGGTTGCG
CTGAGACACGCGCTTTCCTGCTACAACACCGGCTCACTCATAAACGGGATTTCTAACGGGTATGTCACGAAAGTTATCAACGT
GGCGCGTCAATCAACTGATTTGAAAATCCCTACGCTGCTACCTGATGGCCAGACCAGTGAGGACAGCACCGCGACTGAGCCT
CAGCAGGCAAAAAGTACGGCCACGCAGTATGACGGTGAACAAGATGTTTTTGGTTCGGGTGATGGCGATGCCTTCAGCCGA
AATAATACGGATGCCTTTTTAACCAAACAGGAAACAGCGAAGGGGGAGTGAGGTTATGGATGGAACGTTTGTACCTTGTATT
GCAATCACAGATCCTGGAGCTATTTGAAACAGGAAAAGTGAAGGAGGTAACGATAAAACGGGTTTCATTAAAGACGTGGTAT
CCCGTTTTTCAGATAGACGATGAACAGTTGGGCCAGATCGCATGTTCCATTCGGGTTAACAAAGAGCATGAACTACGAACCTG
GGCTGATTTAAGGCTACTGGCAGAGTTTTTGAAAGATAAGTGTGGCGTTGAAGAATGCCGGTTAAATCTGCAATCAACAGAA
GATAGTGAGTAAGGAGAAAGTATGACCACGTTGTTTAAGAAGTATGGCCCTGCGGTAGTTATGGGCGTTTTGTCCATTGCCCT
GCCGCAAATTGCGCTGGCCGCTGGCACCGATACTGGTGAATCAACCGCTACATCAATCCAGACGTGGTTGAGCACATGGATTC
CAATTGGTTGTGCTATTGCGATCATGGTTAGTTGCTTTATGTGGATGCTTCACGTAATCCCAGCCAGCTTTATTCCTCGTATCGTA
ATCTCGCTGATTGGTATTGGTTCTGCATCATTTCTGGTTTCCCTGACGGGCGTAGGAAGCTGAACAACGCGAAAAGGGGGGA
CTTTTGTCCCCCAAAGTGAGGACTACAAAGATGTTCGTTGACGGGAAAAGACCGCTTTTCAAAGGTGCGACTCGCTTACCTC
GCGCGCTGGGTGTACCACGTAATGTAGCTATGATGATATTCATGATTTCTGCCTCGCTTTTTATGATTATTCATATGTGGGCGATC
CTGGTGTTCGTCTTTTTGTGGATTCCTTCAGCTGCATTAACAAAATATGACGACCGCATGTTTCGAATTATGGGCCTGTGGTTG
AAAACCAAATTCAGTAATTGGTTTGATTCTCCGTTTAAGCAGTGGGGAGGATCGTCTTATTCCTCTGTTGACTACAAACGTAAG
GGTTTAAAATAATGAGAGCTGCCACCGCTACGAAGCCAAAAAAATTGATGCCTACCGTAAGGAGCCATCAGTAAATAAAAAG
TATTTGCCCTATTCTTATCACCTCAATGATTACGTGATTTCGATGGAAAACGGCGATCTGATGGCTTTTTTCAAGCTGGATGGCC
GCACACATGACTGCGCATCAGATCGGGAACTGGTCACCTGGCATAAAGACCTTAATACGCTGGTCAAGAGCTTCGGAACAGA
CCATGTAGAGCTGTGGACGCATGAATATCACCATGAGGCTAAAGAGTACCCGGATGGTGAGTATGACCATTTTTTCCCTGCTTA
TGTTGATCAATATAACCGTAAGCTGCACGGTGATTCCAAGCAGCTGATTAATGACCTTTATCTGACCGTTATTTACAAACAGGTA
GGGGATAAAACACAGAAGTTTCTGGCGAAATTTGAAAAGCCGACTCGTGACGAAATTCAGCGAATGCAGAATGAGGCGCTT
GAAGGTCTGGAAGATATTTCTGAACAAATCCTGGAAGCAATGAAGCCGTATGGCATTCAGCAGTTGGGTATCTATTATCGTGA
CAAACGCGGTGTTGAAATTCCTGCGCCTGATAAAAAAGAACGTGAAGAACTTGCTGAAGTCGATGAATCAGACATTTTTGAC
GAAGCCATTGTTATCGAACGCAACGAGCCTGAACCTTCGCAGGCTCACGCTTATTCAAAAGCGCTGGAGTTCCTTTATTTCCT
CGCAAATATGGAATGGGCCATCGTGCCTGTTTGCCGTGATCGTATCCGTGAGTACATCATGGACAACCGCCCTGTTAGCTCACT
GTGGGGGATGTTGTCCAGATCAGAACGGTAGATCACAACTTCTATACCACCGGCATTGAATTTCGTGAATACGAAGAAGATAC
AGAGCCAGGCCAGCTTAACATGCTTAAAGAAGCCGATTTTGAATACCTTCTGACGCAGAGTTTTTCTTGCCTCTCTGAATCTTC
AGCTAAAACGTTTCTGACGCATCAGGAAAAATCTTTGCAGGAAACGCGCGACCGTGCGCAAAGCCAGCTGGCACAGCTTGG
TACCGCGCTCGATATGCTGACGTCCAGAGAGTTCGTGATGGGCTACCATCATGGAACCGTGCATGTCTGGGATAATGACCAAA
ACGCGGTACAGCGCAAAGCGCGTCGTGTGAAGGTTATGCTAACCGGCTGTGGCGTGGTTGGCGGGACTCTCAGCCTGGCCT
CTGAGGCTGCATATTATGCGAGACTGCCTGGCAACCAGAAATGGGCGCCGCGCCCGGTTCCGATAAACTCATGGAACTTCCT
GCACTTCAGCCCGTTCCACAATTTTATGCGTGGCAAGCCTGACAATAACCCGTGGGGGCCAGCGCTGACCATGTTCCGCACG

ATCAGCGGTACGCCACTCTATTTTAATTTCCATGTGACCCCGCTTGAAGAACTTTCCTACGGTAAACGCCCGCTGGGCCATGCG
TTAATAACGGGTATGTCGGGGGAAGGTAAAACCACGCTGCTTAACTTCCTGCTGGCGCAGTCAATGAAGTACAACCCGCGGC
TTTTTGTTTATGACCGTGACCGCGGTATGGAGCCGTTCATTCGAAGCGTTGGTGGCTACTATAAAGTTCTGCAACAGGGTATG
CCGTCCGGGTTTGCCCCGCTTCAGATTGAACCGACCAAACGCAATATTGCCCTCATTAAAAACCTGTTCCGCATTTGTGTGGA
AACCACCAATAACGGGCCTATCAGCGCAACGATGGCTACCGAACTGGCTGAAGGCGTTGATGCGGTTATGGGGGAAGGCTC
ACTTATTCCACGCGAGGCGCGCACCGTTACTATCCTGGACGGGTACGTGAATGAAGTTGTGGAAAATGGCGTATCACTGAAA
GGGCTGCTGCGCGAATGGACGCGCGAAGGCCAGTATGGCTGGCTGTTTGACAATGATAAAGACAGCCTGGATCTCAGCGCG
AATGATATTTTTGGCTTCGATTTATCCGAGTTTATCGCAGCCAAAGAGGAAGTATCCAGCCCGGCCCGTACTCCGCTCATGATG
TACCTTCTGTACCGGGTACGTGACTCCATCGACGGCAAACGCCGCGTCATTCAGTGCTTTGACGAGTTCCACGCCTACCTTGA
CGATCCGGTTATCGAGCGTGAAGTTAAGCGTGGTATCAAAACTGACCGTAAGAAAGACGCTATCTATGTGTTTGCCACGCAGG
AGCCGAACGATGCGCTGTCCAGCCGTATTGGCCGCACGATCATGTCGCAGACCGTCACAAAAATCTGCCTGCGCGATCCGGA
AGCTATCCGAGAGGATTATGCCTTCCTTACTGATGCTGAATACGACGCGCTGATGTCGATTACCGAACACTCCAGACAGTTCCT
GGTTAAACAAGGGCAACAGTCTGCGATTGCTTCTTTCAATCTCTACCCTCGCAACAGCGACGATATTGATGCAGATATTAAGAC
AATGGACAACGTTCTTAGCGTGTTGTCCGGTGAACCACAAAACGCCGAAATTGCGCATGAGCTGGTTGAACGGCTCGGTAAT
GACCCTGAAGTATGGCTCAAAGAATACTGGCGCCTGACGGCTTAACAACGAGGCAAAACACCATGAAAAAAACACTGACGG
CAGTATTGCTGACCACCGGCCTGATACTGGGAGGCGCGCAAAGCGCTTCCGCAGGCATCATCGTGACCAACCCTACTGAGCT
GGCTAAACAGGTCGAGCAGCTTCAGCAAATGGCGCAGCAGCTGGAGCAGCTTAAAAGCCAGCTGCAAACGCAGAAAAATAT
GTATGAGTCGATGGCAAAGACAACCAACCTGGGCGATCTGCTGGGGACGTCTACCAGCACGCTGGCAAATAATTTGCCGGAC
AACTGGAAGGAGATCTACAGCGACGCCATGAACTCCAGTTCTTCCGTCACGCCTTCAGTTAACAGCATGATGGGCCAGTTTAA
TGCGGAAGTTGACGACATGACGCCCAGCGAAGCAATTACCTACATGAACAAAAAGCTGGCTGAAAAAGGCGCTTATGACCG
TGTTATGGCAGAAAAAGCCTACAACAACCAGATGCAGGAACTAACCGATATGCAGGAGCTGACGGAGCAGATTAAAACGAC
TCCAGACCTGAAATCGATTGCTGACTTACAGGCCCGTATCCAGACGTCACAGGGTGCTATTCAGGGTGAGCAGGCGAAGCTG
AATCTGATGAACATGTTGCAGCAGTCACAGGACAAGCTATTACGTGCGCAGAAAGAACGTGCCACCCACAATTTTGTTTTGG
AACCGGCGGGGACGTTACCGCGTCACCTTCAATTAACTGAGGTAATTATGAAAAAACTACTGCTTGTTATCCCTTTCCTCCTAG
TGGCCTGCGATGCCTCGCATGACGTGGAGTGGTACAAAAAACATGAGAAAGAGCGCAAGGCAACAATTCAGGAATGCAAGA
AAGACGCGGATGAACTTCAGAAACCTGATTGCAAAAACGCGCGCGAAGCCGATCGTCAGCTGTTTGTGTTCGGCAAAAAAG
ACGGCGAAATCAATTCACCGAAAATTTAGGAGTAAGGAGGCAATATGGCATTCACCCTAGTCGCAGACATTTTCGCAAAAGTA
GACGGGGCGATTACGTCAATGGTGAGCGCCAATGTTGCCACCATTATCTCTGATGTAACGCCTCTGATTGCCACCTGTCTGACA
ATCAAGCTGATGGTTCAGGGGATGTACTCAGCGTTTAATCCGGGGGCGGGCGACAGCCTGAGTTCGCTGATTAAAGAGTATC
TTTCCATAGCCCTTATCCTGAGCTTTGCAACGGCGGGCGGCTGGTATCAACAGGAACTGGTCAACGTGGCGCTTCACCTGCC
GGATGATTTTGCCGGGATACTGTCTGCCCCTAATAAAGTCGGTGCAAGTGGCGTACCGGCGATTATTGATAGCGGTATTGAAA
AAGGTATCAAGATCGTCAACACCGCATGGGAAGCCGCAGACGTGTTTTCATCGAGCGGCCTGGCCGCGTATGCCATTGGCGG
CATTATGATGATTGCTACCGTTGTGCTGGGCGGCCTCGGTGCGGGCTTTGTGATCATGGCTAAGATCCTTCTGGCCGTTACGCT
TTGTTTTGGCCCGATTGCAATCTTCTGCCTGCTGTGGGGAGCGACAAAAAACATCTTTGCTCGCTGGCTGGCGTCGGTCATTA
ACTATGGCCTTGTCGTCGTCATTCTTGCGCTCGTGTTTGGTTTCATCATGCAGATGTTCGACAACCTCCTGTCCTCGATGAACTC
TGATGCCGCTTACTCATCAATCACTGGTTCTATCTCCGCCTTATTACTGACGGTCATTTCCGTTTTCGTTCTGTTCCAGATTCCGC
AAATTGCCGCCAGCTGGGGTAGCGGTATCAGCGCCGGAGTTGCTGACGCCGCACGCTCTACGGGTTCTTCCATGCAGGCGCT
TGGCAATATGGGCAGCCACGGCATGTTTGGCGGTAATGCGTTCAGAGGCGGTAACAGTGGCGGCGGCCAGCAATCGGCAG
GTGGAGGAAGTGGCAGCAACAGCGGAGGAAGCAGTGGTTCTAATTTAAGTGGTAAGGCAAGGGGCAGTCGCGGGAAGAA
GGCTGCATAAAATTAAAACAAAAGTCGTTGAAATTGCAATTTCGACGACTTATTATAATTAGTACGTTCAACAACCGATAATGG
ATGCCGTAATGCGCAGCTTATTGCTTATGGGAGTTCTTCTGATTAGCGCCTGTTCCAGCGGGCATAAACCGCCACCGGAGCCG
GACTGGAGCAACACCGTTCCAGTAAACAAAACAATCCCGGTTGATACGCAAGGTGGTGCAAATGAAAGCTAATAAAAAAACA
GGGCTTACACGTGAAGCCATTAAAGAGTTCAACGAAAGCCGTAAAGGGCTTGAAGTTGATCTGATGGATGAAGTGCTGAAG
TCCCGGCGTACCGCCTGGATGGTTGCCACCGGTTCAGCGGTGGTAACTGTTTTTGCACTCTCTTTAGTTGGTTACGTGGTGCA
TAAGTACAGCCAGCCAATCCCCGCACATCTGCTAACGCTCAACGAGGCCACTCACGAAGTACAGCAGGTCAAGCTGACCCGC
GACCAGACCTCTTATGGTGACGAAATTGATAAGTTCTGGCTGACACAATATGTCATTCACCGTGAGAGCTATGACTTCTATTCA

GTTCAGGTCGACTATACGGCCGTTGGCTTAATGTCCACGCCGAACGTGGCAGAGTCTTACCAGAGCAAGTTCAAGGGCCGCA
ACGGTCTTGATAAGGTTCTGGGCGACAGTGAAACGACCCGCGTGAAGATTAACTCTGTGATCCTCGATAAACCGCACGGCGT
AGCAACGATACGCTTTACTACGGTTCGCCGCGTGCGCAGCAATCCCGTTGATGATCAGCCGCAGCGCTGGATTGCCATTATGG
GGTATGAATATAAATCGCTGGCGATGAATGCTGAGCAGCGTTATGTCAACCCGCTGGGTTTCCGCGTGACGAGTTATCGCGTC
AACCCTGAAGTTAACTGAGGGCTGCCCCATGAAAAAACTACTTCTTTCAGCAGTCGTTTTGTCAGTCCTGGGAGGCGCGGCC
ACTAACGTTATGGCGCTTGAGGTTGGCCGCAATTCTCCTTATGACTATCGCATTAAAAGCGTTGTTTATAACCCTGTTAATGTGG
TCAAAATTGACGCTATCGCCGGTGTGGCTACCCACATTGTTGTCGCGCCTGACGAAACCTATATCACTCATGCTTTTGGCGATT
CTGAAAGCTGGACGTTTGCGCACAAAATGAACCATTTTTTGTGAAGCCGAAACAGGCCATGAGTGATACCAACCTGGTGATC
GTCACCGATAAGCGCACCTATAACATCGTCCTCCATTTCATCGGTGAAGAAACGAAGAAAAATGCAGACGGTACGGTATCAAA
ATCCTTTATTGAAACGCCGTGGGCTGTGCGCCAGGCCGTTCTTCAGCTGACCTATGAATATCCGTTTGAGCAGCAGGAAAAAG
CCAAAAGCGCGGCTGATAAAAAACGCATTACGCAGAAGCTGAAGCAGACGGCTTTTGCGGGGGCGAAGAACTATCAGTACG
TAATGAGCGAACAGCCTGAAATGCGCAGCATCCAGCCGGTTCACGTCTGGGATAACTACCGCTTTACCCGGTTTGAGTTTCCG
GCCAATGCGGAGTTACCGCAGGTCTACATGATTTCGGCCAGTGGCAAAGAAACGCTGCCTAACTCTCATGTTGTGGGTGAGA
ACCGCAACATCATCGAGGTGGAAACCGTCGCTAAAGAGTGGCGTATTCGTCTGGGCGATAAAGTCGTTGGCGTTCGTAATAAT
AATTTCGCGCCGGGCGCCGGTGCGGTAGCAACCGGTACGGCTTCCCCGGATGTGCGCAGGGTTCAAATTGGGGAGGATAAC
TGATGGCCCGTAAAAGTGTCGATGTAGATCAGGAACTCGATGAAAACACCGGAGACGGTGAATTCGAAAGCGAGCGTGGCG
GATTTAAAGGCAGTAACCGCCGTTCGGCTCCTGGTATGAAAGCCTTTGTCATACTGATGGCGCTGCTTGCTTTGGTATTCATCG
GGATTACGGTCATGGGTAAAATTCGCACCCCGGCTAAAGCTGAAGCTGATAAAGACGGTGGTAAAGCGCAACAGGCCAATAC
ACTGCCAAACTACAGCTTTAACAGCGATCCTGATGTTAATAAACCTGCAACTGCGCAGAATAGCGCCACTGATGCCCGTGCTG
TGCAGGCTGCCGCACAGGCAGATGCAGATGCGGGCAGCAGCAATACCGCCGCGCGTACCTCTAATAAGCGTAAAGAACCTTC
GCCTGAAGAACTGGCTATGCAGCGTCGTCTGGGCGGCGAGCTGGCCCAGACTAATCAGGCGGCTACAAGCAATAGTCCCGG
AGTGCAGCCCCAGGACAACGAAACAAGCGAAGGTAGTTCAGCACTCGCTAAAAACCTGACTCCTGCAAGGCTGAAGGCTAG
CCGCGCTGGAGTCATGGCTAATCCCAGCCTGACTGTTCCGAAAGGCAAAATGATCCCCTGTGGTACCGGCACCGAGCTGGAT
ACCACTGTTCCGGGTCAGGTTTCCTGCCGGGTTTCACAGGACGTTTACTCAGCTGATGGACTCGTTAGGCTGATTGATAAAGG
CTCATGGGTTGACGGGCAGATTACCGGTGGTATCAAAGACGGCCAGGCGCGCGTGTTTGTTCTCTGGGAGCGTATCCGCAAT
GACCAGGACGGGACAATCGTTAATATTGACAGTGCCGGAACGAACTCACTCGGCAGCGCGGGGATTCCGGGCCAGGTGGAT
ACCCATATGTGGGAGCGTCTGCGTGGTGCGATCATGATTTCGTTGTTCTCTGACACCTTAACGGCGCTGGTTAACCAGACGCA
GAGTAATAACATTCAGTACAACAGCACAGAAAACAGCGGTGAGCAGCTGGCGTCTGAAGCACTCCGCTCTTACATGTCTATCC
CCCTACCCTCTACGATCAGCAGGGTGATGCGGTGAGCATTTTTGTTGCCCGCGACCTCGATTTCAGCGGCGTTTATACGCTCG
CAGACAACTAAAAAAGTGGGCGCTTAGCGCCCGCTTTTCTTCAGGAGTAATCATGACTGATGCAGCTTTCTATCAACTTGGCC
CACTGCGCGAGTATTTAGAAGATCCTACTGTTTTTGAAATTCGCATTAACTGCTTTCAGGAAGTTATCTGTGATACGTTCAGCG
GCCGCAGGGTTGTGCAGAACGCGGCAATTACGGCAGATTTTATTAGGAACCTTGCTAAATCGTTGGTGAGCAGCAACAAGCT
GACCATGCAGGCCATTAATGACGTGATCCTGCCTGGCGGGATCAGGGGCGTTATCTGTCTGCCCCCTGCGGTGATTGACGGTA
CAACGGCCGTAGCGTTTCGTAAGGATTTGGCGGCCGATAAAAATCTGGAGCAGCTGACCCGCGAGGGGATTTTCAGTGACT
GCCGGAAGATTACCGGCAGCAAGCAAAGCCTAACGGATGATGATTTTTTCCTTAAAGAGCTGCACAGCAGCGAAAAATGGC
CCGCATTCCTGCAAACCGCCGTTGAGAAGAAACGCACTATCGTGATCTGCGGTGAAACCGGGTCGGGGAAAACGGTACTCA
CGCGCGCGCTGTTAAAATCGCTACATAAAGACGAGCGTGTAATTATTTTAGAGGACGTTCACGAAGTCACGGTCGATCACGTT
GTAGAAGCCGTTTATATGATGTACGGCGATGCAGGAAAGATCGGCCGCGTCAGCGCCACTGATGCCCTGCGAGCCTGTATGC
GTCTGACACCGGGCCGTATCATCATGACTGAGCTTAGGGATGATGCTGCGTGGGATTATCTTAAAGCACTTAATACCGGCCATC
CAGGCGGTGTTATGTCAACGCACGCTAACTCTGCGCGCGATGCCTTTAACCGTATTGGGCTGCTTATCAAGGCGACCCCTATC
GGCCGTATGCTCGATATGAGCGATATTATGCGAATGCTCTACTCCACCATTGACGTTGTGGTGCATATGGAAAAGCGGAAAATC
AAAGAAATTTATTTTGACCCTGAATATAAAATGCAGTGTGTGAACGGGAGCCTGTAATGAAAAACTTAGCAACCTGGCTTCTG
GCCGCAGCATTTACGACAGCCGCCCTGCCCGCCTTTGCGGTGGAACCATCCGTTCAGGTTGGCTACTCGCCTGAAGGGGGGT
AAGCGGGAACCCCAGAAAATTCCGCCATTCCGCATTGTGGAATTTTTGGGGGTGGTCCGCGGCATGACGACCCGCCAAAGG
CTACCGCATCTATCTGAGGCGCTGTGGCGCGACGCTGAAACCCCGGGGAATTCCGCTACTCCAGTTCCAGGTCCGCAATAGTT
CGTCCAGGTCGGGTGGCGTACGCCGGAAACGCCGATTTTTCCGCAACCGTTTCTGCGTTGTCGGCAAGGTGGTCTCAGAGA

GCGCTCAAGAAGCGGTTTTTCTCGTATGTTTAGCTACGGTGGCCTAGGAGCCTGCGCGGCAGAAGCCAAATCGGCGGAAGA
TCGAGGACGATACGGGGATGGCTGCCAGTTACCTTCCTTACCGACCCGACCAATCCTATCTGCTGCCCCCTTCTCTGGGAGAG
TGGCTACCTGAAGGGCATCTTGCCTACTTCATCAGCGAGACTGTCGATACGCTGGACTTGAGCGCATTCCACGCCCGGTATGC
CGGCGGTGGTCCGGGCAATCAGCCGTTTCATCCGGCGATGATGGTCAAAGTGCTGATCTATGGTTACGCGAGCGGCGTCTTC
TCTTCGCGCAAACTAGCCAGGAAGCTGTACGAGGATGTCGCGTTGCGTGTGCTGGCCGCTGGAAACTTCCCGGCCCACCGC
ACGCTGAGTGACTTCCGTGCCCTACACCTGACCGAGCTTGAGAATTTGTTCGTTCAGGTGGTGCAACTGGCGCGCGAATGTG
GGCTGGTGAAGCTTGGCACGATCGCGGTCGACGGCACCAAGGTAAAAGCCAACGCCAGCCGCCACAAGGCGATGAGCTAT
AAGCGCATGAAGCCGGCCGAGGACGAATTGCATTGCGAGATCAAGGCGCTGCTTGATCGCGCCAAGGCTACCGACGACCAG
GAGCGTAACGAGCCGGAGCTGGACATTCCTGCCGAGATTTCTCGCCGCGAGAAGCGCCTGGAGGCGATCCAGGCGGCAAA
GGCGCGCCTGGAAGGGGTCGGTTCCGGCTGAGGGCGAAATGACACCCTAAGCTTTCGGTTCCTTGGGCCAAAGATATTCGC
CAGTCAGTAGAATGTGCGCCCAGCCCAATGGGGATATGTGGGGAAGAAATTCAGGGGGAACATCCAACCCTTCGTTCCGCCG
CTCCGTGACGGCATGACCAAGATGGACGGTATTCCAGTAAATGATCACCGCAGTCAATAAATTGAGCCCAGCGATTCGGTAGT
GCTGCCCCTCTGTCGTGCGATCGCGAATTTCCCCCTGCCTCCCGATACGGAGCGCATTTTTGAGCGCATGGTGGGCCTCTCCC
TTGTTAAGACCGATCTGAGCACGCCGCTGCATGTCCGTATCCAGGATCCACTCAATAATGAAAAGGGTCCGTTCAATACGACC
AACTTCACGAAGCGCAACTGCAAGGTTGTTTTGTCGTGGGTAAGAAGCGAGCTTGCGCAGGAGTTGGCTGGGCCTGATTTT
GCCAGCGGTCATCGTCGCGGCACAACGGAAAATATCAGGCCAGTTCGCAACGATAAGATCCTCCCGGGCTTTTCCACCTACC
AACTTGCGTAACTCCCTGGGGGTCGTATCGGGATTAAATACGTACAACCGCTTCGATGGCAGATCCCTGATTCGCAGAACGAG
ATTGTAGCCGAGCAGGCTACTGGCTCCGAACAAATGGTCGGTGAATCCTGCTGTATCGGCATACTGTTCGCGAACATGGCGAC
CGACCTCGTTCATCAGTAGTCCATCGAGAATATACGGTGCCTCGCTCACGGTCGCCGGGATCGACTGACAAGCGAATGGCGC
GAACTGGTCGCTTACGTGAGTATACGCTTTGAGGCCGGGAACAGAACCATATTTGGCATTGACCATGTTCATGGCTTCGCCAT
GCCGCGCTGTCGGGAAAAACTGACCATCGCTCGATGCTGACGTGCCCATCCCCCAGACGCGTGACATCGGCAGTTTACCCTG
CGCGGCCACCACAATTGCCAATGCCTGGTTCATGGCTTCGCTTTCAACATGCCAGCGGGCAAGGCGTGAGAGCTGCCAGTAA
TCATGCGTGTTTGTAGCTTCCGCCATCTTACGCAGGCCCAGATTGAGCCCTTCAGCGAGCAGGACGTTGAGCAGACCGATCC
GGTCGCGACATGGAGCCCCGGTTCTCAGATGGGTAAACGCATCTGTGAAACCAAGGGCTGCATCAACTTCAAGCAGCATGTC
GGTAATCCGAACGGACGGCATTCGGCGATACAGATCCAGTATGAGTGCCTCGGCACCATCCGGCACGTCTGCTGTCAACCTGT
CGATCCGCAACGTTCCATCTTCTATGCTACCGTGCGGAATAGTGCCGTTACGGGCAGCCCGGGCCAGCCGCTTAAGAGCGATC
GTGAGTCGCGCCTTTCTGTCTGCCAGCCAATCCTGTGGGTTGGAAGGCACGGCCAGTTTTGCATTTTCCTGCGCCGCGATCAT
CGGCACCAGTACCTGCTTGAGGTCACCATAGCGGCGCGAATGAGCGAGCCAGACATCTCCGGAACGAAAAGCATCCCGGAG
GTGAAAGAGTACCGCCACTTCCCAAAGACGGGTATCTCCTTTTTCCTGAGCTCGTAAATGACGGTTCCATTTGGAGCTGGGCC
GCAGGAAACGCCTTTCTGGCGATGCAACACCTTTCATCTCTCCGATCGACAAAGCTGCTGCTACCAATGGTCCGGCGACCGG
CGCGGCTTCGAGCTTCAGACAGCGCAACATGCGGGGCGCATAACGACGAAAGCGATGGTATCCCTGCCCGACATATGCAAG
AGGCTCATCGGCTAGCGTGTTGCTGAGTTGAGTCCCTGTCGCTACCAGTTGAGCGAGCCGGTCCCATGCAACCGAACTGGCG
ACAGCCATCTCCAGCGGGGTTCCGTCACTGCGGGCCTCAAGCAACGAAGCTCCCAGCGCGGTGAAGGTACGGATCGTATCC
GTGAGTGTGGCTTTAGAGCCGGAAATTGTTTCGTCATGCTGGCGCTTCGCTTCCCGCCAGGTTTTTCCTACGATCCTGTCATG
GGTTTCGACTATGGCATCAGCAATCGCCGCTTCCCACTCCACAACACAGACGGCAAGGATCGCCCAGCGGCGGTCCGAAGTG
ATGTCACGCAAACCGTCGGTGAAGTAGCGTTCACCCTGCCGACGCAGCCGGGCAATGCGATGGGCAGGTATGCTGGCCAAA
GCACTATGATTGATATTCAGGGTACGCAGAAATTCGAGCCTGTCGAGCAAACGGTTAGCAGCAGCCGAGTTGTTACCAACCTC
GAAGTTGCGAAGCCAGATGAAACGACTGATATTGCCGGCGAGCATTTCACTCAGAAGTTTGTCCAGGTGATCGCGAACATCC
GCTGTTAAATTTTCCACAATCCGCGTTTCAATCCGCCGCTCAGCGGCGACCAGAGCATCCGCGCACAAGCGCTCGATTGTCGA
TACTGCGGGCAGAATGGTGGAAGTTTCCCGACACCGCACAATAAAACGATGAGCAAGATCCTCGTTTGATCTGGCATCTTCG
GCCTGGCCGAAAGTCCACTCCCGCAGATCACGGGCACCACGGCCCGTGAAGGTCTTGTAGCCGTAAATTTCGCGCAGCGTGT
CCATGTGCTGCTGACGGGTTTGGCGCCGTGTGGCATAAGTGAGAAGCGCATCAGCCGGAACTCCAAGCTGAGCACCGACGA
AGGAAAGGACTTCACGCGGGATCATCTCACCAGGAGCCAGTGCACGGCCCGGATATCGTAAGGCACAAAGTTGCAGGGCAA
AGCCAATCCTGTTTTCCGGTCTGCGGCGCTGCCTAATGTTTTCCAGGTCATCATCGCCCAGCGTGTAGAACTTCAGTAGCGAC
AGTTCGTCCGTGGGCAGATCGAACAGCGCTGCTCGCTGCCGTTCGGTGAAAATATGGCGTCGTGACATACAAATTCGTCCCTT
TTGAAGTATAGTCTGTTTTGGACAACAGCCAGCCCATATAAATCAGGGCGTTCCGATACAAAAATCCAGGAGGGTTCAATTGG

GACATCGTGCCGCCATTTACTGCCGGGTTTCAACAGCGGATCAGTCTTGTGAACGCCAGGAATTTGATCTGCGAGCCTTCGCC
GGCCGTGCCGGCTACGACGTGGTGGGAATATTTAAGGAAACAGGTTCAGGAACTAAACTCGACCGGGCCGAGCGAAAGAA
AGTCCTGGCGCTTGCCCAGTCCAGACAAATTGATGCAATCCTGGTCACTGAGCTTTCCCGGTGGGGGCGCTCGACGCTCGAT
CTGCTCAATACGCTACGTGAACTGGAGAACTGGAAGGTTTCCGTGATAGCCATGAATGGAATGGCGTTCGATCTTTCGTCGCC
GTATGGACGAATGCTGGCGACGTTTCTTTCCGGCATTGCGGAGTTTGAGCGGGATCTCATCAGCGAGCGGGTCAAGTCAGG
CCTTGCTGTTGCGAAGGCACGTGGTAAGAGGCTTGGTCGTCAGGCCGGAGTGCGACCAAAATCAGACCGACTTTTGCCTAA
GGTGGTTGCGATGAGGGCCGAGGGACGCAGCTATCGCTGGATCGCACGCGAGCTCGGTATCAGCAAGAATACCGTCGCTGA
CATCGTGCAACGACACAGAGCTAACGCTTAGGGTGTCATTTCGCCCTCAGCCGGAACCGACCCCTGGAAGCGCGCCAGCGT
GAAGCGGACCAGGCCCGGGGGCGCAGCGAAGACGATGGCCGCCGGCCTCGCCATCCGGATGGCTCGGACAAGGGCGGTG
GCTCGTACAAACGCGAGTTTGGTGTGCCGGATGACCGTGATCAGGAAAGCTTCACCGATCCGGACAGCCGGATCATGAACAC
GCCGGTGGTGGCTCCGAGCAGAGCTACAACGGGTACACAGCGGTCGATGCCGAGCACCAGATCATCGTGGCGGCGGAGTT
GACCAACTGCGCCGCGGACAGTCAGGCGCTGCTGGGCATGCTGGCAGCAGTTCAGGCCAACACCGGAGAAATGCCGGCCC
AGACGCTGGCGGATGCGGGATTCCGTAGTGAGGCTGTTCTGGCAAAGGTCGCCGATCACCACGGCGATGTCATCGTTGCCCT
CGGCCGCGAGGGACGTGAAGATGCCAAGGTCAATGCCAAGACCCATCCGCATACGGCGGCGATTGCGGCGAAATTGAAAAC
GGAGCAAGGCGATGCAGCTTACCGCCGGCGCAAGTCGATCGTGGAGGCTCCGAATGGTTGGATCAAGGCGGTGATGGGAT
TGCGTCAGTTCAGCATGAGGGGCCTGGACAAGGTGCAAGCCGAGTGGAAGCTCGTCTGCATGGCGCTAAATCTGAGGCGAA
TGGCGTATCTGTGAGGGCGAAGGTTAAATGGGGCGGCTCAAATGCACCCCAGTCGTCATAACACGCCGCGCGCCGCAAGATT
GGTATCCTTGGCGCCGACGCCTTGCCAATTGCAGAGAGCGCCGCCGCCATCGTCAGTGCTCTACAGAAAACCGGTCACACGG
CTCTGCCGCGCAGACTCCTAGCCTAAATGTGACAGTGGTTGGTAATCCATGCCGCCCGCAAGGGCGGCGGTGGGCCAATAGA
TGATTTTCAGAGCCTTACTGCCCGTTGACGCCCAATCCCTCGAGCGCGAGTCTAGCCGCAGCGGCGATGACGGCCTCGCTGT
GCTTGTCATCCTTGTTAGGCGCCCGGGTGTAGACGGCCAACACAATAGGTGCGCGCCCAGTGGGCCAGACGACGGCATAGT
CATTTGCCGTGCCATACACTCCGCAGGTTCCGGTTTTGTCTCCGACTGCCCAGTCTGCCGGCACCGCCGCGCGGATGCGGTG
GTTGCCGGTCGTGTTTCCCTTTAGCCAATCAACAAACTGCTGCCGCTGCGGCGCAGCCAGTGCAGAGCCCAGTGTCAGTTTT
TGTAAGCTTTCCGTCACGGCGCGCGGCGATGAGGTATCGCGCGCATCGCCTGGGATGGCGGAGTTCAGCTCCAGCTCCCAGC
GGTCCAGACGGAACGTGGTATCGCCGATAGAGCGCATGAAGGCCGTCAGCCCGGCCGGGCCGCCCAACTCCTTCAGCAACA
AATTGGCGGCGGCGTTATCACTGTATTGCACGGCGGCCGCGGACAGCTCCGCCACCGTCATGCCTGTTGTCAGATATTTTTCC
GAGATGGGTGACCACGGAACCAGCGCATTTTTGCCGTAACGGATGGGTGTGTCCAGCAAGCCGGCCTGCTGCTGGCTGCGA
GCCAGCACAGCGGCAGCAAGAAAGCCCTTGAATGAGCTGCACAGTGGGAAGCGCTCCTCAGCGCGGTAACTTACAGTTGC
GCCTGAGCCGGTATCCATCGCGTACACACCGATGGAGCCGCCAAAGTCCTGTTCGAGTTTAGCGAATGGTTCCGCGACGAGG
TTGGTCAGCGCGGTGGCAGAAAAGCCAGCCAGCGGCCATGAGAGACAAGACAGCAGAACTAGACGGCGATACAGTGACAT
CAACGATATTCCTTGTTTGAAGGTGGAGTTACGGACGGCCTCAGGAAGTCCTGGCCAAGCCCCGACTATTGGGGCGCGAAG
ATAGCACCGAGGGTACGCCGGTGTCAACACGGGGTCTGACGCTCAGTGGAACGAAAACTCACGTTAAGGGATTTTGGTCAT
GAGATTATCAAAAAGGATCTTCACCTAGATCCTTTTAAATTAAAAATGAAGTTTTAAATCAATCTAAAGTATATATGAGTAAACTT
GGTCTGACAGTTACCAATGCTTAATCAGTGAGGCACCTATCTCAGCGATCTGTCTATTTCGTTCATCCATAGTTGCCTGACTCCC
CGTCGTGTAGATAACTACGATACGGGAGGGCTTACCATCTGGCCCCAGTGCTGCAATGATACCGCGAGACCCACGCTCACCGG
CTCCAGATTTATCAGCAATAAACCAGCCAGCCGGAAGGGCCGAGCGCAGAAGTGGTCCTGCAACTTTATCCGCCTCCATCCA
GTCTATTAATTGTTGCCGGGAAGCTAGAGTAAGTAGTTCGCCAGTTAATAGTTTGCGCAACGTTGTTGCCATTGCTGCAGGCAT
CGTGGTGTCACGCTCGTCGTTTGGTATGGCTTCATTCAGCTCCGGTTCCCAACGATCAAGGCGAGTTACATGATCCCCCATGTT
GTGCAAAAAAGCGGTTAGCTCCTTCGGTCCTCCGATCGTTGTCAGAAGTAAGTTGGCAGCAGTGTTATCACTCATGGTTATGG
CAGCACTGCATAATTCTCTTACTGTCATGCCATCCGTAAGATGCTTTTCTGTGACTGGTGAGTACTCAACCAAGTCATTCTGAGA
ATAGTGTATGCGGCGACCGAGTTGCTCTTGCCCGGCGTCAACACGGGATAATACCGCACCACATAGCAGAACTTTAAAAGTGC
TCATCATTGGAAAACGTTCTTCGGGGCGAAAACTCTCAAGGATCTTACCGCTGTTGAGATCCAGTTCGATGTAACCCACTCGT
GCACCCAACTGATCTTCAGCATCTTTTACTTTCACCAGCGTTTCTGGGTGAGCAAAAACAGGAAGGCAAAATGCCGCAAAAA
AGGGAATAAGGGCGACACGAAAATGTTGAATACTCATACTCTTCCTTTTTCAATATTATTGAAGCATTTACCAGGGTTATTGTCT
CATGAGCGGATACATATTTGAATGTATTTAGAAAAATAAACAAATAGGGGTTCCGCGCACATTTCCCCGAAAAGTGCCACCTG
ACGTCTAAGAAACCATTATTATCATGACATTAACCTATAAAAATAGGCGTATCACGAGGCCCTTTCGTCTTCAAGAATTTATAA

ACCGTGGAGCGGGCAATACTGAGCTGATGAGCAATTTCCGTTGCACCAGTGCCCTTCTGATGAAGCGTCAGCACGACGTTCC
TGTCCACGGTACGCCTGCGGCCAAATTTGATTCCTTTCAGCTTTGCTTCCTGTCGGCCCTCATTCGTGCGTTCTAGGATCCTCC
GGCGTTCAGCCTGTGCCACAGCCGACAGGATGGTGACCACCATTTGCCCCATATCACCGTCGGTACGGCACTGTTGCAAAGT
TAGCGATGAGGCAGCCTTTTGTCTTATTCAAAGGCCTTACATTTCAAAAACTCTGCTTACCAGGCGCATTTCGCCCAGGGGAT
CACCATAATAAAATGCTGAGGCCTGGCCTTTGCGTAGTGCACGCATCACCTCAATACCTTTGATGGTGGCGTAAGCCGTCTTCA
TGGATTTAAATCCCAGCGTGGCGCCGATTATCCGTTTCAGTTTGCCATGATCGCATTCAATCACGTTGTTCCGGTACTTAATCTG
TCGGTGTTCAACGTCAGACGGGCACCGGCCTTCGCGTTTGAGCAGAGCAAGCGCGCGACCATAGGCGGGCGCTTTATCCGT
GTTGATGAATCGCGGGATCTGCCACTTCTTCACGTTGTTGAGGATTTTACCCAGAAACCGGTATGCAGCTTTGCTGTTACGAC
GGGAGGAGAGATAAAAATCGACAGTGCGGCCCCGGCTGTCGACGGCCCGGTACAGATACGCCCAGCGGCCATTGACCTTCA
CGTAGGTTTCATCCATGTGCCACGGGCAAAGATCGGAAGGGTTACGCCAGTACCAGCGCAGCCGTTTTTCCATTTCAGGCGC
ATAACGCTGAACCCAGCGGTAAATCGTGGAGTGATCGACATTCACTCCGCGTTCAGCCAGCATCTCCTGCAGCTCACGGTAAC
TGATGCCGTATTTGCAGTACCAGCGTACGGCCCACAGAATGATGTCACGCTGAAAATGCCGGCCTTTGAATGGGTTCATGTGC
AGCTCCATCAGCAAAAGGGGATGATAAGTTTATCACCACCGACTATTTGCAACAGTGCCTTATTTCGAGCATTTTCTTGCGAAA
AAGCCGACCGGGAAAATTGACATGCGTACCATTCGCCTGGGGTGTAATAAGGCGATAGACACCGGTACCTACACATTTACCTT
CGCGGACAAATCAACGGTTTCAGCCCGCTACACATTCACTTATGCATGGGACGGTAAAGAGTGGAAAATTTCCACACACCACT
CTTCAGCGATGCCTGAAGGGTAAAAAGCTGTGCAGGGGGCGGAATATTCACCGCTCCCTGAAGACTAGCCAATAATCGCGAT
GCCAAGACGTTCCATGAGCAACGATGCCTGGTAGTTGTCCAACTTAACGCCTTGTAAATCAACGCGCCGAATATCTAAGTCAC
CCAACTCCGAATTGGTCAGATCGCAATGTGTGAAGTTTGCTGCTCGCCAGTCGAAAGTCGAAAACTCGCCGCCGGAGAGATC
TGAACCACTGAACGTCGCGCCCAGTACCTGGGCCCCCATCCAACGGTTTTCCCACAGCTCACACTTTTCCAACACGACTTTCG
AAAAATTGGCGTAGCTTAGATTTGTGTTAGTGATATATGCACTGCAAAACCAGGTGCGAGTAGTGATCATATTCATAAAACTTG
CGCCGCGAAAATCTGCGCCTTGCGCACGACAGTGGCGAATTTCAATGCCAAGCGCACTGGCATTGCGAAAATCCGCCATGGA
TAAATCACAGCTTTTAAAAATGGCATCTTTCAGCATCGCACGACTAAAATTGCACCCTTTCTGGCTTTCACGATCATAGAACTG
ACAGCCGATAAATTCAGTGCCGCTCAGGTCGGCACCTGAAAAATCACAGTTAAAAAATGTACTATTTTCAATTTTCTCACCGGT
GAAGCGGTTTCTGTTAATTTTTTCGCCAACGAGTGCCAGAGCCATATTTTGTGCCTGTTTTTTTATACAGTAATGGCGTCATGG
TAAACCCTGATGAGGTTATGCGTCAAATCCGCCAATATAACATCTGCAAATGTGCGTTAAATCTGGTGTTTTTTCAGCAAAGCG
CGAAGCTGATGGTAAGTCAGACCCAGTAATTCAGCGGCTTTTTCTGGTTAAATTTTGCCTGCTGTAAGCTGGTTTGTAGAAAG
TCTTTCTCTTGCTGCTGCTGGAATTCACGCAGATCCAGCGGTAACCCTACAGACATCGGTTTAGTTTCCGGCGCCTGCGGCTG
CGTCTGGTTCTGAAATCCATCCCTGTCGGTGTTGCTTATGCAGTCTGGTCGGGACTCGGCGTCGTCATAATTACAGCCATTGCC
TGGTTGCTTCATGGGCAAAAGCTTGATGCGTGGGGCTTTGTAGGTATGGGGCTCATAATTGCTGCCTTTTTGCTCGCCCGATC
CCCATCGTGGAAGTCGCTGCGGAGGCCGACGCCATGGTGACGGTGTTCGGCATTCTGAATCTCACCGAGGACTCCTTCTTCG
ATGAGAGCCGGCGGCTAGACCCCGCCGGCGCTGTCACCGCGGCGATCGAAATGCTGCGAGTCGGATCAGACGTCGTGGATG
TCGGACCGGCCGCCAGCCATCCGGACGCGAGGCCTGTATCGCCGGCCGATGAGATCAGACGTATTGCGCCGCTCTTAGACGC
CCTGTCCGATCAGATGCACCGTGTTTCAATCGACAGCTTCCAACCGGAAACCCAGCGCTATGCGCTCAAGCGCGGCGTGGGC
TACCTGAACGATATCCAAGGATTTCCTGACCCTGCGCTCTATCCCGATATTGCTGAGGCGGACTGCAGGCTGGTGGTTATGCAC
TCAGCGCAGCGGGATGGCATCGCCACCCGCACCGGTCACCTTCGACCCGAAGACGCGCTCGACGAGATTGTGCGGTTCTTC
GAGGCGCGGGTTTCCGCCTTGCGACGGAGCGGGGTCGCTGCCGACCGGCTCATCCTCGATCCGGGGATGGGATTTTTCTTG
AGCCCCGCACCGGAAACATCGCTGCACGTGCTGTCGAACCTTCAAAAGCTGAAGTCGGCGTTGGGGCTTCCGCTATTGGTCT
CGGTGTCGCGGAAATCCTTCTTGGGCGCCACCGTTGGCCTTCCTGTAAAGGATCTGGGTCCAGCGAGCCTTGCGGCGGAACT
TCACGCGATCGGCAATGGCGCTGACTACGTCCGCACCCACGCGCCTGGAGATCTGCGAAGCGCAATCACCTTCTCGGAAACC
CTCGCGAAATTTCGCAGTCGCGACGCCAGAGACCGAGGGTTAGATCATGCCTAGCATTCACCTTCCGGCCGCCCGCTAAATAT
CTCCTTTTGGGTTGTTAATAAAACATCCAATAAGTTGACTGTGCGTGAAAAAGAAAGTTTTGTGTGATGGCGTTGAAGATCGC
ACCGTTAAGCTCTTATGTGGGATGGTGCAGAGCTCGACGACTACCGATAAAACGCAACCGCCGCAAACAGACAAGAAAAAG
CCCCAACTGATAACAGTTGGGGCTTCAGTATTGTGATTGGTGGAGCAATAGCACCCTGAACCCAAAACCTTCTCGCTCAACCG
GTAGTGGCTGATAACAACTCGTGAGGGCTATTGCGGGTTAAGCATTTAGCGATGTCTAGGGCCAGACTGGACGTCTGAACGC
AAGCCGCTGATACTGTACATAACCACAGTATCAGCGGAGGATACCCATGTCGCTGGCAAGGAACGCCACGGCGAGTCAATCG
CCCACTCAAACAAACGGTTACGAACGCCACCAACCCGACCAGACGCTGCTCTACCAGCTGGTTGAGCAGCACTACCCAGCCT

TCAAAGCCTCACTCGAAGCCCAAGGTCAACACCTGCCTCGCTACATCCAACAAGAATTCAACGACCTCCTCCAATGTGGCCGT
CTGGAGTATGGTTTCATGCGGGTTCGCTGCGAGGATTGTCATCACGAGCGTCTGGTCGCCTTCAGCTGTAAACGACGCGGCT
TTTGCCCTAGCTGCGGTGCCCGCCGGATGGCCGAGAGTGCGGCGCTGCTGATAGACGAAGTCTTCCCCAAGGAGCCCATTC
GCCAGTGGGTGCTCAGCTTTCCTTTCCAGCTACGCTTTTTGCTGGCTCGCCATCCCCAGCTGATGGGCCAGGTCTTGAGTATC
GTCTATCGTACACTCTCAACTCATCTGATCAAAAAAGCCGGTTACACCAAAGCCTCTGCACAAACTGGCTCAGTGACTCTTATC
CAACGCTTTGGCTCCGCGCTAAATCTCAATGTCCACTACCACATGCTGTTTCTCGATGGTGTCTATGCCGAAGATGACTATGGC
AAGCAACGCTTCCATCGTGTCAAGGCACCCACTTACGATGAGCTGAATACGCTCGCTCACACCCTCAGCCATCGCATCGCTCG
CTGCATGGAAAAGCGTGGGATTTTGGAGCGTGATGCCGAGAATACGTGGTTGACACTGGAAGAGGGCGAAGACGATACGCT
GACTCAATTACATGGTGCTTCGGTTACGTATCGCATTGCCGTCGGCCCCCAGCAAGGGCGCAAAGTCTTCACCCTGCAAACCT
TGCCAGGGCGTGAGGATAAAGCCGACTCAAGCAGTCGAGTAGCCAACCATGCTGGTTTCTCGCTACACGCCGGTGTGATGGC
CGAAGCGCATCAGCGGGATAAGCTTGAGCGCTTGTGTCGCTACATTAGTCGGCCAGCGGTTTCAGAAAAACGTCTGGCATTA
ACCGCCAATGGGCAGGTGCGTTACGAGCTCAAAACTCCGTACCGCAATGGCACCACCCATGTGATCTTCGAGCCGCTGGACT
TCATCGCCAAACTCGCTGCGTTGGTACCTAAGCCGCGAGTCAACCTCACACGCTTCCACGGCGTCTTTGCACCGAACAGCAA
ACACCGAGTTCAAGTAACACCCGCCAAGCGGGGCAAGAAGCCCGACAAATCGGAAGGTCTCGATACTAACTGGCGTGACAA
GAGTCCTGCAGAGCGCCACCGCGCCATGACCTGGATGCAACGCCTCAAGCGAGTCTTCAATATTGATATTGAAGTCTGCGAAC
ACTGCGGCGGTCACGTCAAAGTGATTGCCAGCATCGAAGATCCGAAGGTCATTGAGCAGATTCTCAAGCATCTGAAACAGAA
AACAGCCAAGGCGAATGCCGCCAAGCAGCGTGAGCTGCCACCAGAACGAGCGCCGCCACTGACTCCCAGCCTGTTCGATCC
ATCACAGAGTCGTCTCTTTGACTGACGACCCCAAATCCAACACTGCTCAACACTGCCAACTTTTAAACGGGGCGGTGGGGCA
GTTTGTATCTCTCGAGCTATCAGGCTAGAGATTTTACCGCCAAATCGAACCTTATTAGAGCGGTTTAGGCTGGACCGGCAGTTA
AAATTGGGGCTTGAGCGGTAAACGAGTGAGGGAATTTCAGGTAAGATACTTCGGATGAGGAGCAAAAAGGTGGTTTATACT
TCCTATACCCAGTAGTGTTCCCCTGTCGGTTTGGTTGGGGTCGATGCCCCACTTTGGGCGCGGCGTTGGGCGCCGCAAGATG
AAAGCTCTGTGTGAAGGCTTGGATATTAAGACCTTGGATGATTTCAATGCCATCACCGAAGAGCAGATCGTTAGCGTCGACAA
ATTCAGCTACAAAACTGCCGCAAAAGTGCTTGCAGGTATGGCTGAACATGCCGAGCTATTCAACAAGATTCAATCAATCATCG
GGTTCAAACAGCAGGTCACTTCGGGGGATCGTTTTACCGGTGAGAAGATCGTGATCACCGGTTTTCGTGACGCGGCCTTGG
AGAAGCTGATTGAAGCTGAGGGTGGTGAAGTGCAATCATCAGTCTCGTCGAAGACCACCATGGTGATCGCGGCATCCACTTC
CGGATCTTCCGGCAAATTGAAGAAAGTGCACGATCTCAACAACAGCGGAAAGGCAAATATCAAACTGATTGATCTGGCCACC
TTCCGTAAGCAATATCTGGAACAACCAGCATCGACTGGTTTGGAGTTTTAATGAGTCACCCACAACTTGAGCTAATAGTCGCT
GTGGATTCTAAGTTGGGATTCGGGAAAGGCGGCAAGATTCCATGGAAATGCAAAGAAGACATGGCGCGATTTACGCGGATT
TCTAAAGAGATCCGCGTGTGCGTTATGGGGAAACACACGTATACTGACATGCGTGACATGCAGTTAGAAAAGGATGGCGCCG
AGGAGCGAATCAAGGAGAAAGGAATTCTCCCCGAACGCGAATCGTTCGTGATCTCCTCGACGTTAAAACAAGAAGATGTCAT
AGGCGCTACTGTCGTTCCTGATCTTCGTGCTGTGATCAACCTGTATGAGAATACCGATCAACGCATTGCTGTCATTGGTGGGGA
GAAGTTGTACATTCAAGCTCTTTCATCAGCAACGAAACTGCACATGACCATAATTCCAAGAGAGTTCGACTGTGATCGATTTAT
TCCTGTTGATCCGATCCAGAACAATTTTCACATTGATTCCAGTGCCAGCGAGACTGTGGAGGCAACCGTTGATGAGACTCAAG
AGCGCATTCACTTTGCTACTTACGTGCGTAACAATCAGTAACGCGCTGGCAGTGGAAGAACAAGAACCAGAAACGAAGCAG
ACGGTGTTGGCCGTCTGCTTCGAAACATCATTATTGGCTCGAGAACTGATCGAGAATACCATTCTTGCGGACGACATCCAAAA
CAATACAGAAAAGATAGCTTTGCAGTTGTCGGAGCCCCTCCCCCAACTTATAATGGGATTATTGCTGGGCTAAACAATGCTGTC
GCCATGCGTGGGACAGCAAACGTTGAATCGCCATCAAGAGCATCCACAGATTTACGTGCAGCCGTCATCGGTTTATGGTGTAT
AAATGACACCATAGCCGCCCTGAATCAGACTGATAATGCAGTCTTACAGGACGGCGAGTTGCAACAAACACTTGTCGATTATT
ACAACAACTTATTGGCTGACATCGTTTGTCGTTTTCAGAAGACGGCTGCACTGAACGTCAGAAGCCGACTGCACTATAGCAGC
GGAGGGGTTGGATCCATCAGGCAACGACGGGCTGCTGCCGGCCATCAGCGGACGCAGGGAGGACTTTCCGCAACCGGCCG
TTCGATGCGGCACCGATGGCCTTCGCGCAGGGGTAGTGAATCCGCCAGGATTGACTTGCGCTGCCCTACCTCTCACTAGTGA
GGGGCGGCAGCGCATCAAGCGGTGAGCGCACTCCGGCACCGCCAACTTTCAGCACATGCGTGTAAATCATCGTCGTAGAGA
CGTCGGAATGGCCGAGCAGATCCTGCACGGTTCGAATGTCGTAACCGCTGCGGAGCAAGGCCGTCGCGAACGAGTGGCGG
AGGGTGTGCGGTGTGGCGGGCTTCGTGATGCCTGCTTGTTCTACGGCACGTTTGAAGGCGCGCTGAAAGGTCTGGTCATAC
ATGTGATGGCGACGCACGACACCGCTCCGTGGATCGGTCGAATGCGTGTGCTGCGCAAAAACCCAGAACCACGGCCAGGAA
TGCCCGGCGCGCGGATACTTCCGCTCAAGGGCGTCGGGAAGCGCAACGCCGCTGCGGCCCTCGGCCTGGTCCTTCAGCCAC

CATGCCCGTGCACGCGACAGCTGCTCGCGCAGGCTGGGTGCCAAGCTCTCGGGTAACATCAGGCCCGATCCTTGGAGCCCTT
GCCCTCCCGCACGATGATCGTGCCGTGATCGAAATCCAGATCCTTGACCCGCAGTTGCAAACCCTCACTGATCCGCATGCCCG
TTCCATACAGAAGCTGGGCGAACAAACGATGCTCGCCTTCCAGAAAACCGAGGATGCGAACCACTTCATCCGGGGTCAGCAC
CACCGGCAAGCGCCGCGACGGCCGAGGTCTTCCGATCTCCTGAAGCCAGGGCAGATCCGTGCACAGCACCTTGCCGTAGAA
GAACAGCAAGGCCGCCAATGCCTGACGATGCGTGGAGACCGAAACCTTGCGCTCGTTCGCCAGCCAGGACAGAAATGCCTC
GACTTCGCTGCTGCCCAAGGTTGCCGGGTGACGCACACCGTGGAAACGGATGAAGGCACGAACCCAGTGGACATAAGCCTG
TTCGGTTCGTAAGCTGTAATGCAAGTAGCGTATGCGCTCACGCAACTGGTCCAGAACCTTGACCGAACGCAGCGGTGGTAAC
GGCGCAGTGGCGGTTTTCATGGCTTGTTATGACTGTTTTTTTGTACAGTCTATGCCTCGGGCATCAAGGCGAGCTCAGAGACC
ATGGAAAGCATGTTCTCGGACTTACGTAGCAACTCGTTTCTTTTCGCAGGTTGAGCCACCTCCGCGCTTCATCAGAAAACTGA
AGGAACCTCCATTGAATCGAACTAATATTTTTTTTGGTGAATCGCATTCTGACTGGTTGCCTGTCAGAGGCGGAGAATCTGGT
GATTTTGTTTTTCGACGTGGTGACGGGCATGCCTTCGCGAAAATCGCACCTGCTTCCCGCCGCGGTGAGCTCGCTGGAGAGC
GTGACCGCCTCATTTGGCTCAAAGGTCGAGGTGTGGCTTGCCCCGAGGTGATCAACTGGCAGGAGGAACAGGAGGGTGCA
TGCTTGGTGATAACGGCAATTCCGGGAGTACCGGCGGCTGATCTGTCTGGAGCGGATTTGCTCAAAGCGTGGCCGTCAATGG
GGCAGCAACTTGGCGCTGTTCACAGCCTATCGGTTGATCAATGTCCGTTTGAGCGCAGGCTGTCGCGAATGTTCGGACGCGC
CGTTGATGTGGTGTCCCGCAATGCCGTCAATCCCGACTTCTTACCGGACGAGGACAAGAGTACGCCGCAGCTCGATCTTTTGG
CTCGTGTCGAACGAGAGCTACCGGTGCGGCTCGACCAAGAGCGCACCGATATGGTTGTTTGCCATGGTGATCCCTGCATGCC
GAACTTCATGGTGGACCCTAAAACTCTTCAATGCACGGGTCTGATCGACCTTGGGCGGCTCGGAACAGCAGATCGCTATGCC
GATTTGGCACTCATGATTGCTAACGCCGAAGAGAACTGGGCAGCGCCAGATGAAGCAGAGCGCGCCTTCGCTGTCCTATTCA
ATGTATTGGGGATCGAAGCCCCCGACCGCGAACGCCTTGCCTTCTATCTGCGATTGGACCCTCTGACTTGGGGTTGATGTTCA
TGCCGCCTGTTTTTCCTGCTCATTGGCACGTTTCGCAACCTGTTCTCATTGCGGACACCTTTTCCAGCCTCGTTTGGAAAGTTT
CATTGCCAGACGGGACTCCTGCAATCGTCAAGGGATTGAAACCTATAGAAGACATTGCTGATGAACTGCGCGGGGCCGACTA
TCTGGTATGGCGCAATGGGAGGGGAGCAGTCCGGTTGCTCGGTCGTGAGAACAATCTGATGTTGCTCGAATATGCCGGGGA
GCGAATGCTCTCTCACATCGTTGCCGAGCACGGCGACTACCAGGCGACCGAAATTGCAGCGGAACTAATGGCGAAGCTGTAT
GCCGCATCTGAGGAACCCCTGCCTTCTGCCCTTCTCCCGATCCGGGATCGCTTTGCAGCTTTGTTTCAGCGGGCGCGCGATGA
TCAAAACGCAGGTTGTCAAACTGACTACGTCCACGCGGCGATTATAGCCGATCAAATGATGAGCAATGCCTCGGAACTGCGT
GGGCTACATGGCGATCTGCATCATGAAAACATCATGTTCTCCAGTCGCGGCTGGCTGGTGATAGATCCCGTCGGTCTGGTCGG
TGAAGTGGGCTTTGGCGCCGCCAATATGTTCTACGATCCGGCTGACAGAGACGACCTTTGTCTCGATCCTAGACGCATTGCAC
AGATGGCGGACGCATTCTCTCGTGCGCTGGACGTCGATCCGCGTCGCCTGCTCGACCAGGCGTACGCTTATGGGTGCCTTTCC
GCAGCTTGGAACGCGGATGGAGAAGAGGAGCAACGCGATCTGGCTATCGCGGCCGCGATCAAGCAGGTGCGACAGACGTC
ATACTAGGGGTCGTCTCAGAATTCGGAAAATAAAGCACGCTAAGGCGTAGTCACCCCGTGACTCCCCGCGCCGATGCAGCGA
GCTTCGTTCCGTCTTGCAGTGACGCAATCAGCGGGCAGGAAACGTTCCCTTTCCGCGCATGGCAGGCGCACACCAGTTCAGA
CAGCACGGCCTCCATGCGTGCCAAGTCGGCCATCTTCTCGCGCACATCCTTGAGCTTGTGCTCGGCCAGGCCGCTGGCTTCCT
CGCAATGGGTGCCATCCTCCAGCCGCAGTAGCTCGGCGATTTCGTCCAGGCTAAAGCCCAGCCGCTGGGCCGATTTCACGAA
CCGCACTCGTGTTACATCCGCCTCGCCATAGCGGCGAATGCTGCCATAGGGCTTGTCTGGCTCCGGCAGCAGGCCCTTGCGCT
GGTAGAACCGGATGGTCTCCACATTGACCCCGGCCGCCTTGGCAAAAACGCCAATGGTCAGATTCTCAAAATTAATTTGCATA
TCGCTTGACTCCGTACATAACTACGGAAGTAAGCTTAAGCTATCCAAACCAAATTTGAAAGGACAAGCGTATGTCTGAACCAC
AAAAGTCTGAACCACAAAAGTCTGAACCACAAAACGGGCGCGGCGCGCTCTTCGCCGGTGGGCTGGCCGCCATTCTTGCGT
CGGCCTGCTGCTGGGGCCGCTGGTTTTGATCGCCTTGGGGTTCAGCGGGGCATGGATCGGCAACCTGACGGTGCTGGAACC
CTATCGCCCGATCTTCATCGGCGCAGCGCTGGTCGCGCTGTTTTTCGCCTGGCGGCGCATCTACCGCCCGGCGCAAGCCTGCA
AACCGGGTGAGGTCTGCGCGATTCCCCAAGTGCGAGCTACTTACAAGCTCATTTTCTGGATCGTGGCCGCGCTGGTCCTGGT
CTCGCTCGGATTTCCCTACGTCATGCCATTTTTCTATTAATCACAGGAGTTCATCATGAAAAAACTGTTTGCCGCCCTCGCCCTC
GCTGCCGTTGTTGCCCCCGTGTGGGCCGCCACCCAGACCGTCACGCTGTCCGTGCCTGGCATGACCTGCGCCTCTTGCCCGAT
CACTGTCAAGCACGCGCTTTCCAAGGTTGAGGGCGTGAGCAAGACCGACGTAAGTTTCGACAAGCGCCAGGCCGTCGTCAC
CTTCGACGATGCCAAGACCAACGTCCAGAAGTTGACCAAGGCGACCGAGGACGCGGGCTATCCGTCCAGCCTCAAACGCTG
ATCCGTTAACCGAACTCGGGAGCGACACATGGGACTCATCACGCGCATCGCTGGCAAAACCGGCGCGCTCGGCAGCGTCGT
TTCCGCGATGGGCTGCGCCGCCTGTTTTCCTGCCATCGCCAGCTTTGGCGCGGCCATCGGACTGGGCTTCTTGAGCCAGTAC

GAGGGGCTATTCATTGGCATCCTGCTGCCGATGTTCGCCGGCATCGCGTTACTCGCCAATGCTATCGCTTGGCTCAATCATCGA
CAGTGGCGACGCACGGCGCTCGGCACGATAGGCCCGATCTTGGTGCTGGCAGCGGTGTTTTTAATGCGGGCTTACGGCTGG
CAGAGCGGTGGACTGCTCTATGTCGGCCTGGCCTTGATGGTTGGGGTGTCGGTCTGGGATTTCATCTCGCCAGCACATCGCC
GCTGCGGGCCGGACAGCTGTGAATTGCCAGAACAACGTGGCTGACGGCAACAGCCGTAGCCACCACAGAAAAGGAAAAAT
ACATGACCACCCTGAAAATCACCGGGATGACCTGCGACTCGTGCGCGGCTCACGTCAAGGAAGCCTTGGAGAAAGTGCCCG
GCGTGCAATCGGCGCTGGTGTCCTATCCGAAGGGCACAGCGCAACTCGCCATTGAGGCGGGCACGTCATCGGATGCGCTGA
CTACCGCCGTGGCCGGACTGGGCTACGAGGCAACGCTTGCCGATGCGCCACCGACGGACAACCGCGCCGGCCTGCTCGACA
AGATGCGCGGCTGGATAGGGGCCGCTGATAAGCCAGTGGCAACGAACGCCCGTTGCAGGTCGTCGTCATTGGTAGCGGTGG
AGCCGCGATGGCGGCAGCACTGAAGGCCGTCGAGCAAGGCGCGCAGGTCACGCTGATTGAGCGCGGCACCATCGGCGGCA
CCTGCGTCAACGTCGGTTGTGTGCCGTCCAAGATCATGATCCGCGCCGCCCACATCGCCCATCTGCGCCGGAAAGCCCATTCG
ACGGCGGCATGCCACCCACACCGCCGACGATCTTGCGCGAGCGGCTGCTGGCCCAGCAGCAGGCCCGTGTCGAAGAACTCC
GTCATGCCAAGTACGAAGGCATCCTGGACGGCAATTCAGCCATCACCGTTCTGCACGGTGAAGCGCGTTTCAAGGACGACCA
GAGCCTTATCGTTAGTTTGAACGAGGGTGGCGAGCGCGTCGTGATGTTCGACCGCTGCCTGGTCGCCACGGGTGCCAGCCC
GGCGGTCCCGCCGATTCCGGGCTTGAAAGAGTCACCCTACTGGACTTCCACCGAGGCCCTGGCGAGCGACACCATTCCCGA
ACGCCTTGCCGTAATCGGCTCGTCGGTGGTGGCGCTGGAGCTGGCGCAAGCCTTTGCCCGGCTGGGCAGCAAGGTCACGGC
CCTGGCGCGCAATACCTTGTTCTTCCGTGAAGACCCGGCCATCGGCGAGGCGGTGACAGCCGCTTTCCGTGCCGAGGGCATC
GAGGTGCTGGAGCACACGCAAGCCAGCCAGGTCGCCCATATGGACGGTGAATTCGTGCTGACCACCACGCACGGTGAATTG
CGCGCCGACAAGCTGCTGGTCGCCACCGGCCGGACACCGAACACGCGCAGCCTGGCATTGGAAGCGGCGGGGGTAGCCGT
CAATGCGCAGGGGGCCATCGTCATCGACAAGGGCATGCGCACCAGTAGCCCGAACATCTACGCGGCCGGCGACTGCACCGA
CCAGCCGCAGTTCGTCTATGTGGCGGCAGCGGCCGGCACTCGTGCGGCGATCAACATGACTGGCGGCGATGCGGCCCTGGA
CCTGACCGCAATGCCGGCCGTGGTGTTCACCGACCCGCAGGTCGCCACCGTGGGCTACAGCGAGGCGGAAGCACATCACGA
CGGGATCGAGACCGACAGTCGCCTGCTAACACTGGATAACGTGCCGCGTGCGCTTGCCAACTTCGACACACGCGGCTTCATC
AAGCTGGTCATCGAGGAAGGTAGCGGACGGCTCATCGGCGTGCAAGCGGTGGCCCCGGAAGCGGGTGAACTGATCCAGAC
GGCGGTGCTCGCCATTCGCAACCGTATGACCGTGCAGGAACTGGCCGACCAATTGTTCCCCTACCTGACCATGGTCGAAGGG
CTGAAGCTCGCGGCGCAGACCTTCAGCAAGGACGTGAAGCAGCTTTCGTGCTGCGCCGGATGAGGAAAGGAGGTGTTCAA
TGAGCGCCTACACAGTGTCCCGGCTGGCCCTTGATGCCGGGGTGAGCGTGCATATCGTGCGCGACTACCTGCTGCGCGGATT
GCTACGGCCGGTCGCGTACACCACGGGCGGCTACGGCTTGTTCGATGACACCGCGTTGCAACGGCTGCGCTTTGTACGGGC
TGCCTTCGAAGCGGGTATCGGCCTGGACGCACTGGCGCGGCTGTGCCGGGCGCTGGATGCTGCGGACGGTGACGGTGCGT
CTGCGCAGCTTGCCGTGTTGCGGCAACTCGTCGAGCGTCGGCGCGGGGCCCTGGCCAGCCTCGAAATGCAACTGGCCGCCA
TGCCAACCGAACCGGCACAGCACGCGGAGAGTCTGCCATGAACAGCCCAGAGCACTTGCCGTCTGAGACGCACAAACCGAT
CACCGGCTACTTGTGGGGCGCGCTGGCCGTGCTCACCTGTCCCTGCCATTTGCCGATTCTCGCCATTGTGCTAGCCGGCACGA
CGGCCGGCGCGTTCATCGGGGAGCACTGGGGTATTGCAGCCCTCACGCTGACCGGCTTGTTTGTCCTGTCTGTGACGCGGCT
GCTGCGGGCCTTCAAGGGAAGATCATGACCGCTTCCCAGCCAGCCGAGAGTGGGCAGCTTTGAGCTTCGCTACCAATCTGG
AGGAGTACCACCATGAACGCAAACGCCCCGAACACTGCCAGTTGCACCACCTGCTGCGTATGCTGCAAAAGAAATTCCGCTC
GATGCCGCCTTCACCCCGGAAGGCGCGGAATACGTCGAACATTTCTGCGGGCTGGATTGCTATGAACGCTTCCAGGCACGCG
CCAAGGCCGCGACAGAATCTGACATTGCGCCTGTCCCTGGCGGTTCGCAGCCGTCAGATTGAGGCATACCCTAACTTGATGTC
AGATGCCATGTGCAAACGATGTCAGAATAGAGTTAAATTTCCTATTGATTGACATATTCCGTCAAAGGTAATAGATTTCATCCTG
ACACTTTTGCCTTTGGAGGCATCTTGCAAGGTCAACGCATCGGCTATGTCCGCGTCAGCAGCTTCGACCAGAACCCGGAACG
GCAATTGGAGGTGTTCAGGTGGCGCGGGTGTTCACCGACAAGGCTTCTGGCAAGGACACCCAGCGTCCCGAGCTGGAAAG
GCTGCTGGCCTTCGTCCGCGAGGGCGACACCGTGGTGGTGCATAGCATGGACAGGCTGGCACGCAACCTTGATGACCTGCG
CCGCATCGTCAAGGGCTGACACAACGGGGCGTGCGCATGGAGTTCGTCAAAGAAGGGCTGAAGTTCACCGGCGAGGACTC
ACCGATGGCCAATCTGATGCTGTCGGTCATGGGAGCCTTCGCTGAGTTCGAGCGCGCCTGATCCGCGAACGTCAGCGCGAG
GGAATCGTGCTGGCCAAGCAGCGCGGTGCCTACCGGGGACGAAAGAAATCGCTGAACAGCGAACAAATTGCCGAGTTGAA
ACGGCGAGTTGCGGCAGGCGACCAAAAAACCTTGGTGGCCCGTGACTTCGGCATCAGCCGCGAAACCTTGTACCAGTACCT
GCGGGAAGACTGACCATGCCACGCCGCTCAATCCTGTCCGCCACCGAGCGCGAAAGCCTGCTGGCACTGCCAGATGCCAAA
GACGAACTGATACGGCACTACACGTTCAACGAAACCGACCTGTCGGTGATCCGTCAGCGTCGCGGCGCCGCGAATCGATTGG

GCTTCGCTGTGCAGCTTTGCTACTTGCGATTCCCTGGCACCTTTTTGGGCGTCGATGAGCCTCCGTTTCCGCCCCTGTTGCGC
ATGGTGGCCGCGCAACTCAAGATGCCAGTGGAAAGTTGGAGCGAGTACGGCCAGCGCGAACAGACACGGCGGGAGCACTT
GGTCGAGCTGCAAACGGTTTTTGGGTTCAAGCCCTTCACCATGAGCCACTATCGGCAAGCCGTGCATACATTGACCGAGCTG
GCCTTGCAGACCGACAAAGGCATCGTGCTGGCGAGCGCACTTGTCGAGAATCTGCGGCGGCAGAGCATTATCCTGCCCGCC
ATGAATGCCATCGAGCGCGCAAGCGCCGAGGCCATCACCCGTGCCAACCGACGCATTTACGCGGCGCTGACCGATTCTTTGT
TATCACCCCACCGTCAGCGCCTGGACGAACTTCTCAAGCGCAAGGACGGCAGTAAAGTGACGTGGCTGGCATGGCTGCGCC
AGTCGCCTGCCAAACCGAACTCTCGCCACATGCTCGAACATATTGAGCGCCTGAAATCCTGGCAAGCACTTGATCTGCCCGCA
GGCATCGAGCGGCAGGTTCACCAGAACCGCCTGCTCAAAATCGCTCGTGAAGGTGGCCAGATGACGCCTGCTGATCTGGCA
AAGTTCGAGGTGCAACGACGCTATGCCACGCTGGTAGCGCTGGCCATCGAAGGCATGGCCACCGTCACCGATGAAATCATCG
ACCTTCACGATCGCATCATCGGCAAGCTGTTCAACGCGGCCAAGAACAAGCATCAGCAGCAGTTCCAGGCTTCCGGCAAGG
CGATCAACGACAAGGTGCGGATGTATGGGCGCATCGGTCAAGCGTTGATTGAGGCCAAGCAAAGCGGCAGCGATCCGTTCG
CCGCCATCGAGGCCGTTATGCCCTGGGACACCTTCGCCGCCAGCGTCACCGAAGCGCAAACATTGGCGCGGCCTGCCGACTT
TGATTTCCTGCACCACATCGGTGAAAGCTATGCCACGCTACGCCGCTACGCGCCGCAGTTCCTGGGCGTGCTCAAATTGCGGG
CTGCGCCCGCCGCCAAAGGTGTGCTCGATGCCATCGACATGCTGCGCGGCATGAACAGCGACAGCGCGCGCAGGTGCCCGC
CGATGCGCCAACCGCATTCATCAAGCCGCGCTGGGCAAAGCTGGTTCTGACCGACGACGGCATCGACCGGCGTTACTACGA
GTTATGCGCCCTGTCGGAGCTGAAGAACGCGCTGCGCTCCGGTGATGTCTGGGTGCAGGGTTCTCGCCAGTTCAAGGACTTC
GACGAATACCTGGTGCCGGTCGAGAAGTTCGCCACTTTGAAGCTGGCCAGCGAATTGCCGCTGGCAGTGGCCACCGACTGC
GACCAATACCTGCATGACCGGTTGGAATTGTTGGAGGCGCAACTCGCCACAGTCAACCGCATGGCTGCGGCCAACGACTTAC
CGGATGCCATCATCACCACCGCGTCAGGCCTGAAGATCACGCCGCTGGACGCGGCGGTACCAGACGCCGCGCAAGCCATGA
TCGACCAGACAGCTATGCTGCTGCCGCACCTCAAAATCACCGAGTTGCTGATGGAGGTCGATGAATGGACGGGCTTCACCCG
CCACTTCACACACCTGAAGACCAGCGACACGGCCAAGGACAAAACCTTGCTGTTGACGACGATCCTGGCCGACGCGATCAA
CCTGGGTCTGACCAAAATGGCCGAGTCCTGCCCTGGCACCACCTACGCCAAGCTGTCTTGGCTGCAAGCACTGGCACATCCG
CGATGAAACCTATTCGACGGCGCTGGCCGAGCTGGTGAATGCGCAGTTTCGGCAACCCTTCGCCGGCAACTGGGGTGACGG
CACCACGTCATCGTCGGACGGCCAGAACTTCAGAACCGGCAGCAAAGCAGAAAGCACTGGTCATATCAACCCGAAGTATGG
AAGCAGTCGGGACGGACTTTCTACACCCATATCTCCGACCAGTACGCGCCCTTCAGTGCCAAGGTGGTCAACGTGGGCATTC
GTGATTCAACTTACCGTGCTTGATGGCCTGCTGTACCACGAGTCGGACTTGCGCATCGAGGAACACTACACCGACACGGCAG
GCTTCACCGATCACGTGTTTGGCTTGATGCATTTGCTGGGATTTCGCTTCGCGCCGCGTATCCGTGACTTGGGCGAAACCAAG
CTATTCATCCCCAAGGGCGATGCCGCCTATGACGCGCTCAAGCCGATGATTAGCAGCGACAGGCTGAACATCAAGCAAATACG
CGCCCATTGGGATGAAATTCTGCGGCTGGCCACCTCCATCAAGCAGCAAGGCACGGTAACGGCTTCGCTGATGCTGCGCAAA
CTCGGCAGCTACCCGCGCCAGAACGGCTTGGCCGTGGCGTTGCGCGAGCTGGGGCGCATCGAGCGCACGCTGTTCATTTTT
GGATTGGCTGCAAAGCGTGGAGCTGCGCCGCCGCGTCCATGCGGGGCTGAATAAGGGCGAGGCGCGCAACGCGCTGGCCA
GGGCGGTCTTCTTCTACCGATTGGGTGAAATCCGCGACCGCAGTTTTGAGCAGCAGCGCTACGGGCCAGCGGCCTCAATCTG
GTGACGGCGGCCATCGTGTTGTGGAACACGGTATATCTGGAGCGTGCCACCAGTGCTTTGCGTGGCAACGGCACGGCGCTG
GACGACACATTGTTGCAATATCTGTCGCCGCTGGGGGTGGGGCACATCAACCTGACCGGCGATTACCTATGGCGCAGCAGCG
CCAAGGTCGGTGCGGGGAAGTTTAGGCCATTGCGACCGCTGCCACCGGCTTAGCGTGCTTTATTTAATGAGATGGTCACTCC
CTCCTTCCGGTACTATGCTGAGGACAGGCTTTCATTCGGAGAACTATCATGGAAAACATTGCGCTCATTGGTATCGATCTGGGT
AAAAACTCTTTCCATATTCATTGCCAGGATCGTCGCGGGAAGGCTGTTTACCGTAAAAAATTTACCCGGCCAAAGTTGATCGA
ATTTTTGGCGACATGCCCCGCTACAACCATCGCAATGGAAGCCTGTGGCGGTTCTCACTTTATGGCACGCAAGTTGGAAGAG
TTGGGGCATTCCCCAAAGCTGATATCACCACAATTTGTCCGCCCGTTCGTTAAAAGCAATAAAAACGACTTTGTCGACGCCGA
AGCTATTTGTGAAGCTGCATCGCGTCCGTCTATGCGTTTTGTGCAGCCCAGAACGGAATCTCAGCAGGCAATGCGGGCTCTGC
ATCGTGTCCGTGAATCCCTGGTTCAGGATAAGGTAAAAACAACCAATCAAATGCATGCTTTTCTGCTGGAATTTGGCATTAGCG
TTCCCCGAGGAGCTGCCGTTATTAGCCGACTGAGTACCATTCTTGAGGATAATAGTTTGCCTCTTTACCTCAGCCAGTTATTGCT
GAAATTACAACAGCATTATCACTATCTTGTTGAGCAGATTAAAGATTTGGAATCCCAGTTGAAACGAAAGTTGGACGAAGATG
AGGTTGGACAGCGCTTGCTGAGCATTCCCTGCGTCGGAACACTGACAGCGAGTACTATTTCAACTGAGATTGGCGACGGGA
AGCAGTACGCCAGCAGCCGTGACTTTGCGGCGGCAACAGGGCTTGTACCTCGGCAGTACAGCACGGGAGGTAGGACGACA
TTGCTGGGAATTAGTAAGCGAGGTAATAAAAAGATCCGAACTTTGTTGGTTCAATGTGCCAGGGTATTCATACAAAAACTGGA

ACACCAGTCTGGCAAATTGGCCGATTGGGTCAGGGATTTACTGTGCCGGAAAAGCAACTTTGTCGTCACTTGTGCTCTGGCA
AACAAGCTGGCCAGAATAGCCTGGGCCCTAACGGCACGACAGCAAACTTATGTAGCATAACGGCAGAAATACACCGGTTTAA
AGAATTACTGATCTGGTTTTGCGAATACTGATATTGATGATACTAACGGCCCACCGGCCTGTTGAGGAACCTGTAAAACGGAA
AGGCTCATTGAAGCCGTATATTTTCTGGAGGTTCATCAGGCGCGGAACTCATCAAGGCGCGGGAATAAAATCCCATTCAGACG
CCGGATAGATTCAAGCAAGCCAACTTGTCGTCAAAATCGGTGTTGCAAAAACGGGAGTGACCATAGATTCCGTTTTCTGAGA
CGACCCCTACTAGATATCAAGCGACTTCTCCTATCCCCTGGGAACACATCAATCTTACCGGAGAATATCGTTGGCCAAAGCCTT
AGCGTAGGATTTCGCCCTCTCCCGCAAACGACCCCTAAAAGCCGTTTCCTCTGTATAAAAGATCAGCTAAATTATGTGTATTGC
ACAATACATATATGTGAGGTTAGCAGTGAATTTGCCTACGCCCGAAACCTACGATGAACTTCAGAGAGCCTACGATTTTTTCAA
TGAGAAGCTATTCAGCAACGAGCTGCCGCCATGCCTGATAACGTTGCAGCGTGAGAAGCGAACGTATGGCTATTGTTCCTTTA
AGCGTTTCGTCGGCCGTGAGAGTGGGTACACGGTAGACGAGATCGCTATGAATCCGGTGTATTTCTCGATCAGAACCATAAAG
GCCACGCTTTCAACACTGGTGCATGAGATGGTTCATCAGTGGCAATTCCATTTTGGCGAGCCTGGCCGCCGTGGCTATCACAA
CAAACAGTGGGCGGCCCGGATGGAACGGGTAGGACTAATGCCTTCTGATACCGGCGAACCGGGAGGCAGGAAAGTGGGCC
AGAGCATGACCCATTATATTATTGCCGGTGGCCCTTTCGATATGGCCTGTGATGAACTGCTGACAGGCCATTTCCAGCTTTCCT
GGATGGACAGGTTTCCGCCTTACCAGCCTAAGCCTGGCGCTGTGCTAAGCCCTACAGGAAAAGGCTATATTGACGACGAGGA
AGATGATAGCGAACACGAACAGGAGGTGGAGGAAGGGCGCGACCCGGTTGAACTCGACGACGAGATCATAGAGGCCATGC
GATTTGTAACCCCACCGCCTGAAGCACCGGTGAACAAAACAAACCGGGAAAAGTACAGCTGCCCGGTGTGTCATATCAATCT
CTGGGGTAAACCGGGGATAGTGGTTTACTGTGGTGGCGAGCACTGTAATAAAGCCGCGTTAGTAGTCTTAAAATAAAGTCCTT
TCGGACTTTATTTTTTTTCCATTTCCGAGGTCGTGATGTTATTAATGCTGTACTTCGCGGCTTCTTTTAAAACAGTTTCAGCAAG
GCTTGCTGGTATCCAGACCTGAACTAATTTTAATGGTTCGCCGTTCTCGGCTTTAAGAGTGGTGTTCTGGTACAAATCCCAGAT
TCGCTTAACGGTGCTGGAAATGTTTTGCTTGGAACGGCCTACTCGCGTGGCTACGTCTGATGATTTCTCACCTTTGACAAGCA
CGGAATAGCCAATATCTGTTGTGATGTGTGCAAAGGAAGCCATTTGCGGCAGCAGCTGTTTCCATTCTGTTTCTGAAATTCTGT
TTTTCTGAGCCATCTGTGGCGCCTCCGTAGTTTTGGTTACAGAAAGGATATACTCAGAATAAACAGGGGTCAATACAAGTACG
ATTTTTATAAACTTTATTTTATTTGAGGGTGAGGCCCGGTGCGGCAGCAGCGCGCGGGCCTCGATGGTGCCGCGAAGGTGCT
GGCGCCATGCTCGGATTAAAACATGAACCGTGAAGAACTGCGAAACTTGTTTTCGCGGTTCTGAGGGTTGACCGAGCCGCG
AAGCGGCGCTGGTAAGCGATGATATGCACATATCCACAGGCATATTTTTAAAAGGGGCACTGTTGCAAAGTTAGCGATGAGG
CAGCCTTTTGTCTTATTCAAAGGCCTTACATTTCAAAAACTCTGCTTACCAGGCGCATTTCGCCCAGGGGATCACCATAATAAA
ATGCTGAGGCCTGGCCTTTGCGTAGTGCACGCATCACCTCAATACCTTTGATGGTGGCGTAAGCCGTCTTCATGGATTTAAATC
CCAGCGTGGCGCCGATTATCCGTTTCAGTTTGCCATGATCGCATTCAATCACGTTGTTCCGGTACTTAATCTGTCGGTGTTCAAC
GTCAGACGGGCACCGGCCTTCGCGTTTGAGCAGAGCAAGCGCGCGACCATAGGCGGGCGCTTTATCCGTGTTGATGAATCG
CGGGATCTGCCACTTCTTCACGTTGTTGAGGATTTTACCCAGAAACCGGTATGCAGCTTTGCTGTTACGACGGGAGGAGAGA
TAAAAATCGACAGTGCGGCCCCGGCTGTCGACGGCCCGGTACAGATACGCCCAGCGGCCATTGACCTTCACGTAGGTTTCAT
CCATGTGCCACGGGCAAAGATCGGAAGGGTTACGCCAGTACCAGCGCAGCCGTTTTTCCATTTCAGGCGCATAACGCTGAAC
CCAGCGGTAAATCGTGGAGTGATCGACATTCACTCCGCGTTCAGCCAGCATCTCCTGCAGCTCACGGTAACTGATGCCGTATT
TGCAGTACCAGCGTACGGCCCACAGAATGATGTCACGCTGAAAATGCCGGCCTTTGAATGGGTTCATGTGCAGCTCCATCAGC
AAAAGGGGATGATAAGTTTATCACCACCGACTATTTGCAACAGTGCCGGTCGCCGGGAGTCAGCAGATCGACGTCAACGCCG
AGCAGCGATTTCAGTTCTTCTTCCAAATCGCCCAAGTCCAACAACGTGGCACCGGGCAGCGCATCGACCAACAGGTCGAGGT
CGCTGCCATCCCGGTCGGTGCCATGCAGCACCGAGCCGAAGACGCGCGGGTTCGCGGCGCGAAAGCGGCCTACCGCTTCAC
GCACTGCGCTTCGCTTCATGTCAAGCACAACAGACGGTCGCATGCGCATCCTTTCTTATCGAAACTCGTTGAGATGATATGCAA
TCAAGAATAGAATTTCAAGAACTCACAAAGTAACGCGGTGGTTAATATCCTGTACCCACGGATTGCCCTTAGCGCTGCCTATAT
CGGCTAAAGCACTCCGGTAGCTTGATTCACCCCACGGCCACGGCAGGATCTTGGCCGTCGCAAGCGCCAGGGGAAAATCTT
CAGCTGCAAGCCTGAGTGATTTCATGTGCGTGTAATCCATCGCCCAGATGATTTTTGTGAAGAAGAACTCGCGCTCGTTCATG
TCCGGGCGCGCGTCCTGGCCCTCCGTGCCAACGGCCAGCAAGTAATCGGCCTGAATTGGCAGTATCAGCGCGCGTAGTAAGT
CATGGATCGCCGGTTGCGGCAGTCTGCGCGCCAGATTAATTACCCGGTCGAACTTCAGTTTATCCTGCTTGCGCTTGTCGGTCT
GCTCCATCAGATTTCATGGCCCCCTTCTTCATGCTCATGCTCATGGGTGTGTTCTTTTCCGGTATGGCTCTGTTCCGCCTGAGAC
GTCTGCGGCATGGCGTAATCGTCGTAAATGCTGCTGTCAAAGTCGTAGTCTGATGCTTCGGCATAATGCTCGTAATCGGCATAC
TCCTGCGCGCTCCACTGCTGATCGTCGGCAGCAGCATAATCATGGGCCAGCTCTGCATCATTTTGCTGTGCTTCATGACGCCGC

AGGCCAACGGAATCATCCATAGGGTTCTGCTTAAGATGAAAGGCGTCCTCTGCGTTGCTCACCGGCTGATAATCAGTGCCGGT
TGTCATGTTATGTTCATCGGGTTTCTGGTTAAACGCCATGCTTTCCCCCGTGGCTTCTGGCAGACCTTTTTCAGCTGATCGGGT
TTCTAAACTGGTATCGCGGCCAATATCCTTAAACCTGGCCTCAAGCCCAAAGAAACGGTCAATTTCTGCGGCCGTGGTTTTCG
GGCTGTCGCGGCTCACGCTCGATGCCAAAGATTTTTTATCGTCGGTAAAAATTTCCACCTCATGACGCGCACGCGAAATACCA
ACATAAAAAAACGTCCTTAGAAGTGGTAAGCGATTTGGTATCTATGTTGAACAACACGCGATCACAGGTAAGCCCTTGGGATTT
GTGGACGGTGGTTGCATAAGCATAGGAAAGATAAGAAGCCTGTTTTTTGTCCAGCTCAACCGTGCGCCCTTTTTTGTCCTCAA
GCGTCAGTTTTTCACCCTCCACGGTTTTCACCGTGAAGCGGTCGCCGTTGGCAACGTCCAGCGTTTTATCGTTACGCGTTACC
ATAACCTTATCGCCCGGCGCCAGTTCGGCGCTGACTGCCTGGTATACAGACAGCTTGGTGTGTGTACGCGGGCTGAAAGCGA
TCTGCTCACCGCTGCTGCTTTCAACCGTCAATTTGTTGCCCGGCCCGGTATCAAGAACCTGGTAAGACTCGCCCCGCTTCATAC
CATTTTTGTAATCCTGTTCGGGGATAATGATTTGCCCTTTACTGAAATAACGGCTGTCGCGGCGTTCCGCCTGTGTCGAATCCA
CGCGGTCAAGTAGCGTGAACGTTTCGCCGGTTCCGGCAAGCCCCAGATTGCCCCGGATGTAGTCATTGAGGGTTTTGCGTGA
GGCGTTCGTACCAGAGATTATCAGGGTGGCATCCTGTTGTTCTGAGGACAGAGACAGGTAGCGATCGGCAAGTTGAGCGAG
TCGGGGCGCTTCTTCCTTCAGTTCGTTCACGCCGGTGATATTTTTCAGGGCGCGCGCGGCATTACCTTCAGCGGCATACTTAA
CCGCCTCAAGCAAAACTTCATTCTTCTGTCGCTGAATGTCTTTCATGTAGCTGGTCTGCATATCTGCTTTAATCAGCTGCTCAAA
AGGCTTACCGGCTTCTACCGCTTTCGTCTGTGACGTATCCCCCAGGAATACCGCGCGAGCGTTATGCTTCTCGATCACCTCCAT
CAGCTGTTTCATCTGTCGGGCGGGTATAACCCCGGCTTCATCAATGAATACGACTGATTTTTCATCCAGCTTTTTATCCTTCGCT
TTGAGGAAAGCGGCAACGGTGCGGGCCGGTAATCCATCATCTTCAAGCGCTTTTTTCTGTGTCCCATAGGGGGCCAGCGCCG
TGACCTTCAGCCCTTGTGACTCCAGCAGCTCTTTAGCGGCCATCGTCATATAGCTTTTACCGGTACCGGCGTAACCATGTGCGG
CCACAAACCGATCTTTGCTCGTCACAATTTCTGTAACCGCGCGCATCTGCTCCTTCTTGAGGTTTTCCCGGCAAGCAGCTGGC
CTGCAATCTCTGCGGTCAGCTGTCGCGGCATCTGCCCCCGGCCGCGTGATTCGATAGTCAGAATGGAACGCTCAAGGCGAAT
ACCCTCCACGGTAGTGACGCGGTGGCTGGTCTTTTTAAGCCTGCCGTTTTTAATACCATCATCTACCGCAAAACGGGCTTTATC
CGCACGCATCCCGCTATTCGTCAGCGAGTCGATCCACTCTTTGCGCGTCAGAGTTTCGGCCATAACTGAAGCACCGACCTTCA
GAGTTGATTGATACCGGGCTTCGCCCTCGATGATGGCGCCCTTCTGTACCGCCTTCAGGTACGCTTTTTCAACATCGGCTATTG
TGGCATGGCCCAGCACCTGCTTATTAGCGATTTGAATCAGCTTCTGGCGTTCAAAGCTGGCATCGCGCTCTGACAGCGACTTA
ACTGCAAACTGGATAGCCCGGTCAGCTTTAACCTCCGGGCTGGTAAAATCCGGGGCCATGTTGCGCGCTATATCAGCCTCCAG
AGGTTTACCGTGTCCCTGCCATTCACGGTTATCAAAATCAATGCCGAGCGTTTTGGCGCGGCTGGCCCATTCCTGGTGAATTTC
TTCACGGGAATGCTCTGTTTTCTTTTCACGCGTAGCCATCGAGACGCGGCTTTTCGTCTGAGCATCGGCGGTTTCCCGCGTCA
GACCCATTGCAGCGAGTCCCTTTTCAATTTGCTCCGACCGGCGGGAAAAGCGCGAATCTGTTCATCTGAAAAATGGGCCATAT
CGAACGTGTTATTTTTGCTGTTGTAACGCAGCTCATAACCGGCTTTGGTCAACTCCAACGCCAGCTCCTGTTTGTAAACATCGC
CCAGGTGCATTTTGTTACGCATCAGCTCATCATTTTTGAGCGCGCGCCACTGGCCGTCCTCGCGCTGGGTCATGTTCATGACA
AAAGCGTGTGTGTGCAAATCAGGATCTAGCGCCCTGGAAGTTTCGTGGCGGAAAGTAGCGACGACAAGGTTATTGGTATTCT
GGGTTACTGATTTCCCCTGGCGAGTCGTCCGGGCCTGCGCGAGTTTTTCAGCTTCACGCACAGCAGCGGCAACAGCTTTTTC
ATGAGCCTCGATAATGGTTTTATCGCCGTGTATCAGCGCCTGCATGGATACCCCTTTAGGCGCTGAAAACGTCAGGTCGTAGCC
CAGACGCTCTTTTTTGGCATCACCCACGTGTCGCTGCATATGCGTGAAGGTATCTATCTCTCCGACAAGCAGCTCTTTAAACCG
GGCTGATTCAACGTCCCCGGATAAGCCGAGGGCTTCAGCTCCGGTTCCCTGCCAGGACGTGAATGATGAATCCTTACTGTAGT
AATCATCCTTTGCATCAGAGTAGTAGCCCACAACGCTAGTGACGTTCTGGCGGGTAATCGTGGTTATATCAAGCATCAGATCTC
CCTCAGTTCAATGCCAGGAACAGGGTTTTTGCGATGGTATTTAACGTGTTTAGCCTTGAACTTAGCGACGGGCATATCACCAG
GCAACGCCAGATAGCCGGTGAGGTTTGGCAACATTGATATTTCGGTAGGCGTTACGGCACGAACAACTTTAACGTCGCGGCG
TTTACGGACAATCCAGGGCTTCTGAGGATCGGATTCTTTACGCTCAACTTCGCCTTCTATCTCACCGAGTGAGCGCGACATTTG
ATCCAACGTTTCATCACCGAGACGGCTGCCGCCCAGCACGATGTTAGAACGCATGTTAGCCAGAATTGTCTGAGCCATATCCC
GACCATAAACCTTAACCAGCTGAGAATAGGTTTGATAGCCAGCATAAACACACAGACCGCTTTTACGCCCTTTGGTCAGTGCA
TCGTTGAGGTTTGGCAGAAACTGGAGTGATTCCAGCTCGTCAATAAATACATTAATGCGGCTTTCTTTTTCACCCATACCCAGC
ACGATAGAAAAAATCGAATCCAGCCAGCAGGAAATTAGCGGATTAAGTGACCTTTTCATTTCTTCCTGCCAGGTGATAAACAG
GGTTCCCGGCTTTCCATCATCAAGCCAGTCACGCAGGGAAAAATTACCTTCCGGCATTTTCAAATGTGGGGCAAGATTCTTAC
TGAGAACAAATCGCGCGCTTCCAACTGCTTTTTCAGACCCGGAAAAAATAGCTTCGGCAGGCGTCCCCATTAAAAATTCTTTT
AATTTTTTCTGGTCAACGTTACAGGCCCAGTGAATAACTTCTTCCATAGTTACTGTGCTGTATAGGCTGTGAAGTTTTTTCGAAA

CTTCACTAAAAATAAGACGGCCATAGCCGAACCATTCTTCAGTAGCCATATCAGGGCTTTCCTGAACAATAGAGTTCACTAAAC
GCTCGTAATCATATGAACGGCGAATTTCATTGAAAAACACCCAGCCTTCAGTGCGTTTATCATAGGCGTTTAAAATAACATCGC
CGGGACGATAGAAATTCTTTAAGAACCCCCCATTTGGATCTAAAGCAATATTTTTGCCGCCTCTAATGATGCTCTTAAATAACAG
TTCATTGAAAATTGTGGTTTTACCAGTACCGGTTGTACCGGCAATCGAAAAATGCAAGTTCTCAGCGTATGTAGGTATGGGGAT
ATTAGCCACGGTTAACTGGTTGACACCTCTTTCGCGTGTTTTATCAGCGAGTGTTCTGGCGCGAACAAGCTCTGTACCACGATA
AATCTTTTTGAATCTTTCGCCTTTAAACACGCGTGATTTATCATAAATGATAAAAGCGATCAGACCGCCAACACCAATAAACCA
GCCAGCAATTAAAGCTGACCATAAAGGCCATAGCGAAAAAGTATTCTTAACCAGATACGGAATCAGGTATTTAGCCGTGGATG
GATCAATACCGTAGGTAAATTTTGCAACTAGAAACCATACCATCACTGGAGGCAAAGTAATTGCAAATAAAAATGCTAAGCCTC
TTTCTCTATCGTCCATTTCAGCGCTCCTTTTTTGGTTCCCAGACTTTGTAGCCGTTACGTTCAACCTCTGCTTTTGCCGCTTTGG
TTTTGCCCGGTTCTGCTATCGAGCGCAGGAGGATTAGCGTTTCAATAGCGAGTGATTCATGCAACATCATCTGTCCTGCCGGTG
GGAATTTTACGCCAGATAGCGTTTCGGTTATTGCCTTCAGCTCATCGCGCAGTGGGCCAAAATCCGCATCTGAAGCACGGTCA
AAAAGATAATCCAGTTTGCGATTTACGTCGCTCAGCCGGTCGGCAACTATTTTCAACCCGGACTCCCGATCACCTGGGCCAGC
TTCAATGCAGCGCCGCAGATAATCTGACCGATTACCTCCTGAAACCAGGTCTATATAGGCCAAAAGTTCATCTGATACTTTTGC
GGTTATTATTGGCATTCAGTCCTCACATTGTGCATTTCTTAAACAAAAAATTGGGATCTAACAAGCTGAAATCTTAGTATTACCA
AAGTAATAAAGCAAACTCATTATAAAACAATGGGTTATTGGGTGTTTTTAATACCTAATTATTACCGAATATTGACGCTATTTATT
TTTTTATCTTTTAAATCAGTACGATAGCGTGATTTATCGCGCTGCGTTAGGTGTATAGCAGGTATAGCAGGTTAAGGAAAAAAA
ATCATCTTTTTTGGTAGGAGCGACCTCCGTAGGTTAAGGGTCATTTGGCTAAAAAGCGTCCTATTCTTTGATGGTCATGCTTGC
ATGACCATCTGAGCAACCAAAAACTACAGATAAACTACAGAGAAACTACAGATAAACTACAAAAAACGATTTACCTTAGCGTT
GTCAGACTACTAATAGACTACAAGGAAACTACAAAGAAACTACAAAGAAACTACAAAGAAACTACAAATAGACTACTAAAACC
GTGGCAGACTACTAATAGACTACAAGAAAACTACAAATAAACTACAAAACTGGATTGACCCCTTCTTACGAGTGTTGTAGAGT
CATCTTCATACAACGGAGGGGTTATGAATAAACCAGCAGATCTGAAACCCCGCAACTTATCGGCTGCTGTCAGATTGCGCCTA
AATGAAATCGAGAACTGGCTGGACAGAGGGCTAACGCGGCATGAAATTGCTGAAATCCTCGACAGCGAATACAGCTTTTCG
GTAACAGCCAAAGGGCTTGAGATGGCACTGTATAGAACGCGGCAAAACCGAAAAATGTATTGCACAATACACATGATAAGAG
TAGCGCGAAGGGTGCAGCGGAAAGTGTATTGCACAATACACAACCGTCTGAGCCTGAAGCGCAGGAAAGTGAAAAAGCAG
AGAGTCCCGGCATTATTGATAAAGAGTTCTTCAATAAAATCGGTGAGGATTTCAACCCTAAGAAGTTCAACAAAAAAATTCTG
AGGTGATTTATGAAAGTAGCGGTAATTAATTACAGTGGCAGTGTTGGTAAAACATTAATTTCATCTTACCTGTTAGCCCCGCGC
CTGACTGGTGCAAAGTTCTATGCGGTAGAGACTATCAACCAGTCTGCTTCCGATCTGGGTATTGAAAATGTGACCAGTTTTAA
AGGTGACGACTTCTCACGTTTGATTGAGGATATTGTTTTTGAAGATGCAGGCATTATTGATATTGGCGCGTCAAACGTTGAAG
CGTTCCTGATGGCTATGTCTCGCTTTGACAGTGGCGCGAACGAATTTGATAAATATGTAATCCCGGTGACGCCGGATAATAAGG
CGATTGATGAAAGCCTGAAAACGGCACACACGTTAAGTAAAGCGGGCGTGAGCAGCAAGAAAATTATCTTTGTTAAACCGTA
TTAGTCCAGACAGTGAAGTAGAAGATGTGCTGGCGCCGGTGTTTGAGTTTGTCAAAGAAACGAAGATTGGCAAAATAAGCA
AGAAGGCTGTTATTTATAACAGTGAGGTTTTCGAATATCTGGCGTTTCACCGTATCTCATTCGAAGTATTGACCGCTGAAGATC
CAGAAGAATTCAAATCCCGTGCAAAACAAACAACCGATGCTGACGAGCGCAAAAAACTGGCACGCCGTTATACATACATGAA
ACAGGCGATTCCGGTAAAAGCTAATCTCGATAAAGCATATGCGGCTTTAATGGGAGAATAAAATGGAAAAGCAGCCGGATAA
ATTAGAAGTTCTGATGGACTGGTTTTTAGGTGACGCGAAGGAAATCACCGCAACTCAGAAAGAAATGACGCAGAAACTTTCT
GAGCTTTCGGAAAAGCTGGCAAAAGACACCGAAAGTTTAGGAGAGACGGCAGACTCTTTTAAACGGGCTTTAGTAGAAAA
CCAGCGTTCAATTAGCCTGGCAATTAGTGATGATGCTAAGGCGCGCGAGGAATTTCTAACTAAATTCCGCCGCGCGCAGGCGT
CCAGTGCTGAGACGTTTACCCGTCAGATCCTTTTTATTACAGCTGGCTGCACCATCGTGGGCGCCGCAGTAGGCGCCGCGATA
GCGATACTTTTACTGAGATAAAGCAAACCGGGCGTGTCCCGGTTTTTTTGTCAAGCGGAGCGCGGAGGCCGAAGGCCGGAG
GCATTAGTGGCCGCCGCCCCCGTAAGCGGGGCGAGACGGGAACCGGCTCGAAGCGCAGCACGGCAGAACGGCCCCGCAG
GGGCAATGCCCGTTTTAATTCATCGTGACAGTCGCGCGTGACCATCACGGGGAGAAAAATAATGAATGACCGACAGCGAGAA
CTGGCCCGTATACGCCAGGCCCGCCGCCGCGCGCGCTCAAGGAAGAAGGCACAAGCGTGACAGTCACGCTAACAAAACAG
GAAGAAGCAATGTTGCAGGAGCTGTGCCGGGTTCGCCGTCCTGGACGAACGCCTTATTCAACGAACGAATTTTTCCAGCTGC
TGCTTATCCGCAACTGGCAGCAGTGGCAGGAGCAGAAGGCACAGCTGGGAAAATGCCAGGCTTGCGGAAAGCTGAAAGCG
GAGGGGGTGCGAGGGTGAACGGAAAGGCGAAACCTTTAACTGCTGGCTTGCCGTCGAAGCCAATGAACTAAATTTGTAGTG
TATTGTGCAATACACATTTACACAGAAACAAAAACCACCGGCAATTCCTGGAACCGGATACCTACGGCTATTCCTGGGTGAAC

GGTACTTTTTGCACCTGGGTGCGCTGAAAAAGCTGAATATGCAGGGTGACGTTCGCGTGCTGTTCTGCTTTGTAGACTGAATG
CGCCAGCTATACGCCTGACTGCTTAAACCTGGTAAAGTTCTGCAACCGGCACTGACCGGAAAGCAAGGCAGGGAAGACCTA
AGCCAGAAACCTTGACTGCTCCCGCCCTTCAGGGCGGGGATTGCGGATCATGTTCTTCTCTTTCAGGGATTCAACGCAGACA
AGAAAGGCTTTCAATTTCCTATACGTGAACGGCCGCGCAGCGGAAAGAAACAAGCCCGGTCAATCCGGGCTTGTTTCTTTAG
GCGGCTCAGAAATCGCCTAAAGGCCCGGCTTGGCCGGGCAGTCAGTGCTATTTAGTTTGTTGCAGCAGCTGGCTTAATTTTG
CCGCCAGTGCATACGTCGATTGAAAGCGCCTTGCAGGCGCTGATTAGGCAGCTGGTTGAACGCTTCGAGGCAGGCGCGCAG
CAATAATTCTTTCTGAGATTCGACTTCTTTTTTCAGTTGGGAAGGGGTGGTAACTGTAGTCATGCTTGCTCCTTAGTGAGCCGA
TATCGGCAATTTTTCGGGTGGCGGTGTTGCCTCCCGATGATTTAATTATCGGTGATTATGCTTTTAAAGTCAATACAGGTACGG
AATTTATTTACCTGTTTTTATGCCCGTCAGGGCATGGAAGGCGACCGCGCCGGACTCCACCGGACACCGGCCGCAAATCGCC
GGAAACTGCGGGACTGACCGGAGCAACAGGCCAACCCCCCTCCCTGCTAAGCCATAACCCAGCCCGCCGCCACGCAGCTGC
CGCACGTCCCCACGGGGTGCGCAGTGGGCGCCGCGCGCCTGCGCGCGGGTACGGCGGCCCGCCTGCGGGTCGCGGCGCC
GTACTGCGAGTTAGCGGCCGCCGCGCGGCCGGTTACGGGGGACACCGCACAGTCACGGCCAGTGCCCCGCTGAGCTGCAC
AATCCACGGATAACACAATAGCGCACTGGCAAAGGATGCCGACGCCTGAAGGGCGTGGGCACCCCGAAGGGGCGGGGCGG
CCGCTTGCGGCCGGGCGAGTCCGGCGCAGGGTGTGGCCTGCCAAGCGGAGCGCGGAGGCCGAAGGCCGGAGGCGTTAGC
GGCCGCTGCCCGCGTAAGCGGGGCGAGACGGGAACCGGCTCGATGCGCAGCACAGCAGAGCGACCCCGAAGGGGTAACG
CCCGGTGTGGCATCAGGATTTAGTGCAATGGCAGAACATGAGCTGGAGAGATCACCGGCAAGCAGCAGCAAAGGGGCGGC
ACAGCCGCCCCGATGGCTGTTTGCCGATACCGGCGATTAATTAGAGCGGTGTTTAATATCCCCGCGTTGCGGGGGACTAGGTT
TCAGCAAGTCATGTTAAATACGTGTCCATCATGTAAACTGAAATCCCCAATAAACAGATCCCGCGCATAGGCTACGATGTCAAA
ATATCGGGCTACGGATTCCGGAATATCATTCAGTAGACCGCTATCATTCAGGTATTCCTCTGCAAAAGTTTCTTCGTCCTTAGCT
TCGCCCATATAGGCATCTCTAAACAGGTCGAAATCAGTGCTATTAAACAGATCAACAAAGGCCACAAACGCCGCTTCGTTACCT
TCCTCGCGGGCTTGTTTAAAGCCGTTAATAAAATCCCAGTTGATATGGCACTCTGACGCCATACCAGACGGAATACCCTCCCAA
TCTTGGAACATAAATTCTGGATCAGCCTCATTTGCGTGTAACTCGCGGCAGCGCTCGTAAAACTCCTCTGAGCTATCAAAATCG
GTCAGATCGAGCCAGGCTCCCGCAATGCTTCCGCAGTTGTATTTATGGTAAGTGCCAACATAAACAGAAGGGGTCGTAATATC
AGTCATGGTGTACTCCTTAAAGCGCCGATACCGGCAATTTTTTCGGGCGGCGGTATTGCCTCCCGATGATTTAATTATCGTTGAT
TATGCTTTTAAAGTCAATACAGATACGGAATTTATTTACCTGTTTTTATGCCCGTCAGGGCATGGAAGGCGACCGCGCCGGACT
CCACCGGACACCGGCCGCAAATCGCCGGAAACTGCGGGACTGACCGGAGCAACAGGCCAACCCCCTCCCTGCTAAGCCATA
ACCCAGCCCGCCGCCACGCAGCTGCCGCACGTCCCCCACGGGGGTGCGCAGTGGGCGCCGCGCGCCTGCGCGCGGGTACG
GCGGCCCGCCTGCGGGTCGCGGCGCCGTACTGCGAGTTAGCGGCCGCCGCGCGGCCGGTTACGGGGGACACCGCACCGTC
ACGGCCAGCGCCCCGCTGAGCTGCACAATCCACGGATAACACAATAGCGCACTGGCAAAGGATGCCGACGCCTGAAGGGCG
TTGGCACCCCGAAGGGGCGGGGCGGCCGCTTGCGGCCGGGCGAGTCCGGCGCAGGTGTGGCCTGCCAAGCGGAGCGCG
GAGGCCGAAGGCCGGAGGCGTTAGCGGCCGCTGCCCGCGTAAGCGGGGCGAGACGGGAACCGGCTCGATGCGCAGCACA
GCAGAGCGGCCCCGAAGGGGTAACGCCCTGTGTGGCATCAGGATTTAGCACAATGTCAGAACATAAACTGGAGAGATCACC
GGCAAGCAGCAGCAAAGGGGCGGCACAGCCGCGCCCGATGGCTGTTACTTGTCTTTGTCGCGTAGCACTTTGATTAGGCCG
GTTACGGCCGTAATCAGAGCGGCCAGCGAGGTGATGATTTGCGGTAGGTTTTCGAGGATGGTAGAGGTCATATAGCACCTGT
AGAGAAGTTGGCGGGGTGTCGTTTCCGACGGCCGCACTGTAACCGGGCGAATAAGGCAGGTTGTCAACAGCTTGAGCGAA
GCGTCTGTTGACAACCTGCCGCGCCCGGTTTCACTGCGGTCATAGGCGGAACGACCCACGCCAACGGAACGGCTTTATGACC
GGGCAGCTGAGATACCGGCGAACCTGGCTGGCGGCTGACGCCAGCCGCCAAGCGCCAGCGCGGAGGGCAAAGCCCGGAG
GCCAAGCGGAGCGCGGAGGCCGAAGGCCGGAGGCCGGAGGCGTTAGCGGCCGCTGCCCGCGTAAGCGGGGCGAGACGG
GAACCGGCTCGATGCGCAGCACAGCAGAGCGGCCCCGAAGGGTAACGCCCGGAGTCTGCCGCTGTTTATCTCTCGTTCCATC
TGAAATCGGCGGTAAGGCCATTAAAAGGGTCAGTTTATCAGGGAGGCGTTAGCCCCCCATGTTGTTAATCATCAGGCAATATC
GTCTTTGTAGCAGGCATAACCGAAGCTAAGCTCTGTTTTCATATAGTGGCGGGCAAAGTCCAGGCATCGTGGCCGAAGTCCTC
ATAATCTGCCAGGACGATTTCGCGGGCTTTGTGCCATTGCTGTACGGAAGAAAGCGGCAGAACAGGGCAGGGGTGCGACCA
GTCAGTGACGGTGTTGCAGATCATATCTGCCAGACGCTCCAGGGAGCCGTAAACCAGCTCTGTGCGCAGATCCGCCACCATG
CGTTGTTTACGCAGTGATGCCAGATAATCAATCTCTTTGGTTATATCAGAATTTAAGCGGGTCTGGTAATCCATGATGTACTCCT
TTGCGCGCCGATACCGGCAATTTTGCGGGCGACGGTGTTGCCTCCCGATGATTTAATTATCGGTGATTATGCCCTCAAAGTCAA
TATAAGTACGGAATATGCATGCATAATTTTATATCTTGCAAAGCGTTCATAGAGTGCCTGAATCGCTTTCTGACAGCCTCAATAA

AAAAAGGCGGGGATTCCCGCCTTTTTTCTTACAGCTGCTTACGTGGCTTTTTACGCGTCATATACAACGGTATCGCGCAGTCTA
CCGCGTACAAAAAGCACGCCAGCGCGCCGCAACCGTACAGAAACGCAAGCGGCTTATTATCGAAGTAGCTGAAAACCCCTGT
CGCCGCACACAGGCCCAGAACAGAGACGCAGGCGCAGGTGATCTGCACCAGATCCCTGTATCCCGCACGAACGATAAACCA
GGGCAGGGCAAGCGCAGCGGCGCTAATAATTAATGCGAGAGGGACAAAAACGAGATAGTGATACATGTGAACTCCTTGATG
GTTGCCGATACCGGCGATTGTTCGGGCGGCGGTATTGCCACCCGATGATTTAATTAGAGGTTTTGCGCGTCCAGGAGATTGAC
CTGAGCCGGGGTAACGTGAAACTTTTCCCCTTTATGGATCACGTTATGCGGGGCGCTAATTTCATCACTGATAAAGCTAACCG
GGTAACGTTTTTTACCGCAAATCCGCTCGCTAAACCATGCCACTTTTGCCGCTGGCCGATCCACTGGATGAACAATCACACCG
GCCATGCTGCAACCCGTTGCGGGTTCGTCCAGCGTAATGCTTACCGGGACTGTATCCCGGTAAAAAAACTTAACCGGCGGCA
CACCTGCCTGCGTAGCGGCTGCGACAACTGCAAGCCCGATAACCGCTATCCGATTAATAAGCATTTTATTCCCCTTACTCATGCT
GATATCACCTTGCCAGCTGTTACCAGTTTACGAAATTCACTTTCATGAATTTCACGCCCATGCTCAAGCGTTGAATACACGTTGC
CGATAAGCCAGTTACCAGCCGTTTTTGTCTCGGTATACCACCATGCTTCTGTCAGAGTAAGCAGCGGTTCGCTATCCTCGCCCT
TCTCATAGATCCAGATTTTAGTGACGTCTTTACCCGTTTCGTTGCTGCCCTGAATGGTAGGGTCAAAGGTATGTTCAATCTCTAT
ATCGAAGTAACGCTGAAGGAAGTTAGTAAAGTGCATGACGACTCCTGTAAGCGCCGATACCGGCAATTTTTCGGGTGGCGGT
GTCGCCTCCCGATGATTTAATTATCGTTGATTATGCTTTTAAAGTCAATACAGGTACGGAATTTATTTACCTGTTTTTATGCCCGT
CAGGGCATGGAAGGCGACCGCGCCGGACTCCACCGGACACCGGCCGCAAATCGCCGGAAACTGCGGGACTGACCGGAGC
AACAGGCCAACCCCCTCCCTGCTAAGCCATAACCCAGCCCGCCGCCACGCAGCTGCCGCACGTCCCCCACGGGGGTGCGCA
GTGGGCGCCGCGCGCCTGCGCGCGGGTACGGCGGCCCGCCTGCGGGTCGCGGCGCCGTACTGCGAGTTAGCGGCCGCCGC
GCGGCCGGTTACGGGGGACACCGCACCGTCACGGCCAGCGCCCCACTGAGCTGCACAATCCACGGATAATGCAGGAGACG
AATCATGATAGGAGGCTGAAGGGGAAATGAGCGGCAGCAGGGGAAGGGGTTGCCAAGCGGAGCGCGGAGGCCGCAGGC
CGGAGGCGTCAGTGGCAGCTGCCCGCGTGAGCGGGGCGAGACGCGTAGCGGCTCGATGCGCAGCACAGCAGAACGGCCC
CGGAGGGGTGACGTCCGGGGGTTCGCTTTTAAAGATTTTCGACCACATCAGTAAATCGTAGTGACACCATGAAGCAAAAGTA
TCGTGCAGACCAGAATCAACAACAGTAACAGCAGACCTTTTTTTCGACGTAATAAAACCGGCCCCAAAGCGCCCGCAAGAAG
CACACTCAACACGATTTCCGTTATACTAATCGTATTCATAATCTCTCACGTTTCCCTTTTTAGAACTCTGCCACACACAGATAAAA
CCTTATAACAAGCTACAAAACCCGTTATTCAGACGCGGTAATGCCTAGTTTTTTTGCCAGTATTTCAGATGACAAAGAAACCCT
TATCCACTTCCCGGTTACAAGGTGAATGATTGTGGCTTCATGCCCTTTAACAGAGTGGGATATTAACTCACGCTGAATGATATG
CGTCTCTCCATCATTTCCCGTCACGGCAATTAGGCCCTCTGTAGAACAGGGGTTGATACTACTAATGTTGGTGTTGTTTTCGGT
TTTGTTCAGCATCGCTGATCCTCAAATATCGGTTTGTGTTACGTCTGCCGCTTTGCGCTGGATAAGCGACTTAAAGAAATCCGA
CGCCTTCAGAATATCGCTATCCTGGAAGTCGGGAAATGTCGCTTCTGTCAGCTCCTGCGCCGTACCTGCCCGTATAACGGTTTC
ATCGCGGATCATCGTTCGCTTAACGCCATCAGCCTGTTTTTTGCTGAAGGAGTGCCGGAAAATAATCGCGTATTGGCCGCCGC
TATCCTGATGAACAGAAGTATCCCAGCGATGATTACCCTGTTTACGATAGTGGTGATAGATTTGCTGGCCGTAGCGCTCCTGAT
CGGAATTACGGTAATCGAACATTAACAGTTCCAAAAGCATTAACCGATCTGGCAATTGCCAGGCGGTAGGGTGTTGAAGTGTC
GCTAACATAGTTTCCCCTGAGCGTGACAGTCACGATAAGGCGGGCTTTGCCCGCCTGGTTATCAGTTAATCAATGGCACGATA
AATACGATTCTGGCTTTCGTTCTCCAGCGTATTAACGTACTCCCGCAACAGGTGATACCGGTTAGCCATCGTTTCGTTCAGTTCG
GCTTTGCCTTCTTCATATGCCAGGCCGCAAAAGTAACTGTAGGCATACAGACAAACAATAATCCCTACTTCGCGCGCGCTGCAT
TCACCTTCAAAATAGTTAGGTAACGAGAGCCAAAGAGGTTGGGGCGCTTCCATAAAAAACGCGCCATTGCTGGCCTGAAGGT
ATTCCCAATACCCTCCCTGGTAGTCTTTAGCGTAACGATTCAGAAAGGACTGAATGAAGTGATCTGCGCTGAAGAAAGCGCCA
CGAAATGCCGCAGGCATGAAGTTCATGCGGGCGTTTTCAGAAATGTAGCGGGCGGTGATTTCGATAGTTTCCATGATACTTCC
TCTTAAAGCCGATACCGGCGATGGTTAAGCGGCAGGCACATCACCTGCCACTTTTTAATTATCGTACAATGGGGCGTTAAAGT
CAATATAAGTACGGATTATATTTACCTAATTTTATGCCCGTCAGAGCATGGAAGGCGACCTCGCCGGACTCCACCGGACACCGG
GGGCAAATCGCCGGAAACTGCGGGACTGACCGGAGCGACAGGCCACCCCCTCCCTGCTAGCCCGCCGCCACGCGGCCGGT
TACAGGGGACACTGAGAAAACAGAAAGCCAACAAACACTATATATAGCGTTCGTTGGCAGCTGAAGCAGCACTACATATAGT
AGAGTACCTGTAAAACTTGCCAACCTGACCATAACAGCGATACTGTATAAGTAAACAGTGATTTGGAAGATCGCTATGAAGGT
CGATATTTTTGAAAGCTCCGGCGCCAGCCGGGTACACAGCATCCCTTTTTATCTGCAAAGAATTTCTGCGGGGTTCCCCAGCC
CGGCCCAGGGCTATGAAAAGCAGGAGTTAAACCTGCATGAGTATTGTGTTCGTCACCCTTCAGCAACTTACTTCCTACGGGTT
TCTGGCTCGTCAATGGAAGATGGCCGCATCCATGATGGTGACGTACTGGTTGTGGATCGCTCGCTGACGGCCAGCCACGGCT
CAATCGTAGTCGCCTGCATCCATAATGAATTTACCGTGAAGCGGCTACTGCTGAGGCCCAGACCCTGCCTGATGCCGATGAAC

AAAGATTTTCCTGTGTACTACATTGACCCGGATAATGAGAGCGTTGAAATCTGGGGAGTGGTTACGCATTCCCTTATCGAGCAT
CCGGTATGTTTGCGCTGATTGATGTCAATGGCATGTACGCCAGCTGTGAGCAGGCATTTAGGCCAGATCTGGCAAACGAGCA
GTGGCCGTTTTATCCAACAATGACGGCAACATTGTGGCCCGTAATTACCTGGCGAAGAAAGCGGGCCTGAAAATGGGCGATC
CGTACTTCAAAGTCAGACCCATAATCGAGCGTCATAACATCGCTATTTTTAGCTCTAATTACACTCTCTATGCCTCCATGTCGGCC
CGGTTCGCGGCCGTAGTTGAGTCCCTTGCAAGCCACGTCGAACAGTATTCAATCGACGAGCTTTTTGTTGACTGCAAAGGGA
TAACGGCCGCCATGAGCCTTGACGCTTTCGGGCGCCAACTGCGCGAGGAAGTCAGGCGACACACAACGCTGGTATGCGGG
GTCGGTATTGCCCGTACTAAGACGCTGGCGAAGCTGTGTAACCACGCTGCAAAAACATGGCCCGCTACTGGCGGGGTGGTTG
CTCTGGACGATGGCGCCAGACTGAAGAAATTAATGAGCATCCTGCCGGTTGCGGAAGTCTGGGGCGTCGGCCATCGTACAG
AGAAAGCACTCGCCACAATGGGGATCAAAACGGTGCTGGATTTAGCCAGGGCAGATACGCGCCTAATCCGTAAAACATTCGG
CGTTGTGCTTGAAAGAACGGTACGGGAGTTGCGCGGCGAGGCTTGCTTCAGCCTGGAAGAAAACCCTCCTGCGAAGCAGC
AGATTGTTGTGTCGCGCTCATTCGGCCAACGCGTAGAAACCCTGACGGACATGCAGCAGGCTGTCACCGGATTTGCAGCGCG
CGCAGCTGAAAAACTGCGTAATGAGAGGCAATACTGCCGCGTCATAAGCGTCTTTATCCGTACCAGTCCTTATTCAGTGCGTG
ATACACAGTATGCCAATCAGGCAACCGAAAAACTGACGGTGGCAACCCAGGACAGCCGCACGATAATTCAGGCAGCACAAG
CCGCGCTGGCGCGGATCTGGCGGGAAGATATTGCGTATGCAAAAGCAGGGGTCATGCTGGCAGATTTTAGCGGGAAGGAG
GCCCAGCTTGATTTATTCGACTCTGCTACGCCTTCAGCTGGCAGCGAGGCTTTAATGGCTGTTCTTGATGGTATAAACCGGCGT
GGAAAGAGCCAGCTTTTTTTGCAGGCCAGGGCATCGATAACTCCTTTGCCATGCGTCGTCAGATGTTGTCACCTGATTACACG
ACAGACTGGCGCTCAATACCAATAGCCACCATCAAATAATTACCGGCGCCGTACACGGGCCGGTTAACCCTCAACCGGCCGAA
ACAAGTTTCGGCACGGTTTCGCGGTTTTCGGTAAAAGCCGTTTCCTCTGTATAAAAGATCAGCTAAATTATGTGTATTGCACAA
TACATATATGTGAGGTTAGCAGTGAATTTGCCTACGCCCGAAACCTACGATGAACTTCAGAGAGCCTACGATTTTTTCAATGAG
AAGCTATTCAGCAACGAGCTGCCGCCATGCCTGATAACGTTGCAGCGTGAGAAGCGAACGTATGGCTATTGTTCCTTTAAGCG
TTTCGTCGGCCGTGAGAGTGGGTACACGGTAGACGAGATCGCTATGAATCCGGTGTATTTCTCGATCAGAACCATAAAGGCCA
CGCTTTCAACACTGGTGCATGAGATGGTTCATCAGTGGCAATTCCATTTTGGCGAGCCTGGCCGCCGTGGCTATCACAACAAA
CAGTGGGCGGCCCGGATGGAACGGGTAGGACTAATGCCTTCTGATACCGGCGAACCGGGAGGCAGGAAAGTGGGCCAGA
GCATGACCCATTATATTATTGCCGGTGGCCCTTTCGATATGGCCTGTGATGAACTGCTGACAGGCCATTTCCGGCTTTCCTGGAT
GGACAGGTTTCCGCCTTACCAGCCTAAGCCTGGCGCTGTGCTAAGCCCTACAGGAAAAGGCTATATTGACGACGAGGAAGAT
GATAGCGAACACGAACAGGAGGTGGAGGAAGGGCGCGACCCGGTTGAACTCGACGACGAGATCATAGAGGCCATGCGATT
TGTAACCCCACCGCCTGAAGCACCGGTGAACAAAACAAACCGGGAAAAGTACAGCTGCCCGGTGTGTCATATCAATCTCTGG
GGTAAACCGGGGATAGTGGTTTACTGTGGTGGCGAGCACTGTAATAAAGCCGCGTTAGTAGTCTTAAAATAAAGTCCTTTCGG
ACTTTATTTTTTCCATTTCCGAGGTCGTGATGTTATTAATGCTGTACTTCGCGGCTTCTTTTAAAACAGTTTCAGCAAGGCTTGC
TGGTATCCAGACCTGAACTAATTTTAATGGTTCGCCGTTCTCGGCTTTAAGAGTGGTGTTCTGGTACAAATCCCAGATTCGCTT
AACGGTGCTGGAAATGTTTTGCTTGGAACGGCCTACTCGCATGGCTACGTCTGATGATTTCTCACCTTTGACAAGCACGGAAT
AGCCAATATCTGTTGTGATGTGTGCAAAGGAAGCCATTTGCGGCAGCAGCTGTTTCCATTCTGTTTCTGAAATTCTGTTTTTCT
GAGCCATCTGTGGCGCCTCCGTAGTTTTGGTTACAGAAAGGATATACTCAGAATAAACAGGGGTCAATACAAGTACGATTTTT
ATAAACTTTATTTTATTTGAGGGTGAGGCCCGGTGCGGCAGCAGCGCGGGCCTCGATGGTGCCGCGAAGGTGCTGGCGCCA
TGCTCGGATTAAAACATGAACCGTGAAGAACTGCGAAACTTGTTTTCGCGGTTCTGAGGGGTTGACCGAGCCGCGAAGCGG
CGCTGGTAAGCGATGATATGCACATATCCACAGGCATATTTTTAAAAGGTATTTTATAGATTTTTTATCTTTTTAAAGTCTTTTAG
AGCTATATAACTCATTGATTTAAAATCATAAATAAGTGTTATCTCTGGGAATCCGCCCACCTTGTTATGGGAATTGGCCCACCTTA
CTATGGGAAACAGCCCACCTTACTATGGGAATTAGCCCACCTTGTTATG

>pQEB1_Tn-IS5

GGAATTGGCCCACCTTAGACGAAACTGTAAAAAATGTATTTACTTGTTTGAACTTTGTGGTAGTGTGGAGAGTAATTTTTAACC
CACAAAGGCAAGGCGCATGGATAAGTTGCTGAACAAAAAGATAAAAGTTAAGCAGTCTAACGAGCTTACCGAAGCTGCTTAC

TACCTCTCGCTAAAAGCAAAGCGCGTTCTCTGGTTATGTCTTATGCAGACGTATTTCACAGCTTCAGTAAGCGAAGATGATGAT
GAGATGGCTGTACTCGGTGACTCTACTTTCAAAGTAAAGGTGGCTGACTATCAGCAAATTTTTCAGGTAAGCCGTAACCAGGC
TATCAAGGATGTTAAAGAAGGCGTGTTTGAGTTAAGCCGTTCTGCGGTAATCTTTTACCCGAAAGAGGGGCGTTTTGACTGC
GTCGCGCGCCCCTGGCTAACAGAGGCTGGCAGCCGATCAGCTCGTGGTATCTGGGAAATCGAATTTAACCATAAACTCCTGC
GGTACATTTACGGCCTGACGAACCAGTTCACCACCTACTCGCTCCGCGATTGTGGCAGTCTTCGAAATCCCCGGACGATCCGC
CTTTATGAAAGTCTTGCTCAATTCAAATCTTCAGGCTTATGGGTTACTACTCATGCTTGGTTAAATGACCGTTTCCTTTTGCCGG
AATCCCAACAGAAGAACTTGGCAGAGTTGAAACGATCTTTCCTTGATCCTGCACTCAAGCAGATAAATGAGAAAACACCTTTA
CTTGCTAAGTATAGTATTGATGATTCAGGAAAATTTCTGTTCTCAATAATTGATAAGCAAAATCCCGTCTGACATAAATCAGCAC
ACATGAGCCTGTCATTTGACAAATTTTTGTCATGAAGATGGGCGAATTTCCACACAGCACCGGCGCCCGGCAAGATGGGCGG
ATTCCCACACGACAGCGGCGCCCGGCAAGATGGGCGGATTTCCACACTACAGCGGCGCCCGGCAAGATGGGCGGATTTCCA
CACGGCAGCGGCGCCCGGCAAGGTGGGCGGATTTCCACACGGCAGCGGCGCCCGGCAAGGTGGGCGGATTCTCACGCGG
CAGCGGCGCCCGGCAAGATGGGCGGATTTCCACACGGCAGCGGCGCCCGGCAAGGTGGGCGGATTCTCACGCGGCAGCG
GCGCCCGGCAAGGTGGGCCGATTCCCACGCGGCAGCGGCGCCCGGCAAGGTGGGCCGATTCCCACGCGGCAGCGGCGCC
CGGCAAGGTGGGCCGATTCCCACGCGGCAGCGGCGCCCGGTAAGGTGGGCGGATTTCCACACGGCTGCCGCGCCCGGCAA
GGTGGGCGGATTTCCACACGGCAGCGGCGCCCGGCAAGGTGGGCGGATTCTCACGCGGCAGCGGCGCCCGGCAAGATGG
GCGGATTTCCACACGGCAGCGGCGCCCGGCAAGGTGGGCGGATTCTCACGCGGCAGCGGCGCCCGGCAAGATGGGCGGA
TTTCCACACGGCAGCGGCGCCCGGCAAGGTGGGCGGATTCCCACACGGCAGCCTCGCCCGGCAAGGTGGGCGGATTCCCA
CACGGCAGCCTCGCCCGGCAAGGTGGGCCGATTCCCACGCGGCAGCCTCGCCCGGCAAGGTGGGCGGATTCCCACACGGC
ACCGGCGTGCGGCAAGGTGGGCGGATTCCCACACGGCACCGGCGCGCGGCAAGGTGGGCCGATTCCCACACGGCACCGG
CGCCCGGCAAGGTGGGCCGATTCCCACACGGCAGCCGCGCCCGGCAAGGTGGGCCGATTCCCACGCGGCAGCCTCGCCCG
GCAAGGTGGGCCGATTCCCACGCGGCAGCCTCGCCCGGCAAGGTGGGCCGATTCCCACACGGCAGCGGCGCCCGGCAAGG
TGGGCGGATTTCCACACGGCAGCGGCGCGGGGCCAGTGGGATTCAGGAGAATAGGTGTTTTACCGAATGCCCTGACGAGG
CGTAAAAAAACCGCTTGGCGGCGGCCTCATAAAGCAGAAAACCCGCTCAAGGCGGGTTATCTGCTCTGTAGCCTGTGATGCT
TCGCGGGCATCCGGCATACAGCGAGGTGAAATTCTTCTTTTGGCATGTTAATTATACGTCTAACGCGGCATATGATCAAACCTG
TATTAAATAAGCCACTGTACCGTTTATAATGCTCTCAGATCAAAGAGGTAAAGCCCGTTTAGCCGCCTGTGTGATGAGCCAGTT
CAGACTCTTCAAAATCGAATTTGGTACTAAACAGGACCCGAACCGTGGGCAAGCACACGGCAACGGTATAGCCCTCTTCCGG
TTTCGCACCCGGAAGCCTGGGCGGCAGCGTGGTGAAATTCTTCTTTTGGTTAAGTGAATGGCATAACCGGATGGGCGGATTA
GAGGAAAGGGGATTGCCTAGTAACCTACGCGCCACAGAGATGGAGGTGCGGGGAATGATTGAGCTGATTATCGCTATTCTGA
CCTTAATTGCGGCTGTATTGCAGTTGATCAACTGGTTCCTTTAATGGTGCCGGAGTCTGTGAAGGTGAAAGCCTGAACGGGC
AAAACTGAAAGGTTTATAGCCGTCCTTCGGGGCGGCTTTTTTTCGGCAAAATTAGGGTTTTACCGAATAATGCAGAGTTTTAA
GGTGAGAATTTGCAGACTTGGCGTTTTACCGAACATAGATACTCCCTAGGCTGATAGGTGCATTAGTTATCACCCTACCTGAAC
ATATTGTAAAAGATGTCAGTCTCCAGTGACTTGTGTACTATCAACTGACAAGACTCTTACACGCAACGCAGGGGGATGGAGTT
TTATGCTTAGAAAAATAATCAGGGGTAGCGGATTCACTCAGTCAGAAGAAAAACTGATAGAGTTCGCTGATGATGCTTTTTTT
GGTCTTTGGTCTTATCCTAATGTTTATAGCGATGAGGGTTACTCTAAAAATAAAATTGGGAAAGAAGTTAGTGACTTATTAGTTA
TTTTTGATAAAGATATAATAATTTTTTCCGATAAAGCTATTACATACAATAAAAACAAAGATCCTAAGGTTGCATGGCAGAGATG
GTTTAAAAAATCAGTCATACAGTCTTGCACACAGTTATTTGGCGCAGAGAAGTTTATAAAAGATCATCCCGAAAGACTTTTTGT
TGACAAAGAATGCTCAGTTAACCTCCCCATTAAAATAGATAATTCTTTTAATTTTCATTTGGTGGCCGTCACTAATAATATTTCAG
ATCCGGCGATCTCGTACTTTGACAAAATAGAAAAAGGCAGCTCTGCTACTTTAGTTAACATATTTCCTTTAAACGCCCATCAATG
TCTAGAAAATCCATTTTGTGTCGGAGACGTTTATCCTGATAAGACTTTTGTCCATATACTTGATGAGACTGCCCTAAAACTACTG
TTAACCGAGTTAAACACAGCAACTGATTTCATTGGCTACCTTAACGAAAAAGAGAGGGTTGTAAGAGAAAGAACATTATTGG
TCAGCGCTGGGGAAGAAGAGACTCTTGCTGCTTACATTATGGGTGATAAAACCATAATATCAAAAGAAATTATTGGAAACGAT
CAAGGGATGACCATACCGGAAGGTGAATGGAAAAACTATAAAACCACTTTCAATTATCAATATCAGCTCTCAATGAAAAAGGG
TAGCGTTTTCTGGGATAACCTAATCCACAACTTCTCGACAAGTATATTGTCAGCTAACGTTGGTTTTTTTAGTGAAATTGAATTT
TCTACACATGAATTAGGTGTTAGAGAATTAGCCAAAGAAAGTAGGCAATCTAGATATTACCTTTCAAAGAACTTTAAAGAGAA
ATTAAAAACAACTCAGCCTCATCTAAGAACGTCAAGAATGGTCGAATCAATCGATGAGCCTGGAAAGTTTTACTTATTCCTTTT
TTTTCCTAACGATAGCAAGTTGAGTTACTCTGATTACAGAATTCAACGTATATCTTATATAAATGCTTATGCTGAGGTTGCCTTTA

ATAAATACAGACATATTAAAAAATTAATTACTATTGCAACAGAGCCGCAAAATACAGAAGGAAGATCTGAAGACCTAATATATA
GCATATCCCCAGAGAAATTTACCAAAGAGCAAAATGAAAAAGCCAAAAGATTATCAAGAGAATACAAAATACTAAGTGATTTT
TTACCTACTAAAACGACAAAGAGCGATAACTTTAAATCAGTTATATCAAAAGGTGAAAAAATAGGGCGGAATACACCTTGTCC
ATGTGGCTCCGGTGTTAAATTTAAAAAGTGCCATGGTGCGAATAATTAGCATTATTGTATGTATAACGGTAATGGCGCGGCAGA
GAAACCGGCGCGTTCTGCCCTAGTGTTGGCCTGCGGGTTCCCCCGCACCCGCTGTATGTAGTATCGGCAGCATCTGAGAAAA
CCACTACATGTAGTTATCAGCGCCACAACGGCGCGGGGACGAGTGCGGTTCGGAAAAAATTGGGGTTTTACCGAATCCGGCA
AAAGATTGCTTCCTATAACGTCCGCTTCTGGCACACAGCAGCCGTTAAGATGTAAGGCCTTACGCCAACTAAATCTAATGGGA
CAGATTTAGTTGGTGATGGTCAAGTAATCTGCAAACGGTCACCAAGTAAAATGCAAATGGGTAGTCAAGTCCGATGCAATTAC
GCACCCGGCAAGGTGGGCCGATTCCCACACGACAGCAGCGCCCGGCAAGGTGGGCGGATTTCCACACGGCAGCGGCGCCC
GGCAAGGTGGGCCTATTCCCACACGGCAGCGGCGCCCGGCAAGGAGGGCCGATTCCCACACAGCACCGGCGCGCGGCAA
GGTGGGCCGATTCCCACACGACAGCAGCGCCCGGCAAGGTGGGCGGATTTCCACACAGCACCGGCGCCCGGCAAGGTGG
GCCGATTCCCACACGGCAGCGGCGCCCGGCAAGGTGGGCCGATTCCCACACGGCAGCGGCGCCCGGCAAGATGGGCGGAT
TTCCACACAGCACCGGCGCCCGGCAAGGTGGGCCGATTCCCACACGGCAGCGGCGCCCGGCAAGGTGGGCGGATTCCCAC
ACGGCAGCGGCGCCCGGCAAGATGGGCGGATTCCCACACGGCAGCGGCGCCCGGCAAGATGGGCGGATTTCCACACGGCA
GCGGCGCCCGGCAAGATGGGCGGATTCCCACACGGCAGCGGCGCCCGGCAAGATGGGCGGATTTCCACACGGCAGCGGC
GCCCGGCAAGGTGGGCCGATTCCCACACGACAGCGGCGCCCGGCAAGGTGGGCCGATTCCCACACGACAGCGGCGCCCGG
CAAGATGGGCGGATTCCCATATCGACATGTATGTAGCTTGTGTTATCCGTGGATTGTGCAGCTCAGCGGGTCGCTTGTCGTATG
GCGTAGTGTCCCCCGTAACCGGCCGCGTGCGGCCGCTAACGCGCAGTACGGCGCCGCGACCCGAAGGCGGGCCGCCGTTCC
CGCGCGCAGGCGCGCGGCGCCCACTGCGCACCCCCGTGGGGGACGTGCGGCAGCTGTGTGGCGGTGAGCGGGATTAGGG
CTTTGCAGGGAGGGGGCTGGGTCGGGCGATACGTTCAGCATTGCGGTTTCCGGCGATTTGCGGCCGGTGCCCGTTTAACTC
CGGCGTGGTCGCCTTCCATGCCCTGACGGCATAAGAAAATAAAACCGCCATGCTGCGGTCATTCATGATTTTGTGGTGTAGCG
ATAAATAGTCATGCGAGAAACGTTGAAGCGCTTAGCAACTGCACCAACTGTCATTTCAGGATCAGCAAGTAAGATTCTAATTT
GTTTAACATCTTCTTCAGAAAGTGACGGTTTTCTCCCTCCCACACGGCCCCTTGCGCGTGCAGCTGCAAGGCCTGAGCGCGTT
CTTTCAATATTGCGGTTGCGTTCAAAGCTAGAGAATATCGCCATCAGATGAGTATAGATTTCCCCTATAACTGGCGCATTTGTGT
CTATTCTGTCCTTGATGGCTATGAAAGTTATTCCGCGTTTCTTCAGGTCGTCGAGTAAAGTAATGACTTGACCCAATGAACCAC
CGAGCCGATCTAGTGCCCAAACTACTAGGGTATCTCCCTCGCGCAATGCTTTCAGGCAGTTCTCCAGTTCCAGCGCACCTTTTT
TGTCGCGCTTTGGGCCGCTACGTGAGGTCTGATCCTGATAGATTTGCTCACATCCAGCTTTTGTTAGTTCGTCAACCTGGTGCG
CCACATCCTGAAGATGCGTAGATTTACGTGCATAGCCGATTTTCATTCTTTTCTCGCTAATTAGTTATGGGGTTATTGTTATGTTG
ATACAGTAACGAGTTTTGTTACATGAGGGGAGTCATTTTTCGGGAGAAGTCAGGACTTTTCAAGACTGTCACAAAAACCATC
GTTTTTGATACATTAATTTAACCAATAGGTTGCAGATCAAATCGTCTGTAACAGCCTTTCTGGCTGTTTGTATATAATCATGAAAA
AATGGTGAGTAGAGTTTCAGGGTAACAGGGGATGCTTATGTCGGTTTTCCACAACTGGCTACTTGAGATCGCATGTGAGAATT
ACTTCGTCTACATCAAACGCCTTTCCGCCAACGATACCGGCGCAACAGGTGGTCACCAGGTAGGGCTTTATATCCCTTCAGGT
ATCGTTGAAAAACTCTTTCCGTCTATCAACCATACCCGTGAACTGAACCCTTCGGTTTTTCTCACCGCACATGTGTCATCGCATG
ATTGCCCTGACAGCGAAGCCCGGGCAATTTATTATAACAGCCGTCATTTTGGTAAAACCCGGAATGAAAAAAGGATTACCCGC
TGGGGTAGAGGCAGCCCACTTCAGAATCCTGAAAATACAGGGGCTCTGACGCTCCTGGCTTTCAAGCTTGATGAGCAAGGG
GGGGACTGTAAGGAAGTAAATATTTGGGTATGCGCCAGCACTGATGAAGAGGACGTCATTGAGACCGCTATTGGTGAAGTTA
TACCCGGAGCGCTTATATCCGGCCCCGCAGGACAGATTCTAGGCGGACTATCTCTACAGCAAGCGCCAGTAAATCATAAATATA
TTCTACCTGAAGACTGGCACCTGCGCTTTCCGTCGGGAAGTGAAATTATTCAGTATGCAGCCAGCCATTATGTGAAAAATTCCC
TTGATCCGGATGAGCAACTTCTTGACCGCCGGCGCGTGGAGTACGACATATTTCTATTGGTTGAGGAACTGCATGTTCTGGAT
ATCATCCGGAAAGGATTTGGCTCTGTGGATGAATTTATTGCGCTGGCCAATTCTGTCAGCAATCGCCGTAAATCCAGAGCCGG
GAAGTCTCTGGAACTGCACCTGGAGCATCTATTCATTGAGCACGGCCTGCGACACTTTGCGACGCAGGCCATCACAGAAGGT
AATAAAAAACCCGATTTCCTTTTCCCTTCCGCAGGGGCTTACCACGATACTGAGTTTCCCGTAGAAAATCTGCGCATGCTGGCA
GTCAAGACTACCTGTAAGGATCGCTGGCGTCAGATACTGAATGAGGCCGATAAAATTCATCAGGTGCATCTGTTTACACTCCA
AGAGGGAGTTTCTCTGGCTCAATATCGGGAGATGCGGGAGTCGGGTGTCAGATTGGTCGTGCCATCATCGCTGCACAAAAAA
TACCCGGAGGCGGTGAGAGCTGAGCTAATGACGCTAGGTGCGTTTATTGCTGAGCTGACAGGGCTTTACGCAGATATTCCAT
AGATTATCTCCCGGCATAAATACCGGGAGGAGCGATCAGATTCGTTCAACCTTGCACGAATCGGCATTAACCGCTTTCAGGAT

ATAAGGTTCAAGCAGTTTGGCTACGGCTTCAAACACGGGCACCACTACGGAGTTACCGAACTGCCTGTACGACTGAGTGTCT
GACACAGGAATGCGAAAAGGCCTGCCATCTACTTTTTCAAAACCCATAAGGCGCGCACTCTCGTGGAGTCAGCCTGCGGG
GCCGATGCGCCTGATTTCTTCGTTCGCGAAGTCTGTTTCACCTGTGGCCATATCCCAGCCACGGTCTATCAGAATTTCAGACC
CGTCTTTGTGATAGCGAGCAGAAAGCGTACGGGCAATGCTTTCTTTATTTTCAGGATTAACGAGGCCAAAACCGAATCCGTTA
CCCTTAGCTGCGTGCTTTTTGGCGTAGTTATAGAGATACTCCCAGAGTTTCGGCGTCAGTATATATTTGCTGTCAACCACGGGT
TCCAGCAGTTCGCCAAATGACGGACGCTGTTCCGGATAAAAACGACTAATATCGCGCAGGGTAAAGCCCTGGTGAATGTTCA
GATCACGACGGAAACCGACCAAAACGATACGTTCTCGGTGCTGAGGTAAAAAGTGCTTTCCGTCGATAACTTTAGGATCGTTT
TTGCCCATCTCAGCTGCATCCGCAACTTCATAGCCCAGTTCGTCGAGGGTATCCATGATGACTTTAAAGGTTTTACCCTTGTCAT
GGCTCTTCAGGTTTTTAACGTTTTCAAGAACAAAGATGGCAGGTTTTTTTGCGCGTATAATACGCGCCACATCGAAGAAAAGC
GTTCCCTGAGCCTCACATTCGAAACCATGCGCGCGCCCGAGCGAGTTTTTCTTGCTTACGCCCGCAAGGCTGAACGGTTGAC
AGGGGAAACCTGCTAGAAGTACATCATGATCCGGCACATGCTCATTAATGTAAGCATAGGCATCGTTTTCAGGTACTTCAGGTT
TATCACTGAGCGTGACTTCCCGAATATCGAGATTGAAAGTGTGTTCCTGAGCATCGTTAAACCAGTTAGCTTTATATGTGCGCA
CAGCCTCTTTATTCCATTCACTGGTAAAAACGCACTGGCCACCGATGGTTTCGAAGCCCTTCCGTATACCTCCAATCCCAGCAA
ACAGGTCAATAAACCGGAAGGCATAGTCAGGGTGATGTGCAGGCGCTTCCGGAAGCATTTTTCGTAGAAGTTCCTCTTCGGC
TAACGTCAGCGTCTTAGGTGAGCACTTACCATTAATCCAGCGATTAAGAGTCTCGCGACTCCACTCATTTTTACCAACTTTTCTA
AGCAGTTCAGCCACGTACTTCTGGTCATAGATTTCCAGCACCTGCCCGAGCAGCTTTTTATAATTTTCCTGTCGCAGTTGTTCTT
CCGCTTCTGCTTTCTCAAGCAGATCCTGCGCCAGTAATTCAAATTCAGACATATTGCCTCCATTGGGTCTTATGGGTGAAACTG
TATCACTCATTTGACCCAGATTGAATGTTTTTATCTGGATATTTAAACAGGTTTATTGTTAGGTAACGCACGTTGGCCACGCTGG
AGCGTCTTCTGGGCCTGCTGTCGGCCTTTGAGGTCGTGGTATGGATGACGGATGGCTGGCCGCTGTATGAATCCCGCCTGAA
GGGAAAGCTGCACGTTATCAGCAAGCGTTACACTCAGCGCATTGAGCGACATAACCTGAATCTGAGACAACATCTGGCAAGG
CTGGGACGGAAGTCACTGTCGTTCTCAAAATCGGTGGAGCTGCATGACAAGGTCATCGGGCATTATCTGAACATAAAACACTA
TCAGTAAGTTGGAGTCATTACCGGTTCTCTTTGTCTTTTAGTGATTCTATAAACCTCATTACGTCTGAATATAAAAATCTATTATTT
GATTTATGTGGCTCATGAGGTTGTGGGATGGTCTTGTTTTTGAATGTGCCAGTTTTCTTAATGGCAAAGATTAATTCACCTTCT
GTTATTCCTAACATTTCAGCAAATGTTTTTGCTTCTATAGTTACTGACTTCATTTAATTAACTCTCATGGTATCGATTTTCTTTACC
GGCATCTTTAACAATGGTGCTCGTTTCTAGTGTTGCTGCGGTACGCTTCATCATCGTCTGCGGGGCGGTTGCGATAGTGAAGG
AGCTGCCGGGCGTGAGCAAATCTATCAGGCGCTGGCCGCTGATAATCTCCATCCGTTCACTGGCAATACTGACAGATTTTGAA
CCTGCGCCGGTTTTCCCGGTATGGCAAAACAGACCGCGACAGTTATGACGTTTAAGCAACTTCTCGAACTCCTGTACGTGCTG
TAAAGCAATATGGCCGCGATAGCGTTTAGCCTGAATAAGATAGCGATATTTTCCTATTATTACCTGGCCGTCAATGCCTCCATCG
CCGGTATAGCGTTTGTTTCTGATGGTTCTGAAGCCATGCGCGGCTTTGTTGAATAAATCGAACTTTTGCTGAGTTGAAGGATC
AGATCACGCATCTTCCCGACAACGCAGACCGTTCCGTGGCAAAGCAAAAGTTCAAAATCACCAACTGGCCCACCTACAATAA
AGCCCTCATCAACCGTGGCTCCATAACTTTCTGGCTGGATGATGAAGCTATTCAGGCCTGGTATGAGTCGGCAACGCCTTCATC
ACGGGGAAGACCTCAGCGCTATTCTGATCTCGCCATCACCACCGTTCTGGTCATTAAACGCGTGTTCAGGCTGACCCTGCGGG
CTGCACAGGGTTTTATTGATTCCATTTTTACACTGATGAATGTTCCGTTGCGCTGCCCGGATTACACCAGTGTCAGCAAGCGCG
CAAAGTCGGTTAATGTCAGTTTCAAAACGTTCACCCGGGGTGAAATCGCGCATCTGGTGATTGATTCCACCGGGCTGAAGGT
CTTTGGTGAAGGCGAATGGAAAGTCAAAAAACACGGCAAAGAACGCCGTCGTATATGGCGAAAGTTGCATCTGGCCGTTGA
CAGCAACACACATGAAATCATCTGTGCAGACCTGTCGCTGAACAATGTGACGGACTCAGAAGCCTTCCCGGGTCTTATCCGGC
AGACTCACAGAAAAATCAGGGCAGCATCGGCAGACGGCGCTTACGACACCCGGCTCTGTCACGATGAACTGCGGCGTAAGA
AAATCAGCGCGCTTATCCCGCCCCGAAAAGGCGCGGGTTACTGGCCCGGTGAATATGCAGACCGTAACCGTGCTGTTGCGAA
TCAGCGGCTGACCGGGAGTAATGCGCGGTGGAAATGGACAACAGATTATAACCGTCGCTCGATAGCGGAAACGGCGATGTA
CCGGGTAAAACAGCTGTTCGGAGGTTCACTGACACTGCGTGACTACGATGGTCAGGTTGCAGAGGCTATGGCCCTGGTACG
AGCGCTGAACAAAATGACGAAAGCAGGTATGCCTGAAAGCGTGCGTATTGCCTGAAAACACAACCCGCTACGGGGGAGACT
TACCCGAAATCTGATTTATTCAACAAAGCCGCCATGCGCTTCAAATCCTTCCAGCAACAGTTCTTCAAACACAAAAGGATCAAT
TTTCCTCAGGTAGTTAATTTTTTGTGGGAAGCCCGGCAACGTCTTTATGCGCTCCAGCACCCGCCGCGCACTTTGCTGCTTCCT
TTTGTGTCGTCGGTTGCGTACTGAACGCCGGAAGAATACAACGGCAAACAGTGCGATGGCGCTGCAAGCCCATAGAATAAGG
TTTTCTGTAGTGGGGAAGGGGAACATGGTGATAGTGTGCTTTCTGTGGGTAAAGAAAAGGGCGGTTAAACCGCCCTGGTGT
TTAGCGACGGCTGTAAACCTGCCACGAAGCGCTGCCTGACTGATTTTGGCAAATCCGCCCGTAAAGTACGGTGCCGGTCGAG

TAGCGGGCGCCATTCAGATAGCAATAGCCGCTGGATTGCAGCAGTTCGTTGCGCAGCTTTTCTTCCTGCTTTGATAGCCGGGA
CTCCGCAGACTGCAAACGAACGGATAATTCGTTAATCTGGCGTTGCTGGTTATTCATCTGGCTCTGCATCGCATTACCTTTATCT
TGGCTGACGCAACCAGTTAAGAGAGCTGTACAGGCTAATGCACTTAATATAATTTTTTTCACGTTGGCTCCTTAAATTGAGATT
ATTCCTAGCCCGCCTATAAGCGAACTTTCCCGTATTTACTTATGATCTGGCTTATCATCGACTGGTTACTTCCACCTTCGCCATTAT
CCGGGCATTCATTAAGAAAAGCCTTCCTGGCATCCCTCGTGTGGTTGGGTAAAAAGCCGTGCTTGTTCTTTTTAACGATATTGA
AGAAAGCAGCTTCAGCACTGTTACACTCGCTTCCGCCGCTATCGCCGGTGAGCTTGCCCGCCATGCACATAATAACTTTGCAG
GGATCTTCAGCATGGCTGGCAGGAAGATAAAGCAGACTACCAGCTGCTATCAGAGGGATTAAGAGTTTCTTCATTGTTTTGTC
CTTAACAGTTTGTTCAGATATACACCCGCCAGAATGTTGATAACGGTAAGTAATATTAATAATAACGCAGAGTTATAAATAGATTT
GTAACCTATATCGCCTGCGATATATTCGACAATAAAAACGAAAATCGTTAACATTGCGAACAACTTAATTAACTTTCCTGTAATTT
TCTTAACGATATAAGCAACTGAATAAGAGCCGGATTTGATGATGCTGGCGAGTAACCTGATAGCATGGACTCGGAATTTTCTTA
AAATGGGCGGAATGGAAAATGCCTTTTTCTTCCTTCTGCCTTTTTGCTTTTCCGCAAAATCAATAACATCGCCCATTTTTATACT
CTCCGGTTATATCTTTAGATCATCAAGTGATAAACCACTATCAAGCAGCTCTTTGAGCCATGTTGGTCGGCGGCCAGTCCCGGA
CCAGGTATTTTCTGCGTTCCTGGGGTCGCGGTATTTTACTTGTCTATGTTCTCTGGGGTTGGCAACGCCCTGCATTTTCATGGC
TTCCAGGCTAATGCCAGCGTCCAATTTTGCAGTTAGCCAGGCCGGGCGCTTCCCTATACCAGTCCACGTATTAAAAGGGTTATC
CGGGTCACGATACATGGGTTCACCTTTGGGGCGTTTTTCTGATTTCAGGGGAACCAGCATTACCTTCCTTCCTTCAGAGGTTT
TCTTTTCGTTTCTTCTTGACACGATCTCATTAGCCCTTATCATAAACATAAGTCGTCGAAATTACAATTACGCGGTGAAACGTAA
ATGAGTAAACATCCAAAACTTCTGGTTCTCGCTCTGGCCTGCCTTGCTTGTGCTGGCCGTGCCAGTGCTGCGCCTGCCTCAGA
TGAAGTTGCCAGGCTTGCGCAGAGATGTGCGCCTGATGTTTCACCCTTAACAATGGCGTACATCGTCGGCCATGAGTCCTCAA
ATGGGCCGTACAGGATCAATATTAACGGTGGTAGTACCCAGTTAAAACAGCAACCACGTACTGAAGCTGAGGCCGTCAGCGT
TTCGAAAGTTCTGCTGAAGGATAATAAAAGTTTTGATATGGGCCTTGCACAAATTAACTCAAATAATTTAGTGGGCCTGGGTCT
TTCGGTTGACGATATTTTCAAGCCCTGCATCAACCTGCGGGCGAGCCAGACCATCCTTAAAGCCTGTTATGATAGCGCCCTGA
AATCCTATCCAGCCGGGCAGGTTGCGCTGAGACACGCGCTTTCCTGCTACAACACCGGCTCACTCATAAACGGGATTTCTAAC
GGGTATGTCACGAAAGTTATCAACGTGGCGCGTCAATCAACTGATTTGAAAATCCCTACGCTGCTACCTGATGGCCAGACCAG
TGAGGACAGCACCGCGACTGAGCCTCAGCAGGCAAAAAGTACGGCCACGCAGTATGACGGTGAACAAGATGTTTTTGGTTC
GGGTGATGGCGATGCCTTCAGCCGAAATAATACGGATGCCTTTTTAACCAAACAGGAAACAGCGAAGGGGGAGTGAGGTTA
TGGATGGAACGTTTGTACCTTGTATTGCAATCACAGATCCTGGAGCTATTTGAAACAGGAAAAGTGAAGGAGGTAACGATAA
AACGGGTTTCATTAAAGACGTGGTATCCCGTTTTTCAGATAGACGATGAACAGTTGGGCCAGATCGCATGTTCCATTCGGGTT
AACAAAGAGCATGAACTACGAACCTGGGCTGATTTAAGGCTACTGGCAGAGTTTTTGAAAGATAAGTGTGGCGTTGAAGAAT
GCCGGTTAAATCTGCAATCAACAGAAGATAGTGAGTAAGGAGAAAGTATGACCACGTTGTTTAAGAAGTATGGCCCTGCGGT
AGTTATGGGCGTTTTGTCCATTGCCCTGCCGCAAATTGCGCTGGCCGCTGGCACCGATACTGGTGAATCAACCGCTACATCAA
TCCAGACGTGGTTGAGCACATGGATTCCAATTGGTTGTGCTATTGCGATCATGGTTAGTTGCTTTATGTGGATGCTTCACGTAA
TCCCAGCCAGCTTTATTCCTCGTATCGTAATCTCGCTGATTGGTATTGGTTCTGCATCATTTCTGGTTTCCCTGACGGGCGTAGG
AAGCTGAACAACGCGAAAAGGGGGGGACTTTTGTCCCCCAAAGTGAGGACTACAAAGATGTTCGTTGACGGGAAAAGACC
GCTTTTCAAAGGTGCGACTCGCTTACCTCGCGCGCTGGGTGTACCACGTAATGTAGCTATGATGATATTCATGATTTCTGCCTC
GCTTTTTATGATTATTCATATGTGGGCGATCCTGGTGTTCGTCTTTTTGTGGATTCCTTCAGCTGCATTAACAAAATATGACGACC
GCATGTTTCGAATTATGGGCCTGTGGTTGAAAACCAAATTCAGTAATTGGTTTGATTCTCCGTTTAAGCAGTGGGGAGGATCG
TCTTATTCCTCTGTTGACTACAAACGTAAGGGTTTAAAATAATGAGAGCTGCCACCGCTACGAAGCCAAAAAAAATTGATGCCT
ACCGTAAGGAGCCATCAGTAAATAAAAAGTATTTGCCCTATTCTTATCACCTCAATGATTACGTGATTTCGATGGAAAACGGCG
ATCTGATGGCTTTTTTCAAGCTGGATCGCCGCACACATGACTGCGCATCAGATCGGGAACTGGTCACCTGGCATAAAGACCTT
AATACGCTGGTCAAGAGCTTCGGAACAGACCATGTAGAGCTGTGGACGCATGAATATCACCATGAGGCTAAAGAGTACCCGG
ATGGTGAGTATGACCATTTTTTCCCTGCTTATGTTGATCAATATAACCGTAAGCTGCACGGTGATTCCAAGCAGCTGATTAATGA
CCTTTATCTGACCGTTATTTACAAACAGGTAGGGGATAAAACACAGAAGTTTCTGGCGAAATTTGAAAAGCCGACTCGTGACG
AAATTCAGCGAATGCAGAATGAGGCGCTTGAAGGTCTGGAAGATATTTCTGAACAAATCCTGGAAGCAATGAAGCCGTATGG
CATTCAGCAGTTGGGTATCTATTATCGTGACAAACGCGGTGTTGAAATTCCTGCGCCTGATAAAAAAGAACGTGAAGAACTTG
CTGAAGTCGATGAATCAGACATTTTTGACGAAGCCATTGTTATCGAACGCAACGAGCCTGAACCTTCGCAGGCTCACGCTTAT
TCAAAAGCGCTGGAGTTCCTTTATTTCCTCGCAAATATGGAATGGGCCATCGTGCCTGTTTGCCGTGATCGTATCCGTGAGTAC

ATCATGGACAACCGCCCTGTTAGCTCACTGTGGGGGGATGTTGTCCAGATCAGAACGGTAGATCACAACTTCTATACCACCGG
CATTGAATTTCGTGAATACGAAGAAGATACAGAGCCAGGCCAGCTTAACATGCTTAAAGAAGCCGATTTTGAATACCTTCTGA
CGCAGAGTTTTTCTTGCCTCTCTGAATCTTCAGCTAAAACGTTTCTGACGCATCAGGAAAAATCTTTGCAGGAAACGCGCGAC
CGTGCGCAAAGCCAGCTGGCACAGCTTGGTACCGCGCTCGATATGCTGACGTCCAGAGAGTTCGTGATGGGCTACCATCATG
GAACCGTGCATGTCTGGGATAATGACCAAAACGCGGTACAGCGCAAAGCGCGTCGTGTGAAGGTTATGCTAACCGGCTGTG
GCGTGGTTGGCGGGACTCTCAGCCTGGCCTCTGAGGCTGCATATTATGCGAGACTGCCTGGCAACCAGAAATGGGCGCCGC
GCCCGGTTCCGATAAACTCATGGAACTTCCTGCACTTCAGCCCGTTCCACAATTTTATGCGTGGCAAGCCTGACAATAACCCGT
GGGGGCCAGCGCTGACCATGTTCCGCACGATCAGCGGTACGCCACTCTATTTTAATTTCCATGTGACCCCGCTTGAAGAACTT
TCCTACGGTAAACGCCCGCTGGGCCATGCGTTAATAACGGGTATGTCGGGGGAAGGTAAAACCACGCTGCTTAACTTCCTGCT
GGCGCAGTCAATGAAGTACAACCCGCGGCTTTTTGTTTATGACCGTGACCGCGGTATGGAGCCGTTCATTCGAAGCGTTGGT
GGCTACTATAAAGTTCTGCAACAGGGTATGCCGTCCGGGTTTGCCCCGCTTCAGATTGAACCGACCAAACGCAATATTGCCCT
CATTAAAAACCTGTTCCGCATTTGTGTGGAAACCACCAATAACGGGCCTATCAGCGCAACGATGGCTACCGAACTGGCTGAAG
GCGTTGATGCGGTTATGGGGGAAGGCTCACTTATTCCACGCGAGGCGCGCACCGTTACTATCCTGGACGGGTACGTGAATGA
AGTTGTGGAAAATGGCGTATCACTGAAAGGGCTGCTGCGCGAATGGACGCGCGAAGGCCAGTATGGCTGGCTGTTTGACAA
TGATAAAGACAGCCTGGATCTCAGCGCGAATGATATTTTTGGCTTCGATTTATCCGAGTTTATCGCAGCCAAAGAGGAAGTATC
CAGCCCGGCCCGTACTCCGCTCATGATGTACCTTCTGTACCGGGTACGTGACTCCATCGACGGCAAACGCCGCGTCATTCAGT
GCTTTGACGAGTTCCACGCCTACCTTGACGATCCGGTTATCGAGCGTGAAGTTAAGCGTGGTATCAAAACTGACCGTAAGAA
AGACGCTATCTATGTGTTTGCCACGCAGGAGCCGAACGATGCGCTGTCCAGCCGTATTGGCCGCACGATCATGTCGCAGACCG
TCACAAAAATCTGCCTGCGCGATCCGGAAGCTATCCGAGAGGATTATGCCTTCCTTACTGATGCTGAATACGACGCGCTGATGT
CGATTACCGAACACTCCAGACAGTTCCTGGTTAAACAAGGGCAACAGTCTGCGATTGCTTCTTTCAATCTCTACCCTCGCAAC
AGCGACGATATTGATGCAGATATTAAGACAATGGACAACGTTCTTAGCGTGTTGTCCGGTGAACCACAAAACGCCGAAATTGC
GCATGAGCTGGTTGAACGGCTCGGTAATGACCCTGAAGTATGGCTCAAAGAATACTGGCGCCTGACGGCTTAACAACGAGGC
AAAACACCATGAAAAAAACACTGACGGCAGTATTGCTGACCACCGGCCTGATACTGGGAGGCGCGCAAAGCGCTTCCGCAG
GCATCATCGTGACCAACCCTACTGAGCTGGCTAAACAGGTCGAGCAGCTTCAGCAAATGGCGCAGCAGCTGGAGCAGCTTA
AAAGCCAGCTGCAAACGCAGAAAAATATGTATGAGTCGATGGCAAAGACAACCAACCTGGGCGATCTGCTGGGGACGTCTA
CCAGCACGCTGGCAAATAATTTGCCGGACAACTGGAAGGAGATCTACAGCGACGCCATGAACTCCAGTTCTTCCGTCACGCC
TTCAGTTAACAGCATGATGGGCCAGTTTAATGCGGAAGTTGACGACATGACGCCCAGCGAAGCAATTACCTACATGAACAAA
AAGCTGGCTGAAAAAGGCGCTTATGACCGTGTTATGGCAGAAAAAGCCTACAACAACCAGATGCAGGAACTAACCGATATGC
AGGAGCTGACGGAGCAGATTAAAACGACTCCAGACCTGAAATCGATTGCTGACTTACAGGCCCGTATCCAGACGTCACAGG
GTGCTATTCAGGGTGAGCAGGCGAAGCTGAATCTGATGAACATGTTGCAGCAGTCACAGGACAAGCTATTACGTGCGCAGAA
AGAACGTGCCACCCACAATTTTGTTTTTGGAACCGGCGGGGACGTTACCGCGTCACCTTCAATTAACTGAGGTAATTATGAAA
AAACTACTGCTTGTTATCCCTTTCCTCCTAGTGGCCTGCGATGCCTCGCATGACGTGGAGTGGTACAAAAAACATGAGAAAGA
GCGCAAGGCAACAATTCAGGAATGCAAGAAAGACGCGGATGAACTTCAGAAACCTGATTGCAAAAACGCGCGCGAAGCCG
ATCGTCAGCTGTTTGTGTTCGGCAAAAAAGACGGCGAAATCAATTCACCGAAAATTTAGGAGTAAGGAGGCAATATGGCATT
CACCCTAGTCGCAGACATTTTCGCAAAAGTAGACGGGGCGATTACGTCAATGGTGAGCGCCAATGTTGCCACCATTATCTCTG
ATGTAACGCCTCTGATTGCCACCTGTCTGACAATCAAGCTGATGGTTCAGGGGATGTACTCAGCGTTTAATCCGGGGGCGGGC
GACAGCCTGAGTTCGCTGATTAAAGAGTATCTTTCCATAGCCCTTATCCTGAGCTTTGCAACGGCGGGCGGCTGGTATCAACA
GGAACTGGTCAACGTGGCGCTTCACCTGCCGGATGATTTTGCCGGGATACTGTCTGCCCCTAATAAAGTCGGTGCAAGTGGC
GTACCGGCGATTATTGATAGCGGTATTGAAAAAGGTATCAAGATCGTCAACACCGCATGGGAAGCCGCAGACGTGTTTTCATC
GAGCGGCCTGGCCGCGTATGCCATTGGCGGCATTATGATGATTGCTACCGTTGTGCTGGGCGGCCTCGGTGCGGGCTTTGTG
ATCATGGCTAAGATCCTTCTGGCCGTTACGCTTTGTTTTGGCCCGATTGCAATCTTCTGCCTGCTGTGGGGAGCGACAAAAAA
CATCTTTGCTCGCTGGCTGGCGTCGGTCATTAACTATGGCCTTGTCGTCGTCATTCTTGCGCTCGTGTTTGGTTTCATCATGCAG
ATGTTCGACAACCTCCTGTCCTCGATGAACTCTGATGCCGCTTACTCATCAATCACTGGTTCTATCTCCGCCTTATTACTGACGGT
CATTTCCGTTTTCGTTCTGTTCCAGATTCCGCAAATTGCCGCCAGCTGGGGTAGCGGTATCAGCGCCGGAGTTGCTGACGCCG
CACGCTCTACGGGTTCTTCCATGCAGGCGCTTGGCAATATGGGCAGCCACGGCATGTTTGGCGGTAATGCGTTCAGAGGCGG
TAACAGTGGCGGCGGCCAGCAATCGGCAGGTGGAGGAAGTGGCAGCAACAGCGGAGGAAGCAGTGGTTCTAATTTAAGTG

GTAAGGCAAGGGGCAGTCGCGGGAAGAAGGCTGCATAAAATTAAAACAAAAGTCGTTGAAATTGCAATTTCGACGACTTAT
TATAATTAGTACGTTCAACAACCGATAATGGATGCCGTAATGCGCAGCTTATTGCTTATGGGAGTTCTTCTGATTAGCGCCTGTT
CCAGCGGGCATAAACCGCCACCGGAGCCGGACTGGAGCAACACCGTTCCAGTAAACAAAACAATCCCGGTTGATACGCAAG
GTGGTGCAAATGAAAGCTAATAAAAAAACAGGGCTTACACGTGAAGCCATTAAAGAGTTCAACGAAAGCCGTAAAGGGCTT
GAAGTTGATCTGATGGATGAAGTGCTGAAGTCCCGGCGTACCGCCTGGATGGTTGCCACCGGTTCAGCGGTGGTAACTGTTT
TTGCACTCTCTTTAGTTGGTTACGTGGTGCATAAGTACAGCCAGCCAATCCCCGCACATCTGCTAACGCTCAACGAGGCCACTC
ACGAAGTACAGCAGGTCAAGCTGACCCGCGACCAGACCTCTTATGGTGACGAAATTGATAAGTTCTGGCTGACACAATATGTC
ATTCACCGTGAGAGCTATGACTTCTATTCAGTTCAGGTCGACTATACGGCCGTTGGCTTAATGTCCACGCCGAACGTGGCAGA
GTCTTACCAGAGCAAGTTCAAGGGCCGCAACGGTCTTGATAAGGTTCTGGGCGACAGTGAAACGACCCGCGTGAAGATTAA
CTCTGTGATCCTCGATAAACCGCACGGCGTAGCAACGATACGCTTTACTACGGTTCGCCGCGTGCGCAGCAATCCCGTTGATG
ATCAGCCGCAGCGCTGGATTGCCATTATGGGGTATGAATATAAATCGCTGGCGATGAATGCTGAGCAGCGTTATGTCAACCCG
CTGGGTTTCCGCGTGACGAGTTATCGCGTCAACCCTGAAGTTAACTGAGGGCTGCCCCATGAAAAAACTACTTCTTTCAGCAG
TCGTTTTGTCAGTCCTGGGAGGCGCGGCCACTAACGTTATGGCGCTTGAGGTTGGCCGCAATTCTCCTTATGACTATCGCATTA
AAAGCGTTGTTTATAACCCTGTTAATGTGGTCAAAATTGACGCTATCGCCGGTGTGGCTACCCACATTGTTGTCGCGCCTGACG
AAACCTATATCACTCATGCTTTTGGCGATTCTGAAAGCTGGACGTTTGCGCACAAAATGAACCATTTTTTTGTGAAGCCGAAA
CAGGCCATGAGTGATACCAACCTGGTGATCGTCACCGATAAGCGCACCTATAACATCGTCCTCCATTTCATCGGTGAAGAAACG
AAGAAAAATGCAGACGGTACGGTATCAAAATCCTTTATTGAAACGCCGTGGGCTGTGCGCCAGGCCGTTCTTCAGCTGACCT
ATGAATATCCGTTTGAGCAGCAGGAAAAAGCCAAAAGCGCGGCTGATAAAAAACGCATTACGCAGAAGCTGAAGCAGACGG
CTTTTGCGGGGGCGAAGAACTATCAGTACGTAATGAGCGAACAGCCTGAAATGCGCAGCATCCAGCCGGTTCACGTCTGGG
ATAACTACCGCTTTACCCGGTTTGAGTTTCCGGCCAATGCGGAGTTACCGCAGGTCTACATGATTTCGGCCAGTGGCAAAGAA
ACGCTGCCTAACTCTCATGTTGTGGGTGAGAACCGCAACATCATCGAGGTGGAAACCGTCGCTAAAGAGTGGCGTATTCGTCT
GGGCGATAAAGTCGTTGGCGTTCGTAATAATAATTTCGCGCCGGGCGCCGGTGCGGTAGCAACCGGTACGGCTTCCCCGGAT
GTGCGCAGGGTTCAAATTGGGGAGGATAACTGATGGCCCGTAAAAGTGTCGATGTAGATCAGGAACTCGATGAAAACACCG
GAGACGGTGAATTCGAAAGCGAGCGTGGCGGATTTAAAGGCAGTAACCGCCGTTCGGCTCCTGGTATGAAAGCCTTTGTCAT
ACTGATGGCGCTGCTTGCTTTGGTATTCATCGGGATTACGGTCATGGGTAAAATTCGCACCCCGGCTAAAGCTGAAGCTGATA
AAGACGGTGGTAAAGCGCAACAGGCCAATACACTGCCAAACTACAGCTTTAACAGCGATCCTGATGTTAATAAACCTGCAACT
GCGCAGAATAGCGCCACTGATGCCCGTGCTGTGCAGGCTGCCGCACAGGCAGATGCAGATGCGGGCAGCAGCAATACCGCC
GCGCGTACCTCTAATAAGCGTAAAGAACCTTCGCCTGAAGAACTGGCTATGCAGCGTCGTCTGGGCGGCGAGCTGGCCCAGA
CTAATCAGGCGGCTACAAGCAATAGTCCCGGAGTGCAGCCCCAGGACAACGAAACAAGCGAAGGTAGTTCAGCACTCGCTA
AAAACCTGACTCCTGCAAGGCTGAAGGCTAGCCGCGCTGGAGTCATGGCTAATCCCAGCCTGACTGTTCCGAAAGGCAAAAT
GATCCCCTGTGGTACCGGCACCGAGCTGGATACCACTGTTCCGGGTCAGGTTTCCTGCCGGGTTTCACAGGACGTTTACTCA
GCTGATGGACTCGTTAGGCTGATTGATAAAGGCTCATGGGTTGACGGGCAGATTACCGGTGGTATCAAAGACGGCCAGGCG
CGCGTGTTTGTTCTCTGGGAGCGTATCCGCAATGACCAGGACGGGACAATCGTTAATATTGACAGTGCCGGAACGAACTCAC
TCGGCAGCGCGGGGATTCCGGGCCAGGTGGATACCCATATGTGGGAGCGTCTGCGTGGTGCGATCATGATTTCGTTGTTCTC
TGACACCTTAACGGCGCTGGTTAACCAGACGCAGAGTAATAACATTCAGTACAACAGCACAGAAAACAGCGGTGAGCAGCT
GGCGTCTGAAGCACTCCGCTCTTACATGTCTATCCCCCCTACCCTCTACGATCAGCAGGGTGATGCGGTGAGCATTTTTGTTGC
CCGCGACCTCGATTTCAGCGGCGTTTATACGCTCGCAGACAACTAAAAAAGTGGGCGCTTAGCGCCCGCTTTTCTTCAGGAG
TAATCATGACTGATGCAGCTTTCTATCAACTTGGCCCACTGCGCGAGTATTTAGAAGATCCTACTGTTTTTGAAATTCGCATTAA
CTGCTTTCAGGAAGTTATCTGTGATACGTTCAGCGGCCGCAGGGTTGTGCAGAACGCGGCAATTACGGCAGATTTTATTAGG
AACCTTGCTAAATCGTTGGTGAGCAGCAACAAGCTGACCATGCAGGCCATTAATGACGTGATCCTGCCTGGCGGGATCAGGG
GCGTTATCTGTCTGCCCCCTGCGGTGATTGACGGTACAACGGCCGTAGCGTTTCGTAAGGATTTGGCGGCCGATAAAAATCTG
GAGCAGCTGACCCGCGAGGGGATTTTCAGTGACTGCCGGAAGATTACCGGCAGCAAGCAAAGCCTAACGGATGATGATTTT
TTCCTTAAAGAGCTGCACAGCAGCGAAAAATGGCCCGCATTCCTGCAAACCGCCGTTGAGAAGAAACGCACTATCGTGATCT
GCGGTGAAACCGGGTCGGGGAAAACGGTACTCACGCGCGCGCTGTTAAAATCGCTACATAAAGACGAGCGTGTAATTATTTT
AGAGGACGTTCACGAAGTCACGGTCGATCACGTTGTAGAAGCCGTTTATATGATGTACGGCGATGCAGGAAAGATCGGCCGC
GTCAGCGCCACTGATGCCCTGCGAGCCTGTATGCGTCTGACACCGGGCCGTATCATCATGACTGAGCTTAGGGATGATGCTGC

GTGGGATTATCTTAAAGCACTTAATACCGGCCATCCAGGCGGTGTTATGTCAACGCACGCTAACTCTGCGCGCGATGCCTTTAA
CCGTATTGGGCTGCTTATCAAGGCGACCCCTATCGGCCGTATGCTCGATATGAGCGATATTATGCGAATGCTCTACTCCACCATT
GACGTTGTGGTGCATATGGAAAAGCGGAAAATCAAAGAAATTTATTTTGACCCTGAATATAAAATGCAGTGTGTGAACGGGA
GCCTGTAATGAAAAACTTAGCAACCTGGCTTCTGGCCGCAGCATTTACGACAGCCGCCCTGCCCGCCTTTGCGGTGGAACCA
TCCGTTCAGGTTGGCTACTCGCCTGAAGGGGGGGTAAGCGGAACCCCAGAAAATTCCGCCATTCCGCATTGTGGAATTTTT
TGGGGGGGTGGTCCGCGGCATGACGACCCGCCAAAGGCTACCGCATCTATCTGAGGCGCTGTGGCGCGACGCTGAAACCCC
GGGGAATTCCGCTACTCCAGTTCCAGGTCCGCAATAGTTCGTCCAGGTCGGGTGGCGTACGCCGGAAACGCCGATTTTTCCG
CAACCGTTTCTGCGTTGTCGGCAAGGTGGTCTCAGAGAGCGCTCAAGAAGCGGTTTTTCTCGTATGTTTAGCTACGGTGGCC
TAGGAGCCTGCGCGGCAGAAGCCAAATCGGCGGAAGATCGAGGACGATACGGGGATGGCTGCCAGTTACCTTCCTTACCGA
CCCGACCAATCCTATCTGCTGCCCCCTTCTCTGGGAGAGTGGCTACCTGAAGGGCATCTTGCCTACTTCATCAGCGAGACTGTC
GATACGCTGGACTTGAGCGCATTCCACGCCCGGTATGCCGGCGGTGGTCCGGGCAATCAGCCGTTTCATCCGGCGATGATGG
TCAAAGTGCTGATCTATGGTTACGCGAGCGGCGTCTTCTCTTCGCGCAAACTAGCCAGGAAGCTGTACGAGGATGTCGCGTT
GCGTGTGCTGGCCGCTGGAAACTTCCCGGCCCACCGCACGCTGAGTGACTTCCGTGCCCTACACCTGACCGAGCTTGAGAAT
TTGTTCGTTCAGGTGGTGCAACTGGCGCGCGAATGTGGGCTGGTGAAGCTTGGCACGATCGCGGTCGACGGCACCAAGGTA
AAAGCCAACGCCAGCCGCCACAAGGCGATGAGCTATAAGCGCATGAAGCCGGCCGAGGACGAATTGCATTGCGAGATCAAG
GCGCTGCTTGATCGCGCCAAGGCTACCGACGACCAGGAGCGTAACGAGCCGGAGCTGGACATTCCTGCCGAGATTTCTCGC
CGCGAGAAGCGCCTGGAGGCGATCCAGGCGGCAAAGGCGCGCCTGGAAGGGGTCGGTTCCGGCTGAGGGCGAAATGACA
CCCTAAGCTTTCGGTTCCTTGGGCCAAAGATATTCGCCAGTCAGTAGAATGTGCGCCCAGCCCAATGGGGATATGTGGGGAA
GAAATTCAGGGGGAACATCCAACCCTTCGTTCCGCCGCTCCGTGACGGCATGACCAAGATGGACGGTATTCCAGTAAATGAT
CACCGCAGTCAATAAATTGAGCCCAGCGATTCGGTAGTGCTGCCCCTCTGTCGTGCGATCGCGAATTTCCCCCTGCCTCCCGAT
ACGGAGCGCATTTTTGAGCGCATGGTGGGCCTCTCCCTTGTTAAGACCGATCTGAGCACGCCGCTGCATGTCCGTATCCAGGA
TCCACTCAATAATGAAAAGGGTCCGTTCAATACGACCAACTTCACGAAGCGCAACTGCAAGGTTGTTTTGTCGTGGGTAAGA
AGCGAGCTTGCGCAGGAGTTGGCTGGGCCTGATTTTGCCAGCGGTCATCGTCGCGGCACAACGGAAAATATCAGGCCAGTT
CGCAACGATAAGATCCTCCCGGGCTTTTCCACCTACCAACTTGCGTAACTCCCTGGGGGTCGTATCGGGATTAAATACGTACAA
CCGCTTCGATGGCAGATCCCTGATTCGCAGAACGAGATTGTAGCCGAGCAGGCTACTGGCTCCGAACAAATGGTCGGTGAAT
CCTGCTGTATCGGCATACTGTTCGCGAACATGGCGACCGACCTCGTTCATCAGTAGTCCATCGAGAATATACGGTGCCTCGCTC
ACGGTCGCCGGGATCGACTGACAAGCGAATGGCGCGAACTGGTCGCTTACGTGAGTATACGCTTTGAGGCCGGGAACAGAA
CCATATTTGGCATTGACCATGTTCATGGCTTCGCCATGCCGCGCTGTCGGGAAAAACTGACCATCGCTCGATGCTGACGTGCC
CATCCCCCAGACGCGTGACATCGGCAGTTTACCCTGCGCGGCCACCACAATTGCCAATGCCTGGTTCATGGCTTCGCTTTCAA
CATGCCAGCGGGCAAGGCGTGAGAGCTGCCAGTAATCATGCGTGTTTGTAGCTTCCGCCATCTTACGCAGGCCCAGATTGAG
CCCTTCAGCGAGCAGGACGTTGAGCAGACCGATCCGGTCGCGACATGGAGCCCCGGTTCTCAGATGGGTAAACGCATCTGT
GAAACCAAGGGCTGCATCAACTTCAAGCAGCATGTCGGTAATCCGAACGGACGGCATTCGGCGATACAGATCCAGTATGAGT
GCCTCGGCACCATCCGGCACGTCTGCTGTCAACCTGTCGATCCGCAACGTTCCATCTTCTATGCTACCGTGCGGAATAGTGCCG
TTACGGGCAGCCCGGGCCAGCCGCTTAAGAGCGATCGTGAGTCGCGCCTTTCTGTCTGCCAGCCAATCCTGTGGGTTGGAAG
GCACGGCCAGTTTTGCATTTTCCTGCGCCGCGATCATCGGCACCAGTACCTGCTTGAGGTCACCATAGCGGCGCGAATGAGC
GAGCCAGACATCTCCGGAACGAAAAGCATCCCGGAGGTGAAAGAGTACCGCCACTTCCCAAAGACGGGTATCTCCTTTTTCC
TGAGCTCGTAAATGACGGTTCCATTTGGAGCTGGGCCGCAGGAAACGCCTTTCTGGCGATGCAACACCTTTCATCTCTCCGAT
CGACAAAGCTGCTGCTACCAATGGTCCGGCGACCGGCGCGGCTTCGAGCTTCAGACAGCGCAACATGCGGGGCGCATAACG
ACGAAAGCGATGGTATCCCTGCCCGACATATGCAAGAGGCTCATCGGCTAGCGTGTTGCTGAGTTGAGTCCCTGTCGCTACCA
GTTGAGCGAGCCGGTCCCATGCAACCGAACTGGCGACAGCCATCTCCAGCGGGGTTCCGTCACTGCGGGCCTCAAGCAACG
AAGCTCCCAGCGCGGTGAAGGTACGGATCGTATCCGTGAGTGTGGCTTTAGAGCCGGAAATTGTTTCGTCATGCTGGCGCTT
CGCTTCCCGCCAGGTTTTTCCTACGATCCTGTCATGGGTTTCGACTATGGCATCAGCAATCGCCGCTTCCCACTCCACAACACA
GACGGCAAGGATCGCCCAGCGGCGGTCCGAAGTGATGTCACGCAAACCGTCGGTGAAGTAGCGTTCACCCTGCCGACGCA
GCCGGGCAATGCGATGGGCAGGTATGCTGGCCAAAGCACTATGATTGATATTCAGGGTACGCAGAAATTCGAGCCTGTCGAG
CAAACGGTTAGCAGCAGCCGAGTTGTTACCAACCTCGAAGTTGCGAAGCCAGATGAAACGACTGATATTGCCGGCGAGCATT
TCACTCAGAAGTTTGTCCAGGTGATCGCGAACATCCGCTGTTAAATTTTCCACAATCCGCGTTTCAATCCGCCGCTCAGCGGC

GACCAGAGCATCCGCGCACAAGCGCTCGATTGTCGATACTGCGGGCAGAATGGTGGAAGTTTCCCGACACCGCACAATAAA
ACGATGAGCAAGATCCTCGTTTGATCTGGCATCTTCGGCCTGGCCGAAAGTCCACTCCCGCAGATCACGGGCACCACGGCCC
GTGAAGGTCTTGTAGCCGTAAATTTCGCGCAGCGTGTCCATGTGCTGCTGACGGGTTTGGCGCCGTGTGGCATAAGTGAGAA
GCGCATCAGCCGGAACTCCAAGCTGAGCACCGACGAAGGAAAGGACTTCACGCGGGATCATCTCACCAGGAGCCAGTGCA
CGGCCCGGATATCGTAAGGCACAAAGTTGCAGGGCAAAGCCAATCCTGTTTTCCGGTCTGCGGCGCTGCCTAATGTTTTCCA
GGTCATCATCGCCCAGCGTGTAGAACTTCAGTAGCGACAGTTCGTCCGTGGGCAGATCGAACAGCGCTGCTCGCTGCCGTTC
GGTGAAAATATGGCGTCGTGACATACAAATTCGTCCCTTTTGAAGTATAGTCTGTTTTGGACAACAGCCAGCCCATATAAATCA
GGGCGTTCCGATACAAAAATCCAGGAGGGTTCAATTGGGACATCGTGCCGCCATTTACTGCCGGGTTTCAACAGCGGATCAG
TCTTGTGAACGCCAGGAATTTGATCTGCGAGCCTTCGCCGGCCGTGCCGGCTACGACGTGGTGGGAATATTTAAGGAAACAG
GTTCAGGAACTAAACTCGACCGGGCCGAGCGAAAGAAAGTCCTGGCGCTTGCCCAGTCCAGACAAATTGATGCAATCCTGG
TCACTGAGCTTTCCCGGTGGGGGCGCTCGACGCTCGATCTGCTCAATACGCTACGTGAACTGGAGAACTGGAAGGTTTCCGT
GATAGCCATGAATGGAATGGCGTTCGATCTTTCGTCGCCGTATGGACGAATGCTGGCGACGTTTCTTTCCGGCATTGCGGAGT
TTGAGCGGGATCTCATCAGCGAGCGGGTCAAGTCAGGCCTTGCTGTTGCGAAGGCACGTGGTAAGAGGCTTGGTCGTCAGG
CCGGAGTGCGACCAAAATCAGACCGACTTTTGCCTAAGGTGGTTGCGATGAGGGCCGAGGGACGCAGCTATCGCTGGATCG
CACGCGAGCTCGGTATCAGCAAGAATACCGTCGCTGACATCGTGCAACGACACAGAGCTAACGCTTAGGGTGTCATTTCGCC
CTCAGCCGGAACCGACCCCTGGAAGCGCGCCAGCGTGAAGCGGACCAGGCCCGGGGGCGCAGCGAAGACGATGGCCGCC
GGCCTCGCCATCCGGATGGCTCGGACAAGGGCGGTGGCTCGTACAAACGCGAGTTTGGTGTGCCGGATGACCGTGATCAGG
AAAGCTTCACCGATCCGGACAGCCGGATCATGAAACACGCCGGTGGTGGCTCCGAGCAGAGCTACAACGGGTACACAGCGG
TCGATGCCGAGCACCAGATCATCGTGGCGGCGGAGTTGACCAACTGCGCCGCGGACAGTCAGGCGCTGCTGGGCATGCTGG
CAGCAGTTCAGGCCAACACCGGAGAAATGCCGGCCCAGACGCTGGCGGATGCGGGATTCCGTAGTGAGGCTGTTCTGGCA
AAGGTCGCCGATCACCACGGCGATGTCATCGTTGCCCTCGGCCGCGAGGGACGTGAAGATGCCAAGGTCAATGCCAAGACC
CATCCGCATACGGCGGCGATTGCGGCGAAATTGAAAACGGAGCAAGGCGATGCAGCTTACCGCCGGCGCAAGTCGATCGTG
GAGGCTCCGAATGGTTGGATCAAGGCGGTGATGGGATTGCGTCAGTTCAGCATGAGGGGCCTGGACAAGGTGCAAGCCGA
GTGGAAGCTCGTCTGCATGGCGCTAAATCTGAGGCGAATGGCGTATCTGTGAGGGCGAAGGTTAAATGGGGCGGCTCAAAT
GCACCCCAGTCGTCATAACACGCCGCGCGCCGCAAGATTGGTATCCTTGGCGCCGACGCCTTGCCAATTGCAGAGAGCGCCG
CCGCCATCGTCAGTGCTCTACAGAAAACCGGTCACACGGCTCTGCCGCGCAGACTCCTAGCCTAAATGTGACAGTGGTTGGT
AATCCATGCCGCCCGCAAGGGCGGCGGTGGTGGGCCAATAGATGATTTTCAGAGCCTTACTGCCCGTTGACGCCCAATCCCT
CGAGCGCGAGTCTAGCCGCAGCGGCGATGACGGCCTCGCTGTGCTTGTCATCCTTGTTAGGCGCCCGGGTGTAGACGGCCA
ACACAATAGGTGCGCGCCCAGTGGGCCAGACGACGGCATAGTCATTTGCCGTGCCATACACTCCGCAGGTTCCGGTTTTGTC
TCCGACTGCCCAGTCTGCCGGCACCGCCGCGCGGATGCGGTGGTTGCCGGTCGTGTTTCCCTTTAGCCAATCAACAAACTGC
TGCCGCTGCGGCGCAGCCAGTGCAGAGCCCAGTGTCAGTTTTTGTAAGCTTTCCGTCACGGCGCGCGGCGATGAGGTATCG
CGCGCATCGCCTGGGATGGCGGAGTTCAGCTCCAGCTCCCAGCGGTCCAGACGGAACGTGGTATCGCCGATAGAGCGCATG
AAGGCCGTCAGCCCGGCCGGGCCGCCCAACTCCTTCAGCAACAAATTGGCGGCGGCGTTATCACTGTATTGCACGGCGGCC
GCGGACAGCTCCGCCACCGTCATGCCTGTTGTCAGATATTTTTCCGAGATGGGTGACCACGGAACCAGCGCATTTTTGCCGTA
ACGGATGGGTGTGTCCAGCAAGCCGGCCTGCTGCTGGCTGCGAGCCAGCACAGCGGCAGCAAGAAAGCCCTTGAATGAGC
TGCACAGTGGGAAGCGCTCCTCAGCGCGGTAACTTACAGTTGCGCCTGAGCCGGTATCCATCGCGTACACACCGATGGAGCC
GCCAAAGTCCTGTTCGAGTTTAGCGAATGGTTCCGCGACGAGGTTGGTCAGCGCGGTGGCAGAAAAGCCAGCCAGCGGCC
ATGAGAGACAAGACAGCAGAACTAGACGGCGATACAGTGACATCAACGATATTCCTTGTTTGAAGGTGGAGTTACGGACGG
CCTCAGGAAGTCCTGGCCAAGCCCCGACTATTGGGGCGCGAAGATAGCACCGAGGGTACGCCGGTGTCAACACGGGGTCTG
ACGCTCAGTGGAACGAAAACTCACGTTAAGGGATTTTGGTCATGAGATTATCAAAAAGGATCTTCACCTAGATCCTTTTAAATT
AAAAATGAAGTTTTAAATCAATCTAAAGTATATATGAGTAAACTTGGTCTGACAGTTACCAATGCTTAATCAGTGAGGCACCTAT
CTCAGCGATCTGTCTATTTCGTTCATCCATAGTTGCCTGACTCCCCGTCGTGTAGATAACTACGATACGGGAGGGCTTACCATCT
GGCCCCAGTGCTGCAATGATACCGCGAGACCCACGCTCACCGGCTCCAGATTTATCAGCAATAAACCAGCCAGCCGGAAGGG
CCGAGCGCAGAAGTGGTCCTGCAACTTTATCCGCCTCCATCCAGTCTATTAATTGTTGCCGGGAAGCTAGAGTAAGTAGTTCG
CCAGTTAATAGTTTGCGCAACGTTGTTGCCATTGCTGCAGGCATCGTGGTGTCACGCTCGTCGTTTGGTATGGCTTCATTCAGC
TCCGGTTCCCAACGATCAAGGCGAGTTACATGATCCCCCATGTTGTGCAAAAAAGCGGTTAGCTCCTTCGGTCCTCCGATCGT

TGTCAGAAGTAAGTTGGCAGCAGTGTTATCACTCATGGTTATGGCAGCACTGCATAATTCTCTTACTGTCATGCCATCCGTAAG
ATGCTTTTCTGTGACTGGTGAGTACTCAACCAAGTCATTCTGAGAATAGTGTATGCGGCGACCGAGTTGCTCTTGCCCGGCGT
CAACACGGGATAATACCGCACCACATAGCAGAACTTTAAAAGTGCTCATCATTGGAAAACGTTCTTCGGGGCGAAAACTCTCA
AGGATCTTACCGCTGTTGAGATCCAGTTCGATGTAACCCACTCGTGCACCCAACTGATCTTCAGCATCTTTTACTTTCACCAGC
GTTTCTGGGTGAGCAAAAACAGGAAGGCAAAATGCCGCAAAAAAGGGAATAAGGGCGACACGAAAATGTTGAATACTCATA
CTCTTCCTTTTTCAATATTATTGAAGCATTTACCAGGGTTATTGTCTCATGAGCGGATACATATTTGAATGTATTTAGAAAAATAA
ACAAATAGGGGTTCCGCGCACATTTCCCCGAAAAGTGCCACCTGACGTCTAAGAAACCATTATTATCATGACATTAACCTATAA
AAATAGGCGTATCACGAGGCCCTTTCGTCTTCAAGAATTTTATAAACCGTGGAGCGGGCAATACTGAGCTGATGAGCAATTTC
CGTTGCACCAGTGCCCTTCTGATGAAGCGTCAGCACGACGTTCCTGTCCACGGTACGCCTGCGGCCAAATTTGATTCCTTTCA
GCTTTGCTTCCTGTCGGCCCTCATTCGTGCGTTCTAGGATCCTCCGGCGTTCAGCCTGTGCCACAGCCGACAGGATGGTGACC
ACCATTTGCCCCATATCACCGTCGGTACGGCACTGTTGCAAAGTTAGCGATGAGGCAGCCTTTTGTCTTATTCAAAGGCCTTAC
ATTTCAAAAACTCTGCTTACCAGGCGCATTTCGCCCAGGGGATCACCATAATAAAATGCTGAGGCCTGGCCTTTGCGTAGTGC
ACGCATCACCTCAATACCTTTGATGGTGGCGTAAGCCGTCTTCATGGATTTAAATCCCAGCGTGGCGCCGATTATCCGTTTCAG
TTTGCCATGATCGCATTCAATCACGTTGTTCCGGTACTTAATCTGTCGGTGTTCAACGTCAGACGGGCACCGGCCTTCGCGTTT
GAGCAGAGCAAGCGCGCGACCATAGGCGGGCGCTTTATCCGTGTTGATGAATCGCGGGATCTGCCACTTCTTCACGTTGTTG
AGGATTTTACCCAGAAACCGGTATGCAGCTTTGCTGTTACGACGGGAGGAGAGATAAAAATCGACAGTGCGGCCCCGGCTG
TCGACGGCCCGGTACAGATACGCCCAGCGGCCATTGACCTTCACGTAGGTTTCATCCATGTGCCACGGGCAAAGATCGGAAG
GGTTACGCCAGTACCAGCGCAGCCGTTTTTCCATTTCAGGCGCATAACGCTGAACCCAGCGGTAAATCGTGGAGTGATCGAC
ATTCACTCCGCGTTCAGCCAGCATCTCCTGCAGCTCACGGTAACTGATGCCGTATTTGCAGTACCAGCGTACGGCCCACAGAA
TGATGTCACGCTGAAAATGCCGGCCTTTGAATGGGTTCATGTGCAGCTCCATCAGCAAAAGGGGATGATAAGTTTATCACCAC
CGACTATTTGCAACAGTGCCTTATTTCGAGCATTTTCTTGCGAAAAAGCCGACCGGGAAAATTGACATGCGTACCATTCGCCT
GGGGTGTAATAAGGCGATAGACACCGGTACCTACACATTTACCTTCGCGGACAAATCAACGGTTTCAGCCCGCTACACATTCA
CTTATGCATGGGACGGTAAAGAGTGGAAAATTTCCACACACCACTCTTCAGCGATGCCTGAAGGGTAAAAAGCTGTGCAGGG
GGCGGAATATTCACCGCTCCCTGAAGACTAGCCAATAATCGCGATGCCAAGACGTTCCATGAGCAACGATGCCTGGTAGTTGT
CCAACTTAACGCCTTGTAAATCAACGCGCCGAATATCTAAGTCACCCAACTCCGAATTGGTCAGATCGCAATGTGTGAAGTTTG
CTGCTCGCCAGTCGAAAGTCGAAAACTCGCCGCCGGAGAGATCTGAACCACTGAACGTCGCGCCCAGTACCTGGGCCCCCAT
CCAACGGTTTTCCCACAGCTCACACTTTTCCAACACGACTTTCGAAAAATTGGCGTAGCTTAGATTTGTGTTAGTGATATATGC
ACTGCAAAACCAGGTGCGAGTAGTGATCATATTCATAAAACTTGCGCCGCGAAAATCTGCGCCTTGCGCACGACAGTGGCGA
ATTTCAATGCCAAGCGCACTGGCATTGCGAAAATCCGCCATGGATAAATCACAGCTTTTAAAAATGGCATCTTTCAGCATCGCA
CGACTAAAATTGCACCCTTTCTGGCTTTCACGATCATAGAACTGACAGCCGATAAATTCAGTGCCGCTCAGGTCGGCACCTGA
AAAATCACAGTTAAAAAATGTACTATTTTCAATTTTCTCACCGGTGAAGCGGTTTCTGTTAATTTTTTCGCCAACGAGTGCCAG
AGCCATATTTTGTGCCTGTTTTTTTATACAGTAATGGCGTCATGGTAAACCCTGATGAGGTTATGCGTCAAATCCGCCAATATAA
CATCTGCAAATGTGCGTTAAATCTGGTGTTTTTTCAGCAAAGCGCGAAGCTGATGGTAAGTCAGACCCAGTAATTCAGCGGCT
TTTTTCTGGTTAAATTTTGCCTGCTGTAAGCTGGTTTGTAGAAAGTCTTTCTCTTGCTGCTGCTGGAATTCACGCAGATCCAGC
GGTAACCCTACAGACATCGGTTTAGTTTCCGGCGCCTGCGGCTGCGTCTGGTTCTGAAATCCATCCCTGTCGGTGTTGCTTATG
CAGTCTGGTCGGGACTCGGCGTCGTCATAATTACAGCCATTGCCTGGTTGCTTCATGGGCAAAAGCTTGATGCGTGGGGCTTT
GTAGGTATGGGGCTCATAATTGCTGCCTTTTTGCTCGCCCGATCCCCATCGTGGAAGTCGCTGCGGAGGCCGACGCCATGGTG
ACGGTGTTCGGCATTCTGAATCTCACCGAGGACTCCTTCTTCGATGAGAGCCGGCGGCTAGACCCCGCCGGCGCTGTCACCG
CGGCGATCGAAATGCTGCGAGTCGGATCAGACGTCGTGGATGTCGGACCGGCCGCCAGCCATCCGGACGCGAGGCCTGTAT
CGCCGGCCGATGAGATCAGACGTATTGCGCCGCTCTTAGACGCCCTGTCCGATCAGATGCACCGTGTTTCAATCGACAGCTTC
CAACCGGAAACCCAGCGCTATGCGCTCAAGCGCGGCGTGGGCTACCTGAACGATATCCAAGGATTTCCTGACCCTGCGCTCT
ATCCCGATATTGCTGAGGCGGACTGCAGGCTGGTGGTTATGCACTCAGCGCAGCGGGATGGCATCGCCACCCGCACCGGTCA
CCTTCGACCCGAAGACGCGCTCGACGAGATTGTGCGGTTCTTCGAGGCGCGGGTTTCCGCCTTGCGACGGAGCGGGGTCGC
TGCCGACCGGCTCATCCTCGATCCGGGGATGGGATTTTTCTTGAGCCCCGCACCGGAAACATCGCTGCACGTGCTGTCGAAC
CTTCAAAAGCTGAAGTCGGCGTTGGGGCTTCCGCTATTGGTCTCGGTGTCGCGGAAATCCTTCTTGGGCGCCACCGTTGGCC
TTCCTGTAAAGGATCTGGGTCCAGCGAGCCTTGCGGCGGAACTTCACGCGATCGGCAATGGCGCTGACTACGTCCGCACCCA

CGCGCCTGGAGATCTGCGAAGCGCAATCACCTTCTCGGAAACCCTCGCGAAATTTCGCAGTCGCGACGCCAGAGACCGAGG
GTTAGATCATGCCTAGCATTCACCTTCCGGCCGCCCGCTAAATATCTCCTTTTGGGTTGTTAATAAAACATCCAATAAGTTGACT
GTGCGTGAAAAAGAAAGTTTTGTGTGATGGCGTTGAAGATCGCACCGTTAAGCTCTTATGTGGGATGGTGCAGAGCTCGACG
ACTACCGATAAAACGCAACCGCCGCAAACAGACAAGAAAAAGCCCCAACTGATAACAGTTGGGGCTTCAGTATTGTGATTGG
TGGAGCAATAGCACCCTGAACCCAAAACCTTCTCGCTCAACCGGTAGTGGCTGATAACAACTCGTGAGGGCTATTGCGGGTT
AAGCATTTAGCGATGTCTAGGGCCAGACTGGACGTCTGAACGCAAGCCGCTGATACTGTACATAACCACAGTATCAGCGGAG
GATACCCATGTCGCTGGCAAGGAACGCCACGGCGAGTCAATCGCCCACTCAAACAAACGGTTACGAACGCCACCAACCCGA
CCAGACGCTGCTCTACCAGCTGGTTGAGCAGCACTACCCAGCCTTCAAAGCCTCACTCGAAGCCCAAGGTCAACACCTGCCT
CGCTACATCCAACAAGAATTCAACGACCTCCTCCAATGTGGCCGTCTGGAGTATGGTTTCATGCGGGTTCGCTGCGAGGATTG
TCATCACGAGCGTCTGGTCGCCTTCAGCTGTAAACGACGCGGCTTTTGCCCTAGCTGCGGTGCCCGCCGGATGGCCGAGAGT
GCGGCGCTGCTGATAGACGAAGTCTTCCCCAAGGAGCCCATTCGCCAGTGGGTGCTCAGCTTTCCTTTCCAGCTACGCTTTTT
GCTGGCTCGCCATCCCCAGCTGATGGGCCAGGTCTTGAGTATCGTCTATCGTACACTCTCAACTCATCTGATCAAAAAAGCCGG
TTACACCAAAGCCTCTGCACAAACTGGCTCAGTGACTCTTATCCAACGCTTTGGCTCCGCGCTAAATCTCAATGTCCACTACCA
CATGCTGTTTCTCGATGGTGTCTATGCCGAAGATGACTATGGCAAGCAACGCTTCCATCGTGTCAAGGCACCCACTTACGATGA
GCTGAATACGCTCGCTCACACCCTCAGCCATCGCATCGCTCGCTGCATGGAAAAGCGTGGGATTTTGGAGCGTGATGCCGAG
AATACGTGGTTGACACTGGAAGAGGGCGAAGACGATACGCTGACTCAATTACATGGTGCTTCGGTTACGTATCGCATTGCCGT
CGGCCCCCAGCAAGGGCGCAAAGTCTTCACCCTGCAAACCTTGCCAGGGCGTGAGGATAAAGCCGACTCAAGCAGTCGAGT
AGCCAACCATGCTGGTTTCTCGCTACACGCCGGTGTGATGGCCGAAGCGCATCAGCGGGATAAGCTTGAGCGCTTGTGTCGC
TACATTAGTCGGCCAGCGGTTTCAGAAAAACGTCTGGCATTAACCGCCAATGGGCAGGTGCGTTACGAGCTCAAAACTCCGT
ACCGCAATGGCACCACCCATGTGATCTTCGAGCCGCTGGACTTCATCGCCAAACTCGCTGCGTTGGTACCTAAGCCGCGAGTC
AACCTCACACGCTTCCACGGCGTCTTTGCACCGAACAGCAAACACCGAGTTCAAGTAACACCCGCCAAGCGGGGCAAGAAG
CCCGACAAATCGGAAGGTCTCGATACTAACTGGCGTGACAAGAGTCCTGCAGAGCGCCACCGCGCCATGACCTGGATGCAAC
GCCTCAAGCGAGTCTTCAATATTGATATTGAAGTCTGCGAACACTGCGGCGGTCACGTCAAAGTGATTGCCAGCATCGAAGAT
CCGAAGGTCATTGAGCAGATTCTCAAGCATCTGAAACAGAAAACAGCCAAGGCGAATGCCGCCAAGCAGCGTGAGCTGCCA
CCAGAACGAGCGCCGCCACTGACTCCCAGCCTGTTCGATCCATCACAGAGTCGTCTCTTTGACTGACGACCCCAAATCCAACA
CTGCTCAACACTGCCAACTTTTAAACGGGGCGGTGGGGCAGTTTGTATCTCTCGAGCTATCAGGCTAGAGATTTTACCGCCAA
ATCGAACCTTATTAGAGCGGTTTAGGCTGGACCGGCAGTTAAAATTGGGGCTTGAGCGGTAAACGAGTGAGGGAATTTCAG
GTAAGATACTTCGGATGAGGAGCAAAAAGGTGGTTTATACTTCCTATACCCAGTAGTGTTCCCCTGTCGGTTTGGTTGGGGTC
GATGCCCCACTTTGGGCGCGGCGTTGGGCGCCGCAAGATGAAAGCTCTGTGTGAAGGCTTGGATATTAAGACCTTGGATGAT
TTCAATGCCATCACCGAAGAGCAGATCGTTAGCGTCGACAAATTCAGCTACAAAACTGCCGCAAAAGTGCTTGCAGGTATGG
CTGAACATGCCGAGCTATTCAACAAGATTCAATCAATCATCGGGTTCAAACAGCAGGTCACTTCGGGGGATCGTTTTACCGGT
GAGAAGATCGTGATCACCGGTTTTCGTGACGCGGCCTTGGAGAAGCTGATTGAAGCTGAGGGTGGTGAAGTGCAATCATCA
GTCTCGTCGAAGACCACCATGGTGATCGCGGCATCCACTTCCGGATCTTCCGGCAAATTGAAGAAAGTGCACGATCTCAACA
ACAGCGGAAAGGCAAATATCAAACTGATTGATCTGGCCACCTTCCGTAAGCAATATCTGGAACAACCAGCATCGACTGGTTTG
GAGTTTTAATGAGTCACCCACAACTTGAGCTAATAGTCGCTGTGGATTCTAAGTTGGGATTCGGGAAAGGCGGCAAGATTCC
ATGGAAATGCAAAGAAGACATGGCGCGATTTACGCGGATTTCTAAAGAGATCCGCGTGTGCGTTATGGGGAAACACACGTAT
ACTGACATGCGTGACATGCAGTTAGAAAAGGATGGCGCCGAGGAGCGAATCAAGGAGAAAGGAATTCTCCCCGAACGCGA
ATCGTTCGTGATCTCCTCGACGTTAAAACAAGAAGATGTCATAGGCGCTACTGTCGTTCCTGATCTTCGTGCTGTGATCAACCT
GTATGAGAATACCGATCAACGCATTGCTGTCATTGGTGGGGAGAAGTTGTACATTCAAGCTCTTTCATCAGCAACGAAACTGC
ACATGACCATAATTCCAAGAGAGTTCGACTGTGATCGATTTATTCCTGTTGATCCGATCCAGAACAATTTTCACATTGATTCCAG
TGCCAGCGAGACTGTGGAGGCAACCGTTGATGAGACTCAAGAGCGCATTCACTTTGCTACTTACGTGCGTAACAATCAGTAA
CGCGCTGGCAGTGGAAGAACAAGAACCAGAAACGAAGCAGACGGTGTTGGCCGTCTGCTTCGAAACATCATTATTGGCTCG
AGAACTGATCGAGAATACCATTCTTGCGGACGACATCCAAAACAATACAGAAAAGATAGCTTTGCAGTTGTCGGAGCCCCTCC
CCCCAACTTATAATGGGATTATTGCTGGGCTAAACAATGCTGTCGCCATGCGTGGGACAGCAAACGTTGAATCGCCATCAAGA
GCATCCACAGATTTACGTGCAGCCGTCATCGGTTTATGGTGTATAAATGACACCATAGCCGCCCTGAATCAGACTGATAATGCA
GTCTTACAGGACGGCGAGTTGCAACAAACACTTGTCGATTATTACAACAACTTATTGGCTGACATCGTTTGTCGTTTTCAGAA

GACGGCTGCACTGAACGTCAGAAGCCGACTGCACTATAGCAGCGGAGGGGTTGGATCCATCAGGCAACGACGGGCTGCTG
CCGGCCATCAGCGGACGCAGGGAGGACTTTCCGCAACCGGCCGTTCGATGCGGCACCGATGGCCTTCGCGCAGGGGTAGT
GAATCCGCCAGGATTGACTTGCGCTGCCCTACCTCTCACTAGTGAGGGGCGGCAGCGCATCAAGCGGTGAGCGCACTCCGG
CACCGCCAACTTTCAGCACATGCGTGTAAATCATCGTCGTAGAGACGTCGGAATGGCCGAGCAGATCCTGCACGGTTCGAAT
GTCGTAACCGCTGCGGAGCAAGGCCGTCGCGAACGAGTGGCGGAGGGTGTGCGGTGTGGCGGGCTTCGTGATGCCTGCTT
GTTCTACGGCACGTTTGAAGGCGCGCTGAAAGGTCTGGTCATACATGTGATGGCGACGCACGACACCGCTCCGTGGATCGGT
CGAATGCGTGTGCTGCGCAAAAACCCAGAACCACGGCCAGGAATGCCCGGCGCGCGGATACTTCCGCTCAAGGGCGTCGG
GAAGCGCAACGCCGCTGCGGCCCTCGGCCTGGTCCTTCAGCCACCATGCCCGTGCACGCGACAGCTGCTCGCGCAGGCTGG
GTGCCAAGCTCTCGGGTAACATCAAGGCCCGATCCTTGGAGCCCTTGCCCTCCCGCACGATGATCGTGCCGTGATCGAAATCC
AGATCCTTGACCCGCAGTTGCAAACCCTCACTGATCCGCATGCCCGTTCCATACAGAAGCTGGGCGAACAAACGATGCTCGCC
TTCCAGAAAACCGAGGATGCGAACCACTTCATCCGGGGTCAGCACCACCGGCAAGCGCCGCGACGGCCGAGGTCTTCCGAT
CTCCTGAAGCCAGGGCAGATCCGTGCACAGCACCTTGCCGTAGAAGAACAGCAAGGCCGCCAATGCCTGACGATGCGTGGA
GACCGAAACCTTGCGCTCGTTCGCCAGCCAGGACAGAAATGCCTCGACTTCGCTGCTGCCCAAGGTTGCCGGGTGACGCAC
ACCGTGGAAACGGATGAAGGCACGAACCCAGTGGACATAAGCCTGTTCGGTTCGTAAGCTGTAATGCAAGTAGCGTATGCGC
TCACGCAACTGGTCCAGAACCTTGACCGAACGCAGCGGTGGTAACGGCGCAGTGGCGGTTTTCATGGCTTGTTATGACTGTT
TTTTTGTACAGTCTATGCCTCGGGCATCAAGGCGAGCTCAGAGACCATGGAAAGCATGTTCTCGGACTTACGTAGCAACTCGT
TTCTTTTCGCAGGTTGAGCCACCTCCGCGCTTCATCAGAAAACTGAAGGAACCTCCATTGAATCGAACTAATATTTTTTTTGGT
GAATCGCATTCTGACTGGTTGCCTGTCAGAGGCGGAGAATCTGGTGATTTTGTTTTTCGACGTGGTGACGGGCATGCCTTCG
CGAAAATCGCACCTGCTTCCCGCCGCGGTGAGCTCGCTGGAGAGCGTGACCGCCTCATTTGGCTCAAAGGTCGAGGTGTGG
CTTGCCCCGAGGTGATCAACTGGCAGGAGGAACAGGAGGGTGCATGCTTGGTGATAACGGCAATTCCGGGAGTACCGGCG
GCTGATCTGTCTGGAGCGGATTTGCTCAAAGCGTGGCCGTCAATGGGGCAGCAACTTGGCGCTGTTCACAGCCTATCGGTTG
ATCAATGTCCGTTTGAGCGCAGGCTGTCGCGAATGTTCGGACGCGCCGTTGATGTGGTGTCCCGCAATGCCGTCAATCCCGAC
TTCTTACCGGACGAGGACAAGAGTACGCCGCAGCTCGATCTTTTGGCTCGTGTCGAACGAGAGCTACCGGTGCGGCTCGACC
AAGAGCGCACCGATATGGTTGTTTGCCATGGTGATCCCTGCATGCCGAACTTCATGGTGGACCCTAAAACTCTTCAATGCACG
GGTCTGATCGACCTTGGGCGGCTCGGAACAGCAGATCGCTATGCCGATTTGGCACTCATGATTGCTAACGCCGAAGAGAACT
GGGCAGCGCCAGATGAAGCAGAGCGCGCCTTCGCTGTCCTATTCAATGTATTGGGGATCGAAGCCCCCGACCGCGAACGCCT
TGCCTTCTATCTGCGATTGGACCCTCTGACTTGGGGTTGATGTTCATGCCGCCTGTTTTTCCTGCTCATTGGCACGTTTCGCAA
CCTGTTCTCATTGCGGACACCTTTTCCAGCCTCGTTTGGAAAGTTTCATTGCCAGACGGGACTCCTGCAATCGTCAAGGGATT
GAAACCTATAGAAGACATTGCTGATGAACTGCGCGGGGCCGACTATCTGGTATGGCGCAATGGGAGGGGAGCAGTCCGGTT
GCTCGGTCGTGAGAACAATCTGATGTTGCTCGAATATGCCGGGGAGCGAATGCTCTCTCACATCGTTGCCGAGCACGGCGAC
TACCAGGCGACCGAAATTGCAGCGGAACTAATGGCGAAGCTGTATGCCGCATCTGAGGAACCCCTGCCTTCTGCCCTTCTCCC
GATCCGGGATCGCTTTGCAGCTTTGTTTCAGCGGGCGCGCGATGATCAAAACGCAGGTTGTCAAACTGACTACGTCCACGCG
GCGATTATAGCCGATCAAATGATGAGCAATGCCTCGGAACTGCGTGGGCTACATGGCGATCTGCATCATGAAAACATCATGTTC
TCCAGTCGCGGCTGGCTGGTGATAGATCCCGTCGGTCTGGTCGGTGAAGTGGGCTTTGGCGCCGCCAATATGTTCTACGATCC
GGCTGACAGAGACGACCTTTGTCTCGATCCTAGACGCATTGCACAGATGGCGGACGCATTCTCTCGTGCGCTGGACGTCGAT
CCGCGTCGCCTGCTCGACCAGGCGTACGCTTATGGGTGCCTTTCCGCAGCTTGGAACGCGGATGGAGAAGAGGAGCAACGC
GATCTAGCTATCGCGGCCGCGATCAAGCAGGTGCGACAGACGTCATACTAGATATCAAGCGACTTCTCCTATCCCCTGGGAAC
ACATCAATCTTACCGGAGAATATCGTTGGCCAAAGCCTTAGCGTAGGATTTCGCCCTCTCCCGCAAACGACCCCTAAAAGCCG
TTTCCTCTGTATAGGGGTTTGAAGGCCAATGGAACGAAAACGTACGTTAAGAATGTAATTTATTGATATTCATAAAAATAAAAA
AAGGATCGCTGATTGATCCTTTTGTTTTGCTATTTGAATTTCGATAAACATTAACCACGAGAATGCAGTAGCTCGGCTATCTCAT
GAGCAATTTCATCGATTGTATGAGTTGCAGTACTACGAGCAACTTTGTCTGCTAATGATGGACTAAAATCGACAACTTCTTGTT
TTGTAATATTATGCCATATGGGAAGCAATATTTGCTCACCAGAAATAGCACGAGTGACGATTCCATCAAGTTCATAATTTGTCCA
ACCTTTGCTTATAAACGCCGGAGACAAAACTACTAACCCAACACGACTGTTTGCTAAACCTTTGTCTATTTTTTGTCTCAGGCT
GTCACCAATTCTTAACGTCATTTCGTCATACCATACATTAAGGCCGTGACTGATTAATGAGTTTGCCAGAGAGCGAACAAAATC
ATCTTTATCCTCTGAGGCATGCGAAATAAATACGTCATGTATCTCGTTGCTCTGTGCTGTTGACGGTGGTCTGTTATCTCTTACCA
GACTAGGAACTTCGCTTAACGGCCTATCCTGAATTTCAGGAAGAACCCCTGGAAGAACGCGAACAGAAGCCCTGGTACTCCC

CCTTAATCCCTGCATATCTACAGCAATATGCCAATGGCCTGATGAAGGGATCTGTAACCTTATGGGGGAGCGCTTTGCTAACCC
ACCAATGTAGGAATGTCGTCGGCCATTCTTATAACTACTGAAGTTAGAACTATCCATTAGTCTAACATTTGCACCACTGGTAAGG
GTGATTTCAACAATCTCCCCTCTTTTTCTTTGACCAAGATCATTATGGATGAATTGCATGTAAGTCTGCTCTCTTCATTTGAATTG
GAAGAATGTAGCCACTTCCTGATTATACTGTATCCAGTGGTTGAGAGTATCTCTTAGTGTGAAAAAATAAATTATAAGACTTTTA
CTCGGTATGGATGAAGATGTAAAATCCGTAAACCAGAGAATAAATGAGGAAAAAGCGTGACCAAGCACAAAGTTTTCATCAG
TTACCACCATGCTAATGACCAATGGTACAAAAACGAACTTGAAAAAATGAATGATGTTTTTGATATTTTTGTGAATCGCTCGGT
ATCACTTGGTGACATTGATGAAGAAGAAGAACCTCAAAAGATCAGAGAAATAATAAGGGATGAATATTTACGTGATACATCCG
CTCTTATTTTACTCGTTGGGACTGAGACTAAAAATAGAAAGCATGTTGATTGGGAATTATATTCATCCATGAGGGATAGTCCCA
GAAACGGCAAATCAGGTATTTTTATTATAAATCTTCCTTCAACAGGGACAAACAACATTAGAGCCAGTCATGGTGAAAATGAA
AAAAATGAATTTCATCCTAATATAACCGAGTGGATAACAATCAACGACAGAACAACATATAAAGAAATATATCCATATATGCCTG
AAAGAGTTATCGATAACTTGATGAGTGAAAAGTCATATATATCAATAGTCAACTGGGATCAGATACACAATAACCCAGAAAACC
TTAGAAAAATGATTGAGTTAACATATCAAGATAAAGATAAGTGCGAATACGTTTTTTCTACCCCAATGAGAATGCGTAATGGCT
AGCAGAAACAGTAAAGGGGAGTATGATACTCCCCTTTACTTGTTACCAATCCCATTTGGTGGTGTTCACGTTAAATGTTTGTTG
AACCCGTGATGTGAAAAAATCATTTACTACTTTAAAATCGTTACATATTTTACTGTAGTTATAGTATTGCTTTTGTATTTTATTTATA
GGCGTCAGGTGAACTTTTAATGGTGTATTAGTTATAACATCAATAACTTCATCCTTATCTGATATATATTTATACCATCTTTTATAAT
TATTTTGATTTTTAGTTAAAAATTTATTTAACACCATTAACATATCATTGCTAATATTATATAACTCATTGTCTTTTTTTGGATTCAAT
AGTTTCATTTCATCTCTAATAAATTGATATGTCTGATAATATGATGAGATAACTTCATCGAATGCGTCCACATTTTTCTTCAACTTA
AAGATAGCTCTTTTTGAATCCAGATGATTTTTTATACTTTTGCGATATAACGTACCACTTTCATTGAACAGAACATTAACGCCGC
CTAAACTTAATTTCTCTACTTTAAATGAGTATTTTTTTAAAAGATATAACGCTACAATTAGCCATATAAAAATAGATATTAGTAGTA
TATATATAATACCAGTTACTAAAGGTTTGTTTTTATTAAATACGGTGTAAATTGAAGGTACATATAATGAAAGATCCACTGTACAC
ATTACAGCAATTACCGCAATGAAAGCTATCGTCCAAATTATAACAAATGTTCTCACAATTCCACCTTGTCAGTTGTAAGATAATC
AATCATTGATTGGTGAACGTTCTGAATTGATAATTTTATATACGGTTGAACGCGCAATACCCATATGCTTAGGGAAGGTGCGAA
CAAGTCCCTGATATGAGATCATGTTTGTCATCTGGAGCCATGGAACAGGGTTCATTATGAGTCATCAACTTACCTTCGCCGACA
GTGAATTCAGCAGTAAGCGCCGTCAGACCAGAAAAGAGATTTTCTTGTCCCGCATGGAGCAGATTCTGCCATGGCAAAACAT
GGTGGAAGTCATCGAGCCGTTTTACCCCAAGGCTGGTAATGGCCGGCGACCTTATCCGCTGGAAACCATGCTACGCATTCACT
GCATGCAGCATTGGTACAACCTGAGCGATGGCGCGATGGAAGATGCTCTGTACGAAATCGCCTCCATGCGTCTGTTTGCCCGG
TTATCCCTGGATAGCGCCCTGCCGGACCGCACCACCATCATGAATTTCCGCCACCTGCTGGAGCAGCATCAACTGGCCCGCCA
ATTGTTCAAGACCATCAATCGCTGGCTGGCCGAAGCAGGCGTCATGATGACTCAAGGCACCTTGGTCGATGCCACCATCATTG
AGGCACCCAGCTCGACCAAGAACAAAGAGCAGCAACGCGATCCGGAGATGTATCAGACCAAGAAAGGCAATCAGTGGCAC
TTTGGCATGAAGGCCCACATTGGTGTCGATGCCAAGAGTGGCCTGACCCACAGCCTAGTCACCACCGCGGCCAACGAGCATG
ACCTCAATCAGCTGGGTAATCTGCTGCATGGAGAGGAGCAATTTGTCTCAGCCGATGCCGGCTACCAAGGGGCGCCACAGCG
CGAGGAGCTGGCCGAGGTGGATGTGGACTGGCTGATCGCCGAGCGCCCCGGCAAGGTAAGAACCTTGAAACAGCATCCAC
GCAAGAACAAAACGGCCATCAACATCGAATACATGAAAGCCAGCATCCGGGCCAAGGTGGAGCACCCATTTCGCATCATCAA
GCGACAGTTCGGCTTCGTGAAAGCCAGATACAAGGGGTTGCTGAAAAACGATAACCAACTGGCGATGTTATTCACGCTGGCC
AACCTGTTTCGGGCGGACCAAATGATACGTCAGTGGGAGAGATCTCACTAAAAACTGTGGATAACGCCTTAAATGGCGAAGA
AACGGTCTAAATAGGCTGATTCAAGGCATTTACGGGAGAAAAAATCGGCTCAAACATGAAGAAATGAAATGACTGAGTCAGC
CGAGAAGAATTTCCCCGCTTATTCGCACCTTCCTTAGCTATTTCAGTTGCTCCAGTTCCTTTATCATGCATAAATTTTATTTTATTC
CTGTCTATTTTCCGTTTTCTGCCAAATTTAATTCCTTTAGACTTGGCCTCAATTCTTCCTTCATTTGTTCGTTCAAAGATGCGAGT
ACGTTCTGCTTGTGCCACTGCTGATAGAATTGTGACGATCATCTTCCCCATTTCTCCATCGGTACTGATCCCATCATCAATGAATC
GGACTGCCACACCCAAGGCATCGAATTCTTTTATTAGTTGAATCATATCGGCAGTGTCACGACCAAGACGGTCTAGCTTCTTCA
CCAGAATGACATCTCCATCTTCCACCTTCATGCGCAACAGCTCAAGTCCTTCCCTGGCAGCTGAACTTCCCGATGCCTTGTCCG
TAAAAATACGGTTGGCTTTTACCCCCGCGTTCTTAAGTGCTCTAATTTGAATATCTAGGGACTGCTGACTGGTTGAGACCCGTG
CGTAACCAAAAAGTCGCATAAAAATGTACCTTAAATCGAATATCAGACACCTTGTGTCTATTATGGCAAAAATACGATTTAATAG
ACAGTCCATGCCTGCCGTTTTATGCTGTCTTATAAATTATAACATTTCGGACGGTTGCGAAATTGTTAATATATAACCGTCAGGCA
GGAAGGCCTATATGCCCGTCGATTTTCTGACCACTGAGCAGGAACTTAATTATGGTCGCTATGTTGCAGAACCCAATGACGTG
CAGCTGGCGCGCTATTTTCATCTTGATGAGCGAGATCTTGCCTTCATTAACCAGCGACGGGGAAGGCATAACCGGCTGGGAA

TTGCGCTTCAGCTCACCACCGCCCGTTTTCTGGGCACCTTTCTGACAGATTTAACCCGGGTTCTGCCGGGTGTTCAGCAATTT
GTCGCGGTGCAGCTTAATATCCGCCGTCCGGAAGTCCTTTCCCGCTACGCAGAACGGGATACCACCCTCAGAGAGCATACTGC
GCTGATTAAGGAATATTACGGTTACCATGAATTTGGCGATTTCCCCTGGTCTTTCCGCCTGAAGCGCCTGCTATACACCCGTGC
ATGGCTCAGTAATGAGCGACCCGGCCTGATGTTTGATTTTGCCACCGCCTGGTTGCTTCAGAATAAGGTCCTGCTGCCCGGAG
CAACCACACTTGTACGTCTCGTCAGTGAAATACGTGAACGGGCAAATCAGCAACTTTGGAAGAAACTGGCCGCGTTGCCGGA
CAGCTGGCAGACCGCCCGCGTGACTGAGCTGCTGGACATTCCTGAGGGGCAGCGAATTTCACCGCTGGAGCAACTGAAAAA
GGGACCTGTCACCGTCAGCGGTCCGGCATTTACCGAAGCGCTGGAGCGGTATATCCGGCTGCGAAACCTGGAGTTTTCCCGA
CTGAGTTTTACCGGTCTGCCCGCTATACAACTACGTAATCTGGCCCGTTACGCAGGCATGGCGTCGGTAAAATATATCGCGCGA
ATGCCACAGCAGAGAAAGCTGGCCGTACTCACCGCATTCGTTAAAGCACAGGAGACTGCGGCGCTGGATGAAGCCGTTGAT
GTGCTCGATATGCTTATACTGGATATCACTCGTGCGGCGAAGAAAACGGGGCAGAAAAAACGGCTCAGGACGCTGAAAGATC
TTGACCGTGCTGCGCTACTGCTGGCGCAGGCATGTTCATTGTTGCTAGCTGAGCAGGCTGACGATGCTGAACTGAGGGAGAC
CATATTCAGCAGCATACCGAAAAGTAGGCTGGCAGAATCCGTCAGCAAGGTAAATGAGCTGGCCCGGCCTCAGAACAACAAT
TTCCATGACGAAATGGTGGAACAGTACGGGCGGGTAAAGCGTTTTCTTCCGGCGGTATTGCGCGACCTGCATTTCCAGGCAG
CGCCAGCCGGAGAACATACGCTGTCCGCCATTCATTATCTGACCGAACTGAACGGCTCGAAAAAGCGCATCCTGGACGATGC
GCCTGAACATATTATTACCGGCCCCTGGAAACGCCTGGTATACGATGCGGAGGGCCGGATACAGCGTGCCGGTTACTCGCTTT
GTCTGCTGGAGCGCCTTCAGGATGCACTACGCCGACGGGACATCTGGCTCGAAAACAGCGATCGCTGGGGAAATCCCCGCG
AGAAGCTGTTGCAGGGGGAAGAATGGCAGGTTCAGCGGGTCCCCATCTGTCGGGCGCTGGGACATCCCACTGATGGACATA
AAGGCGTGCAACAGCTGGCGGTCCAACTGGATAAAACCTGGAAAGCCGTCGCATCTCGCTTTGAAGGAAATGCGGAGGTTA
ATATCTGCCATGACGGTAAATATCCTTCCCTGACTATCAGCAGCCTGGAGAAACTGGAGGAGCCACCATCGTTGCATCGTCTCA
ACAGTCGGGTAAGGCAGCTACTCCCGCCGGTAGATTTGACGGAACTGTTGCTTGAGATAGATGCCAGAACGGGCTTTACACG
TGAGTTTACGCATGTCAGTGAATCCGGGGCCCGGGCGCAGGATCTGCACATCAGCCTGTGCGCGGTCCTGATGGCTGAGGCT
TGCAATATCGGGCTGGAACCGCTGATAAAGCACAATATACCGGCGCTGACGCGCCACCGGCTCAGTTGGGTGAAACAGAATT
ACCTCCGGGCAGAAACGCTGGTCAGCGCCAATGTCCGCCTGGTTGATTTTCAGTCCACACTGGAGCTTGCTGGCCGCTGGG
GTGGCGGCGAAGTGGCTTCAGCTGACGGCATGCGCTTTGTCACGCCGGTGAAAACCGTCAATTCAGGACCTAACAGAAAAT
ATTTTGGTTCCGGACGTGGCATCACCTGGTACAACTTCGTCTCTGATCAGTATTCTGGATTCCACGGCATCGTTGTTCCCGGCA
CATTACGTGATTCCATTTTTGTGCTGGAAGGCCTTCTGGAGCAGCAGACAGGGCTGAATCCGGTTGAGATCATGACAGACAC
AGCCGGTACCAGCGACATTATTTTTGGCCTCTTCTGGCTGCTGGGATACCAGTTTTCTCCTCGGCTTGCCGATGCCGGTGAAG
CAGTATTCTGGCGGGTGGATAAATCGGCAAATTACGGTGCACTAGACGAACTGGCTCGTGGTTGTGCCGATCTGTCGAAAGC
CGAGTATCAGTGGGATGAGATGATGCGAGCCGCCGGTTCGCTGAAACTGGGTACCATTCATGCTTCAGAACTCATTCGCTCTT
TGCTGAAAAGCTCGCGCCCATCAGGGTTGGCACAGGCGATCATGGAAGTGGGGCGCGTCAACAAGACGCTGTACCTTCTAA
ATTATATTGATGATGAAGATTATCGTCGGCGGATCCTGACGCAGCTAAACCGGGGAGAAGGCCGCCATGCTGTGGCGAGGGC
GATCTGCTACGGGCAGCGCGGTGAGATCAGAAAGCGCTATCGTGAAGGTCAGGAAGATCAACTGGGTGCACTGGGCCTCGT
CACTAACGCAGTGGTACTGTGGAACACACTTTATATGCAGGAAGCCCTGAGCTGGATGCGCAGGAATGGAGAAGAAACCGG
GGGTGAAGATATCGTCCGGTTATCCCCACTGATGCACGGGCATATCAATATGCTGGGGCATTATACGTTCACGCTACCGGAGGA
TATTTTGAAGGGGGAACTGAGAGAATTAAATTTTAATTTAAACAATGAATTACCTTCTTAGCGTACGTTTTCGTTCCATTGGCCC
TCAAACCCCGTATAAAAGATCAGCTAAATTATGTGTATTGCACAATACATATATGTGAGGTTAGCAGTGAATTTGCCTACGCCCG
AAACCTACGATGAACTTCAGAGAGCCTACGATTTTTTCAATGAGAAGCTATTCAGCAACGAGCTGCCGCCATGCCTGATAACG
TTGCAGCGTGAGAAGCGAACGTATGGCTATTGTTCCTTTAAGCGTTTCGTCGGCCGTGAGAGTGGGTACACGGTAGACGAGA
TCGCTATGAATCCGGTGTATTTCTCGATCAGAACCATAAAGGCCACGCTTTCAACACTGGTGCATGAGATGGTTCATCAGTGGC
AATTCCATTTTGGCGAGCCTGGCCGCCGTGGCTATCACAACAAACAGTGGGCGGCCCGGATGGAACGGGTAGGACTAATGC
CTTCTGATACCGGCGAACCGGGAGGCAGGAAAGTGGGCCAGAGCATGACCCATTATATTATTGCCGGTGGCCCTTTCGATATG
GCCTGTGATGAACTGCTGACAGGCCATTTCCAGCTTTCCTGGATGGACAGGTTTCCGCCTTACCAGCCTAAGCCTGGCGCTGT
GCTAAGCCCTACAGGAAAAGGCTATATTGACGACGAGGAAGATGATAGCGAACACGAACAGGAGGTGGAGGAAGGGCGCG
ACCCGGTTGAACTCGACGACGAGATCATAGAGGCCATGCGATTTGTAACCCCACCGCCTGAAGCACCGGTGAACAAAACAAA
CCGGGAAAAGTACAGCTGCCCGGTGTGTCATATCAATCTCTGGGGTAAACCGGGGATAGTGGTTTACTGTGGTGGCGAGCAC
TGTAATAAAGCCGCGTTAGTAGTCTTAAAATAAAGTCCTTTCGGACTTTATTTTTTTTCCATTTCCGAGGTCGTGATGTTATTAAT

GCTGTACTTCGCGGCTTCTTTTAAAACAGTTTCAGCAAGGCTTGCTGGTATCCAGACCTGAACTAATTTTAATGGTTCGCCGTT
CTCGGCTTTAAGAGTGGTGTTCTGGTACAAATCCCAGATTCGCTTAACGGTGCTGGAAATGTTTTGCTTGGAACGGCCTACTC
GCGTGGCTACGTCTGATGATTTCTCACCTTTGACAAGCACGGAATAGCCAATATCTGTTGTGATGTGTGCAAAGGAAGCCATT
TGCGGCAGCAGCTGTTTCCATTCTGTTTCTGAAATTCTGTTTTTCTGAGCCATCTGTGGCGCCTCCGTAGTTTTGGTTACAGAA
AGGATATACTCAGAATAAACAGGGGTCAATACAAGTACGATTTTTATAAACTTTATTTTATTTGAGGGTGAGGCCCGGTGCGGC
AGCAGCGCGGGCCTCGATGGTGCCGCGAAGGTGCTGGCGCCATGCTCGGATTAAAACATGAACCGTGAAGAACTGCGAAAC
TTGTTTTCGCGGTTCTGAGGGGTTGACCGAGCCGCGAAGCGGCGCTGGTAAGCGATGATATGCACATATCCACAGGCATATTT
TTAAAAGGGGCACTGTTGCAAAGTTAGCGATGAGGCAGCCTTTTGTCTTATTCAAAGGCCTTACATTTCAAAAACTCTGCTTA
CCAGGCGCATTTCGCCCAGGGGATCACCATAATAAAATGCTGAGGCCTGGCCTTTGCGTAGTGCACGCATCACCTCAATACCT
TTGATGGTGGCGTAAGCCGTCTTCATGGATTTAAATCCCAGCGTGGCGCCGATTATCCGTTTCAGTTTGCCATGATCGCATTCA
ATCACGTTGTTCCGGTACTTAATCTGTCGGTGTTCAACGTCAGACGGGCACCGGCCTTCGCGTTTGAGCAGAGCAAGCGCGC
GACCATAGGCGGGCGCTTTATCCGTGTTGATGAATCGCGGGATCTGCCACTTCTTCACGTTGTTGAGGATTTTACCCAGAAAC
CGGTATGCAGCTTTGCTGTTACGACGGGAGGAGAGATAAAAATCGACAGTGCGGCCCCGGCTGTCGACGGCCCGGTACAGA
TACGCCCAGCGGCCATTGACCTTCACGTAGGTTTCATCCATGTGCCACGGGCAAAGATCGGAAGGGTTACGCCAGTACCAGC
GCAGCCGTTTTTCCATTTCAGGCGCATAACGCTGAACCCAGCGGTAAATCGTGGAGTGATCGACATTCACTCCGCGTTCAGCC
AGCATCTCCTGCAGCTCACGGTAACTGATGCCGTATTTGCAGTACCAGCGTACGGCCCACAGAATGATGTCACGCTGAAAATG
CCGGCCTTTGAATGGGTTCATGTGCAGCTCCATCAGCAAAAGGGGATGATAAGTTTATCACCACCGACTATTTGCAACAGTGC
CGGTCGCCGGGAGTCAGCAGATCGACGTCAACGCCGAGCAGCGATTTCAGTTCTTCTTCCAAATCGCCCAAGTCCAACAACG
TGGCACCGGGCAGCGCATCGACCAACAGGTCGAGGTCGCTGCCATCCCGGTCGGTGCCATGCAGCACCGAGCCGAAGACG
CGCGGGTTCGCGGCGCGAAAGCGGCCTACCGCTTCACGCACTGCGCTTCGCTTCATGTCAAGCACAACAGACGGTCGCATG
CGCATCCTTTCTTATCGAAACTCGTTGAGATGATATGCAATCAAGAATAGAATTTCAAGAACTCACAAAGTAACGCGGTGGTTA
ATATCCTGTACCCACGGATTGCCCTTAGCGCTGCCTATATCGGCTAAAGCACTCCGGTAGCTTGATTCACCCCACGGCCACGGC
AGGATCTTGGCCGTCGCAAGCGCCAGGGGAAAATCTTCAGCTGCAAGCCTGAGTGATTTCATGTGCGTGTAATCCATCGCCC
AGATGATTTTTGTGAAGAAGAACTCGCGCTCGTTCATGTCCGGGCGCGCGTCCTGGCCCTCCGTGCCAACGGCCAGCAAGTA
ATCGGCCTGAATTGGCAGTATCAGCGCGCGTAGTAAGTCATGGATCGCCGGTTGCGGCAGTCTGCGCGCCAGATTAATTACCC
GGTCGAACTTCAGTTTATCCTGCTTGCGCTTGTCGGTCTGCTCCATCAGATTTCATGGCCCCCTTCTTCATGCTCATGCTCATGG
GTGTGTTCTTTTCCGGTATGGCTCTGTTCCGCCTGAGACGTCTGCGGCATGGCGTAATCGTCGTAAATGCTGCTGTCAAAGTCG
TAGTCTGATGCTTCGGCATAATGCTCGTAATCGGCATACTCCTGCGCGCTCCACTGCTGATCGTCGGCAGCAGCATAATCATGG
GCCAGCTCTGCATCATTTTGCTGTGCTTCATGACGCCGCAGGCCAACGGAATCATCCATAGGGTTCTGCTTAAGATGAAAGGC
GTCCTCTGCGTTGCTCACCGGCTGATAATCAGTGCCGGTTGTCATGTTATGTTCATCGGGTTTCTGGTTAAACGCCATGCTTTCC
CCCGTGGCTTCTGGCAGACCTTTTTCAGCTGATCGGGTTTCTAAACTGGTATCGCGGCCAATATCCTTAAACCTGGCCTCAAGC
CCAAAGAAACGGTCAATTTCTGCGGCCGTGGTTTTCGGGCTGTCGCGGCTCACGCTCGATGCCAAAGATTTTTTATCGTCGGT
AAAAATTTCCACCTCATGACGCGCACGCGAAATACCAACATAAAAAACGTCCTTAGAAGTGGTAAGCGATTTGGTATCTATGTT
GAACAACACGCGATCACAGGTAAGCCCTTGGGATTTGTGGACGGTGGTTGCATAAGCATAGGAAAGATAAGAAGCCTGTTTT
TTGTCCAGCTCAACCGTGCGCCCTTTTTTGTCCTCAAGCGTCAGTTTTTCACCCTCCACGGTTTTCACCGTGAAGCGGTCGCC
GTTGGCAACGTCCAGCGTTTTATCGTTACGCGTTACCATAACCTTATCGCCCGGCGCCAGTTCGGCGCTGACTGCCTGGTATAC
AGACAGCTTGGTGTGTGTACGCGGGCTGAAAGCGATCTGCTCACCGCTGCTGCTTTCAACCGTCAATTTGTTGCCCGGCCCG
GTATCAAGAACCTGGTAAGACTCGCCCCGCTTCATACCATTTTTGTAATCCTGTTCGGGGATAATGATTTGCCCTTTACTGAAAT
AACGGCTGTCGCGGCGTTCCGCCTGTGTCGAATCCACGCGGTCAAGTAGCGTGAACGTTTCGCCGGTTCCGGCAAGCCCCA
GATTGCCCCGGATGTAGTCATTGAGGGTTTTGCGTGAGGCGTTCGTACCAGAGATTATCAGGGTGGCATCCTGTTGTTCTGAG
GACAGAGACAGGTAGCGATCGGCAAGTTGAGCGAGTCGGGGCGCTTCTTCCTTCAGTTCGTTCACGCCGGTGATATTTTTCA
GGGCGCGCGCGGCATTACCTTCAGCGGCATACTTAACCGCCTCAAGCAAAACTTCATTCTTCTGTCGCTGAATGTCTTTCATGT
AGCTGGTCTGCATATCTGCTTTAATCAGCTGCTCAAAAGGCTTACCGGCTTCTACCGCTTTCGTCTGTGACGTATCCCCCAGGA
ATACCGCGCGAGCGTTATGCTTCTCGATCACCTCCATCAGCTGTTTCATCTGTCGGGCGGGTATAACCCCGGCTTCATCAATGA
ATACGACTGATTTTTCATCCAGCTTTTTATCCTTCGCTTTGAGGAAAGCGGCAACGGTGCGGGCCGGTAATCCATCATCTTCAA
GCGCTTTTTTCTGTGTCCCATAGGGGGCCAGCGCCGTGACCTTCAGCCCTTGTGACTCCAGCAGCTCTTTAGCGGCCATCGTC

ATATAGCTTTTACCGGTACCGGCGTAACCATGTGCGGCGACAAACCGATCTTTGCTCGTCACAATTTCTGTAACCGCGCGCATC
TGCTCCTTCTTGAGGGTTTTCCCGGCAAGCAGCTGGCCTGCAATCTCTGCGGTCAGCTGTCGCGGCATCTGCCCCCGGCCGC
GTGATTCGATAGTCAGAATGGAACGCTCAAGGCGAATACCCTCCACGGTAGTGACGCGGTGGCTGGTCTTTTTAAGCCTGCC
GTTTTTAATACCATCATCTACCGCAAAACGGGCTTTATCCGCACGCATCCCGCTATTCGTCAGCGAGTCGATCCACTCTTTGCGC
GTCAGAGTTTCGGCCATAACTGAAGCACCGACCTTCAGAGTTGATTGATACCGGGCTTCGCCCTCGATGATGGCGCCCTTCTG
TACCGCCTTCAGGTACGCTTTTTCAACATCGGCTATTGTGGCATGGCCCAGCACCTGCTTATTAGCGATTTGAATCAGCTTCTG
GCGTTCAAAGCTGGCATCGCGCTCTGACAGCGACTTAACTGCAAACTGGATAGCCCGGTCAGCTTTAACCTCCGGGCTGGTA
AAATCCGGGGCCATGTTGCGCGCTATATCAGCCTCCAGAGGTTTACCGTGTCCCTGCCATTCACGGTTATCAAAATCAATGCCG
AGCGTTTTGGCGCGGCTGGCCCATTCCTGGTGAATTTCTTCACGGGAATGCTCTGTTTTCTTTTCACGCGTAGCCATCGAGAC
GCGGCTTTTCGTCTGAGCATCGGCGGTTTCCCGCGTCAGACCCATTGCAGCGAGTCCCTTTTCAATTTGCTCCGACCGGCGG
GAAAAAGCGCGAATCTGTTCATCTGAAAAATGGGCCATATCGAACGTGTTATTTTTGCTGTTGTAACGCAGCTCATAACCGGCT
TTGGTCAACTCCAACGCCAGCTCCTGTTTGTAAACATCGCCCAGGTGCATTTTGTTACGCATCAGCTCATCATTTTTGAGCGCG
CGCCACTGGCCGTCCTCGCGCTGGGTCATGTTCATGACAAAAGCGTGTGTGTGCAAATCAGGATCTAGCGCCCTGGAAGTTT
CGTGGCGGAAAGTAGCGACGACAAGGTTATTGGTATTCTGGGTTACTGATTTCCCCTGGCGAGTCGTCCGGGCCTGCGCGAG
TTTTTCAGCTTCACGCACAGCAGCGGCAACAGCTTTTTCATGAGCCTCGATAATGGTTTTATCGCCGTGTATCAGCGCCTGCAT
GGATACCCCTTTAGGCGCTGAAAACGTCAGGTCGTAGCCCAGACGCTCTTTTTTGGCATCACCCACGTGTCGCTGCATATGCG
TGAAGGTATCTATCTCTCCGACAAGCAGCTCTTTAAACCGGGCTGATTCAACGTCCCCGGATAAGCCGAGGGCTTCAGCTCCG
GTTCCCTGCCAGGACGTGAATGATGAATCCTTACTGTAGTAATCATCCTTTGCATCAGAGTAGTAGCCCACAACGCTAGTGACG
TTCTGGCGGGTAATCGTGGTTATATCAAGCATCAGATCTCCCTCAGTTCAATGCCAGGAACAGGGTTTTTGCGATGGTATTTAA
CGTGTTTAGCCTTGAACTTAGCGACGGGCATATCACCAGGCAACGCCAGATAGCCGGTGAGGTTTGGCAACATTGATATTTCG
GTAGGCGTTACGGCACGAACAACTTTAACGTCGCGGCGTTTACGGACAATCCAGGGCTTCTGAGGATCGGATTCTTTACGCT
CAACTTCGCCTTCTATCTCACCGAGTGAGCGCGACATTTGATCCAACGTTTCATCACCGAGACGGCTGCCGCCCAGCACGATG
TTAGAACGCATGTTAGCCAGAATTGTCTGAGCCATATCCCGACCATAAACCTTAACCAGCTGAGAATAGGTTTGATAGCCAGCA
TAAACACACAGACCGCTTTTACGCCCTTTGGTCAGTGCATCGTTGAGGTTTGGCAGAAACTGGAGTGATTCCAGCTCGTCAAT
AAATACATTAATGCGGCTTTCTTTTTCACCCATACCCAGCACGATAGAAAAAATCGAATCCAGCCAGCAGGAAATTAGCGGATT
AAGTGACCTTTTCATTTCTTCCTGCCAGGTGATAAACAGGGTTCCCGGCTTTCCATCATCAAGCCAGTCACGCAGGGAAAAAT
TACCTTCCGGCATTTTCAAATGTGGGGCAAGATTCTTACTGAGAACAAATCGCGCGCTTCCAACTGCTTTTTCAGACCCGGAA
AAAATAGCTTCGGCAGGCGTCCCCATTAAAAATTCTTTTAATTTTTTCTGGTCAACGTTACAGGCCCAGTGAATAACTTCTTCC
ATAGTTACTGTGCTGTATAGGCTGTGAAGTTTTTTCGAAACTTCACTAAAAATAAGACGGCCATAGCCGAACCATTCTTCAGTA
GCCATATCAGGGCTTTCCTGAACAATAGAGTTCACTAAACGCTCGTAATCATATGAACGGCGAATTTCATTGAAAAACACCCAG
CCTTCAGTGCGTTTATCATAGGCGTTTAAAATAACATCGCCGGGACGATAGAAATTCTTTAAGAACCCCCCATTTGGATCTAAA
GCAATATTTTTGCCGCCTCTAATGATGCTCTTAAATAACAGTTCATTGAAAATTGTGGTTTTACCAGTACCGGTTGTACCGGCAA
TCGAAAAATGCAAGTTCTCAGCGTATGTAGGTATGGGGATATTAGCCACGGTTAACTGGTTGACACCTCTTTCGCGTGTTTTAT
CAGCGAGTGTTCTGGCGCGAACAAGCTCTGTACCACGATAAATCTTTTTGAATCTTTCGCCTTTAAACACGCGTGATTTATCAT
AAATGATAAAAGCGATCAGACCGCCAACACCAATAAACCAGCCAGCAATTAAAGCTGACCATAAAGGCCATAGCGAAAAAGT
ATTCTTAACCAGATACGGAATCAGGTATTTAGCCGTGGATGGATCAATACCGTAGGTAAATTTTGCAACTAGAAACCATACCATC
ACTGGAGGCAAAGTAATTGCAAATAAAAATGCTAAGCCTCTTTCTCTATCGTCCATTTCAGCGCTCCTTTTTTGGTTCCCAGAC
TTTGTAGCCGTTACGTTCAACCTCTGCTTTTGCCGCTTTGGTTTTGCCCGGTTCTGCTATCGAGCGCAGGAGGATTAGCGTTTC
AATAGCGAGTGATTCATGCAACATCATCTGTCCTGCCGGTGGGAATTTTACGCCAGATAGCGTTTCGGTTATTGCCTTCAGCTC
ATCGCGCAGTGGGCCAAAATCCGCATCTGAAGCACGGTCAAAAAGATAATCCAGTTTGCGATTTACGTCGCTCAGCCGGTCG
GCAACTATTTTCAACCCGGACTCCCGATCACCTGGGCCAGCTTCAATGCAGCGCCGCAGATAATCTGACCGATTACCTCCTGA
AACCAGGTCTATATAGGCCAAAAGTTCATCTGATACTTTTGCGGTTATTATTGGCATTCAGTCCTCACATTGTGCATTTCTTAAAC
AAAAAATTGGGATCTAACAAGCTGAAATCTTAGTATTACCAAAGTAATAAAGCAAACTCATTATAAAACAATGGGTTATTGGGT
GTTTTTAATACCTAATTATTACCGAATATTGACGCTATTTATTTTTTTATCTTTTAAATCAGTACGATAGCGTGATTTATCGCGCTGC
GTTAGGTGTATAGCAGGTTAAGGAAAAAAAATCATCTTTTTTGGTAGGAGCGACCTCCGTAGGTTAAGGGTCATTTGGCTAAA
AAGCGTCCTATTCTTTGATGGTCATGCTTGCATGACCATCTGAGCAACCAAAAACTACAGATAAACTACAGAGAAACTACAGAT

AAACTACAAAAAACGATTTACCTTAGCGTTGTCAGACTACTAATAGACTACAAGGAAACTACAAAGAAACTACAAAGAAACTA
CAAAGAAACTACAAATAGACTACTAAAACCGTGGCAGACTACTAATAGACTACAAGAAAACTACAAATAAACTACAAAACTGG
ATTGACCCCTTCTTACGAGTGTTGTAGAGTCATCTTCATACAACGGAGGGGGTTATGAATAAACCAGCAGATCTGAAACCCCG
CAACTTATCGGCTGCTGTCAGATTGCGCCTAAATGAAATCGAGAACTGGCTGGACAGAGGGCTAACGCGGCATGAAATTGCT
GAAATCCTCGACAGCGAATACAGCTTTTCGGTAACAGCCAAAGGGCTTGAGATGGCACTGTATAGAACGCGGCAAAACCGA
AAAAATGTATTGCACAATACACATGATAAGAGTAGCGCGAAGGGTGCAGCGGAAAGTGTATTGCACAATACACAACCGTCTG
AGCCTGAAGCGCAGGAAAGTGAAAAAGCAGAGAGTCCCGGCATTATTGATAAAGAGTTCTTCAATAAAATCGGTGAGGATTT
CAACCCTAAGAAGTTCAACAAAAAATTCTGAGGTGATTTATGAAAGTAGCGGTAATTAATTACAGTGGCAGTGTTGGTAAAAC
ATTAATTTCATCTTACCTGTTAGCCCCGCGCCTGACTGGTGCAAAGTTCTATGCGGTAGAGACTATCAACCAGTCTGCTTCCGAT
CTGGGTATTGAAAATGTGACCAGTTTTAAAGGTGACGACTTCTCACGTTTGATTGAGGATATTGTTTTTGAAGATGCAGGCAT
TATTGATATTGGCGCGTCAAACGTTGAAGCGTTCCTGATGGCTATGTCTCGCTTTGACAGTGGCGCGAACGAATTTGATAAATA
TGTAATCCCGGTGACGCCGGATAATAAGGCGATTGATGAAAGCCTGAAAACGGCACACACGTTAAGTAAAGCGGGCGTGAG
CAGCAAGAAAATTATCTTTGTTCCAAACCGTATTAGTCCAGACAGTGAAGTAGAAGATGTGCTGGCGCCGGTGTTTGAGTTTG
TCAAAGAAACGAAGATTGGCAAAATAAGCAAGAAGGCTGTTATTTATAACAGTGAGGTTTTCGAATATCTGGCGTTTCACCGT
ATCTCATTCGAAGTATTGACCGCTGAAGATCCAGAAGAATTCAAATCCCGTGCAAAACAAACAACCGATGCTGACGAGCGCA
AAAAACTGGCACGCCGTTATACATACATGAAACAGGCGATTCCGGTAAAAGCTAATCTCGATAAAGCATATGCGGCTTTAATGG
GAGAATAAAATGGAAAAGCAGCCGGATAAATTAGAAGTTCTGATGGACTGGTTTTTAGGTGACGCGAAGGAAATCACCGCA
ACTCAGAAAGAAATGACGCAGAAACTTTCTGAGCTTTCGGAAAAGCTGGCAAAAGACACCGAAAGTTTAGGAGAGACGGC
AGACTCTTTTAAACGGGCTTTAGTAGAAAACCAGCGTTCAATTAGCCTGGCAATTAGTGATGATGCTAAGGCGCGCGAGGAA
TTTCTAACTAAATTCCGCCGCGCGCAGGCGTCCAGTGCTGAGACGTTTACCCGTCAGATCCTTTTTATTACAGCTGGCTGCACC
ATCGTGGGCGCCGCAGTAGGCGCCGCGATAGCGATACTTTTACTGAGATAAAGCAAACCGGGCGTGTCCCGGTTTTTTTGTC
AAGCGGAGCGCGGAGGCCGAAGGCCGGAGGCATTAGTGGCCGCCGCCCGCGTAAGCGGGGCGAGACGGGAACCGGCTC
GAAGCGCAGCACGGCAGAACGGCCCCGCAGGGGCAATGCCCGTTTTAATTCATCGTGACAGTCGCGCGTGACCATCACGGG
GAGAAAAATAATGAATGACCGACAGCGAGAACTGGCCCGTATACGCCAGGCCCGCCGCCGCGCGCGGCTCAAGGAAGAAG
GCACAAGCGTGACAGTCACGCTAACAAAACAGGAAGAAGCAATGTTGCAGGAGCTGTGCCGGGTTCGCCGTCCTGGACGA
ACGCCTTATTCAACGAACGAATTTTTCCAGCTGCTGCTTATCCGCAACTGGCAGCAGTGGCAGGAGCAGAAGGCACAGCTGG
GAAAATGCCAGGCTTGCGGAAAGCTGAAAGCGGAGGGGGGGTGCGAGGGTGAACGGAAAGGCGAAACCTTTAACTGCTG
GCTTGCCGTCGAAGCCAATGAACTAAATTTGTAGTGTATTGTGCAATACACATTTACACAGAAACAAAAACCACCGGCAATTC
CTGGAACCGGATACCTACGGCTATTCCTGGGTGAACGGTACTTTTTGCACCTGGGTGCGCTGAAAAAGCTGAATATGCAGGG
TGACGTTCGCGTGCTGTTCTGCTTTGTAGACTGAATGCGCCAGCTATACGCCTGACTGCTTAAACCTGGTAAAGTTCTGCAACC
GGCACTGACCGGAAAGCAAGGCAGGGAAGACCTAAGCCAGAAACCTTGACTGCTCCCCGCCCTTCAGGGCGGGGATTGCG
GATCATGTTCTTCTCTTTCAGGGATTCAACGCAGACAAGAAAGGCTTTCAATTTCCTATACGTGAACGGCCGCGCAGCGGAAG
AAACAAGCCCGGTCAATCCGGGCTTGTTTCTTTAGGCGGCTCAGAAATCGCCTAAAGGCCCGGCTTGGCCGGGCAGTCAGT
GCTATTTAGTTTGTTGCAGCAGCTGGCTTAATTTTGCCGCCAGTGCATACGTCGATTGGAAAGCGCCTTGCAGGCGCTGATTA
GGCAGCTGGTTGAACGCTTCGAGGCAGGCGCGCAGCAATAATTCTTTCTGAGATTCGACTTCTTTTTTCAGTTGGGAAGGGG
TGGTAACTGTAGTCATGCTTGCTCCTTAGTGAGCCGATATCGGCAATTTTTCGGGTGGCGGTGTTGCCTCCCGATGATTTAATT
ATCGGTGATTATGCTTTTAAAGTCAATACAGGTACGGAATTTATTTACCTGTTTTTATGCCCGTCAGGGCATGGAAGGCGACCG
CGCCGGACTCCACCGGACACCGGCCGCAAATCGCCGGAAACTGCGGGACTGACCGGAGCAACAGGCCAACCCCCCTCCCT
GCTAAGCCATAACCCAGCCCGCCGCCACGCAGCTGCCGCACGTCCCCCACGGGGGTGCGCAGTGGGCGCCGCGCGCCTGCG
CGCGGGTACGGCGGCCCGCCTGCGGGTCGCGGCGCCGTACTGCGAGTTAGCGGCCGCCGCGCGGCCGGTTACGGGGGAC
ACCGCACAGTCACGGCCAGTGCCCCGCTGAGCTGCACAATCCACGGATAACACAATAGCGCACTGGCAAAGGATGCCGACG
CCTGAAGGGCGTGGGCACCCCGAAGGGGCGGGGCGGCCGCTTGCGGCCGGGCGAGTCCGGCGCAGGGTGTGGCCTGCC
AAGCGGAGCGCGGAGGCCGAAGGCCGGAGGCGTTAGCGGCCGCTGCCCGCGTAAGCGGGGCGAGACGGGAACCGGCTC
GATGCGCAGCACAGCAGAGCGACCCCGAAGGGGTAACGCCCGGTGTGGCATCAGGATTTAGTGCAATGGCAGAACATGAG
CTGGAGAGATCACCGGCAAGCAGCAGCAAAGGGGCGGCACAGCCGCCCCGATGGCTGTTTGCCGATACCGGCGATTAATTA
GAGCGGTGTTTAATATCCCCCGCGTTGCGGGGGACTAGGTTTCAGCAAGTCATGTTAAATACGTGTCCATCATGTAAACTGAA

ATCCCCAATAAACAGATCCCGCGCATAGGCTACGATGTCAAAATATCGGGCTACGGATTCCGGAATATCATTCAGTAGACCGCT
ATCATTCAGGTATTCCTCTGCAAAAGTTTCTTCGTCCTTAGCTTCGCCCATATAGGCATCTCTAAACAGGTCGAAATCAGTGCTA
TTAAACAGATCAACAAAGGCCACAAACGCCGCTTCGTTACCTTCCTCGCGGGCTTGTTTAAAGCCGTTAATAAAATCCCAGTT
GATATGGCACTCTGACGCCATACCAGACGGAATACCCTCCCAATCTTGGAACATAAATTCTGGATCAGCCTCATTTGCGTGTAA
CTCGCGGCAGCGCTCGTAAAACTCCTCTGAGCTATCAAAATCGGTCAGATCGAGCCAGGCTCCCGCAATGCTTCCGCAGTTGT
ATTTATGGTAAGTGCCAACATAAACAGAAGGGGTCGTAATATCAGTCATGGTGTACTCCTTAAAGCGCCGATACCGGCAATTTT
TCGGGCGGCGGTATTGCCTCCCGATGATTTAATTATCGTTGATTATGCTTTTAAAGTCAATACAGATACGGAATTTATTTACCTGT
TTTTATGCCCGTCAGGGCATGGAAGGCGACCGCGCCGGACTCCACCGGACACCGGCCGCAAATCGCCGGAAACTGCGGGAC
TGACCGGAGCAACAGGCCAACCCCCCTCCCTGCTAAGCCATAACCCAGCCCGCCGCCACGCAGCTGCCGCACGTCCCCCACG
GGGGTGCGCAGTGGGCGCCGCGCGCCTGCGCGCGGGTACGGCGGCCCGCCTGCGGGTCGCGGCGCCGTACTGCGAGTTA
GCGGCCGCCGCGCGGCCGGTTACGGGGGACACCGCACCGTCACGGCCAGCGCCCCGCTGAGCTGCACAATCCACGGATAA
CACAATAGCGCACTGGCAAAGGATGCCGACGCCTGAAGGGCGTTGGCACCCCGAAGGGGCGGGGCGGCCGCTTGCGGCC
GGGCGAGTCCGGCGCAGGGTGTGGCCTGCCAAGCGGAGCGCGGAGGCCGAAGGCCGGAGGCGTTAGCGGCCGCTGCCC
GCGTAAGCGGGGCGAGACGGGAACCGGCTCGATGCGCAGCACAGCAGAGCGGCCCCGAAGGGGTAACGCCCTGTGTGGC
ATCAGGATTTAGCACAATGTCAGAACATAAACTGGAGAGATCACCGGCAAGCAGCAGCAAAGGGGCGGCACAGCCGCCCCG
ATGGCTGTTACTTGTCTTTGTCGCGTAGCACTTTGATTAGGCCGGTTACGGCCGTAATCAGAGCGGCCAGCGAGGTGATGATT
TGCGGTAGGTTTTCGAGGATGGTAGAGGTCATATAGCACCTGTAGAGAAGTTGGCGGGGTGTCGTTTCCGACGGCCGCACT
GTAACCGGGCGAATAAGGCAGGTTGTCAACAGCTTGAGCGAAGCGTCTGTTGACAACCTGCCGCGCCCGGTTTCACTGCGG
TCATAGGCGGAACGACCCACGCCAACGGAACGGCTTTATGACCGGGCAGCTGAGATACCGGCGAACCTGGCTGGCGGCTGA
CGCCAGCCGCCAAGCGCCAGCGCGGAGGGCAAAGCCCGGAGGCCAAGCGGAGCGCGGAGGCCGAAGGCCGGAGGCCG
GAGGCGTTAGCGGCCGCTGCCCGCGTAAGCGGGGCGAGACGGGAACCGGCTCGATGCGCAGCACAGCAGAGCGGCCCCG
AAGGGGTAACGCCCGGAGTCTGCCGCTGTTTATCTCTCGTTCCATCTGAAATCGGCGGTAAGGCCATTAAAAGGGTCAGTTTA
TCAGGGAGGCGTTAGCCCCCCATGTTGTTAATCATCAGGCAATATCGTCTTTGTAGCAGGCATAACCGAAGCTAAGCTCTGTTT
TCATATAGTGGCGGGCAAAGTCCCAGGCATCGTGGCCGAAGTCCTCATAATCTGCCAGGACGATTTCGCGGGCTTTGTGCCAT
TGCTGTACGGAAGAAAGCGGCAGAACAGGGCAGGGGTGCGACCAGTCAGTGACGGTGTTGCAGATCATATCTGCCAGACG
CTCCAGGGAGCCGTAAACCAGCTCTGTGCGCAGATCCGCCACCATGCGTTGTTTACGCAGTGATGCCAGATAATCAATCTCTT
TGGTTATATCAGAATTTAAGCGGGTCTGGTAATCCATGATGTACTCCTTTGCGCGCCGATACCGGCAATTTTGCGGGCGACGGT
GTTGCCTCCCGATGATTTAATTATCGGTGATTATGCCCTCAAAGTCAATATAAGTACGGAATATGCATGCATAATTTTATATCTTGC
AAAGCGTTCATAGAGTGCCTGAATCGCTTTCTGACAGCCTCAATAAAAAAAGGCGGGGATTCCCGCCTTTTTTCTTACAGCTG
CTTACGTGGCTTTTTACGCGTCATATACAACGGTATCGCGCAGTCTACCGCGTACAAAAAGCACGCCAGCGCGCCGCAACCGT
ACAGAAACGCAAGCGGCTTATTATCGAAGTAGCTGAAAACCCCTGTCGCCGCACACAGGCCCAGAACAGAGACGCAGGCGC
AGGTGATCTGCACCAGATCCCTGTATCCCGCACGAACGATAAACCAGGGCAGGGCAAGCGCAGCGGCGCTAATAATTAATGC
GAGAGGGACAAAAACGAGATAGTGATACATGTGAACTCCTTGATGGTTGCCGATACCGGCGATTGTTCGGGCGGCGGTATTG
CCACCCGATGATTTAATTAGAGGTTTTGCGCGTCCAGGAGATTGACCTGAGCCGGGGTAACGTGAAACTTTTCCCCTTTATGG
ATCACGTTATGCGGGGCGCTAATTTCATCACTGATAAAGCTAACCGGGTAACGTTTTTTACCGCAAATCCGCTCGCTAAACCAT
GCCACTTTTGCCGCTGGCCGATCCACTGGATGAACAATCACACCGGCCATGCTGCAACCCGTTGCGGGTTCGTCCAGCGTAAT
GCTTACCGGGACTGTATCCCGGTAAAAAAACTTAACCGGCGGCACACCTGCCTGCGTAGCGGCTGCGACAACTGCAAGCCCG
ATAACCGCTATCCGATTAATAAGCATTTTATTCCCCTTACTCATGCTGATATCACCTTGCCAGCTGTTACCAGTTTACGAAATTCA
CTTTCATGAATTTCACGCCCATGCTCAAGCGTTGAATACACGTTGCCGATAAGCCAGTTACCAGCCGTTTTTGTCTCGGTATACC
ACCATGCTTCTGTCAGAGTAAGCAGCGGTTCGCTATCCTCGCCCTTCTCATAGATCCAGATTTTAGTGACGTCTTTACCCGTTTC
GTTGCTGCCCTGAATGGTAGGGTCAAAGGTATGTTCAATCTCTATATCGAAGTAACGCTGAAGGAAGTTAGTAAAGTGCATGA
CGACTCCTGTAAGCGCCGATACCGGCAATTTTTCGGGTGGCGGTGTCGCCTCCCGATGATTTAATTATCGTTGATTATGCTTTTA
AAGTCAATACAGGTACGGAATTTATTTACCTGTTTTTATGCCCGTCAGGGCATGGAAGGCGACCGCGCCGGACTCCACCGGAC
ACCGGCCGCAAATCGCCGGAAACTGCGGGACTGACCGGAGCAACAGGCCAACCCCCCTCCCTGCTAAGCCATAACCCAGCC
CGCCGCCACGCAGCTGCCGCACGTCCCCCACGGGGGTGCGCAGTGGGCGCCGCGCGCCTGCGCGCGGGTACGGCGGCCCG
CCTGCGGGTCGCGGCGCCGTACTGCGAGTTAGCGGCCGCCGCGCGGCCGGTTACGGGGGACACCGCACCGTCACGGCCAG

CGCCCCACTGAGCTGCACAATCCACGGATAATGCAGGAGACGAATCATGATAGGAGGCTGAAGGGGAAATGAGCGGCAGCA
GGGGAAGGGGTTGCCAAGCGGAGCGCGGAGGCCGCAGGCCGGAGGCGTCAGTGGCAGCTGCCCGCGTGAGCGGGGCGA
GACGCGTAGCGGCTCGATGCGCAGCACAGCAGAACGGCCCCGGAGGGGTGACGTCCGGGGGTTCGCTTTTAAAGATTTTC
GACCACATCAGTAAATCGTAGTGACACCATGAAGCAAAAGTATCGTGCAGACCAGAATCAACAACAGTAACAGCAGACCTTT
TTTTCGACGTAATAAAACCGGCCCCAAAGCGCCCGCAAGAAGCACACTCAACACGATTTCCGTTATACTAATCGTATTCATAAT
CTCTCACGTTTCCCTTTTTAGAACTCTGCCACACACAGATAAAACCTTATAACAAGCTACAAAACCCGTTATTCAGACGCGGTA
ATGCCTAGTTTTTTTGCCAGTATTTCAGATGACAAAGAAACCCTTATCCACTTCCCGGTTACAAGGTGAATGATTGTGGCTTCA
TGCCCTTTAACAGAGTGGGATATTAACTCACGCTGAATGATATGCGTCTCTCCATCATTTCCCGTCACGGCAATTAGGCCCTCTG
TAGAACAGGGGTTGATACTACTAATGTTGGTGTTGTTTTCGGTTTTGTTCAGCATCGCTGATCCTCAAATATCGGTTTGTGTTAC
GTCTGCCGCTTTGCGCTGGATAAGCGACTTAAAGAAATCCGACGCCTTCAGAATATCGCTATCCTGGAAGTCGGGAAATGTCG
CTTCTGTCAGCTCCTGCGCCGTACCTGCCCGTATAACGGTTTCATCGCGGATCATCGTTCGCTTAACGCCATCAGCCTGTTTTTT
GCTGAAGGAGTGCCGGAAAATAATCGCGTATTGGCCGCCGCTATCCTGATGAACAGAAGTATCCCAGCGATGATTACCCTGTT
TACGATAGTGGTGATAGATTTGCTGGCCGTAGCGCTCCTGATCGGAATTACGGTAATCGAACATTAACAGTTCCAAAAGCATTA
ACCGATCTGGCAATTGCCAGGCGGTAGGGTGTTGAAGTGTCGCTAACATAGTTTCCCCTGAGCGTGACAGTCACGATAAGGC
GGGCTTTGCCCGCCTGGTTATCAGTTAATCAATGGCACGATAAATACGATTCTGGCTTTCGTTCTCCAGCGTATTAACGTACTCC
CGCAACAGGTGATACCGGTTAGCCATCGTTTCGTTCAGTTCGGCTTTGCCTTCTTCATATGCCAGGCCGCAAAAGTAACTGTA
GGCATACAGACAAACAATAATCCCTACTTCGCGCGCGCTGCATTCACCTTCAAAATAGTTAGGTAACGAGAGCCAAAGAGGTT
GGGGCGCTTCCATAAAAAACGCGCCATTGCTGGCCTGAAGGTATTCCCAATACCCTCCCTGGTAGTCTTTAGCGTAACGATTC
AGAAAGGACTGAATGAAGTGATCTGCGCTGAAGAAAGCGCCACGAAATGCCGCAGGCATGAAGTTCATGCGGGCGTTTTCA
GAAATGTAGCGGGCGGTGATTTCGATAGTTTCCATGATACTTCCTCTTAAAGCCGATACCGGCGATGGTTAAGCGGCAGGCAC
ATCACCTGCCACTTTTTAATTATCGTACAATGGGGCGTTAAAGTCAATATAAGTACGGATTATATTTACCTAATTTTATGCCCGTC
AGAGCATGGAAGGCGACCTCGCCGGACTCCACCGGACACCGGGGGCAAATCGCCGGAAACTGCGGGACTGACCGGAGCG
ACAGGCCACCCCCCCTCCCTGCTAGCCCGCCGCCACGCGGCCGGTTACAGGGGACACTGAGAAAACAGAAAGCCAACAAAC
ACTATATATAGCGTTCGTTGGCAGCTGAAGCAGCACTACATATAGTAGAGTACCTGTAAAACTTGCCAACCTGACCATAACAGC
GATACTGTATAAGTAAACAGTGATTTGGAAGATCGCTATGAAGGTCGATATTTTTGAAAGCTCCGGCGCCAGCCGGGTACACA
GCATCCCTTTTTATCTGCAAAGAATTTCTGCGGGGTTCCCCAGCCCGGCCCAGGGCTATGAAAAGCAGGAGTTAAACCTGCAT
GAGTATTGTGTTCGTCACCCTTCAGCAACTTACTTCCTACGGGTTTCTGGCTCGTCAATGGAAGATGGCCGCATCCATGATGGT
GACGTACTGGTTGTGGATCGCTCGCTGACGGCCAGCCACGGCTCAATCGTAGTCGCCTGCATCCATAATGAATTTACCGTGAA
GCGGCTACTGCTGAGGCCCAGACCCTGCCTGATGCCGATGAACAAAGATTTTCCTGTGTACTACATTGACCCGGATAATGAGA
GCGTTGAAATCTGGGGAGTGGTTACGCATTCCCTTATCGAGCATCCGGTATGTTTGCGCTGATTGATGTCAATGGCATGTACGC
CAGCTGTGAGCAGGCATTTAGGCCAGATCTGGCAAACCGAGCAGTGGCCGTTTTATCCAACAATGACGGCAACATTGTGGCC
CGTAATTACCTGGCGAAGAAAGCGGGCCTGAAAATGGGCGATCCGTACTTCAAAGTCAGACCCATAATCGAGCGTCATAACAT
CGCTATTTTTAGCTCTAATTACACTCTCTATGCCTCCATGTCGGCCCGGTTCGCGGCCGTAGTTGAGTCCCTTGCAAGCCACGTC
GAACAGTATTCAATCGACGAGCTTTTTGTTGACTGCAAAGGGATAACGGCCGCCATGAGCCTTGACGCTTTCGGGCGCCAAC
TGCGCGAGGAAGTCAGGCGACACACAACGCTGGTATGCGGGGTCGGTATTGCCCGTACTAAGACGCTGGCGAAGCTGTGTA
ACCACGCTGCAAAAACATGGCCCGCTACTGGCGGGGTGGTTGCTCTGGACGATGGCGCCAGACTGAAGAAATTAATGAGCA
TCCTGCCGGTTGCGGAAGTCTGGGGCGTCGGCCATCGTACAGAGAAAGCACTCGCCACAATGGGGATCAAAACGGTGCTGG
ATTTAGCCAGGGCAGATACGCGCCTAATCCGTAAAACATTCGGCGTTGTGCTTGAAAGAACGGTACGGGAGTTGCGCGGCGA
GGCTTGCTTCAGCCTGGAAGAAAACCCTCCTGCGAAGCAGCAGATTGTTGTGTCGCGCTCATTCGGCCAACGCGTAGAAACC
CTGACGGACATGCAGCAGGCTGTCACCGGATTTGCAGCGCGCGCAGCTGAAAAACTGCGTAATGAGAGGCAATACTGCCGC
GTCATAAGCGTCTTTATCCGTACCAGTCCTTATTCAGTGCGTGATACACAGTATGCCAATCAGGCAACCGAAAAACTGACGGTG
GCAACCCAGGACAGCCGCACGATAATTCAGGCAGCACAAGCCGCGCTGGCGCGGATCTGGCGGGAAGATATTGCGTATGCA
AAAGCAGGGGTCATGCTGGCAGATTTAGCGGGAAGGAGGCCCAGCTTGATTTATTCGACTCTGCTACGCCTTCAGCTGGCA
GCGAGGCTTTAATGGCTGTTCTTGATGGTATAAACCGGCGTGGAAAGAGCCAGCTTTTTTTTGCAGGCCAGGGCATCGATAA
CTCCTTTGCCATGCGTCGTCAGATGTTGTCACCTGATTACACGACAGACTGGCGCTCAATACCAATAGCCACCATCAAATAATTA
CCGGCGCCGTACACGGGCCGGTTAACCCCTCAACCGGCCGAAACAAGTTTCGGCACGGTTTCGCGGTTTTCGGTAAAAGCC

GTTTCCTCTGTATAAAAGATCAGCTAAATTATGTGTATTGCACAATACATATATGTGAGGTTAGCAGTGAATTTGCCTACGCCCG
AAACCTACGATGAACTTCAGAGAGCCTACGATTTTTTCAATGATAAGCTATTCAGCAACGAGCTGCCGCCATGCCTGATAACGT
TGCAGCGTGAGAAGCGAACGTATGGCTATTGTTCCTTTAAGCGTTTCGTCGGCCGTGAGAGTGGGTACACGGTAGACGAGAT
CGCTATGAATCCGGTGTATTTCTCGATCAGAACCATAAAGGCCACGCTTTCAACACTGGTGCATGAGATGGTTCATCAGTGGC
AATTCCATTTTGGCGAGCCTGGCCGCCGTGGCTATCACAACAAACAGTGGGCGGCCCGGATGGAACGGGTAGGACTAATGC
CTTCTGATACCGGCGAACCGGGAGGCAGGAAAGTGGGCCAGAGCATGACCCATTATATTATTGCCGGTGGCCCTTTCGATATG
GCCTGTGATGAACTGCTGACAGGCCATTTCCGGCTTTCCTGGATGGACAGGTTTCCGCCTTACCAGCCTAAGCCTGGCGCTGT
GCTAAGCCCTACAGGAAAAGGCTATATTGACGACGAGGAAGATGATAGCGAACACGAACAGGAGGTGGAGGAAGGGCGCG
ACCCGGTTGAACTCGACGACGAGATCATAGAGGCCATGCGATTTGTAACCCCACCGCCTGAAGCACCGGTGAACAAAACAAA
CCGGGAAAAGTACAGCTGCCCGGTGTGTCATATCAATCTCTGGGGTAAACCGGGGATAGTGGTTTACTGTGGTGGCGAGCAC
TGTAATAAAGCCGCGTTAGTAGTCTTAAAATAAAGTCCTTTCGGACTTTATTTTTTTTCCATTTCCGAGGTCGTGATGTTATTAAT
GCTGTACTTCGCGGCTTCTTTTAAAACAGTTTCAGCAAGGCTTGCTGGTATCCAGACCTGAACTAATTTTAATGGTTCGCCGTT
CTCGGCTTTAAGAGTGGTGTTCTGGTACAAATCCCAGATTCGCTTAACGGTGCTGGAAATGTTTTGCTTGGAACGGCCTACTC
GCATGGCTACGTCTGATGATTTCTCACCTTTGACAAGCACGGAATAGCCAATATCTGTTGTGATGTGTGCAAAGGAAGCCATTT
GCGGCAGCAGCTGTTTCCATTCTGTTTCTGAAATTCTGTTTTTCTGAGCCATCTGTGGCGCCTCCGTAGTTTTGGTTACAGAAA
GGATATACTCAGAATAAACAGGGGTCAATACAAGTACGATTTTTATAAACTTTATTTTATTTGAGGGTGAGGCCCGGTGCGGCA
GCAGCGCGGGCCTCGATGGTGCCGCGAAGGTGCTGGCGCCATGCTCGGATTAAAACATGAACCGTGAAGAACTGCGAAACT
TGTTTTCGCGGTTCTGAGGGGTTGACCGAGCCGCGAAGCGGCGCTGGTAAGCGATGATATGCACATATCCACAGGCATATTTT
TAAAAGGTATTTTATAGATTTTTTATCTTTTTAAAGTCTTTTAGAGCTATATAACTCATTGATTTAAAATCATAAATAAGTGTTATCT
CTGGGAATCCGCCCACCTTGTTATGGGAATTGGCCCACCTTACTATGGGAAACAGCCCACCTTACTATGGGAATTAGCCCACCT
TGTTATG

>pQEB1_inv1

GGAATTGGCCCACCTTAGACGAAACTGTAAAAAATGTATTTACTTGTTTGAACTTTGTGGTAGTGTGGAGAGTAATTTTTAACC
CACAAAGGCAAGGCGCATGGATAAGTTGCTGAACAAAAAGATAAAAGTTAAGCAGTCTAACGAGCTTACCGAAGCTGCTTAC
TACCTCTCGCTAAAAGCAAAGCGCGTTCTCTGGTTATGTCTTATGCAGACGTATTTCACAGCTTCAGTAAGCGAAGATGATGAT
GAGATGGCTGTACTCGGTGACTCTACTTTCAAAGTAAAGGTGGCTGACTATCAGCAAATTTTTCAGGTAAGCCGTAACCAGGC
TATCAAGGATGTTAAAGAAGGCGTGTTTGAGTTAAGCCGTTCTGCGGTAATCTTTTACCCGAAAGAGGGGCGTTTTGACTGC
GTCGCGCGCCCCTGGCTAACAGAGGCTGGCAGCCGATCAGCTCGTGGTATCTGGGAAATCGAATTTAACCATAAACTCCTGC
GGTACATTTACGGCCTGACGAACCAGTTCACCACCTACTCGCTCCGCGATTGTGGCAGTCTTCGAAATCCCCGGACGATCCGC
CTTTATGAAAGTCTTGCTCAATTCAAATCTTCAGGCTTATGGGTTACTACTCATGCTTGGTTAAATGACCGTTTCCTTTTGCCGG
AATCCCAACAGAAGAACTTGGCAGAGTTGAAACGATCTTTCCTTGATCCTGCACTCAAGCAGATAAATGAGAAAACACCTTTA
CTTGCTAAGTATAGTATTGATGATTCAGGAAAATTTCTGTTCTCAATAATTGATAAGCAAAATCCCGTCTGACATAAATCAGCAC
ACATGAGCCTGTCATTTGACAAATTTTTGTCATGAAGATGGGCGAATTTCCACACAGCACCGGCGCCCGGCAAGATGGGCGG
ATTCCCACACGACAGCGGCGCCCGGCAAGATGGGCGGATTTCCACACTACAGCGGCGCCCGGCAAGATGGGCGGATTTCCA
CACGGCAGCGGCGCCCGGCAAGGTGGGCGGATTTCCACACGGCAGCGGCGCCCGGCAAGGTGGGCGGATTCTCACGCGG
CAGCGGCGCCCGGCAAGATGGGCGGATTTCCACACGGCAGCGGCGCCCGGCAAGGTGGGCGGATTCTCACGCGGCAGCG
GCGCCCGGCAAGGTGGGCCGATTCCCACGCGGCAGCGGCGCCCGGCAAGGTGGGCCGATTCCCACGCGGCAGCGGCGCC
CGGCAAGGTGGGCCGATTCCCACGCGGCAGCGGCGCCCGGTAAGGTGGGCGGATTTCCACACGGCTGCCGCGCCCGGCAA
GGTGGGCGGATTTCCACACGGCAGCGGCGCCCGGCAAGGTGGGCGGATTCTCACGCGGCAGCGGCGCCCGGCAAGATGG
GCGGATTTCCACACGGCAGCGGCGCCCGGCAAGGTGGGCGGATTCTCACGCGGCAGCGGCGCCCGGCAAGATGGGCGGA
TTTCCACACGGCAGCGGCGCCCGGCAAGGTGGGCGGATTCCCACACGGCAGCCTCGCCCGGCAAGGTGGGCGGATTCCCA

CACGGCAGCCTCGCCCGGCAAGGTGGGCCGATTCCCACGCGGCAGCCTCGCCCGGCAAGGTGGGCGGATTCCCACACGGC
ACCGGCGTGCGGCAAGGTGGGCGGATTCCCACACGGCACCGGCGCGCGGCAAGGTGGGCCGATTCCCACACGGCACCGG
CGCCCGGCAAGGTGGGCCGATTCCCACACGGCAGCCGCGCCCGGCAAGGTGGGCCGATTCCCACGCGGCAGCCTCGCCCG
GCAAGGTGGGCCGATTCCCACGCGGCAGCCTCGCCCGGCAAGGTGGGCCGATTCCCACACGGCAGCGGCGCCCGGCAAGG
TGGGCGGATTTCCACACGGCAGCGGCGCGGGGCCAGTGGGATTCAGGAGAATAGGTGTTTTACCGAATGCCCTGACGAGG
CGTAAAAAAACCGCTTGGCGGCGGCCTCATAAAGCAGAAAACCCGCTCAAGGCGGGTTATCTGCTCTGTAGCCTGTGATGCT
TCGCGGGCATCCGGCATACAGCGAGGTGAAATTCTTCTTTTGGCATGTTAATTATACGTCTAACGCGGCATATGATCAAACCTG
TATTAAATAAGCCACTGTACCGTTTATAATGCTCTCAGATCAAAGAGGTAAAGCCCGTTTAGCCGCCTGTGTGATGAGCCAGTT
CAGACTCTTCAAAATCGAATTTGGTACTAAACAGGACCCGAACCGTGGGCAAGCACACGGCAACGGTATAGCCCTCTTCCGG
TTTCGCACCCGGAAGCCTGGGCGGCAGCGTGGTGAAATTCTTCTTTTGGTTAAGTGAATGGCATAACCGGATGGGCGGATTA
GAGGAAAGGGGATTGCCTAGTAACCTACGCGCCACAGAGATGGAGGTGCGGGGAATGATTGAGCTGATTATCGCTATTCTGA
CCTTAATTGCGGCTGTATTGCAGTTGATCAACTGGTTCCTTTAATGGTGCCGGAGTCTGTGAAGGTGAAAGCCTGAACGGGC
AAAACTGAAAGGTTTATAGCCGTCCTTCGGGGCGGCTTTTTTTCGGCAAAATTAGGGTTTTACCGAATAATGCAGAGTTTTAA
GGTGAGAATTTGCAGACTTGGCGTTTTACCGAACATAGATACTCCCTAGGCTGATAGGTGCATTAGTTATCACCCTACCTGAAC
ATATTGTAAAAGATGTCAGTCTCCAGTGACTTGTGTACTATCAACTGACAAGACTCTTACACGCAACGCAGGGGGATGGAGTT
TTATGCTTAGAAAAATAATCAGGGGTAGCGGATTCACTCAGTCAGAAGAAAAACTGATAGAGTTCGCTGATGATGCTTTTTTT
GGTCTTTGGTCTTATCCTAATGTTTATAGCGATGAGGGTTACTCTAAAAATAAAATTGGGAAAGAAGTTAGTGACTTATTAGTTA
TTTTTGATAAAGATATAATAATTTTTTCCGATAAAGCTATTACATACAATAAAAACAAAGATCCTAAGGTTGCATGGCAGAGATG
GTTTAAAAAATCAGTCATACAGTCTTGCACACAGTTATTTGGCGCAGAGAAGTTTATAAAAGATCATCCCGAAAGACTTTTTGT
TGACAAAGAATGCTCAGTTAACCTCCCCATTAAAATAGATAATTCTTTTAATTTTCATTTGGTGGCCGTCACTAATAATATTTCAG
ATCCGGCGATCTCGTACTTTGACAAAATAGAAAAAGGCAGCTCTGCTACTTTAGTTAACATATTTCCTTTAAACGCCCATCAATG
TCTAGAAAATCCATTTTGTGTCGGAGACGTTTATCCTGATAAGACTTTTGTCCATATACTTGATGAGACTGCCCTAAAACTACTG
TTAACCGAGTTAAACACAGCAACTGATTTCATTGGCTACCTTAACGAAAAAGAGAGGGTTGTAAGAGAAAGAACATTATTGG
TCAGCGCTGGGGAAGAAGAGACTCTTGCTGCTTACATTATGGGTGATAAAACCATAATATCAAAAGAAATTATTGGAAACGAT
CAAGGGATGACCATACCGGAAGGTGAATGGAAAAACTATAAAACCACTTTCAATTATCAATATCAGCTCTCAATGAAAAAGGG
TAGCGTTTTCTGGGATAACCTAATCCACAACTTCTCGACAAGTATATTGTCAGCTAACGTTGGTTTTTTTAGTGAAATTGAATTT
TCTACACATGAATTAGGTGTTAGAGAATTAGCCAAAGAAAGTAGGCAATCTAGATATTACCTTTCAAAGAACTTTAAAGAGAA
ATTAAAAACAACTCAGCCTCATCTAAGAACGTCAAGAATGGTCGAATCAATCGATGAGCCTGGAAAGTTTTACTTATTCCTTTT
TTTTCCTAACGATAGCAAGTTGAGTTACTCTGATTACAGAATTCAACGTATATCTTATATAAATGCTTATGCTGAGGTTGCCTTTA
ATAAATACAGACATATTAAAAAATTAATTACTATTGCAACAGAGCCGCAAAATACAGAAGGAAGATCTGAAGACCTAATATATA
GCATATCCCCAGAGAAATTTACCAAAGAGCAAAATGAAAAAGCCAAAAGATTATCAAGAGAATACAAAATACTAAGTGATTTT
TTACCTACTAAAACGACAAAGAGCGATAACTTTAAATCAGTTATATCAAAAGGTGAAAAAATAGGGCGGAATACACCTTGTCC
ATGTGGCTCCGGTGTTAAATTTAAAAAGTGCCATGGTGCGAATAATTAGCATTATTGTATGTATAACGGTAATGGCGCGGCAGA
GAAACCGGCGCGTTCTGCCCTAGTGTTGGCCTGCGGGTTCCCCCGCACCCGCTGTATGTAGTATCGGCAGCATCTGAGAAAA
CCACTACATGTAGTTATCAGCGCCACAACGGCGCGGGGACGAGTGCGGTTCGGAAAAATTGGGGTTTTACCGAATCCGGCA
AAAGATTGCTTCCTATAACGTCCGCTTCTGGCACACAGCAGCCGTTAAGATGTAAGGCCTTACGCCAACTAAATCTAATGGGA
CAGATTTAGTTGGTGATGGTCAAGTAATCTGCAAACGGTCACCAAGTAAAATGCAAATGGGTAGTCAAGTCCGATGCAATTAC
GCACCCGGCAAGGTGGGCCGATTCCCACACGACAGCAGCGCCCGGCAAGGTGGGCGGATTTCCACACGGCAGCGGCGCCC
GGCAAGGTGGGCCTATTCCCACACGGCAGCGGCGCCCGGCAAGGAGGGCCGATTCCCACACAGCACCGGCGCGCGGCAA
GGTGGGCCGATTCCCACACGACAGCAGCGCCCGGCAAGGTGGGCGGATTTCCACACAGCACCGGCGCCCGGCAAGGTGG
GCCGATTCCCACACGGCAGCGGCGCCCGGCAAGGTGGGCCGATTCCCACACGGCAGCGGCGCCCGGCAAGATGGGCGGAT
TTCCACACAGCACCGGCGCCCGGCAAGGTGGGCCGATTCCCACACGGCAGCGGCGCCCGGCAAGGTGGGCGGATTCCCAC
ACGGCAGCGGCGCCCGGCAAGATGGGCGGATTCCCACACGGCAGCGGCGCCCGGCAAGATGGGCGGATTTCCACACGGCA
GCGGCGCCCGGCAAGATGGGCGGATTCCCACACGGCAGCGGCGCCCGGCAAGATGGGCGGATTTCCACACGGCAGCGGC
GCCCGGCAAGGTGGGCCGATTCCCACACGACAGCGGCGCCCGGCAAGGTGGGCCGATTCCCACACGACAGCGGCGCCCGG
CAAGATGGGCGGATTCCCATATCGACATGTATGTAGCTTGTGTTATCCGTGGATTGTGCAGCTCAGCGGGTCGCTTGTCGTATG

GCGTAGTGTCCCCCGTAACCGGCCGCGTGCGGCCGCTAACGCGCAGTACGGCGCCGCGACCCGAAGGCGGGCCGCCGTTCC
CGCGCGCAGGCGCGCGGCGCCCACTGCGCACCCCCGTGGGGGACGTGCGGCAGCTGTGTGGCGGTGAGCGGGATTAGGG
CTTTGCAGGGAGGGGGCTGGGTCGGGCGATACGTTCAGCATTGCGGTTTCCGGCGATTTGCGGCCGGTGCCCGTTTAACTC
CGGCGTGGTCGCCTTCCATGCCCTGACGGCATAAGAAAATAAAACCGCCATGCTGCGGTCATTCATGATTTTGTGGTGTAGCG
ATAAATAGTCATGCGAGAAACGTTGAAGCGCTTAGCAACTGCACCAACTGTCATTTCAGGATCAGCAAGTAAGATTCTAATTT
GTTTAACATCTTCTTCAGAAAGTGACGGTTTTCTCCCTCCCACACGGCCCCTTGCGCGTGCAGCTGCAAGGCCTGAGCGCGTT
CTTTCAATATTGCGGTTGCGTTCAAAGCTAGAGAATATCGCCATCAGATGAGTATAGATTTCCCCTATAACTGGCGCATTTGTGT
CTATTCTGTCCTTGATGGCTATGAAAGTTATTCCGCGTTTCTTCAGGTTGTCGAGTAAAGTAATGACTTGACCCAATGAACCACC
GAGCCGATCTAGTGCCCAAACTACTAGGGTATCTCCCTCGCGCAATGCTTTCAGGCAGTTCTCCAGTTCCAGCGCACCTTTTTT
GTCGCGCTTTGGGCCGCTACGTGAGGTCTGATCCTGATAGATTTGCTCACATCCAGCTTTTGTTAGTTCGTCAACCTGGTGCGC
CACATCCTGAAGATGCGTAGATTTACGGGCACTGTTGCAAATAGTCGGTGGTGATAAACTTATCATCCCCTTTTGCTGATGGAG
CTGCACATGAACCCATTCAAAGGCCGGCATTTTCAGCGTGACATCATTCTGTGGGCCGTACGCTGGTACTGCAAATACGGCAT
CAGTTACCGTGAGCTGCAGGAGATGCTGGCTGAACGCGGAGTGAATGTCGATCACTCCACGATTTACCGCTGGGTTCAGCGT
TATGCGCCTGAAATGGAAAAACGGCTGCGCTGGTACTGGCGTAACCCTTCCGATCTTTGCCCGTGGCACATGGATGAAACCTA
CGTGAAGGTCAATGGCCGCTGGGCGTATCTGTACCGGGCCGTCGACAGCCGGGGCCGCACTGTCGATTTTTATCTCTCCTCCC
GTCGTAACAGCAAAGCTGCATACCGGTTTCTGGGTAAAATCCTCAACAACGTGAAGAAGTGGCAGATCCCGCGATTCATCAA
CACGGATAAAGCGCCCGCCTATGGTCGCGCGCTTGCTCTGCTCAAACGCGAAGGCCGGTGCCCGTCTGACGTTGAACACCGA
CAGATTAAGTACCGGAACAACGTGATTGAATGCGATCATGGCAAACTGAAACGGATAATCGGCGCCACGCTGGGATTTAAATC
CATGAAGACGGCTTACGCCACCATCAAAGGTATTGAGGTGATGCGTGCACTACGCAAAGGCCAGGCCTCAGCATTTTATTATG
GTGATCCCCTGGGCGAAATGCGCCTGGTAAGCAGAGTTTTTGAAATGTAAGGCCTTTGAATAAGACAAAAGGCTGCCTCATC
GCTAACTTTGCAACAGTGCCCCTTTTAAAAATATGCCTGTGGATATGTGCATATCATCGCTTACCAGCGCCGCTTCGCGGCTCG
GTCAACCCCTCAGAACCGCGAAAACAAGTTTCGCAGTTCTTCACGGTTCATGTTTTAATCCGAGCATGGCGCCAGCACCTTCG
CGGCACCATCGAGGCCCGCGCTGCTGCCGCACCGGGCCTCACCCTCAAATAAAATAAAGTTTATAAAAATCGTACTTGTATTG
ACCCCTGTTTATTCTGAGTATATCCTTTCTGTAACCAAAACTACGGAGGCGCCACAGATGGCTCAGAAAAACAGAATTTCAGA
AACAGAATGGAAACAGCTGCTGCCGCAAATGGCTTCCTTTGCACACATCACAACAGATATTGGCTATTCCGTGCTTGTCAAAG
GTGAGAAATCATCAGACGTAGCCATGCGAGTAGGCCGTTCCAAGCAAAACATTTCCAGCACCGTTAAGCGAATCTGGGATTT
GTACCAGAACACCACTCTTAAAGCCGAGAACGGCGAACCATTAAAATTAGTTCAGGTCTGGATACCAGCAAGCCTTGCTGAA
ACTGTTTTAAAAGAAGCCGCGAAGTACAGCATTAATAACATCACGACCTCGGAAATGGAAAAAAAATAAAGTCCGAAAGGAC
TTTATTTTAAGACTACTAACGCGGCTTTATTACAGTGCTCGCCACCACAGTAAACCACTATCCCCGGTTTACCCCAGAGATTGAT
ATGACACACCGGGCAGCTGTACTTTTCCCGGTTTGTTTTGTTCACCGGTGCTTCAGGCGGTGGGGTTACAAATCGCATGGCCT
CTATGATCTCGTCGTCGAGTTCAACCGGGTCGCGCCCTTCCTCCACCTCCTGTTCGTGTTCGCTATCATCTTCCTCGTCGTCAAT
ATAGCCTTTTCCTGTAGGGCTTAGCACAGCGCCAGGCTTAGGCTGGTAAGGCGGAAACCTGTCCATCCAGGAAAGCCGGAA
ATGGCCTGTCAGCAGTTCATCACAGGCCATATCGAAAGGGCCACCGGCAATAATATAATGGGTCATGCTCTGGCCCACTTTCCT
GCCTCCCGGTTCGCCGGTATCAGAAGGCATTAGTCCTACCCGTTCCATCCGGGCCGCCCACTGTTTGTTGTGATAGCCACGGC
GGCCAGGCTCGCCAAAATGGAATTGCCACTGATGAACCATCTCATGCACCAGTGTTGAAAGCGTGGCCTTTATGGTTCTGATC
GAGAAATACACCGGATTCATAGCGATCTCGTCTACCGTGTACCCACTCTCACGGCCGACGAAACGCTTAAAGGAACAATAGCC
ATACGTTCGCTTCTCACGCTGCAACGTTATCAGGCATGGCGGCAGCTCGTTGCTGAATAGCTTCTCATTGAAAAAATCGTAGGC
TCTCTGAAGTTCATCGTAGGTTTCGGGCGTAGGCAAATTCACTGCTAACCTCACATATATGTATTGTGCAATACACATAATTTAG
CTGATCTTTTATACAGAGGAAACGGCTTTTAGGGGTTGTTTGCGGGAGAGGGCGAAATCCTACGCTAAGGCTTTGGCCAACG
ATATTCTCCGGTAAGATTGATGTGTTCCCAGGGGATAGGAGAAGTCGCTTGATATCTAGTATGACGTCTGTCGCACCTGCTTGA
TCGCGGCCGCGATAGCTAGATCGCGTTGCTCCTCTTCTCCATCCGCGTTCCAAGCTGCGGAAAGGCACCCATAAGCGTACGCC
TGGTCGAGCAGGCGACGCGGATCGACGTCCAGCGCACGAGAGAATGCGTCCGCCATCTGTGCAATGCGTCTAGGATCGAGA
CAAAGGTTGTCTCTGTCAGCCGGATCGTAGAACATATTGGCGGCGCCAAAGCCCACTTCACCGACCAGACCGACGGGATCTA
TCACCAGCCAGCCGCGACTGGAGAACATGATGTTTTCATGATGCAGATCGCCATGTAGCCCACGCAGTTCCGAGGCATTGCTC
ATCATTTGATCGGCTATAATCGCCGCGTGGACGTAGTCAGTTTGACAACCTGCGTTTTGATCATCGCGCGCCCGCTGAAACAA
AGCTGCAAAGCGATCCCGGATCGGGAGAAGGGCAGAAGGCAGGGGTTCCTCAGATGCGGCATACAGCTTCGCCATTAGTTC

CGCTGCAATTTCGGTCGCCTGGTAGTCGCCGTGCTCGGCAACGATGTGAGAGAGCATTCGCTCCCCGGCATATTCGAGCAAC
ATCAGATTGTTCTCACGACCGAGCAACCGGACTGCTCCCCTCCCATTGCGCCATACCAGATAGTCGGCCCCGCGCAGTTCATC
AGCAATGTCTTCTATAGGTTTCAATCCCTTGACGATTGCAGGAGTCCCGTCTGGCAATGAAACTTTCCAAACGAGGCTGGAAA
AGGTGTCCGCAATGAGAACAGGTTGCGAAACGTGCCAATGAGCAGGAAAAACAGGCGGCATGAACATCAACCCCAAGTCA
GAGGGTCCAATCGCAGATAGAAGGCAAGGCGTTCGCGGTCGGGGGCTTCGATCCCCAATACATTGAATAGGACAGCGAAGG
CGCGCTCTGCTTCATCTGGCGCTGCCCAGTTCTCTTCGGCGTTAGCAATCATGAGTGCCAAATCGGCATAGCGATCTGCTGTTC
CGAGCCGCCCAAGGTCGATCAGACCCGTGCATTGAAGAGTTTTAGGGTCCACCATGAAGTTCGGCATGCAGGGATCACCATG
GCAAACAACCATATCGGTGCGCTCTTGGTCGAGCCGCACCGGTAGCTCTCGTTCGACACGAGCCAAAAGATCGAGCTGCGGC
GTACTCTTGTCCTCGTCCGGTAAGAAGTCGGGATTGACGGCATTGCGGGACACCACATCAACGGCGCGTCCGAACATTCGCG
ACAGCCTGCGCTCAAACGGACATTGATCAACCGATAGGCTGTGAACAGCGCCAAGTTGCTGCCCCATTGACGGCCACGCTTT
GAGCAAATCCGCTCCAGACAGATCAGCCGCCGGTACTCCCGGAATTGCCGTTATCACCAAGCATGCACCCTCCTGTTCCTCCT
GCCAGTTGATCACCTCGGGGCAAGCCACACCTCGACCTTTGAGCCAAATGAGGCGGTCACGCTCTCCAGCGAGCTCACCGC
GGCGGGAAGCAGGTGCGATTTTCGCGAAGGCATGCCCGTCACCACGTCGAAAAACAAAATCACCAGATTCTCCGCCTCTGA
CAGGCAACCAGTCAGAATGCGATTCACCAAAAAAAATATTAGTTCGATTCAATGGAGGTTCCTTCAGTTTTCTGATGAAGCGC
GGAGGTGGCTCAACCTGCGAAAAGAAACGAGTTGCTACGTAAGTCCGAGAACATGCTTTCCATGGTCTCTGAGCTCGCCTTG
ATGCCCGAGGCATAGACTGTACAAAAAAACAGTCATAACAAGCCATGAAAACCGCCACTGCGCCGTTACCACCGCTGCGTTC
GGTCAAGGTTCTGGACCAGTTGCGTGAGCGCATACGCTACTTGCATTACAGCTTACGAACCGAACAGGCTTATGTCCACTGG
GTTCGTGCCTTCATCCGTTTCCACGGTGTGCGTCACCCGGCAACCTTGGGCAGCAGCGAAGTCGAGGCATTTCTGTCCTGGC
TGGCGAACGAGCGCAAGGTTTCGGTCTCCACGCATCGTCAGGCATTGGCGGCCTTGCTGTTCTTCTACGGCAAGGTGCTGTG
CACGGATCTGCCCTGGCTTCAGGAGATCGGAAGACCTCGGCCGTCGCGGCGCTTGCCGGTGGTGCTGACCCCGGATGAAGT
GGTTCGCATCCTCGGTTTTCTGGAAGGCGAGCATCGTTTGTTCGCCCAGCTTCTGTATGGAACGGGCATGCGGATCAGTGAG
GGTTTGCAACTGCGGGTCAAGGATCTGGATTTCGATCACGGCACGATCATCGTGCGGGAGGGCAAGGGCTCCAAGGATCGG
GCCTTGATGTTACCCGAGAGCTTGGCACCCAGCCTGCGCGAGCAGCTGTCGCGTGCACGGGCATGGTGGCTGAAGGACCAG
GCCGAGGGCCGCAGCGGCGTTGCGCTTCCCGACGCCCTTGAGCGGAAGTATCCGCGCGCCGGGCATTCCTGGCCGTGGTTC
TGGGTTTTTGCGCAGCACACGCATTCGACCGATCCACGGAGCGGTGTCGTGCGTCGCCATCACATGTATGACCAGACCTTTCA
GCGCGCCTTCAAACGTGCCGTAGAACAAGCAGGCATCACGAAGCCCGCCACACCGCACACCCTCCGCCACTCGTTCGCGAC
GGCCTTGCTCCGCAGCGGTTACGACATTCGAACCGTGCAGGATCTGCTCGGCCATTCCGACGTCTCTACGACGATGATTTACA
CGCATGTGCTGAAAGTTGGCGGTGCCGGAGTGCGCTCACCGCTTGATGCGCTGCCGCCCCTCACTAGTGAGAGGTAGGGCA
GCGCAAGTCAATCCTGGCGGATTCACTACCCCTGCGCGAAGGCCATCGGTGCCGCATCGAACGGCCGGTTGCGGAAAGTCC
TCCCTGCGTCCGCTGATGGCCGGCAGCAGCCCGTCGTTGCCTGATGGATCCAACCCCTCCGCTGCTATAGTGCAGTCGGCTTC
TGACGTTCAGTGCAGCCGTCTTCTGAAAACGACAAACGATGTCAGCCAATAAGTTGTTGTAATAATCGACAAGTGTTTGTTGC
AACTCGCCGTCCTGTAAGACTGCATTATCAGTTTGATTCAGGGCGGCTATGGTGTCATTTATACACCATAAACCGATGACGGCT
GCACGTAAATCTGTGGATGCTCTTGATGGCGATTCAACGTTTGCTGTCCCACGCATGGCGACAGCATTGTTTAGCCCAGCAAT
AATCCCATTATAAGTTGGGGGAGGGGCTCCGACAACTGCAAAGCTATCTTTTCTGTATTGTTTTGGATGTCGTCCGCAAGAAT
GGTATTCTCGATCAGTTCTCGAGCCAATAATGATGTTTCGAAGCAGACGGCCAACACCGTCTGCTTCGTTTCTGGTTCTTGTTC
TTCCACTGCCAGCGCGTTACTGATTGTTACGCACGTAAGTAGCAAAGTGAATGCGCTCTTGAGTCTCATCAACGGTTGCCTCC
ACAGTCTCGCTGGCACTGGAATCAATGTGAAAATTGTTCTGGATCGGATCAACAGGAATAAATCGATCACAGTCGAACTCTCT
TGGAATTATGGTCATGTGCAGTTTCGTTGCTGATGAAAGAGCTTGAATGTACAACTTCTCCCCACCAATGACAGCAATGCGTTG
ATCGGTATTCTCATACAGGTTGATCACAGCACGAAGATCAGGAACGACAGTAGCGCCTATGACATCTTCTTGTTTTAACGTCGA
GGAGATCACGAACGATTCGCGTTCGGGGAGAATTCCTTTCTCCTTGATTCGCTCCTCGGCGCCATCCTTTTCTAACTGCATGTC
ACGCATGTCAGTATACGTGTGTTTCCCCATAACGCACACGCGGATCTCTTTAGAAATCCGCGTAAATCGCGCCATGTCTTCTTTG
CATTTCCATGGAATCTTGCCGCCTTTCCCGAATCCCAACTTAGAATCCACAGCGACTATTAGCTCAAGTTGTGGGTGACTCATT
AAAACTCCAAACCAGTCGATGCTGGTTGTTCCAGATATTGCTTACGGAAGGTGGCCAGATCAATCAGTTTGATATTTGCCTTTC
CGCTGTTGTTGAGATCGTGCACTTTCTTCAATTTGCCGGAAGATCCGGAAGTGGATGCCGCGATCACCATGGTGGTCTTCGAC
GAGACTGATGATTGCACTTCACCACCCTCAGCTTCAATCAGCTTCTCCAAGGCCGCGTCACGAAAACCGGTGATCACGATCTT
CTCACCGGTAAAACGATCCCCCGAAGTGACCTGCTGTTTGAACCCGATGATTGATTGAATCTTGTTGAATAGCTCGGCATGTTC

AGCCATACCTGCAAGCACTTTTGCGGCAGTTTTGTAGCTGAATTTGTCGACGCTAACGATCTGCTCTTCGGTGATGGCATTGA
AATCATCCAAGGTCTTAATATCCAAGCCTTCACACAGAGCTTTCATCTTGCGGCGCCCAACGCCGCGCCCAAAGTGGGGCATC
GACCCCAACCAAACCGACAGGGGAACACTACTGGGTATAGGAAGTATAAACCACCTTTTTGCTCCTCATCCGAAGTATCTTAC
CTGAAATTCCCTCACTCGTTTACCGCTCAAGCCCCAATTTTAACTGCCGGTCCAGCCTAAACCGCTCTAATAAGGTTCGATTTG
GCGGTAAAATCTCTAGCCTGATAGCTCGAGAGATACAAACTGCCCCACCGCCCCGTTTAAAAGTTGGCAGTGTTGAGCAGTG
TTGGATTTGGGGTTGTCAGTCAAAGAGACGACTCTGTGATGGATCGAACAGGCTGGGAGTCAGTGGCGGCGCTCGTTCTGG
TGGCAGCTCACGCTGCTTGGCGGCATTCGCCTTGGCTGTTTTCTGTTTCAGATGCTTGAGAATCTGCTCAATGACCTTCGGATC
TTCGATGCTGGCAATCACTTTGACGTGACCGCCGCAGTGTTCGCAGACTTCAATATCAATATTGAAGACTCGCTTGAGGCGTT
GCATCCAGGTCATGGCGCGGTGGCGCTCTGCAGGACTCTTGTCACGCCAGTTAGTATCGAGACCTTCCGATTTGTCGGGCTTC
TTGCCCCGCTTGGCGGGTGTTACTTGAACTCGGTGTTTGCTGTTCGGTGCAAAGACGCCGTGGAAGCGTGTGAGGTTGACTC
GCGGCTTAGGTACCAACGCAGCGAGTTTGGCGATGAAGTCCAGCGGCTCGAAGATCACATGGGTGGTGCCATTGCGGTACG
GAGTTTTGAGCTCGTAACGCACCTGCCCATTGGCGGTTAATGCCAGACGTTTTTCTGAAACCGCTGGCCGACTAATGTAGCGA
CACAAGCGCTCAAGCTTATCCCGCTGATGCGCTTCGGCCATCACACCGGCGTGTAGCGAGAAACCAGCATGGTTGGCTACTC
GACTGCTTGAGTCGGCTTTATCCTCACGCCCTGGCAAGGTTTGCAGGGTGAAGACTTTGCGCCCTTGCTGGGGGCCGACGG
CAATGCGATACGTAACCGAAGCACCATGTAATTGAGTCAGCGTATCGTCTTCGCCCTCTTCCAGTGTCAACCACGTATTCTCGG
CATCACGCTCCAAAATCCCACGCTTTTCCATGCAGCGAGCGATGCGATGGCTGAGGGTGTGAGCGAGCGTATTCAGCTCATCG
TAAGTGGGTGCCTTGACACGATGGAAGCGTTGCTTGCCATAGTCATCTTCGGCATAGACACCATCGAGAAACAGCATGTGGTA
GTGGACATTGAGATTTAGCGCGGAGCCAAAGCGTTGGATAAGAGTCACTGAGCCAGTTTGTGCAGAGGCTTTGGTGTAACC
GGCTTTTTTGATCAGATGAGTTGAGAGTGTACGATAGACGATACTCAAGACCTGGCCCATCAGCTGGGGATGGCGAGCCAGC
AAAAAGCGTAGCTGGAAAGGAAAGCTGAGCACCCACTGGCGAATGGGCTCCTTGGGGAAGACTTCGTCTATCAGCAGCGCC
GCACTCTCGGCCATCCGGCGGGCACCGCAGCTAGGGCAAAAGCCGCGTCGTTTACAGCTGAAGGCGACCAGACGCTCGTGA
TGACAATCCTCGCAGCGAACCCGCATGAAACCATACTCCAGACGGCCACATTGGAGGAGGTTGTTGAATTCTTGTTGGATGTA
GCGAGGCAGGTGTTGACCTTGGGCTTCGAGTGAGGCTTTGAAGGCTGGGTAGTGCTGCTCAACCAGCTGGTAGAGCAGCG
TCTGGTCGGGTTGGTGGCGTTCGTAACCGTTTGTTTGAGTGGGCGATTGACTCGCCGTGGCGTTCCTTGCCAGCGACATGGG
TATCCTCCGCTGATACTGTGGTTATGTACAGTATCAGCGGCTTGCGTTCAGACGTCCAGTCTGGCCCTAGACATCGCTAAATGCT
TAACCCGCAATAGCCCTCACGAGTTGTTATCAGCCACTACCGGTTGAGCGAGAAGGTTTTGGGTTCAGGGTGCTATTGCTCCA
CCAATCACAATACTGAAGCCCCAACTGTTATCAGTTGGGGCTTTTTCTTGTCTGTTTGCGGCGGTTGCGTTTTATCGGTAGTTG
TCGAGCTCTGCACCATCCCACATAAGAGCTTAACGGTGCGATCTTCAACGCCATCACACAAAACTTTCTTTTTCACGCACAGTC
AACTTATTGGATGTTTTATTAACAACCCAAAAGGAGATATTTAGCGGGCGGCCGGAAGGTGAATGCTAGGCATGATCTAACCC
TCGGTCTCTGGCGTCGCGACTGCGAAATTTCGCGAGGGTTTCCGAGAAGGTGATTGCGCTTCGCAGATCTCCAGGCGCGTG
GGTGCGGACGTAGTCAGCGCCATTGCCGATCGCGTGAAGTTCCGCCGCAAGGCTCGCTGGACCCAGATCCTTTACAGGAAG
GCCAACGGTGGCGCCCAAGAAGGATTTCCGCGACACCGAGACCAATAGCGGAAGCCCCAACGCCGACTTCAGCTTTTGAAG
GTTCGACAGCACGTGCAGCGATGTTTCCGGTGCGGGGCTCAAGAAAAATCCCATCCCCGGATCGAGGATGAGCCGGTCGGC
AGCGACCCCGCTCCGTCGCAAGGCGGAAACCCGCGCCTCGAAGAACCGCACAATCTCGTCGAGCGCGTCTTCGGGTCGAAG
GTGACCGGTGCGGGTGGCGATGCCATCCCGCTGCGCTGAGTGCATAACCACCAGCCTGCAGTCCGCCTCAGCAATATCGGGA
TAGAGCGCAGGGTCAGGAAATCCTTGGATATCGTTCAGGTAGCCCACGCCGCGCTTGAGCGCATAGCGCTGGGTTTCCGGTT
GGAAGCTGTCGATTGAAACACGGTGCATCTGATCGGACAGGGCGTCTAAGAGCGGCGCAATACGTCTGATCTCATCGGCCGG
CGATACAGGCCTCGCGTCCGGATGGCTGGCGGCCGGTCCGACATCCACGACGTCTGATCCGACTCGCAGCATTTCGATCGCC
GCGGTGACAGCGCCGGCGGGGTCTAGCCGCCGGCTCTCATCGAAGAAGGAGTCCTCGGTGAGATTCAGAATGCCGAACACC
GTCACCATGGCGTCGGCCTCCGCAGCGACTTCCACGATGGGGATCGGGCGAGCAAAAAGGCAGCAATTATGAGCCCCATAC
CTACAAAGCCCCACGCATCAAGCTTTTGCCCATGAAGCAACCAGGCAATGGCTGTAATTATGACGACGCCGAGTCCCGACCA
GACTGCATAAGCAACACCGACAGGGATGGATTTCAGAACCAGACGCAGCCGCAGGCGCCGGAAACTAAACCGATGTCTGTA
GGGTTACCGCTGGATCTGCGTGAATTCCAGCAGCAGCAAGAGAAAGACTTTCTACAAACCAGCTTACAGCAGGCAAAATTTA
ACCAGAAAAAAGCCGCTGAATTACTGGGTCTGACTTACCATCAGCTTCGCGCTTTGCTGAAAAAACACCAGATTTAACGCAC
ATTTGCAGATGTTATATTGGCGGATTTGACGCATAACCTCATCAGGGTTTACCATGACGCCATTACTGTATAAAAAAACAGGCA
CAAAATATGGCTCTGGCACTCGTTGGCGAAAAAATTAACAGAAACCGCTTCACCGGTGAGAAAATTGAAAATAGTACATTTTT

TAACTGTGATTTTTCAGGTGCCGACCTGAGCGGCACTGAATTTATCGGCTGTCAGTTCTATGATCGTGAAAGCCAGAAAGGGT
GCAATTTTAGTCGTGCGATGCTGAAAGATGCCATTTTTAAAAGCTGTGATTTATCCATGGCGGATTTTCGCAATGCCAGTGCGC
TTGGCATTGAAATTCGCCACTGTCGTGCGCAAGGCGCAGATTTTCGCGGCGCAAGTTTTATGAATATGATCACTACTCGCACCT
GGTTTTGCAGTGCATATATCACTAACACAAATCTAAGCTACGCCAATTTTTCGAAAGTCGTGTTGGAAAAGTGTGAGCTGTGG
GAAAACCGTTGGATGGGGGCCCAGGTACTGGGCGCGACGTTCAGTGGTTCAGATCTCTCCGGCGGCGAGTTTTCGACTTTC
GACTGGCGAGCAGCAAACTTCACACATTGCGATCTGACCAATTCGGAGTTGGGTGACTTAGATATTCGGCGCGTTGATTTACA
AGGCGTTAAGTTGGACAACTACCAGGCATCGTTGCTCATGGAACGTCTTGGCATCGCGATTATTGGCTAGTCTTCAGGGAGC
GGTGAATATTCCGCCCCCTGCACAGCTTTTTACCCTTCAGGCATCGCTGAAGAGTGGTGTGTGGAAATTTTCCACTCTTTACCG
TCCCATGCATAAGTGAATGTGTAGCGGGCTGAAACCGTTGATTTGTCCGCGAAGGTAAATGTGTAGGTACCGGTGTCTATCGC
CTTATTACACCCCAGGCGAATGGTACGCATGTCAATTTTCCCGGTCGGCTTTTTCGCAAGAAAATGCTCGAAATAAGGCACTG
TTGCAAATAGTCGGTGGTGATAAACTTATCATCCCCTTTTGCTGATGGAGCTGCACATGAACCCATTCAAAGGCCGGCATTTTC
AGCGTGACATCATTCTGTGGGCCGTACGCTGGTACTGCAAATACGGCATCAGTTACCGTGAGCTGCAGGAGATGCTGGCTGA
ACGCGGAGTGAATGTCGATCACTCCACGATTTACCGCTGGGTTCAGCGTTATGCGCCTGAAATGGAAAAACGGCTGCGCTGG
TACTGGCGTAACCCTTCCGATCTTTGCCCGTGGCACATGGATGAAACCTACGTGAAGGTCAATGGCCGCTGGGCGTATCTGTA
CCGGGCCGTCGACAGCCGGGGCCGCACTGTCGATTTTTATCTCTCCTCCCGTCGTAACAGCAAAGCTGCATACCGGTTTCTGG
GTAAAATCCTCAACAACGTGAAGAAGTGGCAGATCCCGCGATTCATCAACACGGATAAAGCGCCCGCCTATGGTCGCGCGCT
TGCTCTGCTCAAACGCGAAGGCCGGTGCCCGTCTGACGTTGAACACCGACAGATTAAGTACCGGAACAACGTGATTGAATGC
GATCATGGCAAACTGAAACGGATAATCGGCGCCACGCTGGGATTTAAATCCATGAAGACGGCTTACGCCACCATCAAAGGTAT
TGAGGTGATGCGTGCACTACGCAAAGGCCAGGCCTCAGCATTTTATTATGGTGATCCCCTGGGCGAAATGCGCCTGGTAAGC
AGAGTTTTTGAAATGTAAGGCCTTTGAATAAGACAAAAGGCTGCCTCATCGCTAACTTTGCAACAGTGCCGTACCGACGGTG
ATATGGGGCAAATGGTGGTCACCATCCTGTCGGCTGTGGCACAGGCTGAACGCCGGAGGATCCTAGAACGCACGAATGAGG
GCCGACAGGAAGCAAAGCTGAAAGGAATCAAATTTGGCCGCAGGCGTACCGTGGACAGGAACGTCGTGCTGACGCTTCATC
AGAAGGGCACTGGTGCAACGGAAATTGCTCATCAGCTCAGTATTGCCCGCTCCACGGTTTATAAAATTCTTGAAGACGAAAG
GGCCTCGTGATACGCCTATTTTTATAGGTTAATGTCATGATAATAATGGTTTCTTAGACGTCAGGTGGCACTTTTCGGGGAAATG
TGCGCGGAACCCCTATTTGTTTATTTTTCTAAATACATTCAAATATGTATCCGCTCATGAGACAATAACCCTGGTAAATGCTTCAA
TAATATTGAAAAAGGAAGAGTATGAGTATTCAACATTTCCGTGTCGCCCTTATTCCCTTTTTTGCGGCATTTTGCCTTCCTGTTT
TTGCTCACCCAGAAACGCTGGTGAAAGTAAAAGATGCTGAAGATCAGTTGGGTGCACGAGTGGGTTACATCGAACTGGATCT
CAACAGCGGTAAGATCCTTGAGAGTTTTCGCCCCGAAGAACGTTTTCCAATGATGAGCACTTTTAAAGTTCTGCTATGTGGTG
CGGTATTATCCCGTGTTGACGCCGGGCAAGAGCAACTCGGTCGCCGCATACACTATTCTCAGAATGACTTGGTTGAGTACTCA
CCAGTCACAGAAAAGCATCTTACGGATGGCATGACAGTAAGAGAATTATGCAGTGCTGCCATAACCATGAGTGATAACACTGC
TGCCAACTTACTTCTGACAACGATCGGAGGACCGAAGGAGCTAACCGCTTTTTTGCACAACATGGGGGATCATGTAACTCGC
CTTGATCGTTGGGAACCGGAGCTGAATGAAGCCATACCAAACGACGAGCGTGACACCACGATGCCTGCAGCAATGGCAACA
ACGTTGCGCAAACTATTAACTGGCGAACTACTTACTCTAGCTTCCCGGCAACAATTAATAGACTGGATGGAGGCGGATAAAGT
TGCAGGACCACTTCTGCGCTCGGCCCTTCCGGCTGGCTGGTTTATTGCTGATAAATCTGGAGCCGGTGAGCGTGGGTCTCGC
GGTATCATTGCAGCACTGGGGCCAGATGGTAAGCCCTCCCGTATCGTAGTTATCTACACGACGGGGAGTCAGGCAACTATGGA
TGAACGAAATAGACAGATCGCTGAGATAGGTGCCTCACTGATTAAGCATTGGTAACTGTCAGACCAAGTTTACTCATATATACT
TTAGATTGATTTAAAACTTCATTTTTAATTTAAAAGGATCTAGGTGAAGATCCTTTTTGATAATCTCATGACCAAAATCCCTTAAC
GTGAGTTTTCGTTCCACTGAGCGTCAGACCCCGTGTTGACACCGGCGTACCCTCGGTGCTATCTTCGCGCCCCAATAGTCGGG
GCTTGGCCAGGACTTCCTGAGGCCGTCCGTAACTCCACCTTCAAACAAGGAATATCGTTGATGTCACTGTATCGCCGTCTAGTT
CTGCTGTCTTGTCTCTCATGGCCGCTGGCTGGCTTTTCTGCCACCGCGCTGACCAACCTCGTCGCGGAACCATTCGCTAAACTC
GAACAGGACTTTGGCGGCTCCATCGGTGTGTACGCGATGGATACCGGCTCAGGCGCAACTGTAAGTTACCGCGCTGAGGAG
CGCTTCCCACTGTGCAGCTCATTCAAGGGCTTTCTTGCTGCCGCTGTGCTGGCTCGCAGCCAGCAGCAGGCCGGCTTGCTGG
ACACACCCATCCGTTACGGCAAAAATGCGCTGGTTCCGTGGTCACCCATCTCGGAAAAAATATCTGACAACAGGCATGACGGTG
GCGGAGCTGTCCGCGGCCGCCGTGCAATACAGTGATAACGCCGCCGCCAATTTGTTGCTGAAGGAGTTGGGCGGCCCGGCC
GGGCTGACGGCCTTCATGCGCTCTATCGGCGATACCACGTTCCGTCTGGACCGCTGGGAGCTGGAGCTGAACTCCGCCATCC
CAGGCGATGCGCGCGATACCTCATCGCCGCGCGCCGTGACGGAAAGCTTACAAAAACTGACACTGGGCTCTGCACTGGCTG

CGCCGCAGCGGCAGCAGTTTGTTGATTGGCTAAAGGGAAACACGACCGGCAACCACCGCATCCGCGCGGCGGTGCCGGCA
GACTGGGCAGTCGGAGACAAAACCGGAACCTGCGGAGTGTATGGCACGGCAAATGACTATGCCGTCGTCTGGCCCACTGGG
CGCGCACCTATTGTGTTGGCCGTCTACACCCGGGCGCCTAACAAGGATGACAAGCACAGCGAGGCCGTCATCGCCGCTGCGG
CTAGACTCGCGCTCGAGGGATTGGGCGTCAACGGGCAGTAAGGCTCTGAAAATCATCTATTGGCCCACCACCGCCGCCCTTG
CGGGCGGCATGGATTACCAACCACTGTCACATTTAGGCTAGGAGTCTGCGCGGCAGAGCCGTGTGACCGGTTTTCTGTAGAG
CACTGACGATGGCGGCGGCGCTCTCTGCAATTGGCAAGGCGTCGGCGCCAAGGATACCAATCTTGCGGCGCGCGGCGTGTT
ATGACGACTGGGGTGCATTTGAGCCGCCCCATTTAACCTTCGCCCTCACAGATACGCCATTCGCCTCAGATTTAGCGCCATGCA
GACGAGCTTCCACTCGGCTTGCACCTTGTCCAGGCCCCTCATGCTGAACTGACGCAATCCCATCACCGCCTTGATCCAACCATT
CGGAGCCTCCACGATCGACTTGCGCCGGCGGTAAGCTGCATCGCCTTGCTCCGTTTTCAATTTCGCCGCAATCGCCGCCGTAT
GCGGATGGGTCTTGGCATTGACCTTGGCATCTTCACGTCCCTCGCGGCCGAGGGCAACGATGACATCGCCGTGGTGATCGGC
GACCTTTGCCAGAACAGCCTCACTACGGAATCCCGCATCCGCCAGCGTCTGGGCCGGCATTTCTCCGGTGTTGGCCTGAACT
GCTGCCAGCATGCCCAGCAGCGCCTGACTGTCCGCGGCGCAGTTGGTCAACTCCGCCGCCACGATGATCTGGTGCTCGGCAT
CGACCGCTGTGTACCCGTTGTAGCTCTGCTCGGAGCCACCACCGGCGTGTTTCATGATCCGGCTGTCCGGATCGGTGAAGCTT
TCCTGATCACGGTCATCCGGCACACCAAACTCGCGTTTGTACGAGCCACCGCCCTTGTCCGAGCCATCCGGATGGCGAGGCC
GGCGGCCATCGTCTTCGCTGCGCCCCCGGGCCTGGTCCGCTTCACGCTGGCGCGCTTCCAGGGGTCGGTTCCGGCTGAGGG
CGAAATGACACCCTAAGCGTTAGCTCTGTGTCGTTGCACGATGTCAGCGACGGTATTCTTGCTGATACCGAGCTCGCGTGCGA
TCCAGCGATAGCTGCGTCCCTCGGCCCTCATCGCAACCACCTTAGGCAAAAGTCGGTCTGATTTTGGTCGCACTCCGGCCTGA
CGACCAAGCCTCTTACCACGTGCCTTCGCAACAGCAAGGCCTGACTTGACCCGCTCGCTGATGAGATCCCGCTCAAACTCCGC
AATGCCGGAAAGAAACGTCGCCAGCATTCGTCCATACGGCGACGAAAGATCGAACGCCATTCCATTCATGGCTATCACGGAA
ACCTTCCAGTTCTCCAGTTCACGTAGCGTATTGAGCAGATCGAGCGTCGAGCGCCCCCACCGGGAAAGCTCAGTGACCAGGA
TTGCATCAATTTGTCTGGACTGGGCAAGCGCCAGGACTTTCTTTCGCTCGGCCCGGTCGAGTTTAGTTCCTGAACCTGTTTCC
TTAAATATTCCCACCACGTCGTAGCCGGCACGGCCGGCGAAGGCTCGCAGATCAAATTCCTGGCGTTCACAAGACTGATCCGC
TGTTGAAACCCGGCAGTAAATGGCGGCACGATGTCCCAATTGAACCCTCCTGGATTTTTGTATCGGAACGCCCTGATTTATATG
GGCTGGCTGTTGTCCAAAACAGACTATACTTCAAAAGGGACGAATTTGTATGTCACGACGCCATATTTTCACCGAACGGCAGC
GAGCAGCGCTGTTCGATCTGCCCACGGACGAACTGTCGCTACTGAAGTTCTACACGCTGGGCGATGATGACCTGGAAAACAT
TAGGCAGCGCCGCAGACCGGAAAACAGGATTGGCTTTGCCCTGCAACTTTGTGCCTTACGATATCCGGGCCGTGCACTGGCT
CCTGGTGAGATGATCCCGCGTGAAGTCCTTTCCTTCGTCGGTGCTCAGCTTGGAGTTCCGGCTGATGCGCTTCTCACTTATGCC
ACACGGCGCCAAACCCGTCAGCAGCACATGGACACGCTGCGCGAAATTTACGGCTACAAGACCTTCACGGGCCGTGGTGCC
CGTGATCTGCGGGAGTGGACTTTCGGCCAGGCCGAAGATGCCAGATCAAACGAGGATCTTGCTCATCGTTTTATTGTGCGGT
GTCGGGAAACTTCCACCATTCTGCCCGCAGTATCGACAATCGAGCGCTTGTGCGCGGATGCTCTGGTCGCCGCTGAGCGGCG
GATTGAAACGCGGATTGTGGAAAATTTAACAGCGGATGTTCGCGATCACCTGGACAAACTTCTGAGTGAAATGCTCGCCGGC
AATATCAGTTGTTTCATCTGGCTTCGCAACTTCGAGGTTGGTAACAACTCGGCTGCTGCTAACCGTTTGCTCGACAGGCTCGA
ATTTCTGCGTACCCTGAATATCAATCATAGTGCTTTGGCCAGCATACCTGCCCATCGCATTGCCCGGCTGCGTCGGCAGGGTGA
ACGCTACTTCACCGACGGTTTGCGTGACATCACTTCGGACCGCCGCTGGGCGATCCTTGCCGTCTGTGTTGTGGAGTGGGAA
GCGGCGATTGCTGATGCCATAGTCGAAACCCATGACAGGATCGTAGGAAAAACCTGGCGGGAAGCGAAGCGCCAGCATGAC
GAAACAATTTCCGGCTCTAAAGCCACACTCACGGATACGATCCGTACCTTCACCGCGCTGGGAGCTTCGTTGCTTGAGGCCCG
CAGTGACGGAACCCCGCTGGAGATGGCTGTCGCCAGTTCGGTTGCATGGGACCGGCTCGCTCAACTGGTAGCGACAGGGAC
TCAACTCAGCAACACGCTAGCCGATGAGCCTCTTGCATATGTCGGGCAGGGATACCATCGCTTTCGTCGTTATGCGCCCCGCAT
GTTGCGCTGTCTGAAGCTCGAAGCCGCGCCGGTCGCCGGACCATTGGTAGCAGCAGCTTTGTCGATCGGAGAGATGAAAGG
TGTTGCATCGCCAGAAAGGCGTTTCCTGCGGCCCAGCTCCAAATGGAACCGTCATTTACGAGCTCAGGAAAAAGGAGATACC
CGTCTTTGGGAAGTGGCGGTACTCTTTCACCTCCGGGATGCTTTTCGTTCCGGAGATGTCTGGCTCGCTCATTCGCGCCGCTA
TGGTGACCTCAAGCAGGTACTGGTGCCGATGATCGCGGCGCAGGAAAATGCAAAACTGGCCGTGCCTTCCAACCCACAGGA
TTGGCTGGCAGACAGAAAGGCGCGACTCACGATCGCTCTTAAGCGGCTGGCCCGGGCTGCCCGTAACGGCACTATTCCGCA
CGGTAGCATAGAAGATGGAACGTTGCGGATCGACAGGTTGACAGCAGACGTGCCGGATGGTGCCGAGGCACTCATACTGGA
TCTGTATCGCCGAATGCCGTCCGTTCGGATTACCGACATGCTGCTTGAAGTTGATGCAGCCCTTGGTTTCACAGATGCGTTTAC
CCATCTGAGAACCGGGGCTCCATGTCGCGACCGGATCGGTCTGCTCAACGTCCTGCTCGCTGAAGGGCTCAATCTGGGCCTG

CGTAAGATGGCGGAAGCTACAAACACGCATGATTACTGGCAGCTCTCACGCCTTGCCCGCTGGCATGTTGAAAGCGAAGCCA
TGAACCAGGCATTGGCAATTGTGGTGGCCGCGCAGGGTAAACTGCCGATGTCACGCGTCTGGGGGATGGGCACGTCAGCAT
CGAGCGATGGTCAGTTTTTCCCGACAGCGCGGCATGGCGAAGCCATGAACATGGTCAATGCCAAATATGGTTCTGTTCCCGG
CCTCAAAGCGTATACTCACGTAAGCGACCAGTTCGCGCCATTCGCTTGTCAGTCGATCCCGGCGACCGTGAGCGAGGCACCG
TATATTCTCGATGGACTACTGATGAACGAGGTCGGTCGCCATGTTCGCGAACAGTATGCCGATACAGCAGGATTCACCGACCAT
TTGTTCGGAGCCAGTAGCCTGCTCGGCTACAATCTCGTTCTGCGAATCAGGGATCTGCCATCGAAGCGGTTGTACGTATTTAAT
CCCGATACGACCCCCAGGGAGTTACGCAAGTTGGTAGGTGGAAAAGCCCGGGAGGATCTTATCGTTGCGAACTGGCCTGATA
TTTTCCGTTGTGCCGCGACGATGACCGCTGGCAAAATCAGGCCCAGCCAACTCCTGCGCAAGCTCGCTTCTTACCCACGACA
AAACAACCTTGCAGTTGCGCTTCGTGAAGTTGGTCGTATTGAACGGACCCTTTTCATTATTGAGTGGATCCTGGATACGGACA
TGCAGCGGCGTGCTCAGATCGGTCTTAACAAGGGAGAGGCCCACCATGCGCTCAAAAATGCGCTCCGTATCGGGAGGCAGG
GGGAAATTCGCGATCGCACGACAGAGGGGCAGCACTACCGAATCGCTGGGCTCAATTTATTGACTGCGGTGATCATTTACTG
GAATACCGTCCATCTTGGTCATGCCGTCACGGAGCGGCGGAACGAAGGGTTGGATGTTCCCCCTGAATTTCTTCCCCACATAT
CCCCATTGGGCTGGGCGCACATTCTACTGACTGGCGAATATCTTTGGCCCAAGGAACCGAAAGCTTAGGGTGTCATTTCGCCC
TCAGCCGGAACCGACCCCTTCCAGGCGCGCCTTTGCCGCCTGGATCGCCTCCAGGCGCTTCTCGCGGCGAGAAATCTCGGCA
GGAATGTCCAGCTCCGGCTCGTTACGCTCCTGGTCGTCGGTAGCCTTGGCGCGATCAAGCAGCGCCTTGATCTCGCAATGCA
ATTCGTCCTCGGCCGGCTTCATGCGCTTATAGCTCATCGCCTTGTGGCGGCTGGCGTTGGCTTTTACCTTGGTGCCGTCGACCG
CGATCGTGCCAAGCTTCACCAGCCCACATTCGCGCGCCAGTTGCACCACCTGAACGAACAAATTCTCAAGCTCGGTCAGGTG
TAGGGCACGGAAGTCACTCAGCGTGCGGTGGGCCGGGAAGTTTCCAGCGGCCAGCACACGCAACGCGACATCCTCGTACA
GCTTCCTGGCTAGTTTGCGCGAAGAGAAGACGCCGCTCGCGTAACCATAGATCAGCACTTTGACCATCATCGCCGGATGAAA
CGGCTGATTGCCCGGACCACCGCCGGCATACCGGGCGTGGAATGCGCTCAAGTCCAGCGTATCGACAGTCTCGCTGATGAAG
TAGGCAAGATGCCCTTCAGGTAGCCACTCTCCCAGAGAAGGGGGCAGCAGATAGGATTGGTCGGGTCGGTAAGGAAGGTA
ACTGGCAGCCATCCCCGTATCGTCCTCGATCTTCCGCCGATTTGGCTTCTGCCGCGCAGGCTCCTAGGCCACCGTAGCTAAACA
TACGAGAAAAACCGCTTCTTGAGCGCTCTCTGAGACCACCTTGCCGACAACGCAGAAACGGTTGCGGAAAAATCGGCGTTT
CCGGCGTACGCCACCCGACCTGGACGAACTATTGCGGACCTGGAACTGGAGTAGCGGAATTCCCCGGGGTTTCAGCGTCGC
GCCACAGCGCCTCAGATAGATGCGGTAGCCTTTGGCGGGTCGTCATGCCGCGGACCACCCCCCAAAAAATTCCACAATGCGG
AATGGCGGAATTTTCTGGGGTTCCCGCTTACCCCCCCTTCAGGCGAGTAGCCAACCTGAACGGATGGTTCCACCGCAAAGGC
GGGCAGGGCGGCTGTCGTAAATGCTGCGGCCAGAAGCCAGGTTGCTAAGTTTTTCATTACAGGCTCCCGTTCACACACTGCA
TTTTATATTCAGGGTCAAAATAAATTTCTTTGATTTTCCGCTTTTCCATATGCACCACAACGTCAATGGTGGAGTAGAGCATTCG
CATAATATCGCTCATATCGAGCATACGGCCGATAGGGGTCGCCTTGATAAGCAGCCCAATACGGTTAAAGGCATCGCGCGCAG
AGTTAGCGTGCGTTGACATAACACCGCCTGGATGGCCGGTATTAAGTGCTTTAAGATAATCCCACGCAGCATCATCCCTAAGCT
CAGTCATGATGATACGGCCCGGTGTCAGACGCATACAGGCTCGCAGGGCATCAGTGGCGCTGACGCGGCCGATCTTTCCTGC
ATCGCCGTACATCATATAAACGGCTTCTACAACGTGATCGACCGTGACTTCGTGAACGTCCTCTAAAATAATTACACGCTCGTCT
TTATGTAGCGATTTTAACAGCGCGCGCGTGAGTACCGTTTTCCCCGACCCGGTTTCACCGCAGATCACGATAGTGCGTTTCTTC
TCAACGGCGGTTTGCAGGAATGCGGGCCATTTTTCGCTGCTGTGCAGCTCTTTAAGGAAAAAATCATCATCCGTTAGGCTTTG
CTTGCTGCCGGTAATCTTCCGGCAGTCACTGAAAATCCCCTCGCGGGTCAGCTGCTCCAGATTTTTATCGGCCGCCAAATCCTT
ACGAAACGCTACGGCCGTTGTACCGTCAATCACCGCAGGGGGCAGACAGATAACGCCCCTGATCCCGCCAGGCAGGATCAC
GTCATTAATGGCCTGCATGGTCAGCTTGTTGCTGCTCACCAACGATTTAGCAAGGTTCCTAATAAAATCTGCCGTAATTGCCGC
GTTCTGCACAACCCTGCGGCCGCTGAACGTATCACAGATAACTTCCTGAAAGCAGTTAATGCGAATTTCAAAAACAGTAGGAT
CTTCTAAATACTCGCGCAGTGGGCCAAGTTGATAGAAAGCTGCATCAGTCATGATTACTCCTGAAGAAAAGCGGGCGCTAAG
CGCCCACTTTTTTAGTTGTCTGCGAGCGTATAAACGCCGCTGAAATCGAGGTCGCGGGCAACAAAAATGCTCACCGCATCACC
CTGCTGATCGTAGAGGGTAGGGGGGATAGACATGTAAGAGCGGAGTGCTTCAGACGCCAGCTGCTCACCGCTGTTTTCTGTG
CTGTTGTACTGAATGTTATTACTCTGCGTCTGGTTAACCAGCGCCGTTAAGGTGTCAGAGAACAACGAAATCATGATCGCACCA
CGCAGACGCTCCCACATATGGGTATCCACCTGGCCCGGAATCCCCGCGCTGCCGAGTGAGTTCGTTCCGGCACTGTCAATATT
AACGATTGTCCCGTCCTGGTCATTGCGGATACGCTCCCAGAGAACAAACACGCGCGCCTGGCCGTCTTTGATACCACCGGTAA
TCTGCCCGTCAACCCATGAGCCTTTATCAATCAGCCTAACGAGTCCATCAGCTGAGTAAACGTCCTGTGAAACCCGGCAGGAA
ACCTGACCCGGAACAGTGGTATCCAGCTCGGTGCCGGTACCACAGGGGATCATTTTGCCTTTCGGAACAGTCAGGCTGGGAT

TAGCCATGACTCCAGCGCGGCTAGCCTTCAGCCTTGCAGGAGTCAGGTTTTTAGCGAGTGCTGAACTACCTTCGCTTGTTTCG
TTGTCCTGGGGCTGCACTCCGGGACTATTGCTTGTAGCCGCCTGATTAGTCTGGGCCAGCTCGCCGCCCAGACGACGCTGCAT
AGCCAGTTCTTCAGGCGAAGGTTCTTTACGCTTATTAGAGGTACGCGCGGCGGTATTGCTGCTGCCCGCATCTGCATCTGCCT
GTGCGGCAGCCTGCACAGCACGGGCATCAGTGGCGCTATTCTGCGCAGTTGCAGGTTTATTAACATCAGGATCGCTGTTAAA
GCTGTAGTTTGGCAGTGTATTGGCCTGTTGCGCTTTACCACCGTCTTTATCAGCTTCAGCTTTAGCCGGGGTGCGAATTTTACC
CATGACCGTAATCCCGATGAATACCAAAGCAAGCAGCGCCATCAGTATGACAAAGGCTTTCATACCAGGAGCCGAACGGCGG
TTACTGCCTTTAAATCCGCCACGCTCGCTTTCGAATTCACCGTCTCCGGTGTTTTCATCGAGTTCCTGATCTACATCGACACTTT
TACGGGCCATCAGTTATCCTCCCCAATTTGAACCCTGCGCACATCCGGGGAAGCCGTACCGGTTGCTACCGCACCGGCGCCCG
GCGCGAAATTATTATTACGAACGCCAACGACTTTATCGCCCAGACGAATACGCCACTCTTTAGCGACGGTTTCCACCTCGATGA
TGTTGCGGTTCTCACCCACAACATGAGAGTTAGGCAGCGTTTCTTTGCCACTGGCCGAAATCATGTAGACCTGCGGTAACTCC
GCATTGGCCGGAAACTCAAACCGGGTAAAGCGGTAGTTATCCCAGACGTGAACCGGCTGGATGCTGCGCATTTCAGGCTGTT
CGCTCATTACGTACTGATAGTTCTTCGCCCCCGCAAAAGCCGTCTGCTTCAGCTTCTGCGTAATGCGTTTTTTATCAGCCGCGCT
TTTGGCTTTTTCCTGCTGCTCAAACGGATATTCATAGGTCAGCTGAAGAACGGCCTGGCGCACAGCCCACGGCGTTTCAATAA
AGGATTTTGATACCGTACCGTCTGCATTTTTCTTCGTTTCTTCACCGATGAAATGGAGGACGATGTTATAGGTGCGCTTATCGGT
GACGATCACCAGGTTGGTATCACTCATGGCCTGTTTCGGCTTCACAAAAAAATGGTTCATTTTGTGCGCAAACGTCCAGCTTT
CAGAATCGCCAAAAGCATGAGTGATATAGGTTTCGTCAGGCGCGACAACAATGTGGGTAGCCACACCGGCGATAGCGTCAAT
TTTGACCACATTAACAGGGTTATAAACAACGCTTTTAATGCGATAGTCATAAGGAGAATTGCGGCCAACCTCAAGCGCCATAA
CGTTAGTGGCCGCGCCTCCCAGGACTGACAAAACAACTGCTGAAAGAAGTAGTTTTTTCATGGGGCAGCCCTCAGTTAACTT
CAGGGTTGACGCGATAACTCGTCACGCGGAAACCCAGCGGGTTGACATAACGCTGCTCAGCATTCATCGCCAGCGATTTATAT
TCATACCCCATAATGGCAATCCAGCGCTGCGGCTGATCATCAACGGGATTGCTGCGCACGCGGCGAACCGTAGTAAAGCGTAT
CGTTGCTACGCCGTGCGGTTTATCGAGGATCACAGAGTTAATCTTCACGCGGGTCGTTTCACTGTCGCCCAGAACCTTATCAA
GACCGTTGCGGCCCTTGAACTTGCTCTGGTAAGACTCTGCCACGTTCGGCGTGGACATTAAGCCAACGGCCGTATAGTCGAC
CTGAACTGAATAGAAGTCATAGCTCTCACGGTGAATGACATATTGTGTCAGCCAGAACTTATCAATTTCGTCACCATAAGAGGT
CTGGTCGCGGGTCAGCTTGACCTGCTGTACTTCGTGAGTGGCCTCGTTGAGCGTTAGCAGATGTGCGGGGATTGGCTGGCTG
TACTTATGCACCACGTAACCAACTAAAGAGAGTGCAAAAACAGTTACCACCGCTGAACCGGTGGCAACCATCCAGGCGGTAC
GCCGGGACTTCAGCACTTCATCCATCAGATCAACTTCAAGCCCTTTACGGCTTTCGTTGAACTCTTTAATGGCTTCACGTGTAA
GCCCTGTTTTTTTATTAGCTTTCATTTGCACCACCTTGCGTATCAACCGGGATTGTTTTGTTTACTGGAACGGTGTTGCTCCAGT
CCGGCTCCGGTGGCGGTTTATGCCCGCTGGAACAGGCGCTAATCAGAAGAACTCCCATAAGCAATAAGCTGCGCATTACGGC
ATCCATTATCGGTTGTTGAACGTACTAATTATAATAAGTCGTCGAAATTGCAATTTCAACGACTTTTGTTTTAATTTTATGCAGCC
TTCTTCCCGCGACTGCCCCTTGCCTTACCACTTAAATTAGAACCACTGCTTCCTCCGCTGTTGCTGCCACTTCCTCCACCTGCCG
ATTGCTGGCCGCCGCCACTGTTACCGCCTCTGAACGCATTACCGCCAAACATGCCGTGGCTGCCCATATTGCCAAGCGCCTGC
ATGGAAGAACCCGTAGAGCGTGCGGCGTCAGCAACTCCGGCGCTGATACCGCTACCCCAGCTGGCGGCAATTTGCGGAATCT
GGAACAGAACGAAAACGGAAATGACCGTCAGTAATAAGGCGGAGATAGAACCAGTGATTGATGAGTAAGCGGCATCAGAGT
TCATCGAGGACAGGAGGTTGTCGAACATCTGCATGATGAAACCAAACACGAGCGCAAGAATGACGACGACAAGGCCATAGT
TAATGACCGACGCCAGCCAGCGAGCAAAGATGTTTTTTGTCGCTCCCCACAGCAGGCAGAAGATTGCAATCGGGCCAAAAC
AAAGCGTAACGGCCAGAAGGATCTTAGCCATGATCACAAAGCCCGCACCGAGGCCGCCCAGCACAACGGTAGCAATCATCAT
AATGCCGCCAATGGCATACGCGGCCAGGCCGCTCGATGAAAACACGTCTGCGGCTTCCCATGCGGTGTTGACGATCTTGATAC
CTTTTTCAATACCGCTATCAATAATCGCCGGTACGCCACTTGCACCGACTTTATTAGGGGCAGACAGTATCCCGGCAAAATCAT
CCGGCAGGTGAAGCGCCACGTTGACCAGTTCCTGTTGATACCAGCCGCCCGCCGTTGCAAAGCTCAGGATAAGGGCTATGG
AAAGATACTCTTTAATCAGCGAACTCAGGCTGTCGCCCGCCCCCGGATTAAACGCTGAGTACATCCCCTGAACCATCAGCTTG
ATTGTCAGACAGGTGGCAATCAGAGGCGTTACATCAGAGATAATGGTGGCAACATTGGCGCTCACCATTGACGTAATCGCCCC
GTCTACTTTTGCGAAAATGTCTGCGACTAGGGTGAATGCCATATTGCCTCCTTACTCCTAAATTTTCGGTGAATTGATTTCGCCG
TCTTTTTTGCCGAACACAAACAGCTGACGATCGGCTTCGCGCGCGTTTTTGCAATCAGGTTTCTGAAGTTCATCCGCGTCTTTC
TTGCATTCCTGAATTGTTGCCTTGCGCTCTTTCTCATGTTTTTTGTACCACTCCACGTCATGCGAGGCATCGCAGGCCACTAGG
AGGAAAGGGATAACAAGCAGTAGTTTTTTCATAATTACCTCAGTTAATTGAAGGTGACGCGGTAACGTCCCCGCCGGTTCCAA
AAACAAAATTGTGGGTGGCACGTTCTTTCTGCGCACGTAATAGCTTGTCCTGTGACTGCTGCAACATGTTCATCAGATTCAGCT

TCGCCTGCTCACCCTGAATAGCACCCTGTGACGTCTGGATACGGGCCTGTAAGTCAGCAATCGATTTCAGGTCTGGAGTCGTT
TTAATCTGCTCCGTCAGCTCCTGCATATCGGTTAGTTCCTGCATCTGGTTGTTGTAGGCTTTTTCTGCCATAACACGGTCATAAG
CGCCTTTTTCAGCCAGCTTTTTGTTCATGTAGGTAATTGCTTCGCTGGGCGTCATGTCGTCAACTTCCGCATTAAACTGGCCCA
TCATGCTGTTAACTGAAGGCGTGACGGAAGAACTGGAGTTCATGGCGTCGCTGTAGATCTCCTTCCAGTTGTCCGGCAAATTA
TTTGCCAGCGTGCTGGTAGACGTCCCCAGCAGATCGCCCAGGTTGGTTGTCTTTGCCATCGACTCATACATATTTTTCTGCGTT
TGCAGCTGGCTTTTAAGCTGCTCCAGCTGCTGCGCCATTTGCTGAAGCTGCTCGACCTGTTTAGCCAGCTCAGTAGGGTTGGT
CACGATGATGCCTGCGGAAGCGCTTTGCGCGCCTCCCAGTATCAGGCCGGTGGTCAGCAATACTGCCGTCAGTGTTTTTTTCA
TGGTGTTTTGCCTCGTTGTTAAGCCGTCAGGCGCCAGTATTCTTTGAGCCATACTTCAGGGTCATTACCGAGCCGTTCAACCA
GCTCATGCGCAATTTCGGCGTTTTGTGGTTCACCGGACAACACGCTAAGAACGTTGTCCATTGTCTTAATATCTGCATCAATATC
GTCGCTGTTGCGAGGGTAGAGATTGAAAGAAGCAATCGCAGACTGTTGCCCTTGTTTAACCAGGAACTGTCTGGAGTGTTCG
GTAATCGACATCAGCGCGTCGTATTCAGCATCAGTAAGGAAGGCATAATCCTCTCGGATAGCTTCCGGATCGCGCAGGCAGAT
TTTTGTGACGGTCTGCGACATGATCGTGCGGCCAATACGGCTGGACAGCGCATCGTTCGGCTCCTGCGTGGCAAACACATAG
ATAGCGTCTTTCTTACGGTCAGTTTTGATACCACGCTTAACTTCACGCTCGATAACCGGATCGTCAAGGTAGGCGTGGAACTCG
TCAAAGCACTGAATGACGCGGCGTTTGCCGTCGATGGAGTCACGTACCCGGTACAGAAGGTACATCATGAGCGGAGTACGG
GCCGGGCTGGATACTTCCTCTTTGGCTGCGATAAACTCGGATAAATCGAAGCCAAAAATATCATTCGCGCTGAGATCCAGGCT
GTCTTTATCATTGTCAAACAGCCAGCCATACTGGCCTTCGCGCGTCCATTCGCGCAGCAGCCCTTTCAGTGATACGCCATTTTC
CACAACTTCATTCACGTACCCGTCCAGGATAGTAACGGTGCGCGCCTCGCGTGGAATAAGTGAGCCTTCCCCCATAACCGCAT
CAACGCCTTCAGCCAGTTCGGTAGCCATCGTTGCGCTGATAGGCCCGTTATTGGTGGTTTCCACACAAATGCGGAACAGGTTT
TTAATGAGGGCAATATTGCGTTTGGTCGGTTCAATCTGAAGCGGGGCAAACCCGGACGGCATACCCTGTTGCAGAACTTTATA
GTAGCCACCAACGCTTCGAATGAACGGCTCCATACCGCGGTCACGGTCATAAACAAAAAGCCGCGGGTTGTACTTCATTGACT
GCGCCAGCAGGAAGTTAAGCAGCGTGGTTTTACCTTCCCCCGACATACCCGTTATTAACGCATGGCCCAGCGGGCGTTTACC
GTAGGAAAGTTCTTCAAGCGGGGTCACATGGAAATTAAAATAGAGTGGCGTACCGCTGATCGTGCGGAACATGGTCAGCGCT
GGCCCCCACGGGTTATTGTCAGGCTTGCCACGCATAAAATTGTGGAACGGGCTGAAGTGCAGGAAGTTCCATGAGTTTATCG
GAACCGGGCGCGGCGCCCATTTCTGGTTGCCAGGCAGTCTCGCATAATATGCAGCCTCAGAGGCCAGGCTGAGAGTCCCGC
CAACCACGCCACAGCCGGTTAGCATAACCTTCACACGACGCGCTTTGCGCTGTACCGCGTTTTGGTCATTATCCCAGACATGC
ACGGTTCCATGATGGTAGCCCATCACGAACTCTCTGGACGTCAGCATATCGAGCGCGGTACCAAGCTGTGCCAGCTGGCTTTG
CGCACGGTCGCGCGTTTCCTGCAAAGATTTTTCCTGATGCGTCAGAAACGTTTTAGCTGAAGATTCAGAGAGGCAAGAAAAA
CTCTGCGTCAGAAGGTATTCAAAATCGGCTTCTTTAAGCATGTTAAGCTGGCCTGGCTCTGTATCTTCTTCGTATTCACGAAATT
CAATGCCGGTGGTATAGAAGTTGTGATCTACCGTTCTGATCTGGACAACATCCCCCCACAGTGAGCTAACAGGGCGGTTGTCC
ATGATGTACTCACGGATACGATCACGGCAAACAGGCACGATGGCCCATTCCATATTTGCGAGGAAATAAAGGAACTCCAGCGC
TTTTGAATAAGCGTGAGCCTGCGAAGGTTCAGGCTCGTTGCGTTCGATAACAATGGCTTCGTCAAAAATGTCTGATTCATCGA
CTTCAGCAAGTTCTTCACGTTCTTTTTTATCAGGCGCAGGAATTTCAACACCGCGTTTGTCACGATAATAGATACCCAACTGCT
GAATGCCATACGGCTTCATTGCTTCCAGGATTTGTTCAGAAATATCTTCCAGACCTTCAAGCGCCTCATTCTGCATTCGCTGAAT
TTCGTCACGAGTCGGCTTTTCAAATTTCGCCAGAAACTTCTGTGTTTTATCCCCTACCTGTTTGTAAATAACGGTCAGATAAAG
GTCATTAATCAGCTGCTTGGAATCACCGTGCAGCTTACGGTTATATTGATCAACATAAGCAGGGAAAAAATGGTCATACTCACC
ATCCGGGTACTCTTTAGCCTCATGGTGATATTCATGCGTCCACAGCTCTACATGGTCTGTTCCGAAGCTCTTGACCAGCGTATTA
AGGTCTTTATGCCAGGTGACCAGTTCCCGATCTGATGCGCAGTCATGTGTGCGGCCATCCAGCTTGAAAAAAGCCATCAGATC
GCCGTTTTCCATCGAAATCACGTAATCATTGAGGTGATAAGAATAGGGCAAATACTTTTTATTTACTGATGGCTCCTTACGGTAG
GCATCAATTTTTTTTGGCTTCGTAGCGGTGGCAGCTCTCATTATTTTAAACCCTTACGTTTGTAGTCAACAGAGGAATAAGACG
ATCCTCCCCACTGCTTAAACGGAGAATCAAACCAATTACTGAATTTGGTTTTCAACCACAGGCCCATAATTCGAAACATGCGGT
CGTCATATTTTGTTAATGCAGCTGAAGGAATCCACAAAAAGACGAACACCAGGATCGCCCACATATGAATAATCATAAAAAGC
GAGGCAGAAATCATGAATATCATCATAGCTACATTACGTGGTACACCCAGCGCGCGAGGTAAGCGAGTCGCACCTTTGAAAA
GCGGTCTTTTCCCGTCAACGAACATCTTTGTAGTCCTCACTTTGGGGGACAAAAGTCCCCCCCTTTTCGCGTTGTTCAGCTTCC
TACGCCCGTCAGGGAAACCAGAAATGATGCAGAACCAATACCAATCAGCGAGATTACGATACGAGGAATAAAGCTGGCTGGG
ATTACGTGAAGCATCCACATAAAGCAACTAACCATGATCGCAATAGCACAACCAATTGGAATCCATGTGCTCAACCACGTCTGG
ATTGATGTAGCGGTTGATTCACCAGTATCGGTGCCAGCGGCCAGCGCAATTTGCGGCAGGGCAATGGACAAAACGCCCATAA

CTACCGCAGGGCCATACTTCTTAAACAACGTGGTCATACTTTCTCCTTACTCACTATCTTCTGTTGATTGCAGATTTAACCGGCA
TTCTTCAACGCCACACTTATCTTTCAAAAACTCTGCCAGTAGCCTTAAATCAGCCCAGGTTCGTAGTTCATGCTCTTTGTTAACC
CGAATGGAACATGCGATCTGGCCCAACTGTTCATCGTCTATCTGAAAAACGGGATACCACGTCTTTAATGAAACCCGTTTTATC
GTTACCTCCTTCACTTTTCCTGTTTCAAATAGCTCCAGGATCTGTGATTGCAATACAAGGTACAAACGTTCCATCCATAACCTCA
CTCCCCCTTCGCTGTTTCCTGTTTGGTTAAAAAGGCATCCGTATTATTTCGGCTGAAGGCATCGCCATCACCCGAACCAAAAAC
ATCTTGTTCACCGTCATACTGCGTGGCCGTACTTTTTGCCTGCTGAGGCTCAGTCGCGGTGCTGTCCTCACTGGTCTGGCCATC
AGGTAGCAGCGTAGGGATTTTCAAATCAGTTGATTGACGCGCCACGTTGATAACTTTCGTGACATACCCGTTAGAAATCCCGT
TTATGAGTGAGCCGGTGTTGTAGCAGGAAAGCGCGTGTCTCAGCGCAACCTGCCCGGCTGGATAGGATTTCAGGGCGCTATC
ATAACAGGCTTTAAGGATGGTCTGGCTCGCCCGCAGGTTGATGCAGGGCTTGAAAATATCGTCAACCGAAAGACCCAGGCCC
ACTAAATTATTTGAGTTAATTTGTGCAAGGCCCATATCAAAACTTTTATTATCCTTCAGCAGAACTTTCGAAACGCTGACGGCCT
CAGCTTCAGTACGTGGTTGCTGTTTTAACTGGGTACTACCACCGTTAATATTGATCCTGTACGGCCCATTTGAGGACTCATGGC
CGACGATGTACGCCATTGTTAAGGGTGAAACATCAGGCGCACATCTCTGCGCAAGCCTGGCAACTTCATCTGAGGCAGGCGC
AGCACTGGCACGGCCAGCACAAGCAAGGCAGGCCAGAGCGAGAACCAGAAGTTTTGGATGTTTACTCATTTACGTTTCACC
GCGTAATTGTAATTTCGACGACTTATGTTTATGATAAGGGCTAATGAGATCGTGTCAAGAAGAAACGAAAAGAAAACCTCTGA
AGGAAGGAAGGTAATGCTGGTTCCCCTGAAATCAGAAAAACGCCCCAAAGGTGAACCCATGTATCGTGACCCGGATAACCCT
TTTAATACGTGGACTGGTATAGGGAAGCGCCCGGCCTGGCTAACTGCAAAATTGGACGCTGGCATTAGCCTGGAAGCCATGA
AAATGCAGGGCGTTGCCAACCCCAGAGAACATAGACAAGTAAAATACCGCGACCCCAGGAACGCAGAAAATACCTGGTCCG
GGACTGGCCGCCGACCAACATGGCTCAAAGAGCTGCTTGATAGTGGTTTATCACTTGATGATCTAAAGATATAACCGGAGAGT
ATAAAAATGGGCGATGTTATTGATTTTGCGGAAAAGCAAAAAGGCAGAAGGAAGAAAAAGGCATTTTCCATTCCGCCCATTT
TAAGAAAATTCCGAGTCCATGCTATCAGGTTACTCGCCAGCATCATCAAATCCGGCTCTTATTCAGTTGCTTATATCGTTAAGAA
AATTACAGGAAAGTTAATTAAGTTGTTCGCAATGTTAACGATTTTCGTTTTTATTGTCGAATATATCGCAGGCGATATAGGTTAC
AAATCTATTTATAACTCTGCGTTATTATTAATATTACTTACCGTTATCAACATTCTGGCGGGTGTATATCTGAACAAACTGTTAAGG
ACAAAACAATGAAGAAACTCTTAATCCCTCTGATAGCAGCTGGTAGTCTGCTTTATCTTCCTGCCAGCCATGCTGAAGATCCCT
GCAAAGTTATTATGTGCATGGCGGGCAAGCTCACCGGCGATAGCGGCGGAAGCGAGTGTAACAGTGCTGAAGCTGCTTTCTT
CAATATCGTTAAAAAGAACAAGCACGGCTTTTTACCCAACCACACGAGGGATGCCAGGAAGGCTTTTCTTAATGAATGCCCG
GATAATGGCGAAGGTGGAAGTAACCAGTCGATGATAAGCCAGATCATAAGTAAATACGGGAAAGTTCGCTTATAGGCGGGCT
AGGAATAATCTCAATTTAAGGAGCCAACGTGAAAAAAATTATATTAAGTGCATTAGCCTGTACAGCTCTCTTAACTGGTTGCGT
CAGCCAAGATAAAGGTAATGCGATGCAGAGCCAGATGAATAACCAGCAACGCCAGATTAACGAATTATCCGTTCGTTTGCAGT
CTGCGGAGTCCCGGCTATCAAAGCAGGAAGAAAAGCTGCGCAACGAACTGCTGCAATCCAGCGGCTATTGCTATCTGAATGG
CGCCCGCTACTCGACCGGCACCGTACTTTACGGGCGGATTTGCCAAAATCAGTCAGGCAGCGCTTCGTGGCAGGTTTACAGC
CGTCGCTAAACACCAGGGCGGTTTAACCGCCCTTTTCTTTACCCACAGAAAGCACACTATCACCATGTTCCCCTTCCCCACTAC
AGAAAACCTTATTCTATGGGCTTGCAGCGCCATCGCACTGTTTGCCGTTGTATTCTTCCGGCGTTCAGTACGCAACCGACGAC
ACAAAAGGAAGCAGCAAAGTGCGCGGCGGGTGCTGGAGCGCATAAAGACGTTGCCGGGCTTCCCACAAAAAATTAACTAC
CTGAGGAAAATTGATCCTTTTGTGTTTGAAGAACTGTTGCTGGAAGGATTTGAAGCGCATGGCGGCTTTGTTGAATAAATCA
GATTTCGGGTAAGTCTCCCCCGTAGCGGGTTGTGTTTTCAGGCAATACGCACGCTTTCAGGCATACCTGCTTTCGTCATTTTGT
TCAGCGCTCGTACCAGGGCCATAGCCTCTGCAACCTGACCATCGTAGTCACGCAGTGTCAGTGAACCTCCGAACAGCTGTTTT
ACCCGGTACATCGCCGTTTCCGCTATCGAGCGACGGTTATAATCTGTTGTCCATTTCCACCGCGCATTACTCCCGGTCAGCCGC
TGATTCGCAACAGCACGGTTACGGTCTGCATATTCACCGGGCCAGTAACCCGCGCCTTTTCGGGGCGGGATAAGCGCGCTGA
TTTTCTTACGCCGCAGTTCATCGTGACAGAGCCGGGTGTCGTAAGCGCCGTCTGCCGATGCTGCCCTGATTTTTCTGTGAGTC
TGCCGGATAAGACCCGGGAAGGCTTCTGAGTCCGTCACATTGTTCAGCGACAGGTCTGCACAGATGATTTCATGTGTGTTGCT
GTCAACGGCCAGATGCAACTTTCGCCATATACGACGGCGTTCTTTGCCGTGTTTTTTGACTTTCCATTCGCCTTCACCAAAGAC
CTTCAGCCCGGTGGAATCAATCACCAGATGCGCGATTTCACCCCGGGTGAACGTTTTGAAACTGACATTAACCGACTTTGCGC
GCTTGCTGACACTGGTGTAATCCGGGCAGCGCAACGGAACATTCATCAGTGTAAAAATGGAATCAATAAAACCCTGTGCAGC
CCGCAGGGTCAGCCTGAACACGCGTTTAATGACCAGAACGGTGGTGATGGCGAGATCAGAATAGCGCTGAGGTCTTCCCCG
TGATGAAGGCGTTGCCGACTCATACCAGGCCTGAATAGCTTCATCATCCAGCCAGAAAGTTATGGAGCCACGGTTGATGAGG
GCTTTATTGTAGGTGGGCCAGTTGGTGATTTTGAACTTTTGCTTTGCCACGGAACGGTCTGCGTTGTCGGGAAGATGCGTGA

TCTGATCCTTCAACTCAGCAAAAGTTCGATTTATTCAACAAAGCCGCGCATGGCTTCAGAACCATCAGAAACAAACGCTATACC
GGCGATGGAGGCATTGACGGCCAGGTAATAATAGGAAAATATCGCTATCTTATTCAGGCTAAACGCTATCGCGGCCATATTGCT
TTACAGCACGTACAGGAGTTCGAGAAGTTGCTTAAACGTCATAACTGTCGCGGTCTGTTTTGCCATACCGGGAAAACCGGCG
CAGGTTCAAAATCTGTCAGTATTGCCAGTGAACGGATGGAGATTATCAGCGGCCAGCGCCTGATAGATTTGCTCACGCCCGGC
AGCTCCTTCACTATCGCAACCGCCCCGCAGACGATGATGAAGCGTACCGCAGCAACACTAGAAACGAGCACCATTGTTAAAG
ATGCCGGTAAAGAAAATCGATACCATGAGAGTTAATTAAATGAAGTCAGTAACTATAGAAGCAAAAACATTTGCTGAAATGTTA
GGAATAACAGAAGGTGAATTAATCTTTGCCATTAAGAAAACTGGCACATTCAAAAACAAGACCATCCCACAACCTCATGAGCC
ACATAAATCAAATAATAGATTTTTATATTCAGACGTAATGAGGTTTATAGAATCACTAAAAGACAAAGAGAACCGGTAATGACTC
CAACTTACTGATAGTGTTTTATGTTCAGATAATGCCCGATGACCTTGTCATGCAGCTCCACCGATTTTGAGAACGACAGTGACT
TCCGTCCCAGCCTTGCCAGATGTTGTCTCAGATTCAGGTTATGTCGCTCAATGCGCTGAGTGTAACGCTTGCTGATAACGTGCA
GCTTTCCCTTCAGGCGGGATTCATACAGCGGCCAGCCATCCGTCATCCATACCACGACCTCAAAGGCCGACAGCAGGCCCAG
AAGACGCTCCAGCGTGGCCAACGTGCGTTACCTAACAATAAACCTGTTTAAATATCCAGATAAAAACATTCAATCTGGGTCAAA
TGAGTGATACAGTTTCACCCATAAGACCCAATGGAGGCAATATGTCTGAATTTGAATTACTGGCGCAGGATCTGCTTGAGAAA
GCAGAAGCGGAAGAACAACTGCGACAGGAAAATTATAAAAAGCTGCTCGGGCAGGTGCTGGAAATCTATGACCAGAAGTAC
GTGGCTGAACTGCTTAGAAAAGTTGGTAAAAATGAGTGGAGTCGCGAGACTCTTAATCGCTGGATTAATGGTAAGTGCTCAC
CTAAGACGCTGACGTTAGCCGAAGAGGAACTTCTACGAAAAATGCTTCCGGAAGCGCCTGCACATCACCCTGACTATGCCTTC
CGGTTTATTGACCTGTTTGCTGGGATTGGAGGTATACGGAAGGGCTTCGAAACCATCGGTGGCCAGTGCGTTTTTACCAGTG
AATGGAATAAAGAGGCTGTGCGCACATATAAAGCTAACTGGTTTAACGATGCTCAGGAACACACTTTCAATCTCGATATTCGG
GAAGTCACGCTCAGTGATAAACCTGAAGTACCTGAAAACGATGCCTATGCTTACATTAATGAGCATGTGCCGGATCATGATGTA
CTTCTAGCAGGTTTCCCCTGTCAACCGTTCAGCCTTGCGGGCGTAAGCAAGAAAAACTCGCTCGGGCGCGCGCATGGTTTCG
AATGTGAGGCTCAGGGAACGCTTTTCTTCGATGTGGCGCGTATTATACGCGCAAAAAAACCTGCCATCTTTGTTCTTGAAAAC
GTTAAAAACCTGAAGAGCCATGACAAGGGTAAAACCTTTAAAGTCATCATGGATACCCTCGACGAACTGGGCTATGAAGTTG
CGGATGCAGCTGAGATGGGCAAAAACGATCCTAAAGTTATCGACGGAAAGCACTTTTTACCTCAGCACCGAGAACGTATCGT
TTTGGTCGGTTTCCGTCGTGATCTGAACATTCACCAGGGCTTTACCCTGCGCGATATTAGTCGTTTTTATCCGGAACAGCGTCC
GTCATTTGGCGAACTGCTGGAACCCGTGGTTGACAGCAAATATATACTGACGCCGAAACTCTGGGAGTATCTCTATAACTACGC
CAAAAAGCACGCAGCTAAGGGTAACGGATTCGGTTTTGGCCTCGTTAATCCTGAAAATAAAGAAAGCATTGCCCGTACGCTT
TCTGCTCGCTATCACAAAGACGGGTCTGAAATTCTGATAGACCGTGGCTGGGATATGGCCACAGGTGAAACAGACTTCGCGA
ACGAAGAAAATCAGGCGCATCGGCCCCGCAGGCTGACTCCACGAGAGTGCGCGCGCCTTATGGGTTTTGAAAAAGTAGATG
GCAGGCCTTTTCGCATTCCTGTGTCAGACACTCAGTCGTACAGGCAGTTCGGTAACTCCGTAGTGGTGCCCGTGTTTGAAGCC
GTAGCCAAACTGCTTGAACCTTATATCCTGAAAGCGGTTAATGCCGATTCGTGCAAGGTTGAACGAATCTGATCGCTCCTCCCG
GTATTTATGCCGGGAGATAATCTATGGAATATCTGCGTAAAGCCCTGTCAGCTCAGCAATAAACGCACCTAGCGTCATTAGCTC
AGCTCTCACCGCCTCCGGGTATTTTTTGTGCAGCGATGATGGCACAACCAATCTGACACCCGACTCCCGCATCTCCCGATATTG
AGCCAGAGAAACTCCCTCTTGGAGTGTAAACAGATGCACCTGATGAATTTTATCGGCCTCATTCAGTATCTGACGCCAGCGAT
CCTTACAGGTAGTCTTGACTGCCAGCATGCGCAGATTTTCTACGGGAAACTCAGTATCGTGGTAAGCCCCTGCGGAAGGGAA
AAGGAAATCGGGTTTTTTATTACCTTCTGTGATGGCCTGCGTCGCAAAGTGTCGCAGGCCGTGCTCAATGAATAGATGCTCCA
GGTGCAGTTCCAGAGACTTCCCGGCTCTGGATTTACGGCGATTGCTGACAGAATTGGCCAGCGCAATAAATTCATCCACAGA
GCCAAATCCTTTCCGGATGATATCCAGAACATGCAGTTCCTCAACCAATAGAAATATGTCGTACTCCACGCGCCGGCGGTCAAG
AAGTTGCTCATCCGGATCAAGGGAATTTTTCACATAATGGCTGGCTGCATACTGAATAATTTCACTTCCCGACGGAAAGCGCA
GGTGCCAGTCTTCAGGTAGAATATATTTATGATTTACTGGCGCTTGCTGTAGAGATAGTCCGCCTAGAATCTGTCCTGCGGGGC
CGGATATAAGCGCTCCGGGTATAACTTCACCAATAGCGGTCTCAATGACGTCCTCTTCATCAGTGCTGGCGCATACCCAAATATT
TACTTCCTTACAGTCCCCCCCTTGCTCATCAAGCTTGAAAGCCAGGAGCGTCAGAGCCCCTGTATTTTCAGGATTCTGAAGTG
GGCTGCCTCTACCCCAGCGGGTAATCCTTTTTTCATTCCGGGTTTTACCAAAATGACGGCTGTTATAATAAATTGCCCGGGCTT
CGCTGTCAGGGCAATCATGCGATGACACATGTGCGGTGAGAAAAACCGAAGGGTTCAGTTCACGGGTATGGTTGATAGACG
GAAAGAGTTTTTCAACGATACCTGAAGGGATATAAAGCCCTACCTGGTGACCACCTGTTGCGCCGGTATCGTTGGCGGAAAG
GCGTTTGATGTAGACGAAGTAATTCTCACATGCGATCTCAAGTAGCCAGTTGTGGAAAACCGACATAAGCATCCCCTGTTACCC
TGAAACTCTACTCACCATTTTTTCATGATTATATACAAACAGCCAGAAAGGCTGTTACAGACGATTTGATCTGCAACCTATTGGT

TAAATTAATGTATCAAAAACGATGGTTTTTGTGACAGTCTTGAAAAGTCCTGACTTCTCCCGAAAAATGACTCCCCTCATGTAA
CAAAACTCGTTACTGTATCAACATAACAATAACCCCATAACTAATTAGCGAGAAAAGAATGAAAATCGGCTATGCACGTAAATC
GGCACTGTTGCAAAGTTAGCGATGAGGCAGCCTTTTGTCTTATTCAAAGGCCTTACATTTCAAAAACTCTGCTTACCAGGCGC
ATTTCGCCCAGGGGATCACCATAATAAAATGCTGAGGCCTGGCCTTTGCGTAGTGCACGCATCACCTCAATACCTTTGATGGTG
GCGTAAGCCGTCTTCATGGATTTAAATCCCAGCGTGGCGCCGATTATCCGTTTCAGTTTGCCATGATCGCATTCAATCACGTTGT
TCCGGTACTTAATCTGTCGGTGTTCAACGTCAGACGGGCACCGGCCTTCGCGTTTGAGCAGAGCAAGCGCGCGACCATAGGC
GGGCGCTTTATCCGTGTTGATGAATCGCGGGATCTGCCACTTCTTCACGTTGTTGAGGATTTTACCCAGAAACCGGTATGCAG
CTTTGCTGTTACGACGGGAGGAGAGATAAAAATCGACAGTGCGGCCCCGGCTGTCGACGGCCCGGTACAGATACGCCCAGC
GGCCATTGACCTTCACGTAGGTTTCATCCATGTGCCACGGGCAAAGATCGGAAGGGTTACGCCAGTACCAGCGCAGCCGTTT
TTCCATTTCAGGCGCATAACGCTGAACCCAGCGGTAAATCGTGGAGTGATCGACATTCACTCCGCGTTCAGCCAGCATCTCCT
GCAGCTCACGGTAACTGATGCCGTATTTGCAGTACCAGCGTACGGCCCACAGAATGATGTCACGCTGAAAATGCCGGCCTTTG
AATGGGTTCATGTGCAGCTCCATCAGCAAAAGGGGATGATAAGTTTATCACCACCGACTATTTGCAACAGTGCCGGTCGCCGG
GAGTCAGCAGATCGACGTCAACGCCGAGCAGCGATTTCAGTTCTTCTTCCAAATCGCCCAAGTCCAACAACGTGGCACCGGG
CAGCGCATCGACCAACAGGTCGAGGTCGCTGCCATCCCGGTCGGTGCCATGCAGCACCGAGCCGAAGACGCGCGGGTTCGC
GGCGCGAAAGCGGCCTACCGCTTCACGCACTGCGCTTCGCTTCATGTCAAGCACAACAGACGGTCGCATGCGCATCCTTTCT
TATCGAAACTCGTTGAGATGATATGCAATCAAGAATAGAATTTCAAGAACTCACAAAGTAACGCGGTGGTTAATATCCTGTACC
CACGGATTGCCCTTAGCGCTGCCTATATCGGCTAAAGCACTCCGGTAGCTTGATTCACCCCACGGCCACGGCAGGATCTTGGC
CGTCGCAAGCGCCAGGGGAAAATCTTCAGCTGCAAGCCTGAGTGATTTCATGTGCGTGTAATCCATCGCCCAGATGATTTTTG
TGAAGAAGAACTCGCGCTCGTTCATGTCCGGGCGCGCGTCCTGGCCCTCCGTGCCAACGGCCAGCAAGTAATCGGCCTGAAT
TGGCAGTATCAGCGCGCGTAGTAAGTCATGGATCGCCGGTTGCGGCAGTCTGCGCGCCAGATTAATTACCCGGTCGAACTTCA
GTTTATCCTGCTTGCGCTTGTCGGTCTGCTCCATCAGATTTCATGGCCCCCTTCTTCATGCTCATGCTCATGGGTGTGTTCTTTTC
CGGTATGGCTCTGTTCCGCCTGAGACGTCTGCGGCATGGCGTAATCGTCGTAAATGCTGCTGTCAAAGTCGTAGTCTGATGCTT
CGGCATAATGCTCGTAATCGGCATACTCCTGCGCGCTCCACTGCTGATCGTCGGCAGCAGCATAATCATGGGCCAGCTCTGCAT
CATTTTGCTGTGCTTCATGACGCCGCAGGCCAACGGAATCATCCATAGGGTTCTGCTTAAGATGAAAGGCGTCCTCTGCGTTG
CTCACCGGCTGATAATCAGTGCCGGTTGTCATGTTATGTTCATCGGGTTTCTGGTTAAACGCCATGCTTTCCCCCGTGGCTTCT
GGCAGACCTTTTTCAGCTGATCGGGTTTCTAAACTGGTATCGCGGCCAATATCCTTAAACCTGGCCTCAAGCCCAAAGAAACG
GTCAATTTCTGCGGCCGTGGTTTTCGGGCTGTCGCGGCTCACGCTCGATGCCAAAGATTTTTTATCGTCGGTAAAAATTTCCAC
CTCATGACGCGCACGCGAAATACCAACATAAAAAACGTCCTTAGAAGTGGTAAGCGATTTGGTATCTATGTTGAACAACACGC
GATCACAGGTAAGCCCTTGGGATTTGTGGACGGTGGTTGCATAAGCATAGGAAAGATAAGAAGCCTGTTTTTTGTCCAGCTC
AACCGTGCGCCCTTTTTTGTCCTCAAGCGTCAGTTTTTCACCCTCCACGGTTTTCACCGTGAAGCGGTCGCCGTTGGCAACGT
CCAGCGTTTTATCGTTACGCGTTACCATAACCTTATCGCCCGGCGCCAGTTCGGCGCTGACTGCCTGGTATACAGACAGCTTGG
TGTGTGTACGCGGGCTGAAAGCGATCTGCTCACCGCTGCTGCTTTCAACCGTCAATTTGTTGCCCGGCCCGGTATCAAGAACC
TGGTAAGACTCGCCCCGCTTCATACCATTTTTGTAATCCTGTTCGGGGATAATGATTTGCCCTTTACTGAAATAACGGCTGTCGC
GGCGTTCCGCCTGTGTCGAATCCACGCGGTCAAGTAGCGTGAACGTTTCGCCGGTTCCGGCAAGCCCCAGATTGCCCCGGAT
GTAGTCATTGAGGGTTTTGCGTGAGGCGTTCGTACCAGAGATTATCAGGGTGGCATCCTGTTGTTCTGAGGACAGAGACAGG
TAGCGATCGGCAAGTTGAGCGAGTCGGGGCGCTTCTTCCTTCAGTTCGTTCACGCCGGTGATATTTTTCAGGGCGCGCGCGG
CATTACCTTCAGCGGCATACTTAACCGCCTCAAGCAAAACTTCATTCTTCTGTCGCTGAATGTCTTTCATGTAGCTGGTCTGCAT
ATCTGCTTTAATCAGCTGCTCAAAAGGCTTACCGGCTTCTACCGCTTTCGTCTGTGACGTATCCCCCAGGAATACCGCGCGAGC
GTTATGCTTCTCGATCACCTCCATCAGCTGTTTCATCTGTCGGGCGGGTATAACCCCGGCTTCATCAATGAATACGACTGATTTT
TCATCCAGCTTTTTATCCTTCGCTTTGAGGAAAGCGGCAACGGTGCGGGCCGGTAATCCATCATCTTCAAGCGCTTTTTTCTGT
GTCCCATAGGGGGCCAGCGCCGTGACCTTCAGCCCTTGTGACTCCAGCAGCTCTTTAGCGGCCATCGTCATATAGCTTTTACC
GGTACCGGCGTAACCATGTGCGGCCACAAACCGATCTTTGCTCGTCACAATTTCTGTAACCGCGCGCATCTGCTCCTTCTTGAG
GGTTTTCCCGGCAAGCAGCTGGCCTGCAATCTCTGCGGTCAGCTGTCGCGGCATCTGCCCCCGGCCGCGTGATTCGATAGTC
AGAATGGAACGCTCAAGGCGAATACCCTCCACGGTAGTGACGCGGTGGCTGGTCTTTTTAAGCCTGCCGTTTTTAATACCATC
ATCTACCGCAAAACGGGCTTTATCCGCACGCATCCCGCTATTCGTCAGCGAGTCGATCCACTCTTTGCGCGTCAGAGTTTCGG
CCATAACTGAAGCACCGACCTTCAGAGTTGATTGATACCGGGCTTCGCCCTCGATGATGGCGCCCTTCTGTACCGCCTTCAGG

TACGCTTTTTCAACATCGGCTATTGTGGCATGGCCCAGCACCTGCTTATTAGCGATTTGAATCAGCTTCTGGCGTTCAAAGCTG
GCATCGCGCTCTGACAGCGACTTAACTGCAAACTGGATAGCCCGGTCAGCTTTAACCTCCGGGCTGGTAAAATCCGGGGCCA
TGTTGCGCGCTATATCAGCCTCCAGAGGTTTACCGTGTCCCTGCCATTCACGGTTATCAAAATCAATGCCGAGCGTTTTGGCGC
GGCTGGCCCATTCCTGGTGAATTTCTTCACGGGAATGCTCTGTTTTCTTTTCACGCGTAGCCATCGAGACGCGGCTTTTCGTCT
GAGCATCGGCGGTTTCCCGCGTCAGACCCATTGCAGCGAGTCCCTTTTCAATTTGCTCCGACCGGCGGGAAAAAGCGCGAAT
CTGTTCATCTGAAAAATGGGCCATATCGAACGTGTTATTTTTGCTGTTGTAACGCAGCTCATAACCGGCTTTGGTCAACTCCAA
CGCCAGCTCCTGTTTGTAAACATCGCCCAGGTGCATTTTGTTACGCATCAGCTCATCATTTTTGAGCGCGCGCCACTGGCCGTC
CTCGCGCTGGGTCATGTTCATGACAAAAGCGTGTGTGTGCAAATCAGGATCTAGCGCCCTGGAAGTTTCGTGGCGGAAAGTA
GCGACGACAAGGTTATTGGTATTCTGGGTTACTGATTTCCCCTGGCGAGTCGTCCGGGCCTGCGCGAGTTTTTCAGCTTCACG
CACAGCAGCGGCAACAGCTTTTTCATGAGCCTCGATAATGGTTTTATCGCCGTGTATCAGCGCCTGCATGGATACCCCTTTAGG
CGCTGAAAACGTCAGGTCGTAGCCCAGACGCTCTTTTTTGGCATCACCCACGTGTCGCTGCATATGCGTGAAGGTATCTATCTC
TCCGACAAGCAGCTCTTTAAACCGGGCTGATTCAACGTCCCCGGATAAGCCGAGGGCTTCAGCTCCGGTTCCCTGCCAGGAC
GTGAATGATGAATCCTTACTGTAGTAATCATCCTTTGCATCAGAGTAGTAGCCCACAACGCTAGTGACGTTCTGGCGGGTAATC
GTGGTTATATCAAGCATCAGATCTCCCTCAGTTCAATGCCAGGAACAGGGTTTTTGCGATGGTATTTAACGTGTTTAGCCTTGA
ACTTAGCGACGGGCATATCACCAGGCAACGCCAGATAGCCGGTGAGGTTTGGCAACATTGATATTTCGGTAGGCGTTACGGC
ACGAACAACTTTAACGTCGCGGCGTTTACGGACAATCCAGGGCTTCTGAGGATCGGATTCTTTACGCTCAACTTCGCCTTCTA
TCTCACCGAGTGAGCGCGACATTTGATCCAACGTTTCATCACCGAGACGGCTGCCGCCCAGCACGATGTTAGAACGCATGTTA
GCCAGAATTGTCTGAGCCATATCCCGACCATAAACCTTAACCAGCTGAGAATAGGTTTGATAGCCAGCATAAACACACAGACC
GCTTTTACGCCCTTTGGTCAGTGCATCGTTGAGGTTTGGCAGAAACTGGAGTGATTCCAGCTCGTCAATAAATACATTAATGC
GGCTTTCTTTTTCACCCATACCCAGCACGATAGAAAAAATCGAATCCAGCCAGCAGGAAATTAGCGGATTAAGTGACCTTTTC
ATTTCTTCCTGCCAGGTGATAAACAGGGTTCCCGGCTTTCCATCATCAAGCCAGTCACGCAGGGAAAAATTACCTTCCGGCAT
TTTCAAATGTGGGGCAAGATTCTTACTGAGAACAAATCGCGCGCTTCCAACTGCTTTTTCAGACCCGGAAAAAATAGCTTCGG
CAGGCGTCCCCATTAAAAATTCTTTTAATTTTTTCTGGTCAACGTTACAGGCCCAGTGAATAACTTCTTCCATAGTTACTGTGCT
GTATAGGCTGTGAAGTTTTTTCGAAACTTCACTAAAAATAAGACGGCCATAGCCGAACCATTCTTCAGTAGCCATATCAGGGCT
TTCCTGAACAATAGAGTTCACTAAACGCTCGTAATCATATGAACGGCGAATTTCATTGAAAAACACCCAGCCTTCAGTGCGTTT
ATCATAGGCGTTTAAAATAACATCGCCGGGACGATAGAAATTCTTTAAGAACCCCCCATTTGGATCTAAAGCAATATTTTTGCC
GCCTCTAATGATGCTCTTAAATAACAGTTCATTGAAAATTGTGGTTTTACCAGTACCGGTTGTACCGGCAATCGAAAAATGCAA
GTTCTCAGCGTATGTAGGTATGGGGATATTAGCCACGGTTAACTGGTTGACACCTCTTTCGCGTGTTTTATCAGCGAGTGTTCT
GGCGCGAACAAGCTCTGTACCACGATAAATCTTTTTGAATCTTTCGCCTTTAAACACGCGTGATTTATCATAAATGATAAAAGC
GATCAGACCGCCAACACCAATAAACCAGCCAGCAATTAAAGCTGACCATAAAGGCCATAGCGAAAAAGTATTCTTAACCAGAT
ACGGAATCAGGTATTTAGCCGTGGATGGATCAATACCGTAGGTAAATTTTGCAACTAGAAACCATACCATCACTGGAGGCAAA
GTAATTGCAAATAAAAATGCTAAGCCTCTTTCTCTATCGTCCATTTCAGCGCTCCTTTTTTGGTTCCCAGACTTTGTAGCCGTTA
CGTTCAACCTCTGCTTTTGCCGCTTTGGTTTTGCCCGGTTCTGCTATCGAGCGCAGGAGGATTAGCGTTTCAATAGCGAGTGA
TTCATGCAACATCATCTGTCCTGCCGGTGGGAATTTTACGCCAGATAGCGTTTCGGTTATTGCCTTCAGCTCATCGCGCAGTGG
GCCAAAATCCGCATCTGAAGCACGGTCAAAAAGATAATCCAGTTTGCGATTTACGTCGCTCAGCCGGTCGGCAACTATTTTCA
ACCCGGACTCCCGATCACCTGGGCCAGCTTCAATGCAGCGCCGCAGATAATCTGACCGATTACCTCCTGAAACCAGGTCTATAT
AGGCCAAAAGTTCATCTGATACTTTTGCGGTTATTATTGGCATTCAGTCCTCACATTGTGCATTTCTTAAACAAAAAATTGGGAT
CTAACAAGCTGAAATCTTAGTATTACCAAAGTAATAAAGCAAACTCATTATAAAACAATGGGTTATTGGGTGTTTTTAATACCTA
ATTATTACCGAATATTGACGCTATTTATTTTTTTATCTTTTAAATCAGTACGATAGCGTGATTTATCGCGCTGCGTTAGGTGTATAG
CAGGTTAAGGAAAAAAAATCATCTTTTTTGGTAGGAGCGACCTCCGTAGGTTAAGGGTCATTTGGCTAAAAAGCGTCCTATTC
TTTGATGGTCATGCTTGCATGACCATCTGAGCAACCAAAAACTACAGATAAACTACAGAGAAACTACAGATAAACTACAAAAA
ACGATTTACCTTAGCGTTGTCAGACTACTAATAGACTACAAGGAAACTACAAAGAAACTACAAAGAAACTACAAAGAAACTAC
AAATAGACTACTAAAACCGTGGCAGACTACTAATAGACTACAAGAAAACTACAAATAAACTACAAAACTGGATTGACCCCTTCT
TACGAGTGTTGTAGAGTCATCTTCATACAACGGAGGGGGTTATGAATAAACCAGCAGATCTGAAACCCCGCAACTTATCGGCT
GCTGTCAGATTGCGCCTAAATGAAATCGAGAACTGGCTGGACAGAGGGCTAACGCGGCATGAAATTGCTGAAATCCTCGACA
GCGAATACAGCTTTTCGGTAACAGCCAAAGGGCTTGAGATGGCACTGTATAGAACGCGGCAAAACCGAAAAAATGTATTGCA

CAATACACATGATAAGAGTAGCGCGAAGGGTGCAGCGGAAAGTGTATTGCACAATACACAACCGTCTGAGCCTGAAGCGCA
GGAAAGTGAAAAAGCAGAGAGTCCCGGCATTATTGATAAAGAGTTCTTCAATAAAATCGGTGAGGATTTCAACCCTAAGAAG
TTCAACAAAAAATTCTGAGGTGATTTATGAAAGTAGCGGTAATTAATTACAGTGGCAGTGTTGGTAAAACATTAATTTCATCTT
ACCTGTTAGCCCCGCGCCTGACTGGTGCAAAGTTCTATGCGGTAGAGACTATCAACCAGTCTGCTTCCGATCTGGGTATTGAA
AATGTGACCAGTTTTAAAGGTGACAACTTCTCACGTTTGATTGAGGATATTGTTTTTGAAGATGCAGGCATTATTGATATTGGC
GCGTCAAACGTTGAAGCGTTCCTGATGGCTATGTCTCGCTTTGACAGTGGCGCGAACGAATTTGATAAATATGTAATCCCGGT
GACGCCGGATAATAAGGCGATTGATGAAAGCCTGAAAACGGCACACACGTTAAGTAAAGCGGGCGTGAGCAGCAAGAAAAT
TATCTTTGTTCCAAACCGTATTAGTCCAGACAGTGAAGTAGAAGATGTGCTGGCGCCGGTGTTTGAGTTTGTCAAAGAAACGA
AGATTGGCAAAATAAGCAAGAAGGCTGTTATTTATAACAGTGAGGTTTTCGAATATCTGGCGTTTCACCGTATCTCATTCGAAG
TATTGACCGCTGAAGATCCAGAAGAATTCAAATCCCGTGCAAAACAAACAACCGATGCTGACGAGCGCAAAAAACTGGCACG
CCGTTATACATACATGAAACAGGCGATTCCGGTAAAAGCTAATCTCGATAAAGCATATGCGGCTTTAATGGGAGAATAAAATGG
AAAAGCAGCCGGATAAATTAGAAGTTCTGATGGACTGGTTTTTAGGTGACGCGAAGGAAATCACCGCAACTCAGAAAGAAA
TGACGCAGAAACTTTCTGAGCTTTCGGAAAAGCTGGCAAAAGACACCGAAAGTTTAGGAGAGACGGCAGACTCTTTTAAAC
GGGCTTTAGTAGAAAACCAGCGTTCAATTAGCCTGGCAATTAGTGATGATGCTAAGGCGCGCGAGGAATTTCTAACTAAATTC
CGCCGCGCGCAGGCGTCCAGTGCTGAGACGTTTACCCGTCAGATCCTTTTTATTACAGCTGGCTGCACCATCGTGGGCGCCG
CAGTAGGCGCCGCGATAGCGATACTTTTACTGAGATAAAGCAAACCGGGCGTGTCCCGGTTTTTTTGTCAAGCGGAGCGCGG
AGGCCGAAGGCCGGAGGCATTAGTGGCCGCCGCCCGCGTAAGCGGGGCGAGACGGGAACCGGCTCGAAGCGCAGCACGG
CAGAACGGCCCCGCAGGGGCAATGCCCGTTTTAATTCATCGTGACAGTCGCGCGTGACCATCACGGGGAGAAAAATAATGA
ATGACCGACAGCGAGAACTGGCCCGTATACGCCAGGCCCGCCGCCGCGCGCGGCTCAAGGAAGAAGGCACAAGCGTGACA
GTCACGCTAACAAAACAGGAAGAAGCAATGTTGCAGGAGCTGTGCCGGGTTCGCCGTCCTGGACGAACGCCTTATTCAACG
AACGAATTTTTCCAGCTGCTGCTTATCCGCAACTGGCAGCAGTGGCAGGAGCAGAAGGCACAGCTGGGAAAATGCCAGGCT
TGCGGAAAGCTGAAAGCGGAGGGGGGGTGCGAGGGTGAACGGAAAGGCGAAACCTTTAACTGCTGGCTTGCCGTCGAAG
CCAATGAACTAAATTTGTAGTGTATTGTGCAATACACATTTACACAGAAACAAAAACCACCGGCAATTCCTGGAACCGGATACC
TACGGCTATTCCTGGGTGAACGGTACTTTTTGCACCTGGGTGCGCTGAAAAAGCTGAATATGCAGGGTGACGTTCGCGTGCT
GTTCTGCTTTGTAGACTGAATGCGCCAGCTATACGCCTGACTGCTTAAACCTGGTAAAGTTCTGCAACCGGCACTGACCGGAA
AGCAAGGCAGGGAAGACCTAAGCCAGAAACCTTGACTGCTCCCCGCCCTTCAGGGCGGGGATTGCGGATCATGTTCTTCTCT
TTCAGGGATTCAACGCAGACAAGAAAGGCTTTCAATTTCCTATACGTGAACGGCCGCGCAGCGGAAAGAAACAAGCCCGGT
CAATCCGGGCTTGTTTCTTTAGGCGGCTCAGAAATCGCCTAAAGGCCCGGCTTGGCCGGGCAGTCAGTGCTATTTAGTTTGTT
GCAGCAGCTGGCTTAATTTTGCCGCCAGTGCATACGTCGATTGGAAAGCGCCTTGCAGGCGCTGATTAGGCAGCTGGTTGAA
CGCTTCGAGGCAGGCGCGCAGCAATAATTCTTTCTGAGATTCGACTTCTTTTTTCAGTTGGGAAGGGGTGGTAACTGTAGTC
ATGCTTGCTCCTTAGTGAGCCGATATCGGCAATTTTTCGGGTGGCGGTGTTGCCTCCCGATGATTTAATTATCGGTGATTATGCT
TTTAAAGTCAATACAGGTACGGAATTTATTTACCTGTTTTTATGCCCGTCAGGGCATGGAAGGCGACCGCGCCGGACTCCACC
GGACACCGGCCGCAAATCGCCGGAAACTGCGGGACTGACCGGAGCAACAGGCCAACCCCCCTCCCTGCTAAGCCATAACCC
AGCCCGCCGCCACGCAGCTGCCGCACGTCCCCCACGGGGGTGCGCAGTGGGCGCCGCGCGCCTGCGCGCGGGTACGGCG
GCCCGCCTGCGGGTCGCGGCGCCGTACTGCGAGTTAGCGGCCGCCGCGCGGCCGGTTACGGGGGACACCGCACAGTCACG
GCCAGTGCCCCGCTGAGCTGCACAATCCACGGATAACACAATAGCGCACTGGCAAAGGATGCCGACGCCTGAAGGGCGTGG
GCACCCCGAAGGGGCGGGGCGGCCGCTTGCGGCCGGGCGAGTCCGGCGCAGGGTGTGGCCTGCCAAGCGGAGCGCGGA
GGCCGAAGGCCGGAGGCGTTAGCGGCCGCTGCCCGCGTAAGCGGGGCGAGACGGGAACCGGCTCGATGCGCAGCACAGC
AGAGCGACCCCGAAGGGGTAACGCCCGGTGTGGCATCAGGATTTAGTGCAATGGCAGAACATGAGCTGGAGAGATCACCG
GCAAGCAGCAGCAAAGGGGCGGCACAGCCGCCCCGATGGCTGTTTGCCGATACCGGCGATTAATTAGAGCGGTGTTTAATAT
CCCCCGCGTTGCGGGGGACTAGGTTTCAGCAAGTCATGTTAAATACGTGTCCATCATGTAAACTGAAATCCCCAATAAACAGA
TCCCGCGCATAGGCTACGATGTCAAAATATCGGGCTACGGATTCCGGAATATCATTCAGTAGACCGCTATCATTCAGGTATTCCT
CTGCAAAAGTTTCTTCGTCCTTAGCTTCGCCCATATAGGCATCTCTAAACAGGTCGAAATCAGTGCTATTAAACAGATCAACAA
AGGCCACAAACGCCGCTTCGTTACCTTCCTCGCGGGCTTGTTTAAAGCCGTTAATAAAATCCCAGTTGATATGGCACTCTGAC
GCCATACCAGACGGAATACCCTCCCAATCTTGGAACATAAATTCTGGATCAGCCTCATTTGCGTGTAACTCGCGGCAGCGCTCG
TAAAACTCCTCTGAGCTATCAAAATCGGTCAGATCGAGCCAGGCTCCCGCAATGCTTCCGCAGTTGTATTTATGGTAAGTGCCA

ACATAAACAGAAGGGGTCGTAATATCAGTCATGGTGTACTCCTTAAAGCGCCGATACCGGCAATTTTTCGGGCGGCGGTATTG
CCTCCCGATGATTTAATTATCGTTGATTATGCTTTTAAAGTCAATACAGATACGGAATTTATTTACCTGTTTTTATGCCCGTCAGG
GCATGGAAGGCGACCGCGCCGGACTCCACCGGACACCGGCCGCAAATCGCCGGAAACTGCGGGACTGACCGGAGCAACA
GGCCAACCCCCCTCCCTGCTAAGCCATAACCCAGCCCGCCGCCACGCAGCTGCCGCACGTCCCCCACGGGGGTGCGCAGTG
GGCGCCGCGCGCCTGCGCGCGGGTACGGCGGCCCGCCTGCGGGTCGCGGCGCCGTACTGCGAGTTAGCGGCCGCCGCGCG
GCCGGTTACGGGGGACACCGCACCGTCACGGCCAGCGCCCCGCTGAGCTGCACAATCCACGGATAACACAATAGCGCACTG
GCAAAGGATGCCGACGCCTGAAGGGCGTTGGCACCCCGAAGGGGCGGGGCGGCCGCTTGCGGCCGGGCGAGTCCGGCGC
AGGGTGTGGCCTGCCAAGCGGAGCGCGGAGGCCGAAGGCCGGAGGCGTTAGCGGCCGCTGCCCGCGTAAGCGGGGCGA
GACGGGAACCGGCTCGATGCGCAGCACAGCAGAGCGGCCCCGAAGGGGTAACGCCCTGTGTGGCATCAGGATTTAGCACA
ATGTCAGAACATAAACTGGAGAGATCACCGGCAAGCAGCAGCAAAGGGGCGGCACAGCCGCCCCGATGGCTGTTACTTGTC
TTTGTCGCGTAGCACTTTGATTAGGCCGGTTACGGCCGTAATCAGAGCGGCCAGCGAGGTGATGATTTGCGGTAGGTTTTCG
AGGATGGTAGAGGTCATATAGCACCTGTAGAGAAGTTGGCGGGGTGTCGTTTCCGACGGCCGCACTGTAACCGGGCGAATA
AGGCAGGTTGTCAACAGCTTGAGCGAAGCGTCTGTTGACAACCTGCCGCGCCCGGTTTCACTGCGGTCATAGGCGGAACGA
CCCACGCCAACGGAACGGCTTTATGACCGGGCAGCTGAGATACCGGCGAACCTGGCTGGCGGCTGACGCCAGCCGCCAAG
CGCCAGCGCGGAGGGCAAAGCCCGGAGGCCAAGCGGAGCGCGGAGGCCGAAGGCCGGAGGCCGGAGGCGTTAGCGGC
CGCTGCCCGCGTAAGCGGGGCGAGACGGGAACCGGCTCGATGCGCAGCACAGCAGAGCGGCCCCGAAGGGGTAACGCCC
GGAGTCTGCCGCTGTTTATCTCTCGTTCCATCTGAAATCGGCGGTAAGGCCATTAAAAGGGTCAGTTTATCAGGGAGGCGTTA
GCCCCCCATGTTGTTAATCATCAGGCAATATCGTCTTTGTAGCAGGCATAACCGAAGCTAAGCTCTGTTTTCATATAGTGGCGG
GCAAAGTCCCAGGCATCGTGGCCGAAGTCCTCATAATCTGCCAGGACGATTTCGCGGGCTTTGTGCCATTGCTGTACGGAAG
AAAGCGGCAGAACAGGGCAGGGGTGCGACCAGTCAGTGACGGTGTTGCAGATCATATCTGCCAGACGCTCCAGGGAGCCG
TAAACCAGCTCTGTGCGCAGATCCGCCACCATGCGTTGTTTACGCAGTGATGCCAGATAATCAATCTCTTTGGTTATATCAGAAT
TTAAGCGGGTCTGGTAATCCATGATGTACTCCTTTGCGCGCCGATACCGGCAATTTTGCGGGCGACGGTGTTGCCTCCCGATG
ATTTAATTATCGGTGATTATGCCCTCAAAGTCAATATAAGTACGGAATATGCATGCATAATTTTATATCTTGCAAAGCGTTCATAG
AGTGCCTGAATCGCTTTCTGACAGCCTCAATAAAAAAAGGCGGGGATTCCCGCCTTTTTTCTTACAGCTGCTTACGTGGCTTT
TTACGCGTCATATACAACGGTATCGCGCAGTCTACCGCGTACAAAAAGCACGCCAGCGCGCCGCAACCGTACAGAAACGCAA
GCGGCTTATTATCGAAGTAGCTGAAAACCCCTGTCGCCGCACACAGGCCCAGAACAGAGACGCAGGCGCAGGTGATCTGCA
CCAGATCCCTGTATCCCGCACGAACGATAAACCAGGGCAGGGCAAGCGCAGCGGCGCTAATAATTAATGCGAGAGGGACAA
AAACGAGATAGTGATACATGTGAACTCCTTGATGGTTGCCGATACCGGCGATTGTTCGGGCGGCGGTATTGCCACCCGATGAT
TTAATTAGAGGTTTTGCGCGTCCAGGAGATTGACCTGAGCCGGGGTAACGTGAAACTTTTCCCCTTTATGGATCACGTTATGC
GGGGCGCTAATTTCATCACTGATAAAGCTAACCGGGTAACGTTTTTTACCGCAAATCCGCTCGCTAAACCATGCCACTTTTGCC
GCTGGCCGATCCACTGGATGAACAATCACACCGGCCATGCTGCAACCCGTTGCGGGTTCGTCCAGCGTAATGCTTACCGGGA
CTGTATCCCGGTAAAAAAACTTAACCGGCGGCACACCTGCCTGCGTAGCGGCTGCGACAACTGCAAGCCCGATAACCGCTATC
CGATTAATAAGCATTTTATTCCCCTTACTCATGCTGATATCACCTTGCCAGCTGTTACCAGTTTACGAAATTCACTTTCATGAATTT
CACGCCCATGCTCAAGCGTTGAATACACGTTGCCGATAAGCCAGTTACCAGCCGTTTTTGTCTCGGTATACCACCATGCTTCTG
TCAGAGTAAGCAGCGGTTCGCTATCCTCGCCCTTCTCATAGATCCAGATTTTAGTGACGTCTTTACCCGTTTCGTTGCTGCCCTG
AATGGTAGGGTCAAAGGTATGTTCAATCTCTATATCGAAGTAACGCTGAAGGAAGTTAGTAAAGTGCATGACGACTCCTGTAA
GCGCCGATACCGGCAATTTTTCGGGTGGCGGTGTCGCCTCCCGATGATTTAATTATCGTTGATTATGCTTTTAAAGTCAATACA
GGTACGGAATTTATTTACCTGTTTTTATGCCCGTCAGGGCATGGAAGGCGACCGCGCCGGACTCCACCGGACACCGGCCGCA
AATCGCCGGAAACTGCGGGACTGACCGGAGCAACAGGCCAACCCCCCTCCCTGCTAAGCCATAACCCAGCCCGCCGCCACG
CAGCTGCCGCACGTCCCCCACGGGGGTGCGCAGTGGGCGCCGCGCGCCTGCGCGCGGGTACGGCGGCCCGCCTGCGGGTC
GCGGCGCCGTACTGCGAGTTAGCGGCCGCCGCGCGGCCGGTTACGGGGGACACCGCACCGTCACGGCCAGCGCCCCACTG
AGCTGCACAATCCACGGATAATGCAGGAGACGAATCATGATAGGAGGCTGAAGGGGAAATGAGCGGCAGCAGGGGAAGG
GGTTGCCAAGCGGAGCGCGGAGGCCGCAGGCCGGAGGCGTCAGTGGCAGCTGCCCGCGTGAGCGGGGCGAGACGCGTA
GCGGCTCGATGCGCAGCACAGCAGAACGGCCCCGGAGGGGTGACGTCCGGGGGTTCGCTTTTAAAGATTTTCGACCACATC
AGTAAATCGTAGTGACACCATGAAGCAAAAGTATCGTGCAGACCAGAATCAACAACAGTAACAGCAGACCTTTTTTTCGACG
TAATAAAACCGGCCCCAAAGCGCCCGCAAGAAGCACACTCAACACGATTTCCGTTATACTAATCGTATTCATAATCTCTCACGTT

TCCCTTTTTAGAACTCTGCCACACACAGATAAAACCTTATAACAAGCTACAAAACCCGTTATTCAGACGCGGTAATGCCTAGTT
TTTTTGCCAGTATTTCAGATGACAAAGAAACCCTTATCCACTTCCCGGTTACAAGGTGAATGATTGTGGCTTCATGCCCTTTAA
CAGAGTGGGATATTAACTCACGCTGAATGATATGCGTCTCTCCATCATTTCCCGTCACGGCAATTAGGCCCTCTGTAGAACAGG
GGTTGATACTACTAATGTTGGTGTTGTTTTCGGTTTTGTTCAGCATCGCTGATCCTCAAATATCGGTTTGTGTTACGTCTGCCGC
TTTGCGCTGGATAAGCGACTTAAAGAAATCCGACGCCTTCAGAATATCGCTATCCTGGAAGTCGGGAAATGTCGCTTCTGTCA
GCTCCTGCGCCGTACCTGCCCGTATAACGGTTTCATCGCGGATCATCGTTCGCTTAACGCCATCAGCCTGTTTTTTGCTGAAGG
AGTGCCGGAAAATAATCGCGTATTGGCCGCCGCTATCCTGATGAACAGAAGTATCCCAGCGATGATTACCCTGTTTACGATAGT
GGTGATAGATTTGCTGGCCGTAGCGCTCCTGATCGGAATTACGGTAATCGAACATTAACAGTTCCAAAAGCATTAACCGATCTG
GCAATTGCCAGGCGGTAGGGTGTTGAAGTGTCGCTAACATAGTTTCCCCTGAGCGTGACAGTCACGATAAGGCGGGCTTTGC
CCGCCTGGTTATCAGTTAATCAATGGCACGATAAATACGATTCTGGCTTTCGTTCTCCAGCGTATTAACGTACTCCCGCAACAGG
TGATACCGGTTAGCCATCGTTTCGTTCAGTTCGGCTTTGCCTTCTTCATATGCCAGGCCGCAAAAGTAACTGTAGGCATACAGA
CAAACAATAATCCCTACTTCGCGCGCGCTGCATTCACCTTCAAAATAGTTAGGTAACGAGAGCCAAAGAGGTTGGGGCGCTTC
CATAAAAAAACGCGCCATTGCTGGCCTGAAGGTATTCCCAATACCCTCCCTGGTAGTCTTTAGCGTAACGATTCAGAAAGGACT
GAATGAAGTGATCTGCGCTGAAGAAAGCGCCACGAAATGCCGCAGGCATGAAGTTCATGCGGGCGTTTTCAGAAATGTAGC
GGGCGGTGATTTCGATAGTTTCCATGATACTTCCTCTTAAAGCCGATACCGGCGATGGTTAAGCGGCAGGCACATCACCTGCC
ACTTTTTAATTATCGTACAATGGGGCGTTAAAGTCAATATAAGTACGGATTATATTTACCTAATTTTATGCCCGTCAGAGCATGGA
AGGCGACCTCGCCGGACTCCACCGGACACCGGGGGCAAATCGCCGGAAACTGCGGGACTGACCGGAGCGACAGGCCACC
CCCCCTCCCTGCTAGCCCGCCGCCACGCGGCCGGTTACAGGGGACACTGAGAAAACAGAAAGCCAACAAACACTATATATAG
CGTTCGTTGGCAGCTGAAGCAGCACTACATATAGTAGAGTACCTGTAAAACTTGCCAACCTGACCATAACAGCGATACTGTATA
AGTAAACAGTGATTTGGAAGATCGCTATGAAGGTCGATATTTTTGAAAGCTCCGGCGCCAGCCGGGTACACAGCATCCCTTTT
TATCTGCAAAGAATTTCTGCGGGGTTCCCCAGCCCGGCCCAGGGCTATGAAAAGCAGGAGTTAAACCTGCATGAGTATTGTG
TTCGTCACCCTTCAGCAACTTACTTCCTACGGGTTTCTGGCTCGTCAATGGAAGATGGCCGCATCCATGATGGTGACGTACTGG
TTGTGGATCGCTCGCTGACGGCCAGCCACGGCTCAATCGTAGTCGCCTGCATCCATAATGAATTTACCGTGAAGCGGCTACTG
CTGAGGCCCAGACCCTGCCTGATGCCGATGAACAAAGATTTTCCTGTGTACTACATTGACCCGGATAATGAGAGCGTTGAAAT
CTGGGGAGTGGTTACGCATTCCCTTATCGAGCATCCGGTATGTTTGCGCTGATTGATGTCAATGGCATGTACGCCAGCTGTGA
GCAGGCATTTAGGCCAGATCTGGCAAACCGAGCAGTGGCCGTTTTATCCAACAATGACGGCAACATTGTGGCCCGTAATTACC
TGGCGAAGAAAGCGGGCCTGAAAATGGGCGATCCGTACTTCAAAGTCAGACCCATAATCGAGCGTCATAACATCGCTATTTTT
AGCTCTAATTACACTCTCTATGCCTCCATGTCGGCCCGGTTCGCGGCCGTAGTTGAGTCCCTTGCAAGCCACGTCGAACAGTAT
TCAATCGACGAGCTTTTTGTTGACTGCAAAGGGATAACGGCCGCCATGAGCCTTGACGCTTTCGGGCGCCAACTGCGCGAG
GAAGTCAGGCGACACACAACGCTGGTATGCGGGGTCGGTATTGCCCGTACTAAGACGCTGGCGAAGCTGTGTAACCACGCT
GCAAAAACATGGCCCGCTACTGGCGGGGTGGTTGCTCTGGACGATGGCGCCAGACTGAAGAAATTAATGAGCATCCTGCCG
GTTGCGGAAGTCTGGGGCGTCGGCCATCGTACAGAGAAAGCACTCGCCACAATGGGGATCAAAACGGTGCTGGATTTAGCC
AGGGCAGATACGCGCCTAATCCGTAAAACATTCGGCGTTGTGCTTGAAAGAACGGTACGGGAGTTGCGCGGCGAGGCTTGC
TTCAGCCTGGAAGAAAACCCTCCTGCGAAGCAGCAGATTGTTGTGTCGCGCTCATTCGGCCAACGCGTAGAAACCCTGACGG
ACATGCAGCAGGCTGTCACCGGATTTGCAGCGCGCGCAGCTGAAAAACTGCGTAATGAGAGGCAATACTGCCGCGTCATAAG
CGTCTTTATCCGTACCAGTCCTTATTCAGTGCGTGATACACAGTATGCCAATCAGGCAACCGAAAAACTGACGGTGGCAACCC
AGGACAGCCGCACGATAATTCAGGCAGCACAAGCCGCGCTGGCGCGGATCTGGCGGGAAGATATTGCGTATGCAAAAGCAG
GGGTCATGCTGGCAGATTTTAGCGGGAAGGAGGCCCAGCTTGATTTATTCGACTCTGCTACGCCTTCAGCTGGCAGCGAGGC
TTTAATGGCTGTTCTTGATGGTATAAACCGGCGTGGAAAGAGCCAGCTTTTTTTTGCAGGCCAGGGCATCGATAACTCCTTTG
CCATGCGTCGTCAGATGTTGTCACCTGATTACACGACAGACTGGCGCTCAATACCAATAGCCACCATCAAATAATTACCGGCGC
CGTACACGGGCCGGTTAACCCCTCAACCGGCCGAAACAAGTTTCGGCACGGTTTCGCGGTTTTCGGTAAAAGCCGTTTCCTC
TGTATAAAAGATCAGCTAAATTATGTGTATTGCACAATACATATATGTGAGGTTAGCAGTGAATTTGCCTACGCCCGAAACCTAC
GATGAACTTCAGAGAGCCTACGATTTTTTCAATGATAAGCTATTCAGCAACGAGCTGCCGCCATGCCTGATAACGTTGCAGCG
TGAGAAGCGAACGTATGGCTATTGTTCCTTTAAGCGTTTCGTCGGCCGTGAGAGTGGGTACACGGTAGACGAGATCGCTATG
AATCCGGTGTATTTCTCGATCAGAACCATAAAGGCCACGCTTTCAACACTGGTGCATGAGATGGTTCATCAGTGGCAATTCCAT
TTTGGCGAGCCTGGCCGCCGTGGCTATCACAACAAACAGTGGGCGGCCCGGATGGAACGGGTAGGACTAATGCCTTCTGAT

ACCGGCGAACCGGGAGGCAGGAAAGTGGGCCAGAGCATGACCCATTATATTATTGCCGGTGGCCCTTTCGATATGGCCTGTG
ATGAACTGCTGACAGGCCATTTCCGGCTTTCCTGGATGGACAGGTTTCCGCCTTACCAGCCTAAGCCTGGCGCTGTGCTAAGC
CCTACAGGAAAAGGCTATATTGACGACGAGGAAGATGATAGCGAACACGAACAGGAGGTGGAGGAAGGGCGCGACCCGGT
TGAACTCGACGACGAGATCATAGAGGCCATGCGATTTGTAACCCCACCGCCTGAAGCACCGGTGAACAAAACAAACCGGGA
AAAGTACAGCTGCCCGGTGTGTCATATCAATCTCTGGGGTAAACCGGGGATAGTGGTTTACTGTGGTGGCGAGCACTGTAATA
AAGCCGCGTTAGTAGTCTTAAAATAAAGTCCTTTCGGACTTTATTTTTTTTCCATTTCCGAGGTCGTGATGTTATTAATGCTGTA
CTTCGCGGCTTCTTTTAAAACAGTTTCAGCAAGGCTTGCTGGTATCCAGACCTGAACTAATTTTAATGGTTCGCCGTTCTCGGC
TTTAAGAGTGGTGTTCTGGTACAAATCCCAGATTCGCTTAACGGTGCTGGAAATGTTTTGCTTGGAACGGCCTACTCGCATGG
CTACGTCTGATGATTTCTCACCTTTGACAAGCACGGAATAGCCAATATCTGTTGTGATGTGTGCAAAGGAAGCCATTTGCGGC
AGCAGCTGTTTCCATTCTGTTTCTGAAATTCTGTTTTTCTGAGCCATCTGTGGCGCCTCCGTAGTTTTGGTTACAGAAAGGATA
TACTCAGAATAAACAGGGGTCAATACAAGTACGATTTTTATAAACTTTATTTTATTTGAGGGTGAGGCCCGGTGCGGCAGCAG
CGCGGGCCTCGATGGTGCCGCGAAGGTGCTGGCGCCATGCTCGGATTAAAACATGAACCGTGAAGAACTGCGAAACTTGTT
TTCGCGGTTCTGAGGGGTTGACCGAGCCGCGAAGCGGCGCTGGTAAGCGATGATATGCACATATCCACAGGCATATTTTTAA
AAGGTATTTTATAGATTTTTTATCTTTTTAAAGTCTTTTAGAGCTATATAACTCATTGATTTAAAATCATAAATAAGTGTTATCTCTG
GGAATCCGCCCACCTTGTTATGGGAATTGGCCCACCTTACTATGGGAAACAGCCCACCTTACTATGGGAATTAGCCCACCTTGT
TATG

>pQEB1_inv1_IS5708

GGAATTGGCCCACCTTAGACGAAACTGTAAAAAATGTATTTACTTGTTTGAACTTTGTGGTAGTGTGGAGAGTAATTTTTAACC
CACAAAGGCAAGGCGCATGGATAAGTTGCTGAACAAAAAGATAAAAGTTAAGCAGTCTAACGAGCTTACCGAAGCTGCTTAC
TACCTCTCGCTAAAAGCAAAGCGCGTTCTCTGGTTATGTCTTATGCAGACGTATTTCACAGCTTCAGTAAGCGAAGATGATGAT
GAGATGGCTGTACTCGGTGACTCTACTTTCAAAGTAAAGGTGGCTGACTATCAGCAAATTTTTCAGGTAAGCCGTAACCAGGC
TATCAAGGATGTTAAAGAAGGCGTGTTTGAGTTAAGCCGTTCTGCGGTAATCTTTTACCCGAAAGAGGGGCGTTTTGACTGC
GTCGCGCGCCCCTGGCTAACAGAGGCTGGCAGCCGATCAGCTCGTGGTATCTGGGAAATCGAATTTAACCATAAACTCCTGC
GGTACATTTACGGCCTGACGAACCAGTTCACCACCTACTCGCTCCGCGATTGTGGCAGTCTTCGAAATCCCCGGACGATCCGC
CTTTATGAAAGTCTTGCTCAATTCAAATCTTCAGGCTTATGGGTTACTACTCATGCTTGGTTAAATGACCGTTTCCTTTTGCCGG
AATCCCAACAGAAGAACTTGGCAGAGTTGAAACGATCTTTCCTTGATCCTGCACTCAAGCAGATAAATGAGAAAACACCTTTA
CTTGCTAAGTATAGTATTGATGATTCAGGAAAATTTCTGTTCTCAATAATTGATAAGCAAAATCCCGTCTGACATAAATCAGCAC
ACATGAGCCTGTCATTTGACAAATTTTTGTCATGAAGATGGGCGAATTTCCACACAGCACCGGCGCCCGGCAAGATGGGCGG
ATTCCCACACGACAGCGGCGCCCGGCAAGATGGGCGGATTTCCACACTACAGCGGCGCCCGGCAAGATGGGCGGATTTCCA
CACGGCAGCGGCGCCCGGCAAGGTGGGCGGATTTCCACACGGCAGCGGCGCCCGGCAAGGTGGGCGGATTCTCACGCGG
CAGCGGCGCCCGGCAAGATGGGCGGATTTCCACACGGCAGCGGCGCCCGGCAAGGTGGGCGGATTCTCACGCGGCAGCG
GCGCCCGGCAAGGTGGGCCGATTCCCACGCGGCAGCGGCGCCCGGCAAGGTGGGCCGATTCCCACGCGGCAGCGGCGCC
CGGCAAGGTGGGCCGATTCCCACGCGGCAGCGGCGCCCGGTAAGGTGGGCGGATTTCCACACGGCTGCCGCGCCCGGCAA
GGTGGGCGGATTTCCACACGGCAGCGGCGCCCGGCAAGGTGGGCGGATTCTCACGCGGCAGCGGCGCCCGGCAAGATGG
GCGGATTTCCACACGGCAGCGGCGCCCGGCAAGGTGGGCGGATTCTCACGCGGCAGCGGCGCCCGGCAAGATGGGCGGA
TTTCCACACGGCAGCGGCGCCCGGCAAGGTGGGCGGATTCCCACACGGCAGCCTCGCCCGGCAAGGTGGGCGGATTCCCA
CACGGCAGCCTCGCCCGGCAAGGTGGGCCGATTCCCACGCGGCAGCCTCGCCCGGCAAGGTGGGCGGATTCCCACACGGC
ACCGGCGTGCGGCAAGGTGGGCGGATTCCCACACGGCACCGGCGCGCGGCAAGGTGGGCCGATTCCCACACGGCACCGG
CGCCCGGCAAGGTGGGCCGATTCCCACACGGCAGCCGCGCCCGGCAAGGTGGGCCGATTCCCACGCGGCAGCCTCGCCCG
GCAAGGTGGGCCGATTCCCACGCGGCAGCCTCGCCCGGCAAGGTGGGCCGATTCCCACACGGCAGCGGCGCCCGGCAAGG
TGGGCGGATTTCCACACGGCAGCGGCGCGGGGCCAGTGGGATTCAGGAGAATAGGTGTTTTACCGAATGCCCTGACGAGG

CGTAAAAAAACCGCTTGGCGGCGGCCTCATAAAGCAGAAAACCCGCTCAAGGCGGGTTATCTGCTCTGTAGCCTGTGATGCT
TCGCGGGCATCCGGCATACAGCGAGGTGAAATTCTTCTTTTGGCATGTTAATTATACGTCTAACGCGGCATATGATCAAACCTG
TATTAAATAAGCCACTGTACCGTTTATAATGCTCTCAGATCAAAGAGGTAAAGCCCGTTTAGCCGCCTGTGTGATGAGCCAGTT
CAGACTCTTCAAAATCGAATTTGGTACTAAACAGGACCCGAACCGTGGGCAAGCACACGGCAACGGTATAGCCCTCTTCCGG
TTTCGCACCCGGAAGCCTGGGCGGCAGCGTGGTGAAATTCTTCTTTTGGTTAAGTGAATGGCATAACCGGATGGGCGGATTA
GAGGAAAGGGGATTGCCTAGTAACCTACGCGCCACAGAGATGGAGGTGCGGGGAATGATTGAGCTGATTATCGCTATTCTGA
CCTTAATTGCGGCTGTATTGCAGTTGATCAACTGGTTCCTTTAATGGTGCCGGAGTCTGTGAAGGTGAAAGCCTGAACGGGC
AAAACTGAAAGGTTTATAGCCGTCCTTCGGGGCGGCTTTTTTTCGGCAAAATTAGGGTTTTACCGAATAATGCAGAGTTTTAA
GGTGAGAATTTGCAGACTTGGCGTTTTACCGAACATAGATACTCCCTAGGCTGATAGGTGCATTAGTTATCACCCTACCTGAAC
ATATTGTAAAAGATGTCAGTCTCCAGTGACTTGTGTACTATCAACTGACAAGACTCTTACACGCAACGCAGGGGGATGGAGTT
TTATGCTTAGAAAAATAATCAGGGGTAGCGGATTCACTCAGTCAGAAGAAAAACTGATAGAGTTCGCTGATGATGCTTTTTTT
GGTCTTTGGTCTTATCCTAATGTTTATAGCGATGAGGGTTACTCTAAAAATAAAATTGGGAAAGAAGTTAGTGACTTATTAGTTA
TTTTTGATAAAGATATAATAATTTTTTCCGATAAAGCTATTACATACAATAAAAACAAAGATCCTAAGGTTGCATGGCAGAGATG
GTTTAAAAAATCAGTCATACAGTCTTGCACACAGTTATTTGGCGCAGAGAAGTTTATAAAAGATCATCCCGAAAGACTTTTTGT
TGACAAAGAATGCTCAGTTAACCTCCCCATTAAAATAGATAATTCTTTTAATTTTCATTTGGTGGCCGTCACTAATAATATTTCAG
ATCCGGCGATCTCGTACTTTGACAAAATAGAAAAAGGCAGCTCTGCTACTTTAGTTAACATATTTCCTTTAAACGCCCATCAATG
TCTAGAAAATCCATTTTGTGTCGGAGACGTTTATCCTGATAAGACTTTTGTCCATATACTTGATGAGACTGCCCTAAAACTACTG
TTAACCGAGTTAAACACAGCAACTGATTTCATTGGCTACCTTAACGAAAAAGAGAGGGTTGTAAGAGAAAGAACATTATTGG
TCAGCGCTGGGGAAGAAGAGACTCTTGCTGCTTACATTATGGGTGATAAAACCATAATATCAAAAGAAATTATTGGAAACGAT
CAAGGGATGACCATACCGGAAGGTGAATGGAAAAACTATAAAACCACTTTCAATTATCAATATCAGCTCTCAATGAAAAAGGG
TAGCGTTTTCTGGGATAACCTAATCCACAACTTCTCGACAAGTATATTGTCAGCTAACGTTGGTTTTTTTAGTGAAATTGAATTT
TCTACACATGAATTAGGTGTTAGAGAATTAGCCAAAGAAAGTAGGCAATCTAGATATTACCTTTCAAAGAACTTTAAAGAGAA
ATTAAAAACAACTCAGCCTCATCTAAGAACGTCAAGAATGGTCGAATCAATCGATGAGCCTGGAAAGTTTTACTTATTCCTTTT
TTTTCCTAACGATAGCAAGTTGAGTTACTCTGATTACAGAATTCAACGTATATCTTATATAAATGCTTATGCTGAGGTTGCCTTTA
ATAAATACAGACATATTAAAAAATTAATTACTATTGCAACAGAGCCGCAAAATACAGAAGGAAGATCTGAAGACCTAATATATA
GCATATCCCCAGAGAAATTTACCAAAGAGCAAAATGAAAAAGCCAAAAGATTATCAAGAGAATACAAAATACTAAGTGATTTT
TTACCTACTAAAACGACAAAGAGCGATAACTTTAAATCAGTTATATCAAAAGGTGAAAAAATAGGGCGGAATACACCTTGTCC
ATGTGGCTCCGGTGTTAAATTTAAAAAGTGCCATGGTGCGAATAATTAGCATTATTGTATGTATAACGGTAATGGCGCGGCAGA
GAAACCGGCGCGTTCTGCCCTAGTGTTGGCCTGCGGGTTCCCCCGCACCCGCTGTATGTAGTATCGGCAGCATCTGAGAAAA
CCACTACATGTAGTTATCAGCGCCACAACGGCGCGGGGACGAGTGCGGTTCGGAAAAATTGGGGTTTTACCGAATCCGGCA
AAAGATTGCTTCCTATAACGTCCGCTTCTGGCACACAGCAGCCGTTAAGATGTAAGGCCTTACGCCAACTAAATCTAATGGGA
CAGATTTAGTTGGTGATGGTCAAGTAATCTGCAAACGGTCACCAAGTAAAATGCAAATGGGTAGTCAAGTCCGATGCAATTAC
GCACCCGGCAAGGTGGGCCGATTCCCACACGACAGCAGCGCCCGGCAAGGTGGGCGGATTTCCACACGGCAGCGGCGCCC
GGCAAGGTGGGCCTATTCCCACACGGCAGCGGCGCCCGGCAAGGAGGGCCGATTCCCACACAGCACCGGCGCGCGGCAA
GGTGGGCCGATTCCCACACGACAGCAGCGCCCGGCAAGGTGGGCGGATTTCCACACAGCACCGGCGCCCGGCAAGGTGG
GCCGATTCCCACACGGCAGCGGCGCCCGGCAAGGTGGGCCGATTCCCACACGGCAGCGGCGCCCGGCAAGATGGGCGGAT
TTCCACACAGCACCGGCGCCCGGCAAGGTGGGCCGATTCCCACACGGCAGCGGCGCCCGGCAAGGTGGGCGGATTCCCAC
ACGGCAGCGGCGCCCGGCAAGATGGGCGGATTCCCACACGGCAGCGGCGCCCGGCAAGATGGGCGGATTTCCACACGGCA
GCGGCGCCCGGCAAGATGGGCGGATTCCCACACGGCAGCGGCGCCCGGCAAGATGGGCGGATTTCCACACGGCAGCGGC
GCCCGGCAAGGTGGGCCGATTCCCACACGACAGCGGCGCCCGGCAAGGTGGGCCGATTCCCACACGACAGCGGCGCCCGG
CAAGATGGGCGGATTCCCATATCGACATGTATGTAGCTTGTGTTATCCGTGGATTGTGCAGCTCAGCGGGTCGCTTGTCGTATG
GCGTAGTGTCCCCCGTAACCGGCCGCGTGCGGCCGCTAACGCGCAGTACGGCGCCGCGACCCGAAGGCGGGCCGCCGTTCC
CGCGCGCAGGCGCGCGGCGCCCACTGCGCACCCCCGTGGGGGACGTGCGGCAGCTGTGTGGCGGTGAGCGGGATTAGGG
CTTTGCAGGGAGGGGGCTGGGTCGGGCGATACGTTCAGCATTGCGGTTTCCGGCGATTTGCGGCCGGTGCCCGTTTAACTC
CGGCGTGGTCGCCTTCCATGCCCTGACGGCATAAGAAAATAAAACCGCCATGCTGCGGTCATTCATGATTTTGTGGTGTAGCG
ATAAATAGTCATGCGAGAAACGTTGAAGCGCTTAGCAACTGCACCAACTGTCATTTCAGGATCAGCAAGTAAGATTCTAATTT

GTTTAACATCTTCTTCAGAAAGTGACGGTTTTCTCCCTCCCACACGGCCCCTTGCGCGTGCAGCTGCAAGGCCTGAGCGCGTT
CTTTCAATATTGCGGTTGCGTTCAAAGCTAGAGAATATCGCCATCAGATGAGTATAGATTTCCCCTATAACTGGCGCATTTGTGT
CTATTCTGTCCTTGATGGCTATGAAAGTTATTCCGCGTTTCTTCAGGTCGTCGAGTAAAGTAATGACTTGACCCAATGAACCAC
CGAGCCGATCTAGTGCCCAAACTACTAGGGTATCTCCCTCGCGCAATGCTTTCAGGCAGTTCTCCAGTTCCAGCGCACCTTTTT
TGTCGCGCTTTGGGCCGCTACGTGAGGTCTGATCCTGATAGATTTGCTCACATCCAGCTTTTGTTAGTTCGTCAACCTGGTGCG
CCACATCCTGAAGATGCGTAGATTTACGGGCACTGTTGCAAATAGTCGGTGGTGATAAACTTATCATCCCCTTTTGCTGATGGA
GCTGCACATGAACCCATTCAAAGGCCGGCATTTTCAGCGTGACATCATTCTGTGGGCCGTACGCTGGTACTGCAAATACGGCA
TCAGTTACCGTGAGCTGCAGGAGATGCTGGCTGAACGCGGAGTGAATGTCGATCACTCCACGATTTACCGCTGGGTTCAGCG
TTATGCGCCTGAAATGGAAAAACGGCTGCGCTGGTACTGGCGTAACCCTTCCGATCTTTGCCCGTGGCACATGGATGAAACCT
ACGTGAAGGTCAATGGCCGCTGGGCGTATCTGTACCGGGCCGTCGACAGCCGGGGCCGCACTGTCGATTTTTATCTCTCCTCC
CGTCGTAACAGCAAAGCTGCATACCGGTTTCTGGGTAAAATCCTCAACAACGTGAAGAAGTGGCAGATCCCGCGATTCATCA
ACACGGATAAAGCGCCCGCCTATGGTCGCGCGCTTGCTCTGCTCAAACGCGAAGGCCGGTGCCCGTCTGACGTTGAACACCG
ACAGATTAAGTACCGGAACAACGTGATTGAATGCGATCATGGCAAACTGAAACGGATAATCGGCGCCACGCTGGGATTTAAA
TCCATGAAGACGGCTTACGCCACCATCAAAGGTATTGAGGTGATGCGTGCACTACGCAAAGGCCAGGCCTCAGCATTTTATTA
TGGTGATCCCCTGGGCGAAATGCGCCTGGTAAGCAGAGTTTTTGAAATGTAAGGCCTTTGAATAAGACAAAAGGCTGCCTCA
TCGCTAACTTTGCAACAGTGCCCCTTTTAAAAATATGCCTGTGGATATGTGCATATCATCGCTTACCAGCGCCGCTTCGCGGCTC
GGTCAACCCCTCAGAACCGCGAAAACAAGTTTCGCAGTTCTTCACGGTTCATGTTTTAATCCGAGCATGGCGCCAGCACCTTC
GCGGCACCATCGAGGCCCGCGCTGCTGCCGCACCGGGCCTCACCCTCAAATAAAATAAAGTTTATAAAAATCGTACTTGTATT
GACCCCTGTTTATTCTGAGTATATCCTTTCTGTAACCAAAACTACGGAGGCGCCACAGATGGCTCAGAAAAACAGAATTTCAG
AAACAGAATGGAAACAGCTGCTGCCGCAAATGGCTTCCTTTGCACACATCACAACAGATATTGGCTATTCCGTGCTTGTCAAA
GGTGAGAAATCATCAGACGTAGCCACGCGAGTAGGCCGTTCCAAGCAAAACATTTCCAGCACCGTTAAGCGAATCTGGGATT
TGTACCAGAACACCACTCTTAAAGCCGAGAACGGCGAACCATTAAAATTAGTTCAGGTCTGGATACCAGCAAGCCTTGCTGA
AACTGTTTTAAAAGAAGCCGCGAAGTACAGCATTAATAACATCACGACCTCGGAAATGGAAAAAAAATAAAGTCCGAAAGGA
CTTTATTTTAAGACTACTAACGCGGCTTTATTACAGTGCTCGCCACCACAGTAAACCACTATCCCCGGTTTACCCCAGAGATTGA
TATGACACACCGGGCAGCTGTACTTTTCCCGGTTTGTTTTGTTCACCGGTGCTTCAGGCGGTGGGGTTACAAATCGCATGGCC
TCTATGATCTCGTCGTCGAGTTCAACCGGGTCGCGCCCTTCCTCCACCTCCTGTTCGTGTTCGCTATCATCTTCCTCGTCGTCAA
TATAGCCTTTTCCTGTAGGGCTTAGCACAGCGCCAGGCTTAGGCTGGTAAGGCGGAAACCTGTCCATCCAGGAAAGCTGGAA
ATGGCCTGTCAGCAGTTCATCACAGGCCATATCGAAAGGGCCACCGGCAATAATATAATGGGTCATGCTCTGGCCCACTTTCCT
GCCTCCCGGTTCGCCGGTATCAGAAGGCATTAGTCCTACCCGTTCCATCCGGGCCGCCCACTGTTTGTTGTGATAGCCACGGC
GGCCAGGCTCGCCAAAATGGAATTGCCACTGATGAACCATCTCATGCACCAGTGTTGAAAGCGTGGCCTTTATGGTTCTGATC
GAGAAATACACCGGATTCATAGCGATCTCGTCTACCGTGTACCCACTCTCACGGCCGACGAAACGCTTAAAGGAACAATAGCC
ATACGTTCGCTTCTCACGCTGCAACGTTATCAGGCATGGCGGCAGCTCGTTGCTGAATAGCTTCTCATTGAAAAAATCGTAGGC
TCTCTGAAGTTCATCGTAGGTTTCGGGCGTAGGCAAATTCACTGCTAACCTCACATATATGTATTGTGCAATACACATAATTTAG
CTGATCTTTTATACAGAGGAAACGGCTTTTAGGGGTCGTTTGCGGGAGAGGGCGAAATCCTACTATAAGGGCTTCCCCGAAC
AGTCAACGCCGGTTTTTAATTTCTGTCTGTTCATCAATGAAAGGCAAGGGATAAGACTGCGCTACTATAAACGGTCATGCTGA
ATCGCTAGTGATTCGGACCCTGATGAAAGACGCTCGTGAGATCCTCCTTCGTTAATCGGATAGTGATATCCAGTAGGATATGCA
GATACTTCTCACGGGGGTCTAACCCTTTACCATCAACAATTTACAGTTCAGTTACTACTCAGTTAAATTTTTCATGTTGTTGAATA
CAGTCTGAAGTCATCGCCGTAATGTAATGATGCCCAGCACAACCTCGCATTCTTGGCGGCGATTGCAACGACAGCACGCCAGT
ATCCCCGCCTACCAATCAACGAGCAGACCCAACGGCTAAACGGGTCCGTTCTATTCTCTGCCCCTACCATTACAGAACGGGCT
CCCTGGACCAGTAGGGTTCGCAGATAGGCATCACCCGCTTTTGTTATCCTGCCAAGCTTTGATTTTCCGCCACTACTGTATTGT
GATGGCGTTAGCCCCAACCAGGCTGCCAGTTGGCGCCCGTTCTTAAAATCATGGGCATTGCCAATACTGGCGACCAGCGCAC
TCGCTGTGGTGGGCCCAACACCTTTCAGTTCCATTAACTGCTGGCTGCGATGATCTGTTTTGGCCACACGAGATAATACCCTGT
CATATTCGGCGATTTTCTCTTCAATATTAGTGACATGTGTCAATAGATCATCAATGCACAGCCTAACCTGTACTGGCAGAGTTTC
CTTCTGGTCAGAAACAACATGACGCAGAGCATCAGTGCTTTGTGGGGCAATAACCCCGAATTCAGATATCAGGCCCCGAAGA
CGGTTATACGTTGCTGTTCTTTCCTGGATAAATCCCTGTCTTGTCCGATGTAAGCATTGCATTGCCTGCTGGCTTTCGTCTTTGA
CTGGTACAAAGCGCATATGCGGCCGCTGCACTGCCTCACAAATAGCTATTGCATCAGCTGCATCATTTTTCCCGGTCTTACCCG

CCATGCGATAGGGTGAGACAAATTTCGGAGCCATCAACCGGACATCATGGCCATACTGCCTGAATAATCTTGCCCAATAGTGG
GCACCCGAGCAGGCTTCCATTCCAATGACACAGGGAGGTAAACCTGCGATGAGTTCAGAAAGTGCTGCACGCGACACCTTG
GGTTTAACCAGAACAGCTTTACCATTTTGGTCAATGCAGTGAACAGCAAACACATTTTTAGCAAGATCGATACCGACAGTGGT
GATGGTCATAACGAATCCCTCTGGGTCAATGTTTATCCTATGATTGCACGAAAGTTAATCAGGCGCATATCTAGGGGAAGTCCC
TTCCATTCGCTAAGGCTTTGGCCAACGATATTCTCCGGTAAGATTGATGTGTTCCCAGGGGATAGGAGAAGTCGCTTGATATCT
AGTATGACGTCTGTCGCACCTGCTTGATCGCGGCCGCGATAGCTAGATCGCGTTGCTCCTCTTCTCCATCCGCGTTCCAAGCTG
CGGAAAGGCACCCATAAGCGTACGCCTGGTCGAGCAGGCGACGCGGATCGACGTCCAGCGCACGAGAGAATGCGTCCGCC
ATCTGTGCAATGCGTCTAGGATCGAGACAAAGGTCGTCTCTGTCAGCCGGATCGTAGAACATATTGGCGGCGCCAAAGCCCA
CTTCACCGACCAGACCGACGGGATCTATCACCAGCCAGCCGCGACTGGAGAACATGATGTTTTCATGATGCAGATCGCCATGT
AGCCCACGCAGTTCCGAGGCATTGCTCATCATTTGATCGGCTATAATCGCCGCGTGGACGTAGTCAGTTTGACAACCTGCGTT
TTGATCATCGCGCGCCCGCTGAAACAAAGCTGCAAAGCGATCCCGGATCGGGAGAAGGGCAGAAGGCAGGGGTTCCTCAG
ATGCGGCATACAGCTTCGCCATTAGTTCCGCTGCAATTTCGGTCGCCTGGTAGTCGCCGTGCTCGGCAACGATGTGAGAGAGC
ATTCGCTCCCCGGCATATTCGAGCAACATCAGATTGTTCTCACGACCGAGCAACCGGACTGCTCCCCTCCCATTGCGCCATACC
AGATAGTCGGCCCCGCGCAGTTCATCAGCAATGTCTTCTATAGGTTTCAATCCCTTGACGATTGCAGGAGTCCCGTCTGGCAAT
GAAACTTTCCAAACGAGGCTGGAAAAGGTGTCCGCAATGAGAACAGGTTGCGAAACGTGCCAATGAGCAGGAAAAACAGG
CGGCATGAACATCAACCCCAAGTCAGAGGGTCCAATCGCAGATAGAAGGCAAGGCGTTCGCGGTCGGGGGCTTCGATCCCC
AATACATTGAATAGGACAGCGAAGGCGCGCTCTGCTTCATCTGGCGCTGCCCAGTTCTCTTCGGCGTTAGCAATCATGAGTGC
CAAATCGGCATAGCGATCTGCTGTTCCGAGCCGCCCAAGGTCGATCAGACCCGTGCATTGAAGAGTTTTAGGGTCCACCATG
AAGTTCGGCATGCAGGGATCACCATGGCAAACAACCATATCGGTGCGCTCTTGGTCGAGCCGCACCGGTAGCTCTCGTTCGA
CACGAGCCAAAAGATCGAGCTGCGGCGTACTCTTGTCCTCGTCCGGTAAGAAGTCGGGATTGACGGCATTGCGGGACACCA
CATCAACGGCGCGTCCGAACATTCGCGACAGCCTGCGCTCAAACGGACATTGATCAACCGATAGGCTGTGAACAGCGCCAAG
TTGCTGCCCCATTGACGGCCACGCTTTGAGCAAATCCGCTCCAGACAGATCAGCCGCCGGTACTCCCGGAATTGCCGTTATCA
CCAAGCATGCACCCTCCTGTTCCTCCTGCCAGTTGATCACCTCGGGGCAAGCCACACCTCGACCTTTGAGCCAAATGAGGCG
GTCACGCTCTCCAGCGAGCTCACCGCGGCGGGAAGCAGGTGCGATTTTCGCGAAGGCATGCCCGTCACCACGTCGAAAAAC
AAAATCACCAGATTCTCCGCCTCTGACAGGCAACCAGTCAGAATGCGATTCACCAAAAAAAATATTAGTTCGATTCAATGGAG
GTTCCTTCAGTTTTCTGATGAAGCGCGGAGGTGGCTCAACCTGCGAAAAGAAACGAGTTGCTACGTAAGTCCGAGAACATGC
TTTCCATGGTCTCTGAGCTCGCCTTGATGCCCGAGGCATAGACTGTACAAAAAAACAGTCATAACAAGCCATGAAAACCGCCA
CTGCGCCGTTACCACCGCTGCGTTCGGTCAAGGTTCTGGACCAGTTGCGTGAGCGCATACGCTACTTGCATTACAGCTTACGA
ACCGAACAGGCTTATGTCCACTGGGTTCGTGCCTTCATCCGTTTCCACGGTGTGCGTCACCCGGCAACCTTGGGCAGCAGCG
AAGTCGAGGCATTTCTGTCCTGGCTGGCGAACGAGCGCAAGGTTTCGGTCTCCACGCATCGTCAGGCATTGGCGGCCTTGCT
GTTCTTCTACGGCAAGGTGCTGTGCACGGATCTGCCCTGGCTTCAGGAGATCGGAAGACCTCGGCCGTCGCGGCGCTTGCC
GGTGGTGCTGACCCCGGATGAAGTGGTTCGCATCCTCGGTTTTCTGGAAGGCGAGCATCGTTTGTTCGCCCAGCTTCTGTATG
GAACGGGCATGCGGATCAGTGAGGGTTTGCAACTGCGGGTCAAGGATCTGGATTTCGATCACGGCACGATCATCGTGCGGG
AGGGCAAGGGCTCCAAGGATCGGGCCTTGATGTTACCCGAGAGCTTGGCACCCAGCCTGCGCGAGCAGCTGTCGCGTGCAC
GGGCATGGTGGCTGAAGGACCAGGCCGAGGGCCGCAGCGGCGTTGCGCTTCCCGACGCCCTTGAGCGGAAGTATCCGCGC
GCCGGGCATTCCTGGCCGTGGTTCTGGGTTTTTGCGCAGCACACGCATTCGACCGATCCACGGAGCGGTGTCGTGCGTCGCC
ATCACATGTATGACCAGACCTTTCAGCGCGCCTTCAAACGTGCCGTAGAACAAGCAGGCATCACGAAGCCCGCCACACCGCA
CACCCTCCGCCACTCGTTCGCGACGGCCTTGCTCCGCAGCGGTTACGACATTCGAACCGTGCAGGATCTGCTCGGCCATTCCG
ACGTCTCTACGACGATGATTTACACGCATGTGCTGAAAGTTGGCGGTGCCGGAGTGCGCTCACCGCTTGATGCGCTGCCGCC
CCTCACTAGTGAGAGGTAGGGCAGCGCAAGTCAATCCTGGCGGATTCACTACCCCTGCGCGAAGGCCATCGGTGCCGCATCG
AACGGCCGGTTGCGGAAAGTCCTCCCTGCGTCCGCTGATGGCCGGCAGCAGCCCGTCGTTGCCTGATGGATCCAACCCCTCC
GCTGCTATAGTGCAGTCGGCTTCTGACGTTCAGTGCAGCCGTCTTCTGAAAACGACAAACGATGTCAGCCAATAAGTTGTTGT
AATAATCGACAAGTGTTTGTTGCAACTCGCCGTCCTGTAAGACTGCATTATCAGTCTGATTCAGGGCGGCTATGGTGTCATTTA
TACACCATAAACCGATGACGGCTGCACGTAAATCTGTGGATGCTCTTGATGGCGATTCAACGTTTGCTGTCCCACGCATGGCG
ACAGCATTGTTTAGCCCAGCAATAATCCCATTATAAGTTGGGGGGAGGGGCTCCGACAACTGCAAAGCTATCTTTTCTGTATTG
TTTTGGATGTCGTCCGCAAGAATGGTATTCTCGATCAGTTCTCGAGCCAATAATGATGTTTCGAAGCAGACGGCCAACACCGT

CTGCTTCGTTTCTGGTTCTTGTTCTTCCACTGCCAGCGCGTTACTGATTGTTACGCACGTAAGTAGCAAAGTGAATGCGCTCTT
GAGTCTCATCAACGGTTGCCTCCACAGTCTCGCTGGCACTGGAATCAATGTGAAAATTGTTCTGGATCGGATCAACAGGAATA
AATCGATCACAGTCGAACTCTCTTGGAATTATGGTCATGTGCAGTTTCGTTGCTGATGAAAGAGCTTGAATGTACAACTTCTCC
CCACCAATGACAGCAATGCGTTGATCGGTATTCTCATACAGGTTGATCACAGCACGAAGATCAGGAACGACAGTAGCGCCTAT
GACATCTTCTTGTTTTAACGTCGAGGAGATCACGAACGATTCGCGTTCGGGGAGAATTCCTTTCTCCTTGATTCGCTCCTCGGC
GCCATCCTTTTCTAACTGCATGTCACGCATGTCAGTATACGTGTGTTTCCCCATAACGCACACGCGGATCTCTTTAGAAATCCGC
GTAAATCGCGCCATGTCTTCTTTGCATTTCCATGGAATCTTGCCGCCTTTCCCGAATCCCAACTTAGAATCCACAGCGACTATTA
GCTCAAGTTGTGGGTGACTCATTAAAACTCCAAACCAGTCGATGCTGGTTGTTCCAGATATTGCTTACGGAAGGTGGCCAGAT
CAATCAGTTTGATATTTGCCTTTCCGCTGTTGTTGAGATCGTGCACTTTCTTCAATTTGCCGGAAGATCCGGAAGTGGATGCCG
CGATCACCATGGTGGTCTTCGACGAGACTGATGATTGCACTTCACCACCCTCAGCTTCAATCAGCTTCTCCAAGGCCGCGTCA
CGAAAACCGGTGATCACGATCTTCTCACCGGTAAAACGATCCCCCGAAGTGACCTGCTGTTTGAACCCGATGATTGATTGAAT
CTTGTTGAATAGCTCGGCATGTTCAGCCATACCTGCAAGCACTTTTGCGGCAGTTTTGTAGCTGAATTTGTCGACGCTAACGAT
CTGCTCTTCGGTGATGGCATTGAAATCATCCAAGGTCTTAATATCCAAGCCTTCACACAGAGCTTTCATCTTGCGGCGCCCAAC
GCCGCGCCCAAAGTGGGGCATCGACCCCAACCAAACCGACAGGGGAACACTACTGGGTATAGGAAGTATAAACCACCTTTTT
GCTCCTCATCCGAAGTATCTTACCTGAAATTCCCTCACTCGTTTACCGCTCAAGCCCCAATTTTAACTGCCGGTCCAGCCTAAAC
CGCTCTAATAAGGTTCGATTTGGCGGTAAAATCTCTAGCCTGATAGCTCGAGAGATACAAACTGCCCCACCGCCCCGTTTAAAA
GTTGGCAGTGTTGAGCAGTGTTGGATTTGGGGTCGTCAGTCAAAGAGACGACTCTGTGATGGATCGAACAGGCTGGGAGTC
AGTGGCGGCGCTCGTTCTGGTGGCAGCTCACGCTGCTTGGCGGCATTCGCCTTGGCTGTTTTCTGTTTCAGATGCTTGAGAAT
CTGCTCAATGACCTTCGGATCTTCGATGCTGGCAATCACTTTGACGTGACCGCCGCAGTGTTCGCAGACTTCAATATCAATATT
GAAGACTCGCTTGAGGCGTTGCATCCAGGTCATGGCGCGGTGGCGCTCTGCAGGACTCTTGTCACGCCAGTTAGTATCGAGA
CCTTCCGATTTGTCGGGCTTCTTGCCCCGCTTGGCGGGTGTTACTTGAACTCGGTGTTTGCTGTTCGGTGCAAAGACGCCGTG
GAAGCGTGTGAGGTTGACTCGCGGCTTAGGTACCAACGCAGCGAGTTTGGCGATGAAGTCCAGCGGCTCGAAGATCACATG
GGTGGTGCCATTGCGGTACGGAGTTTTGAGCTCGTAACGCACCTGCCCATTGGCGGTTAATGCCAGACGTTTTTCTGAAACC
GCTGGCCGACTAATGTAGCGACACAAGCGCTCAAGCTTATCCCGCTGATGCGCTTCGGCCATCACACCGGCGTGTAGCGAGA
AACCAGCATGGTTGGCTACTCGACTGCTTGAGTCGGCTTTATCCTCACGCCCTGGCAAGGTTTGCAGGGTGAAGACTTTGCG
CCCTTGCTGGGGGCCGACGGCAATGCGATACGTAACCGAAGCACCATGTAATTGAGTCAGCGTATCGTCTTCGCCCTCTTCCA
GTGTCAACCACGTATTCTCGGCATCACGCTCCAAAATCCCACGCTTTTCCATGCAGCGAGCGATGCGATGGCTGAGGGTGTGA
GCGAGCGTATTCAGCTCATCGTAAGTGGGTGCCTTGACACGATGGAAGCGTTGCTTGCCATAGTCATCTTCGGCATAGACACC
ATCGAGAAACAGCATGTGGTAGTGGACATTGAGATTTAGCGCGGAGCCAAAGCGTTGGATAAGAGTCACTGAGCCAGTTTG
TGCAGAGGCTTTGGTGTAACCGGCTTTTTTGATCAGATGAGTTGAGAGTGTACGATAGACGATACTCAAGACCTGGCCCATCA
GCTGGGGATGGCGAGCCAGCAAAAAGCGTAGCTGGAAAGGAAAGCTGAGCACCCACTGGCGAATGGGCTCCTTGGGGAA
GACTTCGTCTATCAGCAGCGCCGCACTCTCGGCCATCCGGCGGGCACCGCAGCTAGGGCAAAAGCCGCGTCGTTTACAGCTG
AAGGCGACCAGACGCTCGTGATGACAATCCTCGCAGCGAACCCGCATGAAACCATACTCCAGACGGCCACATTGGAGGAGG
TCGTTGAATTCTTGTTGGATGTAGCGAGGCAGGTGTTGACCTTGGGCTTCGAGTGAGGCTTTGAAGGCTGGGTAGTGCTGCT
CAACCAGCTGGTAGAGCAGCGTCTGGTCGGGTTGGTGGCGTTCGTAACCGTTTGTTTGAGTGGGCGATTGACTCGCCGTGG
CGTTCCTTGCCAGCGACATGGGTATCCTCCGCTGATACTGTGGTTATGTACAGTATCAGCGGCTTGCGTTCAGACGTCCAGTCT
GGCCCTAGACATCGCTAAATGCTTAACCCGCAATAGCCCTCACGAGTTGTTATCAGCCACTACCGGTTGAGCGAGAAGGTTTT
GGGTTCAGGGTGCTATTGCTCCACCAATCACAATACTGAAGCCCCAACTGTTATCAGTTGGGGCTTTTTCTTGTCTGTTTGCGG
CGGTTGCGTTTTATCGGTAGTCGTCGAGCTCTGCACCATCCCACATAAGAGCTTAACGGTGCGATCTTCAACGCCATCACACAA
AACTTTCTTTTTCACGCACAGTCAACTTATTGGATGTTTTATTAACAACCCAAAAGGAGATATTTAGCGGGCGGCCGGAAGGT
GAATGCTAGGCATGATCTAACCCTCGGTCTCTGGCGTCGCGACTGCGAAATTTCGCGAGGGTTTCCGAGAAGGTGATTGCGC
TTCGCAGATCTCCAGGCGCGTGGGTGCGGACGTAGTCAGCGCCATTGCCGATCGCGTGAAGTTCCGCCGCAAGGCTCGCTG
GACCCAGATCCTTTACAGGAAGGCCAACGGTGGCGCCCAAGAAGGATTTCCGCGACACCGAGACCAATAGCGGAAGCCCCA
ACGCCGACTTCAGCTTTTGAAGGTTCGACAGCACGTGCAGCGATGTTTCCGGTGCGGGGCTCAAGAAAAATCCCATCCCCGG
ATCGAGGATGAGCCGGTCGGCAGCGACCCCGCTCCGTCGCAAGGCGGAAACCCGCGCCTCGAAGAACCGCACAATCTCGTC
GAGCGCGTCTTCGGGTCGAAGGTGACCGGTGCGGGTGGCGATGCCATCCCGCTGCGCTGAGTGCATAACCACCAGCCTGCA

GTCCGCCTCAGCAATATCGGGATAGAGCGCAGGGTCAGGAAATCCTTGGATATCGTTCAGGTAGCCCACGCCGCGCTTGAGC
GCATAGCGCTGGGTTTCCGGTTGGAAGCTGTCGATTGAAACACGGTGCATCTGATCGGACAGGGCGTCTAAGAGCGGCGCA
ATACGTCTGATCTCATCGGCCGGCGATACAGGCCTCGCGTCCGGATGGCTGGCGGCCGGTCCGACATCCACGACGTCTGATCC
GACTCGCAGCATTTCGATCGCCGCGGTGACAGCGCCGGCGGGGTCTAGCCGCCGGCTCTCATCGAAGAAGGAGTCCTCGGT
GAGATTCAGAATGCCGAACACCGTCACCATGGCGTCGGCCTCCGCAGCGACTTCCACGATGGGGATCGGGCGAGCAAAAAG
GCAGCAATTATGAGCCCCATACCTACAAAGCCCCACGCATCAAGCTTTTGCCCATGAAGCAACCAGGCAATGGCTGTAATTAT
GACGACGCCGAGTCCCGACCAGACTGCATAAGCAACACCGACAGGGATGGATTTCAGAACCAGACGCAGCCGCAGGCGCC
GGAAACTAAACCGATGTCTGTAGGGTTACCGCTGGATCTGCGTGAATTCCAGCAGCAGCAAGAGAAAGACTTTCTACAAACC
AGCTTACAGCAGGCAAAATTTAACCAGAAAAAAGCCGCTGAATTACTGGGTCTGACTTACCATCAGCTTCGCGCTTTGCTGAA
AAAACACCAGATTTAACGCACATTTGCAGATGTTATATTGGCGGATTTGACGCATAACCTCATCAGGGTTTACCATGACGCCAT
TACTGTATAAAAAAACAGGCACAAAATATGGCTCTGGCACTCGTTGGCGAAAAAATTAACAGAAACCGCTTCACCGGTGAGA
AAATTGAAAATAGTACATTTTTTAACTGTGATTTTTCAGGTGCCGACCTGAGCGGCACTGAATTTATCGGCTGTCAGTTCTATG
ATCGTGAAAGCCAGAAAGGGTGCAATTTTAGTCGTGCGATGCTGAAAGATGCCATTTTTAAAAGCTGTGATTTATCCATGGCG
GATTTTCGCAATGCCAGTGCGCTTGGCATTGAAATTCGCCACTGTCGTGCGCAAGGCGCAGATTTTCGCGGCGCAAGTTTTAT
GAATATGATCACTACTCGCACCTGGTTTTGCAGTGCATATATCACTAACACAAATCTAAGCTACGCCAATTTTTCGAAAGTCGTG
TTGGAAAAGTGTGAGCTGTGGGAAAACCGTTGGATGGGGGCCCAGGTACTGGGCGCGACGTTCAGTGGTTCAGATCTCTCC
GGCGGCGAGTTTTCGACTTTCGACTGGCGAGCAGCAAACTTCACACATTGCGATCTGACCAATTCGGAGTTGGGTGACTTAG
ATATTCGGCGCGTTGATTTACAAGGCGTTAAGTTGGACAACTACCAGGCATCGTTGCTCATGGAACGTCTTGGCATCGCGATT
ATTGGCTAGTCTTCAGGGAGCGGTGAATATTCCGCCCCCTGCACAGCTTTTTACCCTTCAGGCATCGCTGAAGAGTGGTGTGT
GGAAATTTTCCACTCTTTACCGTCCCATGCATAAGTGAATGTGTAGCGGGCTGAAACCGTTGATTTGTCCGCGAAGGTAAATG
TGTAGGTACCGGTGTCTATCGCCTTATTACACCCCAGGCGAATGGTACGCATGTCAATTTTCCCGGTCGGCTTTTTCGCAAGAA
AATGCTCGAAATAAGGCACTGTTGCAAATAGTCGGTGGTGATAAACTTATCATCCCCTTTTGCTGATGGAGCTGCACATGAACC
CATTCAAAGGCCGGCATTTTCAGCGTGACATCATTCTGTGGGCCGTACGCTGGTACTGCAAATACGGCATCAGTTACCGTGAG
CTGCAGGAGATGCTGGCTGAACGCGGAGTGAATGTCGATCACTCCACGATTTACCGCTGGGTTCAGCGTTATGCGCCTGAAA
TGGAAAAACGGCTGCGCTGGTACTGGCGTAACCCTTCCGATCTTTGCCCGTGGCACATGGATGAAACCTACGTGAAGGTCAA
TGGCCGCTGGGCGTATCTGTACCGGGCCGTCGACAGCCGGGGCCGCACTGTCGATTTTTATCTCTCCTCCCGTCGTAACAGCA
AAGCTGCATACCGGTTTCTGGGTAAAATCCTCAACAACGTGAAGAAGTGGCAGATCCCGCGATTCATCAACACGGATAAAGC
GCCCGCCTATGGTCGCGCGCTTGCTCTGCTCAAACGCGAAGGCCGGTGCCCGTCTGACGTTGAACACCGACAGATTAAGTAC
CGGAACAACGTGATTGAATGCGATCATGGCAAACTGAAACGGATAATCGGCGCCACGCTGGGATTTAAATCCATGAAGACGG
CTTACGCCACCATCAAAGGTATTGAGGTGATGCGTGCACTACGCAAAGGCCAGGCCTCAGCATTTTATTATGGTGATCCCCTG
GGCGAAATGCGCCTGGTAAGCAGAGTTTTTGAAATGTAAGGCCTTTGAATAAGACAAAAGGCTGCCTCATCGCTAACTTTGC
AACAGTGCCAGACAAAAGGCTGCCTCATCGCTAACTTTGCAACAGTGCCGATTTACGTGCATAGCCGATTTTCATTCTTTTCTC
GCTAATTAGTTATGGGGTTATTGTTATGTTGATACAGTAACGAGTTTTGTTACATGAGGGGAGTCATTTTTCGGGAGAAGTCAG
GACTTTTCAAGACTGTCACAAAAACCATCGTTTTTGATACATTAATTTAACCAATAGGTTGCAGATCAAATCGTCTGTAACAGCC
TTTCTGGCTGTTTGTATATAATCATGAAAAAATGGTGAGTAGAGTTTCAGGGTAACAGGGGATGCTTATGTCGGTTTTCCACAA
CTGGCTACTTGAGATCGCATGTGAGAATTACTTCGTCTACATCAAACGCCTTTCCGCCAACGATACCGGCGCAACAGGTGGTC
ACCAGGTAGGGCTTTATATCCCTTCAGGTATCGTTGAAAAACTCTTTCCGTCTATCAACCATACCCGTGAACTGAACCCTTCGGT
TTTTCTCACCGCACATGTGTCATCGCATGATTGCCCTGACAGCGAAGCCCGGGCAATTTATTATAACAGCCGTCATTTTGGTAA
AACCCGGAATGAAAAAAGGATTACCCGCTGGGGTAGAGGCAGCCCACTTCAGAATCCTGAAAATACAGGGGCTCTGACGCT
CCTGGCTTTCAAGCTTGATGAGCAAGGGGGGGACTGTAAGGAAGTAAATATTTGGGTATGCGCCAGCACTGATGAAGAGGA
CGTCATTGAGACCGCTATTGGTGAAGTTATACCCGGAGCGCTTATATCCGGCCCCGCAGGACAGATTCTAGGCGGACTATCTCT
ACAGCAAGCGCCAGTAAATCATAAATATATTCTACCTGAAGACTGGCACCTGCGCTTTCCGTCGGGAAGTGAAATTATTCAGTA
TGCAGCCAGCCATTATGTGAAAAATTCCCTTGATCCGGATGAGCAACTTCTTGACCGCCGGCGCGTGGAGTACGACATATTTC
TATTGGTTGAGGAACTGCATGTTCTGGATATCATCCGGAAAGGATTTGGCTCTGTGGATGAATTTATTGCGCTGGCCAATTCTG
TCAGCAATCGCCGTAAATCCAGAGCCGGGAAGTCTCTGGAACTGCACCTGGAGCATCTATTCATTGAGCACGGCCTGCGACA
CTTTGCGACGCAGGCCATCACAGAAGGTAATAAAAAACCCGATTTCCTTTTCCCTTCCGCAGGGGCTTACCACGATACTGAGT

TTCCCGTAGAAAATCTGCGCATGCTGGCAGTCAAGACTACCTGTAAGGATCGCTGGCGTCAGATACTGAATGAGGCCGATAAA
ATTCATCAGGTGCATCTGTTTACACTCCAAGAGGGAGTTTCTCTGGCTCAATATCGGGAGATGCGGGAGTCGGGTGTCAGATT
GGTCGTGCCATCATCGCTGCACAAAAAATACCCGGAGGCGGTGAGAGCTGAGCTAATGACGCTAGGTGCGTTTATTGCTGAG
CTGACAGGGCTTTACGCAGATATTCCATAGATTATCTCCCGGCATAAATACCGGGAGGAGCGATCAGATTCGTTCAACCTTGCA
CGAATCGGCATTAACCGCTTTCAGGATATAAGGTTCAAGCAGTTTGGCTACGGCTTCAAACACGGGCACCACTACGGAGTTAC
CGAACTGCCTGTACGACTGAGTGTCTGACACAGGAATGCGAAAAGGCCTGCCATCTACTTTTTCAAAACCCATAAGGCGCGC
GCACTCTCGTGGAGTCAGCCTGCGGGGCCGATGCGCCTGATTTTCTTCGTTCGCGAAGTCTGTTTCACCTGTGGCCATATCCC
AGCCACGGTCTATCAGAATTTCAGACCCGTCTTTGTGATAGCGAGCAGAAAGCGTACGGGCAATGCTTTCTTTATTTTCAGGA
TTAACGAGGCCAAAACCGAATCCGTTACCCTTAGCTGCGTGCTTTTTGGCGTAGTTATAGAGATACTCCCAGAGTTTCGGCGT
CAGTATATATTTGCTGTCAACCACGGGTTCCAGCAGTTCGCCAAATGACGGACGCTGTTCCGGATAAAAACGACTAATATCGC
GCAGGGTAAAGCCCTGGTGAATGTTCAGATCACGACGGAAACCGACCAAAACGATACGTTCTCGGTGCTGAGGTAAAAAGT
GCTTTCCGTCGATAACTTTAGGATCGTTTTTGCCCATCTCAGCTGCATCCGCAACTTCATAGCCCAGTTCGTCGAGGGTATCCAT
GATGACTTTAAAGGTTTTACCCTTGTCATGGCTCTTCAGGTTTTTAACGTTTTCAAGAACAAAGATGGCAGGTTTTTTTGCGC
GTATAATACGCGCCACATCGAAGAAAAGCGTTCCCTGAGCCTCACATTCGAAACCATGCGCGCGCCCGAGCGAGTTTTTCTTG
CTTACGCCCGCAAGGCTGAACGGTTGACAGGGGAAACCTGCTAGAAGTACATCATGATCCGGCACATGCTCATTAATGTAAGC
ATAGGCATCGTTTTCAGGTACTTCAGGTTTATCACTGAGCGTGACTTCCCGAATATCGAGATTGAAAGTGTGTTCCTGAGCATC
GTTAAACCAGTTAGCTTTATATGTGCGCACAGCCTCTTTATTCCATTCACTGGTAAAAACGCACTGGCCACCGATGGTTTCGAA
GCCCTTCCGTATACCTCCAATCCCAGCAAACAGGTCAATAAACCGGAAGGCATAGTCAGGGTGATGTGCAGGCGCTTCCGGA
AGCATTTTTCGTAGAAGTTCCTCTTCGGCTAACGTCAGCGTCTTAGGTGAGCACTTACCATTAATCCAGCGATTAAGAGTCTCG
CGACTCCACTCATTTTTACCAACTTTTCTAAGCAGTTCAGCCACGTACTTCTGGTCATAGATTTCCAGCACCTGCCCGAGCAGC
TTTTTATAATTTTCCTGTCGCAGTTGTTCTTCCGCTTCTGCTTTCTCAAGCAGATCCTGCGCCAGTAATTCAAATTCAGACATATT
GCCTCCATTGGGTCTTATGGGTGAAACTGTATCACTCATTTGACCCAGATTGAATGTTTTTATCTGGATATTTAAACAGGTTTATT
GTTAGGTAACGCACGTTGGCCACGCTGGAGCGTCTTCTGGGCCTGCTGTCGGCCTTTGAGGTCGTGGTATGGATGACGGATG
GCTGGCCGCTGTATGAATCCCGCCTGAAGGGAAAGCTGCACGTTATCAGCAAGCGTTACACTCAGCGCATTGAGCGACATAA
CCTGAATCTGAGACAACATCTGGCAAGGCTGGGACGGAAGTCACTGTCGTTCTCAAAATCGGTGGAGCTGCATGACAAGGT
CATCGGGCATTATCTGAACATAAAACACTATCAGTAAGTTGGAGTCATTACCGGTTCTCTTTGTCTTTTAGTGATTCTATAAACCT
CATTACGTCTGAATATAAAAATCTATTATTTGATTTATGTGGCTCATGAGGTTGTGGGATGGTCTTGTTTTTGAATGTGCCAGTTT
TCTTAATGGCAAAGATTAATTCACCTTCTGTTATTCCTAACATTTCAGCAAATGTTTTTGCTTCTATAGTTACTGACTTCATTTAAT
TAACTCTCATGGTATCGATTTTCTTTACCGGCATCTTTAACAATGGTGCTCGTTTCTAGTGTTGCTGCGGTACGCTTCATCATCGT
CTGCGGGGCGGTTGCGATAGTGAAGGAGCTGCCGGGCGTGAGCAAATCTATCAGGCGCTGGCCGCTGATAATCTCCATCCGT
TCACTGGCAATACTGACAGATTTTGAACCTGCGCCGGTTTTCCCGGTATGGCAAAACAGACCGCGACAGTTATGACGTTTAAG
CAACTTCTCGAACTCCTGTACGTGCTGTAAAGCAATATGGCCGCGATAGCGTTTAGCCTGAATAAGATAGCGATATTTTCCTATT
ATTACCTGGCCGTCAATGCCTCCATCGCCGGTATAGCGTTTGTTTCTGATGGTTCTGAAGCCATGCGCGGCTTTGTTGAATAAA
TCGAACTTTTGCTGAGTTGAAGGATCAGATCACGCATCTTCCCGACAACGCAGACCGTTCCGTGGCAAAGCAAAAGTTCAAA
ATCACCAACTGGCCCACCTACAATAAAGCCCTCATCAACCGTGGCTCCATAACTTTCTGGCTGGATGATGAAGCTATTCAGGCC
TGGTATGAGTCGGCAACGCCTTCATCACGGGGAAGACCTCAGCGCTATTCTGATCTCGCCATCACCACCGTTCTGGTCATTAAA
CGCGTGTTCAGGCTGACCCTGCGGGCTGCACAGGGTTTTATTGATTCCATTTTTACACTGATGAATGTTCCGTTGCGCTGCCC
GGATTACACCAGTGTCAGCAAGCGCGCAAAGTCGGTTAATGTCAGTTTCAAAACGTTCACCCGGGGTGAAATCGCGCATCTG
GTGATTGATTCCACCGGGCTGAAGGTCTTTGGTGAAGGCGAATGGAAAGTCAAAAAACACGGCAAAGAACGCCGTCGTATA
TGGCGAAAGTTGCATCTGGCCGTTGACAGCAACACACATGAAATCATCTGTGCAGACCTGTCGCTGAACAATGTGACGGACT
CAGAAGCCTTCCCGGGTCTTATCCGGCAGACTCACAGAAAAATCAGGGCAGCATCGGCAGACGGCGCTTACGACACCCGGC
TCTGTCACGATGAACTGCGGCGTAAGAAAATCAGCGCGCTTATCCCGCCCCGAAAAGGCGCGGGTTACTGGCCCGGTGAATA
TGCAGACCGTAACCGTGCTGTTGCGAATCAGCGGCTGACCGGGAGTAATGCGCGGTGGAAATGGACAACAGATTATAACCGT
CGCTCGATAGCGGAAACGGCGATGTACCGGGTAAAACAGCTGTTCGGAGGTTCACTGACACTGCGTGACTACGATGGTCAG
GTTGCAGAGGCTATGGCCCTGGTACGAGCGCTGAACAAAATGACGAAAGCAGGTATGCCTGAAAGCGTGCGTATTGCCTGA
AAACACAACCCGCTACGGGGGAGACTTACCCGAAATCTGATTTATTCAACAAAGCCGCCATGCGCTTCAAATCCTTCCAGCAA

CAGTTCTTCAAACACAAAAGGATCAATTTTCCTCAGGTAGTTAATTTTTTGTGGGAAGCCCGGCAACGTCTTTATGCGCTCCA
GCACCCGCCGCGCACTTTGCTGCTTCCTTTTGTGTCGTCGGTTGCGTACTGAACGCCGGAAGAATACAACGGCAAACAGTGC
GATGGCGCTGCAAGCCCATAGAATAAGGTTTTCTGTAGTGGGGAAGGGGAACATGGTGATAGTGTGCTTTCTGTGGGTAAAG
AAAAGGGCGGTTAAACCGCCCTGGTGTTTAGCGACGGCTGTAAACCTGCCACGAAGCGCTGCCTGACTGATTTTGGCAAATC
CGCCCGTAAAGTACGGTGCCGGTCGAGTAGCGGGCGCCATTCAGATAGCAATAGCCGCTGGATTGCAGCAGTTCGTTGCGCA
GCTTTTCTTCCTGCTTTGATAGCCGGGACTCCGCAGACTGCAAACGAACGGATAATTCGTTAATCTGGCGTTGCTGGTTATTCA
TCTGGCTCTGCATCGCATTACCTTTATCTTGGCTGACGCAACCAGTTAAGAGAGCTGTACAGGCTAATGCACTTAATATAATTTT
TTTCACGTTGGCTCCTTAAATTGAGATTATTCCTAGCCCGCCTATAAGCGAACTTTCCCGTATTTACTTATGATCTGGCTTATCATC
GACTGGTTACTTCCACCTTCGCCATTATCCGGGCATTCATTAAGAAAAGCCTTCCTGGCATCCCTCGTGTGGTTGGGTAAAAAG
CCGTGCTTGTTCTTTTTAACGATATTGAAGAAAGCAGCTTCAGCACTGTTACACTCGCTTCCGCCGCTATCGCCGGTGAGCTTG
CCCGCCATGCACATAATAACTTTGCAGGGATCTTCAGCATGGCTGGCAGGAAGATAAAGCAGACTACCAGCTGCTATCAGAG
GGATTAAGAGTTTCTTCATTGTTTTGTCCTTAACAGTTTGTTCAGATATACACCCGCCAGAATGTTGATAACGGTAAGTAATATT
AATAATAACGCAGAGTTATAAATAGATTTGTAACCTATATCGCCTGCGATATATTCGACAATAAAAACGAAAATCGTTAACATTGC
GAACAACTTAATTAACTTTCCTGTAATTTTCTTAACGATATAAGCAACTGAATAAGAGCCGGATTTGATGATGCTGGCGAGTAA
CCTGATAGCATGGACTCGGAATTTTCTTAAAATGGGCGGAATGGAAAATGCCTTTTTCTTCCTTCTGCCTTTTTGCTTTTCCGC
AAAATCAATAACATCGCCCATTTTTATACTCTCCGGTTATATCTTTAGATCATCAAGTGATAAACCACTATCAAGCAGCTCTTTGA
GCCATGTTGGTCGGCGGCCAGTCCCGGACCAGGTATTTTCTGCGTTCCTGGGGTCGCGGTATTTTACTTGTCTATGTTCTCTGG
GGTTGGCAACGCCCTGCATTTTCATGGCTTCCAGGCTAATGCCAGCGTCCAATTTTGCAGTTAGCCAGGCCGGGCGCTTCCCT
ATACCAGTCCACGTATTAAAAGGGTTATCCGGGTCACGATACATGGGTTCACCTTTGGGGCGTTTTTCTGATTTCAGGGGAAC
CAGCATTACCTTCCTTCCTTCAGAGGTTTTCTTTTCGTTTCTTCTTGACACGATCTCATTAGCCCTTATCATAAACATAAGTCGTC
GAAATTACAATTACGCGGTGAAACGTAAATGAGTAAACATCCAAAACTTCTGGTTCTCGCTCTGGCCTGCCTTGCTTGTGCTG
GCCGTGCCAGTGCTGCGCCTGCCTCAGATGAAGTTGCCAGGCTTGCGCAGAGATGTGCGCCTGATGTTTCACCCTTAACAAT
GGCGTACATCGTCGGCCATGAGTCCTCAAATGGGCCGTACAGGATCAATATTAACGGTGGTAGTACCCAGTTAAAACAGCAAC
CACGTACTGAAGCTGAGGCCGTCAGCGTTTCGAAAGTTCTGCTGAAGGATAATAAAAGTTTTGATATGGGCCTTGCACAAATT
AACTCAAATAATTTAGTGGGCCTGGGTCTTTCGGTTGACGATATTTTCAAGCCCTGCATCAACCTGCGGGCGAGCCAGACCAT
CCTTAAAGCCTGTTATGATAGCGCCCTGAAATCCTATCCAGCCGGGCAGGTTGCGCTGAGACACGCGCTTTCCTGCTACAACA
CCGGCTCACTCATAAACGGGATTTCTAACGGGTATGTCACGAAAGTTATCAACGTGGCGCGTCAATCAACTGATTTGAAAATC
CCTACGCTGCTACCTGATGGCCAGACCAGTGAGGACAGCACCGCGACTGAGCCTCAGCAGGCAAAAAGTACGGCCACGCAG
TATGACGGTGAACAAGATGTTTTTGGTTCGGGTGATGGCGATGCCTTCAGCCGAAATAATACGGATGCCTTTTTAACCAAACA
GGAAACAGCGAAGGGGGAGTGAGGTTATGGATGGAACGTTTGTACCTTGTATTGCAATCACAGATCCTGGAGCTATTTGAAA
CAGGAAAAGTGAAGGAGGTAACGATAAAACGGGTTTCATTAAAGACGTGGTATCCCGTTTTTCAGATAGACGATGAACAGTT
GGGCCAGATCGCATGTTCCATTCGGGTTAACAAAGAGCATGAACTACGAACCTGGGCTGATTTAAGGCTACTGGCAGAGTTT
TTGAAAGATAAGTGTGGCGTTGAAGAATGCCGGTTAAATCTGCAATCAACAGAAGATAGTGAGTAAGGAGAAAGTATGACCA
CGTTGTTTAAGAAGTATGGCCCTGCGGTAGTTATGGGCGTTTTGTCCATTGCCCTGCCGCAAATTGCGCTGGCCGCTGGCACC
GATACTGGTGAATCAACCGCTACATCAATCCAGACGTGGTTGAGCACATGGATTCCAATTGGTTGTGCTATTGCGATCATGGTT
AGTTGCTTTATGTGGATGCTTCACGTAATCCCAGCCAGCTTTATTCCTCGTATCGTAATCTCGCTGATTGGTATTGGTTCTGCATC
ATTTCTGGTTTCCCTGACGGGCGTAGGAAGCTGAACAACGCGAAAAGGGGGGACTTTTGTCCCCCAAAGTGAGGACTACAA
AGATGTTCGTTGACGGGAAAAGACCGCTTTTCAAAGGTGCGACTCGCTTACCTCGCGCGCTGGGTGTACCACGTAATGTAGC
TATGATGATATTCATGATTTCTGCCTCGCTTTTTATGATTATTCATATGTGGGCGATCCTGGTGTTCGTCTTTTTGTGGATTCCTTC
AGCTGCATTAACAAAATATGACGACCGCATGTTTCGAATTATGGGCCTGTGGTTGAAAACCAAATTCAGTAATTGGTTTGATTC
TCCGTTTAAGCAGTGGGGAGGATCGTCTTATTCCTCTGTTGACTACAAACGTAAGGGTTTAAAATAATGAGAGCTGCCACCGC
TACGAAGCCAAAAAAAAATTGATGCCTACCGTAAGGAGCCATCAGTAAATAAAAAGTATTTGCCCTATTCTTATCACCTCAATGAT
TACGTGATTTCGATGGAAAACGGCGATCTGATGGCTTTTTTCAAGCTGGATGGCCGCACACATGACTGCGCATCAGATCGGG
AACTGGTCACCTGGCATAAAGACCTTAATACGCTGGTCAAGAGCTTCGGAACAGACCATGTAGAGCTGTGGACGCATGAATAT
CACCATGAGGCTAAAGAGTACCCGGATGGTGAGTATGACCATTTTTTCCCTGCTTATGTTGATCAATATAACCGTAAGCTGCAC
GGTGATTCCAAGCAGCTGATTAATGACCTTTATCTGACCGTTATTTACAAACAGGTAGGGGATAAAACACAGAAGTTTCTGGC

GAAATTTGAAAAGCCGACTCGTGACGAAATTCAGCGAATGCAGAATGAGGCGCTTGAAGGTCTGGAAGATATTTCTGAACA
AATCCTGGAAGCAATGAAGCCGTATGGCATTCAGCAGTTGGGTATCTATTATCGTGACAAACGCGGTGTTGAAATTCCTGCGC
CTGATAAAAAAGAACGTGAAGAACTTGCTGAAGTCGATGAATCAGACATTTTTGACGAAGCCATTGTTATCGAACGCAACGA
GCCTGAACCTTCGCAGGCTCACGCTTATTCAAAAGCGCTGGAGTTCCTTTATTTCCTCGCAAATATGGAATGGGCCATCGTGCC
TGTTTGCCGTGATCGTATCCGTGAGTACATCATGGACAACCGCCCTGTTAGCTCACTGTGGGGGGATGTTGTCCAGATCAGAA
CGGTAGATCACAACTTCTATACCACCGGCATTGAATTTCGTGAATACGAAGAAGATACAGAGCCAGGCCAGCTTAACATGCTT
AAAGAAGCCGATTTTGAATACCTTCTGACGCAGAGTTTTTCTTGCCTCTCTGAATCTTCAGCTAAAACGTTTCTGACGCATCAG
GAAAAATCTTTGCAGGAAACGCGCGACCGTGCGCAAAGCCAGCTGGCACAGCTTGGTACCGCGCTCGATATGCTGACGTCC
AGAGAGTTCGTGATGGGCTACCATCATGGAACCGTGCATGTCTGGGATAATGACCAAAACGCGGTACAGCGCAAAGCGCGTC
GTGTGAAGGTTATGCTAACCGGCTGTGGCGTGGTTGGCGGGACTCTCAGCCTGGCCTCTGAGGCTGCATATTATGCGAGACT
GCCTGGCAACCAGAAATGGGCGCCGCGCCCGGTTCCGATAAACTCATGGAACTTCCTGCACTTCAGCCCGTTCCACAATTTTA
TGCGTGGCAAGCCTGACAATAACCCGTGGGGGCCAGCGCTGACCATGTTCCGCACGATCAGCGGTACGCCACTCTATTTTAAT
TTCCATGTGACCCCGCTTGAAGAACTTTCCTACGGTAAACGCCCGCTGGGCCATGCGTTAATAACGGGTATGTCGGGGGAAG
GTAAAACCACGCTGCTTAACTTCCTGCTGGCGCAGTCAATGAAGTACAACCCGCGGCTTTTTGTTTATGACCGTGACCGCGGT
ATGGAGCCGTTCATTCGAAGCGTTGGTGGCTACTATAAAGTTCTGCAACAGGGTATGCCGTCCGGGTTTGCCCCGCTTCAGAT
TGAACCGACCAAACGCAATATTGCCCTCATTAAAAACCTGTTCCGCATTTGTGTGGAAACCACCAATAACGGGCCTATCAGCG
CAACGATGGCTACCGAACTGGCTGAAGGCGTTGATGCGGTTATGGGGGAAGGCTCACTTATTCCACGCGAGGCGCGCACCG
TTACTATCCTGGACGGGTACGTGAATGAAGTTGTGGAAAATGGCGTATCACTGAAAGGGCTGCTGCGCGAATGGACGCGCGA
AGGCCAGTATGGCTGGCTGTTTGACAATGATAAAGACAGCCTGGATCTCAGCGCGAATGATATTTTTGGCTTCGATTTATCCGA
GTTTATCGCAGCCAAAGAGGAAGTATCCAGCCCGGCCCGTACTCCGCTCATGATGTACCTTCTGTACCGGGTACGTGACTCCAT
CGACGGCAAACGCCGCGTCATTCAGTGCTTTGACGAGTTCCACGCCTACCTTGACGATCCGGTTATCGAGCGTGAAGTTAAG
CGTGGTATCAAAACTGACCGTAAGAAAGACGCTATCTATGTGTTTGCCACGCAGGAGCCGAACGATGCGCTGTCCAGCCGTAT
TGGCCGCACGATCATGTCGCAGACCGTCACAAAAATCTGCCTGCGCGATCCGGAAGCTATCCGAGAGGATTATGCCTTCCTTA
CTGATGCTGAATACGACGCGCTGATGTCGATTACCGAACACTCCAGACAGTTCCTGGTTAAACAAGGGCAACAGTCTGCGATT
GCTTCTTTCAATCTCTACCCTCGCAACAGCGACGATATTGATGCAGATATTAAGACAATGGACAACGTTCTTAGCGTGTTGTCC
GGTGAACCACAAAACGCCGAAATTGCGCATGAGCTGGTTGAACGGCTCGGTAATGACCCTGAAGTATGGCTCAAAGAATACT
GGCGCCTGACGGCTTAACAACGAGGCAAAACACCATGAAAAAAACACTGACGGCAGTATTGCTGACCACCGGCCTGATACT
GGGAGGCGCGCAAAGCGCTTCCGCAGGCATCATCGTGACCAACCCTACTGAGCTGGCTAAACAGGTCGAGCAGCTTCAGCA
AATGGCGCAGCAGCTGGAGCAGCTTAAAAGCCAGCTGCAAACGCAGAAAAATATGTATGAGTCGATGGCAAAGACAACCAA
CCTGGGCGATCTGCTGGGGACGTCTACCAGCACGCTGGCAAATAATTTGCCGGACAACTGGAAGGAGATCTACAGCGACGC
CATGAACTCCAGTTCTTCCGTCACGCCTTCAGTTAACAGCATGATGGGCCAGTTTAATGCGGAAGTTGACGACATGACGCCCA
GCGAAGCAATTACCTACATGAACAAAAAGCTGGCTGAAAAAGGCGCTTATGACCGTGTTATGGCAGAAAAAGCCTACAACAA
CCAGATGCAGGAACTAACCGATATGCAGGAGCTGACGGAGCAGATTAAAACGACTCCAGACCTGAAATCGATTGCTGACTTA
CAGGCCCGTATCCAGACGTCACAGGGTGCTATTCAGGGTGAGCAGGCGAAGCTGAATCTGATGAACATGTTGCAGCAGTCAC
AGGACAAGCTATTACGTGCGCAGAAAGAACGTGCCACCCACAATTTTGTTTTTGGAACCGGCGGGGACGTTACCGCGTCACC
TTCAATTAACTGAGGTAATTATGAAAAAACTACTGCTTGTTATCCCTTTCCTCCTAGTGGCCTGCGATGCCTCGCATGACGTGGA
GTGGTACAAAAAACATGAGAAAGAGCGCAAGGCAACAATTCAGGAATGCAAGAAAGACGCGGATGAACTTCAGAAACCTG
ATTGCAAAAACGCGCGCGAAGCCGATCGTCAGCTGTTTGTGTTCGGCAAAAAAGACGGCGAAATCAATTCACCGAAAATTTA
GGAGTAAGGAGGCAATATGGCATTCACCCTAGTCGCAGACATTTTCGCAAAAGTAGACGGGGCGATTACGTCAATGGTGAGC
GCCAATGTTGCCACCATTATCTCTGATGTAACGCCTCTGATTGCCACCTGTCTGACAATCAAGCTGATGGTTCAGGGGATGTAC
TCAGCGTTTAATCCGGGGGCGGGCGACAGCCTGAGTTCGCTGATTAAAGAGTATCTTTCCATAGCCCTTATCCTGAGCTTTGC
AACGGCGGGCGGCTGGTATCAACAGGAACTGGTCAACGTGGCGCTTCACCTGCCGGATGATTTTGCCGGGATACTGTCTGCC
CCTAATAAAGTCGGTGCAAGTGGCGTACCGGCGATTATTGATAGCGGTATTGAAAAAGGTATCAAGATCGTCAACACCGCATG
GGAAGCCGCAGACGTGTTTTCATCGAGCGGCCTGGCCGCGTATGCCATTGGCGGCATTATGATGATTGCTACCGTTGTGCTGG
GCGGCCTCGGTGCGGGCTTTGTGATCATGGCTAAGATCCTTCTGGCCGTTACGCTTTGTTTTGGCCCGATTGCAATCTTCTGCC
TGCTGTGGGGAGCGACAAAAAACATCTTTGCTCGCTGGCTGGCGTCGGTCATTAACTATGGCCTTGTCGTCGTCATTCTTGCG

CTCGTGTTTGGTTTCATCATGCAGATGTTCGACAACCTCCTGTCCTCGATGAACTCTGATGCCGCTTACTCATCAATCACTGGTT
CTATCTCCGCCTTATTACTGACGGTCATTTCCGTTTTCGTTCTGTTCCAGATTCCGCAAATTGCCGCCAGCTGGGGTAGCGGTAT
CAGCGCCGGAGTTGCTGACGCCGCACGCTCTACGGGTTCTTCCATGCAGGCGCTTGGCAATATGGGCAGCCACGGCATGTTT
GGCGGTAATGCGTTCAGAGGCGGTAACAGTGGCGGCGGCCAGCAATCGGCAGGTGGAGGAAGTGGCAGCAACAGCGGAG
GAAGCAGTGGTTCTAATTTAAGTGGTAAGGCAAGGGGCAGTCGCGGGAAGAAGGCTGCATAAAATTAAAACAAAAGTCGTT
GAAATTGCAATTTCGACGACTTATTATAATTAGTACGTTCAACAACCGATAATGGATGCCGTAATGCGCAGCTTATTGCTTATGG
GAGTTCTTCTGATTAGCGCCTGTTCCAGCGGGCATAAACCGCCACCGGAGCCGGACTGGAGCAACACCGTTCCAGTAAACAA
AACAATCCCGGTTGATACGCAAGGTGGTGCAAATGAAAGCTAATAAAAAAACAGGGCTTACACGTGAAGCCATTAAAGAGTT
CAACGAAAGCCGTAAAGGGCTTGAAGTTGATCTGATGGATGAAGTGCTGAAGTCCCGGCGTACCGCCTGGATGGTTGCCAC
CGGTTCAGCGGTGGTAACTGTTTTTGCACTCTCTTTAGTTGGTTACGTGGTGCATAAGTACAGCCAGCCAATCCCCGCACATCT
GCTAACGCTCAACGAGGCCACTCACGAAGTACAGCAGGTCAAGCTGACCCGCGACCAGACCTCTTATGGTGACGAAATTGAT
AAGTTCTGGCTGACACAATATGTCATTCACCGTGAGAGCTATGACTTCTATTCAGTTCAGGTCGACTATACGGCCGTTGGCTTA
ATGTCCACGCCGAACGTGGCAGAGTCTTACCAGAGCAAGTTCAAGGGCCGCAACGGTCTTGATAAGGTTCTGGGCGACAGT
GAAACGACCCGCGTGAAGATTAACTCTGTGATCCTCGATAAACCGCACGGCGTAGCAACGATACGCTTTACTACGGTTCGCCG
CGTGCGCAGCAATCCCGTTGATGATCAGCCGCAGCGCTGGATTGCCATTATGGGGTATGAATATAAATCGCTGGCGATGAATG
CTGAGCAGCGTTATGTCAACCCGCTGGGTTTCCGCGTGACGAGTTATCGCGTCAACCCTGAAGTTAACTGAGGGCTGCCCCAT
GAAAAAACTACTTCTTTCAGCAGTCGTTTTGTCAGTCCTGGGAGGCGCGGCCACTAACGTTATGGCGCTTGAGGTTGGCCGC
AATTCTCCTTATGACTATCGCATTAAAAGCGTTGTTTATAACCCTGTTAATGTGGTCAAAATTGACGCTATCGCCGGTGTGGCTA
CCCACATTGTTGTCGCGCCTGACGAAACCTATATCACTCATGCTTTTGGCGATTCTGAAAGCTGGACGTTTGCGCACAAAATG
AACCATTTTTTTGTGAAGCCGAAACAGGCCATGAGTGATACCAACCTGGTGATCGTCACCGATAAGCGCACCTATAACATCGT
CCTCCATTTCATCGGTGAAGAAACGAAGAAAAATGCAGACGGTACGGTATCAAAATCCTTTATTGAAACGCCGTGGGCTGTG
CGCCAGGCCGTTCTTCAGCTGACCTATGAATATCCGTTTGAGCAGCAGGAAAAAGCCAAAAGCGCGGCTGATAAAAAACGCA
TTACGCAGAAGCTGAAGCAGACGGCTTTTGCGGGGGCGAAGAACTATCAGTACGTAATGAGCGAACAGCCTGAAATGCGCA
GCATCCAGCCGGTTCACGTCTGGGATAACTACCGCTTTACCCGGTTTGAGTTTCCGGCCAATGCGGAGTTACCGCAGGTCTAC
ATGATTTCGGCCAGTGGCAAAGAAACGCTGCCTAACTCTCATGTTGTGGGTGAGAACCGCAACATCATCGAGGTGGAAACCG
TCGCTAAAGAGTGGCGTATTCGTCTGGGCGATAAAGTCGTTGGCGTTCGTAATAATAATTTCGCGCCGGGCGCCGGTGCGGTA
GCAACCGGTACGGCTTCCCCGGATGTGCGCAGGGTTCAAATTGGGGAGGATAACTGATGGCCCGTAAAAGTGTCGATGTAG
ATCAGGAACTCGATGAAAACACCGGAGACGGTGAATTCGAAAGCGAGCGTGGCGGATTTAAAGGCAGTAACCGCCGTTCG
GCTCCTGGTATGAAAGCCTTTGTCATACTGATGGCGCTGCTTGCTTTGGTATTCATCGGGATTACGGTCATGGGTAAAATTCGC
ACCCCGGCTAAAGCTGAAGCTGATAAAGACGGTGGTAAAGCGCAACAGGCCAATACACTGCCAAACTACAGCTTTAACAGC
GATCCTGATGTTAATAAACCTGCAACTGCGCAGAATAGCGCCACTGATGCCCGTGCTGTGCAGGCTGCCGCACAGGCAGATG
CAGATGCGGGCAGCAGCAATACCGCCGCGCGTACCTCTAATAAGCGTAAAGAACCTTCGCCTGAAGAACTGGCTATGCAGCG
TCGTCTGGGCGGCGAGCTGGCCCAGACTAATCAGGCGGCTACAAGCAATAGTCCCGGAGTGCAGCCCCAGGACAACGAAAC
AAGCGAAGGTAGTTCAGCACTCGCTAAAAACCTGACTCCTGCAAGGCTGAAGGCTAGCCGCGCTGGAGTCATGGCTAATCCC
AGCCTGACTGTTCCGAAAGGCAAAATGATCCCCTGTGGTACCGGCACCGAGCTGGATACCACTGTTCCGGGTCAGGTTTCCT
GCCGGGTTTCACAGGACGTTTACTCAGCTGATGGACTCGTTAGGCTGATTGATAAAGGCTCATGGGTTGACGGGCAGATTAC
CGGTGGTATCAAAGACGGCCAGGCGCGCGTGTTTGTTCTCTGGGAGCGTATCCGCAATGACCAGGACGGGACAATCGTTAAT
ATTGACAGTGCCGGAACGAACTCACTCGGCAGCGCGGGGATTCCGGGCCAGGTGGATACCCATATGTGGGAGCGTCTGCGT
GGTGCGATCATGATTTCGTTGTTCTCTGACACCTTAACGGCGCTGGTTAACCAGACGCAGAGTAATAACATTCAGTACAACAG
CACAGAAAACAGCGGTGAGCAGCTGGCGTCTGAAGCACTCCGCTCTTACATGTCTATCCCCCCTACCCTCTACGATCAGCAGG
GTGATGCGGTGAGCATTTTTGTTGCCCGCGACCTCGATTTCAGCGGCGTTTATACGCTCGCAGACAACTAAAAAAGTGGGCG
CTTAGCGCCCGCTTTTCTTCAGGAGTAATCATGACTGATGCAGCTTTCTATCAACTTGGCCCACTGCGCGAGTATTTAGAAGAT
CCTACTGTTTTTGAAATTCGCATTAACTGCTTTCAGGAAGTTATCTGTGATACGTTCAGCGGCCGCAGGGTTGTGCAGAACGC
GGCAATTACGGCAGATTTTATTAGGAACCTTGCTAAATCGTTGGTGAGCAGCAACAAGCTGACCATGCAGGCCATTAATGACG
TGATCCTGCCTGGCGGGATCAGGGGCGTTATCTGTCTGCCCCCTGCGGTGATTGACGGTACAACGGCCGTAGCGTTTCGTAA
GGATTTGGCGGCCGATAAAAATCTGGAGCAGCTGACCCGCGAGGGGATTTTCAGTGACTGCCGGAAGATTACCGGCAGCAA

GCAAAGCCTAACGGATGATGATTTTTTCCTTAAAGAGCTGCACAGCAGCGAAAAATGGCCCGCATTCCTGCAAACCGCCGTT
GAGAAGAAACGCACTATCGTGATCTGCGGTGAAACCGGGTCGGGGAAAACGGTACTCACGCGCGCGCTGTTAAAATCGCTA
CATAAAGACGAGCGTGTAATTATTTTAGAGGACGTTCACGAAGTCACGGTCGATCACGTTGTAGAAGCCGTTTATATGATGTAC
GGCGATGCAGGAAAGATCGGCCGCGTCAGCGCCACTGATGCCCTGCGAGCCTGTATGCGTCTGACACCGGGCCGTATCATCA
TGACTGAGCTTAGGGATGATGCTGCGTGGGATTATCTTAAAGCACTTAATACCGGCCATCCAGGCGGTGTTATGTCAACGCAC
GCTAACTCTGCGCGCGATGCCTTTAACCGTATTGGGCTGCTTATCAAGGCGACCCCTATCGGCCGTATGCTCGATATGAGCGAT
ATTATGCGAATGCTCTACTCCACCATTGACGTTGTGGTGCATATGGAAAAGCGGAAAATCAAAGAAATTTATTTTGACCCTGAA
TATAAAATGCAGTGTGTGAACGGGAGCCTGTAATGAAAAACTTAGCAACCTGGCTTCTGGCCGCAGCATTTACGACAGCCGC
CCTGCCCGCCTTTGCGGTGGAACCATCCGTTCAGGTTGGCTACTCGCCTGAAGGGGGGGTAAGCGGGAACCCCAGAAAATT
CCGCCATTCCGCATTGTGGAATTTTTTGGGGGGGTGGTCCGCGGCATGACGACCCGCCAAAGGCTACCGCATCTATCTGAGG
CGCTGTGGCGCGACGCTGAAACCCCGGGGAATTCCGCTACTCCAGTTCCAGGTCCGCAATAGTTCGTCCAGGTCGGGTGGC
GTACGCCGGAAACGCCGATTTTTCCGCAACCGTTTCTGCGTTGTCGGCAAGGTGGTCTCAGAGAGCGCTCAAGAAGCGGTT
TTTCTCGTATGTTTAGCTACGGTGGCCTAGGAGCCTGCGCGGCAGAAGCCAAATCGGCGGAAGATCGAGGACGATACGGGG
ATGGCTGCCAGTTACCTTCCTTACCGACCCGACCAATCCTATCTGCTGCCCCCTTCTCTGGGAGAGTGGCTACCTGAAGGGCAT
CTTGCCTACTTCATCAGCGAGACTGTCGATACGCTGGACTTGAGCGCATTCCACGCCCGGTATGCCGGCGGTGGTCCGGGCA
ATCAGCCGTTTCATCCGGCGATGATGGTCAAAGTGCTGATCTATGGTTACGCGAGCGGCGTCTTCTCTTCGCGCAAACTAGCC
AGGAAGCTGTACGAGGATGTCGCGTTGCGTGTGCTGGCCGCTGGAAACTTCCCGGCCCACCGCACGCTGAGTGACTTCCGT
GCCCTACACCTGACCGAGCTTGAGAATTTGTTCGTTCAGGTGGTGCAACTGGCGCGCGAATGTGGGCTGGTGAAGCTTGGC
ACGATCGCGGTCGACGGCACCAAGGTAAAAGCCAACGCCAGCCGCCACAAGGCGATGAGCTATAAGCGCATGAAGCCGGCC
GAGGACGAATTGCATTGCGAGATCAAGGCGCTGCTTGATCGCGCCAAGGCTACCGACGACCAGGAGCGTAACGAGCCGGA
GCTGGACATTCCTGCCGAGATTTCTCGCCGCGAGAAGCGCCTGGAGGCGATCCAGGCGGCAAAGGCGCGCCTGGAAGGGG
TCGGTTCCGGCTGAGGGCGAAATGACACCCTAAGCTTTCGGTTCCTTGGGCCAAAGATATTCGCCAGTCAGTAGAATGTGCG
CCCAGCCCAATGGGGATATGTGGGGAAGAAATTCAGGGGGAACATCCAACCCTTCGTTCCGCCGCTCCGTGACGGCATGACC
AAGATGGACGGTATTCCAGTAAATGATCACCGCAGTCAATAAATTGAGCCCAGCGATTCGGTAGTGCTGCCCCTCTGTCGTGC
GATCGCGAATTTCCCCCTGCCTCCCGATACGGAGCGCATTTTTGAGCGCATGGTGGGCCTCTCCCTTGTTAAGACCGATCTGA
GCACGCCGCTGCATGTCCGTATCCAGGATCCACTCAATAATGAAAAGGGTCCGTTCAATACGACCAACTTCACGAAGCGCAAC
TGCAAGGTTGTTTTGTCGTGGGTAAGAAGCGAGCTTGCGCAGGAGTTGGCTGGGCCTGATTTTGCCAGCGGTCATCGTCGC
GGCACAACGGAAAATATCAGGCCAGTTCGCAACGATAAGATCCTCCCGGGCTTTTCCACCTACCAACTTGCGTAACTCCCTGG
GGGTCGTATCGGGATTAAATACGTACAACCGCTTCGATGGCAGATCCCTGATTCGCAGAACGAGATTGTAGCCGAGCAGGCTA
CTGGCTCCGAACAAATGGTCGGTGAATCCTGCTGTATCGGCATACTGTTCGCGAACATGGCGACCGACCTCGTTCATCAGTAG
TCCATCGAGAATATACGGTGCCTCGCTCACGGTCGCCGGGATCGACTGACAAGCGAATGGCGCGAACTGGTCGCTTACGTGA
GTATACGCTTTGAGGCCGGGAACAGAACCATATTTGGCATTGACCATGTTCATGGCTTCGCCATGCCGCGCTGTCGGGAAAAA
CTGACCATCGCTCGATGCTGACGTGCCCATCCCCCAGACGCGTGACATCGGCAGTTTACCCTGCGCGGCCACCACAATTGCCA
ATGCCTGGTTCATGGCTTCGCTTTCAACATGCCAGCGGGCAAGGCGTGAGAGCTGCCAGTAATCATGCGTGTTTGTAGCTTCC
GCCATCTTACGCAGGCCCAGATTGAGCCCTTCAGCGAGCAGGACGTTGAGCAGACCGATCCGGTCGCGACATGGAGCCCCG
GTTCTCAGATGGGTAAACGCATCTGTGAAACCAAGGGCTGCATCAACTTCAAGCAGCATGTCGGTAATCCGAACGGACGGCA
TTCGGCGATACAGATCCAGTATGAGTGCCTCGGCACCATCCGGCACGTCTGCTGTCAACCTGTCGATCCGCAACGTTCCATCTT
CTATGCTACCGTGCGGAATAGTGCCGTTACGGGCAGCCCGGGCCAGCCGCTTAAGAGCGATCGTGAGTCGCGCCTTTCTGTC
TGCCAGCCAATCCTGTGGGTTGGAAGGCACGGCCAGTTTTGCATTTTCCTGCGCCGCGATCATCGGCACCAGTACCTGCTTGA
GGTCACCATAGCGGCGCGAATGAGCGAGCCAGACATCTCCGGAACGAAAAGCATCCCGGAGGTGAAAGAGTACCGCCACTT
CCCAAAGACGGGTATCTCCTTTTTCCTGAGCTCGTAAATGACGGTTCCATTTGGAGCTGGGCCGCAGGAAACGCCTTTCTGG
CGATGCAACACCTTTCATCTCTCCGATCGACAAAGCTGCTGCTACCAATGGTCCGGCGACCGGCGCGGCTTCGAGCTTCAGAC
AGCGCAACATGCGGGGCGCATAACGACGAAAGCGATGGTATCCCTGCCCGACATATGCAAGAGGCTCATCGGCTAGCGTGTT
GCTGAGTTGAGTCCCTGTCGCTACCAGTTGAGCGAGCCGGTCCCATGCAACCGAACTGGCGACAGCCATCTCCAGCGGGGT
TCCGTCACTGCGGGCCTCAAGCAACGAAGCTCCCAGCGCGGTGAAGGTACGGATCGTATCCGTGAGTGTGGCTTTAGAGCC
GGAAATTGTTTCGTCATGCTGGCGCTTCGCTTCCCGCCAGGTTTTTCCTACGATCCTGTCATGGGTTTCGACTATGGCATCAGC

AATCGCCGCTTCCCACTCCACAACACAGACGGCAAGGATCGCCCAGCGGCGGTCCGAAGTGATGTCACGCAAACCGTCGGT
GAAGTAGCGTTCACCCTGCCGACGCAGCCGGGCAATGCGATGGGCAGGTATGCTGGCCAAAGCACTATGATTGATATTCAGG
GTACGCAGAAATTCGAGCCTGTCGAGCAAACGGTTAGCAGCAGCCGAGTTGTTACCAACCTCGAAGTTGCGAAGCCAGATG
AAACGACTGATATTGCCGGCGAGCATTTCACTCAGAAGTTTGTCCAGGTGATCGCGAACATCCGCTGTTAAATTTTCCACAATC
CGCGTTTCAATCCGCCGCTCAGCGGCGACCAGAGCATCCGCGCACAAGCGCTCGATTGTCGATACTGCGGGCAGAATGGTG
GAAGTTTCCCGACACCGCACAATAAAACGATGAGCAAGATCCTCGTTTGATCTGGCATCTTCGGCCTGGCCGAAAGTCCACTC
CCGCAGATCACGGGCACCACGGCCCGTGAAGGTCTTGTAGCCGTAAATTTCGCGCAGCGTGTCCATGTGCTGCTGACGGGTT
TGGCGCCGTGTGGCATAAGTGAGAAGCGCATCAGCCGGAACTCCAAGCTGAGCACCGACGAAGGAAAGGACTTCACGCGG
GATCATCTCACCAGGAGCCAGTGCACGGCCCGGATATCGTAAGGCACAAAGTTGCAGGGCAAAGCCAATCCTGTTTTCCGGT
CTGCGGCGCTGCCTAATGTTTTCCAGGTCATCATCGCCCAGCGTGTAGAACTTCAGTAGCGACAGTTCGTCCGTGGGCAGATC
GAACAGCGCTGCTCGCTGCCGTTCGGTGAAAATATGGCGTCGTGACATACAAATTCGTCCCTTTTGAAGTATAGTCTGTTTTG
GACAACAGCCAGCCCATATAAATCAGGGCGTTCCGATACAAAAATCCAGGAGGGTTCAATTGGGACATCGTGCCGCCATTTAC
TGCCGGGTTTCAACAGCGGATCAGTCTTGTGAACGCCAGGAATTTGATCTGCGAGCCTTCGCCGGCCGTGCCGGCTACGACG
TGGTGGGAATATTTAAGGAAACAGGTTCAGGAACTAAACTCGACCGGGCCGAGCGAAAGAAAGTCCTGGCGCTTGCCCAGT
CCAGACAAATTGATGCAATCCTGGTCACTGAGCTTTCCCGGTGGGGGCGCTCGACGCTCGATCTGCTCAATACGCTACGTGAA
CTGGAGAACTGGAAGGTTTCCGTGATAGCCATGAATGGAATGGCGTTCGATCTTTCGTCGCCGTATGGACGAATGCTGGCGA
CGTTTCTTTCCGGCATTGCGGAGTTTGAGCGGGATCTCATCAGCGAGCGGGTCAAGTCAGGCCTTGCTGTTGCGAAGGCAC
GTGGTAAGAGGCTTGGTCGTCAGGCCGGAGTGCGACCAAAATCAGACCGACTTTTGCCTAAGGTGGTTGCGATGAGGGCCG
AGGGACGCAGCTATCGCTGGATCGCACGCGAGCTCGGTATCAGCAAGAATACCGTCGCTGACATCGTGCAACGACACAGAG
CTAACGCTTAGGGTGTCATTTCGCCCTCAGCCGGAACCGACCCCTGGAAGCGCGCCAGCGTGAAGCGGACCAGGCCCGGG
GGCGCAGCGAAGACGATGGCCGCCGGCCTCGCCATCCGGATGGCTCGGACAAGGGCGGTGGCTCGTACAAACGCGAGTTT
GGTGTGCCGGATGACCGTGATCAGGAAAGCTTCACCGATCCGGACAGCCGGATCATGAAACACGCCGGTGGTGGCTCCGAG
CAGAGCTACAACGGGTACACAGCGGTCGATGCCGAGCACCAGATCATCGTGGCGGCGGAGTTGACCAACTGCGCCGCGGAC
AGTCAGGCGCTGCTGGGCATGCTGGCAGCAGTTCAGGCCAACACCGGAGAAATGCCGGCCCAGACGCTGGCGGATGCGGG
ATTCCGTAGTGAGGCTGTTCTGGCAAAGGTCGCCGATCACCACGGCGATGTCATCGTTGCCCTCGGCCGCGAGGGACGTGAA
GATGCCAAGGTCAATGCCAAGACCCATCCGCATACGGCGGCGATTGCGGCGAAATTGAAAACGGAGCAAGGCGATGCAGCT
TACCGCCGGCGCAAGTCGATCGTGGAGGCTCCGAATGGTTGGATCAAGGCGGTGATGGGATTGCGTCAGTTCAGCATGAGG
GGCCTGGACAAGGTGCAAGCCGAGTGGAAGCTCGTCTGCATGGCGCTAAATCTGAGGCGAATGGCGTATCTGTGAGGGCGA
AGGTTAAATGGGGCGGCTCAAATGCACCCCAGTCGTCATAACACGCCGCGCGCCGCAAGATTGGTATCCTTGGCGCCGACGC
CTTGCCAATTGCAGAGAGCGCCGCCGCCATCGTCAGTGCTCTACAGAAAACCGGTCACACGGCTCTGCCGCGCAGACTCCTA
GCCTAAATGTGACAGTGGTTGGTAATCCATGCCGCCCGCAAGGGCGGCGGTGGTGGGCCAATAGATGATTTTCAGAGCCTTA
CTGCCCGTTGACGCCCAATCCCTCGAGCGCGAGTCTAGCCGCAGCGGCGATGACGGCCTCGCTGTGCTTGTCATCCTTGTTAG
GCGCCCGGGTGTAGACGGCCAACACAATAGGTGCGCGCCCAGTGGGCCAGACGACGGCATAGTCATTTGCCGTGCCATACA
CTCCGCAGGTTCCGGTTTTGTCTCCGACTGCCCAGTCTGCCGGCACCGCCGCGCGGATGCGGTGGTTGCCGGTCGTGTTTCC
CTTTAGCCAATCAACAAACTGCTGCCGCTGCGGCGCAGCCAGTGCAGAGCCCAGTGTCAGTTTTTGTAAGCTTTCCGTCACG
GCGCGCGGCGATGAGGTATCGCGCGCATCGCCTGGGATGGCGGAGTTCAGCTCCAGCTCCCAGCGGTCCAGACGGAACGTG
GTATCGCCGATAGAGCGCATGAAGGCCGTCAGCCCGGCCGGGCCGCCCAACTCCTTCAGCAACAAATTGGCGGCGGCGTTAT
CACTGTATTGCACGGCGGCCGCGGACAGCTCCGCCACCGTCATGCCTGTTGTCAGATATTTTTCCGAGATGGGTGACCACGG
AACCAGCGCATTTTTGCCGTAACGGATGGGTGTGTCCAGCAAGCCGGCCTGCTGCTGGCTGCGAGCCAGCACAGCGGCAGC
AAGAAAGCCCTTGAATGAGCTGCACAGTGGGAAGCGCTCCTCAGCGCGGTAACTTACAGTTGCGCCTGAGCCGGTATCCATC
GCGTACACACCGATGGAGCCGCCAAAGTCCTGTTCGAGTTTAGCGAATGGTTCCGCGACGAGGTTGGTCAGCGCGGTGGCA
GAAAAGCCAGCCAGCGGCCATGAGAGACAAGACAGCAGAACTAGACGGCGATACAGTGACATCAACGATATTCCTTGTTTG
AAGGTGGAGTTACGGACGGCCTCAGGAAGTCCTGGCCAAGCCCCGACTATTGGGGCGCGAAGATAGCACCGAGGGTACGC
CGGTGTCAACACGGGGTCTGACGCTCAGTGGAACGAAAACTCACGTTAAGGGATTTTGGTCATGAGATTATCAAAAAGGATC
TTCACCTAGATCCTTTTAAATTAAAAATGAAGTTTTAAATCAATCTAAAGTATATATGAGTAAACTTGGTCTGACAGTTACCAATG
CTTAATCAGTGAGGCACCTATCTCAGCGATCTGTCTATTTCGTTCATCCATAGTTGCCTGACTCCCCGTCGTGTAGATAACTACG

ATACGGGAGGGCTTACCATCTGGCCCCAGTGCTGCAATGATACCGCGAGACCCACGCTCACCGGCTCCAGATTTATCAGCAAT
AAACCAGCCAGCCGGAAGGGCCGAGCGCAGAAGTGGTCCTGCAACTTTATCCGCCTCCATCCAGTCTATTAATTGTTGCCGG
GAAGCTAGAGTAAGTAGTTCGCCAGTTAATAGTTTGCGCAACGTTGTTGCCATTGCTGCAGGCATCGTGGTGTCACGCTCGTC
GTTTGGTATGGCTTCATTCAGCTCCGGTTCCCAACGATCAAGGCGAGTTACATGATCCCCCATGTTGTGCAAAAAAGCGGTTA
GCTCCTTCGGTCCTCCGATCGTTGTCAGAAGTAAGTTGGCAGCAGTGTTATCACTCATGGTTATGGCAGCACTGCATAATTCTC
TTACTGTCATGCCATCCGTAAGATGCTTTTCTGTGACTGGTGAGTACTCAACCAAGTCATTCTGAGAATAGTGTATGCGGCGAC
CGAGTTGCTCTTGCCCGGCGTCAACACGGGATAATACCGCACCACATAGCAGAACTTTAAAAGTGCTCATCATTGGAAAACGT
TCTTCGGGGCGAAAACTCTCAAGGATCTTACCGCTGTTGAGATCCAGTTCGATGTAACCCACTCGTGCACCCAACTGATCTTC
AGCATCTTTTACTTTCACCAGCGTTTCTGGGTGAGCAAAAACAGGAAGGCAAAATGCCGCAAAAAAGGGAATAAGGGCGAC
ACGAAAATGTTGAATACTCATACTCTTCCTTTTTCAATATTATTGAAGCATTTACCAGGGTTATTGTCTCATGAGCGGATACATAT
TTGAATGTATTTAGAAAAATAAACAAATAGGGGTTCCGCGCACATTTCCCCGAAAAGTGCCACCTGACGTCTAAGAAACCATT
ATTATCATGACATTAACCTATAAAAATAGGCGTATCACGAGGCCCTTTCGTCTTCAAGAATTTTATAAACCGTGGAGCGGGCAA
TACTGAGCTGATGAGCAATTTCCGTTGCACCAGTGCCCTTCTGATGAAGCGTCAGCACGACGTTCCTGTCCACGGTACGCCTG
CGGCCAAATTTGATTCCTTTCAGCTTTGCTTCCTGTCGGCCCTCATTCGTGCGTTCTAGGATCCTCCGGCGTTCAGCCTGTGCC
ACAGCCGACAGGATGGTGACCACCATTTGCCCCATATCACCGTCGGTACGGCACTGTTGCAAAGTTAGCGATGAGGCAGCCT
TTTGTCTTATTCAAAGGCCTTACATTTCAAAAACTCTGCTTACCAGGCGCATTTCGCCCAGGGGATCACCATAATAAAATGCTG
AGGCCTGGCCTTTGCGTAGTGCACGCATCACCTCAATACCTTTGATGGTGGCGTAAGCCGTCTTCATGGATTTAAATCCCAGCG
TGGCGCCGATTATCCGTTTCAGTTTGCCATGATCGCATTCAATCACGTTGTTCCGGTACTTAATCTGTCGGTGTTCAACGTCAGA
CGGGCACCGGCCTTCGCGTTTGAGCAGAGCAAGCGCGCGACCATAGGCGGGCGCTTTATCCGTGTTGATGAATCGCGGGAT
CTGCCACTTCTTCACGTTGTTGAGGATTTTACCCAGAAACCGGTATGCAGCTTTGCTGTTACGACGGGAGGAGAGATAAAAAT
CGACAGTGCGGCCCCGGCTGTCGACGGCCCGGTACAGATACGCCCAGCGGCCATTGACCTTCACGTAGGTTTCATCCATGTG
CCACGGGCAAAGATCGGAAGGGTTACGCCAGTACCAGCGCAGCCGTTTTTCCATTTCAGGCGCATAACGCTGAACCCAGCG
GTAAATCGTGGAGTGATCGACATTCACTCCGCGTTCAGCCAGCATCTCCTGCAGCTCACGGTAACTGATGCCGTATTTGCAGTA
CCAGCGTACGGCCCACAGAATGATGTCACGCTGAAAATGCCGGCCTTTGAATGGGTTCATGTGCAGCTCCATCAGCAAAAGG
GGATGATAAGTTTATCACCACCGACTATTTGCAACAGTGCCGGTCGCCGGGAGTCAGCAGATCGACGTCAACGCCGAGCAGC
GATTTCAGTTCTTCTTCCAAATCGCCCAAGTCCAACAACGTGGCACCGGGCAGCGCATCGACCAACAGGTCGAGGTCGCTGC
CATCCCGGTCGGTGCCATGCAGCACCGAGCCGAAGACGCGCGGGTTCGCGGCGCGAAAGCGGCCTACCGCTTCACGCACTG
CGCTTCGCTTCATGTCAAGCACAACAGACGGTCGCATGCGCATCCTTTCTTATCGAAACTCGTTGAGATGATATGCAATCAAGA
ATAGAATTTCAAGAACTCACAAAGTAACGCGGTGGTTAATATCCTGTACCCACGGATTGCCCTTAGCGCTGCCTATATCGGCTA
AAGCACTCCGGTAGCTTGATTCACCCCACGGCCACGGCAGGATCTTGGCCGTCGCAAGCGCCAGGGGAAAATCTTCAGCTG
CAAGCCTGAGTGATTTCATGTGCGTGTAATCCATCGCCCAGATGATTTTTGTGAAGAAGAACTCGCGCTCGTTCATGTCCGGG
CGCGCGTCCTGGCCCTCCGTGCCAACGGCCAGCAAGTAATCGGCCTGAATTGGCAGTATCAGCGCGCGTAGTAAGTCATGGA
TCGCCGGTTGCGGCAGTCTGCGCGCCAGATTAATTACCCGGTCGAACTTCAGTTTATCCTGCTTGCGCTTGTCGGTCTGCTCCA
TCAGATTTCATGGCCCCCTTCTTCATGCTCATGCTCATGGGTGTGTTCTTTTCCGGTATGGCTCTGTTCCGCCTGAGACGTCTGC
GGCATGGCGTAATCGTCGTAAATGCTGCTGTCAAAGTCGTAGTCTGATGCTTCGGCATAATGCTCGTAATCGGCATACTCCTGC
GCGCTCCACTGCTGATCGTCGGCAGCAGCATAATCATGGGCCAGCTCTGCATCATTTTGCTGTGCTTCATGACGCCGCAGGCC
AACGGAATCATCCATAGGGTTCTGCTTAAGATGAAAGGCGTCCTCTGCGTTGCTCACCGGCTGATAATCAGTGCCGGTTGTCA
TGTTATGTTCATCGGGTTTCTGGTTAAACGCCATGCTTTCCCCCGTGGCTTCTGGCAGACCTTTTTCAGCTGATCGGGTTTCTA
AACTGGTATCGCGGCCAATATCCTTAAACCTGGCCTCAAGCCCAAAGAAACGGTCAATTTCTGCGGCCGTGGTTTTCGGGCTG
TCGCGGCTCACGCTCGATGCCAAAGATTTTTATCGTCGGTAAAAATTTCCACCTCATGACGCGCACGCGAAATACCAACATAA
AAAACGTCCTTAGAAGTGGTAAGCGATTTGGTATCTATGTTGAACAACACGCGATCACAGGTAAGCCCTTGGGATTTGTGGAC
GGTGGTTGCATAAGCATAGGAAAGATAAGAAGCCTGTTTTTTGTCCAGCTCAACCGTGCGCCCTTTTTTGTCCTCAAGCGTCA
GTTTTTCACCCTCCACGGTTTTCACCGTGAAGCGGTCGCCGTTGGCAACGTCCAGCGTTTTATCGTTACGCGTTACCATAACCT
TATCGCCCGGCGCCAGTTCGGCGCTGACTGCCTGGTATACAGACAGCTTGGTGTGTGTACGCGGGCTGAAAGCGATCTGCTC
ACCGCTGCTGCTTTCAACCGTCAATTTGTTGCCCGGCCCGGTATCAAGAACCTGGTAAGACTCGCCCCGCTTCATACCATTTTT
GTAATCCTGTTCGGGGATAATGATTTGCCCTTTACTGAAATAACGGCTGTCGCGGCGTTCCGCCTGTGTCGAATCCACGCGGTC

AAGTAGCGTGAACGTTTCGCCGGTTCCGGCAAGCCCCAGATTGCCCCGGATGTAGTCATTGAGGGTTTTGCGTGAGGCGTTC
GTACCAGAGATTATCAGGGTGGCATCCTGTTGTTCTGAGGACAGAGACAGGTAGCGATCGGCAAGTTGAGCGAGTCGGGGC
GCTTCTTCCTTCAGTTCGTTCACGCCGGTGATATTTTTCAGGGCGCGCGCGGCATTACCTTCAGCGGCATACTTAACCGCCTCA
AGCAAAACTTCATTCTTCTGTCGCTGAATGTCTTTCATGTAGCTGGTCTGCATATCTGCTTTAATCAGCTGCTCAAAAGGCTTAC
CGGCTTCTACCGCTTTCGTCTGTGACGTATCCCCCAGGAATACCGCGCGAGCGTTATGCTTCTCGATCACCTCCATCAGCTGTT
TCATCTGTCGGGCGGGTATAACCCCGGCTTCATCAATGAATACGACTGATTTTTCATCCAGCTTTTTATCCTTCGCTTTGAGGAA
AGCGGCAACGGTGCGGGCCGGTAATCCATCATCTTCAAGCGCTTTTTTCTGTGTCCCATAGGGGGCCAGCGCCGTGACCTTC
AGCCCTTGTGACTCCAGCAGCTCTTTAGCGGCCATCGTCATATAGCTTTTACCGGTACCGGCGTAACCATGTGCGGCCACAAA
CCGATCTTTGCTCGTCACAATTTCTGTAACCGCGCGCATCTGCTCCTTCTTGAGGGTTTTCCCGGCAAGCAGCTGGCCTGCAAT
CTCTGCGGTCAGCTGTCGCGGCATCTGCCCCCGGCCGCGTGATTCGATAGTCAGAATGGAACGCTCAAGGCGAATACCCTCC
ACGGTAGTGACGCGGTGGCTGGTCTTTTTAAGCCTGCCGTTTTTAATACCATCATCTACCGCAAAACGGGCTTTATCCGCACGC
ATCCCGCTATTCGTCAGCGAGTCGATCCACTCTTTGCGCGTCAGAGTTTCGGCCATAACTGAAGCACCGACCTTCAGAGTTGA
TTGATACCGGGCTTCGCCCTCGATGATGGCGCCCTTCTGTACCGCCTTCAGGTACGCTTTTTCAACATCGGCTATTGTGGCATG
GCCCAGCACCTGCTTATTAGCGATTTGAATCAGCTTCTGGCGTTCAAAGCTGGCATCGCGCTCTGACAGCGACTTAACTGCAA
ACTGGATAGCCCGGTCAGCTTTAACCTCCGGGCTGGTAAAATCCGGGGCCATGTTGCGCGCTATATCAGCCTCCAGAGGTTTA
CCGTGTCCCTGCCATTCACGGTTATCAAAATCAATGCCGAGCGTTTTGGCGCGGCTGGCCCATTCCTGGTGAATTTCTTCACG
GGAATGCTCTGTTTTCTTTTCACGCGTAGCCATCGAGACGCGGCTTTTCGTCTGAGCATCGGCGGTTTCCCGCGTCAGACCCA
TTGCAGCGAGTCCCTTTTCAATTTGCTCCGACCGGCGGGAAAAAGCGCGAATCTGTTCATCTGAAAAATGGGCCATATCGAAC
GTGTTATTTTTGCTGTTGTAACGCAGCTCATAACCGGCTTTGGTCAACTCCAACGCCAGCTCCTGTTTGTAAACATCGCCCAGG
TGCATTTTGTTACGCATCAGCTCATCATTTTTGAGCGCGCGCCACTGGCCGTCCTCGCGCTGGGTCATGTTCATGACAAAAGC
GTGTGTGTGCAAATCAGGATCTAGCGCCCTGGAAGTTTCGTGGCGGAAAGTAGCGACGACAAGGTTATTGGTATTCTGGGTT
ACTGATTTCCCCTGGCGAGTCGTCCGGGCCTGCGCGAGTTTTTCAGCTTCACGCACAGCAGCGGCAACAGCTTTTTCATGAG
CCTCGATAATGGTTTTATCGCCGTGTATCAGCGCCTGCATGGATACCCCTTTAGGCGCTGAAAACGTCAGGTCGTAGCCCAGAC
GCTCTTTTTTGGCATCACCCACGTGTCGCTGCATATGCGTGAAGGTATCTATCTCTCCGACAAGCAGCTCTTTAAACCGGGCTG
ATTCAACGTCCCCGGATAAGCCGAGGGCTTCAGCTCCGGTTCCCTGCCAGGACGTGAATGATGAATCCTTACTGTAGTAATCA
TCCTTTGCATCAGAGTAGTAGCCCACAACGCTAGTGACGTTCTGGCGGGTAATCGTGGTTATATCAAGCATCAGATCTCCCTCA
GTTCAATGCCAGGAACAGGGTTTTTGCGATGGTATTTAACGTGTTTAGCCTTGAACTTAGCGACGGGCATATCACCAGGCAAC
GCCAGATAGCCGGTGAGGTTTGGCAACATTGATATTTCGGTAGGCGTTACGGCACGAACAACTTTAACGTCGCGGCGTTTAC
GGACAATCCAGGGCTTCTGAGGATCGGATTCTTTACGCTCAACTTCGCCTTCTATCTCACCGAGTGAGCGCGACATTTGATCC
AACGTTTCATCACCGAGACGGCTGCCGCCCAGCACGATGTTAGAACGCATGTTAGCCAGAATTGTCTGAGCCATATCCCGACC
ATAAACCTTAACCAGCTGAGAATAGGTTTGATAGCCAGCATAAACACACAGACCGCTTTTACGCCCTTTGGTCAGTGCATCGTT
GAGGTTTGGCAGAAACTGGAGTGATTCCAGCTCGTCAATAAATACATTAATGCGGCTTTCTTTTTCACCCATACCCAGCACGAT
AGAAAAAATCGAATCCAGCCAGCAGGAAATTAGCGGATTAAGTGACCTTTTCATTTCTTCCTGCCAGGTGATAAACAGGGTTC
CCGGCTTTCCATCATCAAGCCAGTCACGCAGGGAAAAATTACCTTCCGGCATTTTCAAATGTGGGGCAAGATTCTTACTGAGA
ACAAATCGCGCGCTTCCAACTGCTTTTTCAGACCCGGAAAAAATAGCTTCGGCAGGCGTCCCCATTAAAAATTCTTTTAATTTT
TTCTGGTCAACGTTACAGGCCCAGTGAATAACTTCTTCCATAGTTACTGTGCTGTATAGGCTGTGAAGTTTTTTCGAAACTTCA
CTAAAAATAAGACGGCCATAGCCGAACCATTCTTCAGTAGCCATATCAGGGCTTTCCTGAACAATAGAGTTCACTAAACGCTCG
TAATCATATGAACGGCGAATTTCATTGAAAAACACCCAGCCTTCAGTGCGTTTATCATAGGCGTTTAAAATAACATCGCCGGGA
CGATAGAAATTCTTTAAGAACCCCCCATTTGGATCTAAAGCAATATTTTTGCCGCCTCTAATGATGCTCTTAAATAACAGTTCATT
GAAAATTGTGGTTTTACCAGTACCGGTTGTACCGGCAATCGAAAAATGCAAGTTCTCAGCGTATGTAGGTATGGGGATATTAG
CCACGGTTAACTGGTTGACACCTCTTTCGCGTGTTTTATCAGCGAGTGTTCTGGCGCGAACAAGCTCTGTACCACGATAAATCT
TTTTGAATCTTTCGCCTTTAAACACGCGTGATTTATCATAAATGATAAAAGCGATCAGACCGCCAACACCAATAAACCAGCCAG
CAATTAAAGCTGACCATAAAGGCCATAGCGAAAAAGTATTCTTAACCAGATACGGAATCAGGTATTTAGCCGTGGATGGATCA
ATACCGTAGGTAAATTTTGCAACTAGAAACCATACCATCACTGGAGGCAAAGTAATTGCAAATAAAAATGCTAAGCCTCTTTCT
CTATCGTCCATTTCAGCGCTCCTTTTTTGGTTCCCAGACTTTGTAGCCGTTACGTTCAACCTCTGCTTTTGCCGCTTTGGTTTTG
CCCGGTTCTGCTATCGAGCGCAGGAGGATTAGCGTTTCAATAGCGAGTGATTCATGCAACATCATCTGTCCTGCCGGTGGGAA

TTTTACGCCAGATAGCGTTTCGGTTATTGCCTTCAGCTCATCGCGCAGTGGGCCAAAATCCGCATCTGAAGCACGGTCAAAAA
GATAATCCAGTTTGCGATTTACGTCGCTCAGCCGGTCGGCAACTATTTTCAACCCGGACTCCCGATCACCTGGGCCAGCTTCA
ATGCAGCGCCGCAGATAATCTGACCGATTACCTCCTGAAACCAGGTCTATATAGGCCAAAAGTTCATCTGATACTTTTGCGGTT
ATTATTGGCATTCAGTCCTCACATTGTGCATTTCTTAAACAAAAAATTGGGATCTAACAAGCTGAAATCTTAGTATTACCAAAGT
AATAAAGCAAACTCATTATAAAACAATGGGTTATTGGGTGTTTTTAATACCTAATTATTACCGAATATTGACGCTATTTATTTTTT
ATCTTTTAAATCAGTACGATAGCGTGATTTATCGCGCTGCGTTAGGTGTATAGCAGGTTAAGGAAAAAAAATCATCTTTTTTGG
TAGGAGCGACCTCCGTAGGTTAAGGGTCATTTGGCTAAAAAGCGTCCTATTCTTTGATGGTCATGCTTGCATGACCATCTGAGC
AACCAAAAACTACAGATAAACTACAGAGAAACTACAGATAAACTACAAAAAAACGATTTACCTTAGCGTTGTCAGACTACTAATA
GACTACAAGGAAACTACAAAGAAACTACAAAGAAACTACAAAGAAACTACAAATAGACTACTAAAACCGTGGCAGACTACTA
ATAGACTACAAGAAAACTACAAATAAACTACAAAACTGGATTGACCCCTTCTTACGAGTGTTGTAGAGTCATCTTCATACAACG
GAGGGGGTTATGAATAAACCAGCAGATCTGAAACCCCGCAACTTATCGGCTGCTGTCAGATTGCGCCTAAATGAAATCGAGA
ACTGGCTGGACAGAGGGCTAACGCGGCATGAAATTGCTGAAATCCTCGACAGCGAATACAGCTTTTCGGTAACAGCCAAAG
GGCTTGAGATGGCACTGTATAGAACGCGGCAAAACCGAAAAAATGTATTGCACAATACACATGATAAGAGTAGCGCGAAGGG
TGCAGCGGAAAGTGTATTGCACAATACACAACCGTCTGAGCCTGAAGCGCAGGAAAGTGAAAAAGCAGAGAGTCCCGGCAT
TATTGATAAAGAGTTCTTCAATAAAATCGGTGAGGATTTCAACCCTAAGAAGTTCAACAAAAAATTCTGAGGTGATTTATGAAA
GTAGCGGTAATTAATTACAGTGGCAGTGTTGGTAAAACATTAATTTCATCTTACCTGTTAGCCCCGCGCCTGACTGGTGCAAAG
TTCTATGCGGTAGAGACTATCAACCAGTCTGCTTCCGATCTGGGTATTGAAAATGTGACCAGTTTTAAAGGTGACGACTTCTCA
CGTTTGATTGAGGATATTGTTTTTGAAGATGCAGGCATTATTGATATTGGCGCGTCAAACGTTGAAGCGTTCCTGATGGCTATG
TCTCGCTTTGACAGTGGCGCGAACGAATTTGATAAATATGTAATCCCGGTGACGCCGGATAATAAGGCGATTGATGAAAGCCT
GAAAACGGCACACACGTTAAGTAAAGCGGGCGTGAGCAGCAAGAAAATTATCTTTGTTCCAAACCGTATTAGTCCAGACAGT
GAAGTAGAAGATGTGCTGGCGCCGGTGTTTGAGTTTGTCAAAGAAACGAAGATTGGCAAAATAAGCAAGAAGGCTGTTATT
TATAACAGTGAGGTTTTCGAATATCTGGCGTTTCACCGTATCTCATTCGAAGTATTGACCGCTGAAGATCCAGAAGAATTCAAA
TCCCGTGCAAAACAAACAACCGATGCTGACGAGCGCAAAAAACTGGCACGCCGTTATACATACATGAAACAGGCGATTCCGG
TAAAAGCTAATCTCGATAAAGCATATGCGGCTTTAATGGGAGAATAAAATGGAAAAGCAGCCGGATAAATTAGAAGTTCTGAT
GGACTGGTTTTTAGGTGACGCGAAGGAAATCACCGCAACTCAGAAAGAAATGACGCAGAAACTTTCTGAGCTTTCGGAAAA
GCTGGCAAAAGACACCGAAAGTTTAGGAGAGACGGCAGACTCTTTTAAACGGGCTTTAGTAGAAAACCAGCGTTCAATTAG
CCTGGCAATTAGTGATGATGCTAAGGCGCGCGAGGAATTTCTAACTAAATTCCGCCGCGCGCAGGCGTCCAGTGCTGAGACG
TTTACCCGTCAGATCCTTTTTATTACAGCTGGCTGCACCATCGTGGGCGCCGCAGTAGGCGCCGCGATAGCGATACTTTTACTG
AGATAAAGCAAACCGGGCGTGTCCCGGTTTTTTTGTCAAGCGGAGCGCGGAGGCCGAAGGCCGGAGGCATTAGTGGCCGC
CGCCCGCGTAAGCGGGGCGAGACGGGAACCGGCTCGAAGCGCAGCACGGCAGAACGGCCCCGCAGGGGCAATGCCCGTT
TTAATTCATCGTGACAGTCGCGCGTGACCATCACGGGGAGAAAAATAATGAATGACCGACAGCGAGAACTGGCCCGTATACG
CCAGGCCCGCCGCCGCGCGCGGCTCAAGGAAGAAGGCACAAGCGTGACAGTCACGCTAACAAAACAGGAAGAAGCAATGT
TGCAGGAGCTGTGCCGGGTTCGCCGTCCTGGACGAACGCCTTATTCAACGAACGAATTTTTCCAGCTGCTGCTTATCCGCAAC
TGGCAGCAGTGGCAGGAGCAGAAGGCACAGCTGGGAAAATGCCAGGCTTGCGGAAAGCTGAAAGCGGAGGGGGGGTGC
GAGGGTGAACGGAAAGGCGAAACCTTTAACTGCTGGCTTGCCGTCGAAGCCAATGAACTAAATTTGTAGTGTATTGTGCAAT
ACACATTTACACAGAAACAAAAACCACCGGCAATTCCTGGAACCGGATACCTACGGCTATTCCTGGGTGAACGGTACTTTTTG
CACCTGGGTGCGCTGAAAAAGCTGAATATGCAGGGTGACGTTCGCGTGCTGTTCTGCTTTGTAGACTGAATGCGCCAGCTATA
CGCCTGACTGCTTAAACCTGGTAAAGTTCTGCAACCGGCACTGACCGGAAAGCAAGGCAGGGAAGACCTAAGCCAGAAACC
TTGACTGCTCCCCGCCCTTCAGGGCGGGGATTGCGGATCATGTTCTTCTCTTTCAGGGATTCAACGCAGACAAGAAAGGCTT
TCAATTTCCTATACGTGAACGGCCGCGCAGCGGAAAGAAACAAGCCCGGTCAATCCGGGCTTGTTTCTTTAGGCGGCTCAGA
AATCGCCTAAAGGCCCGGCTTGGCCGGGCAGTCAGTGCTATTTAGTTTGTTGCAGCAGCTGGCTTAATTTTGCCGCCAGTGCA
TACGTCGATTGGAAAGCGCCTTGCAGGCGCTGATTAGGCAGCTGGTTGAACGCTTCGAGGCAGGCGCGCAGCAATAATTCTT
TCTGAGATTCGACTTCTTTTTTCAGTTGGGAAGGGGTGGTAACTGTAGTCATGCTTGCTCCTTAGTGAGCCGATATCGGCAATT
TTTCGGGTGGCGGTGTTGCCTCCCGATGATTTAATTATCGGTGATTATGCTTTTAAAGTCAATACAGGTACGGAATTTATTTACC
TGTTTTTATGCCCGTCAGGGCATGGAAGGCGACCGCGCCGGACTCCACCGGACACCGGCCGCAAATCGCCGGAAACTGCGG
GACTGACCGGAGCAACAGGCCAACCCCCCTCCCTGCTAAGCCATAACCCAGCCCGCCGCCACGCAGCTGCCGCACGTCCCCC

ACGGGGGTGCGCAGTGGGCGCCGCGCGCCTGCGCGCGGGTACGGCGGCCCGCCTGCGGGTCGCGGCGCCGTACTGCGAG
TTAGCGGCCGCCGCGCGGCCGGTTACGGGGGACACCGCACAGTCACGGCCAGTGCCCCGCTGAGCTGCACAATCCACGGAT
AACACAATAGCGCACTGGCAAAGGATGCCGACGCCTGAAGGGCGTGGGCACCCCGAAGGGGCGGGGCGGCCGCTTGCGG
CCGGGCGAGTCCGGCGCAGGGTGTGGCCTGCCAAGCGGAGCGCGGAGGCCGAAGGCCGGAGGCGTTAGCGGCCGCTGC
CCGCGTAAGCGGGGCGAGACGGGAACCGGCTCGATGCGCAGCACAGCAGAGCGACCCCGAAGGGGTAACGCCCGGTGTG
GCATCAGGATTTAGTGCAATGGCAGAACATGAGCTGGAGAGATCACCGGCAAGCAGCAGCAAAGGGGCGGCACAGCCGCC
CCGATGGCTGTTTGCCGATACCGGCGATTAATTAGAGCGGTGTTTAATATCCCCCGCGTTGCGGGGGACTAGGTTTCAGCAAG
TCATGTTAAATACGTGTCCATCATGTAAACTGAAATCCCCAATAAACAGATCCCGCGCATAGGCTACGATGTCAAAATATCGGGC
TACGGATTCCGGAATATCATTCAGTAGACCGCTATCATTCAGGTATTCCTCTGCAAAAGTTTCTTCGTCCTTAGCTTCGCCCATAT
AGGCATCTCTAAACAGGTCGAAATCAGTGCTATTAAACAGATCAACAAAGGCCACAAACGCCGCTTCGTTACCTTCCTCGCGG
GCTTGTTTAAAGCCGTTAATAAAATCCCAGTTGATATGGCACTCTGACGCCATACCAGACGGAATACCCTCCCAATCTTGGAAC
ATAAATTCTGGATCAGCCTCATTTGCGTGTAACTCGCGGCAGCGCTCGTAAAACTCCTCTGAGCTATCAAAATCGGTCAGATCG
AGCCAGGCTCCCGCAATGCTTCCGCAGTTGTATTTATGGTAAGTGCCAACATAAACAGAAGGGGTCGTAATATCAGTCATGGT
GTACTCCTTAAAGCGCCGATACCGGCAATTTTTCGGGCGGCGGTATTGCCTCCCGATGATTTAATTATCGTTGATTATGCTTTTA
AAGTCAATACAGATACGGAATTTATTTACCTGTTTTTATGCCCGTCAGGGCATGGAAGGCGACCGCGCCGGACTCCACCGGAC
ACCGGCCGCAAATCGCCGGAAACTGCGGGACTGACCGGAGCAACAGGCCAACCCCCCTCCCTGCTAAGCCATAACCCAGCC
CGCCGCCACGCAGCTGCCGCACGTCCCCCACGGGGGTGCGCAGTGGGCGCCGCGCGCCTGCGCGCGGGTACGGCGGCCCG
CCTGCGGGTCGCGGCGCCGTACTGCGAGTTAGCGGCCGCCGCGCGGCCGGTTACGGGGGACACCGCACCGTCACGGCCAG
CGCCCCGCTGAGCTGCACAATCCACGGATAACACAATAGCGCACTGGCAAAGGATGCCGACGCCTGAAGGGCGTTGGCACC
CCGAAGGGGCGGGGCGGCCGCTTGCGGCCGGGCGAGTCCGGCGCAGGGTGTGGCCTGCCAAGCGGAGCGCGGAGGCCG
AAGGCCGGAGGCGTTAGCGGCCGCTGCCCGCGTAAGCGGGGCGAGACGGGAACCGGCTCGATGCGCAGCACAGCAGAGC
GGCCCCGAAGGGGTAACGCCCTGTGTGGCATCAGGATTTAGCACAATGTCAGAACATAAACTGGAGAGATCACCGGCAAGC
AGCAGCAAAGGGGCGGCACAGCCGCCCCGATGGCTGTTACTTGTCTTTGTCGCGTAGCACTTTGATTAGGCCGGTTACGGCC
GTAATCAGAGCGGCCAGCGAGGTGATGATTTGCGGTAGGTTTTCGAGGATGGTAGAGGTCATATAGCACCTGTAGAGAAGTT
GGCGGGGTGTCGTTTCCGACGGCCGCACTGTAACCGGGCGAATAAGGCAGGTTGTCAACAGCTTGAGCGAAGCGTCTGTTG
ACAACCTGCCGCGCCCGGTTTCACTGCGGTCATAGGCGGAACGACCCACGCCAACGGAACGGCTTTATGACCGGGCAGCTG
AGATACCGGCGAACCTGGCTGGCGGCTGACGCCAGCCGCCAAGCGCCAGCGCGGAGGGCAAAGCCCGGAGGCCAAGCGG
AGCGCGGAGGCCGAAGGCCGGAGGCCGGAGGCGTTAGCGGCCGCTGCCCGCGTAAGCGGGGCGAGACGGGAACCGGCT
CGATGCGCAGCACAGCAGAGCGGCCCCGAAGGGGTAACGCCCGGAGTCTGCCGCTGTTTATCTCTCGTTCCATCTGAAATCG
GCGGTAAGGCCATTAAAAGGGTCAGTTTATCAGGGAGGCGTTAGCCCCCCATGTTGTTAATCATCAGGCAATATCGTCTTTGTA
GCAGGCATAACCGAAGCTAAGCTCTGTTTTCATATAGTGGCGGGCAAAGTCCCAGGCATCGTGGCCGAAGTCCTCATAATCTG
CCAGGACGATTTCGCGGGCTTTGTGCCATTGCTGTACGGAAGAAAGCGGCAGAACAGGGCAGGGGTGCGACCAGTCAGTG
ACGGTGTTGCAGATCATATCTGCCAGACGCTCCAGGGAGCCGTAAACCAGCTCTGTGCGCAGATCCGCCACCATGCGTTGTTT
ACGCAGTGATGCCAGATAATCAATCTCTTTGGTTATATCAGAATTTAAGCGGGTCTGGTAATCCATGATGTACTCCTTTGCGCGC
CGATACCGGCAATTTTGCGGGCGACGGTGTTGCCTCCCGATGATTTAATTATCGGTGATTATGCCCTCAAAGTCAATATAAGTA
CGGAATATGCATGCATAATTTTATATCTTGCAAAGCGTTCATAGAGTGCCTGAATCGCTTTCTGACAGCCTCAATAAAAAAAGG
CGGGGATTCCCGCCTTTTTTCTTACAGCTGCTTACGTGGCTTTTTACGCGTCATATACAACGGTATCGCGCAGTCTACCGCGTA
CAAAAAGCACGCCAGCGCGCCGCAACCGTACAGAAACGCAAGCGGCTTATTATCGAAGTAGCTGAAAACCCCTGTCGCCGC
ACACAGGCCCAGAACAGAGACGCAGGCGCAGGTGATCTGCACCAGATCCCTGTATCCCGCACGAACGATAAACCAGGGCAG
GGCAAGCGCAGCGGCGCTAATAATTAATGCGAGAGGGACAAAAACGAGATAGTGATACATGTGAACTCCTTGATGGTTGCCG
ATACCGGCGATTGTTCGGGCGGCGGTATTGCCACCCGATGATTTAATTAGAGGTTTTGCGCGTCCAGGAGATTGACCTGAGCC
GGGGTAACGTGAAACTTTTCCCCTTTATGGATCACGTTATGCGGGGCGCTAATTTCATCACTGATAAAGCTAACCGGGTAACGT
TTTTTACCGCAAATCCGCTCGCTAAACCATGCCACTTTTGCCGCTGGCCGATCCACTGGATGAACAATCACACCGGCCATGCTG
CAACCCGTTGCGGGTTCGTCCAGCGTAATGCTTACCGGGACTGTATCCCGGTAAAAAAACTTAACCGGCGGCACACCTGCCT
GCGTAGCGGCTGCGACAACTGCAAGCCCGATAACCGCTATCCGATTAATAAGCATTTTATTCCCCTTACTCATGCTGATATCACC
TTGCCAGCTGTTACCAGTTTACGAAATTCACTTTCATGAATTTCACGCCCATGCTCAAGCGTTGAATACACGTTGCCGATAAGC

CAGTTACCAGCCGTTTTTGTCTCGGTATACCACCATGCTTCTGTCAGAGTAAGCAGCGGTTCGCTATCCTCGCCCTTCTCATAGA
TCCAGATTTTAGTGACGTCTTTACCCGTTTCGTTGCTGCCCTGAATGGTAGGGTCAAAGGTATGTTCAATCTCTATATCGAAGTA
ACGCTGAAGGAAGTTAGTAAAGTGCATGACGACTCCTGTAAGCGCCGATACCGGCAATTTTTCGGGTGGCGGTGTCGCCTCC
CGATGATTTAATTATCGTTGATTATGCTTTTAAAGTCAATACAGGTACGGAATTTATTTACCTGTTTTTATGCCCGTCAGGGCATG
GAAGGCGACCGCGCCGGACTCCACCGGACACCGGCCGCAAATCGCCGGAAACTGCGGGACTGACCGGAGCAACAGGCCA
ACCCCCCTCCCTGCTAAGCCATAACCCAGCCCGCCGCCACGCAGCTGCCGCACGTCCCCCACGGGGGTGCGCAGTGGGCGC
CGCGCGCCTGCGCGCGGGTACGGCGGCCCGCCTGCGGGTCGCGGCGCCGTACTGCGAGTTAGCGGCCGCCGCGCGGCCGG
TTACGGGGGACACCGCACCGTCACGGCCAGCGCCCCACTGAGCTGCACAATCCACGGATAATGCAGGAGACGAATCATGATA
GGAGGCTGAAGGGGAAATGAGCGGCAGCAGGGGAAGGGGTTGCCAAGCGGAGCGCGGAGGCCGCAGGCCGGAGGCGT
CAGTGGCAGCTGCCCGCGTGAGCGGGGCGAGACGCGTAGCGGCTCGATGCGCAGCACAGCAGAACGGCCCCGGAGGGGT
GACGTCCGGGGGTTCGCTTTTAAAGATTTTCGACCACATCAGTAAATCGTAGTGACACCATGAAGCAAAAGTATCGTGCAGAC
CAGAATCAACAACAGTAACAGCAGACCTTTTTTTCGACGTAATAAAACCGGCCCCAAAGCGCCCGCAAGAAGCACACTCAAC
ACGATTTCCGTTATACTAATCGTATTCATAATCTCTCACGTTTCCCTTTTTAGAACTCTGCCACACACAGATAAAACCTTATAACA
AGCTACAAAACCCGTTATTCAGACGCGGTAATGCCTAGTTTTTTTGCCAGTATTTCAGATGACAAAGAAACCCTTATCCACTTC
CCGGTTACAAGGTGAATGATTGTGGCTTCATGCCCTTTAACAGAGTGGGATATTAACTCACGCTGAATGATATGCGTCTCTCCA
TCATTTCCCGTCACGGCAATTAGGCCCTCTGTAGAACAGGGGTTGATACTACTAATGTTGGTGTTGTTTTCGGTTTTGTTCAGC
ATCGCTGATCCTCAAATATCGGTTTGTGTTACGTCTGCCGCTTTGCGCTGGATAAGCGACTTAAAGAAATCCGACGCCTTCAGA
ATATCGCTATCCTGGAAGTCGGGAAATGTCGCTTCTGTCAGCTCCTGCGCCGTACCTGCCCGTATAACGGTTTCATCGCGGATC
ATCGTTCGCTTAACGCCATCAGCCTGTTTTTTGCTGAAGGAGTGCCGGAAAATAATCGCGTATTGGCCGCCGCTATCCTGATGA
ACAGAAGTATCCCAGCGATGATTACCCTGTTTACGATAGTGGTGATAGATTTGCTGGCCGTAGCGCTCCTGATCGGAATTACGG
TAATCGAACATTAACAGTTCCAAAAGCATTAACCGATCTGGCAATTGCCAGGCGGTAGGGTGTTGAAGTGTCGCTAACATAGT
TTCCCCTGAGCGTGACAGTCACGATAAGGCGGGCTTTGCCCGCCTGGTTATCAGTTAATCAATGGCACGATAAATACGATTCT
GGCTTTCGTTCTCCAGCGTATTAACGTACTCCCGCAACAGGTGATACCGGTTAGCCATCGTTTCGTTCAGTTCGGCTTTGCCTT
CTTCATATGCCAGGCCGCAAAAGTAACTGTAGGCATACAGACAAACAATAATCCCTACTTCGCGCGCGCTGCATTCACCTTCAA
AATAGTTAGGTAACGAGAGCCAAAGAGGTTGGGGCGCTTCCATAAAAAACGCGCCATTGCTGGCCTGAAGGTATTCCCAATA
CCCTCCCTGGTAGTCTTTAGCGTAACGATTCAGAAAGGACTGAATGAAGTGATCTGCGCTGAAGAAAGCGCCACGAAATGCC
GCAGGCATGAAGTTCATGCGGGCGTTTTCAGAAATGTAGCGGGCGGTGATTTCGATAGTTTCCATGATACTTCCTCTTAAAGC
CGATACCGGCGATGGTTAAGCGGCAGGCACATCACCTGCCACTTTTTAATTATCGTACAATGGGGCGTTAAAGTCAATATAAGT
ACGGATTATATTTACCTAATTTTATGCCCGTCAGAGCATGGAAGGCGACCTCGCCGGACTCCACCGGACACCGGGGGCAAATC
GCCGGAAACTGCGGGACTGACCGGAGCGACAGGCCACCCCCCCTCCCTGCTAGCCCGCCGCCACGCGGCCGGTTACAGGG
GACACTGAGAAAACAGAAAGCCAACAAACACTATATATAGCGTTCGTTGGCAGCTGAAGCAGCACTACATATAGTAGAGTACC
TGTAAAACTTGCCAACCTGACCATAACAGCGATACTGTATAAGTAAACAGTGATTTGGAAGATCGCTATGAAGGTCGATATTTT
TGAAAGCTCCGGCGCCAGCCGGGTACACAGCATCCCTTTTTATCTGCAAAGAATTTCTGCGGGGTTCCCCAGCCCGGCCCAG
GGCTATGAAAAGCAGGAGTTAAACCTGCATGAGTATTGTGTTCGTCACCCTTCAGCAACTTACTTCCTACGGGTTTCTGGCTC
GTCAATGGAAGATGGCCGCATCCATGATGGTGACGTACTGGTTGTGGATCGCTCGCTGACGGCCAGCCACGGCTCAATCGTA
GTCGCCTGCATCCATAATGAATTTACCGTGAAGCGGCTACTGCTGAGGCCCAGACCCTGCCTGATGCCGATGAACAAAGATTT
TCCTGTGTACTACATTGACCCGGATAATGAGAGCGTTGAAATCTGGGGAGTGGTTACGCATTCCCTTATCGAGCATCCGGTATG
TTTGCGCTGATTGATGTCAATGGCATGTACGCCAGCTGTGAGCAGGCATTTAGGCCAGATCTGGCAAACCGAGCAGTGGCCG
TTTTATCCAACAATGACGGCAACATTGTGGCCCGTAATTACCTGGCGAAGAAAGCGGGCCTGAAAATGGGCGATCCGTACTTC
AAAGTCAGACCCATAATCGAGCGTCATAACATCGCTATTTTTAGCTCTAATTACACTCTCTATGCCTCCATGTCGGCCCGGTTCG
CGGCCGTAGTTGAGTCCCTTGCAAGCCACGTCGAACAGTATTCAATCGACGAGCTTTTTGTTGACTGCAAAGGGATAACGGC
CGCCATGAGCCTTGACGCTTTCGGGCGCCAACTGCGCGAGGAAGTCAGGCGACACACAACGCTGGTATGCGGGGTCGGTAT
TGCCCGTACTAAGACGCTGGCGAAGCTGTGTAACCACGCTGCAAAAACATGGCCCGCTACTGGCGGGGTGGTTGCTCTGGA
CGATGGCGCCAGACTGAAGAAATTAATGAGCATCCTGCCGGTTGCGGAAGTCTGGGGCGTCGGCCATCGTACAGAGAAAGC
ACTCGCCACAATGGGGATCAAAACGGTGCTGGATTTAGCCAGGGCAGATACGCGCCTAATCCGTAAAACATTCGGCGTTGTG
CTTGAAAGAACGGTACGGGAGTTGCGCGGCGAGGCTTGCTTCAGCCTGGAAGAAAACCCTCCTGCGAAGCAGCAGATTGT

TGTGTCGCGCTCATTCGGCCAACGCGTAGAAACCCTGACGGACATGCAGCAGGCTGTCACCGGATTTGCAGCGCGCGCAGC
TGAAAAACTGCGTAATGAGAGGCAATACTGCCGCGTCATAAGCGTCTTTATCCGTACCAGTCCTTATTCAGTGCGTGATACACA
GTATGCCAATCAGGCAACCGAAAAACTGACGGTGGCAACCCAGGACAGCCGCACGATAATTCAGGCAGCACAAGCCGCGCT
GGCGCGGATCTGGCGGGAAGATATTGCGTATGCAAAAGCAGGGGTCATGCTGGCAGATTTTAGCGGGAAGGAGGCCCAGC
TTGATTTATTCGACTCTGCTACGCCTTCAGCTGGCAGCGAGGCTTTAATGGCTGTTCTTGATGGTATAAACCGGCGTGGAAAG
AGCCAGCTTTTTTTTGCAGGCCAGGGCATCGATAACTCCTTTGCCATGCGTCGTCAGATGTTGTCACCTGATTACACGACAGA
CTGGCGCTCAATACCAATAGCCACCATCAAATAATTACCGGCGCCGTACACGGGCCGGTTAACCCCTCAACCGGCCGAAACAA
GTTTCGGCACGGTTTCGCGGTTTTCGGTAAAAGCCGTTTCCTCTGTATAAAAGATCAGCTAAATTATGTGTATTGCACAATACAT
ATATGTGAGGTTAGCAGTGAATTTGCCTACGCCCGAAACCTACGATGAACTTCAGAGAGCCTACGATTTTTTCAATGATAAGCT
ATTCAGCAACGAGCTGCCGCCATGCCTGATAACGTTGCAGCGTGAGAAGCGAACGTATGGCTATTGTTCCTTTAAGCGTTTCG
TCGGCCGTGAGAGTGGGTACACGGTAGACGAGATCGCTATGAATCCGGTGTATTTCTCGATCAGAACCATAAAGGCCACGCTT
TCAACACTGGTGCATGAGATGGTTCATCAGTGGCAATTCCATTTTGGCGAGCCTGGCCGCCGTGGCTATCACAACAAACAGTG
GGCGGCCCGGATGGAACGGGTAGGACTAATGCCTTCTGATACCGGCGAACCGGGAGGCAGGAAAGTGGGCCAGAGCATGA
CCCATTATATTATTGCCGGTGGCCCTTTCGATATGGCCTGTGATGAACTGCTGACAGGCCATTTCCGGCTTTCCTGGATGGACA
GGTTTCCGCCTTACCAGCCTAAGCCTGGCGCTGTGCTAAGCCCTACAGGAAAAGGCTATATTGACGACGAGGAAGATGATAG
CGAACACGAACAGGAGGTGGAGGAAGGGCGCGACCCGGTTGAACTCGACGACGAGATCATAGAGGCCATGCGATTTGTAA
CCCCACCGCCTGAAGCACCGGTGAACAAAACAAACCGGGAAAAGTACAGCTGCCCGGTGTGTCATATCAATCTCTGGGGTAA
ACCGGGGATAGTGGTTTACTGTGGTGGCGAGCACTGTAATAAAGCCGCGTTAGTAGTCTTAAAATAAAGTCCTTTCGGACTTT
ATTTTTTTTCCATTTCCGAGGTCGTGATGTTATTAATGCTGTACTTCGCGGCTTCTTTTAAAACAGTTTCAGCAAGGCTTGCTGG
TATCCAGACCTGAACTAATTTTAATGGTTCGCCGTTCTCGGCTTTAAGAGTGGTGTTCTGGTACAAATCCCAGATTCGCTTAAC
GGTGCTGGAAATGTTTTGCTTGGAACGGCCTACTCGCATGGCTACGTCTGATGATTTCTCACCTTTGACAAGCACGGAATAGC
CAATATCTGTTGTGATGTGTGCAAAGGAAGCCATTTGCGGCAGCAGCTGTTTCCATTCTGTTTCTGAAATTCTGTTTTTCTGAG
CCATCTGTGGCGCCTCCGTAGTTTTGGTTACAGAAAGGATATACTCAGAATAAACAGGGGTCAATACAAGTACGATTTTTATAA
ACTTTATTTTATTTGAGGGTGAGGCCCGGTGCGGCAGCAGCGCGGGCCTCGATGGTGCCGCGAAGGTGCTGGCGCCATGCT
CGGATTAAAACATGAACCGTGAAGAACTGCGAAACTTGTTTTCGCGGTTCTGAGGGGTTGACCGAGCCGCGAAGCGGCGCT
GGTAAGCGATGATATGCACATATCCACAGGCATATTTTTAAAAGGTATTTTATAGATTTTTTATCTTTTTAAAGTCTTTTAGAGCT
ATATAACTCATTGATTTAAAATCATAAATAAGTGTTATCTCTGGGAATCCGCCCACCTTGTTATGGGAATTGGCCCACCTTACTAT
GGGAAACAGCCCACCTTACTATGGGAATTAGCCCACCTTGTTATG

>pQEB1_inv1_arsTn

GGAATTGGCCCACCTTAGACGAAACTGTAAAAAATGTATTTACTTGTTTGAACTTTGTGGTAGTGTGGAGAGTAATTTTTAACC
CACAAAGGCAAGGCGCATGGATAAGTTGCTGAACAAAAAGATAAAAGTTAAGCAGTCTAACGAGCTTACCGAAGCTGCTTAC
TACCTCTCGCTAAAAGCAAAGCGCGTTCTCTGGTTATGTCTTATGCAGACGTATTTCACAGCTTCAGTAAGCGAAGATGATGAT
GAGATGGCTGTACTCGGTGACTCTACTTTCAAAGTAAAGGTGGCTGACTATCAGCAAATTTTTCAGGTAAGCCGTAACCAGGC
TATCAAGGATGTTAAAGAAGGCGTGTTTGAGTTAAGCCGTTCTGCGGTAATCTTTTACCCGAAAGAGGGGCGTTTTGACTGC
GTCGCGCGCCCCTGGCTAACAGAGGCTGGCAGCCGATCAGCTCGTGGTATCTGGGAAATCGAATTTAACCATAAACTCCTGC
GGTACATTTACGGCCTGACGAACCAGTTCACCACCTACTCGCTCCGCGATTGTGGCAGTCTTCGAAATCCCCGGACGATCCGC
CTTTATGAAAGTCTTGCTCAATTCAAATCTTCAGGCTTATGGGTTACTACTCATGCTTGGTTAAATGACCGTTTCCTTTTGCCGG
AATCCCAACAGAAGAACTTGGCAGAGTTGAAACGATCTTTCCTTGATCCTGCACTCAAGCAGATAAATGAGAAAACACCTTTA
CTTGCTAAGTATAGTATTGATGATTCAGGAAAATTTCTGTTCTCAATAATTGATAAGCAAAATCCCGTCTGACATAAATCAGCAC
ACATGAGCCTGTCATTTGACAAATTTTTGTCATGAAGATGGGCGAATTTCCACACAGCACCGGCGCCCGGCAAGATGGGCGG
ATTCCCACACGACAGCGGCGCCCGGCAAGATGGGCGGATTTCCACACTACAGCGGCGCCCGGCAAGATGGGCGGATTTCCA

CACGGCAGCGGCGCCCGGCAAGGTGGGCGGATTTCCACACGGCAGCGGCGCCCGGCAAGGTGGGCGGATTCTCACGCGG
CAGCGGCGCCCGGCAAGATGGGCGGATTTCCACACGGCAGCGGCGCCCGGCAAGGTGGGCGGATTCTCACGCGGCAGCG
GCGCCCGGCAAGGTGGGCCGATTCCCACGCGGCAGCGGCGCCCGGCAAGGTGGGCCGATTCCCACGCGGCAGCGGCGCC
CGGCAAGGTGGGCCGATTCCCACGCGGCAGCGGCGCCCGGTAAGGTGGGCGGATTTCCACACGGCTGCCGCGCCCGGCAA
GGTGGGCGGATTTCCACACGGCAGCGGCGCCCGGCAAGGTGGGCGGATTCTCACGCGGCAGCGGCGCCCGGCAAGATGG
GCGGATTTCCACACGGCAGCGGCGCCCGGCAAGGTGGGCGGATTCTCACGCGGCAGCGGCGCCCGGCAAGATGGGCGGA
TTTCCACACGGCAGCGGCGCCCGGCAAGGTGGGCGGATTCCCACACGGCAGCCTCGCCCGGCAAGGTGGGCGGATTCCCA
CACGGCAGCCTCGCCCGGCAAGGTGGGCCGATTCCCACGCGGCAGCCTCGCCCGGCAAGGTGGGCGGATTCCCACACGGC
ACCGGCGTGCGGCAAGGTGGGCGGATTCCCACACGGCACCGGCGCGCGGCAAGGTGGGCCGATTCCCACACGGCACCGG
CGCCCGGCAAGGTGGGCCGATTCCCACACGGCAGCCGCGCCCGGCAAGGTGGGCCGATTCCCACGCGGCAGCCTCGCCCG
GCAAGGTGGGCCGATTCCCACGCGGCAGCCTCGCCCGGCAAGGTGGGCCGATTCCCACACGGCAGCGGCGCCCGGCAAGG
TGGGCGGATTTCCACACGGCAGCGGCGCGGGGCCAGTGGGATTCAGGAGAATAGGTGTTTTACCGAATGCCCTGACGAGG
CGTAAAAAAACCGCTTGGCGGCGGCCTCATAAAGCAGAAAACCCGCTCAAGGCGGGTTATCTGCTCTGTAGCCTGTGATGCT
TCGCGGGCATCCGGCATACAGCGAGGTGAAATTCTTCTTTTGGCATGTTAATTATACGTCTAACGCGGCATATGATCAAACCTG
TATTAAATAAGCCACTGTACCGTTTATAATGCTCTCAGATCAAAGAGGTAAAGCCCGTTTAGCCGCCTGTGTGATGAGCCAGTT
CAGACTCTTCAAAATCGAATTTGGTACTAAACAGGACCCGAACCGTGGGCAAGCACACGGCAACGGTATAGCCCTCTTCCGG
TTTCGCACCCGGAAGCCTGGGCGGCAGCGTGGTGAAATTCTTCTTTTGGTTAAGTGAATGGCATAACCGGATGGGCGGATTA
GAGGAAAGGGGATTGCCTAGTAACCTACGCGCCACAGAGATGGAGGTGCGGGGAATGATTGAGCTGATTATCGCTATTCTGA
CCTTAATTGCGGCTGTATTGCAGTTGATCAACTGGTTCCTTTAATGGTGCCGGAGTCTGTGAAGGTGAAAGCCTGAACGGGC
AAAACTGAAAGGTTTATAGCCGTCCTTCGGGGCGGCTTTTTTTCGGCAAAATTAGGGTTTTACCGAATAATGCAGAGTTTTAA
GGTGAGAATTTGCAGACTTGGCGTTTTACCGAACATAGATACTCCCTAGGCTGATAGGTGCATTAGTTATCACCCTACCTGAAC
ATATTGTAAAAGATGTCAGTCTCCAGTGACTTGTGTACTATCAACTGACAAGACTCTTACACGCAACGCAGGGGGATGGAGTT
TTATGCTTAGAAAAATAATCAGGGGTAGCGGATTCACTCAGTCAGAAGAAAAACTGATAGAGTTCGCTGATGATGCTTTTTTT
GGTCTTTGGTCTTATCCTAATGTTTATAGCGATGAGGGTTACTCTAAAAATAAAATTGGGAAAGAAGTTAGTGACTTATTAGTTA
TTTTTGATAAAGATATAATAATTTTTTCCGATAAAGCTATTACATACAATAAAAACAAAGATCCTAAGGTTGCATGGCAGAGATG
GTTTAAAAAATCAGTCATACAGTCTTGCACACAGTTATTTGGCGCAGAGAAGTTTATAAAAGATCATCCCGAAAGACTTTTTGT
TGACAAAGAATGCTCAGTTAACCTCCCCATTAAAATAGATAATTCTTTTAATTTTCATTTGGTGGCCGTCACTAATAATATTTCAG
ATCCGGCGATCTCGTACTTTGACAAAATAGAAAAAGGCAGCTCTGCTACTTTAGTTAACATATTTCCTTTAAACGCCCATCAATG
TCTAGAAAATCCATTTTGTGTCGGAGACGTTTATCCTGATAAGACTTTTGTCCATATACTTGATGAGACTGCCCTAAAACTACTG
TTAACCGAGTTAAACACAGCAACTGATTTCATTGGCTACCTTAACGAAAAAGAGAGGGTTGTAAGAGAAAGAACATTATTGG
TCAGCGCTGGGGAAGAAGAGACTCTTGCTGCTTACATTATGGGTGATAAAACCATAATATCAAAAGAAATTATTGGAAACGAT
CAAGGGATGACCATACCGGAAGGTGAATGGAAAAACTATAAAACCACTTTCAATTATCAATATCAGCTCTCAATGAAAAAGGG
TAGCGTTTTCTGGGATAACCTAATCCACAACTTCTCGACAAGTATATTGTCAGCTAACGTTGGTTTTTTTAGTGAAATTGAATTT
TCTACACATGAATTAGGTGTTAGAGAATTAGCCAAAGAAAGTAGGCAATCTAGATATTACCTTTCAAAGAACTTTAAAGAGAA
ATTAAAAACAACTCAGCCTCATCTAAGAACGTCAAGAATGGTCGAATCAATCGATGAGCCTGGAAAGTTTTACTTATTCCTTTT
TTTTCCTAACGATAGCAAGTTGAGTTACTCTGATTACAGAATTCAACGTATATCTTATATAAATGCTTATGCTGAGGTTGCCTTTA
ATAAATACAGACATATTAAAAAATTAATTACTATTGCAACAGAGCCGCAAAATACAGAAGGAAGATCTGAAGACCTAATATATA
GCATATCCCCAGAGAAATTTACCAAAGAGCAAAATGAAAAAGCCAAAAGATTATCAAGAGAATACAAAATACTAAGTGATTTT
TTACCTACTAAAACGACAAAGAGCGATAACTTTAAATCAGTTATATCAAAAGGTGAAAAAATAGGGCGGAATACACCTTGTCC
ATGTGGCTCCGGTGTTAAATTTAAAAAGTGCCATGGTGCGAATAATTAGCATTATTGTATGTATAACGGTAATGGCGCGGCAGA
GAAACCGGCGCGTTCTGCCCTAGTGTTGGCCTGCGGGTTCCCCCGCACCCGCTGTATGTAGTATCGGCAGCATCTGAGAAAA
CCACTACATGTAGTTATCAGCGCCACAACGGCGCGGGGACGAGTGCGGTTCGGAAAAATTGGGGTTTTACCGAATCCGGCA
AAAGATTGCTTCCTATAACGTCCGCTTCTGGCACACAGCAGCCGTTAAGATGTAAGGCCTTACGCCAACTAAATCTAATGGGA
CAGATTTAGTTGGTGATGGTCAAGTAATCTGCAAACGGTCACCAAGTAAAATGCAAATGGGTAGTCAAGTCCGATGCAATTAC
GCACCCGGCAAGGTGGGCCGATTCCCACACGACAGCAGCGCCCGGCAAGGTGGGCGGATTTCCACACGGCAGCGGCGCCC
GGCAAGGTGGGCCTATTCCCACACGGCAGCGGCGCCCGGCAAGGAGGGCCGATTCCCACACAGCACCGGCGCGCGGCAA

GGTGGGCCGATTCCCACACGACAGCAGCGCCCGGCAAGGTGGGCGGATTTCCACACAGCACCGGCGCCCGGCAAGGTGG
GCCGATTCCCACACGGCAGCGGCGCCCGGCAAGGTGGGCCGATTCCCACACGGCAGCGGCGCCCGGCAAGATGGGCGGAT
TTCCACACAGCACCGGCGCCCGGCAAGGTGGGCCGATTCCCACACGGCAGCGGCGCCCGGCAAGGTGGGCGGATTCCCAC
ACGGCAGCGGCGCCCGGCAAGATGGGCGGATTCCCACACGGCAGCGGCGCCCGGCAAGATGGGCGGATTTCCACACGGCA
GCGGCGCCCGGCAAGATGGGCGGATTCCCACACGGCAGCGGCGCCCGGCAAGATGGGCGGATTTCCACACGGCAGCGGC
GCCCGGCAAGGTGGGCCGATTCCCACACGACAGCGGCGCCCGGCAAGGTGGGCCGATTCCCACACGACAGCGGCGCCCGG
CAAGATGGGCGGATTCCCATATCGACATGTATGTAGCTTGTGTTATCCGTGGATTGTGCAGCTCAGCGGGTCGCTTGTCGTATG
GCGTAGTGTCCCCCGTAACCGGCCGCGTGCGGCCGCTAACGCGCAGTACGGCGCCGCGACCCGAAGGCGGGCCGCCGTTCC
CGCGCGCAGGCGCGCGGCGCCCACTGCGCACCCCCGTGGGGGACGTGCGGCAGCTGTGTGGCGGTGAGCGGGATTAGGG
CTTTGCAGGGAGGGGGCTGGGTCGGGCGATACGTTCAGCATTGCGGTTTCCGGCGATTTGCGGCCGGTGCCCGTTTAACTC
CGGCGTGGTCGCCTTCCATGCCCTGACGGCATAAGAAAATAAAACCGCCATGCTGCGGTCATTCATGATTTTGTGGTGTAGCG
ATAAATAGTCATGCGAGAAACGTTGAAGCGCTTAGCAACTGCACCAACTGTCATTTCAGGATCAGCAAGTAAGATTCTAATTT
GTTTAACATCTTCTTCAGAAAGTGACGGTTTTCTCCCTCCCACACGGCCCCTTGCGCGTGCAGCTGCAAGGCCTGAGCGCGTT
CTTTCAATATTGCGGTTGCGTTCAAAGCTAGAGAATATCGCCATCAGATGAGTATAGATTTCCCCTATAACTGGCGCATTTGTGT
CTATTCTGTCCTTGATGGCTATGAAAGTTATTCCGCGTTTCTTCAGGTCGTCGAGTAAAGTAATGACTTGACCCAATGAACCAC
CGAGCCGATCTAGTGCCCAAACTACTAGGGTATCTCCCTCGCGCAATGCTTTCAGGCAGTTCTCCAGTTCCAGCGCACCTTTTT
TGTCGCGCTTTGGGCCGCTACGTGAGGTCTGATCCTGATAGATTTGCTCACATCCAGCTTTTGTTAGTTCGTCAACCTGGTGCG
CCACATCCTGAAGATGCGTAGATTTACGGGCACTGTTGCAAATAGTCGGTGGTGATAAACTTATCATCCCCTTTTGCTGATGGA
GCTGCACATGAACCCATTCAAAGGCCGGCATTTTCAGCGTGACATCATTCTGTGGGCCGTACGCTGGTACTGCAAATACGGCA
TCAGTTACCGTGAGCTGCAGGAGATGCTGGCTGAACGCGGAGTGAATGTCGATCACTCCACGATTTACCGCTGGGTTCAGCG
TTATGCGCCTGAAATGGAAAAACGGCTGCGCTGGTACTGGCGTAACCCTTCCGATCTTTGCCCGTGGCACATGGATGAAACCT
ACGTGAAGGTCAATGGCCGCTGGGCGTATCTGTACCGGGCCGTCGACAGCCGGGGCCGCACTGTCGATTTTTATCTCTCCTCC
CGTCGTAACAGCAAAGCTGCATACCGGTTTCTGGGTAAAATCCTCAACAACGTGAAGAAGTGGCAGATCCCGCGATTCATCA
ACACGGATAAAGCGCCCGCCTATGGTCGCGCGCTTGCTCTGCTCAAACGCGAAGGCCGGTGCCCGTCTGACGTTGAACACCG
ACAGATTAAGTACCGGAACAACGTGATTGAATGCGATCATGGCAAACTGAAACGGATAATCGGCGCCACGCTGGGATTTAAA
TCCATGAAGACGGCTTACGCCACCATCAAAGGTATTGAGGTGATGCGTGCACTACGCAAAGGCCAGGCCTCAGCATTTTATTA
TGGTGATCCCCTGGGCGAAATGCGCCTGGTAAGCAGAGTTTTTGAAATGTAAGGCCTTTGAATAAGACAAAAGGCTGCCTCA
TCGCTAACTTTGCAACAGTGCCCCTTTTAAAAATATGCCTGTGGATATGTGCATATCATCGCTTACCAGCGCCGCTTCGCGGCTC
GGTCAACCCCTCAGAACCGCGAAAACAAGTTTCGCAGTTCTTCACGGTTCATGTTTTAATCCGAGCATGGCGCCAGCACCTTC
GCGGCACCATCGAGGCCCGCGCTGCTGCCGCACCGGGCCTCACCCTCAAATAAAATAAAGTTTATAAAAATCGTACTTGTATT
GACCCCTGTTTATTCTGAGTATATCCTTTCTGTAACCAAAACTACGGAGGCGCCACAGATGGCTCAGAAAAACAGAATTTCAG
AAACAGAATGGAAACAGCTGCTGCCGCAAATGGCTTCCTTTGCACACATCACAACAGATATTGGCTATTCCGTGCTTGTCAAA
GGTGAGAAATCATCAGACGTAGCCACGCGAGTAGGCCGTTCCAAGCAAAACATTTCCAGCACCGTTAAGCGAATCTGGGATT
TGTACCAGAACACCACTCTTAAAGCCGAGAACGGCGAACCATTAAAATTAGTTCAGGTCTGGATACCAGCAAGCCTTGCTGA
AACTGTTTTAAAAGAAGCCGCGAAGTACAGCATTAATAACATCACGACCTCGGAAATGGAAAAAAAATAAAGTCCGAAAGGA
CTTTATTTTAAGACTACTAACGCGGCTTTATTACAGTGCTCGCCACCACAGTAAACCACTATCCCCGGTTTACCCCAGAGATTGA
TATGACACACCGGGCAGCTGTACTTTTCCCGGTTTGTTTTGTTCACCGGTGCTTCAGGCGGTGGGGTTACAAATCGCATGGCC
TCTATGATCTCGTCGTCGAGTTCAACCGGGTCGCGCCCTTCCTCCACCTCCTGTTCGTGTTCGCTATCATCTTCCTCGTCGTCAA
TATAGCCTTTTCCTGTAGGGCTTAGCACAGCGCCAGGCTTAGGCTGGTAAGGCGGAAACCTGTCCATCCAGGAAAGCTGGAA
ATGGCCTGTCAGCAGTTCATCACAGGCCATATCGAAAGGGCCACCGGCAATAATATAATGGGTCATGCTCTGGCCCACTTTCCT
GCCTCCCGGTTCGCCGGTATCAGAAGGCATTAGTCCTACCCGTTCCATCCGGGCCGCCCACTGTTTGTTGTGATAGCCACGGC
GGCCAGGCTCGCCAAAATGGAATTGCCACTGATGAACCATCTCATGCACCAGTGTTGAAAGCGTGGCCTTTATGGTTCTGATC
GAGAAATACACCGGATTCATAGCGATCTCGTCTACCGTGTACCCACTCTCACGGCCGACGAAACGCTTAAAGGAACAATAGCC
ATACGTTCGCTTCTCACGCTGCAACGTTATCAGGCATGGCGGCAGCTCGTTGCTGAATAGCTTCTCATTGAAAAAATCGTAGGC
TCTCTGAAGTTCATCGTAGGTTTCGGGCGTAGGCAAATTCACTGCTAACCTCACATATATGTATTGTGCAATACACATAATTTAG
CTGATCTTTTATACAGAGGAAACGGCTTTTAGGGGTCGTTTGCGGGAGAGGGCGAAATCCTACGCTAAGGCTTTGGCCAACG

ATATTCTCCGGTAAGATTGATGTGTTCCCAGGGGATAGGAGAAGTCGCTTGATATCTAGTATGACGTCTGTCGCACCTGCTTGA
TCGCGGCCGCGATAGCTAGATCGCGTTGCTCCTCTTCTCCATCCGCGTTCCAAGCTGCGGAAAGGCACCCATAAGCGTACGCC
TGGTCGAGCAGGCGACGCGGATCGACGTCCAGCGCACGAGAGAATGCGTCCGCCATCTGTGCAATGCGTCTAGGATCGAGA
CAAAGGTCGTCTCTGTCAGCCGGATCGTAGAACATATTGGCGGCGCCAAAGCCCACTTCACCGACCAGACCGACGGGATCTA
TCACCAGCCAGCCGCGACTGGAGAACATGATGTTTTCATGATGCAGATCGCCATGTAGCCCACGCAGTTCCGAGGCATTGCTC
ATCATTTGATCGGCTATAATCGCCGCGTGGACGTAGTCAGTTTGACAACCTGCGTTTTGATCATCGCGCGCCCGCTGAAACAA
AGCTGCAAAGCGATCCCGGATCGGGAGAAGGGCAGAAGGCAGGGGTTCCTCAGATGCGGCATACAGCTTCGCCATTAGTTC
CGCTGCAATTTCGGTCGCCTGGTAGTCGCCGTGCTCGGCAACGATGTGAGAGAGCATTCGCTCCCCGGCATATTCGAGCAAC
ATCAGATTGTTCTCACGACCGAGCAACCGGACTGCTCCCCTCCCATTGCGCCATACCAGATAGTCGGCCCCGCGCAGTTCATC
AGCAATGTCTTCTATAGGTTTCAATCCCTTGACGATTGCAGGAGTCCCGTCTGGCAATGAAACTTTCCAAACGAGGCTGGAAA
AGGTGTCCGCAATGAGAACAGGTTGCGAAACGTGCCAATGAGCAGGAAAAACAGGCGGCATGAACATCAACCCCAAGTCA
GAGGGTCCAATCGCAGATAGAAGGCAAGGCGTTCGCGGTCGGGGGCTTCGATCCCCAATACATTGAATAGGACAGCGAAGG
CGCGCTCTGCTTCATCTGGCGCTGCCCAGTTCTCTTCGGCGTTAGCAATCATGAGTGCCAAATCGGCATAGCGATCTGCTGTTC
CGAGCCGCCCAAGGTCGATCAGACCCGTGCATTGAAGAGTTTTAGGGTCCACCATGAAGTTCGGCATGCAGGGATCACCATG
GCAAACAACCATATCGGTGCGCTCTTGGTCGAGCCGCACCGGTAGCTCTCGTTCGACACGAGCCAAAAGATCGAGCTGCGGC
GTACTCTTGTCCTCGTCCGGTAAGAAGTCGGGATTGACGGCATTGCGGGACACCACATCAACGGCGCGTCCGAACATTCGCG
ACAGCCTGCGCTCAAACGGACATTGATCAACCGATAGGCTGTGAACAGCGCCAAGTTGCTGCCCCATTGACGGCCACGCTTT
GAGCAAATCCGCTCCAGACAGATCAGCCGCCGGTACTCCCGGAATTGCCGTTATCACCAAGCATGCACCCTCCTGTTCCTCCT
GCCAGTTGATCACCTCGGGGCAAGCCACACCTCGACCTTTGAGCCAAATGAGGCGGTCACGCTCTCCAGCGAGCTCACCGC
GGCGGGAAGCAGGTGCGATTTTCGCGAAGGCATGCCCGTCACCACGTCGAAAAACAAAATCACCAGATTCTCCGCCTCTGA
CAGGCAACCAGTCAGAATGCGATTCACCAAAAAAAATATTAGTTCGATTCAATGGAGGTTCCTTCAGTTTTCTGATGAAGCGC
GGAGGTGGCTCAACCTGCGAAAAGAAACGAGTTGCTACGTAAGTCCGAGAACATGCTTTCCATGGTCTCTGAGCTCGCCTTG
ATGCCCGAGGCATAGACTGTACAAAAAAACAGTCATAACAAGCCATGAAAACCGCCACTGCGCCGTTACCACCGCTGCGTTC
GGTCAAGGTTCTGGACCAGTTGCGTGAGCGCATACGCTACTTGCATTACAGCTTACGAACCGAACAGGCTTATGTCCACTGG
GTTCGTGCCTTCATCCGTTTCCACGGTGTGCGTCACCCGGCAACCTTGGGCAGCAGCGAAGTCGAGGCATTTCTGTCCTGGC
TGGCGAACGAGCGCAAGGTTTCGGTCTCCACGCATCGTCAGGCATTGGCGGCCTTGCTGTTCTTCTACGGCAAGGTGCTGTG
CACGGATCTGCCCTGGCTTCAGGAGATCGGAAGACCTCGGCCGTCGCGGCGCTTGCCGGTGGTGCTGACCCCGGATGAAGT
GGTTCGCATCCTCGGTTTTCTGGAAGGCGAGCATCGTTTGTTCGCCCAGCTTCTGTATGGAACGGGCATGCGGATCAGTGAG
GGTTTGCAACTGCGGGTCAAGGATCTGGATTTCGATCACGGCACGATCATCGTGCGGGAGGGCAAGGGCTCCAAGGATCGG
GCCTTGATGTTACCCGAGAGCTTGGCACCCAGCCTGCGCGAGCAGCTGTCGCGTGCACGGGCATGGTGGCTGAAGGACCAG
GCCGAGGGCCGCAGCGGCGTTGCGCTTCCCGACGCCCTTGAGCGGAAGTATCCGCGCGCCGGGCATTCCTGGCCGTGGTTC
TGGGTTTTTGCGCAGCACACGCATTCGACCGATCCACGGAGCGGTGTCGTGCGTCGCCATCACATGTATGACCAGACCTTTCA
GCGCGCCTTCAAACGTGCCGTAGAACAAGCAGGCATCACGAAGCCCGCCACACCGCACACCCTCCGCCACTCGTTCGCGAC
GGCCTTGCTCCGCAGCGGTTACGACATTCGAACCGTGCAGGATCTGCTCGGCCATTCCGACGTCTCTACGACGATGATTTACA
CGCATGTGCTGAAAGTTGGCGGTGCCGGAGTGCGCTCACCGCTTGATGCGCTGCCGCCCCTCACTAGTGAGAGGTAGGGCA
GCGCAAGTCAATCCTGGCGGATTCACTACCCCTGCGCGAAGGCCATCGGTGCCGCATCGAACGGCCGGTTGCGGAAAGTCC
TCCCTGCGTCCGCTGATGGCCGGCAGCAGCCCGTCGTTGCCTGATGGATCCAACCCCTCCGCTGCTATAGTGCAGTCGGCTTC
TGACGTTCAGTGCAGCCGTCTTCTGAAAACGACAAACGATGTCAGCCAATAAGTTGTTGTAATAATCGACAAGTGTTTGTTGC
AACTCGCCGTCCTGTAAGACTGCATTATCAGTCTGATTCAGGGCGGCTATGGTGTCATTTATACACCATAAACCGATGACGGCT
GCACGTAAATCTGTGGATGCTCTTGATGGCGATTCAACGTTTGCTGTCCCACGCATGGCGACAGCATTGTTTAGCCCAGCAAT
AATCCCATTATAAGTTGGGGGGAGGGGCTCCGACAACTGCAAAGCTATCTTTTCTGTATTGTTTTGGATGTCGTCCGCAAGAA
TGGTATTCTCGATCAGTTCTCGAGCCAATAATGATGTTTCGAAGCAGACGGCCAACACCGTCTGCTTCGTTTCTGGTTCTTGTT
CTTCCACTGCCAGCGCGTTACTGATTGTTACGCACGTAAGTAGCAAAGTGAATGCGCTCTTGAGTCTCATCAACGGTTGCCTCC
ACAGTCTCGCTGGCACTGGAATCAATGTGAAAATTGTTCTGGATCGGATCAACAGGAATAAATCGATCACAGTCGAACTCTCT
TGGAATTATGGTCATGTGCAGTTTCGTTGCTGATGAAAGAGCTTGAATGTACAACTTCTCCCCACCAATGACAGCAATGCGTTG
ATCGGTATTCTCATACAGGTTGATCACAGCACGAAGATCAGGAACGACAGTAGCGCCTATGACATCTTCTTGTTTTAACGTCGA

GGAGATCACGAACGATTCGCGTTCGGGGAGAATTCCTTTCTCCTTGATTCGCTCCTCGGCGCCATCCTTTTCTAACTGCATGTC
ACGCATGTCAGTATACGTGTGTTTCCCCATAACGCACACGCGGATCTCTTTAGAAATCCGCGTAAATCGCGCCATGTCTTCTTTG
CATTTCCATGGAATCTTGCCGCCTTTCCCGAATCCCAACTTAGAATCCACAGCGACTATTAGCTCAAGTTGTGGGTGACTCATT
AAAACTCCAAACCAGTCGATGCTGGTTGTTCCAGATATTGCTTACGGAAGGTGGCCAGATCAATCAGTTTGATATTTGCCTTTC
CGCTGTTGTTGAGATCGTGCACTTTCTTCAATTTGCCGGAAGATCCGGAAGTGGATGCCGCGATCACCATGGTGGTCTTCGAC
GAGACTGATGATTGCACTTCACCACCCTCAGCTTCAATCAGCTTCTCCAAGGCCGCGTCACGAAAACCGGTGATCACGATCTT
CTCACCGGTAAAACGATCCCCCGAAGTGACCTGCTGTTTGAACCCGATGATTGATTGAATCTTGTTGAATAGCTCGGCATGTTC
AGCCATACCTGCAAGCACTTTTGCGGCAGTTTTGTAGCTGAATTTGTCGACGCTAACGATCTGCTCTTCGGTGATGGCATTGA
AATCATCCAAGGTCTTAATATCCAAGCCTTCACACAGAGCTTTCATCTTGCGGCGCCCAACGCCGCGCCCAAAGTGGGGCATC
GACCCCAACCAAACCGACAGGGGAACACTACTGGGTATAGGAAGTATAAACCACCTTTTTGCTCCTCATCCGAAGTATCTTAC
CTGAAATTCCCTCACTCGTTTACCGCTCAAGCCCCAATTTTAACTGCCGGTCCAGCCTAAACCGCTCTAATAAGGTTCGATTTG
GCGGTAAAATCTCTAGCCTGATAGCTCGAGAGATACAAACTGCCCCACCGCCCCGTTTAAAAGTTGGCAGTGTTGAGCAGTG
TTGGATTTGGGGTCGTCAGTCAAAGAGACGACTCTGTGATGGATCGAACAGGCTGGGAGTCAGTGGCGGCGCTCGTTCTGG
TGGCAGCTCACGCTGCTTGGCGGCATTCGCCTTGGCTGTTTTCTGTTTCAGATGCTTGAGAATCTGCTCAATGACCTTCGGATC
TTCGATGCTGGCAATCACTTTGACGTGACCGCCGCAGTGTTCGCAGACTTCAATATCAATATTGAAGACTCGCTTGAGGCGTT
GCATCCAGGTCATGGCGCGGTGGCGCTCTGCAGGACTCTTGTCACGCCAGTTAGTATCGAGACCTTCCGATTTGTCGGGCTTC
TTGCCCCGCTTGGCGGGTGTTACTTGAACTCGGTGTTTGCTGTTCGGTGCAAAGACGCCGTGGAAGCGTGTGAGGTTGACTC
GCGGCTTAGGTACCAACGCAGCGAGTTTGGCGATGAAGTCCAGCGGCTCGAAGATCACATGGGTGGTGCCATTGCGGTACG
GAGTTTTGAGCTCGTAACGCACCTGCCCATTGGCGGTTAATGCCAGACGTTTTTCTGAAACCGCTGGCCGACTAATGTAGCGA
CACAAGCGCTCAAGCTTATCCCGCTGATGCGCTTCGGCCATCACACCGGCGTGTAGCGAGAAACCAGCATGGTTGGCTACTC
GACTGCTTGAGTCGGCTTTATCCTCACGCCCTGGCAAGGTTTGCAGGGTGAAGACTTTGCGCCCTTGCTGGGGGCCGACGG
CAATGCGATACGTAACCGAAGCACCATGTAATTGAGTCAGCGTATCGTCTTCGCCCTCTTCCAGTGTCAACCACGTATTCTCGG
CATCACGCTCCAAAATCCCACGCTTTTCCATGCAGCGAGCGATGCGATGGCTGAGGGTGTGAGCGAGCGTATTCAGCTCATCG
TAAGTGGGTGCCTTGACACGATGGAAGCGTTGCTTGCCATAGTCATCTTCGGCATAGACACCATCGAGAAACAGCATGTGGTA
GTGGACATTGAGATTTAGCGCGGAGCCAAAGCGTTGGATAAGAGTCACTGAGCCAGTTTGTGCAGAGGCTTTGGTGTAACC
GGCTTTTTTGATCAGATGAGTTGAGAGTGTACGATAGACGATACTCAAGACCTGGCCCATCAGCTGGGGATGGCGAGCCAGC
AAAAAGCGTAGCTGGAAAGGAAAGCTGAGCACCCACTGGCGAATGGGCTCCTTGGGGAAGACTTCGTCTATCAGCAGCGCC
GCACTCTCGGCCATCCGGCGGGCACCGCAGCTAGGGCAAAAGCCGCGTCGTTTACAGCTGAAGGCGACCAGACGCTCGTGA
TGACAATCCTCGCAGCGAACCCGCATGAAACCATACTCCAGACGGCCACATTGGAGGAGGTCGTTGAATTCTTGTTGGATGTA
GCGAGGCAGGTGTTGACCTTGGGCTTCGAGTGAGGCTTTGAAGGCTGGGTAGTGCTGCTCAACCAGCTGGTAGAGCAGCG
TCTGGTCGGGTTGGTGGCGTTCGTAACCGTTTGTTTGAGTGGGCGATTGACTCGCCGTGGCGTTCCTTGCCAGCGACATGGG
TATCCTCCGCTGATACTGTGGTTATGTACAGTATCAGCGGCTTGCGTTCAGACGTCCAGTCTGGCCCTAGACATCGCTAAATGCT
TAACCCGCAATAGCCCTCACGAGTTGTTATCAGCCACTACCGGTTGAGCGAGAAGGTTTTGGGTTCAGGGTGCTATTGCTCCA
CCAATCACAATACTGAAGCCCCAACTGTTATCAGTTGGGGCTTTTTCTTGTCTGTTTGCGGCGGTTGCGTTTTATCGGTAGTCG
TCGAGCTCTGCACCATCCCACATAAGAGCTTAACGGTGCGATCTTCAACGCCATCACACAAAACTTTCTTTTTCACGCACAGTC
AACTTATTGGATGTTTTATTAACAACCCAAAAGGAGATATTTAGCGGGCGGCCGGAAGGTGAATGCTAGGCATGATCTAACCC
TCGGTCTCTGGCGTCGCGACTGCGAAATTTCGCGAGGGTTTCCGAGAAGGTGATTGCGCTTCGCAGATCTCCAGGCGCGTG
GGTGCGGACGTAGTCAGCGCCATTGCCGATCGCGTGAAGTTCCGCCGCAAGGCTCGCTGGACCCAGATCCTTTACAGGAAG
GCCAACGGTGGCGCCCAAGAAGGATTTCCGCGACACCGAGACCAATAGCGGAAGCCCCAACGCCGACTTCAGCTTTTGAAG
GTTCGACAGCACGTGCAGCGATGTTTCCGGTGCGGGGCTCAAGAAAAATCCCATCCCCGGATCGAGGATGAGCCGGTCGGC
AGCGACCCCGCTCCGTCGCAAGGCGGAAACCCGCGCCTCGAAGAACCGCACAATCTCGTCGAGCGCGTCTTCGGGTCGAAG
GTGACCGGTGCGGGTGGCGATGCCATCCCGCTGCGCTGAGTGCATAACCACCAGCCTGCAGTCCGCCTCAGCAATATCGGGA
TAGAGCGCAGGGTCAGGAAATCCTTGGATATCGTTCAGGTAGCCCACGCCGCGCTTGAGCGCATAGCGCTGGGTTTCCGGTT
GGAAGCTGTCGATTGAAACACGGTGCATCTGATCGGACAGGGCGTCTAAGAGCGGCGCAATACGTCTGATCTCATCGGCCGG
CGATACAGGCCTCGCGTCCGGATGGCTGGCGGCCGGTCCGACATCCACGACGTCTGATCCGACTCGCAGCATTTCGATCGCC
GCGGTGACAGCGCCGGCGGGGTCTAGCCGCCGGCTCTCATCGAAGAAGGAGTCCTCGGTGAGATTCAGAATGCCGAACACC

GTCACCATGGCGTCGGCCTCCGCAGCGACTTCCACGATGGGGATCGGGCGAGCAAAAAGGCAGCAATTATGAGCCCCATAC
CTACAAAGCCCCACGCATCAAGCTTTTGCCCATGAAGCAACCAGGCAATGGCTGTAATTATGACGACGCCGAGTCCCGACCA
GACTGCATAAGCAACACCGACAGGGATGGATTTCAGAACCAGACGCAGCCGCAGGCGCCGGAAACTAAACCGATGTCTGTA
GGGTTACCGCTGGATCTGCGTGAATTCCAGCAGCAGCAAGAGAAAGACTTTCTACAAACCAGCTTACAGCAGGCAAAATTTA
ACCAGAAAAAAGCCGCTGAATTACTGGGTCTGACTTACCATCAGCTTCGCGCTTTGCTGAAAAAACACCAGATTTAACGCAC
ATTTGCAGATGTTATATTGGCGGATTTGACGCATAACCTCATCAGGGTTTACCATGACGCCATTACTGTATAAAAAAACAGGCA
CAAAATATGGCTCTGGCACTCGTTGGCGAAAAAATTAACAGAAACCGCTTCACCGGTGAGAAAATTGAAAATAGTACATTTTT
TAACTGTGATTTTTCAGGTGCCGACCTGAGCGGCACTGAATTTATCGGCTGTCAGTTCTATGATCGTGAAAGCCAGAAAGGGT
GCAATTTTAGTCGTGCGATGCTGAAAGATGCCATTTTTAAAAGCTGTGATTTATCCATGGCGGATTTTCGCAATGCCAGTGCGC
TTGGCATTGAAATTCGCCACTGTCGTGCGCAAGGCGCAGATTTTCGCGGCGCAAGTTTTATGAATATGATCACTACTCGCACCT
GGTTTTGCAGTGCATATATCACTAACACAAATCTAAGCTACGCCAATTTTTCGAAAGTCGTGTTGGAAAAGTGTGAGCTGTGG
GAAAACCGTTGGATGGGGGCCCAGGTACTGGGCGCGACGTTCAGTGGTTCAGATCTCTCCGGCGGCGAGTTTTCGACTTTC
GACTGGCGAGCAGCAAACTTCACACATTGCGATCTGACCAATTCGGAGTTGGGTGACTTAGATATTCGGCGCGTTGATTTACA
AGGCGTTAAGTTGGACAACTACCAGGCATCGTTGCTCATGGAACGTCTTGGCATCGCGATTATTGGCTAGTCTTCAGGGAGC
GGTGAATATTCCGCCCCCTGCACAGCTTTTTACCCTTCAGGCATCGCTGAAGAGTGGTGTGTGGAAATTTTCCACTCTTTACCG
TCCCATGCATAAGTGAATGTGTAGCGGGCTGAAACCGTTGATTTGTCCGCGAAGGTAAATGTGTAGGTACCGGTGTCTATCGC
CTTATTACACCCCAGGCGAATGGTACGCATGTCAATTTTCCCGGTCGGCTTTTTCGCAAGAAAATGCTCGAAATAAGGCACTG
TTGCAAATAGTCGGTGGTGATAAACTTATCATCCCCTTTTGCTGATGGAGCTGCACATGAACCCATTCAAAGGCCGGCATTTTC
AGCGTGACATCATTCTGTGGGCCGTACGCTGGTACTGCAAATACGGCATCAGTTACCGTGAGCTGCAGGAGATGCTGGCTGA
ACGCGGAGTGAATGTCGATCACTCCACGATTTACCGCTGGGTTCAGCGTTATGCGCCTGAAATGGAAAAACGGCTGCGCTGG
TACTGGCGTAACCCTTCCGATCTTTGCCCGTGGCACATGGATGAAACCTACGTGAAGGTCAATGGCCGCTGGGCGTATCTGTA
CCGGGCCGTCGACAGCCGGGGCCGCACTGTCGATTTTTATCTCTCCTCCCGTCGTAACAGCAAAGCTGCATACCGGTTTCTGG
GTAAAATCCTCAACAACGTGAAGAAGTGGCAGATCCCGCGATTCATCAACACGGATAAAGCGCCCGCCTATGGTCGCGCGCT
TGCTCTGCTCAAACGCGAAGGCCGGTGCCCGTCTGACGTTGAACACCGACAGATTAAGTACCGGAACAACGTGATTGAATGC
GATCATGGCAAACTGAAACGGATAATCGGCGCCACGCTGGGATTTAAATCCATGAAGACGGCTTACGCCACCATCAAAGGTAT
TGAGGTGATGCGTGCACTACGCAAAGGCCAGGCCTCAGCATTTTATTATGGTGATCCCCTGGGCGAAATGCGCCTGGTAAGC
AGAGTTTTTGAAATGTAAGGCCTTTGAATAAGACAAAAGGCTGCCTCATCGCTAACTTTGCAACAGTGCCGTACCGACGGTG
ATATGGGGCAAATGGTGGTCACCATCCTGTCGGCTGTGGCACAGGCTGAACGCCGGAGGATCCTAGAACGCACGAATGAGG
GCCGACAGGAAGCAAAGCTGAAAGGAATCAAATTTGGCCGCAGGCGTACCGTGGACAGGAACGTCGTGCTGACGCTTCATC
AGAAGGGCACTGGTGCAACGGAAATTGCTCATCAGCTCAGTATTGCCCGCTCCACGGTTTATAAAATTCTTGAAGACGAAAG
GGCCTCGTGATACGCCTATTTTTATAGGTTAATGTCATGATAATAATGGTTTCTTAGACGTCAGGTGGCACTTTTCGGGGAAATG
TGCGCGGAACCCCTATTTGTTTATTTTTCTAAATACATTCAAATATGTATCCGCTCATGAGACAATAACCCTGGTAAATGCTTCAA
TAATATTGAAAAAGGAAGAGTATGAGTATTCAACATTTCCGTGTCGCCCTTATTCCCTTTTTTGCGGCATTTTGCCTTCCTGTTT
TTGCTCACCCAGAAACGCTGGTGAAAGTAAAAGATGCTGAAGATCAGTTGGGTGCACGAGTGGGTTACATCGAACTGGATCT
CAACAGCGGTAAGATCCTTGAGAGTTTTCGCCCCGAAGAACGTTTTCCAATGATGAGCACTTTTAAAGTTCTGCTATGTGGTG
CGGTATTATCCCGTGTTGACGCCGGGCAAGAGCAACTCGGTCGCCGCATACACTATTCTCAGAATGACTTGGTTGAGTACTCA
CCAGTCACAGAAAAGCATCTTACGGATGGCATGACAGTAAGAGAATTATGCAGTGCTGCCATAACCATGAGTGATAACACTGC
TGCCAACTTACTTCTGACAACGATCGGAGGACCGAAGGAGCTAACCGCTTTTTTGCACAACATGGGGGATCATGTAACTCGC
CTTGATCGTTGGGAACCGGAGCTGAATGAAGCCATACCAAACGACGAGCGTGACACCACGATGCCTGCAGCAATGGCAACA
ACGTTGCGCAAACTATTAACTGGCGAACTACTTACTCTAGCTTCCCGGCAACAATTAATAGACTGGATGGAGGCGGATAAAGT
TGCAGGACCACTTCTGCGCTCGGCCCTTCCGGCTGGCTGGTTTATTGCTGATAAATCTGGAGCCGGTGAGCGTGGGTCTCGC
GGTATCATTGCAGCACTGGGGCCAGATGGTAAGCCCTCCCGTATCGTAGTTATCTACACGACGGGGAGTCAGGCAACTATGGA
TGAACGAAATAGACAGATCGCTGAGATAGGTGCCTCACTGATTAAGCATTGGTAACTGTCAGACCAAGTTTACTCATATATACT
TTAGATTGATTTAAAACTTCATTTTTAATTTAAAAGGATCTAGGTGAAGATCCTTTTTGATAATCTCATGACCAAAATCCCTTAAC
GTGAGTTTTCGTTCCACTGAGCGTCAGACCCCGTGTTGACACCGGCGTACCCTCGGTGCTATCTTCGCGCCCCAATAGTCGGG
GCTTGGCCAGGACTTCCTGAGGCCGTCCGTAACTCCACCTTCAAACAAGGAATATCGTTGATGTCACTGTATCGCCGTCTAGTT

CTGCTGTCTTGTCTCTCATGGCCGCTGGCTGGCTTTTCTGCCACCGCGCTGACCAACCTCGTCGCGGAACCATTCGCTAAACTC
GAACAGGACTTTGGCGGCTCCATCGGTGTGTACGCGATGGATACCGGCTCAGGCGCAACTGTAAGTTACCGCGCTGAGGAG
CGCTTCCCACTGTGCAGCTCATTCAAGGGCTTTCTTGCTGCCGCTGTGCTGGCTCGCAGCCAGCAGCAGGCCGGCTTGCTGG
ACACACCCATCCGTTACGGCAAAAATGCGCTGGTTCCGTGGTCACCCATCTCGGAAAAATATCTGACAACAGGCATGACGGTG
GCGGAGCTGTCCGCGGCCGCCGTGCAATACAGTGATAACGCCGCCGCCAATTTGTTGCTGAAGGAGTTGGGCGGCCCGGCC
GGGCTGACGGCCTTCATGCGCTCTATCGGCGATACCACGTTCCGTCTGGACCGCTGGGAGCTGGAGCTGAACTCCGCCATCC
CAGGCGATGCGCGCGATACCTCATCGCCGCGCGCCGTGACGGAAAGCTTACAAAAACTGACACTGGGCTCTGCACTGGCTG
CGCCGCAGCGGCAGCAGTTTGTTGATTGGCTAAAGGGAAACACGACCGGCAACCACCGCATCCGCGCGGCGGTGCCGGCA
GACTGGGCAGTCGGAGACAAAACCGGAACCTGCGGAGTGTATGGCACGGCAAATGACTATGCCGTCGTCTGGCCCACTGGG
CGCGCACCTATTGTGTTGGCCGTCTACACCCGGGCGCCTAACAAGGATGACAAGCACAGCGAGGCCGTCATCGCCGCTGCGG
CTAGACTCGCGCTCGAGGGATTGGGCGTCAACGGGCAGTAAGGCTCTGAAAATCATCTATTGGCCCACCACCGCCGCCCTTG
CGGGCGGCATGGATTACCAACCACTGTCACATTTAGGCTAGGAGTCTGCGCGGCAGAGCCGTGTGACCGGTTTTCTGTAGAG
CACTGACGATGGCGGCGGCGCTCTCTGCAATTGGCAAGGCGTCGGCGCCAAGGATACCAATCTTGCGGCGCGCGGCGTGTT
ATGACGACTGGGGTGCATTTGAGCCGCCCCATTTAACCTTCGCCCTCACAGATACGCCATTCGCCTCAGATTTAGCGCCATGCA
GACGAGCTTCCACTCGGCTTGCACCTTGTCCAGGCCCCTCATGCTGAACTGACGCAATCCCATCACCGCCTTGATCCAACCATT
CGGAGCCTCCACGATCGACTTGCGCCGGCGGTAAGCTGCATCGCCTTGCTCCGTTTTCAATTTCGCCGCAATCGCCGCCGTAT
GCGGATGGGTCTTGGCATTGACCTTGGCATCTTCACGTCCCTCGCGGCCGAGGGCAACGATGACATCGCCGTGGTGATCGGC
GACCTTTGCCAGAACAGCCTCACTACGGAATCCCGCATCCGCCAGCGTCTGGGCCGGCATTTCTCCGGTGTTGGCCTGAACT
GCTGCCAGCATGCCCAGCAGCGCCTGACTGTCCGCGGCGCAGTTGGTCAACTCCGCCGCCACGATGATCTGGTGCTCGGCAT
CGACCGCTGTGTACCCGTTGTAGCTCTGCTCGGAGCCACCACCGGCGTGTTTCATGATCCGGCTGTCCGGATCGGTGAAGCTT
TCCTGATCACGGTCATCCGGCACACCAAACTCGCGTTTGTACGAGCCACCGCCCTTGTCCGAGCCATCCGGATGGCGAGGCC
GGCGGCCATCGTCTTCGCTGCGCCCCCGGGCCTGGTCCGCTTCACGCTGGCGCGCTTCCAGGGGTCGGTTCCGGCTGAGGG
CGAAATGACACCCTAAGCGTTAGCTCTGTGTCGTTGCACGATGTCAGCGACGGTATTCTTGCTGATACCGAGCTCGCGTGCGA
TCCAGCGATAGCTGCGTCCCTCGGCCCTCATCGCAACCACCTTAGGCAAAAGTCGGTCTGATTTTGGTCGCACTCCGGCCTGA
CGACCAAGCCTCTTACCACGTGCCTTCGCAACAGCAAGGCCTGACTTGACCCGCTCGCTGATGAGATCCCGCTCAAACTCCGC
AATGCCGGAAAGAAACGTCGCCAGCATTCGTCCATACGGCGACGAAAGATCGAACGCCATTCCATTCATGGCTATCACGGAA
ACCTTCCAGTTCTCCAGTTCACGTAGCGTATTGAGCAGATCGAGCGTCGAGCGCCCCCACCGGGAAAGCTCAGTGACCAGGA
TTGCATCAATTTGTCTGGACTGGGCAAGCGCCAGGACTTTCTTTCGCTCGGCCCGGTCGAGTTTAGTTCCTGAACCTGTTTCC
TTAAATATTCCCACCACGTCGTAGCCGGCACGGCCGGCGAAGGCTCGCAGATCAAATTCCTGGCGTTCACAAGACTGATCCGC
TGTTGAAACCCGGCAGTAAATGGCGGCACGATGTCCCAATTGAACCCTCCTGGATTTTTGTATCGGAACGCCCTGATTTATATG
GGCTGGCTGTTGTCCAAAACAGACTATACTTCAAAAGGGACGAATTTGTATGTCACGACGCCATATTTTCACCGAACGGCAGC
GAGCAGCGCTGTTCGATCTGCCCACGGACGAACTGTCGCTACTGAAGTTCTACACGCTGGGCGATGATGACCTGGAAAACAT
TAGGCAGCGCCGCAGACCGGAAAACAGGATTGGCTTTGCCCTGCAACTTTGTGCCTTACGATATCCGGGCCGTGCACTGGCT
CCTGGTGAGATGATCCCGCGTGAAGTCCTTTCCTTCGTCGGTGCTCAGCTTGGAGTTCCGGCTGATGCGCTTCTCACTTATGCC
ACACGGCGCCAAACCCGTCAGCAGCACATGGACACGCTGCGCGAAATTTACGGCTACAAGACCTTCACGGGCCGTGGTGCC
CGTGATCTGCGGGAGTGGACTTTCGGCCAGGCCGAAGATGCCAGATCAAACGAGGATCTTGCTCATCGTTTTATTGTGCGGT
GTCGGGAAACTTCCACCATTCTGCCCGCAGTATCGACAATCGAGCGCTTGTGCGCGGATGCTCTGGTCGCCGCTGAGCGGCG
GATTGAAACGCGGATTGTGGAAAATTTAACAGCGGATGTTCGCGATCACCTGGACAAACTTCTGAGTGAAATGCTCGCCGGC
AATATCAGTCGTTTCATCTGGCTTCGCAACTTCGAGGTTGGTAACAACTCGGCTGCTGCTAACCGTTTGCTCGACAGGCTCGA
ATTTCTGCGTACCCTGAATATCAATCATAGTGCTTTGGCCAGCATACCTGCCCATCGCATTGCCCGGCTGCGTCGGCAGGGTGA
ACGCTACTTCACCGACGGTTTGCGTGACATCACTTCGGACCGCCGCTGGGCGATCCTTGCCGTCTGTGTTGTGGAGTGGGAA
GCGGCGATTGCTGATGCCATAGTCGAAACCCATGACAGGATCGTAGGAAAAACCTGGCGGGAAGCGAAGCGCCAGCATGAC
GAAACAATTTCCGGCTCTAAAGCCACACTCACGGATACGATCCGTACCTTCACCGCGCTGGGAGCTTCGTTGCTTGAGGCCCG
CAGTGACGGAACCCCGCTGGAGATGGCTGTCGCCAGTTCGGTTGCATGGGACCGGCTCGCTCAACTGGTAGCGACAGGGAC
TCAACTCAGCAACACGCTAGCCGATGAGCCTCTTGCATATGTCGGGCAGGGATACCATCGCTTTCGTCGTTATGCGCCCCGCAT
GTTGCGCTGTCTGAAGCTCGAAGCCGCGCCGGTCGCCGGACCATTGGTAGCAGCAGCTTTGTCGATCGGAGAGATGAAAGG

TGTTGCATCGCCAGAAAGGCGTTTCCTGCGGCCCAGCTCCAAATGGAACCGTCATTTACGAGCTCAGGAAAAAGGAGATACC
CGTCTTTGGGAAGTGGCGGTACTCTTTCACCTCCGGGATGCTTTTCGTTCCGGAGATGTCTGGCTCGCTCATTCGCGCCGCTA
TGGTGACCTCAAGCAGGTACTGGTGCCGATGATCGCGGCGCAGGAAAATGCAAAACTGGCCGTGCCTTCCAACCCACAGGA
TTGGCTGGCAGACAGAAAGGCGCGACTCACGATCGCTCTTAAGCGGCTGGCCCGGGCTGCCCGTAACGGCACTATTCCGCA
CGGTAGCATAGAAGATGGAACGTTGCGGATCGACAGGTTGACAGCAGACGTGCCGGATGGTGCCGAGGCACTCATACTGGA
TCTGTATCGCCGAATGCCGTCCGTTCGGATTACCGACATGCTGCTTGAAGTTGATGCAGCCCTTGGTTTCACAGATGCGTTTAC
CCATCTGAGAACCGGGGCTCCATGTCGCGACCGGATCGGTCTGCTCAACGTCCTGCTCGCTGAAGGGCTCAATCTGGGCCTG
CGTAAGATGGCGGAAGCTAGGGGTTTGAGGGCCAATGGAACGAAAACGTACGCTAAGAAGTTAATTCATTGTTTAAATTGAA
ATTTAGTTCTCTTAGTTCCCCCTTCAAAATATCCTCCGGTAGCGTAAACGTATAATGCCCCAGCATATTGATATGCCCGTGCATCA
GTGGGGACAACCGGGCGATATCTTCAACCCCGATTTCTTCTCCATTACTGCGTATCCAGCTCAGGGCTTCCTGCATATAAAGTG
TGTTCCACAGTACCACTGCGTTAGTGACCAGGCCCAGTGCCCCCAGCTGATCTTCCTGACCTTCACGATAGCGCTTTCTGATCT
CACCGCGCTGCCCGTAGCAGATCGCCCTCGCCACAGCATGGCGGCCTTCCCCCCGGTTTAGCTGCGTCAGGATCCGCCGACG
ATAATCCTCATCATCAATATAATTAAGAAGGTACAGCGTCTTGTTGACGCGCCCCACTTCCATGATCGCCTGTGCCAGCCCTGAT
GGGCGCGAGCTTTTCAGCAAAGAGCGAATGAGTTCTGAAGCATGAATGGTGCCCAGTTTCAGCGAACCGGCGGTTCGCATC
ATCTCATCCCACTGATCCTCGGCTTTCGACAGATCGGCACAACCACGTGCCAGTTCGTCCAGTGCACCGTAATTTGCCGATTTA
TCCACTCGCCAGAATACCGCTTCACCGGCATCGGCAAGCCGGGGGAAAACTGGTATCCCAGCAGCCAGAAGAGGCCAAAAA
TAATGTCGCTGGTACCGGCTGTGTCTGTCATGATCTCAACCGGATTCAGCCCTGTCTGCTGCTCCAGAAGGCCTTCCAGCACA
AAAATGGAATCTCGTAATGTGCCGGGGACAACGATGCCGTGGAATCCAGAGTACTGATCAGAGACGAAGTTGTACCAGGTGA
TGCCACGTCCGGAACCAAAATATTTCTGTTAGGTCCTGAATTGACGGTTTTCACCGGCGTGACAAAGCGCATGCCATCAGCT
GAAGCCACTTCGCCACCACCCCAGCGGCCAGCAAGCTCCAGTGTGGACTGAAAATCAACCAGGCGGGCATTGGCGCTGACC
AGCGTTTCTGCCCGGAGGTAATTCTGTTTTACCCAGCTAAGCCGGTGGCGCGTCAGCGCCGGTATATTGTGCTTTATCAGCGG
TTCCAGCCCGATATTGCAGGCCTCTGCCATCAGGACCGCGCACAGGCTGATGTGCAGATCCTGTGCCCGGGCCCCGGATTCA
CTGACATGCGTAAACTCACGTGTAAATCCCGTTCTGGCATCTATTTCAAGCAACAGTTCCGTCAAATCTACTGGCGGGAGTAGC
AGCCTTACCCGACTGTTGAGACGATGCAACGATGGTGGCTCCTCCAGTTTCTCCAGGCTGCTGATAGTCAGGGAAGGATGTT
TACCGTCATGGCAAATATGAACCTCCGCATTTCCTTCAAAGCGGGATGCGACGGTTTTCCAGGTTTCATCTAGCTGGACCGCC
AACTGTTGCACGCCTTTATGTCCATCAGTGGGATGTCCCAGCGCCCGGCAAACGGGGACCCGCTGAGCCTGCCATTCTTCCCC
CTGCAACAGCTTCTCGCGGGGATTTCCCCAGCGATCGCTGTTTTCGAGCCAGATGTCCCTTCGACGTAGTGCATCCTGAAGGC
GCTCCAGCAGACAAAGCGAGTAACCGGCACGCTGTATCCGGCCCTCCGCATCGTATACCAGGCGTTTCCAGGGGCCGGTAAT
AATATGTTCAGGCGCATTGTCCAGGATGCGCTTTTTCGAGCCGTTCAGTTCGGTCAGATAATGAATGGCGGACAACGTATGTT
CTCCGGCTGGCGCTGCCTGGAAATGCAGGTCGCGCAATACCGCCGGAAGAAAGCGCTTTACCCGCCCGTACTGCTCCACCAT
TTCGTCATGGAAATTGTTGTTCTGAGGCCGGGCCAGCTCATTTACCTTGCTGACGGATTCTGCCAGCCTGCTTTTCGGTATGCT
GTTGAATATGGTCTCCCTCAGTTCAGCATCGTCAGCCTGTTCATCCAGCAACAATGAACATGCCCGCGCCAATATTAGCGCAGC
ACGATCAAGATCTTTAAGCGTCCTGAGCCGTTTTTTCTGCCCCGTTTTCTTCGCCGCACGAGTGATATCCAGTATCAGCATATCG
AGCACATCAACGGCCTCATCCAGCGCCGCAGTCTCCTGCGCTTTAACGAATGCTGTGAGTACGGCCAGTTTTCTCTGCTGTGG
CATTCGCGCGATATATTTTACCGACGCCATGCCTGCGTAACGGGCCAAATTACGCAGTTGTATAGCAGGCAGACCGGTAAAATT
CAGTCGGGAAAATTCCAGGTTTCGCAGCCGGATATACCGCTCCAGCGCTTCGGTAAATGCCGGGCCGCTGACGGTGACCGGT
CCCTTTTTCAGTTGCTCCAGCGGTGAAGTACGCTGCCCCTCAGGAATGTCCAGAAGCTCCGTCACGCGGGCGGTCTGCCAGC
TGTCCGGCAACGCGGCCAGTTTCTTCCACAGCCGCTGATTTGCCCGTTCGCGTATTTCACTGACGAGACGTACAAGTGTGGTT
GCTCCGGGCAGCAGGACCTTATTCTGAAGCAGCCACGCGGTGGCAAAATCAAACATAAGGCCGGGTCGCTCATTACTGAGCC
ATGCGCGGGTGTACAGCAGGCGCTTCAGGCGGAAAGACCAGGGGAAATCGCCAAATTCATGGTAACCGTAATATTCCTTAAT
CAGCGCAGTATGCTCTCTGAGGGTGGTAACCCGTTCTGCGTAGCGGGTAAGGACTTCCGGACGGCGAATATTAAGCTGCACC
GCGACAAATTGCTGAACACCTGGCAGAACCCGGGTTAAGTCTGTCAGAAAGGTGCCCAGAAAACGGGCGGTGGTGAGCTG
AAGCGCAATTCCCAGCCGGTTATGCCTTCCCCGCCGCTGGTTAATGAAGGCAAGATCCCGCTCATCAAGATGAAAATAGCGCG
CCAGCTGCACGTCATTGGGTTCTGCAACATAGCGACCATAATTCAGTTCCTGCTCAGTGGTCAGAAAATCGACGGGCATACCG
GCCTCCCTGCCTGATGGTCATGAATTAACAATTTCGCAACCGTCCGAAATATTATAAATTCTCGGACACACTAAAATGGTGTTGT
CTAGGTGTCTATTAAATCGATTTTTTGTTATAACAGACACCCACTGTCCGATATTTGATTTAGGATACATTTTTATGCGACTTTTT

GGTTACGCACGGGTATCAACAAGCCAGCAGTCCCTCGATATTCAGATCAAGGGGCTTAAAGAGGCGGGGGTGAAAGCCAGT
CGCATATTTACCGACAAAGCATCCGGCAGTTCTACCGACAGGAAAGGACTGGATCTGCTGCGAATGAAGGTGGAGGAAGGT
GACGTCATCCTGGTGAAGAAACTCGACCGGCTTGGCCGCGACACTGCCGATATGATCCAACTGATAAAAGAATTCGACGCTC
AGGGAGTCGCGGTCCGATTCATAGACGACGGGATCAGCACCGACGGTGAAATGGGGAAAATGGTTGTGACCATTCTTTCAG
CTGTAGCCCAGGCCGAACGCAGGAGGATTCTGGAGCGTACGAACGAAGGACGGCAGGAGGCCAGGCTGAAAGGTATCCG
GTTTGGCCGAAAGCGCATCATCGACAGAAATAGTGTACTGGCACTTCATCAGCAGGGAACTGGTGCAACAGATATTGCCCGC
CGGCTCAGTATTGCCCGTTCCACTGTTTATAAAATTCTGGAAGATGAGAGCCGGGTTAATCTGAGCAAAATTTGAGGACGAAC
ATGAGTGATGTAAAGGCATTCAACAATTGCATGAGTGTGTTCTGGCGACAGCATGAAGCCGAATTCTCTCGCTTTCTGACATC
GAAGACAGGTGATAGCGAAAAAGCAGCAGACCCGGAGAAGACGAAGGTGATTATCGTGACGCTTGCCGAAACGACACCTG
TACTGGAAGCCGCTAACCTGCAGCAGGATCTACGCCGAGCTGGCATCGAGCCGTGGGCATGGGTCGTTAATAACAGCCTGGC
AGCTGCTCAACCGTCTTCGCCTTTCCTGAAGATCAGGGCGAACCGCGAGCTGCCTCTCATCTCTGATGTGGAAGAGCAGTATG
CAAAGCGTATTGCATTAACAGCGCTACAGAGCGAAGAGCCGGTTGGTATTGACCTGCTGGAAGAAATGGCGAAGTAATAATG
AAGGGGGCAAATGCCCCCTTCGTCTTTATTTCAGTCTTTTACCTGAATCATCAACGACTTTTTCACCGTCTTCCTTGGTGAAAG
CCGCTTTCTGGGCATCTGGAAGGATATCGAGAACAACCTCTGAAGGACGGCAAAGTTTAGTTCCCAGTGGCGTCACTACGAT
CGGACGGTTGATCAGAATCGGATGCTGTAGCATAAAGTCGATGAGCTGGTCGTCAGTAAATTTATCTTCTGCAAGACCCAGTT
CCTCATACGGCTCGACGTTCTTACGCAGCAAAGCCCGGACGGAAATGCCCATATCCGCAATGAGTTTGAGCAGCTCATCGCGT
GAAGGTGGAGTCTCAAGGTAAAGAATAACGGTCGGCTCGATACCGCTGTTGCGGATCATCTCCAGCGTATTACGCGACGTGC
CGCAGGCTGGGTTGTGATAAATGGTGATGTTGCTCATATCAGTATCTCATTACAAAGTGAAAGAGAGACGTAGCGCCAGCGCG
GCCAGCGTTACAAACAGCACAGGCAGAGTCATGACGATCCCGGTGCGGAAATAGTATCCCCATGTGATGGTCATGTTCTTCTG
TGAAAGGACATGCAGCCAGAGCAACGTTGCCAGGCTACCAATAGGTGTAATTTTCGGTCCAAGATCGCAGCCAATCACGTTG
GCAAAAATCATCGCCTCTTTGATAACGCCTGTTGCAGTGCTGCCATCAATAGAGAGAGCACCAACCAGCACGGTAGGCATATT
GTTCATGATGGAAGAGAGGAAAGCCGTCAGGAAGCCAGTACCAAACGTCGCGGCCCAAAGTCCTTTATCTGCCAGCACGTT
CAACACACCGGAGAGGTATTCTGTTAGTCCGGCATTACGCAGACCATAGACCACCAGATACATTCCCAGAGAGAATATGACGA
TCTGCCATGGCGCACCGCGAAGCACTTTGCCAGTGTTAATGGCATGGCCTCGCTTCGCCACCGCAAACAGAATGACAGCCCC
TACAGCAGCAATCGCACTGACCGGGATACCCAACGGCTCAAGGACGAAAAAGCCAACCAGAAGAAGGATCAACACTATCCA
GCCGGTTCTGAAGGTTGCCAGATCTTTAATCGCTTTTGCCGGTGCTTTCAGGAGAGCCAGGTCGTAAGTTGGCGGAATATCTT
TGCGGAAGAACAGATGCAGCATCACCAGCGTGGCGATAATGGCTGCGATATCCACCGGCACCATTACCGACGCATACTCAGT
GAAACCCAGCCCAAAGAAGTCTGCAGAAACGATGTTAACCAGGTTGGACACGATAAGCGGCAGACTGGCCGTATCGGCAAT
AAACCCGGCGGCCATGACAAATGCCAGCGTAGTGCCTTTGCTGAAACCTAATGCAAGCAGCATGGCGATAACAATTGGCGTC
AGAATCAATGCCGCACCATCGTTGGCAAACAGTGCCGCCACCGCTGCGCCGAGCAAAACGATATAGGTAAACAGCAAACGCC
CGCGACCGTTACCCCAACGTGAAACGTGCAGCGCCGCCCATTCGAAAAAGCCCGATTCATCGAGCAGAAGGCTGATAATAAT
CACGGCAATAAAGGTCGCCGTCGCATTCCAGACAATATTCCACACTACAGGGATATCACCAAAATGGACTACGCCAGAGATCA
GCGCCAGTACCGCGCCCAGCGTCGCACTCCAGCCGATCCCTAATCCCTTCGGTTGCCAGATAACCAAAACGATGGTCAGGAC
AAAAATAGCGCCTGCCAGTAACATAAATCCTCCAGACAGGACGGCTTTGCCGCCCTGTATATACAAAAAAAATTAAACAACCA
GCTCTCTGAGTTTCTCGATGCCAGTGGGCTCTGCAGCCATTACCGGTACTAACGCTATGCGGCTTGCATGCTGGTTTTTCACAA
CCTCGATTTGAGGACGCTCTTGTAGGGCGCGCTGGCAAAGCAGCGGCGATTGCGTCTGTGCAATCGAAAGGCTGTTATTGAT
AATCCAGCCCCAGGGATGAATCCCCGCACGTTCAAGATCGGACTGCAGGTTTGCCGCTTCCAGTACAGGTGTGGTTTCAGGC
AGAGTGACCAGCAAAACTTTGGTACGTTCCTGGTCCTGAAGCTGCATCATCGGAGTGGTAAAATGACCTTTATCCCCCATCTTC
CTGGCAATCTCACGGTGATAAGCCCCGGTAGCATCAAGCAGTAATAGCGTATGCCCGGTAGGTGCCGTATCCATGACCACAAA
CCGTTTGCCAGCTTCACGAATTACGCGTGAAAACGCCTGGAACACCGCAATCTCTTCTGTGCAAGGAGAACGTAAATCCTCTT
CGAGCAGCCGTTTCCCAGCCTCATCCAGATCCCTGCCTTTCGTCTCAAGAACATGCTGACGATAGCGTTCAGTTTCATCGTGAG
GGTTGATTCGGCTGACCTGCAGATTTTTGAGGCTGCCATTGAGCGTTGTACTGAGATGTGCAGCAGGATCTGAGGTGGTGAG
ATGCACATCAAAACCCTTATCCGCCAGACTGACGGCGATAGCCGCAGCCATTGTGGTTTTGCCCACACCACCTTTGCCCATCA
GCATAATCAAACCGTGTTCACTGCGGGCGATATCATCAACCAGGACAGACAACGACATATTCTCTGGCTTGTTCTGTGGGCTTT
GTTCAGGAAGCGATGTTATCTCAGCGTGTTCGTTGAGCAGGCCTTTCAATGCGGATACACCAACCATGTTCAGCGGCTGCAG
GTATAAGTTGTCTGTCGGCAAATCAGATAACCCGGCAGGAAGATTTGCCAGCGCCTCTTGCTCCCGTTGCCATATTGCAGCGG

CCAGTGCATCACGTTCTGCTTCGCTCGCAGGCAGGACACCGTTAATGACTAAATACTGATTTTTAAGGCCAATAGCTGATAATT
CGTCATGAGTACGAGCGACTTCCTGCAGTGTTGATTTTTGCAATCGCGCGACCAGCACAAGGCGAGTTCGTTCAGGATCGGA
TAATGCCTCAACCGCATGAGCATATTGCTCGCGTTGTTTTTCCAGCCCAGCCATTGGACCAAGACAGGAAGCACCATCCGGGT
TACTTTCGATGAAGCTGCTCCAGGCTCCGGGAAGCTGAAGAAGGCGAATAGTGTGGCCAGTTGGGGCAGTATCAAAAATGA
TGTGATCAAAGCGGGTCAGCAGAGAAGCGTCAGTCAGTAAGCCAGTAAACTCGTCGAATGCAGCGATCTCTGTCGTACAAGC
TCCTGAAAGCTGTTCACTGATGCTGTTAACAACGTCATCAGGCAAAAGACCTTTAATAGGGTCAACGATTCTGGCGCGGTATT
GCTGGGCGGCGTCCTGCGGGTCAATCTCCAGAGCCGAAAGACCAGACACAGCAGTCACGGGCTGGATAGTGTTACCGATAG
TCTGATCGAATACCTGACCGACGTTAGAAGCCGGGTCGGTACTGACAAGCAGAACACGCTTTCCCAGTTCAGCCAGACGTAT
AGCCGTTGCGCAGGAAATCGAAGTTTTACCTACACCTCCCTTACCGGTGAAAAACAGGTAAGACGGGATATTCTTTAAGAATT
TCATATGTCCTCCTGACATACTCAACAACAGGAAGTTTTACCACCACAGCAACTGGTGGAAGCTAAACCCACCTTTTCCAGCG
GTATACCAAACCAGCGAGCCAGTTCAGCGCGTTTTGGGTATCGCCCGGCCATCACCGTTTCACCATCAAGCAACAGCAGCGG
AAGCCCTTCAGCTCCAGAAGCCTCAAGGAATGCTTTCGCTTTTTCGTTCTCAACGAAGCTCATAGGCTGCTGCGCCAGGTTAT
AACGTTCGATCTGAACCCCACGCCCTTTCAGCCATTGCACATCAGCAGAAAAATTGACCAAAACCTGATCGACATCTGAACCA
CAAACGCCGGTACTGCAGCACATCGCCGGATCAAACACCGTTAACATTTTCATCTTTAACACCTCACATTCGTAAAAACATATAT
GTACAGGCAAAATTTTTAGATACAAACAGCCTTACCGCTGCCAGAGCAGTTGGCCGATGCCAGCTTACGGGCGATGGCCTGT
ACGTCGTCCTGTTGACTTAACCAGGCCTGCTCAATCACCAGGGCAGCCCAGGAAGGAATATGCGGGGATAAGCGATAATGAA
CCCATTTCCCCTGCTTGCGATCCAGCAACAGGCCACTTTCCCGAAGCATCGCCAGATGGCGGGAGGTCTTGGGTTGTGATTG
TTCCAGCGCTGTGCAGAGATCGCATACGCACAGCTCCCCCATCTCCCTGAGCAGTAGCACGATACCCAGGCGGGTTTCATCAG
ATAGTATTTTGAAAAGTTGTAGGGATGCAATTTCTGGCATTATGTACTCCTGCTATGAATGGGTATTGTCTCTCCACCAAAACAA
GAAGTCAAAATCATATGTGTTTTCTCGCATGTGAGTGCAGGTGTCACTATGCGGTTGAAAAACGACCTGATGTGCGATCGTTA
CGTTAGTTGGGTAGGTAAAAAAGTCATTTGAGGCTAAAACATTGCTTAGTGCGGGCTTTCAGTTCATAGAGAAGAATGAATAA
TATCAATTATAACAATTGGTTATTTCCTTAGCGTACGTTTTCGTTCCATTGGCCCTCAGACCCCAGCTACAAACACGCATGATTAC
TGGCAGCTCTCACGCCTTGCCCGCTGGCATGTTGAAAGCGAAGCCATGAACCAGGCATTGGCAATTGTGGTGGCCGCGCAG
GGTAAACTGCCGATGTCACGCGTCTGGGGGATGGGCACGTCAGCATCGAGCGATGGTCAGTTTTTCCCGACAGCGCGGCAT
GGCGAAGCCATGAACATGGTCAATGCCAAATATGGTTCTGTTCCCGGCCTCAAAGCGTATACTCACGTAAGCGACCAGTTCGC
GCCATTCGCTTGTCAGTCGATCCCGGCGACCGTGAGCGAGGCACCGTATATTCTCGATGGACTACTGATGAACGAGGTCGGTC
GCCATGTTCGCGAACAGTATGCCGATACAGCAGGATTCACCGACCATTTGTTCGGAGCCAGTAGCCTGCTCGGCTACAATCTC
GTTCTGCGAATCAGGGATCTGCCATCGAAGCGGTTGTACGTATTTAATCCCGATACGACCCCCAGGGAGTTACGCAAGTTGGT
AGGTGGAAAAGCCCGGGAGGATCTTATCGTTGCGAACTGGCCTGATATTTTCCGTTGTGCCGCGACGATGACCGCTGGCAAA
ATCAGGCCCAGCCAACTCCTGCGCAAGCTCGCTTCTTACCCACGACAAAACAACCTTGCAGTTGCGCTTCGTGAAGTTGGTC
GTATTGAACGGACCCTTTTCATTATTGAGTGGATCCTGGATACGGACATGCAGCGGCGTGCTCAGATCGGTCTTAACAAGGGA
GAGGCCCACCATGCGCTCAAAAATGCGCTCCGTATCGGGAGGCAGGGGGAAATTCGCGATCGCACGACAGAGGGGCAGCA
CTACCGAATCGCTGGGCTCAATTTATTGACTGCGGTGATCATTTACTGGAATACCGTCCATCTTGGTCATGCCGTCACGGAGCG
GCGGAACGAAGGGTTGGATGTTCCCCCTGAATTTCTTCCCCACATATCCCCATTGGGCTGGGCGCACATTCTACTGACTGGCG
AATATCTTTGGCCCAAGGAACCGAAAGCTTAGGGTGTCATTTCGCCCTCAGCCGGAACCGACCCCTTCCAGGCGCGCCTTTG
CCGCCTGGATCGCCTCCAGGCGCTTCTCGCGGCGAGAAATCTCGGCAGGAATGTCCAGCTCCGGCTCGTTACGCTCCTGGTC
GTCGGTAGCCTTGGCGCGATCAAGCAGCGCCTTGATCTCGCAATGCAATTCGTCCTCGGCCGGCTTCATGCGCTTATAGCTCAT
CGCCTTGTGGCGGCTGGCGTTGGCTTTTACCTTGGTGCCGTCGACCGCGATCGTGCCAAGCTTCACCAGCCCACATTCGCGC
GCCAGTTGCACCACCTGAACGAACAAATTCTCAAGCTCGGTCAGGTGTAGGGCACGGAAGTCACTCAGCGTGCGGTGGGCC
GGGAAGTTTCCAGCGGCCAGCACACGCAACGCGACATCCTCGTACAGCTTCCTGGCTAGTTTGCGCGAAGAGAAGACGCCG
CTCGCGTAACCATAGATCAGCACTTTGACCATCATCGCCGGATGAAACGGCTGATTGCCCGGACCACCGCCGGCATACCGGGC
GTGGAATGCGCTCAAGTCCAGCGTATCGACAGTCTCGCTGATGAAGTAGGCAAGATGCCCTTCAGGTAGCCACTCTCCCAGA
GAAGGGGGCAGCAGATAGGATTGGTCGGGTCGGTAAGGAAGGTAACTGGCAGCCATCCCCGTATCGTCCTCGATCTTCCGCC
GATTTGGCTTCTGCCGCGCAGGCTCCTAGGCCACCGTAGCTAAACATACGAGAAAAACCGCTTCTTGAGCGCTCTCTGAGAC
CACCTTGCCGACAACGCAGAAACGGTTGCGGAAAAATCGGCGTTTCCGGCGTACGCCACCCGACCTGGACGAACTATTGCG
GACCTGGAACTGGAGTAGCGGAATTCCCCGGGGTTTCAGCGTCGCGCCACAGCGCCTCAGATAGATGCGGTAGCCTTTGGC

GGGTCGTCATGCCGCGGACCACCCCCCCAAAAAATTCCACAATGCGGAATGGCGGAATTTTCTGGGGTTCCCGCTTACCCCC
CCTTCAGGCGAGTAGCCAACCTGAACGGATGGTTCCACCGCAAAGGCGGGCAGGGCGGCTGTCGTAAATGCTGCGGCCAG
AAGCCAGGTTGCTAAGTTTTTCATTACAGGCTCCCGTTCACACACTGCATTTTATATTCAGGGTCAAAATAAATTTCTTTGATTT
TCCGCTTTTCCATATGCACCACAACGTCAATGGTGGAGTAGAGCATTCGCATAATATCGCTCATATCGAGCATACGGCCGATAG
GGGTCGCCTTGATAAGCAGCCCAATACGGTTAAAGGCATCGCGCGCAGAGTTAGCGTGCGTTGACATAACACCGCCTGGATG
GCCGGTATTAAGTGCTTTAAGATAATCCCACGCAGCATCATCCCTAAGCTCAGTCATGATGATACGGCCCGGTGTCAGACGCAT
ACAGGCTCGCAGGGCATCAGTGGCGCTGACGCGGCCGATCTTTCCTGCATCGCCGTACATCATATAAACGGCTTCTACAACGT
GATCGACCGTGACTTCGTGAACGTCCTCTAAAATAATTACACGCTCGTCTTTATGTAGCGATTTTAACAGCGCGCGCGTGAGTA
CCGTTTTCCCCGACCCGGTTTCACCGCAGATCACGATAGTGCGTTTCTTCTCAACGGCGGTTTGCAGGAATGCGGGCCATTTT
TCGCTGCTGTGCAGCTCTTTAAGGAAAAAATCATCATCCGTTAGGCTTTGCTTGCTGCCGGTAATCTTCCGGCAGTCACTGAA
AATCCCCTCGCGGGTCAGCTGCTCCAGATTTTTATCGGCCGCCAAATCCTTACGAAACGCTACGGCCGTTGTACCGTCAATCAC
CGCAGGGGGCAGACAGATAACGCCCCTGATCCCGCCAGGCAGGATCACGTCATTAATGGCCTGCATGGTCAGCTTGTTGCTG
CTCACCAACGATTTAGCAAGGTTCCTAATAAAATCTGCCGTAATTGCCGCGTTCTGCACAACCCTGCGGCCGCTGAACGTATCA
CAGATAACTTCCTGAAAGCAGTTAATGCGAATTTCAAAAACAGTAGGATCTTCTAAATACTCGCGCAGTGGGCCAAGTTGATA
GAAAGCTGCATCAGTCATGATTACTCCTGAAGAAAAGCGGGCGCTAAGCGCCCACTTTTTTAGTTGTCTGCGAGCGTATAAAC
GCCGCTGAAATCGAGGTCGCGGGCAACAAAAATGCTCACCGCATCACCCTGCTGATCGTAGAGGGTAGGGGGGATAGACAT
GTAAGAGCGGAGTGCTTCAGACGCCAGCTGCTCACCGCTGTTTTCTGTGCTGTTGTACTGAATGTTATTACTCTGCGTCTGGTT
AACCAGCGCCGTTAAGGTGTCAGAGAACAACGAAATCATGATCGCACCACGCAGACGCTCCCACATATGGGTATCCACCTGG
CCCGGAATCCCCGCGCTGCCGAGTGAGTTCGTTCCGGCACTGTCAATATTAACGATTGTCCCGTCCTGGTCATTGCGGATACG
CTCCCAGAGAACAAACACGCGCGCCTGGCCGTCTTTGATACCACCGGTAATCTGCCCGTCAACCCATGAGCCTTTATCAATCA
GCCTAACGAGTCCATCAGCTGAGTAAACGTCCTGTGAAACCCGGCAGGAAACCTGACCCGGAACAGTGGTATCCAGCTCGGT
GCCGGTACCACAGGGGATCATTTTGCCTTTCGGAACAGTCAGGCTGGGATTAGCCATGACTCCAGCGCGGCTAGCCTTCAGC
CTTGCAGGAGTCAGGTTTTTAGCGAGTGCTGAACTACCTTCGCTTGTTTCGTTGTCCTGGGGCTGCACTCCGGGACTATTGCT
TGTAGCCGCCTGATTAGTCTGGGCCAGCTCGCCGCCCAGACGACGCTGCATAGCCAGTTCTTCAGGCGAAGGTTCTTTACGCT
TATTAGAGGTACGCGCGGCGGTATTGCTGCTGCCCGCATCTGCATCTGCCTGTGCGGCAGCCTGCACAGCACGGGCATCAGT
GGCGCTATTCTGCGCAGTTGCAGGTTTATTAACATCAGGATCGCTGTTAAAGCTGTAGTTTGGCAGTGTATTGGCCTGTTGCGC
TTTACCACCGTCTTTATCAGCTTCAGCTTTAGCCGGGGTGCGAATTTTACCCATGACCGTAATCCCGATGAATACCAAAGCAAG
CAGCGCCATCAGTATGACAAAGGCTTTCATACCAGGAGCCGAACGGCGGTTACTGCCTTTAAATCCGCCACGCTCGCTTTCGA
ATTCACCGTCTCCGGTGTTTTCATCGAGTTCCTGATCTACATCGACACTTTTACGGGCCATCAGTTATCCTCCCCAATTTGAACC
CTGCGCACATCCGGGGAAGCCGTACCGGTTGCTACCGCACCGGCGCCCGGCGCGAAATTATTATTACGAACGCCAACGACTT
TATCGCCCAGACGAATACGCCACTCTTTAGCGACGGTTTCCACCTCGATGATGTTGCGGTTCTCACCCACAACATGAGAGTTAG
GCAGCGTTTCTTTGCCACTGGCCGAAATCATGTAGACCTGCGGTAACTCCGCATTGGCCGGAAACTCAAACCGGGTAAAGCG
GTAGTTATCCCAGACGTGAACCGGCTGGATGCTGCGCATTTCAGGCTGTTCGCTCATTACGTACTGATAGTTCTTCGCCCCCGC
AAAAGCCGTCTGCTTCAGCTTCTGCGTAATGCGTTTTTTATCAGCCGCGCTTTTGGCTTTTTCCTGCTGCTCAAACGGATATTC
ATAGGTCAGCTGAAGAACGGCCTGGCGCACAGCCCACGGCGTTTCAATAAAGGATTTTGATACCGTACCGTCTGCATTTTTCT
TCGTTTCTTCACCGATGAAATGGAGGACGATGTTATAGGTGCGCTTATCGGTGACGATCACCAGGTTGGTATCACTCATGGCCT
GTTTCGGCTTCACAAAAAAATGGTTCATTTTGTGCGCAAACGTCCAGCTTTCAGAATCGCCAAAAGCATGAGTGATATAGGTT
TCGTCAGGCGCGACAACAATGTGGGTAGCCACACCGGCGATAGCGTCAATTTTGACCACATTAACAGGGTTATAAACAACGC
TTTTAATGCGATAGTCATAAGGAGAATTGCGGCCAACCTCAAGCGCCATAACGTTAGTGGCCGCGCCTCCCAGGACTGACAA
AACGACTGCTGAAAGAAGTAGTTTTTTCATGGGGCAGCCCTCAGTTAACTTCAGGGTTGACGCGATAACTCGTCACGCGGAA
ACCCAGCGGGTTGACATAACGCTGCTCAGCATTCATCGCCAGCGATTTATATTCATACCCCATAATGGCAATCCAGCGCTGCGG
CTGATCATCAACGGGATTGCTGCGCACGCGGCGAACCGTAGTAAAGCGTATCGTTGCTACGCCGTGCGGTTTATCGAGGATCA
CAGAGTTAATCTTCACGCGGGTCGTTTCACTGTCGCCCAGAACCTTATCAAGACCGTTGCGGCCCTTGAACTTGCTCTGGTAA
GACTCTGCCACGTTCGGCGTGGACATTAAGCCAACGGCCGTATAGTCGACCTGAACTGAATAGAAGTCATAGCTCTCACGGTG
AATGACATATTGTGTCAGCCAGAACTTATCAATTTCGTCACCATAAGAGGTCTGGTCGCGGGTCAGCTTGACCTGCTGTACTTC
GTGAGTGGCCTCGTTGAGCGTTAGCAGATGTGCGGGGATTGGCTGGCTGTACTTATGCACCACGTAACCAACTAAAGAGAGT

GCAAAAACAGTTACCACCGCTGAACCGGTGGCAACCATCCAGGCGGTACGCCGGGACTTCAGCACTTCATCCATCAGATCAA
CTTCAAGCCCTTTACGGCTTTCGTTGAACTCTTTAATGGCTTCACGTGTAAGCCCTGTTTTTTTATTAGCTTTCATTTGCACCAC
CTTGCGTATCAACCGGGATTGTTTTGTTTACTGGAACGGTGTTGCTCCAGTCCGGCTCCGGTGGCGGTTTATGCCCGCTGGAA
CAGGCGCTAATCAGAAGAACTCCCATAAGCAATAAGCTGCGCATTACGGCATCCATTATCGGTTGTTGAACGTACTAATTATAAT
AAGTCGTCGAAATTGCAATTTCAACGACTTTTGTTTTAATTTTATGCAGCCTTCTTCCCGCGACTGCCCCTTGCCTTACCACTTA
AATTAGAACCACTGCTTCCTCCGCTGTTGCTGCCACTTCCTCCACCTGCCGATTGCTGGCCGCCGCCACTGTTACCGCCTCTGA
ACGCATTACCGCCAAACATGCCGTGGCTGCCCATATTGCCAAGCGCCTGCATGGAAGAACCCGTAGAGCGTGCGGCGTCAGC
AACTCCGGCGCTGATACCGCTACCCCAGCTGGCGGCAATTTGCGGAATCTGGAACAGAACGAAAACGGAAATGACCGTCAG
TAATAAGGCGGAGATAGAACCAGTGATTGATGAGTAAGCGGCATCAGAGTTCATCGAGGACAGGAGGTTGTCGAACATCTGC
ATGATGAAACCAAACACGAGCGCAAGAATGACGACGACAAGGCCATAGTTAATGACCGACGCCAGCCAGCGAGCAAAGATG
TTTTTTGTCGCTCCCCACAGCAGGCAGAAGATTGCAATCGGGCCAAAACAAAGCGTAACGGCCAGAAGGATCTTAGCCATGA
TCACAAAGCCCGCACCGAGGCCGCCCAGCACAACGGTAGCAATCATCATAATGCCGCCAATGGCATACGCGGCCAGGCCGCT
CGATGAAAACACGTCTGCGGCTTCCCATGCGGTGTTGACGATCTTGATACCTTTTTCAATACCGCTATCAATAATCGCCGGTAC
GCCACTTGCACCGACTTTATTAGGGGCAGACAGTATCCCGGCAAAATCATCCGGCAGGTGAAGCGCCACGTTGACCAGTTCC
TGTTGATACCAGCCGCCCGCCGTTGCAAAGCTCAGGATAAGGGCTATGGAAAGATACTCTTTAATCAGCGAACTCAGGCTGTC
GCCCGCCCCCGGATTAAACGCTGAGTACATCCCCTGAACCATCAGCTTGATTGTCAGACAGGTGGCAATCAGAGGCGTTACAT
CAGAGATAATGGTGGCAACATTGGCGCTCACCATTGACGTAATCGCCCCGTCTACTTTTGCGAAAATGTCTGCGACTAGGGTG
AATGCCATATTGCCTCCTTACTCCTAAATTTTCGGTGAATTGATTTCGCCGTCTTTTTTGCCGAACACAAACAGCTGACGATCGG
CTTCGCGCGCGTTTTTGCAATCAGGTTTCTGAAGTTCATCCGCGTCTTTCTTGCATTCCTGAATTGTTGCCTTGCGCTCTTTCTC
ATGTTTTTTGTACCACTCCACGTCATGCGAGGCATCGCAGGCCACTAGGAGGAAAGGGATAACAAGCAGTAGTTTTTTCATAA
TTACCTCAGTTAATTGAAGGTGACGCGGTAACGTCCCCGCCGGTTCCAAAAACAAAATTGTGGGTGGCACGTTCTTTCTGCGC
ACGTAATAGCTTGTCCTGTGACTGCTGCAACATGTTCATCAGATTCAGCTTCGCCTGCTCACCCTGAATAGCACCCTGTGACGT
CTGGATACGGGCCTGTAAGTCAGCAATCGATTTCAGGTCTGGAGTCGTTTTAATCTGCTCCGTCAGCTCCTGCATATCGGTTAG
TTCCTGCATCTGGTTGTTGTAGGCTTTTTCTGCCATAACACGGTCATAAGCGCCTTTTTCAGCCAGCTTTTTGTTCATGTAGGTA
ATTGCTTCGCTGGGCGTCATGTCGTCAACTTCCGCATTAAACTGGCCCATCATGCTGTTAACTGAAGGCGTGACGGAAGAACT
GGAGTTCATGGCGTCGCTGTAGATCTCCTTCCAGTTGTCCGGCAAATTATTTGCCAGCGTGCTGGTAGACGTCCCCAGCAGAT
CGCCCAGGTTGGTTGTCTTTGCCATCGACTCATACATATTTTTCTGCGTTTGCAGCTGGCTTTTAAGCTGCTCCAGCTGCTGCG
CCATTTGCTGAAGCTGCTCGACCTGTTTAGCCAGCTCAGTAGGGTTGGTCACGATGATGCCTGCGGAAGCGCTTTGCGCGCC
TCCCAGTATCAGGCCGGTGGTCAGCAATACTGCCGTCAGTGTTTTTTTCATGGTGTTTTGCCTCGTTGTTAAGCCGTCAGGCG
CCAGTATTCTTTGAGCCATACTTCAGGGTCATTACCGAGCCGTTCAACCAGCTCATGCGCAATTTCGGCGTTTTGTGGTTCACC
GGACAACACGCTAAGAACGTTGTCCATTGTCTTAATATCTGCATCAATATCGTCGCTGTTGCGAGGGTAGAGATTGAAAGAAG
CAATCGCAGACTGTTGCCCTTGTTTAACCAGGAACTGTCTGGAGTGTTCGGTAATCGACATCAGCGCGTCGTATTCAGCATCA
GTAAGGAAGGCATAATCCTCTCGGATAGCTTCCGGATCGCGCAGGCAGATTTTTGTGACGGTCTGCGACATGATCGTGCGGC
CAATACGGCTGGACAGCGCATCGTTCGGCTCCTGCGTGGCAAACACATAGATAGCGTCTTTCTTACGGTCAGTTTTGATACCA
CGCTTAACTTCACGCTCGATAACCGGATCGTCAAGGTAGGCGTGGAACTCGTCAAAGCACTGAATGACGCGGCGTTTGCCGT
CGATGGAGTCACGTACCCGGTACAGAAGGTACATCATGAGCGGAGTACGGGCCGGGCTGGATACTTCCTCTTTGGCTGCGAT
AAACTCGGATAAATCGAAGCCAAAAATATCATTCGCGCTGAGATCCAGGCTGTCTTTATCATTGTCAAACAGCCAGCCATACTG
GCCTTCGCGCGTCCATTCGCGCAGCAGCCCTTTCAGTGATACGCCATTTTCCACAACTTCATTCACGTACCCGTCCAGGATAGT
AACGGTGCGCGCCTCGCGTGGAATAAGTGAGCCTTCCCCCATAACCGCATCAACGCCTTCAGCCAGTTCGGTAGCCATCGTTG
CGCTGATAGGCCCGTTATTGGTGGTTTCCACACAAATGCGGAACAGGTTTTTAATGAGGGCAATATTGCGTTTGGTCGGTTCA
ATCTGAAGCGGGGCAAACCCGGACGGCATACCCTGTTGCAGAACTTTATAGTAGCCACCAACGCTTCGAATGAACGGCTCCA
TACCGCGGTCACGGTCATAAACAAAAAGCCGCGGGTTGTACTTCATTGACTGCGCCAGCAGGAAGTTAAGCAGCGTGGTTTT
ACCTTCCCCCGACATACCCGTTATTAACGCATGGCCCAGCGGGCGTTTACCGTAGGAAAGTTCTTCAAGCGGGGTCACATGGA
AATTAAAATAGAGTGGCGTACCGCTGATCGTGCGGAACATGGTCAGCGCTGGCCCCCACGGGTTATTGTCAGGCTTGCCACG
CATAAAATTGTGGAACGGGCTGAAGTGCAGGAAGTTCCATGAGTTTATCGGAACCGGGCGCGGCGCCCATTTCTGGTTGCCA
GGCAGTCTCGCATAATATGCAGCCTCAGAGGCCAGGCTGAGAGTCCCGCCAACCACGCCACAGCCGGTTAGCATAACCTTCA

CACGACGCGCTTTGCGCTGTACCGCGTTTTGGTCATTATCCCAGACATGCACGGTTCCATGATGGTAGCCCATCACGAACTCTC
TGGACGTCAGCATATCGAGCGCGGTACCAAGCTGTGCCAGCTGGCTTTGCGCACGGTCGCGCGTTTCCTGCAAAGATTTTTC
CTGATGCGTCAGAAACGTTTTAGCTGAAGATTCAGAGAGGCAAGAAAAACTCTGCGTCAGAAGGTATTCAAAATCGGCTTCT
TTAAGCATGTTAAGCTGGCCTGGCTCTGTATCTTCTTCGTATTCACGAAATTCAATGCCGGTGGTATAGAAGTTGTGATCTACCG
TTCTGATCTGGACAACATCCCCCCACAGTGAGCTAACAGGGCGGTTGTCCATGATGTACTCACGGATACGATCACGGCAAACA
GGCACGATGGCCCATTCCATATTTGCGAGGAAATAAAGGAACTCCAGCGCTTTTGAATAAGCGTGAGCCTGCGAAGGTTCAG
GCTCGTTGCGTTCGATAACAATGGCTTCGTCAAAAATGTCTGATTCATCGACTTCAGCAAGTTCTTCACGTTCTTTTTTATCAGG
CGCAGGAATTTCAACACCGCGTTTGTCACGATAATAGATACCCAACTGCTGAATGCCATACGGCTTCATTGCTTCCAGGATTTG
TTCAGAAATATCTTCCAGACCTTCAAGCGCCTCATTCTGCATTCGCTGAATTTCGTCACGAGTCGGCTTTTCAAATTTCGCCAG
AAACTTCTGTGTTTTATCCCCTACCTGTTTGTAAATAACGGTCAGATAAAGGTCATTAATCAGCTGCTTGGAATCACCGTGCAGC
TTACGGTTATATTGATCAACATAAGCAGGGAAAAAATGGTCATACTCACCATCCGGGTACTCTTTAGCCTCATGGTGATATTCAT
GCGTCCACAGCTCTACATGGTCTGTTCCGAAGCTCTTGACCAGCGTATTAAGGTCTTTATGCCAGGTGACCAGTTCCCGATCTG
ATGCGCAGTCATGTGTGCGGCCATCCAGCTTGAAAAAAGCCATCAGATCGCCGTTTTCCATCGAAATCACGTAATCATTGAGG
TGATAAGAATAGGGCAAATACTTTTTATTTACTGATGGCTCCTTACGGTAGGCATCAATTTTTTTTGGCTTCGTAGCGGTGGCA
GCTCTCATTATTTTAAACCCTTACGTTTGTAGTCAACAGAGGAATAAGACGATCCTCCCCACTGCTTAAACGGAGAATCAAACC
AATTACTGAATTTGGTTTTCAACCACAGGCCCATAATTCGAAACATGCGGTCGTCATATTTTGTTAATGCAGCTGAAGGAATCC
ACAAAAAGACGAACACCAGGATCGCCCACATATGAATAATCATAAAAAGCGAGGCAGAAATCATGAATATCATCATAGCTACAT
TACGTGGTACACCCAGCGCGCGAGGTAAGCGAGTCGCACCTTTGAAAAGCGGTCTTTTCCCGTCAACGAACATCTTTGTAGT
CCTCACTTTGGGGGACAAAAGTCCCCCCTTTTCGCGTTGTTCAGCTTCCTACGCCCGTCAGGGAAACCAGAAATGATGCAGA
ACCAATACCAATCAGCGAGATTACGATACGAGGAATAAAGCTGGCTGGGATTACGTGAAGCATCCACATAAAGCAACTAACCA
TGATCGCAATAGCACAACCAATTGGAATCCATGTGCTCAACCACGTCTGGATTGATGTAGCGGTTGATTCACCAGTATCGGTGC
CAGCGGCCAGCGCAATTTGCGGCAGGGCAATGGACAAAACGCCCATAACTACCGCAGGGCCATACTTCTTAAACAACGTGGT
CATACTTTCTCCTTACTCACTATCTTCTGTTGATTGCAGATTTAACCGGCATTCTTCAACGCCACACTTATCTTTCAAAAACTCTG
CCAGTAGCCTTAAATCAGCCCAGGTTCGTAGTTCATGCTCTTTGTTAACCCGAATGGAACATGCGATCTGGCCCAACTGTTCAT
CGTCTATCTGAAAAACGGGATACCACGTCTTTAATGAAACCCGTTTTATCGTTACCTCCTTCACTTTTCCTGTTTCAAATAGCTCC
AGGATCTGTGATTGCAATACAAGGTACAAACGTTCCATCCATAACCTCACTCCCCCTTCGCTGTTTCCTGTTTGGTTAAAAAGG
CATCCGTATTATTTCGGCTGAAGGCATCGCCATCACCCGAACCAAAAACATCTTGTTCACCGTCATACTGCGTGGCCGTACTTTT
TGCCTGCTGAGGCTCAGTCGCGGTGCTGTCCTCACTGGTCTGGCCATCAGGTAGCAGCGTAGGGATTTTCAAATCAGTTGATT
GACGCGCCACGTTGATAACTTTCGTGACATACCCGTTAGAAATCCCGTTTATGAGTGAGCCGGTGTTGTAGCAGGAAAGCGC
GTGTCTCAGCGCAACCTGCCCGGCTGGATAGGATTTCAGGGCGCTATCATAACAGGCTTTAAGGATGGTCTGGCTCGCCCGC
AGGTTGATGCAGGGCTTGAAAATATCGTCAACCGAAAGACCCAGGCCCACTAAATTATTTGAGTTAATTTGTGCAAGGCCCAT
ATCAAAACTTTTATTATCCTTCAGCAGAACTTTCGAAACGCTGACGGCCTCAGCTTCAGTACGTGGTTGCTGTTTTAACTGGGT
ACTACCACCGTTAATATTGATCCTGTACGGCCCATTTGAGGACTCATGGCCGACGATGTACGCCATTGTTAAGGGTGAAACATC
AGGCGCACATCTCTGCGCAAGCCTGGCAACTTCATCTGAGGCAGGCGCAGCACTGGCACGGCCAGCACAAGCAAGGCAGG
CCAGAGCGAGAACCAGAAGTTTTGGATGTTTACTCATTTACGTTTCACCGCGTAATTGTAATTTCGACGACTTATGTTTATGATA
AGGGCTAATGAGATCGTGTCAAGAAGAAACGAAAAGAAAACCTCTGAAGGAAGGAAGGTAATGCTGGTTCCCCTGAAATCA
GAAAAACGCCCCAAAGGTGAACCCATGTATCGTGACCCGGATAACCCTTTTAATACGTGGACTGGTATAGGGAAGCGCCCGG
CCTGGCTAACTGCAAAATTGGACGCTGGCATTAGCCTGGAAGCCATGAAAATGCAGGGCGTTGCCAACCCCAGAGAACATAG
ACAAGTAAAATACCGCGACCCCAGGAACGCAGAAAATACCTGGTCCGGGACTGGCCGCCGACCAACATGGCTCAAAGAGCT
GCTTGATAGTGGTTTATCACTTGATGATCTAAAGATATAACCGGAGAGTATAAAAATGGGCGATGTTATTGATTTTGCGGAAAA
GCAAAAAGGCAGAAGGAAGAAAAAGGCATTTTCCATTCCGCCCATTTTAAGAAAATTCCGAGTCCATGCTATCAGGTTACTC
GCCAGCATCATCAAATCCGGCTCTTATTCAGTTGCTTATATCGTTAAGAAAATTACAGGAAAGTTAATTAAGTTGTTCGCAATGT
TAACGATTTTCGTTTTTATTGTCGAATATATCGCAGGCGATATAGGTTACAAATCTATTTATAACTCTGCGTTATTATTAATATTACT
TACCGTTATCAACATTCTGGCGGGTGTATATCTGAACAAACTGTTAAGGACAAAACAATGAAGAAACTCTTAATCCCTCTGATA
GCAGCTGGTAGTCTGCTTTATCTTCCTGCCAGCCATGCTGAAGATCCCTGCAAAGTTATTATGTGCATGGCGGGCAAGCTCACC
GGCGATAGCGGCGGAAGCGAGTGTAACAGTGCTGAAGCTGCTTTCTTCAATATCGTTAAAAAGAACAAGCACGGCTTTTTAC

CCAACCACACGAGGGATGCCAGGAAGGCTTTTCTTAATGAATGCCCGGATAATGGCGAAGGTGGAAGTAACCAGTCGATGAT
AAGCCAGATCATAAGTAAATACGGGAAAGTTCGCTTATAGGCGGGCTAGGAATAATCTCAATTTAAGGAGCCAACGTGAAAA
AAATTATATTAAGTGCATTAGCCTGTACAGCTCTCTTAACTGGTTGCGTCAGCCAAGATAAAGGTAATGCGATGCAGAGCCAGA
TGAATAACCAGCAACGCCAGATTAACGAATTATCCGTTCGTTTGCAGTCTGCGGAGTCCCGGCTATCAAAGCAGGAAGAAAA
GCTGCGCAACGAACTGCTGCAATCCAGCGGCTATTGCTATCTGAATGGCGCCCGCTACTCGACCGGCACCGTACTTTACGGGC
GGATTTGCCAAAATCAGTCAGGCAGCGCTTCGTGGCAGGTTTACAGCCGTCGCTAAACACCAGGGCGGTTTAACCGCCCTTT
TCTTTACCCACAGAAAGCACACTATCACCATGTTCCCCTTCCCCACTACAGAAAACCTTATTCTATGGGCTTGCAGCGCCATCGC
ACTGTTTGCCGTTGTATTCTTCCGGCGTTCAGTACGCAACCGACGACACAAAAGGAAGCAGCAAAGTGCGCGGCGGGTGCT
GGAGCGCATAAAGACGTTGCCGGGCTTCCCACAAAAAATTAACTACCTGAGGAAAATTGATCCTTTTGTGTTTGAAGAACTG
TTGCTGGAAGGATTTGAAGCGCATGGCGGCTTTGTTGAATAAATCAGATTTCGGGTAAGTCTCCCCCGTAGCGGGTTGTGTTT
TCAGGCAATACGCACGCTTTCAGGCATACCTGCTTTCGTCATTTTGTTCAGCGCTCGTACCAGGGCCATAGCCTCTGCAACCTG
ACCATCGTAGTCACGCAGTGTCAGTGAACCTCCGAACAGCTGTTTTACCCGGTACATCGCCGTTTCCGCTATCGAGCGACGGT
TATAATCTGTTGTCCATTTCCACCGCGCATTACTCCCGGTCAGCCGCTGATTCGCAACAGCACGGTTACGGTCTGCATATTCACC
GGGCCAGTAACCCGCGCCTTTTCGGGGCGGGATAAGCGCGCTGATTTTCTTACGCCGCAGTTCATCGTGACAGAGCCGGGT
GTCGTAAGCGCCGTCTGCCGATGCTGCCCTGATTTTTCTGTGAGTCTGCCGGATAAGACCCGGGAAGGCTTCTGAGTCCGTCA
CATTGTTCAGCGACAGGTCTGCACAGATGATTTCATGTGTGTTGCTGTCAACGGCCAGATGCAACTTTCGCCATATACGACGG
CGTTCTTTGCCGTGTTTTTTGACTTTCCATTCGCCTTCACCAAAGACCTTCAGCCCGGTGGAATCAATCACCAGATGCGCGATT
TCACCCCGGGTGAACGTTTTGAAACTGACATTAACCGACTTTGCGCGCTTGCTGACACTGGTGTAATCCGGGCAGCGCAACG
GAACATTCATCAGTGTAAAAATGGAATCAATAAAACCCTGTGCAGCCCGCAGGGTCAGCCTGAACACGCGTTTAATGACCAG
AACGGTGGTGATGGCGAGATCAGAATAGCGCTGAGGTCTTCCCCGTGATGAAGGCGTTGCCGACTCATACCAGGCCTGAATA
GCTTCATCATCCAGCCAGAAAGTTATGGAGCCACGGTTGATGAGGGCTTTATTGTAGGTGGGCCAGTTGGTGATTTTGAACTT
TTGCTTTGCCACGGAACGGTCTGCGTTGTCGGGAAGATGCGTGATCTGATCCTTCAACTCAGCAAAAGTTCGATTTATTCAAC
AAAGCCGCGCATGGCTTCAGAACCATCAGAAACAAACGCTATACCGGCGATGGAGGCATTGACGGCCAGGTAATAATAGGAA
AATATCGCTATCTTATTCAGGCTAAACGCTATCGCGGCCATATTGCTTTACAGCACGTACAGGAGTTCGAGAAGTTGCTTAAAC
GTCATAACTGTCGCGGTCTGTTTTGCCATACCGGGAAAACCGGCGCAGGTTCAAAATCTGTCAGTATTGCCAGTGAACGGATG
GAGATTATCAGCGGCCAGCGCCTGATAGATTTGCTCACGCCCGGCAGCTCCTTCACTATCGCAACCGCCCCGCAGACGATGAT
GAAGCGTACCGCAGCAACACTAGAAACGAGCACCATTGTTAAAGATGCCGGTAAAGAAAATCGATACCATGAGAGTTAATTA
AATGAAGTCAGTAACTATAGAAGCAAAAACATTTGCTGAAATGTTAGGAATAACAGAAGGTGAATTAATCTTTGCCATTAAGA
AAACTGGCACATTCAAAAACAAGACCATCCCACAACCTCATGAGCCACATAAATCAAATAATAGATTTTTATATTCAGACGTAAT
GAGGTTTATAGAATCACTAAAAGACAAAGAGAACCGGTAATGACTCCAACTTACTGATAGTGTTTTATGTTCAGATAATGCCCG
ATGACCTTGTCATGCAGCTCCACCGATTTTGAGAACGACAGTGACTTCCGTCCCAGCCTTGCCAGATGTTGTCTCAGATTCAG
GTTATGTCGCTCAATGCGCTGAGTGTAACGCTTGCTGATAACGTGCAGCTTTCCCTTCAGGCGGGATTCATACAGCGGCCAGC
CATCCGTCATCCATACCACGACCTCAAAGGCCGACAGCAGGCCCAGAAGACGCTCCAGCGTGGCCAACGTGCGTTACCTAAC
AATAAACCTGTTTAAATATCCAGATAAAAACATTCAATCTGGGTCAAATGAGTGATACAGTTTCACCCATAAGACCCAATGGAG
GCAATATGTCTGAATTTGAATTACTGGCGCAGGATCTGCTTGAGAAAGCAGAAGCGGAAGAACAACTGCGACAGGAAAATTA
TAAAAAGCTGCTCGGGCAGGTGCTGGAAATCTATGACCAGAAGTACGTGGCTGAACTGCTTAGAAAAGTTGGTAAAAATGA
GTGGAGTCGCGAGACTCTTAATCGCTGGATTAATGGTAAGTGCTCACCTAAGACGCTGACGTTAGCCGAAGAGGAACTTCTA
CGAAAAATGCTTCCGGAAGCGCCTGCACATCACCCTGACTATGCCTTCCGGTTTATTGACCTGTTTGCTGGGATTGGAGGTAT
ACGGAAGGGCTTCGAAACCATCGGTGGCCAGTGCGTTTTTACCAGTGAATGGAATAAAGAGGCTGTGCGCACATATAAAGCT
AACTGGTTTAACGATGCTCAGGAACACACTTTCAATCTCGATATTCGGGAAGTCACGCTCAGTGATAAACCTGAAGTACCTGA
AAACGATGCCTATGCTTACATTAATGAGCATGTGCCGGATCATGATGTACTTCTAGCAGGTTTCCCCTGTCAACCGTTCAGCCTT
GCGGGCGTAAGCAAGAAAAACTCGCTCGGGCGCGCGCATGGTTTCGAATGTGAGGCTCAGGGAACGCTTTTCTTCGATGTG
GCGCGTATTATACGCGCAAAAAAACCTGCCATCTTTGTTCTTGAAAACGTTAAAAACCTGAAGAGCCATGACAAGGGTAAAAC
CTTTAAAGTCATCATGGATACCCTCGACGAACTGGGCTATGAAGTTGCGGATGCAGCTGAGATGGGCAAAAACGATCCTAAA
GTTATCGACGGAAAGCACTTTTTACCTCAGCACCGAGAACGTATCGTTTTGGTCGGTTTCCGTCGTGATCTGAACATTCACCA
GGGCTTTACCCTGCGCGATATTAGTCGTTTTTATCCGGAACAGCGTCCGTCATTTGGCGAACTGCTGGAACCCGTGGTTGACA

GCAAATATATACTGACGCCGAAACTCTGGGAGTATCTCTATAACTACGCCAAAAAGCACGCAGCTAAGGGTAACGGATTCGGT
TTTGGCCTCGTTAATCCTGAAAATAAAGAAAGCATTGCCCGTACGCTTTCTGCTCGCTATCACAAAGACGGGTCTGAAATTCTG
ATAGACCGTGGCTGGGATATGGCCACAGGTGAAACAGACTTCGCGAACGAAGAAAATCAGGCGCATCGGCCCCGCAGGCTG
ACTCCACGAGAGTGCGCGCGCCTTATGGGTTTTGAAAAAGTAGATGGCAGGCCTTTTCGCATTCCTGTGTCAGACACTCAGT
CGTACAGGCAGTTCGGTAACTCCGTAGTGGTGCCCGTGTTTGAAGCCGTAGCCAAACTGCTTGAACCTTATATCCTGAAAGCG
GTTAATGCCGATTCGTGCAAGGTTGAACGAATCTGATCGCTCCTCCCGGTATTTATGCCGGGAGATAATCTATGGAATATCTGC
GTAAAGCCCTGTCAGCTCAGCAATAAACGCACCTAGCGTCATTAGCTCAGCTCTCACCGCCTCCGGGTATTTTTTGTGCAGCG
ATGATGGCACGACCAATCTGACACCCGACTCCCGCATCTCCCGATATTGAGCCAGAGAAACTCCCTCTTGGAGTGTAAACAGA
TGCACCTGATGAATTTTATCGGCCTCATTCAGTATCTGACGCCAGCGATCCTTACAGGTAGTCTTGACTGCCAGCATGCGCAGA
TTTTCTACGGGAAACTCAGTATCGTGGTAAGCCCCTGCGGAAGGGAAAAGGAAATCGGGTTTTTTATTACCTTCTGTGATGGC
CTGCGTCGCAAAGTGTCGCAGGCCGTGCTCAATGAATAGATGCTCCAGGTGCAGTTCCAGAGACTTCCCGGCTCTGGATTTAC
GGCGATTGCTGACAGAATTGGCCAGCGCAATAAATTCATCCACAGAGCCAAATCCTTTCCGGATGATATCCAGAACATGCAGT
TCCTCAACCAATAGAAATATGTCGTACTCCACGCGCCGGCGGTCAAGAAGTTGCTCATCCGGATCAAGGGAATTTTTCACATA
ATGGCTGGCTGCATACTGAATAATTTCACTTCCCGACGGAAAGCGCAGGTGCCAGTCTTCAGGTAGAATATATTTATGATTTAC
TGGCGCTTGCTGTAGAGATAGTCCGCCTAGAATCTGTCCTGCGGGGCCGGATATAAGCGCTCCGGGTATAACTTCACCAATAG
CGGTCTCAATGACGTCCTCTTCATCAGTGCTGGCGCATACCCAAATATTTACTTCCTTACAGTCCCCCCTTGCTCATCAAGCTT
GAAAGCCAGGAGCGTCAGAGCCCCTGTATTTTCAGGATTCTGAAGTGGGCTGCCTCTACCCCAGCGGGTAATCCTTTTTTCAT
TCCGGGTTTTACCAAAATGACGGCTGTTATAATAAATTGCCCGGGCTTCGCTGTCAGGGCAATCATGCGATGACACATGTGCG
GTGAGAAAAACCGAAGGGTTCAGTTCACGGGTATGGTTGATAGACGGAAAGAGTTTTTCAACGATACCTGAAGGGATATAA
AGCCCTACCTGGTGACCACCTGTTGCGCCGGTATCGTTGGCGGAAAGGCGTTTGATGTAGACGAAGTAATTCTCACATGCGAT
CTCAAGTAGCCAGTTGTGGAAAACCGACATAAGCATCCCCTGTTACCCTGAAACTCTACTCACCATTTTTTCATGATTATATACA
AACAGCCAGAAAGGCTGTTACAGACGATTTGATCTGCAACCTATTGGTTAAATTAATGTATCAAAAACGATGGTTTTTGTGACA
GTCTTGAAAAGTCCTGACTTCTCCCGAAAAATGACTCCCCTCATGTAACAAAACTCGTTACTGTATCAACATAACAATAACCCCA
TAACTAATTAGCGAGAAAAGAATGAAAATCGGCTATGCACGTAAATCGGCACTGTTGCAAAGTTAGCGATGAGGCAGCCTTTT
GTCTTATTCAAAGGCCTTACATTTCAAAAACTCTGCTTACCAGGCGCATTTCGCCCAGGGGATCACCATAATAAAATGCTGAGG
CCTGGCCTTTGCGTAGTGCACGCATCACCTCAATACCTTTGATGGTGGCGTAAGCCGTCTTCATGGATTTAAATCCCAGCGTGG
CGCCGATTATCCGTTTCAGTTTGCCATGATCGCATTCAATCACGTTGTTCCGGTACTTAATCTGTCGGTGTTCAACGTCAGACGG
GCACCGGCCTTCGCGTTTGAGCAGAGCAAGCGCGCGACCATAGGCGGGCGCTTTATCCGTGTTGATGAATCGCGGGATCTGC
CACTTCTTCACGTTGTTGAGGATTTTACCCAGAAACCGGTATGCAGCTTTGCTGTTACGACGGGAGGAGAGATAAAAATCGA
CAGTGCGGCCCCGGCTGTCGACGGCCCGGTACAGATACGCCCAGCGGCCATTGACCTTCACGTAGGTTTCATCCATGTGCCA
CGGGCAAAGATCGGAAGGGTTACGCCAGTACCAGCGCAGCCGTTTTTCCATTTCAGGCGCATAACGCTGAACCCAGCGGTA
AATCGTGGAGTGATCGACATTCACTCCGCGTTCAGCCAGCATCTCCTGCAGCTCACGGTAACTGATGCCGTATTTGCAGTACCA
GCGTACGGCCCACAGAATGATGTCACGCTGAAAATGCCGGCCTTTGAATGGGTTCATGTGCAGCTCCATCAGCAAAAGGGGA
TGATAAGTTTATCACCACCGACTATTTGCAACAGTGCCGGTCGCCGGGAGTCAGCAGATCGACGTCAACGCCGAGCAGCGAT
TTCAGTTCTTCTTCCAAATCGCCCAAGTCCAACAACGTGGCACCGGGCAGCGCATCGACCAACAGGTCGAGGTCGCTGCCAT
CCCGGTCGGTGCCATGCAGCACCGAGCCGAAGACGCGCGGGTTCGCGGCGCGAAAGCGGCCTACCGCTTCACGCACTGCG
CTTCGCTTCATGTCAAGCACAACAGACGGTCGCATGCGCATCCTTTCTTATCGAAACTCGTTGAGATGATATGCAATCAAGAAT
AGAATTTCAAGAACTCACAAAGTAACGCGGTGGTTAATATCCTGTACCCACGGATTGCCCTTAGCGCTGCCTATATCGGCTAAA
GCACTCCGGTAGCTTGATTCACCCCACGGCCACGGCAGGATCTTGGCCGTCGCAAGCGCCAGGGGAAAATCTTCAGCTGCA
AGCCTGAGTGATTTCATGTGCGTGTAATCCATCGCCCAGATGATTTTTGTGAAGAAGAACTCGCGCTCGTTCATGTCCGGGCG
CGCGTCCTGGCCCTCCGTGCCAACGGCCAGCAAGTAATCGGCCTGAATTGGCAGTATCAGCGCGCGTAGTAAGTCATGGATC
GCCGGTTGCGGCAGTCTGCGCGCCAGATTAATTACCCGGTCGAACTTCAGTTTATCCTGCTTGCGCTTGTCGGTCTGCTCCATC
AGATTTCATGGCCCCCTTCTTCATGCTCATGCTCATGGGTGTGTTCTTTTCCGGTATGGCTCTGTTCCGCCTGAGACGTCTGCG
GCATGGCGTAATCGTCGTAAATGCTGCTGTCAAAGTCGTAGTCTGATGCTTCGGCATAATGCTCGTAATCGGCATACTCCTGCG
CGCTCCACTGCTGATCGTCGGCAGCAGCATAATCATGGGCCAGCTCTGCATCATTTTGCTGTGCTTCATGACGCCGCAGGCCA
ACGGAATCATCCATAGGGTTCTGCTTAAGATGAAAGGCGTCCTCTGCGTTGCTCACCGGCTGATAATCAGTGCCGGTTGTCAT

GTTATGTTCATCGGGTTTCTGGTTAAACGCCATGCTTTCCCCCGTGGCTTCTGGCAGACCTTTTTCAGCTGATCGGGTTTCTAA
ACTGGTATCGCGGCCAATATCCTTAAACCTGGCCTCAAGCCCAAAGAAACGGTCAATTTCTGCGGCCGTGGTTTTCGGGCTGT
CGCGGCTCACGCTCGATGCCAAAGATTTTTTATCGTCGGTAAAAATTTCCACCTCATGACGCGCACGCGAAATACCAACATAAA
AAACGTCCTTAGAAGTGGTAAGCGATTTGGTATCTATGTTGAACAACACGCGATCACAGGTAAGCCCTTGGGATTTGTGGACG
GTGGTTGCATAAGCATAGGAAAGATAAGAAGCCTGTTTTTTGTCCAGCTCAACCGTGCGCCCTTTTTTGTCCTCAAGCGTCAG
TTTTTCACCCTCCACGGTTTTCACCGTGAAGCGGTCGCCGTTGGCAACGTCCAGCGTTTTATCGTTACGCGTTACCATAACCTT
ATCGCCCGGCGCCAGTTCGGCGCTGACTGCCTGGTATACAGACAGCTTGGTGTGTGTACGCGGGCTGAAAGCGATCTGCTCA
CCGCTGCTGCTTTCAACCGTCAATTTGTTGCCCGGCCCGGTATCAAGAACCTGGTAAGACTCGCCCCGCTTCATACCATTTTTG
TAATCCTGTTCGGGGATAATGATTTGCCCTTTACTGAAATAACGGCTGTCGCGGCGTTCCGCCTGTGTCGAATCCACGCGGTCA
AGTAGCGTGAACGTTTCGCCGGTTCCGGCAAGCCCCAGATTGCCCCGGATGTAGTCATTGAGGGTTTTGCGTGAGGCGTTCG
TACCAGAGATTATCAGGGTGGCATCCTGTTGTTCTGAGGACAGAGACAGGTAGCGATCGGCAAGTTGAGCGAGTCGGGGCG
CTTCTTCCTTCAGTTCGTTCACGCCGGTGATATTTTTCAGGGCGCGCGCGGCATTACCTTCAGCGGCATACTTAACCGCCTCAA
GCAAAACTTCATTCTTCTGTCGCTGAATGTCTTTCATGTAGCTGGTCTGCATATCTGCTTTAATCAGCTGCTCAAAAGGCTTACC
GGCTTCTACCGCTTTCGTCTGTGACGTATCCCCCAGGAATACCGCGCGAGCGTTATGCTTCTCGATCACCTCCATCAGCTGTTTC
ATCTGTCGGGCGGGTATAACCCCGGCTTCATCAATGAATACGACTGATTTTTCATCCAGCTTTTTATCCTTCGCTTTGAGGAAA
GCGGCAACGGTGCGGGCCGGTAATCCATCATCTTCAAGCGCTTTTTTCTGTGTCCCATAGGGGGCCAGCGCCGTGACCTTCA
GCCCTTGTGACTCCAGCAGCTCTTTAGCGGCCATCGTCATATAGCTTTTACCGGTACCGGCGTAACCATGTGCGGCCACAAACC
GATCTTTGCTCGTCACAATTTCTGTAACCGCGCGCATCTGCTCCTTCTTGAGGGTTTTCCCGGCAAGCAGCTGGCCTGCAATCT
CTGCGGTCAGCTGTCGCGGCATCTGCCCCCGGCCGCGTGATTCGATAGTCAGAATGGAACGCTCAAGGCGAATACCCTCCAC
GGTAGTGACGCGGTGGCTGGTCTTTTTAAGCCTGCCGTTTTTAATACCATCATCTACCGCAAAACGGGCTTTATCCGCACGCAT
CCCGCTATTCGTCAGCGAGTCGATCCACTCTTTGCGCGTCAGAGTTTCGGCCATAACTGAAGCACCGACCTTCAGAGTTGATT
GATACCGGGCTTCGCCCTCGATGATGGCGCCCTTCTGTACCGCCTTCAGGTACGCTTTTTCAACATCGGCTATTGTGGCATGGC
CCAGCACCTGCTTATTAGCGATTTGAATCAGCTTCTGGCGTTCAAAGCTGGCATCGCGCTCTGACAGCGACTTAACTGCAAAC
TGGATAGCCCGGTCAGCTTTAACCTCCGGGCTGGTAAAATCCGGGGCCATGTTGCGCGCTATATCAGCCTCCAGAGGTTTACC
GTGTCCCTGCCATTCACGGTTATCAAAATCAATGCCGAGCGTTTTGGCGCGGCTGGCCCATTCCTGGTGAATTTCTTCACGGG
AATGCTCTGTTTTCTTTTCACGCGTAGCCATCGAGACGCGGCTTTTCGTCTGAGCATCGGCGGTTTCCCGCGTCAGACCCATT
GCAGCGAGTCCCTTTTCAATTTGCTCCGACCGGCGGGAAAAAGCGCGAATCTGTTCATCTGAAAAATGGGCCATATCGAACG
TGTTATTTTTGCTGTTGTAACGCAGCTCATAACCGGCTTTGGTCAACTCCAACGCCAGCTCCTGTTTGTAAACATCGCCCAGGT
GCATTTTGTTACGCATCAGCTCATCATTTTTGAGCGCGCGCCACTGGCCGTCCTCGCGCTGGGTCATGTTCATGACAAAAGCG
TGTGTGTGCAAATCAGGATCTAGCGCCCTGGAAGTTTCGTGGCGGAAAGTAGCGACGACAAGGTTATTGGTATTCTGGGTTA
CTGATTTCCCCTGGCGAGTCGTCCGGGCCTGCGCGAGTTTTTCAGCTTCACGCACAGCAGCGGCAACAGCTTTTTCATGAGC
CTCGATAATGGTTTTATCGCCGTGTATCAGCGCCTGCATGGATACCCCTTTAGGCGCTGAAAACGTCAGGTCGTAGCCCAGAC
GCTCTTTTTTGGCATCACCCACGTGTCGCTGCATATGCGTGAAGGTATCTATCTCTCCGACAAGCAGCTCTTTAAACCGGGCTG
ATTCAACGTCCCCGGATAAGCCGAGGGCTTCAGCTCCGGTTCCCTGCCAGGACGTGAATGATGAATCCTTACTGTAGTAATCA
TCCTTTGCATCAGAGTAGTAGCCCACAACGCTAGTGACGTTCTGGCGGGTAATCGTGGTTATATCAAGCATCAGATCTCCCTCA
GTTCAATGCCAGGAACAGGGTTTTTGCGATGGTATTTAACGTGTTTAGCCTTGAACTTAGCGACGGGCATATCACCAGGCAAC
GCCAGATAGCCGGTGAGGTTTGGCAACATTGATATTTCGGTAGGCGTTACGGCACGAACAACTTTAACGTCGCGGCGTTTAC
GGACAATCCAGGGCTTCTGAGGATCGGATTCTTTACGCTCAACTTCGCCTTCTATCTCACCGAGTGAGCGCGACATTTGATCC
AACGTTTCATCACCGAGACGGCTGCCGCCCAGCACGATGTTAGAACGCATGTTAGCCAGAATTGTCTGAGCCATATCCCGACC
ATAAACCTTAACCAGCTGAGAATAGGTTTGATAGCCAGCATAAACACACAGACCGCTTTTACGCCCTTTGGTCAGTGCATCGTT
GAGGTTTGGCAGAAACTGGAGTGATTCCAGCTCGTCAATAAATACATTAATGCGGCTTTCTTTTTCACCCATACCCAGCACGAT
AGAAAAAAATCGAATCCAGCCAGCAGGAAATTAGCGGATTAAGTGACCTTTTCATTTCTTCCTGCCAGGTGATAAACAGGGTTC
CCGGCTTTCCATCATCAAGCCAGTCACGCAGGGAAAAATTACCTTCCGGCATTTTCAAATGTGGGGCAAGATTCTTACTGAGA
ACAAATCGCGCGCTTCCAACTGCTTTTTCAGACCCGGAAAAAATAGCTTCGGCAGGCGTCCCCATTAAAAATTCTTTTAATTTT
TTCTGGTCAACGTTACAGGCCCAGTGAATAACTTCTTCCATAGTTACTGTGCTGTATAGGCTGTGAAGTTTTTTCGAAACTTCA
CTAAAAATAAGACGGCCATAGCCGAACCATTCTTCAGTAGCCATATCAGGGCTTTCCTGAACAATAGAGTTCACTAAACGCTCG

TAATCATATGAACGGCGAATTTCATTGAAAAACACCCAGCCTTCAGTGCGTTTATCATAGGCGTTTAAAATAACATCGCCGGGA
CGATAGAAATTCTTTAAGAACCCCCCATTTGGATCTAAAGCAATATTTTTGCCGCCTCTAATGATGCTCTTAAATAACAGTTCATT
GAAAATTGTGGTTTTACCAGTACCGGTTGTACCGGCAATCGAAAAATGCAAGTTCTCAGCGTATGTAGGTATGGGGATATTAG
CCACGGTTAACTGGTTGACACCTCTTTCGCGTGTTTTATCAGCGAGTGTTCTGGCGCGAACAAGCTCTGTACCACGATAAATCT
TTTTGAATCTTTCGCCTTTAAACACGCGTGATTTATCATAAATGATAAAAGCGATCAGACCGCCAACACCAATAAACCAGCCAG
CAATTAAAGCTGACCATAAAGGCCATAGCGAAAAAGTATTCTTAACCAGATACGGAATCAGGTATTTAGCCGTGGATGGATCA
ATACCGTAGGTAAATTTTGCAACTAGAAACCATACCATCACTGGAGGCAAAGTAATTGCAAATAAAAATGCTAAGCCTCTTTCT
CTATCGTCCATTTCAGCGCTCCTTTTTTGGTTCCCAGACTTTGTAGCCGTTACGTTCAACCTCTGCTTTTGCCGCTTTGGTTTTG
CCCGGTTCTGCTATCGAGCGCAGGAGGATTAGCGTTTCAATAGCGAGTGATTCATGCAACATCATCTGTCCTGCCGGTGGGAA
TTTTACGCCAGATAGCGTTTCGGTTATTGCCTTCAGCTCATCGCGCAGTGGGCCAAAATCCGCATCTGAAGCACGGTCAAAAA
GATAATCCAGTTTGCGATTTACGTCGCTCAGCCGGTCGGCAACTATTTTCAACCCGGACTCCCGATCACCTGGGCCAGCTTCA
ATGCAGCGCCGCAGATAATCTGACCGATTACCTCCTGAAACCAGGTCTATATAGGCCAAAAGTTCATCTGATACTTTTGCGGTT
ATTATTGGCATTCAGTCCTCACATTGTGCATTTCTTAAACAAAAAATTGGGATCTAACAAGCTGAAATCTTAGTATTACCAAAGT
AATAAAGCAAACTCATTATAAAACAATGGGTTATTGGGTGTTTTTAATACCTAATTATTACCGAATATTGACGCTATTTATTTTTTT
ATCTTTTAAATCAGTACGATAGCGTGATTTATCGCGCTGCGTTAGGTGTATAGCAGGTTAAGGAAAAAAAATCATCTTTTTTGG
TAGGAGCGACCTCCGTAGGTTAAGGGTCATTTGGCTAAAAAGCGTCCTATTCTTTGATGGTCATGCTTGCATGACCATCTGAGC
AACCAAAAACTACAGATAAACTACAGAGAAACTACAGATAAACTACAAAAAACGATTTACCTTAGCGTTGTCAGACTACTAATA
GACTACAAGGAAACTACAAAGAAACTACAAAGAAACTACAAAGAAACTACAAATAGACTACTAAAACCGTGGCAGACTACTA
ATAGACTACAAGAAAACTACAAATAAACTACAAAACTGGATTGACCCCTTCTTACGAGTGTTGTAGAGTCATCTTCATACAACG
GAGGGGGTTATGAATAAACCAGCAGATCTGAAACCCCGCAACTTATCGGCTGCTGTCAGATTGCGCCTAAATGAAATCGAGA
ACTGGCTGGACAGAGGGCTAACGCGGCATGAAATTGCTGAAATCCTCGACAGCGAATACAGCTTTTCGGTAACAGCCAAAG
GGCTTGAGATGGCACTGTATAGAACGCGGCAAAACCGAAAAAATGTATTGCACAATACACATGATAAGAGTAGCGCGAAGGG
TGCAGCGGAAAGTGTATTGCACAATACACAACCGTCTGAGCCTGAAGCGCAGGAAAGTGAAAAAGCAGAGAGTCCCGGCAT
TATTGATAAAGAGTTCTTCAATAAAATCGGTGAGGATTTCAACCCTAAGAAGTTCAACAAAAAATTCTGAGGTGATTTATGAAA
GTAGCGGTAATTAATTACAGTGGCAGTGTTGGTAAAACATTAATTTCATCTTACCTGTTAGCCCCGCGCCTGACTGGTGCAAAG
TTCTATGCGGTAGAGACTATCAACCAGTCTGCTTCCGATCTGGGTATTGAAAATGTGACCAGTTTTAAAGGTGACGACTTCTCA
CGTTTGATTGAGGATATTGTTTTTGAAGATGCAGGCATTATTGATATTGGCGCGTCAAACGTTGAAGCGTTCCTGATGGCTATG
TCTCGCTTTGACAGTGGCGCGAACGAATTTGATAAATATGTAATCCCGGTGACGCCGGATAATAAGGCGATTGATGAAAGCCT
GAAAACGGCACACACGTTAAGTAAAGCGGGCGTGAGCAGCAAGAAAATTATCTTTGTTCCAAACCGTATTAGTCCAGACAGT
GAAGTAGAAGATGTGCTGGCGCCGGTGTTTGAGTTTGTCAAAGAAACGAAGATTGGCAAAATAAGCAAGAAGGCTGTTATT
TATAACAGTGAGGTTTTCGAATATCTGGCGTTTCACCGTATCTCATTCGAAGTATTGACCGCTGAAGATCCAGAAGAATTCAAA
TCCCGTGCAAAACAAACAACCGATGCTGACGAGCGCAAAAAACTGGCACGCCGTTATACATACATGAAACAGGCGATTCCGG
TAAAAGCTAATCTCGATAAAGCATATGCGGCTTTAATGGGAGAATAAAATGGAAAAGCAGCCGGATAAATTAGAAGTTCTGAT
GGACTGGTTTTTAGGTGACGCGAAGGAAATCACCGCAACTCAGAAAGAAATGACGCAGAAACTTTCTGAGCTTTCGGAAAA
GCTGGCAAAAGACACCGAAAGTTTAGGAGAGACGGCAGACTCTTTTAAACGGGCTTTAGTAGAAAACCAGCGTTCAATTAG
CCTGGCAATTAGTGATGATGCTAAGGCGCGCGAGGAATTTCTAACTAAATTCCGCCGCGCGCAGGCGTCCAGTGCTGAGACG
TTTACCCGTCAGATCCTTTTTATTACAGCTGGCTGCACCATCGTGGGCGCCGCAGTAGGCGCCGCGATAGCGATACTTTTACTG
AGATAAAGCAAACCGGGCGTGTCCCGGTTTTTTTGTCAAGCGGAGCGCGGAGGCCGAAGGCCGGAGGCATTAGTGGCCGC
CGCCCGCGTAAGCGGGGCGAGACGGGAACCGGCTCGAAGCGCAGCACGGCAGAACGGCCCCGCAGGGGCAATGCCCGTT
TTAATTCATCGTGACAGTCGCGCGTGACCATCACGGGGAGAAAAATAATGAATGACCGACAGCGAGAACTGGCCCGTATACG
CCAGGCCCGCCGCCGCGCGCGGCTCAAGGAAGAAGGCACAAGCGTGACAGTCACGCTAACAAAACAGGAAGAAGCAATGT
TGCAGGAGCTGTGCCGGGTTCGCCGTCCTGGACGAACGCCTTATTCAACGAACGAATTTTTCCAGCTGCTGCTTATCCGCAAC
TGGCAGCAGTGGCAGGAGCAGAAGGCACAGCTGGGAAAATGCCAGGCTTGCGGAAAGCTGAAAGCGGAGGGGGGGTGC
GAGGGTGAACGGAAAGGCGAAACCTTTAACTGCTGGCTTGCCGTCGAAGCCAATGAACTAAATTTGTAGTGTATTGTGCAAT
ACACATTTACACAGAAACAAAAACCACCGGCAATTCCTGGAACCGGATACCTACGGCTATTCCTGGGTGAACGGTACTTTTTG
CACCTGGGTGCGCTGAAAAAGCTGAATATGCAGGGTGACGTTCGCGTGCTGTTCTGCTTTGTAGACTGAATGCGCCAGCTATA

CGCCTGACTGCTTAAACCTGGTAAAGTTCTGCAACCGGCACTGACCGGAAAGCAAGGCAGGGAAGACCTAAGCCAGAAACC
TTGACTGCTCCCCGCCCTTCAGGGCGGGGATTGCGGATCATGTTCTTCTCTTTCAGGGATTCAACGCAGACAAGAAAGGCTT
TCAATTTCCTATACGTGAACGGCCGCGCAGCGGAAAGAAACAAGCCCGGTCAATCCGGGCTTGTTTCTTTAGGCGGCTCAGA
AATCGCCTAAAGGCCCGGCTTGGCCGGGCAGTCAGTGCTATTTAGTTTGTTGCAGCAGCTGGCTTAATTTTGCCGCCAGTGCA
TACGTCGATTGGAAAGCGCCTTGCAGGCGCTGATTAGGCAGCTGGTTGAACGCTTCGAGGCAGGCGCGCAGCAATAATTCTT
TCTGAGATTCGACTTCTTTTTTCAGTTGGGAAGGGGTGGTAACTGTAGTCATGCTTGCTCCTTAGTGAGCCGATATCGGCAATT
TTTCGGGTGGCGGTGTTGCCTCCCGATGATTTAATTATCGGTGATTATGCTTTTAAAGTCAATACAGGTACGGAATTTATTTACC
TGTTTTTATGCCCGTCAGGGCATGGAAGGCGACCGCGCCGGACTCCACCGGACACCGGCCGCAAATCGCCGGAAACTGCGG
GACTGACCGGAGCAACAGGCCAACCCCCCTCCCTGCTAAGCCATAACCCAGCCCGCCGCCACGCAGCTGCCGCACGTCCCCC
ACGGGGGTGCGCAGTGGGCGCCGCGCGCCTGCGCGCGGGTACGGCGGCCCGCCTGCGGGTCGCGGCGCCGTACTGCGAG
TTAGCGGCCGCCGCGCGGCCGGTTACGGGGGACACCGCACAGTCACGGCCAGTGCCCCGCTGAGCTGCACAATCCACGGAT
AACACAATAGCGCACTGGCAAAGGATGCCGACGCCTGAAGGGCGTGGGCACCCCGAAGGGGCGGGGCGGCCGCTTGCGG
CCGGGCGAGTCCGGCGCAGGGTGTGGCCTGCCAAGCGGAGCGCGGAGGCCGAAGGCCGGAGGCGTTAGCGGCCGCTGC
CCGCGTAAGCGGGGCGAGACGGGAACCGGCTCGATGCGCAGCACAGCAGAGCGACCCCGAAGGGGTAACGCCCGGTGTG
GCATCAGGATTTAGTGCAATGGCAGAACATGAGCTGGAGAGATCACCGGCAAGCAGCAGCAAAGGGGCGGCACAGCCGCC
CCGATGGCTGTTTGCCGATACCGGCGATTAATTAGAGCGGTGTTTAATATCCCCCGCGTTGCGGGGGACTAGGTTTCAGCAAG
TCATGTTAAATACGTGTCCATCATGTAAACTGAAATCCCCAATAAACAGATCCCGCGCATAGGCTACGATGTCAAAATATCGGGC
TACGGATTCCGGAATATCATTCAGTAGACCGCTATCATTCAGGTATTCCTCTGCAAAAGTTTCTTCGTCCTTAGCTTCGCCCATAT
AGGCATCTCTAAACAGGTCGAAATCAGTGCTATTAAACAGATCAACAAAGGCCACAAACGCCGCTTCGTTACCTTCCTCGCGG
GCTTGTTTAAAGCCGTTAATAAAATCCCAGTTGATATGGCACTCTGACGCCATACCAGACGGAATACCCTCCCAATCTTGGAAC
ATAAATTCTGGATCAGCCTCATTTGCGTGTAACTCGCGGCAGCGCTCGTAAAACTCCTCTGAGCTATCAAAATCGGTCAGATCG
AGCCAGGCTCCCGCAATGCTTCCGCAGTTGTATTTATGGTAAGTGCCAACATAAACAGAAGGGGTCGTAATATCAGTCATGGT
GTACTCCTTAAAGCGCCGATACCGGCAATTTTTCGGGCGGCGGTATTGCCTCCCGATGATTTAATTATCGTTGATTATGCTTTTA
AAGTCAATACAGATACGGAATTTATTTACCTGTTTTTATGCCCGTCAGGGCATGGAAGGCGACCGCGCCGGACTCCACCGGAC
ACCGGCCGCAAATCGCCGGAAACTGCGGGACTGACCGGAGCAACAGGCCAACCCCCCTCCCTGCTAAGCCATAACCCAGCC
CGCCGCCACGCAGCTGCCGCACGTCCCCCACGGGGGTGCGCAGTGGGCGCCGCGCGCCTGCGCGCGGGTACGGCGGCCCG
CCTGCGGGTCGCGGCGCCGTACTGCGAGTTAGCGGCCGCCGCGCGGCCGGTTACGGGGGACACCGCACCGTCACGGCCAG
CGCCCCGCTGAGCTGCACAATCCACGGATAACACAATAGCGCACTGGCAAAGGATGCCGACGCCTGAAGGGCGTTGGCACC
CCGAAGGGGCGGGGCGGCCGCTTGCGGCCGGGCGAGTCCGGCGCAGGGTGTGGCCTGCCAAGCGGAGCGCGGAGGCCG
AAGGCCGGAGGCGTTAGCGGCCGCTGCCCGCGTAAGCGGGGCGAGACGGGAACCGGCTCGATGCGCAGCACAGCAGAGC
GGCCCCGAAGGGGTAACGCCCTGTGTGGCATCAGGATTTAGCACAATGTCAGAACATAAACTGGAGAGATCACCGGCAAGC
AGCAGCAAAGGGGCGGCACAGCCGCCCCGATGGCTGTTACTTGTCTTTGTCGCGTAGCACTTTGATTAGGCCGGTTACGGCC
GTAATCAGAGCGGCCAGCGAGGTGATGATTTGCGGTAGGTTTTCGAGGATGGTAGAGGTCATATAGCACCTGTAGAGAAGTT
GGCGGGGTGTCGTTTCCGACGGCCGCACTGTAACCGGGCGAATAAGGCAGGTTGTCAACAGCTTGAGCGAAGCGTCTGTTG
ACAACCTGCCGCGCCCGGTTTCACTGCGGTCATAGGCGGAACGACCCACGCCAACGGAACGGCTTTATGACCGGGCAGCTG
AGATACCGGCGAACCTGGCTGGCGGCTGACGCCAGCCGCCAAGCGCCAGCGCGGAGGGCAAAGCCCGGAGGCCAAGCGG
AGCGCGGAGGCCGAAGGCCGGAGGCCGGAGGCGTTAGCGGCCGCTGCCCGCGTAAGCGGGGCGAGACGGGAACCGGCT
CGATGCGCAGCACAGCAGAGCGGCCCCGAAGGGGTAACGCCCGGAGTCTGCCGCTGTTTATCTCTCGTTCCATCTGAAATCG
GCGGTAAGGCCATTAAAAGGGTCAGTTTATCAGGGAGGCGTTAGCCCCCCATGTTGTTAATCATCAGGCAATATCGTCTTTGTA
GCAGGCATAACCGAAGCTAAGCTCTGTTTTCATATAGTGGCGGGCAAAGTCCCAGGCATCGTGGCCGAAGTCCTCATAATCTG
CCAGGACGATTTCGCGGGCTTTGTGCCATTGCTGTACGGAAGAAAGCGGCAGAACAGGGCAGGGGTGCGACCAGTCAGTG
ACGGTGTTGCAGATCATATCTGCCAGACGCTCCAGGGAGCCGTAAACCAGCTCTGTGCGCAGATCCGCCACCATGCGTTGTTT
ACGCAGTGATGCCAGATAATCAATCTCTTTGGTTATATCAGAATTTAAGCGGGTCTGGTAATCCATGATGTACTCCTTTGCGCGC
CGATACCGGCAATTTTGCGGGCGACGGTGTTGCCTCCCGATGATTTAATTATCGGTGATTATGCCCTCAAAGTCAATATAAGTA
CGGAATATGCATGCATAATTTTATATCTTGCAAAGCGTTCATAGAGTGCCTGAATCGCTTTCTGACAGCCTCAATAAAAAAAGG
CGGGGATTCCCGCCTTTTTTCTTACAGCTGCTTACGTGGCTTTTTACGCGTCATATACAACGGTATCGCGCAGTCTACCGCGTA

CAAAAAGCACGCCAGCGCGCCGCAACCGTACAGAAACGCAAGCGGCTTATTATCGAAGTAGCTGAAAACCCCTGTCGCCGC
ACACAGGCCCAGAACAGAGACGCAGGCGCAGGTGATCTGCACCAGATCCCTGTATCCCGCACGAACGATAAACCAGGGCAG
GGCAAGCGCAGCGGCGCTAATAATTAATGCGAGAGGGACAAAAACGAGATAGTGATACATGTGAACTCCTTGATGGTTGCCG
ATACCGGCGATTGTTCGGGCGGCGGTATTGCCACCCGATGATTTAATTAGAGGTTTTGCGCGTCCAGGAGATTGACCTGAGCC
GGGGTAACGTGAAACTTTTCCCCTTTATGGATCACGTTATGCGGGGCGCTAATTTCATCACTGATAAAGCTAACCGGGTAACGT
TTTTTACCGCAAATCCGCTCGCTAAACCATGCCACTTTTGCCGCTGGCCGATCCACTGGATGAACAATCACACCGGCCATGCTG
CAACCCGTTGCGGGTTCGTCCAGCGTAATGCTTACCGGGACTGTATCCCGGTAAAAAAACTTAACCGGCGGCACACCTGCCT
GCGTAGCGGCTGCGACAACTGCAAGCCCGATAACCGCTATCCGATTAATAAGCATTTTATTCCCCTTACTCATGCTGATATCACC
TTGCCAGCTGTTACCAGTTTACGAAATTCACTTTCATGAATTTCACGCCCATGCTCAAGCGTTGAATACACGTTGCCGATAAGC
CAGTTACCAGCCGTTTTTGTCTCGGTATACCACCATGCTTCTGTCAGAGTAAGCAGCGGTTCGCTATCCTCGCCCTTCTCATAGA
TCCAGATTTTAGTGACGTCTTTACCCGTTTCGTTGCTGCCCTGAATGGTAGGGTCAAAGGTATGTTCAATCTCTATATCGAAGTA
ACGCTGAAGGAAGTTAGTAAAGTGCATGACGACTCCTGTAAGCGCCGATACCGGCAATTTTTCGGGTGGCGGTGTCGCCTCC
CGATGATTTAATTATCGTTGATTATGCTTTTAAAGTCAATACAGGTACGGAATTTATTTACCTGTTTTTATGCCCGTCAGGGCATG
GAAGGCGACCGCGCCGGACTCCACCGGACACCGGCCGCAAATCGCCGGAAACTGCGGGACTGACCGGAGCAACAGGCCA
ACCCCCCTCCCTGCTAAGCCATAACCCAGCCCGCCGCCACGCAGCTGCCGCACGTCCCCCACGGGGGTGCGCAGTGGGCGC
CGCGCGCCTGCGCGCGGGTACGGCGGCCCGCCTGCGGGTCGCGGCGCCGTACTGCGAGTTAGCGGCCGCCGCGCGGCCGG
TTACGGGGGACACCGCACCGTCACGGCCAGCGCCCCACTGAGCTGCACAATCCACGGATAATGCAGGAGACGAATCATGATA
GGAGGCTGAAGGGGAAATGAGCGGCAGCAGGGGAAGGGGTTGCCAAGCGGAGCGCGGAGGCCGCAGGCCGGAGGCGT
CAGTGGCAGCTGCCCGCGTGAGCGGGGCGAGACGCGTAGCGGCTCGATGCGCAGCACAGCAGAACGGCCCCGGAGGGGT
GACGTCCGGGGGTTCGCTTTTAAAGATTTTCGACCACATCAGTAAATCGTAGTGACACCATGAAGCAAAAGTATCGTGCAGAC
CAGAATCAACAACAGTAACAGCAGACCTTTTTTTCGACGTAATAAAACCGGCCCCAAAGCGCCCGCAAGAAGCACACTCAAC
ACGATTTCCGTTATACTAATCGTATTCATAATCTCTCACGTTTCCCTTTTTAGAACTCTGCCACACACAGATAAAACCTTATAACA
AGCTACAAAACCCGTTATTCAGACGCGGTAATGCCTAGTTTTTTTGCCAGTATTTCAGATGACAAAGAAACCCTTATCCACTTC
CCGGTTACAAGGTGAATGATTGTGGCTTCATGCCCTTTAACAGAGTGGGATATTAACTCACGCTGAATGATATGCGTCTCTCCA
TCATTTCCCGTCACGGCAATTAGGCCCTCTGTAGAACAGGGGTTGATACTACTAATGTTGGTGTTGTTTTCGGTTTTGTTCAGC
ATCGCTGATCCTCAAATATCGGTTTGTGTTACGTCTGCCGCTTTGCGCTGGATAAGCGACTTAAAGAAATCCGACGCCTTCAGA
ATATCGCTATCCTGGAAGTCGGGAAATGTCGCTTCTGTCAGCTCCTGCGCCGTACCTGCCCGTATAACGGTTTCATCGCGGATC
ATCGTTCGCTTAACGCCATCAGCCTGTTTTTTGCTGAAGGAGTGCCGGAAAATAATCGCGTATTGGCCGCCGCTATCCTGATGA
ACAGAAGTATCCCAGCGATGATTACCCTGTTTACGATAGTGGTGATAGATTTGCTGGCCGTAGCGCTCCTGATCGGAATTACGG
TAATCGAACATTAACAGTTCCAAAAGCATTAACCGATCTGGCAATTGCCAGGCGGTAGGGTGTTGAAGTGTCGCTAACATAGT
TTCCCCTGAGCGTGACAGTCACGATAAGGCGGGCTTTGCCCGCCTGGTTATCAGTTAATCAATGGCACGATAAATACGATTCT
GGCTTTCGTTCTCCAGCGTATTAACGTACTCCCGCAACAGGTGATACCGGTTAGCCATCGTTTCGTTCAGTTCGGCTTTGCCTT
CTTCATATGCCAGGCCGCAAAAGTAACTGTAGGCATACAGACAAACAATAATCCCTACTTCGCGCGCGCTGCATTCACCTTCAA
AATAGTTAGGTAACGAGAGCCAAAGAGGTTGGGGCGCTTCCATAAAAAAACGCGCCATTGCTGGCCTGAAGGTATTCCCAATA
CCCTCCCTGGTAGTCTTTAGCGTAACGATTCAGAAAGGACTGAATGAAGTGATCTGCGCTGAAGAAAGCGCCACGAAATGCC
GCAGGCATGAAGTTCATGCGGGCGTTTTCAGAAATGTAGCGGGCGGTGATTTCGATAGTTTCCATGATACTTCCTCTTAAAGC
CGATACCGGCGATGGTTAAGCGGCAGGCACATCACCTGCCACTTTTTAATTATCGTACAATGGGGCGTTAAAGTCAATATAAGT
ACGGATTATATTTACCTAATTTTATGCCCGTCAGAGCATGGAAGGCGACCTCGCCGGACTCCACCGGACACCGGGGGCAAATC
GCCGGAAACTGCGGGACTGACCGGAGCGACAGGCCACCCCCCCTCCCTGCTAGCCCGCCGCCACGCGGCCGGTTACAGGG
GACACTGAGAAAACAGAAAGCCAACAAACACTATATATAGCGTTCGTTGGCAGCTGAAGCAGCACTACATATAGTAGAGTACC
TGTAAAACTTGCCAACCTGACCATAACAGCGATACTGTATAAGTAAACAGTGATTTGGAAGATCGCTATGAAGGTCGATATTTT
TGAAAGCTCCGGCGCCAGCCGGGTACACAGCATCCCTTTTTATCTGCAAAGAATTTCTGCGGGGTTCCCCAGCCCGGCCCAG
GGCTATGAAAAGCAGGAGTTAAACCTGCATGAGTATTGTGTTCGTCACCCTTCAGCAACTTACTTCCTACGGGTTTCTGGCTC
GTCAATGGAAGATGGCCGCATCCATGATGGTGACGTACTGGTTGTGGATCGCTCGCTGACGGCCAGCCACGGCTCAATCGTA
GTCGCCTGCATCCATAATGAATTTACCGTGAAGCGGCTACTGCTGAGGCCCAGACCCTGCCTGATGCCGATGAACAAAGATTT
TCCTGTGTACTACATTGACCCGGATAATGAGAGCGTTGAAATCTGGGGAGTGGTTACGCATTCCCTTATCGAGCATCCGGTATG

TTTGCGCTGATTGATGTCAATGGCATGTACGCCAGCTGTGAGCAGGCATTTAGGCCAGATCTGGCAAACCGAGCAGTGGCCG
TTTTATCCAACAATGACGGCAACATTGTGGCCCGTAATTACCTGGCGAAGAAAGCGGGCCTGAAAATGGGCGATCCGTACTTC
AAAGTCAGACCCATAATCGAGCGTCATAACATCGCTATTTTTAGCTCTAATTACACTCTCTATGCCTCCATGTCGGCCCGGTTCG
CGGCCGTAGTTGAGTCCCTTGCAAGCCACGTCGAACAGTATTCAATCGACGAGCTTTTTGTTGACTGCAAAGGGATAACGGC
CGCCATGAGCCTTGACGCTTTCGGGCGCCAACTGCGCGAGGAAGTCAGGCGACACACAACGCTGGTATGCGGGGTCGGTAT
TGCCCGTACTAAGACGCTGGCGAAGCTGTGTAACCACGCTGCAAAAACATGGCCCGCTACTGGCGGGGTGGTTGCTCTGGA
CGATGGCGCCAGACTGAAGAAATTAATGAGCATCCTGCCGGTTGCGGAAGTCTGGGGCGTCGGCCATCGTACAGAGAAAGC
ACTCGCCACAATGGGGATCAAAACGGTGCTGGATTTAGCCAGGGCAGATACGCGCCTAATCCGTAAAACATTCGGCGTTGTG
CTTGAAAGAACGGTACGGGAGTTGCGCGGCGAGGCTTGCTTCAGCCTGGAAGAAAACCCTCCTGCGAAGCAGCAGATTGT
TGTGTCGCGCTCATTCGGCCAACGCGTAGAAACCCTGACGGACATGCAGCAGGCTGTCACCGGATTTGCAGCGCGCGCAGC
TGAAAAACTGCGTAATGAGAGGCAATACTGCCGCGTCATAAGCGTCTTTATCCGTACCAGTCCTTATTCAGTGCGTGATACACA
GTATGCCAATCAGGCAACCGAAAAACTGACGGTGGCAACCCAGGACAGCCGCACGATAATTCAGGCAGCACAAGCCGCGCT
GGCGCGGATCTGGCGGGAAGATATTGCGTATGCAAAAGCAGGGGTCATGCTGGCAGATTTAGCGGGAAGGAGGCCCAGC
TTGATTTATTCGACTCTGCTACGCCTTCAGCTGGCAGCGAGGCTTTAATGGCTGTTCTTGATGGTATAAACCGGCGTGGAAAG
AGCCAGCTTTTTTTTGCAGGCCAGGGCATCGATAACTCCTTTGCCATGCGTCGTCAGATGTTGTCACCTGATTACACGACAGA
CTGGCGCTCAATACCAATAGCCACCATCAAATAATTACCGGCGCCGTACACGGGCCGGTTAACCCCTCAACCGGCCGAAACAA
GTTTCGGCACGGTTTCGCGGTTTTCGGTAAAAGCCGTTTCCTCTGTATAAAAGATCAGCTAAATTATGTGTATTGCACAATACAT
ATATGTGAGGTTAGCAGTGAATTTGCCTACGCCCGAAACCTACGATGAACTTCAGAGAGCCTACGATTTTTTCAATGATAAGCT
ATTCAGCAACGAGCTGCCGCCATGCCTGATAACGTTGCAGCGTGAGAAGCGAACGTATGGCTATTGTTCCTTTAAGCGTTTCG
TCGGCCGTGAGAGTGGGTACACGGTAGACGAGATCGCTATGAATCCGGTGTATTTCTCGATCAGAACCATAAAGGCCACGCTT
TCAACACTGGTGCATGAGATGGTTCATCAGTGGCAATTCCATTTTGGCGAGCCTGGCCGCCGTGGCTATCACAACAAACAGTG
GGCGGCCCGGATGGAACGGGTAGGACTAATGCCTTCTGATACCGGCGAACCGGGAGGCAGGAAAGTGGGCCAGAGCATGA
CCCATTATATTATTGCCGGTGGCCCTTTCGATATGGCCTGTGATGAACTGCTGACAGGCCATTTCCGGCTTTCCTGGATGGACA
GGTTTCCGCCTTACCAGCCTAAGCCTGGCGCTGTGCTAAGCCCTACAGGAAAAGGCTATATTGACGACGAGGAAGATGATAG
CGAACACGAACAGGAGGTGGAGGAAGGGCGCGACCCGGTTGAACTCGACGACGAGATCATAGAGGCCATGCGATTTGTAA
CCCCACCGCCTGAAGCACCGGTGAACAAAACAAACCGGGAAAAGTACAGCTGCCCGGTGTGTCATATCAATCTCTGGGGTAA
ACCGGGGATAGTGGTTTACTGTGGTGGCGAGCACTGTAATAAAGCCGCGTTAGTAGTCTTAAAATAAAGTCCTTTCGGACTTT
ATTTTTTTTCCATTTCCGAGGTCGTGATGTTATTAATGCTGTACTTCGCGGCTTCTTTTAAAACAGTTTCAGCAAGGCTTGCTGG
TATCCAGACCTGAACTAATTTTAATGGTTCGCCGTTCTCGGCTTTAAGAGTGGTGTTCTGGTACAAATCCCAGATTCGCTTAAC
GGTGCTGGAAATGTTTTGCTTGGAACGGCCTACTCGCATGGCTACGTCTGATGATTTCTCACCTTTGACAAGCACGGAATAGC
CAATATCTGTTGTGATGTGTGCAAAGGAAGCCATTTGCGGCAGCAGCTGTTTCCATTCTGTTTCTGAAATTCTGTTTTTCTGAG
CCATCTGTGGCGCCTCCGTAGTTTTGGTTACAGAAAGGATATACTCAGAATAAACAGGGGTCAATACAAGTACGATTTTTATAA
ACTTTATTTTATTTGAGGGTGAGGCCCGGTGCGGCAGCAGCGCGGGCCTCGATGGTGCCGCGAAGGTGCTGGCGCCATGCT
CGGATTAAAACATGAACCGTGAAGAACTGCGAAACTTGTTTTCGCGGTTCTGAGGGGTTGACCGAGCCGCGAAGCGGCGCT
GGTAAGCGATGATATGCACATATCCACAGGCATATTTTTAAAAGGTATTTTATAGATTTTTTATCTTTTTAAAGTCTTTTAGAGCT
ATATAACTCATTGATTTAAAATCATAAATAAGTGTTATCTCTGGGAATCCGCCCACCTTGTTATGGGAATTGGCCCACCTTACTAT
GGGAAACAGCCCACCTTACTATGGGAATTAGCCCACCTTGTTATG

>pQEB1_inv1_inv2

GGAATTGGCCCACCTTAGACGAAACTGTAAAAAATGTATTTACTTGTTTGAACTTTGTGGTAGTGTGGAGAGTAATTTTTAACC
CACAAAGGCAAGGCGCATGGATAAGTTGCTGAACAAAAAGATAAAAGTTAAGCAGTCTAACGAGCTTACCGAAGCTGCTTAC
TACCTCTCGCTAAAAGCAAAGCGCGTTCTCTGGTTATGTCTTATGCAGACGTATTTCACAGCTTCAGTAAGCGAAGATGATGAT

GAGATGGCTGTACTCGGTGACTCTACTTTCAAAGTAAAGGTGGCTGACTATCAGCAAATTTTTCAGGTAAGCCGTAACCAGGC
TATCAAGGATGTTAAAGAAGGCGTGTTTGAGTTAAGCCGTTCTGCGGTAATCTTTTACCCGAAAGAGGGGCGTTTTGACTGC
GTCGCGCGCCCCTGGCTAACAGAGGCTGGCAGCCGATCAGCTCGTGGTATCTGGGAAATCGAATTTAACCATAAACTCCTGC
GGTACATTTACGGCCTGACGAACCAGTTCACCACCTACTCGCTCCGCGATTGTGGCAGTCTTCGAAATCCCCGGACGATCCGC
CTTTATGAAAGTCTTGCTCAATTCAAATCTTCAGGCTTATGGGTTACTACTCATGCTTGGTTAAATGACCGTTTCCTTTTGCCGG
AATCCCAACAGAAGAACTTGGCAGAGTTGAAACGATCTTTCCTTGATCCTGCACTCAAGCAGATAAATGAGAAAACACCTTTA
CTTGCTAAGTATAGTATTGATGATTCAGGAAAATTTCTGTTCTCAATAATTGATAAGCAAAATCCCGTCTGACATAAATCAGCAC
ACATGAGCCTGTCATTTGACAAATTTTTGTCATGAAGATGGGCGAATTTCCACACAGCACCGGCGCCCGGCAAGATGGGCGG
ATTCCCACACGACAGCGGCGCCCGGCAAGATGGGCGGATTTCCACACTACAGCGGCGCCCGGCAAGATGGGCGGATTTCCA
CACGGCAGCGGCGCCCGGCAAGGTGGGCGGATTTCCACACGGCAGCGGCGCCCGGCAAGGTGGGCGGATTCTCACGCGG
CAGCGGCGCCCGGCAAGATGGGCGGATTTCCACACGGCAGCGGCGCCCGGCAAGGTGGGCGGATTCTCACGCGGCAGCG
GCGCCCGGCAAGGTGGGCCGATTCCCACGCGGCAGCGGCGCCCGGCAAGGTGGGCCGATTCCCACGCGGCAGCGGCGCC
CGGCAAGGTGGGCCGATTCCCACGCGGCAGCGGCGCCCGGTAAGGTGGGCGGATTTCCACACGGCTGCCGCGCCCGGCAA
GGTGGGCGGATTTCCACACGGCAGCGGCGCCCGGCAAGGTGGGCGGATTCTCACGCGGCAGCGGCGCCCGGCAAGATGG
GCGGATTTCCACACGGCAGCGGCGCCCGGCAAGGTGGGCGGATTCTCACGCGGCAGCGGCGCCCGGCAAGATGGGCGGA
TTTCCACACGGCAGCGGCGCCCGGCAAGGTGGGCGGATTCCCACACGGCAGCCTCGCCCGGCAAGGTGGGCGGATTCCCA
CACGGCAGCCTCGCCCGGCAAGGTGGGCCGATTCCCACGCGGCAGCCTCGCCCGGCAAGGTGGGCGGATTCCCACACGGC
ACCGGCGTGCGGCAAGGTGGGCGGATTCCCACACGGCACCGGCGCGCGGCAAGGTGGGCCGATTCCCACACGGCACCGG
CGCCCGGCAAGGTGGGCCGATTCCCACACGGCAGCCGCGCCCGGCAAGGTGGGCCGATTCCCACGCGGCAGCCTCGCCCG
GCAAGGTGGGCCGATTCCCACGCGGCAGCCTCGCCCGGCAAGGTGGGCCGATTCCCACACGGCAGCGGCGCCCGGCAAGG
TGGGCGGATTTCCACACGGCAGCGGCGCGGGGCCAGTGGGATTCAGGAGAATAGGTGTTTTACCGAATGCCCTGACGAGG
CGTAAAAAAACCGCTTGGCGGCGGCCTCATAAAGCAGAAAACCCGCTCAAGGCGGGTTATCTGCTCTGTAGCCTGTGATGCT
TCGCGGGCATCCGGCATACAGCGAGGTGAAATTCTTCTTTTGGCATGTTAATTATACGTCTAACGCGGCATATGATCAAACCTG
TATTAAATAAGCCACTGTACCGTTTATAATGCTCTCAGATCAAAGAGGTAAAGCCCGTTTAGCCGCCTGTGTGATGAGCCAGTT
CAGACTCTTCAAAATCGAATTTGGTACTAAACAGGACCCGAACCGTGGGCAAGCACACGGCAACGGTATAGCCCTCTTCCGG
TTTCGCACCCGGAAGCCTGGGCGGCAGCGTGGTGAAATTCTTCTTTTGGTTAAGTGAATGGCATAACCGGATGGGCGGATTA
GAGGAAAGGGGATTGCCTAGTAACCTACGCGCCACAGAGATGGAGGTGCGGGGAATGATTGAGCTGATTATCGCTATTCTGA
CCTTAATTGCGGCTGTATTGCAGTTGATCAACTGGTTCCTTTAATGGTGCCGGAGTCTGTGAAGGTGAAAGCCTGAACGGGC
AAAACTGAAAGGTTTATAGCCGTCCTTCGGGGCGGCTTTTTTTCGGCAAAATTAGGGTTTTACCGAATAATGCAGAGTTTTAA
GGTGAGAATTTGCAGACTTGGCGTTTTACCGAACATAGATACTCCCTAGGCTGATAGGTGCATTAGTTATCACCCTACCTGAAC
ATATTGTAAAAGATGTCAGTCTCCAGTGACTTGTGTACTATCAACTGACAAGACTCTTACACGCAACGCAGGGGGATGGAGTT
TTATGCTTAGAAAAATAATCAGGGGTAGCGGATTCACTCAGTCAGAAGAAAAACTGATAGAGTTCGCTGATGATGCTTTTTTT
GGTCTTTGGTCTTATCCTAATGTTTATAGCGATGAGGGTTACTCTAAAAATAAAATTGGGAAAGAAGTTAGTGACTTATTAGTTA
TTTTTGATAAAGATATAATAATTTTTTCCGATAAAGCTATTACATACAATAAAAACAAAGATCCTAAGGTTGCATGGCAGAGATG
GTTTAAAAAATCAGTCATACAGTCTTGCACACAGTTATTTGGCGCAGAGAAGTTTATAAAAGATCATCCCGAAAGACTTTTTGT
TGACAAAGAATGCTCAGTTAACCTCCCCATTAAAATAGATAATTCTTTTAATTTTCATTTGGTGGCCGTCACTAATAATATTTCAG
ATCCGGCGATCTCGTACTTTGACAAAATAGAAAAAGGCAGCTCTGCTACTTTAGTTAACATATTTCCTTTAAACGCCCATCAATG
TCTAGAAAATCCATTTTGTGTCGGAGACGTTTATCCTGATAAGACTTTTGTCCATATACTTGATGAGACTGCCCTAAAACTACTG
TTAACCGAGTTAAACACAGCAACTGATTTCATTGGCTACCTTAACGAAAAAGAGAGGGTTGTAAGAGAAAGAACATTATTGG
TCAGCGCTGGGGAAGAAGAGACTCTTGCTGCTTACATTATGGGTGATAAAACCATAATATCAAAAGAAATTATTGGAAACGAT
CAAGGGATGACCATACCGGAAGGTGAATGGAAAAACTATAAAACCACTTTCAATTATCAATATCAGCTCTCAATGAAAAAGGG
TAGCGTTTTCTGGGATAACCTAATCCACAACTTCTCGACAAGTATATTGTCAGCTAACGTTGGTTTTTTTAGTGAAATTGAATTT
TCTACACATGAATTAGGTGTTAGAGAATTAGCCAAAGAAAGTAGGCAATCTAGATATTACCTTTCAAAGAACTTTAAAGAGAA
ATTAAAAACAACTCAGCCTCATCTAAGAACGTCAAGAATGGTCGAATCAATCGATGAGCCTGGAAAGTTTTACTTATTCCTTTT
TTTTCCTAACGATAGCAAGTTGAGTTACTCTGATTACAGAATTCAACGTATATCTTATATAAATGCTTATGCTGAGGTTGCCTTTA
ATAAATACAGACATATTAAAAAATTAATTACTATTGCAACAGAGCCGCAAAATACAGAAGGAAGATCTGAAGACCTAATATATA

GCATATCCCCAGAGAAATTTACCAAAGAGCAAAATGAAAAAGCCAAAAGATTATCAAGAGAATACAAAATACTAAGTGATTTT
TTACCTACTAAAACGACAAAGAGCGATAACTTTAAATCAGTTATATCAAAAGGTGAAAAAATAGGGCGGAATACACCTTGTCC
ATGTGGCTCCGGTGTTAAATTTAAAAAGTGCCATGGTGCGAATAATTAGCATTATTGTATGTATAACGGTAATGGCGCGGCAGA
GAAACCGGCGCGTTCTGCCCTAGTGTTGGCCTGCGGGTTCCCCCGCACCCGCTGTATGTAGTATCGGCAGCATCTGAGAAAA
CCACTACATGTAGTTATCAGCGCCACAACGGCGCGGGGACGAGTGCGGTTCGGAAAAATTGGGGTTTTACCGAATCCGGCA
AAAGATTGCTTCCTATAACGTCCGCTTCTGGCACACAGCAGCCGTTAAGATGTAAGGCCTTACGCCAACTAAATCTAATGGGA
CAGATTTAGTTGGTGATGGTCAAGTAATCTGCAAACGGTCACCAAGTAAAATGCAAATGGGTAGTCAAGTCCGATGCAATTAC
GCACCCGGCAAGGTGGGCCGATTCCCACACGACAGCAGCGCCCGGCAAGGTGGGCGGATTTCCACACGGCAGCGGCGCCC
GGCAAGGTGGGCCTATTCCCACACGGCAGCGGCGCCCGGCAAGGAGGGCCGATTCCCACACAGCACCGGCGCGCGGCAA
GGTGGGCCGATTCCCACACGACAGCAGCGCCCGGCAAGGTGGGCGGATTTCCACACAGCACCGGCGCCCGGCAAGGTGG
GCCGATTCCCACACGGCAGCGGCGCCCGGCAAGGTGGGCCGATTCCCACACGGCAGCGGCGCCCGGCAAGATGGGCGGAT
TTCCACACAGCACCGGCGCCCGGCAAGGTGGGCCGATTCCCACACGGCAGCGGCGCCCGGCAAGGTGGGCGGATTCCCAC
ACGGCAGCGGCGCCCGGCAAGATGGGCGGATTCCCACACGGCAGCGGCGCCCGGCAAGATGGGCGGATTTCCACACGGCA
GCGGCGCCCGGCAAGATGGGCGGATTCCCACACGGCAGCGGCGCCCGGCAAGATGGGCGGATTTCCACACGGCAGCGGC
GCCCGGCAAGGTGGGCCGATTCCCACACGACAGCGGCGCCCGGCAAGGTGGGCCGATTCCCACACGACAGCGGCGCCCGG
CAAGATGGGCGGATTCCCATATCGACATGTATGTAGCTTGTGTTATCCGTGGATTGTGCAGCTCAGCGGGTCGCTTGTCGTATG
GCGTAGTGTCCCCCGTAACCGGCCGCGTGCGGCCGCTAACGCGCAGTACGGCGCCGCGACCCGAAGGCGGGCCGCCGTTCC
CGCGCGCAGGCGCGCGGCGCCCACTGCGCACCCCCGTGGGGGACGTGCGGCAGCTGTGTGGCGGTGAGCGGGATTAGGG
CTTTGCAGGGAGGGGGCTGGGTCGGGCGATACGTTCAGCATTGCGGTTTCCGGCGATTTGCGGCCGGTGCCCGTTTAACTC
CGGCGTGGTCGCCTTCCATGCCCTGACGGCATAAGAAAATAAAACCGCCATGCTGCGGTCATTCATGATTTTGTGGTGTAGCG
ATAAATAGTCATGCGAGAAACGTTGAAGCGCTTAGCAACTGCACCAACTGTCATTTCAGGATCAGCAAGTAAGATTCTAATTT
GTTTAACATCTTCTTCAGAAAGTGACGGTTTTCTCCCTCCCACACGGCCCCTTGCGCGTGCAGCTGCAAGGCCTGAGCGCGTT
CTTTCAATATTGCGGTTGCGTTCAAAGCTAGAGAATATCGCCATCAGATGAGTATAGATTTCCCCTATAACTGGCGCATTTGTGT
CTATTCTGTCCTTGATGGCTATGAAAGTTATTCCGCGTTTCTTCAGGTTGTCGAGTAAAGTAATGACTTGACCCAATGAACCACC
GAGCCGATCTAGTGCCCAAACTACTAGGGTATCTCCCTCGCGCAATGCTTTCAGGCAGTTCTCCAGTTCCAGCGCACCTTTTTT
GTCGCGCTTTGGGCCGCTACGTGAGGTCTGATCCTGATAGATTTGCTCACATCCAGCTTTTGTTAGTTCGTCAACCTGGTGCGC
CACATCCTGAAGATGCGTAGATTTACGGGCACTGTTGCAAATAGTCGGTGGTGATAAACTTATCATCCCCTTTTGCTGATGGAG
CTGCACATGAACCCATTCAAAGGCCGGCATTTTCAGCGTGACATCATTCTGTGGGCCGTACGCTGGTACTGCAAATACGGCAT
CAGTTACCGTGAGCTGCAGGAGATGCTGGCTGAACGCGGAGTGAATGTCGATCACTCCACGATTTACCGCTGGGTTCAGCGT
TATGCGCCTGAAATGGAAAAACGGCTGCGCTGGTACTGGCGTAACCCTTCCGATCTTTGCCCGTGGCACATGGATGAAACCTA
CGTGAAGGTCAATGGCCGCTGGGCGTATCTGTACCGGGCCGTCGACAGCCGGGGCCGCACTGTCGATTTTTATCTCTCCTCCC
GTCGTAACAGCAAAGCTGCATACCGGTTTCTGGGTAAAATCCTCAACAACGTGAAGAAGTGGCAGATCCCGCGATTCATCAA
CACGGATAAAGCGCCCGCCTATGGTCGCGCGCTTGCTCTGCTCAAACGCGAAGGCCGGTGCCCGTCTGACGTTGAACACCGA
CAGATTAAGTACCGGAACAACGTGATTGAATGCGATCATGGCAAACTGAAACGGATAATCGGCGCCACGCTGGGATTTAAATC
CATGAAGACGGCTTACGCCACCATCAAAGGTATTGAGGTGATGCGTGCACTACGCAAAGGCCAGGCCTCAGCATTTTATTATG
GTGATCCCCTGGGCGAAATGCGCCTGGTAAGCAGAGTTTTTGAAATGTAAGGCCTTTGAATAAGACAAAAGGCTGCCTCATC
GCTAACTTTGCAACAGTGCCCCTTTTAAAAATATGCCTGTGGATATGTGCATATCATCGCTTACCAGCGCCGCTTCGCGGCTCG
GTCAACCCCTCAGAACCGCGAAAACAAGTTTCGCAGTTCTTCACGGTTCATGTTTTAATCCGAGCATGGCGCCAGCACCTTCG
CGGCACCATCGAGGCCCGCGCTGCTGCCGCACCGGGCCTCACCCTCAAATAAAATAAAGTTTATAAAAATCGTACTTGTATTG
ACCCCTGTTTATTCTGAGTATATCCTTTCTGTAACCAAAACTACGGAGGCGCCACAGATGGCTCAGAAAAACAGAATTTCAGA
AACAGAATGGAAACAGCTGCTGCCGCAAATGGCTTCCTTTGCACACATCACAACAGATATTGGCTATTCCGTGCTTGTCAAAG
GTGAGAAATCATCAGACGTAGCCATGCGAGTAGGCCGTTCCAAGCAAAACATTTCCAGCACCGTTAAGCGAATCTGGGATTT
GTACCAGAACACCACTCTTAAAGCCGAGAACGGCGAACCATTAAAATTAGTTCAGGTCTGGATACCAGCAAGCCTTGCTGAA
ACTGTTTTAAAAGAAGCCGCGAAGTACAGCATTAATAACATCACGACCTCGGAAATGGAAAAAAAATAAAGTCCGAAAGGAC
TTTATTTTAAGACTACTAACGCGGCTTTATTACAGTGCTCGCCACCACAGTAAACCACTATCCCCGGTTTACCCCAGAGATTGAT
ATGACACACCGGGCAGCTGTACTTTTCCCGGTTTGTTTTGTTCACCGGTGCTTCAGGCGGTGGGGTTACAAATCGCATGGCCT

CTATGATCTCGTCGTCGAGTTCAACCGGGTCGCGCCCTTCCTCCACCTCCTGTTCGTGTTCGCTATCATCTTCCTCGTCGTCAAT
ATAGCCTTTTCCTGTAGGGCTTAGCACAGCGCCAGGCTTAGGCTGGTAAGGCGGAAACCTGTCCATCCAGGAAAGCCGGAA
ATGGCCTGTCAGCAGTTCATCACAGGCCATATCGAAAGGGCCACCGGCAATAATATAATGGGTCATGCTCTGGCCCACTTTCCT
GCCTCCCGGTTCGCCGGTATCAGAAGGCATTAGTCCTACCCGTTCCATCCGGGCCGCCCACTGTTTGTTGTGATAGCCACGGC
GGCCAGGCTCGCCAAAATGGAATTGCCACTGATGAACCATCTCATGCACCAGTGTTGAAAGCGTGGCCTTTATGGTTCTGATC
GAGAAATACACCGGATTCATAGCGATCTCGTCTACCGTGTACCCACTCTCACGGCCGACGAAACGCTTAAAGGAACAATAGCC
ATACGTTCGCTTCTCACGCTGCAACGTTATCAGGCATGGCGGCAGCTCGTTGCTGAATAGCTTCTCATTGAAAAAATCGTAGGC
TCTCTGAAGTTCATCGTAGGTTTCGGGCGTAGGCAAATTCACTGCTAACCTCACATATATGTATTGTGCAATACACATAATTTAG
CTGATCTTTTATACAGAGGAAACGGCTTTTAGGGGTTGTTTGCGGGAGAGGGCGAAATCCTACGCTAAGGCTTTGGCCAACG
ATATTCTCCGGTAAGATTGATGTGTTCCCAGGGGATAGGAGAAGTCGCTTGATATCTAGTATGACGTCTGTCGCACCTGCTTGA
TCGCGGCCGCGATAGCTAGATCGCGTTGCTCCTCTTCTCCATCCGCGTTCCAAGCTGCGGAAAGGCACCCATAAGCGTACGCC
TGGTCGAGCAGGCGACGCGGATCGACGTCCAGCGCACGAGAGAATGCGTCCGCCATCTGTGCAATGCGTCTAGGATCGAGA
CAAAGGTTGTCTCTGTCAGCCGGATCGTAGAACATATTGGCGGCGCCAAAGCCCACTTCACCGACCAGACCGACGGGATCTA
TCACCAGCCAGCCGCGACTGGAGAACATGATGTTTTCATGATGCAGATCGCCATGTAGCCCACGCAGTTCCGAGGCATTGCTC
ATCATTTGATCGGCTATAATCGCCGCGTGGACGTAGTCAGTTTGACAACCTGCGTTTTGATCATCGCGCGCCCGCTGAAACAA
AGCTGCAAAGCGATCCCGGATCGGGAGAAGGGCAGAAGGCAGGGGTTCCTCAGATGCGGCATACAGCTTCGCCATTAGTTC
CGCTGCAATTTCGGTCGCCTGGTAGTCGCCGTGCTCGGCAACGATGTGAGAGAGCATTCGCTCCCCGGCATATTCGAGCAAC
ATCAGATTGTTCTCACGACCGAGCAACCGGACTGCTCCCCTCCCATTGCGCCATACCAGATAGTCGGCCCCGCGCAGTTCATC
AGCAATGTCTTCTATAGGTTTCAATCCCTTGACGATTGCAGGAGTCCCGTCTGGCAATGAAACTTTCCAAACGAGGCTGGAAA
AGGTGTCCGCAATGAGAACAGGTTGCGAAACGTGCCAATGAGCAGGAAAAACAGGCGGCATGAACATCAACCCCAAGTCA
GAGGGTCCAATCGCAGATAGAAGGCAAGGCGTTCGCGGTCGGGGGCTTCGATCCCCAATACATTGAATAGGACAGCGAAGG
CGCGCTCTGCTTCATCTGGCGCTGCCCAGTTCTCTTCGGCGTTAGCAATCATGAGTGCCAAATCGGCATAGCGATCTGCTGTTC
CGAGCCGCCCAAGGTCGATCAGACCCGTGCATTGAAGAGTTTTAGGGTCCACCATGAAGTTCGGCATGCAGGGATCACCATG
GCAAACAACCATATCGGTGCGCTCTTGGTCGAGCCGCACCGGTAGCTCTCGTTCGACACGAGCCAAAAGATCGAGCTGCGGC
GTACTCTTGTCCTCGTCCGGTAAGAAGTCGGGATTGACGGCATTGCGGGACACCACATCAACGGCGCGTCCGAACATTCGCG
ACAGCCTGCGCTCAAACGGACATTGATCAACCGATAGGCTGTGAACAGCGCCAAGTTGCTGCCCCATTGACGGCCACGCTTT
GAGCAAATCCGCTCCAGACAGATCAGCCGCCGGTACTCCCGGAATTGCCGTTATCACCAAGCATGCACCCTCCTGTTCCTCCT
GCCAGTTGATCACCTCGGGGCAAGCCACACCTCGACCTTTGAGCCAAATGAGGCGGTCACGCTCTCCAGCGAGCTCACCGC
GGCGGGAAGCAGGTGCGATTTTCGCGAAGGCATGCCCGTCACCACGTCGAAAAACAAAATCACCAGATTCTCCGCCTCTGA
CAGGCAACCAGTCAGAATGCGATTCACCAAAAAAAATATTAGTTCGATTCAATGGAGGTTCCTTCAGTTTTCTGATGAAGCGC
GGAGGTGGCTCAACCTGCGAAAAGAAACGAGTTGCTACGTAAGTCCGAGAACATGCTTTCCATGGTCTCTGAGCTCGCCTTG
ATGCCCGAGGCATAGACTGTACAAAAAAACAGTCATAACAAGCCATGAAAACCGCCACTGCGCCGTTACCACCGCTGCGTTC
GGTCAAGGTTCTGGACCAGTTGCGTGAGCGCATACGCTACTTGCATTACAGCTTACGAACCGAACAGGCTTATGTCCACTGG
GTTCGTGCCTTCATCCGTTTCCACGGTGTGCGTCACCCGGCAACCTTGGGCAGCAGCGAAGTCGAGGCATTTCTGTCCTGGC
TGGCGAACGAGCGCAAGGTTTCGGTCTCCACGCATCGTCAGGCATTGGCGGCCTTGCTGTTCTTCTACGGCAAGGTGCTGTG
CACGGATCTGCCCTGGCTTCAGGAGATCGGAAGACCTCGGCCGTCGCGGCGCTTGCCGGTGGTGCTGACCCCGGATGAAGT
GGTTCGCATCCTCGGTTTTCTGGAAGGCGAGCATCGTTTGTTCGCCCAGCTTCTGTATGGAACGGGCATGCGGATCAGTGAG
GGTTTGCAACTGCGGGTCAAGGATCTGGATTTCGATCACGGCACGATCATCGTGCGGGAGGGCAAGGGCTCCAAGGATCGG
GCCTTGATGTTACCCGAGAGCTTGGCACCCAGCCTGCGCGAGCAGCTGTCGCGTGCACGGGCATGGTGGCTGAAGGACCAG
GCCGAGGGCCGCAGCGGCGTTGCGCTTCCCGACGCCCTTGAGCGGAAGTATCCGCGCGCCGGGCATTCCTGGCCGTGGTTC
TGGGTTTTTGCGCAGCACACGCATTCGACCGATCCACGGAGCGGTGTCGTGCGTCGCCATCACATGTATGACCAGACCTTTCA
GCGCGCCTTCAAACGTGCCGTAGAACAAGCAGGCATCACGAAGCCCGCCACACCGCACACCCTCCGCCACTCGTTCGCGAC
GGCCTTGCTCCGCAGCGGTTACGACATTCGAACCGTGCAGGATCTGCTCGGCCATTCCGACGTCTCTACGACGATGATTTACA
CGCATGTGCTGAAAGTTGGCGGTGCCGGAGTGCGCTCACCGCTTGATGCGCTGCCGCCCCTCACTAGTGAGAGGTAGGGCA
GCGCAAGTCAATCCTGGCGGATTCACTACCCCTGCGCGAAGGCCATCGGTGCCGCATCGAACGGCCGGTTGCGGAAAGTCC
TCCCTGCGTCCGCTGATGGCCGGCAGCAGCCCGTCGTTGCCTGATGGATCCAACCCCTCCGCTGCTATAGTGCAGTCGGCTTC

TGACGTTCAGTGCAGCCGTCTTCTGAAAACGACAAACGATGTCAGCCAATAAGTTGTTGTAATAATCGACAAGTGTTTGTTGC
AACTCGCCGTCCTGTAAGACTGCATTATCAGTTTGATTCAGGGCGGCTATGGTGTCATTTATACACCATAAACCGATGACGGCT
GCACGTAAATCTGTGGATGCTCTTGATGGCGATTCAACGTTTGCTGTCCCACGCATGGCGACAGCATTGTTTAGCCCAGCAAT
AATCCCATTATAAGTTGGGGAGGGGCTCCGACAACTGCAAAGCTATCTTTTCTGTATTGTTTTGGATGTCGTCCGCAAGAATG
GTATTCTCGATCAGTTCTCGAGCCAATAATGATGTTTCGAAGCAGACGGCCAACACCGTCTGCTTCGTTTCTGGTTCTTGTTCT
TCCACTGCCAGCGCGTTACTGATTGTTACGCACGTAAGTAGCAAAGTGAATGCGCTCTTGAGTCTCATCAACGGTTGCCTCCA
CAGTCTCGCTGGCACTGGAATCAATGTGAAAATTGTTCTGGATCGGATCAACAGGAATAAATCGATCACAGTCGAACTCTCTT
GGAATTATGGTCATGTGCAGTTTCGTTGCTGATGAAAGAGCTTGAATGTACAACTTCTCCCCACCAATGACAGCAATGCGTTG
ATCGGTATTCTCATACAGGTTGATCACAGCACGAAGATCAGGAACGACAGTAGCGCCTATGACATCTTCTTGTTTTAACGTCGA
GGAGATCACGAACGATTCGCGTTCGGGGAGAATTCCTTTCTCCTTGATTCGCTCCTCGGCGCCATCCTTTTCTAACTGCATGTC
ACGCATGTCAGTATACGTGTGTTTCCCCATAACGCACACGCGGATCTCTTTAGAAATCCGCGTAAATCGCGCCATGTCTTCTTTG
CATTTCCATGGAATCTTGCCGCCTTTCCCGAATCCCAACTTAGAATCCACAGCGACTATTAGCTCAAGTTGTGGGTGACTCATT
AAAACTCCAAACCAGTCGATGCTGGTTGTTCCAGATATTGCTTACGGAAGGTGGCCAGATCAATCAGTTTGATATTTGCCTTTC
CGCTGTTGTTGAGATCGTGCACTTTCTTCAATTTGCCGGAAGATCCGGAAGTGGATGCCGCGATCACCATGGTGGTCTTCGAC
GAGACTGATGATTGCACTTCACCACCCTCAGCTTCAATCAGCTTCTCCAAGGCCGCGTCACGAAAACCGGTGATCACGATCTT
CTCACCGGTAAAACGATCCCCCGAAGTGACCTGCTGTTTGAACCCGATGATTGATTGAATCTTGTTGAATAGCTCGGCATGTTC
AGCCATACCTGCAAGCACTTTTGCGGCAGTTTTGTAGCTGAATTTGTCGACGCTAACGATCTGCTCTTCGGTGATGGCATTGA
AATCATCCAAGGTCTTAATATCCAAGCCTTCACACAGAGCTTTCATCTTGCGGCGCCCAACGCCGCGCCCAAAGTGGGGCATC
GACCCCAACCAAACCGACAGGGGAACACTACTGGGTATAGGAAGTATAAACCACCTTTTTGCTCCTCATCCGAAGTATCTTAC
CTGAAATTCCCTCACTCGTTTACCGCTCAAGCCCCAATTTTAACTGCCGGTCCAGCCTAAACCGCTCTAATAAGGTTCGATTTG
GCGGTAAAATCTCTAGCCTGATAGCTCGAGAGATACAAACTGCCCCACCGCCCCGTTTAAAAGTTGGCAGTGTTGAGCAGTG
TTGGATTTGGGGTCGTCAGTCAAAGAGACGACTCTGTGATGGATCGAACAGGCTGGGAGTCAGTGGCGGCGCTCGTTCTGG
TGGCAGCTCACGCTGCTTGGCGGCATTCGCCTTGGCTGTTTTCTGTTTCAGATGCTTGAGAATCTGCTCAATGACCTTCGGATC
TTCGATGCTGGCAATCACTTTGACGTGACCGCCGCAGTGTTCGCAGACTTCAATATCAATATTGAAGACTCGCTTGAGGCGTT
GCATCCAGGTCATGGCGCGGTGGCGCTCTGCAGGACTCTTGTCACGCCAGTTAGTATCGAGACCTTCCGATTTGTCGGGCTTC
TTGCCCCGCTTGGCGGGTGTTACTTGAACTCGGTGTTTGCTGTTCGGTGCAAAGACGCCGTGGAAGCGTGTGAGGTTGACTC
GCGGCTTAGGTACCAACGCAGCGAGTTTGGCGATGAAGTCCAGCGGCTCGAAGATCACATGGGTGGTGCCATTGCGGTACG
GAGTTTTGAGCTCGTAACGCACCTGCCCATTGGCGGTTAATGCCAGACGTTTTTCTGAAACCGCTGGCCGACTAATGTAGCGA
CACAAGCGCTCAAGCTTATCCCGCTGATGCGCTTCGGCCATCACACCGGCGTGTAGCGAGAAACCAGCATGGTTGGCTACTC
GACTGCTTGAGTCGGCTTTATCCTCACGCCCTGGCAAGGTTTGCAGGGTGAAGACTTTGCGCCCTTGCTGGGGGCCGACGG
CAATGCGATACGTAACCGAAGCACCATGTAATTGAGTCAGCGTATCGTCTTCGCCCTCTTCCAGTGTCAACCACGTATTCTCGG
CATCACGCTCCAAAATCCCACGCTTTTCCATGCAGCGAGCGATGCGATGGCTGAGGGTGTGAGCGAGCGTATTCAGCTCATCG
TAAGTGGGTGCCTTGACACGATGGAAGCGTTGCTTGCCATAGTCATCTTCGGCATAGACACCATCGAGAAACAGCATGTGGTA
GTGGACATTGAGATTTAGCGCGGAGCCAAAGCGTTGGATAAGAGTCACTGAGCCAGTTTGTGCAGAGGCTTTGGTGTAACC
GGCTTTTTTGATCAGATGAGTTGAGAGTGTACGATAGACGATACTCAAGACCTGGCCCATCAGCTGGGGATGGCGAGCCAGC
AAAAAGCGTAGCTGGAAAGGAAAGCTGAGCACCCACTGGCGAATGGGCTCCTTGGGGAAGACTTCGTCTATCAGCAGCGCC
GCACTCTCGGCCATCCGGCGGGCACCGCAGCTAGGGCAAAAGCCGCGTCGTTTACAGCTGAAGGCGACCAGACGCTCGTGA
TGACAATCCTCGCAGCGAACCCGCATGAAACCATACTCCAGACGGCCACATTGGAGGAGGTCGTTGAATTCTTGTTGGATGTA
GCGAGGCAGGTGTTGACCTTGGGCTTCGAGTGAGGCTTTGAAGGCTGGGTAGTGCTGCTCAACCAGCTGGTAGAGCAGCG
TCTGGTCGGGTTGGTGGCGTTCGTAACCGTTTGTTTGAGTGGGCGATTGACTCGCCGTGGCGTTCCTTGCCAGCGACATGGG
TATCCTCCGCTGATACTGTGGTTATGTACAGTATCAGCGGCTTGCGTTCAGACGTCCAGTCTGGCCCTAGACATCGCTAAATGCT
TAACCCGCAATAGCCCTCACGAGTTGTTATCAGCCACTACCGGTTGAGCGAGAAGGTTTTGGGTTCAGGGTGCTATTGCTCCA
CCAATCACAATACTGAAGCCCCAACTGTTATCAGTTGGGGCTTTTTCTTGTCTGTTTGCGGCGGTTGCGTTTTATCGGTAGTCG
TCGAGCTCTGCACCATCCCACATAAGAGCTTAACGGTGCGATCTTCAACGCCATCACACAAAACTTTCTTTTTCACGCACAGTC
AACTTATTGGATGTTTTATTAACAACCCAAAAGGAGATATTTAGCGGGCGGCCGGAAGGTGAATGCTAGGCATGATCTAACCC
TCGGTCTCTGGCGTCGCGACTGCGAAATTTCGCGAGGGTTTCCGAGAAGGTGATTGCGCTTCGCAGATCTCCAGGCGCGTG

GGTGCGGACGTAGTCAGCGCCATTGCCGATCGCGTGAAGTTCCGCCGCAAGGCTCGCTGGACCCAGATCCTTTACAGGAAG
GCCAACGGTGGCGCCCAAGAAGGATTTCCGCGACACCGAGACCAATAGCGGAAGCCCCAACGCCGACTTCAGCTTTTGAAG
GTTCGACAGCACGTGCAGCGATGTTTCCGGTGCGGGGCTCAAGAAAAATCCCATCCCCGGATCGAGGATGAGCCGGTCGGC
AGCGACCCCGCTCCGTCGCAAGGCGGAAACCCGCGCCTCGAAGAACCGCACAATCTCGTCGAGCGCGTCTTCGGGTCGAAG
GTGACCGGTGCGGGTGGCGATGCCATCCCGCTGCGCTGAGTGCATAACCACCAGCCTGCAGTCCGCCTCAGCAATATCGGGA
TAGAGCGCAGGGTCAGGAAATCCTTGGATATCGTTCAGGTAGCCCACGCCGCGCTTGAGCGCATAGCGCTGGGTTTCCGGTT
GGAAGCTGTCGATTGAAACACGGTGCATCTGATCGGACAGGGCGTCTAAGAGCGGCGCAATACGTCTGATCTCATCGGCCGG
CGATACAGGCCTCGCGTCCGGATGGCTGGCGGCCGGTCCGACATCCACGACGTCTGATCCGACTCGCAGCATTTCGATCGCC
GCGGTGACAGCGCCGGCGGGGTCTAGCCGCCGGCTCTCATCGAAGAAGGAGTCCTCGGTGAGATTCAGAATGCCGAACACC
GTCACCATGGCGTCGGCCTCCGCAGCGACTTCCACGATGGGGATCGGGCGAGCAAAAAGGCAGCAATTATGAGCCCCATAC
CTACAAAGCCCCACGCATCAAGCTTTTGCCCATGAAGCAACCAGGCAATGGCTGTAATTATGACGACGCCGAGTCCCGACCA
GACTGCATAAGCAACACCGACAGGGATGGATTTCAGAACCAGACGCAGCCGCAGGCGCCGGAAACTAAACCGATGTCTGTA
GGGTTACCGCTGGATCTGCGTGAATTCCAGCAGCAGCAAGAGAAAGACTTTCTACAAACCAGCTTACAGCAGGCAAAATTTA
ACCAGAAAAAAGCCGCTGAATTACTGGGTCTGACTTACCATCAGCTTCGCGCTTTGCTGAAAAAACACCAGATTTAACGCAC
ATTTGCAGATGTTATATTGGCGGATTTGACGCATAACCTCATCAGGGTTTACCATGACGCCATTACTGTATAAAAAAACAGGCA
CAAAATATGGCTCTGGCACTCGTTGGCGAAAAAATTAACAGAAACCGCTTCACCGGTGAGAAAATTGAAAATAGTACATTTTT
TAACTGTGATTTTTCAGGTGCCGACCTGAGCGGCACTGAATTTATCGGCTGTCAGTTCTATGATCGTGAAAGCCAGAAAGGGT
GCAATTTTAGTCGTGCGATGCTGAAAGATGCCATTTTTAAAAGCTGTGATTTATCCATGGCGGATTTTCGCAATGCCAGTGCGC
TTGGCATTGAAATTCGCCACTGTCGTGCGCAAGGCGCAGATTTTCGCGGCGCAAGTTTTATGAATATGATCACTACTCGCACCT
GGTTTTGCAGTGCATATATCACTAACACAAATCTAAGCTACGCCAATTTTTCGAAAGTCGTGTTGGAAAAGTGTGAGCTGTGG
GAAAACCGTTGGATGGGGGCCCAGGTACTGGGCGCGACGTTCAGTGGTTCAGATCTCTCCGGCGGCGAGTTTTCGACTTTC
GACTGGCGAGCAGCAAACTTCACACATTGCGATCTGACCAATTCGGAGTTGGGTGACTTAGATATTCGGCGCGTTGATTTACA
AGGCGTTAAGTTGGACAACTACCAGGCATCGTTGCTCATGGAACGTCTTGGCATCGCGATTATTGGCTAGTCTTCAGGGAGC
GGTGAATATTCCGCCCCCTGCACAGCTTTTTACCCTTCAGGCATCGCTGAAGAGTGGTGTGTGGAAATTTTCCACTCTTTACCG
TCCCATGCATAAGTGAATGTGTAGCGGGCTGAAACCGTTGATTTGTCCGCGAAGGTAAATGTGTAGGTACCGGTGTCTATCGC
CTTATTACACCCCAGGCGAATGGTACGCATGTCAATTTTCCCGGTCGGCTTTTTCGCAAGAAAATGCTCGAAATAAGGCACTG
TTGCAAATAGTCGGTGGTGATAAACTTATCATCCCCTTTTGCTGATGGAGCTGCACATGAACCCATTCAAAGGCCGGCATTTTC
AGCGTGACATCATTCTGTGGGCCGTACGCTGGTACTGCAAATACGGCATCAGTTACCGTGAGCTGCAGGAGATGCTGGCTGA
ACGCGGAGTGAATGTCGATCACTCCACGATTTACCGCTGGGTTCAGCGTTATGCGCCTGAAATGGAAAAACGGCTGCGCTGG
TACTGGCGTAACCCTTCCGATCTTTGCCCGTGGCACATGGATGAAACCTACGTGAAGGTCAATGGCCGCTGGGCGTATCTGTA
CCGGGCCGTCGACAGCCGGGGCCGCACTGTCGATTTTTATCTCTCCTCCCGTCGTAACAGCAAAGCTGCATACCGGTTTCTGG
GTAAAATCCTCAACAACGTGAAGAAGTGGCAGATCCCGCGATTCATCAACACGGATAAAGCGCCCGCCTATGGTCGCGCGCT
TGCTCTGCTCAAACGCGAAGGCCGGTGCCCGTCTGACGTTGAACACCGACAGATTAAGTACCGGAACAACGTGATTGAATGC
GATCATGGCAAACTGAAACGGATAATCGGCGCCACGCTGGGATTTAAATCCATGAAGACGGCTTACGCCACCATCAAAGGTAT
TGAGGTGATGCGTGCACTACGCAAAGGCCAGGCCTCAGCATTTTATTATGGTGATCCCCTGGGCGAAATGCGCCTGGTAAGC
AGAGTTTTTGAAATGTAAGGCCTTTGAATAAGACAAAAGGCTGCCTCATCGCTAACTTTGCAACAGTGCCGTACCGACGGTG
ATATGGGGCAAATGGTGGTCACCATCCTGTCGGCTGTGGCACAGGCTGAACGCCGGAGGATCCTAGAACGCACGAATGAGG
GCCGACAGGAAGCAAAGCTGAAAGGAATCAAATTTGGCCGCAGGCGTACCGTGGACAGGAACGTCGTGCTGACGCTTCATC
AGAAGGGCACTGGTGCAACGGAAATTGCTCATCAGCTCAGTATTGCCCGCTCCACGGTTTATAAAATTCTTGAAGACGAAAG
GGCCTCGTGATACGCCTATTTTTATAGGTTAATGTCATGATAATAATGGTTTCTTAGACGTCAGGTGGCACTTTTCGGGGAAATG
TGCGCGGAACCCCTATTTGTTTATTTTTCTAAATACATTCAAATATGTATCCGCTCATGAGACAATAACCCTGGTAAATGCTTCAA
TAATATTGAAAAAGGAAGAGTATGAGTATTCAACATTTCCGTGTCGCCCTTATTCCCTTTTTTGCGGCATTTTGCCTTCCTGTTT
TTGCTCACCCAGAAACGCTGGTGAAAGTAAAAGATGCTGAAGATCAGTTGGGTGCACGAGTGGGTTACATCGAACTGGATCT
CAACAGCGGTAAGATCCTTGAGAGTTTTCGCCCCGAAGAACGTTTTCCAATGATGAGCACTTTTAAAGTTCTGCTATGTGGTG
CGGTATTATCCCGTGTTGACGCCGGGCAAGAGCAACTCGGTCGCCGCATACACTATTCTCAGAATGACTTGGTTGAGTACTCA
CCAGTCACAGAAAAGCATCTTACGGATGGCATGACAGTAAGAGAATTATGCAGTGCTGCCATAACCATGAGTGATAACACTGC

TGCCAACTTACTTCTGACAACGATCGGAGGACCGAAGGAGCTAACCGCTTTTTTGCACAACATGGGGGATCATGTAACTCGC
CTTGATCGTTGGGAACCGGAGCTGAATGAAGCCATACCAAACGACGAGCGTGACACCACGATGCCTGCAGCAATGGCAACA
ACGTTGCGCAAACTATTAACTGGCGAACTACTTACTCTAGCTTCCCGGCAACAATTAATAGACTGGATGGAGGCGGATAAAGT
TGCAGGACCACTTCTGCGCTCGGCCCTTCCGGCTGGCTGGTTTATTGCTGATAAATCTGGAGCCGGTGAGCGTGGGTCTCGC
GGTATCATTGCAGCACTGGGGCCAGATGGTAAGCCCTCCCGTATCGTAGTTATCTACACGACGGGGAGTCAGGCAACTATGGA
TGAACGAAATAGACAGATCGCTGAGATAGGTGCCTCACTGATTAAGCATTGGTAACTGTCAGACCAAGTTTACTCATATATACT
TTAGATTGATTTAAAACTTCATTTTTAATTTAAAAGGATCTAGGTGAAGATCCTTTTTGATAATCTCATGACCAAAATCCCTTAAC
GTGAGTTTTCGTTCCACTGAGCGTCAGACCCCGTGTTGACACCGGCGTACCCTCGGTGCTATCTTCGCGCCCCAATAGTCGGG
GCTTGGCCAGGACTTCCTGAGGCCGTCCGTAACTCCACCTTCAAACAAGGAATATCGTTGATGTCACTGTATCGCCGTCTAGTT
CTGCTGTCTTGTCTCTCATGGCCGCTGGCTGGCTTTTCTGCCACCGCGCTGACCAACCTCGTCGCGGAACCATTCGCTAAACTC
GAACAGGACTTTGGCGGCTCCATCGGTGTGTACGCGATGGATACCGGCTCAGGCGCAACTGTAAGTTACCGCGCTGAGGAG
CGCTTCCCACTGTGCAGCTCATTCAAGGGCTTTCTTGCTGCCGCTGTGCTGGCTCGCAGCCAGCAGCAGGCCGGCTTGCTGG
ACACACCCATCCGTTACGGCAAAAATGCGCTGGTTCCGTGGTCACCCATCTCGGAAAAATATCTGACAACAGGCATGACGGTG
GCGGAGCTGTCCGCGGCCGCCGTGCAATACAGTGATAACGCCGCCGCCAATTTGTTGCTGAAGGAGTTGGGCGGCCCGGCC
GGGCTGACGGCCTTCATGCGCTCTATCGGCGATACCACGTTCCGTCTGGACCGCTGGGAGCTGGAGCTGAACTCCGCCATCC
CAGGCGATGCGCGCGATACCTCATCGCCGCGCGCCGTGACGGAAAGCTTACAAAAACTGACACTGGGCTCTGCACTGGCTG
CGCCGCAGCGGCAGCAGTTTGTTGATTGGCTAAAGGGAAACACAACCGGCAACCACCGCATCCGCGCGGCGGTGCCGGCA
GACTGGGCAGTCGGAGACAAAACCGGAACCTGCGGAGTGTATGGCACGGCAAATGACTATGCCGTCGTCTGGCCCACTGGG
CGCGCACCTATTGTGTTGGCCGTCTACACCCGGGCGCCTAACAAGGATGACAAGCACAGCGAGGCCGTCATCGCCGCTGCGG
CTAGACTCGCGCTCGAGGGATTGGGCGTCAACGGGCAGTAAGGCTCTGAAAATCATCTATTGGCCCACCACCGCCGCCCTTG
CGGGCGGCATGGATTACCAACCACTGTCACATTTAGGCTAGGAGTCTGCGCGGCAGAGCCGTGTGACCGGTTTTCTGTAGAG
CACTGACGATGGCGGCGGCGCTCTCTGCAATTGGCAAGGCGTCGGCGCCAAGGATACCAATCTTGCGGCGCGCGGCGTGTT
ATGACGACTGGGGTGCATTTGAGCCGCCCCATTTAACCTTCGCCCTCACAGATACGCCATTCGCCTCAGATTTAGCGCCATGCA
GACGAGCTTCCACTCGGCTTGCACCTTGTCCAGGCCCCTCATGCTGAACTGACGCAATCCCATCACCGCCTTGATCCAACCATT
CGGAGCCTCCACGATCGACTTGCGCCGGCGGTAAGCTGCATCGCCTTGCTCCGTTTTCAATTTCGCCGCAATCGCCGCCGTAT
GCGGATGGGTCTTGGCATTGACCTTGGCATCTTCACGTCCCTCGCGGCCGAGGGCAACGATGACATCGCCGTGGTGATCGGC
GACCTTTGCCAGAACAGCCTCACTACGGAATCCCGCATCCGCCAGCGTCTGGGCCGGCATTTCTCCGGTGTTGGCCTGAACT
GCTGCCAGCATGCCCAGCAGCGCCTGACTGTCCGCGGCGCAGTTGGTCAACTCCGCCGCCACGATGATCTGGTGCTCGGCAT
CGACCGCTGTGTACCCGTTGTAGCTCTGCTCGGAGCCACCACCGGCGTGTTTCATGATCCGGCTGTCCGGATCGGTGAAGCTT
TCCTGATCACGGTCATCCGGCACACCAAACTCGCGTTTGTACGAGCCACCGCCCTTGTCCGAGCCATCCGGATGGCGAGGCC
GGCGGCCATCGTCTTCGCTGCGCCCCCGGGCCTGGTCCGCTTCACGCTGGCGCGCTTCCAGGGGTCGGTTCCGGCTGAGGG
CGAAATGACACCCTAAGCGTTAGCTCTGTGTCGTTGCACGATGTCAGCGACGGTATTCTTGCTGATACCGAGCTCGCGTGCGA
TCCAGCGATAGCTGCGTCCCTCGGCCCTCATCGCAACCACCTTAGGCAAAAGTCGGTCTGATTTTGGTCGCACTCCGGCCTGA
CAACCAAGCCTCTTACCACGTGCCTTCGCAACAGCAAGGCCTGACTTGACCCGCTCGCTGATGAGATCCCGCTCAAACTCCGC
AATGCCGGAAAGAAACGTCGCCAGCATTCGTCCATACGGCGACGAAAGATCGAACGCCATTCCATTCATGGCTATCACGGAA
ACCTTCCAGTTCTCCAGTTCACGTAGCGTATTGAGCAGATCGAGCGTCGAGCGCCCCCACCGGGAAAGCTCAGTGACCAGGA
TTGCATCAATTTGTCTGGACTGGGCAAGCGCCAGGACTTTCTTTCGCTCGGCCCGGTCGAGTTTAGTTCCTGAACCTGTTTCC
TTAAATATTCCCACCACGTCGTAGCCGGCACGGCCGGCGAAGGCTCGCAGATCAAATTCCTGGCGTTCACAAGACTGATCCGC
TGTTGAAACCCGGCAGTAAATGGCGGCACGATGTCCCAATTGAACCCTCCTGGATTTTTGTATCGGAACGCCCTGATTTATATG
GGCTGGCTGTTGTCCAAAACAGACTATACTTCAAAAGGGACGAATTTGTATGTCACGACGCCATATTTTCACCGAACGGCAGC
GAGCAGCGCTGTTCGATCTGCCCACGGACGAACTGTCGCTACTGAAGTTCTACACGCTGGGCGATGATGACCTGGAAAACAT
TAGGCAGCGCCGCAGACCGGAAAACAGGATTGGCTTTGCCCTGCAACTTTGTGCCTTACGATATCCGGGCCGTGCACTGGCT
CCTGGTGAGATGATCCCGCGTGAAGTCCTTTCCTTCGTCGGTGCTCAGCTTGGAGTTCCGGCTGATGCGCTTCTCACTTATGCC
ACACGGCGCCAAACCCGTCAGCAGCACATGGACACGCTGCGCGAAATTTACGGCTACAAGACCTTCACGGGCCGTGGTGCC
CGTGATCTGCGGGAGTGGACTTTCGGCCAGGCCGAAGATGCCAGATCAAACGAGGATCTTGCTCATCGTTTTATTGTGCGGT
GTCGGGAAACTTCCACCATTCTGCCCGCAGTATCGACAATCGAGCGCTTGTGCGCGGATGCTCTGGTCGCCGCTGAGCGGCG

GATTGAAACGCGGATTGTGGAAAATTTAACAGCGGATGTTCGCGATCACCTGGACAAACTTCTGAGTGAAATGCTCGCCGGC
AATATCAGTCGTTTCATCTGGCTTCGCAACTTCGAGGTTGGTAACAACTCGGCTGCTGCTAACCGTTTGCTCGACAGGCTCGA
ATTTCTGCGTACCCTGAATATCAATCATAGTGCTTTGGCCAGCATACCTGCCCATCGCATTGCCCGGCTGCGTCGGCAGGGTGA
ACGCTACTTCACCGACGGTTTGCGTGACATCACTTCGGACCGCCGCTGGGCGATCCTTGCCGTCTGTGTTGTGGAGTGGGAA
GCGGCGATTGCTGATGCCATAGTCGAAACCCATGACAGGATCGTAGGAAAAACCTGGCGGGAAGCGAAGCGCCAGCATGAC
GAAACAATTTCCGGCTCTAAAGCCACACTCACGGATACGATCCGTACCTTCACCGCGCTGGGAGCTTCGTTGCTTGAGGCCCG
CAGTGACGGAACCCCGCTGGAGATGGCTGTCGCCAGTTCGGTTGCATGGGACCGGCTCGCTCAACTGGTAGCGACAGGGAC
TCAACTCAGCAACACGCTAGCCGATGAGCCTCTTGCATATGTCGGGCAGGGATACCATCGCTTTCGTCGTTATGCGCCCCGCAT
GTTGCGCTGTCTGAAGCTCGAAGCCGCGCCGGTCGCCGGACCATTGGTAGCAGCAGCTTTGTCGATCGGAGAGATGAAAGG
TGTTGCATCGCCAGAAAGGCGTTTCCTGCGGCCCAGCTCCAAATGGAACCGTCATTTACGAGCTCAGGAAAAAGGAGATACC
CGTCTTTGGGAAGTGGCGGTACTCTTTCACCTCCGGGATGCTTTTCGTTCCGGAGATGTCTGGCTCGCTCATTCGCGCCGCTA
TGGTGACCTCAAGCAGGTACTGGTGCCGATGATCGCGGCGCAGGAAAATGCAAAACTGGCCGTGCCTTCCAACCCACAGGA
TTGGCTGGCAGACAGAAAGGCGCGACTCACGATCGCTCTTAAGCGGCTGGCCCGGGCTGCCCGTAACGGCACTATTCCGCA
CGGTAGCATAGAAGATGGAACGTTGCGGATCGACAGGTTGACAGCAGACGTGCCGGATGGTGCCGAGGCACTCATACTGGA
TCTGTATCGCCGAATGCCGTCCGTTCGGATTACCGACATGCTGCTTGAAGTTGATGCAGCCCTTGGTTTCACAGATGCGTTTAC
CCATCTGAGAACCGGGGCTCCATGTCGCGACCGGATCGGTCTGCTCAACGTCCTGCTCGCTGAAGGGCTCAATCTGGGCCTG
CGTAAGATGGCGGAAGCTACAAACACGCATGATTACTGGCAGCTCTCACGCCTTGCCCGCTGGCATGTTGAAAGCGAAGCCA
TGAACCAGGCATTGGCAATTGTGGTGGCCGCGCAGGGTAAACTGCCGATGTCACGCGTCTGGGGGATGGGCACGTCAGCAT
CGAGCGATGGTCAGTTTTTCCCGACAGCGCGGCATGGCGAAGCCATGAACATGGTCAATGCCAAATATGGTTCTGTTCCCGG
CCTCAAAGCGTATACTCACGTAAGCGACCAGTTCGCGCCATTCGCTTGTCAGTCGATCCCGGCGACCGTGAGCGAGGCACCG
TATATTCTCGATGGACTACTGATGAACGAGGTCGGTCGCCATGTTCGCGAACAGTATGCCGATACAGCAGGATTCACCGACCAT
TTGTTCGGAGCCAGTAGCCTGCTCGGCTACAATCTCGTTCTGCGAATCAGGGATCTGCCATCGAAGCGGTTGTACGTATTTAAT
CCCGATACGACCCCCAGGGAGTTACGCAAGTTGGTAGGTGGAAAAGCCCGGGAGGATCTTATCGTTGCGAACTGGCCTGATA
TTTTCCGTTGTGCCGCGACGATGACCGCTGGCAAAATCAGGCCCAGCCAACTCCTGCGCAAGCTCGCTTCTTACCCACGACA
AAACAACCTTGCAGTTGCGCTTCGTGAAGTTGGTCGTATTGAACGGACCCTTTTCATTATTGAGTGGATCCTGGATACGGACA
TGCAGCGGCGTGCTCAGATCGGTCTTAACAAGGGAGAGGCCCACCATGCGCTCAAAAATGCGCTCCGTATCGGGAGGCAGG
GGGAAATTCGCGATCGCACGACAGAGGGGCAGCACTACCGAATCGCTGGGCTCAATTTATTGACTGCGGTGATCATTTACTG
GAATACCGTCCATCTTGGTCATGCCGTCACGGAGCGGCGGAACGAAGGGTTGGATGTTCCCCCTGAATTTCTTCCCCACATAT
CCCCATTGGGCTGGGCGCACATTCTACTGACTGGCGAATATCTTTGGCCCAAGGAACCGAAAGCTTAGGGTGTCATTTCGCCC
TCAGCCGGAACCGACCCCTTCCAGGCGCGCCTTTGCCGCCTGGATCGCCTCCAGGCGCTTCTCGCGGCGAGAAATCTCGGCA
GGAATGTCCAGCTCCGGCTCGTTACGCTCCTGGTCGTCGGTAGCCTTGGCGCGATCAAGCAGCGCCTTGATCTCGCAATGCA
ATTCGTCCTCGGCCGGCTTCATGCGCTTATAGCTCATCGCCTTGTGGCGGCTGGCGTTGGCTTTTACCTTGGTGCCGTCGACCG
CGATCGTGCCAAGCTTCACCAGCCCACATTCGCGCGCCAGTTGCACCACCTGAACGAACAAATTCTCAAGCTCGGTCAGGTG
TAGGGCACGGAAGTCACTCAGCGTGCGGTGGGCCGGGAAGTTTCCAGCGGCCAGCACACGCAACGCGACATCCTCGTACA
GCTTCCTGGCTAGTTTGCGCGAAGAGAAGACGCCGCTCGCGTAACCATAGATCAGCACTTTGACCATCATCGCCGGATGAAA
CGGCTGATTGCCCGGACCACCGCCGGCATACCGGGCGTGGAATGCGCTCAAGTCCAGCGTATCGACAGTCTCGCTGATGAAG
TAGGCAAGATGCCCTTCAGGTAGCCACTCTCCCAGAGAAGGGGGCAGCAGATAGGATTGGTCGGGTCGGTAAGGAAGGTA
ACTGGCAGCCATCCCCGTATCGTCCTCGATCTTCCGCCGATTTGGCTTCTGCCGCGCAGGCTCCTAGGCCACCGTAGCTAAACA
TACGAGAAAAACCGCTTCTTGAGCGCTCTCTGAGACCACCTTGCCGACAACGCAGAAACGGTTGCGGAAAAATCGGCGTTT
CCGGCGTACGCCACCCGACCTGGACGAACTATTGCGGACCTGGAACTGGAGTAGCGGAATTCCCCGGGGTTTCAGCGTCGC
GCCACAGCGCCTCAGATAGATGCGGTAGCCTTTGGCGGGTCGTCATGCCGCGGACCACCCCCCAAAAAATTCCACAATGCGG
AATGGCGGAATTTTCTGGGGTTCCCGCTTACCCCCCCTTCAGGCGAGTAGCCAACCTGAACGGATGGTTCCACCGCAAAGGC
GGGCAGGGCGGCTGTCGTAAATGCTGCGGCCAGAAGCCAGGTTGCTAAGTTTTTCATTACAGGCTCCCGTTCACACACTGCA
TTTTATATTCAGGGTCAAAATAAATTTCTTTGATTTTCCGCTTTTCCATATGCACCACAACGTCAATGGTGGAGTAGAGCATTCG
CATAATATCGCTCATATCGAGCATACGGCCGATAGGGGTCGCCTTGATAAGCAGCCCAATACGGTTAAAGGCATCGCGCGCAG
AGTTAGCGTGCGTTGACATAACACCGCCTGGATGGCCGGTATTAAGTGCTTTAAGATAATCCCACGCAGCATCATCCCTAAGCT

CAGTCATGATGATACGGCCCGGTGTCAGACGCATACAGGCTCGCAGGGCATCAGTGGCGCTGACGCGGCCGATCTTTCCTGC
ATCGCCGTACATCATATAAACGGCTTCTACAACGTGATCGACCGTGACTTCGTGAACGTCCTCTAAAATAATTACACGCTCGTCT
TTATGTAGCGATTTAACAGCGCGCGCGTGAGTACCGTTTTCCCCGACCCGGTTTCACCGCAGATCACGATAGTGCGTTTCTTC
TCAACGGCGGTTTGCAGGAATGCGGGCCATTTTTCGCTGCTGTGCAGCTCTTTAAGGAAAAAATCATCATCCGTTAGGCTTTG
CTTGCTGCCGGTAATCTTCCGGCAGTCACTGAAAATCCCCTCGCGGGTCAGCTGCTCCAGATTTTTATCGGCCGCCAAATCCTT
ACGAAACGCTACGGCCGTTGTACCGTCAATCACCGCAGGGGCAGACAGATAACGCCCCTGATCCCGCCAGGCAGGATCACGT
CATTAATGGCCTGCATGGTCAGCTTGTTGCTGCTCACCAACGATTTAGCAAGGTTCCTAATAAAATCTGCCGTAATTGCCGCGT
TCTGCACAACCCTGCGGCCGCTGAACGTATCACAGATAACTTCCTGAAAGCAGTTAATGCGAATTTCAAAAACAGTAGGATCT
TCTAAATACTCGCGCAGTGGGCCAAGTTGATAGAAAGCTGCATCAGTCATGATTACTCCTGAAGAAAAGCGGGCGCTAAGCG
CCCACTTTTTTAGTTGTCTGCGAGCGTATAAACGCCGCTGAAATCGAGGTCGCGGGCAACAAAAATGCTCACCGCATCACCCT
GCTGATCGTAGAGGGTAGGGGGGATAGACATGTAAGAGCGGAGTGCTTCAGACGCCAGCTGCTCACCGCTGTTTTCTGTGCT
GTTGTACTGAATGTTATTACTCTGCGTCTGGTTAACCAGCGCCGTTAAGGTGTCAGAGAACAACGAAATCATGATCGCACCAC
GCAGACGCTCCCACATATGGGTATCCACCTGGCCCGGAATCCCCGCGCTGCCGAGTGAGTTCGTTCCGGCACTGTCAATATTA
ACGATTGTCCCGTCCTGGTCATTGCGGATACGCTCCCAGAGAACAAACACGCGCGCCTGGCCGTCTTTGATACCACCGGTAAT
CTGCCCGTCAACCCATGAGCCTTTATCAATCAGCCTAACGAGTCCATCAGCTGAGTAAACGTCCTGTGAAACCCGGCAGGAAA
CCTGACCCGGAACAGTGGTATCCAGCTCGGTGCCGGTACCACAGGGGATCATTTTGCCTTTCGGAACAGTCAGGCTGGGATT
AGCCATGACTCCAGCGCGGCTAGCCTTCAGCCTTGCAGGAGTCAGGTTTTTAGCGAGTGCTGAACTACCTTCGCTTGTTTCGT
TGTCCTGGGGCTGCACTCCGGGACTATTGCTTGTAGCCGCCTGATTAGTCTGGGCCAGCTCGCCGCCCAGACGACGCTGCATA
GCCAGTTCTTCAGGCGAAGGTTCTTTACGCTTATTAGAGGTACGCGCGGCGGTATTGCTGCTGCCCGCATCTGCATCTGCCTG
TGCGGCAGCCTGCACAGCACGGGCATCAGTGGCGCTATTCTGCGCAGTTGCAGGTTTATTAACATCAGGATCGCTGTTAAAG
CTGTAGTTTGGCAGTGTATTGGCCTGTTGCGCTTTACCACCGTCTTTATCAGCTTCAGCTTTAGCCGGGGTGCGAATTTTACCC
ATGACCGTAATCCCGATGAATACCAAAGCAAGCAGCGCCATCAGTATGACAAAGGCTTTCATACCAGGAGCCGAACGGCGGT
TACTGCCTTTAAATCCGCCACGCTCGCTTTCGAATTCACCGTCTCCGGTGTTTTCATCGAGTTCCTGATCTACATCGACACTTTT
ACGGGCCATCAGTTATCCTCCCCAATTTGAACCCTGCGCACATCCGGGGAAGCCGTACCGGTTGCTACCGCACCGGCGCCCG
GCGCGAAATTATTATTACGAACGCCAACGACTTTATCGCCCAGACGAATACGCCACTCTTTAGCGACGGTTTCCACCTCGATGA
TGTTGCGGTTCTCACCCACAACATGAGAGTTAGGCAGCGTTTCTTTGCCACTGGCCGAAATCATGTAGACCTGCGGTAACTCC
GCATTGGCCGGAAACTCAAACCGGGTAAAGCGGTAGTTATCCCAGACGTGAACCGGCTGGATGCTGCGCATTTCAGGCTGTT
CGCTCATTACGTACTGATAGTTCTTCGCCCCCGCAAAAGCCGTCTGCTTCAGCTTCTGCGTAATGCGTTTTTTATCAGCCGCGCT
TTTGGCTTTTTCCTGCTGCTCAAACGGATATTCATAGGTCAGCTGAAGAACGGCCTGGCGCACAGCCCACGGCGTTTCAATAA
AGGATTTTGATACCGTACCGTCTGCATTTTTCTTCGTTTCTTCACCGATGAAATGGAGGACGATGTTATAGGTGCGCTTATCGGT
GACGATCACCAGGTTGGTATCACTCATGGCCTGTTTCGGCTTCACAAAAAAATGGTTCATTTTGTGCGCAAACGTCCAGCTTT
CAGAATCGCCAAAAGCATGAGTGATATAGGTTTCGTCAGGCGCGACAACAATGTGGGTAGCCACACCGGCGATAGCGTCAAT
TTTGACCACATTAACAGGGTTATAAACAACGCTTTTAATGCGATAGTCATAAGGAGAATTGCGGCCAACCTCAAGCGCCATAA
CGTTAGTGGCCGCGCCTCCCAGGACTGACAAAACAACTGCTGAAAGAAGTAGTTTTTTCATGGGGCAGCCCTCAGTTAACTT
CAGGGTTGACGCGATAACTCGTCACGCGGAAACCCAGCGGGTTGACATAACGCTGCTCAGCATTCATCGCCAGCGATTTATAT
TCATACCCCATAATGGCAATCCAGCGCTGCGGCTGATCATCAACGGGATTGCTGCGCACGCGGCGAACCGTAGTAAAGCGTAT
CGTTGCTACGCCGTGCGGTTTATCGAGGATCACAGAGTTAATCTTCACGCGGGTCGTTTCACTGTCGCCCAGAACCTTATCAA
GACCGTTGCGGCCCTTGAACTTGCTCTGGTAAGACTCTGCCACGTTCGGCGTGGACATTAAGCCAACGGCCGTATAGTCGAC
CTGAACTGAATAGAAGTCATAGCTCTCACGGTGAATGACATATTGTGTCAGCCAGAACTTATCAATTTCGTCACCATAAGAGGT
CTGGTCGCGGGTCAGCTTGACCTGCTGTACTTCGTGAGTGGCCTCGTTGAGCGTTAGCAGATGTGCGGGGATTGGCTGGCTG
TACTTATGCACCACGTAACCAACTAAAGAGAGTGCAAAAACAGTTACCACCGCTGAACCGGTGGCAACCATCCAGGCGGTAC
GCCGGGACTTCAGCACTTCATCCATCAGATCAACTTCAAGCCCTTTACGGCTTTCGTTGAACTCTTTAATGGCTTCACGTGTAA
GCCCTGTTTTTTTATTAGCTTTCATTTGCACCACCTTGCGTATCAACCGGGATTGTTTTGTTTACTGGAACGGTGTTGCTCCAGT
CCGGCTCCGGTGGCGGTTTATGCCCGCTGGAACAGGCGCTAATCAGAAGAACTCCCATAAGCAATAAGCTGCGCATTACGGC
ATCCATTATCGGTTGTTGAACGTACTAATTATAATAAGTCGTCGAAATTGCAATTTCAACGACTTTTGTTTTAATTTTATGCAGCC
TTCTTCCCGCGACTGCCCCTTGCCTTACCACTTAAATTAGAACCACTGCTTCCTCCGCTGTTGCTGCCACTTCCTCCACCTGCCG

ATTGCTGGCCGCCGCCACTGTTACCGCCTCTGAACGCATTACCGCCAAACATGCCGTGGCTGCCCATATTGCCAAGCGCCTGC
ATGGAAGAACCCGTAGAGCGTGCGGCGTCAGCAACTCCGGCGCTGATACCGCTACCCCAGCTGGCGGCAATTTGCGGAATCT
GGAACAGAACGAAAACGGAAATGACCGTCAGTAATAAGGCGGAGATAGAACCAGTGATTGATGAGTAAGCGGCATCAGAGT
TCATCGAGGACAGGAGGTTGTCGAACATCTGCATGATGAAACCAAACACGAGCGCAAGAATGACGACGACAAGGCCATAGT
TAATGACCGACGCCAGCCAGCGAGCAAAGATGTTTTTTGTCGCTCCCCACAGCAGGCAGAAGATTGCAATCGGGCCAAAAC
AAAGCGTAACGGCCAGAAGGATCTTAGCCATGATCACAAAGCCCGCACCGAGGCCGCCCAGCACAACGGTAGCAATCATCAT
AATGCCGCCAATGGCATACGCGGCCAGGCCGCTCGATGAAAACACGTCTGCGGCTTCCCATGCGGTGTTGACGATCTTGATAC
CTTTTTCAATACCGCTATCAATAATCGCCGGTACGCCACTTGCACCGACTTTATTAGGGGCAGACAGTATCCCGGCAAAATCAT
CCGGCAGGTGAAGCGCCACGTTGACCAGTTCCTGTTGATACCAGCCGCCCGCCGTTGCAAAGCTCAGGATAAGGGCTATGG
AAAGATACTCTTTAATCAGCGAACTCAGGCTGTCGCCCGCCCCCGGATTAAACGCTGAGTACATCCCCTGAACCATCAGCTTG
ATTGTCAGACAGGTGGCAATCAGAGGCGTTACATCAGAGATAATGGTGGCAACATTGGCGCTCACCATTGACGTAATCGCCCC
GTCTACTTTTGCGAAAATGTCTGCGACTAGGGTGAATGCCATATTGCCTCCTTACTCCTAAATTTTCGGTGAATTGATTTCGCCG
TCTTTTTTGCCGAACACAAACAGCTGACGATCGGCTTCGCGCGCGTTTTTGCAATCAGGTTTCTGAAGTTCATCCGCGTCTTTC
TTGCATTCCTGAATTGTTGCCTTGCGCTCTTTCTCATGTTTTTTGTACCACTCCACGTCATGCGAGGCATCGCAGGCCACTAGG
AGGAAAGGGATAACAAGCAGTAGTTTTTTCATAATTACCTCAGTTAATTGAAGGTGACGCGGTAACGTCCCCGCCGGTTCCAA
AAACAAAATTGTGGGTGGCACGTTCTTTCTGCGCACGTAATAGCTTGTCCTGTGACTGCTGCAACATGTTCATCAGATTCAGCT
TCGCCTGCTCACCCTGAATAGCACCCTGTGACGTCTGGATACGGGCCTGTAAGTCAGCAATCGATTTCAGGTCTGGAGTCGTT
TTAATCTGCTCCGTCAGCTCCTGCATATCGGTTAGTTCCTGCATCTGGTTGTTGTAGGCTTTTTCTGCCATAACACGGTCATAAG
CGCCTTTTTCAGCCAGCTTTTTGTTCATGTAGGTAATTGCTTCGCTGGGCGTCATGTCGTCAACTTCCGCATTAAACTGGCCCA
TCATGCTGTTAACTGAAGGCGTGACGGAAGAACTGGAGTTCATGGCGTCGCTGTAGATCTCCTTCCAGTTGTCCGGCAAATTA
TTTGCCAGCGTGCTGGTAGACGTCCCCAGCAGATCGCCCAGGTTGGTTGTCTTTGCCATCGACTCATACATATTTTTCTGCGTT
TGCAGCTGGCTTTTAAGCTGCTCCAGCTGCTGCGCCATTTGCTGAAGCTGCTCGACCTGTTTAGCCAGCTCAGTAGGGTTGGT
CACGATGATGCCTGCGGAAGCGCTTTGCGCGCCTCCCAGTATCAGGCCGGTGGTCAGCAATACTGCCGTCAGTGTTTTTTTCA
TGGTGTTTTGCCTCGTTGTTAAGCCGTCAGGCGCCAGTATTCTTTGAGCCATACTTCAGGGTCATTACCGAGCCGTTCAACCA
GCTCATGCGCAATTTCGGCGTTTTGTGGTTCACCGGACAACACGCTAAGAACGTTGTCCATTGTCTTAATATCTGCATCAATATC
GTCGCTGTTGCGAGGGTAGAGATTGAAAGAAGCAATCGCAGACTGTTGCCCTTGTTTAACCAGGAACTGTCTGGAGTGTTCG
GTAATCGACATCAGCGCGTCGTATTCAGCATCAGTAAGGAAGGCATAATCCTCTCGGATAGCTTCCGGATCGCGCAGGCAGAT
TTTTGTGACGGTCTGCGACATGATCGTGCGGCCAATACGGCTGGACAGCGCATCGTTCGGCTCCTGCGTGGCAAACACATAG
ATAGCGTCTTTCTTACGGTCAGTTTTGATACCACGCTTAACTTCACGCTCGATAACCGGATCGTCAAGGTAGGCGTGGAACTCG
TCAAAGCACTGAATGACGCGGCGTTTGCCGTCGATGGAGTCACGTACCCGGTACAGAAGGTACATCATGAGCGGAGTACGG
GCCGGGCTGGATACTTCCTCTTTGGCTGCGATAAACTCGGATAAATCGAAGCCAAAAATATCATTCGCGCTGAGATCCAGGCT
GTCTTTATCATTGTCAAACAGCCAGCCATACTGGCCTTCGCGCGTCCATTCGCGCAGCAGCCCTTTCAGTGATACGCCATTTTC
CACAACTTCATTCACGTACCCGTCCAGGATAGTAACGGTGCGCGCCTCGCGTGGAATAAGTGAGCCTTCCCCCATAACCGCAT
CAACGCCTTCAGCCAGTTCGGTAGCCATCGTTGCGCTGATAGGCCCGTTATTGGTGGTTTCCACACAAATGCGGAACAGGTTT
TTAATGAGGGCAATATTGCGTTTGGTCGGTTCAATCTGAAGCGGGGCAAACCCGGACGGCATACCCTGTTGCAGAACTTTATA
GTAGCCACCAACGCTTCGAATGAACGGCTCCATACCGCGGTCACGGTCATAAACAAAAAGCCGCGGGTTGTACTTCATTGACT
GCGCCAGCAGGAAGTTAAGCAGCGTGGTTTTACCTTCCCCCGACATACCCGTTATTAACGCATGGCCCAGCGGGCGTTTACC
GTAGGAAAGTTCTTCAAGCGGGGTCACATGGAAATTAAAATAGAGTGGCGTACCGCTGATCGTGCGGAACATGGTCAGCGCT
GGCCCCCACGGGTTATTGTCAGGCTTGCCACGCATAAAATTGTGGAACGGGCTGAAGTGCAGGAAGTTCCATGAGTTTATCG
GAACCGGGCGCGGCGCCCATTTCTGGTTGCCAGGCAGTCTCGCATAATATGCAGCCTCAGAGGCCAGGCTGAGAGTCCCGC
CAACCACGCCACAGCCGGTTAGCATAACCTTCACACGACGCGCTTTGCGCTGTACCGCGTTTTGGTCATTATCCCAGACATGC
ACGGTTCCATGATGGTAGCCCATCACGAACTCTCTGGACGTCAGCATATCGAGCGCGGTACCAAGCTGTGCCAGCTGGCTTTG
CGCACGGTCGCGCGTTTCCTGCAAAGATTTTTCCTGATGCGTCAGAAACGTTTTAGCTGAAGATTCAGAGAGGCAAGAAAAA
CTCTGCGTCAGAAGGTATTCAAAATCGGCTTCTTTAAGCATGTTAAGCTGGCCTGGCTCTGTATCTTCTTCGTATTCACGAAATT
CAATGCCGGTGGTATAGAAGTTGTGATCTACCGTTCTGATCTGGACAACATCCCCCCACAGTGAGCTAACAGGGCGGTTGTCC
ATGATGTACTCACGGATACGATCACGGCAAACAGGCACGATGGCCCATTCCATATTTGCGAGGAAATAAAGGAACTCCAGCGC

TTTTGAATAAGCGTGAGCCTGCGAAGGTTCAGGCTCGTTGCGTTCGATAACAATGGCTTCGTCAAAAATGTCTGATTCATCGA
CTTCAGCAAGTTCTTCACGTTCTTTTTTATCAGGCGCAGGAATTTCAACACCGCGTTTGTCACGATAATAGATACCCAACTGCT
GAATGCCATACGGCTTCATTGCTTCCAGGATTTGTTCAGAAATATCTTCCAGACCTTCAAGCGCCTCATTCTGCATTCGCTGAAT
TTCGTCACGAGTCGGCTTTTCAAATTTCGCCAGAAACTTCTGTGTTTTATCCCCTACCTGTTTGTAAATAACGGTCAGATAAAG
GTCATTAATCAGCTGCTTGGAATCACCGTGCAGCTTACGGTTATATTGATCAACATAAGCAGGGAAAAAATGGTCATACTCACC
ATCCGGGTACTCTTTAGCCTCATGGTGATATTCATGCGTCCACAGCTCTACATGGTCTGTTCCGAAGCTCTTGACCAGCGTATTA
AGGTCTTTATGCCAGGTGACCAGTTCCCGATCTGATGCGCAGTCATGTGTGCGGCCATCCAGCTTGAAAAAAGCCATCAGATC
GCCGTTTTCCATCGAAATCACGTAATCATTGAGGTGATAAGAATAGGGCAAATACTTTTTATTTACTGATGGCTCCTTACGGTAG
GCATCAATTTTTTTTGGCTTCGTAGCGGTGGCAGCTCTCATTATTTTAAACCCTTACGTTTGTAGTCAACAGAGGAATAAGACG
ATCCTCCCCACTGCTTAAACGGAGAATCAAACCAATTACTGAATTTGGTTTTCAACCACAGGCCCATAATTCGAAACATGCGGT
CGTCATATTTTGTTAATGCAGCTGAAGGAATCCACAAAAAGACGAACACCAGGATCGCCCACATATGAATAATCATAAAAAGC
GAGGCAGAAATCATGAATATCATCATAGCTACATTACGTGGTACACCCAGCGCGCGAGGTAAGCGAGTCGCACCTTTGAAAA
GCGGTCTTTTCCCGTCAACGAACATCTTTGTAGTCCTCACTTTGGGGGACAAAAGTCCCCCCTTTTCGCGTTGTTCAGCTTCCT
ACGCCCGTCAGGGAAACCAGAAATGATGCAGAACCAATACCAATCAGCGAGATTACGATACGAGGAATAAAGCTGGCTGGG
ATTACGTGAAGCATCCACATAAAGCAACTAACCATGATCGCAATAGCACAACCAATTGGAATCCATGTGCTCAACCACGTCTGG
ATTGATGTAGCGGTTGATTCACCAGTATCGGTGCCAGCGGCCAGCGCAATTTGCGGCAGGGCAATGGACAAAACGCCCATAA
CTACCGCAGGGCCATACTTCTTAAACAACGTGGTCATACTTTCTCCTTACTCACTATCTTCTGTTGATTGCAGATTTAACCGGCA
TTCTTCAACGCCACACTTATCTTTCAAAAACTCTGCCAGTAGCCTTAAATCAGCCCAGGTTCGTAGTTCATGCTCTTTGTTAACC
CGAATGGAACATGCGATCTGGCCCAACTGTTCATCGTCTATCTGAAAAACGGGATACCACGTCTTTAATGAAACCCGTTTTATC
GTTACCTCCTTCACTTTTCCTGTTTCAAATAGCTCCAGGATCTGTGATTGCAATACAAGGTACAAACGTTCCATCCATAACCTCA
CTCCCCCTTCGCTGTTTCCTGTTTGGTTAAAAAGGCATCCGTATTATTTCGGCTGAAGGCATCGCCATCACCCGAACCAAAAAC
ATCTTGTTCACCGTCATACTGCGTGGCCGTACTTTTTGCCTGCTGAGGCTCAGTCGCGGTGCTGTCCTCACTGGTCTGGCCATC
AGGTAGCAGCGTAGGGATTTTCAAATCAGTTGATTGACGCGCCACGTTGATAACTTTCGTGACATACCCGTTAGAAATCCCGT
TTATGAGTGAGCCGGTGTTGTAGCAGGAAAGCGCGTGTCTCAGCGCAACCTGCCCGGCTGGATAGGATTTCAGGGCGCTATC
ATAACAGGCTTTAAGGATGGTCTGGCTCGCCCGCAGGTTGATGCAGGGCTTGAAAATATCGTCAACCGAAAGACCCAGGCCC
ACTAAATTATTTGAGTTAATTTGTGCAAGGCCCATATCAAAACTTTTATTATCCTTCAGCAGAACTTTCGAAACGCTGACGGCCT
CAGCTTCAGTACGTGGTTGCTGTTTTAACTGGGTACTACCACCGTTAATATTGATCCTGTACGGCCCATTTGAGGACTCATGGC
CGACGATGTACGCCATTGTTAAGGGTGAAACATCAGGCGCACATCTCTGCGCAAGCCTGGCAACTTCATCTGAGGCAGGCGC
AGCACTGGCACGGCCAGCACAAGCAAGGCAGGCCAGAGCGAGAACCAGAAGTTTTGGATGTTTACTCATTTACGTTTCACC
GCGTAATTGTAATTTCGACGACTTATGTTTATGATAAGGGCTAATGAGATCGTGTCAAGAAGAAACGAAAAGAAAACCTCTGA
AGGAAGGAAGGTAATGCTGGTTCCCCTGAAATCAGAAAAACGCCCCAAAGGTGAACCCATGTATCGTGACCCGGATAACCCT
TTTAATACGTGGACTGGTATAGGGAAGCGCCCGGCCTGGCTAACTGCAAAATTGGACGCTGGCATTAGCCTGGAAGCCATGA
AAATGCAGGGCGTTGCCAACCCCAGAGAACATAGACAAGTAAAATACCGCGACCCCAGGAACGCAGAAAATACCTGGTCCG
GGACTGGCCGCCGACCAACATGGCTCAAAGAGCTGCTTGATAGTGGTTTATCACTTGATGATCTAAAGATATAACCGGAGAGT
ATAAAAATGGGCGATGTTATTGATTTTGCGGAAAAGCAAAAAGGCAGAAGGAAGAAAAAGGCATTTTCCATTCCGCCCATTT
TAAGAAAATTCCGAGTCCATGCTATCAGGTTACTCGCCAGCATCATCAAATCCGGCTCTTATTCAGTTGCTTATATCGTTAAGAA
AATTACAGGAAAGTTAATTAAGTTGTTCGCAATGTTAACGATTTTCGTTTTTATTGTCGAATATATCGCAGGCGATATAGGTTAC
AAATCTATTTATAACTCTGCGTTATTATTAATATTACTTACCGTTATCAACATTCTGGCGGGTGTATATCTGAACAAACTGTTAAGG
ACAAAACAATGAAGAAACTCTTAATCCCTCTGATAGCAGCTGGTAGTCTGCTTTATCTTCCTGCCAGCCATGCTGAAGATCCCT
GCAAAGTTATTATGTGCATGGCGGGCAAGCTCACCGGCGATAGCGGCGGAAGCGAGTGTAACAGTGCTGAAGCTGCTTTCTT
CAATATCGTTAAAAAGAACAAGCACGGCTTTTTACCCAACCACACGAGGGATGCCAGGAAGGCTTTTCTTAATGAATGCCCG
GATAATGGCGAAGGTGGAAGTAACCAGTCGATGATAAGCCAGATCATAAGTAAATACGGGAAAGTTCGCTTATAGGCGGGCT
AGGAATAATCTCAATTTAAGGAGCCAACGTGAAAAAAATTATATTAAGTGCATTAGCCTGTACAGCTCTCTTAACTGGTTGCGT
CAGCCAAGATAAAGGTAATGCGATGCAGAGCCAGATGAATAACCAGCAACGCCAGATTAACGAATTATCCGTTCGTTTGCAGT
CTGCGGAGTCCCGGCTATCAAAGCAGGAAGAAAAGCTGCGCAACGAACTGCTGCAATCCAGCGGCTATTGCTATCTGAATGG
CGCCCGCTACTCGACCGGCACCGTACTTTACGGGCGGATTTGCCAAAATCAGTCAGGCAGCGCTTCGTGGCAGGTTTACAGC

CGTCGCTAAACACCAGGGCGGTTTAACCGCCCTTTTCTTTACCCACAGAAAGCACACTATCACCATGTTCCCCTTCCCCACTAC
AGAAAACCTTATTCTATGGGCTTGCAGCGCCATCGCACTGTTTGCCGTTGTATTCTTCCGGCGTTCAGTACGCAACCGACGAC
ACAAAAGGAAGCAGCAAAGTGCGCGGCGGGTGCTGGAGCGCATAAAGACGTTGCCGGGCTTCCCACAAAAAATTAACTAC
CTGAGGAAAATTGATCCTTTTGTGTTTGAAGAACTGTTGCTGGAAGGATTTGAAGCGCATGGCGGCTTTGTTGAATAAATCA
GATTTCGGGTAAGTCTCCCCGTAGCGGGTTGTGTTTTCAGGCAATACGCACGCTTTCAGGCATACCTGCTTTCGTCATTTTGTT
CAGCGCTCGTACCAGGGCCATAGCCTCTGCAACCTGACCATCGTAGTCACGCAGTGTCAGTGAACCTCCGAACAGCTGTTTTA
CCCGGTACATCGCCGTTTCCGCTATCGAGCGACGGTTATAATCTGTTGTCCATTTCCACCGCGCATTACTCCCGGTCAGCCGCT
GATTCGCAACAGCACGGTTACGGTCTGCATATTCACCGGGCCAGTAACCCGCGCCTTTTCGGGGCGGGATAAGCGCGCTGAT
TTTCTTACGCCGCAGTTCATCGTGACAGAGCCGGGTGTCGTAAGCGCCGTCTGCCGATGCTGCCCTGATTTTTCTGTGAGTCT
GCCGGATAAGACCCGGGAAGGCTTCTGAGTCCGTCACATTGTTCAGCGACAGGTCTGCACAGATGATTTCATGTGTGTTGCT
GTCAACGGCCAGATGCAACTTTCGCCATATACGACGGCGTTCTTTGCCGTGTTTTTTGACTTTCCATTCGCCTTCACCAAAGAC
CTTCAGCCCGGTGGAATCAATCACCAGATGCGCGATTTCACCCCGGGTGAACGTTTTGAAACTGACATTAACCGACTTTGCGC
GCTTGCTGACACTGGTGTAATCCGGGCAGCGCAACGGAACATTCATCAGTGTAAAAATGGAATCAATAAAACCCTGTGCAGC
CCGCAGGGTCAGCCTGAACACGCGTTTAATGACCAGAACGGTGGTGATGGCGAGATCAGAATAGCGCTGAGGTCTTCCCCG
TGATGAAGGCGTTGCCGACTCATACCAGGCCTGAATAGCTTCATCATCCAGCCAGAAAGTTATGGAGCCACGGTTGATGAGG
GCTTTATTGTAGGTGGGCCAGTTGGTGATTTTGAACTTTTGCTTTGCCACGGAACGGTCTGCGTTGTCGGGAAGATGCGTGA
TCTGATCCTTCAACTCAGCAAAAGTTCGATTTATTCAACAAAGCCGCGCATGGCTTCAGAACCATCAGAAACAAACGCTATACC
GGCGATGGAGGCATTGACGGCCAGGTAATAATAGGAAAATATCGCTATCTTATTCAGGCTAAACGCTATCGCGGCCATATTGCT
TTACAGCACGTACAGGAGTTCGAGAAGTTGCTTAAACGTCATAACTGTCGCGGTCTGTTTTGCCATACCGGGAAAACCGGCG
CAGGTTCAAAATCTGTCAGTATTGCCAGTGAACGGATGGAGATTATCAGCGGCCAGCGCCTGATAGATTTGCTCACGCCCGGC
AGCTCCTTCACTATCGCAACCGCCCCGCAGACGATGATGAAGCGTACCGCAGCAACACTAGAAACGAGCACCATTGTTAAAG
ATGCCGGTAAAGAAAATCGATACCATGAGAGTTAATTAAATGAAGTCAGTAACTATAGAAGCAAAAACATTTGCTGAAATGTTA
GGAATAACAGAAGGTGAATTAATCTTTGCCATTAAGAAAACTGGCACATTCAAAAACAAGACCATCCCACAACCTCATGAGCC
ACATAAATCAAATAATAGATTTTTATATTCAGACGTAATGAGGTTTATAGAATCACTAAAAGACAAAGAGAACCGGTAATGACTC
CAACTTACTGATAGTGTTTTATGTTCAGATAATGCCCGATGACCTTGTCATGCAGCTCCACCGATTTTGAGAACGACAGTGACT
TCCGTCCCAGCCTTGCCAGATGTTGTCTCAGATTCAGGTTATGTCGCTCAATGCGCTGAGTGTAACGCTTGCTGATAACGTGCA
GCTTTCCCTTCAGGCGGGATTCATACAGCGGCCAGCCATCCGTCATCCATACCACGACCTCAAAGGCCGACAGCAGGCCCAG
AAGACGCTCCAGCGTGGCCAACGTGCGTTACCTAACAATAAACCTGTTTAAATATCCAGATAAAAACATTCAATCTGGGTCAAA
TGAGTGATACAGTTTCACCCATAAGACCCAATGGAGGCAATATGTCTGAATTTGAATTACTGGCGCAGGATCTGCTTGAGAAA
GCAGAAGCGGAAGAACAACTGCGACAGGAAAATTATAAAAAGCTGCTCGGGCAGGTGCTGGAAATCTATGACCAGAAGTAC
GTGGCTGAACTGCTTAGAAAAGTTGGTAAAAATGAGTGGAGTCGCGAGACTCTTAATCGCTGGATTAATGGTAAGTGCTCAC
CTAAGACGCTGACGTTAGCCGAAGAGGAACTTCTACGAAAAATGCTTCCGGAAGCGCCTGCACATCACCCTGACTATGCCTTC
CGGTTTATTGACCTGTTTGCTGGGATTGGAGGTATACGGAAGGGCTTCGAAACCATCGGTGGCCAGTGCGTTTTTACCAGTG
AATGGAATAAAGAGGCTGTGCGCACATATAAAGCTAACTGGTTTAACGATGCTCAGGAACACACTTTCAATCTCGATATTCGG
GAAGTCACGCTCAGTGATAAACCTGAAGTACCTGAAAACGATGCCTATGCTTACATTAATGAGCATGTGCCGGATCATGATGTA
CTTCTAGCAGGTTTCCCCTGTCAACCGTTCAGCCTTGCGGGCGTAAGCAAGAAAAACTCGCTCGGGCGCGCGCATGGTTTCG
AATGTGAGGCTCAGGGAACGCTTTTCTTCGATGTGGCGCGTATTATACGCGCAAAAAAACCTGCCATCTTTGTTCTTGAAAAC
GTTAAAAACCTGAAGAGCCATGACAAGGGTAAAACCTTTAAAGTCATCATGGATACCCTCGACGAACTGGGCTATGAAGTTG
CGGATGCAGCTGAGATGGGCAAAAACGATCCTAAAGTTATCGACGGAAAGCACTTTTTACGGCACTGTTGCAAATAGTCGGT
GGTGATAAACTTATCATCCCCTTTTGCTGATGGAGCTGCACATGAACCCATTCAAAGGCCGGCATTTTCAGCGTGACATCATTC
TGTGGGCCGTACGCTGGTACTGCAAATACGGCATCAGTTACCGTGAGCTGCAGGAGATGCTGGCTGAACGCGGAGTGAATGT
CGATCACTCCACGATTTACCGCTGGGTTCAGCGTTATGCGCCTGAAATGGAAAAACGGCTGCGCTGGTACTGGCGTAACCCTT
CCGATCTTTGCCCGTGGCACATGGATGAAACCTACGTGAAGGTCAATGGCCGCTGGGCGTATCTGTACCGGGCCGTCGACAG
CCGGGGCCGCACTGTCGATTTTTATCTCTCCTCCCGTCGTAACAGCAAAGCTGCATACCGGTTTCTGGGTAAAATCCTCAACAA
CGTGAAGAAGTGGCAGATCCCGCGATTCATCAACACGGATAAAGCGCCCGCCTATGGTCGCGCGCTTGCTCTGCTCAAACGC
GAAGGCCGGTGCCCGTCTGACGTTGAACACCGACAGATTAAGTACCGGAACAACGTGATTGAATGCGATCATGGCAAACTGA

AACGGATAATCGGCGCCACGCTGGGATTTAAATCCATGAAGACGGCTTACGCCACCATCAAAGGTATTGAGGTGATGCGTGC
ACTACGCAAAGGCCAGGCCTCAGCATTTTATTATGGTGATCCCCTGGGCGAAATGCGCCTGGTAAGCAGAGTTTTTGAAATGT
AAGGCCTTTGAATAAGACAAAAGGCTGCCTCATCGCTAACTTTGCAACAGTGCCGATTTACGTGCATAGCCGATTTTCATTCTT
TTCTCGCTAATTAGTTATGGGGTTATTGTTATGTTGATACAGTAACGAGTTTTGTTACATGAGGGGAGTCATTTTTCGGGAGAA
GTCAGGACTTTTCAAGACTGTCACAAAAACCATCGTTTTTGATACATTAATTTAACCAATAGGTTGCAGATCAAATCGTCTGTA
ACAGCCTTTCTGGCTGTTTGTATATAATCATGAAAAAATGGTGAGTAGAGTTTCAGGGTAACAGGGGATGCTTATGTCGGTTTT
CCACAACTGGCTACTTGAGATCGCATGTGAGAATTACTTCGTCTACATCAAACGCCTTTCCGCCAACGATACCGGCGCAACAG
GTGGTCACCAGGTAGGGCTTTATATCCCTTCAGGTATCGTTGAAAAACTCTTTCCGTCTATCAACCATACCCGTGAACTGAACC
CTTCGGTTTTTCTCACCGCACATGTGTCATCGCATGATTGCCCTGACAGCGAAGCCCGGGCAATTTATTATAACAGCCGTCATT
TTGGTAAAACCCGGAATGAAAAAAGGATTACCCGCTGGGGTAGAGGCAGCCCACTTCAGAATCCTGAAAATACAGGGGCTC
TGACGCTCCTGGCTTTCAAGCTTGATGAGCAAGGGGGGACTGTAAGGAAGTAAATATTTGGGTATGCGCCAGCACTGATGAA
GAGGACGTCATTGAGACCGCTATTGGTGAAGTTATACCCGGAGCGCTTATATCCGGCCCCGCAGGACAGATTCTAGGCGGAC
TATCTCTACAGCAAGCGCCAGTAAATCATAAATATATTCTACCTGAAGACTGGCACCTGCGCTTTCCGTCGGGAAGTGAAATTA
TTCAGTATGCAGCCAGCCATTATGTGAAAAATTCCCTTGATCCGGATGAGCAACTTCTTGACCGCCGGCGCGTGGAGTACGAC
ATATTTCTATTGGTTGAGGAACTGCATGTTCTGGATATCATCCGGAAAGGATTTGGCTCTGTGGATGAATTTATTGCGCTGGCC
AATTCTGTCAGCAATCGCCGTAAATCCAGAGCCGGGAAGTCTCTGGAACTGCACCTGGAGCATCTATTCATTGAGCACGGCCT
GCGACACTTTGCGACGCAGGCCATCACAGAAGGTAATAAAAAACCCGATTTCCTTTTCCCTTCCGCAGGGGCTTACCACGATA
CTGAGTTTCCCGTAGAAAATCTGCGCATGCTGGCAGTCAAGACTACCTGTAAGGATCGCTGGCGTCAGATACTGAATGAGGCC
GATAAAATTCATCAGGTGCATCTGTTTACACTCCAAGAGGGAGTTTCTCTGGCTCAATATCGGGAGATGCGGGAGTCGGGTGT
CAGATTGGTCGTGCCATCATCGCTGCACAAAAAATACCCGGAGGCGGTGAGAGCTGAGCTAATGACGCTAGGTGCGTTTATT
GCTGAGCTGACAGGGCTTTACGCAGATATTCCATAGATTATCTCCCGGCATAAATACCGGGAGGAGCGATCAGATTCGTTCAA
CCTTGCACGAATCGGCATTAACCGCTTTCAGGATATAAGGTTCAAGCAGTTTGGCTACGGCTTCAAACACGGGCACCACTACG
GAGTTACCGAACTGCCTGTACGACTGAGTGTCTGACACAGGAATGCGAAAAGGCCTGCCATCTACTTTTTCAAAACCCATAAG
GCGCGCGCACTCTCGTGGAGTCAGCCTGCGGGGCCGATGCGCCTGATTTTCTTCGTTCGCGAAGTCTGTTTCACCTGTGGCC
ATATCCCAGCCACGGTCTATCAGAATTTCAGACCCGTCTTTGTGATAGCGAGCAGAAAGCGTACGGGCAATGCTTTCTTTATTT
TCAGGATTAACGAGGCCAAAACCGAATCCGTTACCCTTAGCTGCGTGCTTTTTGGCGTAGTTATAGAGATACTCCCAGAGTTTC
GGCGTCAGTATATATTTGCTGTCAACCACGGGTTCCAGCAGTTCGCCAAATGACGGACGCTGTTCCGGATAAAAACGACTAAT
ATCGCGCAGGGTAAAGCCCTGGTGAATGTTCAGATCACGACGGAAACCGACCAAAACGATACGTTCTCGGTGCTGAGGTAAA
AAGGGCACTGTTGCAAAGTTAGCGATGAGGCAGCCTTTTGTCTTATTCAAAGGCCTTACATTTCAAAAACTCTGCTTACCAGG
CGCATTTCGCCCAGGGGATCACCATAATAAAATGCTGAGGCCTGGCCTTTGCGTAGTGCACGCATCACCTCAATACCTTTGATG
GTGGCGTAAGCCGTCTTCATGGATTTAAATCCCAGCGTGGCGCCGATTATCCGTTTCAGTTTGCCATGATCGCATTCAATCACG
TTGTTCCGGTACTTAATCTGTCGGTGTTCAACGTCAGACGGGCACCGGCCTTCGCGTTTGAGCAGAGCAAGCGCGCGACCAT
AGGCGGGCGCTTTATCCGTGTTGATGAATCGCGGGATCTGCCACTTCTTCACGTTGTTGAGGATTTTACCCAGAAACCGGTAT
GCAGCTTTGCTGTTACGACGGGAGGAGAGATAAAAATCGACAGTGCGGCCCCGGCTGTCGACGGCCCGGTACAGATACGCC
CAGCGGCCATTGACCTTCACGTAGGTTTCATCCATGTGCCACGGGCAAAGATCGGAAGGGTTACGCCAGTACCAGCGCAGCC
GTTTTTCCATTTCAGGCGCATAACGCTGAACCCAGCGGTAAATCGTGGAGTGATCGACATTCACTCCGCGTTCAGCCAGCATC
TCCTGCAGCTCACGGTAACTGATGCCGTATTTGCAGTACCAGCGTACGGCCCACAGAATGATGTCACGCTGAAAATGCCGGCC
TTTGAATGGGTTCATGTGCAGCTCCATCAGCAAAAGGGGATGATAAGTTTATCACCACCGACTATTTGCAACAGTGCCGGTCG
CCGGGAGTCAGCAGATCGACGTCAACGCCGAGCAGCGATTTCAGTTCTTCTTCCAAATCGCCCAAGTCCAACAACGTGGCAC
CGGGCAGCGCATCGACCAACAGGTCGAGGTCGCTGCCATCCCGGTCGGTGCCATGCAGCACCGAGCCGAAGACGCGCGGG
TTCGCGGCGCGAAAGCGGCCTACCGCTTCACGCACTGCGCTTCGCTTCATGTCAAGCACAACAGACGGTCGCATGCGCATCC
TTTCTTATCGAAACTCGTTGAGATGATATGCAATCAAGAATAGAATTTCAAGAACTCACAAAGTAACGCGGTGGTTAATATCCT
GTACCCACGGATTGCCCTTAGCGCTGCCTATATCGGCTAAAGCACTCCGGTAGCTTGATTCACCCCACGGCCACGGCAGGATC
TTGGCCGTCGCAAGCGCCAGGGGAAAATCTTCAGCTGCAAGCCTGAGTGATTTCATGTGCGTGTAATCCATCGCCCAGATGAT
TTTTGTGAAGAAGAACTCGCGCTCGTTCATGTCCGGGCGCGCGTCCTGGCCCTCCGTGCCAACGGCCAGCAAGTAATCGGCC
TGAATTGGCAGTATCAGCGCGCGTAGTAAGTCATGGATCGCCGGTTGCGGCAGTCTGCGCGCCAGATTAATTACCCGGTCGA

ACTTCAGTTTATCCTGCTTGCGCTTGTCGGTCTGCTCCATCAGATTTCATGGCCCCCTTCTTCATGCTCATGCTCATGGGTGTGT
TCTTTTCCGGTATGGCTCTGTTCCGCCTGAGACGTCTGCGGCATGGCGTAATCGTCGTAAATGCTGCTGTCAAAGTCGTAGTCT
GATGCTTCGGCATAATGCTCGTAATCGGCATACTCCTGCGCGCTCCACTGCTGATCGTCGGCAGCAGCATAATCATGGCCAGC
TCTGCATCATTTTGCTGTGCTTCATGACGCCGCAGGCCAACGGAATCATCCATAGGGTTCTGCTTAAGATGAAAGGCGTCCTCT
GCGTTGCTCACCGGCTGATAATCAGTGCCGGTTGTCATGTTATGTTCATCGGGTTTCTGGTTAAACGCCATGCTTTCCCCCGTG
GCTTCTGGCAGACCTTTTTCAGCTGATCGGGTTTCTAAACTGGTATCGCGGCCAATATCCTTAAACCTGGCCTCAAGCCCAAA
GAAACGGTCAATTTCTGCGGCCGTGGTTTTCGGGCTGTCGCGGCTCACGCTCGATGCCAAAGATTTTTATCGTCGGTAAAAA
TTTCCACCTCATGACGCGCACGCGAAATACCAACATAAAAAACGTCCTTAGAAGTGGTAAGCGATTTGGTATCTATGTTGAACA
ACACGCGATCACAGGTAAGCCCTTGGGATTTGTGGACGGTGGTTGCATAAGCATAGGAAAGATAAGAAGCCTGTTTTTTGTC
CAGCTCAACCGTGCGCCCTTTTTTGTCCTCAAGCGTCAGTTTTTCACCCTCCACGGTTTTCACCGTGAAGCGGTCGCCGTTGG
CAACGTCCAGCGTTTTATCGTTACGCGTTACCATAACCTTATCGCCCGGCGCCAGTTCGGCGCTGACTGCCTGGTATACAGACA
GCTTGGTGTGTGTACGCGGGCTGAAAGCGATCTGCTCACCGCTGCTGCTTTCAACCGTCAATTTGTTGCCCGGCCCGGTATCA
AGAACCTGGTAAGACTCGCCCCGCTTCATACCATTTTTGTAATCCTGTTCGGGGATAATGATTTGCCCTTTACTGAAATAACGG
CTGTCGCGGCGTTCCGCCTGTGTCGAATCCACGCGGTCAAGTAGCGTGAACGTTTCGCCGGTTCCGGCAAGCCCCAGATTGC
CCCGGATGTAGTCATTGAGGGTTTTGCGTGAGGCGTTCGTACCAGAGATTATCAGGGTGGCATCCTGTTGTTCTGAGGACAG
AGACAGGTAGCGATCGGCAAGTTGAGCGAGTCGGGGCGCTTCTTCCTTCAGTTCGTTCACGCCGGTGATATTTTTCAGGGCG
CGCGCGGCATTACCTTCAGCGGCATACTTAACCGCCTCAAGCAAAACTTCATTCTTCTGTCGCTGAATGTCTTTCATGTAGCTG
GTCTGCATATCTGCTTTAATCAGCTGCTCAAAAGGCTTACCGGCTTCTACCGCTTTCGTCTGTGACGTATCCCCCAGGAATACCG
CGCGAGCGTTATGCTTCTCGATCACCTCCATCAGCTGTTTCATCTGTCGGGCGGGTATAACCCCGGCTTCATCAATGAATACGA
CTGATTTTTCATCCAGCTTTTTATCCTTCGCTTTGAGGAAAGCGGCAACGGTGCGGGCCGGTAATCCATCATCTTCAAGCGCTT
TTTTCTGTGTCCCATAGGGGGCCAGCGCCGTGACCTTCAGCCCTTGTGACTCCAGCAGCTCTTTAGCGGCCATCGTCATATAGC
TTTTACCGGTACCGGCGTAACCATGTGCGGCCACAAACCGATCTTTGCTCGTCACAATTTCTGTAACCGCGCGCATCTGCTCCT
TCTTGAGGGTTTTCCCGGCAAGCAGCTGGCCTGCAATCTCTGCGGTCAGCTGTCGCGGCATCTGCCCCCGGCCGCGTGATTC
GATAGTCAGAATGGAACGCTCAAGGCGAATACCCTCCACGGTAGTGACGCGGTGGCTGGTCTTTTTAAGCCTGCCGTTTTTAA
TACCATCATCTACCGCAAAACGGGCTTTATCCGCACGCATCCCGCTATTCGTCAGCGAGTCGATCCACTCTTTGCGCGTCAGAG
TTTCGGCCATAACTGAAGCACCGACCTTCAGAGTTGATTGATACCGGGCTTCGCCCTCGATGATGGCGCCCTTCTGTACCGCC
TTCAGGTACGCTTTTTCAACATCGGCTATTGTGGCATGGCCCAGCACCTGCTTATTAGCGATTTGAATCAGCTTCTGGCGTTCA
AAGCTGGCATCGCGCTCTGACAGCGACTTAACTGCAAACTGGATAGCCCGGTCAGCTTTAACCTCCGGGCTGGTAAAATCCG
GGGCCATGTTGCGCGCTATATCAGCCTCCAGAGGTTTACCGTGTCCCTGCCATTCACGGTTATCAAAATCAATGCCGAGCGTTT
TGGCGCGGCTGGCCCATTCCTGGTGAATTTCTTCACGGGAATGCTCTGTTTTCTTTTCACGCGTAGCCATCGAGACGCGGCTT
TTCGTCTGAGCATCGGCGGTTTCCCGCGTCAGACCCATTGCAGCGAGTCCCTTTTCAATTTGCTCCGACCGGCGGGAAAAAG
CGCGAATCTGTTCATCTGAAAAATGGGCCATATCGAACGTGTTATTTTTGCTGTTGTAACGCAGCTCATAACCGGCTTTGGTCA
ACTCCAACGCCAGCTCCTGTTTGTAAACATCGCCCAGGTGCATTTTGTTACGCATCAGCTCATCATTTTTGAGCGCGCGCCACT
GGCCGTCCTCGCGCTGGGTCATGTTCATGACAAAAGCGTGTGTGTGCAAATCAGGATCTAGCGCCCTGGAAGTTTCGTGGCG
GAAAGTAGCGACGACAAGGTTATTGGTATTCTGGGTTACTGATTTCCCCTGGCGAGTTGTCCGGGCCTGCGCGAGTTTTTCA
GCTTCACGCACAGCAGCGGCAACAGCTTTTTCATGAGCCTCGATAATGGTTTTATCGCCGTGTATCAGCGCCTGCATGGATACC
CCTTTAGGCGCTGAAAACGTCAGGTCGTAGCCCAGACGCTCTTTTTTGGCATCACCCACGTGTCGCTGCATATGCGTGAAGGT
ATCTATCTCTCCGACAAGCAGCTCTTTAAACCGGGCTGATTCAACGTCCCCGGATAAGCCGAGGGCTTCAGCTCCGGTTCCCT
GCCAGGACGTGAATGATGAATCCTTACTGTAGTAATCATCCTTTGCATCAGAGTAGTAGCCCACAACGCTAGTGACGTTCTGGC
GGGTAATCGTGGTTATATCAAGCATCAGATCTCCCTCAGTTCAATGCCAGGAACAGGGTTTTTGCGATGGTATTTAACGTGTTT
AGCCTTGAACTTAGCGACGGGCATATCACCAGGCAACGCCAGATAGCCGGTGAGGTTTGGCAACATTGATATTTCGGTAGGC
GTTACGGCACGAACAACTTTAACGTCGCGGCGTTTACGGACAATCCAGGGCTTCTGAGGATCGGATTCTTTACGCTCAACTTC
GCCTTCTATCTCACCGAGTGAGCGCGACATTTGATCCAACGTTTCATCACCGAGACGGCTGCCGCCCAGCACGATGTTAGAAC
GCATGTTAGCCAGAATTGTCTGAGCCATATCCCGACCATAAACCTTAACCAGCTGAGAATAGGTTTGATAGCCAGCATAAACAC
ACAGACCGCTTTTACGCCCTTTGGTCAGTGCATCGTTGAGGTTTGGCAGAAACTGGAGTGATTCCAGCTCGTCAATAAATACA
TTAATGCGGCTTTCTTTTTCACCCATACCCAGCACGATAGAAAAAATCGAATCCAGCCAGCAGGAAATTAGCGGATTAAGTGA

CCTTTTCATTTCTTCCTGCCAGGTGATAAACAGGGTTCCCGGCTTTCCATCATCAAGCCAGTCACGCAGGGAAAAATTACCTTC
CGGCATTTTCAAATGTGGGGCAAGATTCTTACTGAGAACAAATCGCGCGCTTCCAACTGCTTTTTCAGACCCGGAAAAAAATAG
CTTCGGCAGGCGTCCCCATTAAAAATTCTTTTAATTTTTTCTGGTCAACGTTACAGGCCCAGTGAATAACTTCTTCCATAGTTAC
TGTGCTGTATAGGCTGTGAAGTTTTTTCGAAACTTCACTAAAAATAAGACGGCCATAGCCGAACCATTCTTCAGTAGCCATATC
AGGGCTTTCCTGAACAATAGAGTTCACTAAACGCTCGTAATCATATGAACGGCGAATTTCATTGAAAAACACCCAGCCTTCAG
TGCGTTTATCATAGGCGTTTAAAATAACATCGCCGGGACGATAGAAATTCTTTAAGAACCCCCCATTTGGATCTAAAGCAATATT
TTTGCCGCCTCTAATGATGCTCTTAAATAACAGTTCATTGAAAATTGTGGTTTTACCAGTACCGGTTGTACCGGCAATCGAAAA
ATGCAAGTTCTCAGCGTATGTAGGTATGGGGATATTAGCCACGGTTAACTGGTTGACACCTCTTTCGCGTGTTTTATCAGCGAG
TGTTCTGGCGCGAACAAGCTCTGTACCACGATAAATCTTTTTGAATCTTTCGCCTTTAAACACGCGTGATTTATCATAAATGATA
AAAGCGATCAGACCGCCAACACCAATAAACCAGCCAGCAATTAAAGCTGACCATAAAGGCCATAGCGAAAAAGTATTCTTAA
CCAGATACGGAATCAGGTATTTAGCCGTGGATGGATCAATACCGTAGGTAAATTTTGCAACTAGAAACCATACCATCACTGGAG
GCAAAGTAATTGCAAATAAAAATGCTAAGCCTCTTTCTCTATCGTCCATTTCAGCGCTCCTTTTTTGGTTCCCAGACTTTGTAGC
CGTTACGTTCAACCTCTGCTTTTGCCGCTTTGGTTTTGCCCGGTTCTGCTATCGAGCGCAGGAGGATTAGCGTTTCAATAGCG
AGTGATTCATGCAACATCATCTGTCCTGCCGGTGGGAATTTTACGCCAGATAGCGTTTCGGTTATTGCCTTCAGCTCATCGCGC
AGTGGGCCAAAATCCGCATCTGAAGCACGGTCAAAAAGATAATCCAGTTTGCGATTTACGTCGCTCAGCCGGTCGGCAACTAT
TTTCAACCCGGACTCCCGATCACCTGGGCCAGCTTCAATGCAGCGCCGCAGATAATCTGACCGATTACCTCCTGAAACCAGGT
CTATATAGGCCAAAAGTTCATCTGATACTTTTGCGGTTATTATTGGCATTCAGTCCTCACATTGTGCATTTCTTAAACAAAAAATT
GGGATCTAACAAGCTGAAATCTTAGTATTACCAAAGTAATAAAGCAAACTCATTATAAAACAATGGGTTATTGGGTGTTTTTAAT
ACCTAATTATTACCGAATATTGACGCTATTTATTTTTTTATCTTTTAAATCAGTACGATAGCGTGATTTATCGCGCTGCGTTAGGTG
TATAGCAGGTTAAGGAAAAAAAATCATCTTTTTTGGTAGGAGCGACCTCCGTAGGTTAAGGGTCATTTGGCTAAAAAGCGTCC
TATTCTTTGATGGTCATGCTTGCATGACCATCTGAGCAACCAAAAACTACAGATAAACTACAGAGAAACTACAGATAAACTACA
AAAAACGATTTACCTTAGCGTTGTCAGACTACTAATAGACTACAAGGAAACTACAAAGAAACTACAAAGAAACTACAAAGAA
ACTACAAATAGACTACTAAAACCGTGGCAGACTACTAATAGACTACAAGAAAACTACAAATAAACTACAAAACTGGATTGACC
CCTTCTTACGAGTGTTGTAGAGTCATCTTCATACAACGGAGGGGGTTATGAATAAACCAGCAGATCTGAAACCCCGCAACTTAT
CGGCTGCTGTCAGATTGCGCCTAAATGAAATCGAGAACTGGCTGGACAGAGGGCTAACGCGGCATGAAATTGCTGAAATCCT
CGACAGCGAATACAGCTTTTCGGTAACAGCCAAAGGGCTTGAGATGGCACTGTATAGAACGCGGCAAAACCGAAAAAATGT
ATTGCACAATACACATGATAAGAGTAGCGCGAAGGGTGCAGCGGAAAGTGTATTGCACAATACACAACCGTCTGAGCCTGAA
GCGCAGGAAAGTGAAAAAGCAGAGAGTCCCGGCATTATTGATAAAGAGTTCTTCAATAAAATCGGTGAGGATTTCAACCCTA
AGAAGTTCAACAAAAAATTCTGAGGTGATTTATGAAAGTAGCGGTAATTAATTACAGTGGCAGTGTTGGTAAAACATTAATTT
CATCTTACCTGTTAGCCCCGCGCCTGACTGGTGCAAAGTTCTATGCGGTAGAGACTATCAACCAGTCTGCTTCCGATCTGGGTA
TTGAAAATGTGACCAGTTTTAAAGGTGACGACTTCTCACGTTTGATTGAGGATATTGTTTTTGAAGATGCAGGCATTATTGATA
TTGGCGCGTCAAACGTTGAAGCGTTCCTGATGGCTATGTCTCGCTTTGACAGTGGCGCGAACGAATTTGATAAATATGTAATCC
CGGTGACGCCGGATAATAAGGCGATTGATGAAAGCCTGAAAACGGCACACACGTTAAGTAAAGCGGGCGTGAGCAGCAAG
AAAATTATCTTTGTTCCAAACCGTATTAGTCCAGACAGTGAAGTAGAAGATGTGCTGGCGCCGGTGTTTGAGTTTGTCAAAGA
AACGAAGATTGGCAAAATAAGCAAGAAGGCTGTTATTTATAACAGTGAGGTTTTCGAATATCTGGCGTTTCACCGTATCTCATT
CGAAGTATTGACCGCTGAAGATCCAGAAGAATTCAAATCCCGTGCAAAACAAACAACCGATGCTGACGAGCGCAAAAAACT
GGCACGCCGTTATACATACATGAAACAGGCGATTCCGGTAAAAGCTAATCTCGATAAAGCATATGCGGCTTTAATGGGAGAAT
AAAATGGAAAAGCAGCCGGATAAATTAGAAGTTCTGATGGACTGGTTTTTAGGTGACGCGAAGGAAATCACCGCAACTCAG
AAAGAAATGACGCAGAAACTTTCTGAGCTTTCGGAAAAGCTGGCAAAAGACACCGAAAGTTTAGGAGAGACGGCAGACTC
TTTTAAACGGGCTTTAGTAGAAAACCAGCGTTCAATTAGCCTGGCAATTAGTGATGATGCTAAGGCGCGCGAGGAATTTCTAA
CTAAATTCCGCCGCGCGCAGGCGTCCAGTGCTGAGACGTTTACCCGTCAGATCCTTTTTATTACAGCTGGCTGCACCATCGTG
GGCGCCGCAGTAGGCGCCGCGATAGCGATACTTTTACTGAGATAAAGCAAACCGGGCGTGTCCCGGTTTTTTTGTCAAGCGG
AGCGCGGAGGCCGAAGGCCGGAGGCATTAGTGGCCGCCGCCCGCGTAAGCGGGGCGAGACGGGAACCGGCTCGAAGCG
CAGCACGGCAGAACGGCCCCGCAGGGGCAATGCCCGTTTTAATTCATCGTGACAGTCGCGCGTGACCATCACGGGGAGAAA
AATAATGAATGACCGACAGCGAGAACTGGCCCGTATACGCCAGGCCCGCCGCCGCGCGCGGCTCAAGGAAGAAGGCACAA
GCGTGACAGTCACGCTAACAAAACAGGAAGAAGCAATGTTGCAGGAGCTGTGCCGGGTTCGCCGTCCTGGACGAACGCCTT

ATTCAACGAACGAATTTTTCCAGCTGCTGCTTATCCGCAACTGGCAGCAGTGGCAGGAGCAGAAGGCACAGCTGGGAAAAT
GCCAGGCTTGCGGAAAGCTGAAAGCGGAGGGGGGGTGCGAGGGTGAACGGAAAGGCGAAACCTTTAACTGCTGGCTTGC
CGTCGAAGCCAATGAACTAAATTTGTAGTGTATTGTGCAATACACATTTACACAGAAACAAAAACCACCGGCAATTCCTGGAA
CCGGATACCTACGGCTATTCCTGGGTGAACGGTACTTTTTGCACCTGGGTGCGCTGAAAAAGCTGAATATGCAGGGTGACGT
TCGCGTGCTGTTCTGCTTTGTAGACTGAATGCGCCAGCTATACGCCTGACTGCTTAAACCTGGTAAAGTTCTGCAACCGGCAC
TGACCGGAAAGCAAGGCAGGGAAGACCTAAGCCAGAAACCTTGACTGCTCCCCGCCCTTCAGGGCGGGGATTGCGGATCA
TGTTCTTCTCTTTCAGGGATTCAACGCAGACAAGAAAGGCTTTCAATTTCCTATACGTGAACGGCCGCGCAGCGGAAGAAAC
AAGCCCGGTCAATCCGGGCTTGTTTCTTTAGGCGGCTCAGAAATCGCCTAAAGGCCCGGCTTGGCCGGGCAGTCAGTGCTAT
TTAGTTTGTTGCAGCAGCTGGCTTAATTTTGCCGCCAGTGCATACGTCGATTGGAAAGCGCCTTGCAGGCGCTGATTAGGCAG
CTGGTTGAACGCTTCGAGGCAGGCGCGCAGCAATAATTCTTTCTGAGATTCGACTTCTTTTTTCAGTTGGGAAGGGGTGGTA
ACTGTAGTCATGCTTGCTCCTTAGTGAGCCGATATCGGCAATTTTTCGGGTGGCGGTGTTGCCTCCCGATGATTTAATTATCGG
TGATTATGCTTTTAAAGTCAATACAGGTACGGAATTTATTTACCTGTTTTTATGCCCGTCAGGGCATGGAAGGCGACCGCGCCG
GACTCCACCGGACACCGGCCGCAAATCGCCGGAAACTGCGGGACTGACCGGAGCAACAGGCCAACCCCCCTCCCTGCTAAG
CCATAACCCAGCCCGCCGCCACGCAGCTGCCGCACGTCCCCCACGGGGGTGCGCAGTGGGCGCCGCGCGCCTGCGCGCGG
GTACGGCGGCCCGCCTGCGGGTCGCGGCGCCGTACTGCGAGTTAGCGGCCGCCGCGCGGCCGGTTACGGGGGACACCGCA
CAGTCACGGCCAGTGCCCCGCTGAGCTGCACAATCCACGGATAACACAATAGCGCACTGGCAAAGGATGCCGACGCCTGAA
GGGCGTGGGCACCCCGAAGGGGCGGGGCGGCCGCTTGCGGCCGGGCGAGTCCGGCGCAGGGTGTGGCCTGCCAAGCGG
AGCGCGGAGGCCGAAGGCCGGAGGCGTTAGCGGCCGCTGCCCGCGTAAGCGGGGCGAGACGGGAACCGGCTCGATGCGC
AGCACAGCAGAGCGACCCCGAAGGGGTAACGCCCGGTGTGGCATCAGGATTTAGTGCAATGGCAGAACATGAGCTGGAGA
GATCACCGGCAAGCAGCAGCAAAGGGGCGGCACAGCCGCCCCGATGGCTGTTTGCCGATACCGGCGATTAATTAGAGCGGT
GTTTAATATCCCCCGCGTTGCGGGGGACTAGGTTTCAGCAAGTCATGTTAAATACGTGTCCATCATGTAAACTGAAATCCCCAA
TAAACAGATCCCGCGCATAGGCTACGATGTCAAAATATCGGGCTACGGATTCCGGAATATCATTCAGTAGACCGCTATCATTCA
GGTATTCCTCTGCAAAAGTTTCTTCGTCCTTAGCTTCGCCCATATAGGCATCTCTAAACAGGTCGAAATCAGTGCTATTAAACAG
ATCAACAAAGGCCACAAACGCCGCTTCGTTACCTTCCTCGCGGGCTTGTTTAAAGCCGTTAATAAAATCCCAGTTGATATGGC
ACTCTGACGCCATACCAGACGGAATACCCTCCCAATCTTGGAACATAAATTCTGGATCAGCCTCATTTGCGTGTAACTCGCGGC
AGCGCTCGTAAAACTCCTCTGAGCTATCAAAATCGGTCAGATCGAGCCAGGCTCCCGCAATGCTTCCGCAGTTGTATTTATGGT
AAGTGCCAACATAAACAGAAGGGGTCGTAATATCAGTCATGGTGTACTCCTTAAAGCGCCGATACCGGCAATTTTTCGGGCGG
CGGTATTGCCTCCCGATGATTTAATTATCGTTGATTATGCTTTTAAAGTCAATACAGATACGGAATTTATTTACCTGTTTTTATGCC
CGTCAGGGCATGGAAGGCGACCGCGCCGGACTCCACCGGACACCGGCCGCAAATCGCCGGAAACTGCGGGACTGACCGGA
GCAACAGGCCAACCCCCCTCCCTGCTAAGCCATAACCCAGCCCGCCGCCACGCAGCTGCCGCACGTCCCCCACGGGGGTGC
GCAGTGGGCGCCGCGCGCCTGCGCGCGGGTACGGCGGCCCGCCTGCGGGTCGCGGCGCCGTACTGCGAGTTAGCGGCCGC
CGCGCGGCCGGTTACGGGGGACACCGCACCGTCACGGCCAGCGCCCCGCTGAGCTGCACAATCCACGGATAACACAATAGC
GCACTGGCAAAGGATGCCGACGCCTGAAGGGCGTTGGCACCCCGAAGGGGCGGGGCGGCCGCTTGCGGCCGGGCGAGTC
CGGCGCAGGGTGTGGCCTGCCAAGCGGAGCGCGGAGGCCGAAGGCCGGAGGCGTTAGCGGCCGCTGCCCGCGTAAGCGG
GGCGAGACGGGAACCGGCTCGATGCGCAGCACAGCAGAGCGGCCCCGAAGGGGTAACGCCCTGTGTGGCATCAGGATTTA
GCACAATGTCAGAACATAAACTGGAGAGATCACCGGCAAGCAGCAGCAAAGGGGCGGCACAGCCGCCCCGATGGCTGTTA
CTTGTCTTTGTCGCGTAGCACTTTGATTAGGCCGGTTACGGCCGTAATCAGAGCGGCCAGCGAGGTGATGATTTGCGGTAGG
TTTTCGAGGATGGTAGAGGTCATATAGCACCTGTAGAGAAGTTGGCGGGGTGTCGTTTCCGACGGCCGCACTGTAACCGGGC
GAATAAGGCAGGTTGTCAACAGCTTGAGCGAAGCGTCTGTTGACAACCTGCCGCGCCCGGTTTCACTGCGGTCATAGGCGG
AACGACCCACGCCAACGGAACGGCTTTATGACCGGGCAGCTGAGATACCGGCGAACCTGGCTGGCGGCTGACGCCAGCCG
CCAAGCGCCAGCGCGGAGGGCAAAGCCCGGAGGCCAAGCGGAGCGCGGAGGCCGAAGGCCGGAGGCCGGAGGCGTTA
GCGGCCGCTGCCCGCGTAAGCGGGGCGAGACGGGAACCGGCTCGATGCGCAGCACAGCAGAGCGGCCCCGAAGGGGTAA
CGCCCGGAGTCTGCCGCTGTTTATCTCTCGTTCCATCTGAAATCGGCGGTAAGGCCATTAAAAGGGTCAGTTTATCAGGGAGG
CGTTAGCCCCCCATGTTGTTAATCATCAGGCAATATCGTCTTTGTAGCAGGCATAACCGAAGCTAAGCTCTGTTTTCATATAGTG
GCGGGCAAAGTCCCAGGCATCGTGGCCGAAGTCCTCATAATCTGCCAGGACGATTTCGCGGGCTTTGTGCCATTGCTGTACG
GAAGAAAGCGGCAGAACAGGGCAGGGGTGCGACCAGTCAGTGACGGTGTTGCAGATCATATCTGCCAGACGCTCCAGGGA

GCCGTAAACCAGCTCTGTGCGCAGATCCGCCACCATGCGTTGTTTACGCAGTGATGCCAGATAATCAATCTCTTTGGTTATATC
AGAATTTAAGCGGGTCTGGTAATCCATGATGTACTCCTTTGCGCGCCGATACCGGCAATTTTGCGGGCGACGGTGTTGCCTCC
CGATGATTTAATTATCGGTGATTATGCCCTCAAAGTCAATATAAGTACGGAATATGCATGCATAATTTTATATCTTGCAAAGCGTT
CATAGAGTGCCTGAATCGCTTTCTGACAGCCTCAATAAAAAAAGGCGGGGATTCCCGCCTTTTTCTTACAGCTGCTTACGTGG
CTTTTTACGCGTCATATACAACGGTATCGCGCAGTCTACCGCGTACAAAAAGCACGCCAGCGCGCCGCAACCGTACAGAAACG
CAAGCGGCTTATTATCGAAGTAGCTGAAAACCCCTGTCGCCGCACACAGGCCCAGAACAGAGACGCAGGCGCAGGTGATCT
GCACCAGATCCCTGTATCCCGCACGAACGATAAACCAGGGCAGGGCAAGCGCAGCGGCGCTAATAATTAATGCGAGAGGGA
CAAAAACGAGATAGTGATACATGTGAACTCCTTGATGGTTGCCGATACCGGCGATTGTTCGGGCGGCGGTATTGCCACCCGAT
GATTTAATTAGAGGTTTTGCGCGTCCAGGAGATTGACCTGAGCCGGGGTAACGTGAAACTTTTCCCCTTTATGGATCACGTTA
TGCGGGGCGCTAATTTCATCACTGATAAAGCTAACCGGGTAACGTTTTTTACCGCAAATCCGCTCGCTAAACCATGCCACTTTT
GCCGCTGGCCGATCCACTGGATGAACAATCACACCGGCCATGCTGCAACCCGTTGCGGGTTCGTCCAGCGTAATGCTTACCG
GGACTGTATCCCGGTAAAAAAACTTAACCGGCGGCACACCTGCCTGCGTAGCGGCTGCGACAACTGCAAGCCCGATAACCGC
TATCCGATTAATAAGCATTTTATTCCCCTTACTCATGCTGATATCACCTTGCCAGCTGTTACCAGTTTACGAAATTCACTTTCATGA
ATTTCACGCCCATGCTCAAGCGTTGAATACACGTTGCCGATAAGCCAGTTACCAGCCGTTTTTGTCTCGGTATACCACCATGCT
TCTGTCAGAGTAAGCAGCGGTTCGCTATCCTCGCCCTTCTCATAGATCCAGATTTAGTGACGTCTTTACCCGTTTCGTTGCTGC
CCTGAATGGTAGGGTCAAAGGTATGTTCAATCTCTATATCGAAGTAACGCTGAAGGAAGTTAGTAAAGTGCATGACGACTCCT
GTAAGCGCCGATACCGGCAATTTTTCGGGTGGCGGTGTCGCCTCCCGATGATTTAATTATCGTTGATTATGCTTTTAAAGTCAA
TACAGGTACGGAATTTATTTACCTGTTTTTATGCCCGTCAGGGCATGGAAGGCGACCGCGCCGGACTCCACCGGACACCGGC
CGCAAATCGCCGGAAACTGCGGGACTGACCGGAGCAACAGGCCAACCCCCCTCCCTGCTAAGCCATAACCCAGCCCGCCGC
CACGCAGCTGCCGCACGTCCCCCACGGGGGTGCGCAGTGGGCGCCGCGCGCCTGCGCGCGGGTACGGCGGCCCGCCTGCG
GGTCGCGGCGCCGTACTGCGAGTTAGCGGCCGCCGCGCGGCCGGTTACGGGGGACACCGCACCGTCACGGCCAGCGCCCC
ACTGAGCTGCACAATCCACGGATAATGCAGGAGACGAATCATGATAGGAGGCTGAAGGGGAAATGAGCGGCAGCAGGGGA
AGGGGTTGCCAAGCGGAGCGCGGAGGCCGCAGGCCGGAGGCGTCAGTGGCAGCTGCCCGCGTGAGCGGGGCGAGACGC
GTAGCGGCTCGATGCGCAGCACAGCAGAACGGCCCCGGAGGGGTGACGTCCGGGGGTTCGCTTTTAAAGATTTTCGACCAC
ATCAGTAAATCGTAGTGACACCATGAAGCAAAAGTATCGTGCAGACCAGAATCAACAACAGTAACAGCAGACCTTTTTTTCGA
CGTAATAAAACCGGCCCCAAAGCGCCCGCAAGAAGCACACTCAACACGATTTCCGTTATACTAATCGTATTCATAATCTCTCAC
GTTTCCCTTTTTAGAACTCTGCCACACACAGATAAAACCTTATAACAAGCTACAAAACCCGTTATTCAGACGCGGTAATGCCTA
GTTTTTTTGCCAGTATTTCAGATGACAAAGAAACCCTTATCCACTTCCCGGTTACAAGGTGAATGATTGTGGCTTCATGCCCTT
TAACAGAGTGGGATATTAACTCACGCTGAATGATATGCGTCTCTCCATCATTTCCCGTCACGGCAATTAGGCCCTCTGTAGAAC
AGGGGTTGATACTACTAATGTTGGTGTTGTTTTCGGTTTTGTTCAGCATCGCTGATCCTCAAATATCGGTTTGTGTTACGTCTGC
CGCTTTGCGCTGGATAAGCGACTTAAAGAAATCCGACGCCTTCAGAATATCGCTATCCTGGAAGTCGGGAAATGTCGCTTCTG
TCAGCTCCTGCGCCGTACCTGCCCGTATAACGGTTTCATCGCGGATCATCGTTCGCTTAACGCCATCAGCCTGTTTTTTGCTGA
AGGAGTGCCGGAAAATAATCGCGTATTGGCCGCCGCTATCCTGATGAACAGAAGTATCCCAGCGATGATTACCCTGTTTACGAT
AGTGGTGATAGATTTGCTGGCCGTAGCGCTCCTGATCGGAATTACGGTAATCGAACATTAACAGTTCCAAAAGCATTAACCGA
TCTGGCAATTGCCAGGCGGTAGGGTGTTGAAGTGTCGCTAACATAGTTTCCCCTGAGCGTGACAGTCACGATAAGGCGGGCT
TTGCCCGCCTGGTTATCAGTTAATCAATGGCACGATAAATACGATTCTGGCTTTCGTTCTCCAGCGTATTAACGTACTCCCGCAA
CAGGTGATACCGGTTAGCCATCGTTTCGTTCAGTTCGGCTTTGCCTTCTTCATATGCCAGGCCGCAAAAGTAACTGTAGGCATA
CAGACAAACAATAATCCCTACTTCGCGCGCGCTGCATTCACCTTCAAAATAGTTAGGTAACGAGAGCCAAAGAGGTTGGGGC
GCTTCCATAAAAAACGCGCCATTGCTGGCCTGAAGGTATTCCCAATACCCTCCCTGGTAGTCTTTAGCGTAACGATTCAGAAAG
GACTGAATGAAGTGATCTGCGCTGAAGAAAGCGCCACGAAATGCCGCAGGCATGAAGTTCATGCGGGCGTTTTCAGAAATG
TAGCGGGCGGTGATTTCGATAGTTTCCATGATACTTCCTCTTAAAGCCGATACCGGCGATGGTTAAGCGGCAGGCACATCACC
TGCCACTTTTTAATTATCGTACAATGGGGCGTTAAAGTCAATATAAGTACGGATTATATTTACCTAATTTTATGCCCGTCAGAGCA
TGGAAGGCGACCTCGCCGGACTCCACCGGACACCGGGGGCAAATCGCCGGAAACTGCGGGACTGACCGGAGCGACAGGC
CACCCCCCCTCCCTGCTAGCCCGCCGCCACGCGGCCGGTTACAGGGGACACTGAGAAAACAGAAAGCCAACAAACACTATAT
ATAGCGTTCGTTGGCAGCTGAAGCAGCACTACATATAGTAGAGTACCTGTAAAACTTGCCAACCTGACCATAACAGCGATACT
GTATAAGTAAACAGTGATTTGGAAGATCGCTATGAAGGTCGATATTTTTGAAAGCTCCGGCGCCAGCCGGGTACACAGCATCC

CTTTTTATCTGCAAAGAATTTCTGCGGGGTTCCCCAGCCCGGCCCAGGGCTATGAAAAGCAGGAGTTAAACCTGCATGAGTAT
TGTGTTCGTCACCCTTCAGCAACTTACTTCCTACGGGTTTCTGGCTCGTCAATGGAAGATGGCCGCATCCATGATGGTGACGTA
CTGGTTGTGGATCGCTCGCTGACGGCCAGCCACGGCTCAATCGTAGTCGCCTGCATCCATAATGAATTTACCGTGAAGCGGCT
ACTGCTGAGGCCCAGACCCTGCCTGATGCCGATGAACAAAGATTTTCCTGTGTACTACATTGACCCGGATAATGAGAGCGTTG
AAATCTGGGGAGTGGTTACGCATTCCCTTATCGAGCATCCGGTATGTTTGCGCTGATTGATGTCAATGGCATGTACGCCAGCTG
TGAGCAGGCATTTAGGCCAGATCTGGCAAACCGAGCAGTGGCCGTTTTATCCAACAATGACGGCAACATTGTGGCCCGTAAT
TACCTGGCGAAGAAAGCGGGCCTGAAAATGGGCGATCCGTACTTCAAAGTCAGACCCATAATCGAGCGTCATAACATCGCTAT
TTTTAGCTCTAATTACACTCTCTATGCCTCCATGTCGGCCCGGTTCGCGGCCGTAGTTGAGTCCCTTGCAAGCCACGTCGAACA
GTATTCAATCGACGAGCTTTTTGTTGACTGCAAAGGGATAACGGCCGCCATGAGCCTTGACGCTTTCGGGCGCCAACTGCGC
GAGGAAGTCAGGCGACACACAACGCTGGTATGCGGGGTCGGTATTGCCCGTACTAAGACGCTGGCGAAGCTGTGTAACCAC
GCTGCAAAAACATGGCCCGCTACTGGCGGGGTGGTTGCTCTGGACGATGGCGCCAGACTGAAGAAATTAATGAGCATCCTG
CCGGTTGCGGAAGTCTGGGGCGTCGGCCATCGTACAGAGAAAGCACTCGCCACAATGGGGATCAAAACGGTGCTGGATTTA
GCCAGGGCAGATACGCGCCTAATCCGTAAAACATTCGGCGTTGTGCTTGAAAGAACGGTACGGGAGTTGCGCGGCGAGGCT
TGCTTCAGCCTGGAAGAAAACCCTCCTGCGAAGCAGCAGATTGTTGTGTCGCGCTCATTCGGCCAACGCGTAGAAACCCTGA
CGGACATGCAGCAGGCTGTCACCGGATTTGCAGCGCGCGCAGCTGAAAAACTGCGTAATGAGAGGCAATACTGCCGCGTCA
TAAGCGTCTTTATCCGTACCAGTCCTTATTCAGTGCGTGATACACAGTATGCCAATCAGGCAACCGAAAAACTGACGGTGGCA
ACCCAGGACAGCCGCACGATAATTCAGGCAGCACAAGCCGCGCTGGCGCGGATCTGGCGGGAAGATATTGCGTATGCAAAA
GCAGGGGTCATGCTGGCAGATTTTAGCGGGAAGGAGGCCCAGCTTGATTTATTCGACTCTGCTACGCCTTCAGCTGGCAGCG
AGGCTTTAATGGCTGTTCTTGATGGTATAAACCGGCGTGGAAAGAGCCAGCTTTTTTTTGCAGGCCAGGGCATCGATAACTCC
TTTGCCATGCGTCGTCAGATGTTGTCACCTGATTACACGACAGACTGGCGCTCAATACCAATAGCCACCATCAAATAATTACCG
GCGCCGTACACGGGCCGGTTAACCCCTCAACCGGCCGAAACAAGTTTCGGCACGGTTTCGCGGTTTTCGGTAAAAGCCGTT
TCCTCTGTATAAAAGATCAGCTAAATTATGTGTATTGCACAATACATATATGTGAGGTTAGCAGTGAATTTGCCTACGCCCGAAA
CCTACGATGAACTTCAGAGAGCCTACGATTTTTTCAATGATAAGCTATTCAGCAACGAGCTGCCGCCATGCCTGATAACGTTGC
AGCGTGAGAAGCGAACGTATGGCTATTGTTCCTTTAAGCGTTTCGTCGGCCGTGAGAGTGGGTACACGGTAGACGAGATCGC
TATGAATCCGGTGTATTTCTCGATCAGAACCATAAAGGCCACGCTTTCAACACTGGTGCATGAGATGGTTCATCAGTGGCAATT
CCATTTTGGCGAGCCTGGCCGCCGTGGCTATCACAACAAACAGTGGGCGGCCCGGATGGAACGGGTAGGACTAATGCCTTC
TGATACCGGCGAACCGGGAGGCAGGAAAGTGGGCCAGAGCATGACCCATTATATTATTGCCGGTGGCCCTTTCGATATGGCC
TGTGATGAACTGCTGACAGGCCATTTCCGGCTTTCCTGGATGGACAGGTTTCCGCCTTACCAGCCTAAGCCTGGCGCTGTGCT
AAGCCCTACAGGAAAAGGCTATATTGACGACGAGGAAGATGATAGCGAACACGAACAGGAGGTGGAGGAAGGGCGCGACC
CGGTTGAACTCGACGACGAGATCATAGAGGCCATGCGATTTGTAACCCCACCGCCTGAAGCACCGGTGAACAAAACAAACC
GGGAAAAGTACAGCTGCCCGGTGTGTCATATCAATCTCTGGGGTAAACCGGGGATAGTGGTTTACTGTGGTGGCGAGCACTG
TAATAAAGCCGCGTTAGTAGTCTTAAAATAAAGTCCTTTCGGACTTTATTTTTTTTCCATTTCCGAGGTCGTGATGTTATTAATGC
TGTACTTCGCGGCTTCTTTTAAAACAGTTTCAGCAAGGCTTGCTGGTATCCAGACCTGAACTAATTTTAATGGTTCGCCGTTCT
CGGCTTTAAGAGTGGTGTTCTGGTACAAATCCCAGATTCGCTTAACGGTGCTGGAAATGTTTTGCTTGGAACGGCCTACTCGC
ATGGCTACGTCTGATGATTTCTCACCTTTGACAAGCACGGAATAGCCAATATCTGTTGTGATGTGTGCAAAGGAAGCCATTTGC
GGCAGCAGCTGTTTCCATTCTGTTTCTGAAATTCTGTTTTTCTGAGCCATCTGTGGCGCCTCCGTAGTTTTGGTTACAGAAAGG
ATATACTCAGAATAAACAGGGGTCAATACAAGTACGATTTTTATAAACTTTATTTTATTTGAGGGTGAGGCCCGGTGCGGCAGC
AGCGCGGGCCTCGATGGTGCCGCGAAGGTGCTGGCGCCATGCTCGGATTAAAACATGAACCGTGAAGAACTGCGAAACTTG
TTTTCGCGGTTCTGAGGGGTTGACCGAGCCGCGAAGCGGCGCTGGTAAGCGATGATATGCACATATCCACAGGCATATTTTTA
AAAGGTATTTTATAGATTTTTTATCTTTTTAAAGTCTTTTAGAGCTATATAACTCATTGATTTAAAATCATAAATAAGTGTTATCTCT
GGGAATCCGCCCACCTTGTTATGGGAATTGGCCCACCTTACTATGGGAAACAGCCCACCTTACTATGGGAATTAGCCCACCTT
GTTATG

>pQEB1_inv3_Tn5403

GGAATTGGCCCACCTTAGACGAAACTGTAAAAAATGTATTTACTTGTTTGAACTTTGTGGTAGTGTGGAGAGTAATTTTTAACC
CACAAAGGCAAGGCGCATGGATAAGTTGCTGAACAAAAAGATAAAAGTTAAGCAGTCTAACGAGCTTACCGAAGCTGCTTAC
TACCTCTTCGCTAAAAGCAAAGCGCGTTCTCTGGTTATGTCTTATGCAGACGTATTTCACAGCTTCAGTAAGCGAAGATGATGA
TGAGATGGCTGTACTCGGTGACTCTACTTTCAAAGTAAAGGTGGCTGACTATCAGCAAATTTTTCAGGTAAGCCGTAACCAGG
CTATCAAGGATGTTAAAGAAGGCGTGTTTGAGTTAAGCCGTTCTGCGGTAATCTTTTACCCGAAAGAGGGGCGTTTTGACTG
CGTCGCGCGCCCCTGGCTAACAGAGGCTGGCAGCCGATCAGCTCGTGGTATCTGGGAAATCGAATTTAACCATAAACTCCTGC
GGTACATTTACGGCCTGACGAACCAGTTCACCACCTACTCGCTCCGCGATTGTGGCAGTCTTCGAAATCCCCGGACGATCCGC
CTTTATGAAAGTCTTGCTCAATTCAAATCTTCAGGCTTATGGGTTACTACTCATGCTTGGTTAAATGACCGTTTCCTTTTGCCGG
AATCCCAACAGAAGAACTTGGCAGAGTTGAAACGATCTTTCCTTGATCCTGCACTCAAGCAGATAAATGAGAAAACACCTTTA
CTTGCTAAGTATAGTATTGATGATTCAGGAAAATTTCTGTTCTCAATAATTGATAAGCAAAATCCCGTCTGACATAAATCAGCAC
ACATGAGCCTGTCATTTGACAAATTTTTGTCATGAAGATGGGCGAATTTCCACACAGCACCGGCGCCCGGCAAGATGGGCGG
ATTCCCACACGACAGCGGCGCCCGGCAAGATGGGCGGATTTCCACACTACAGCGGCGCCCGGCAAGATGGGCGGATTTCCA
CACGGCAGCGGCGCCCGGCAAGGTGGGCGGATTTCCACACGGCAGCGGCGCCCGGCAAGGTGGGCGGATTCTCACGCGG
CAGCGGCGCCCGGCAAGATGGGCGGATTTCCACACGGCAGCGGCGCCCGGCAAGGTGGGCGGATTCTCACGCGGCAGCG
GCGCCCGGCAAGGTGGGCCGATTCCCACGCGGCAGCGGCGCCCGGCAAGGTGGGCCGATTCCCACGCGGCAGCGGCGCC
CGGCAAGGTGGGCCGATTCCCACGCGGCAGCGGCGCCCGGTAAGGTGGGCGGATTTCCACACGGCTGCCGCGCCCGGCAA
GGTGGGCGGATTTCCACACGGCAGCGGCGCCCGGCAAGGTGGGCGGATTCTCACGCGGCAGCGGCGCCCGGCAAGATGG
GCGGATTTCCACACGGCAGCGGCGCCCGGCAAGGTGGGCGGATTCTCACGCGGCAGCGGCGCCCGGCAAGATGGGCGGA
TTTCCACACGGCAGCGGCGCCCGGCAAGGTGGGCGGATTCCCACACGGCAGCCTCGCCCGGCAAGGTGGGCGGATTCCCA
CACGGCAGCCTCGCCCGGCAAGGTGGGCCGATTCCCACGCGGCAGCCTCGCCCGGCAAGGTGGGCGGATTCCCACACGGC
ACCGGCGTGCGGCAAGGTGGCGGATTCCCACACGGCACCGGCGCGCGGCAAGGTGGGCCGATTCCCACACGGCACCGGC
GCCCGGCAAGGTGGGCCGATTCCCACACGGCAGCCGCGCCCGGCAAGGTGGGCCGATTCCCACGCGGCAGCCTCGCCCGG
CAAGGTGGGCCGATTCCCACGCGGCAGCCTCGCCCGGCAAGGTGGGCCGATTCCCACACGGCAGCGGCGCCCGGCAAGGT
GGGCGGATTTCCACACGGCAGCGGCGCGGGGCCAGTGGGATTCAGGAGAATAGGTGTTTACGAATGCCCTGACAGGCGTA
AAAAAACCGCTTGGCGGCGGCCTCATAAAGCAGAAAACCCGCTCAAGGCGGGTTATCTGCTCTGTAGCCTGTGATGCTTCGC
GGGCATCCGGCATACAGCGAGGTGAAATTCTTCTTTTGGCATGTTAATTATACGTCTAACGCGGCATATGATCAAACCTGTATTA
AATAAGCCACTGTACCGTTTATAATGCTCTCAGATCAAAGAGGTAAAGCCCGTTTAGCCGCCTGTGTGATGAGCCAGTTCAGA
CTCTTCAAAATCGAATTTGGTACTAAACAGGACCCGAACCGTGGGCAAGCACACGGCAACGGTATAGCCCTCTTCCGGTTTTG
CACCCGGAAGCCTGGGCGGCAGCGTGGTGAAATTCTTCTTTTGGTTAAGTGAATGGCATAACCGGATGGGCGGATCAAGAG
GAAAGGGGATTGCCTAGTAACCTACGCGCCACAGAGATGGAGGTGCGGGGAATGATTGAGCTGATTATCGCTATTCTGACCT
TAATTGCGGCTGTATTGCAGTTGATCAACTGGTTCCTTTAATGGTGCCGGAGTCTGTGAAGGTGAAAGCCTGAACGGGCAAA
ACTGAAAGGTTTATAGCCGTCCTTCGGGGCGGCTTCTTTTCGGCAAAATTAGGGTTTTACCGAATAATGCAGAGTTTTAAGGT
GAGAATTTGCAGACTTGGCGTTTTACCGAACATAGATACTCCCTAGGCTGATAGGTGCATTAGTTATCACCCTACCTGAACATAT
TGTAAAAGATGTCAGTCTCCAGTGACTTGTGTACTATCAACTGACAAGACTCTTACACGCAACGCAGGGGGATGGAGTTTTAT
GCTTAGAAAAATAATCAGGGGTAGCGGATTCACTCAGTCAGAAGAAAAACTGATAGAGTTCGCTGATGATGCTTTGGTCTTTG
GTCTTATCCTAATGTTTATAGCGATGAGGGTTACTCTAAAAATAAAATTGGGAAAGAAGTTAGTGACTTATTAGTTATTTTTGAT
AAAGATATAATAATTTTTTCCGATAAAGCTATTACATACAATAAAACAAAGATCCTAAGGTTGCATGGCAGAGATGGTTTAAAA
AATCAGTCATACAGTCTTGCACACAGTTATTTGGCGCAGAGAAGTTTATAAAAGATCATCCCGAAAGACTTTTTGTTGACAAA
GAATGCTCAGTTAACCTCCCCATTAAAATAGATAATTCTTTTAATTTTCATTTGGTGGCCGTCACTAATAATATTTCAGATCCGCG
ATCCTTATGACAAAATAGAAAAAGGCAGCTCTGCTACTTTAGTTAACATATTTCCTTTAAACGCCCATCAATGTCTAGAAAATCC
ATTTTGTGTCGGAGACGTTTATCCTGATAAGACTTTTGTCCATATACTTGATGAGACTGCCCTAAAACTACTGTTAACCGAGTTA
AACACAGCAACTGATTTCATTGGCTACCTTAACGAAAAAGAGAGGGTTGTAAGAAAAAGAACATTATTGGTCAGCGCTGGGG
AAGAAGAGACTCTGCTGCTTACATTATGGGTGATAAAACCATAATATCAAAAGAAATTATTGGAAACGATCAAGGGATGACCA

TACCGGAAGGTGAATGGAAAAACTATAAAACCACTTTCAATTATCAATATCAGCTCTCAATGAAAAAGGGTAGCGTTTTCTGG
GATAACCTAATCCACAACTTCTCGACAAGTATATTGTCAGCTAACGTTGGTTTTTTTAGTGAAATTGAATTTTCTACACATGAAT
GAGGTGTTAGAGAATTAGCCAAAGAAAGTAGGCAATCTAAATATTACCTTTCAAAGAACTTTAAAGAGAAATTAAAAACAACT
CAGCCTCATCTAAGAACGTCAAGAATGGTCGAATCAATCGATGAGCCTGGAAAGTTTTACTTATTCCTTTTTTTTCCTAACGATA
GCAAGTTGAGTTACTCTGATTACAGAATTCAACGTATATCTTATATAAATGCTTATGCTGAGGTTGCCTTTAATAAATACAGACAT
ATTAAAAAATTAATTACTATTGCAACAGAGCCGCAAAATACAGAAGGAAAATCTGAAGACCTAATATATAAGCATATCCCAGAG
AAATTTACAAAAAGCAAATGAAAAAGCCAAAAGATTATCAAGAGAATACAAAATACTAAGTGATTTTTTACCTACTAAAACGA
CAAAGAGCGATAACTTTAAATCAGTTATATCAAAAGGTGAAAAAATAGGGCGGAATACACCTTGTCCATGTGGCTCCGGTGTT
AAATTTAAAAAGTGCCATGGTGCGAATAATTAGCATTATTGTATGTATAACGGTAATGGCGCGGCAGAGGAACCGGCGCGTTC
TGCCCTAGTGTTGGCCTGCGGGTTCCCCCGCACCCGCTGTATGTAGTATCGGCAGCATCTGAGAAAACCACTACATGTAGCTAT
CAGCGCCACAACGGCGCGGGGACGAGTGCGGTTCGGAAAAATTGGGGTTTTACCGAATCCGGCAAAAGATTGCTTCCTATA
ACGTCCGCTTCTGGCACACAGCAGCCGTTAAGATGTAAGGCTGCACGCCAACTAAATCTAATGGGACAGATTTAGTTGGTGAT
GGTCAAGTAATCTGCAAACGGTCACCAAGTAAAATGCAAATGGGTAGTCAAGTCCGATGCAATTACGCACCCGGCAAGGTGG
GCCGATTCCCACACCACAGCAGCGCCCGGCAAGGTGGGCGGATTTCCACACGGCAGCGGCGCCCGGCAAGGTGGGCCTAT
TCCCACACGGCAGCGGCGCCCGGCAAGGAGGGCCGATTCCCACACAGCACCGGCGCGCGGCAAGGTGGGCCGAGTCCCA
CACCGACAGCAGCGCCCGGCAAGGTGGGCGGATCTCCACACAGCACCGGCGCCCGGCAAGGTGGGCCGATTCCCACACGG
CAGCGGCGCCCGGCAAGGTGGGCCGATTCCCACACGGCAGCGGCGCCCGGCAAGATCGGCGGATTTCCACACAGCACCGG
CGCCCGGCAAGGTGGGCCGATTCCCACACGGCAGCGGCGCCCGGCAAGGTGGGCGGATTCCCACACGGCAGCGGCGCCC
GGCAAGATGGGCGGATTCCCACACGGCAGCGGCGCCCGGCAAGATGGGCGGATTTCCACACGGCAGCGGCGCCCGGCAA
GATGGGCGGATTCCCACACGGCAGCGGCGCCCGGCAAGATGGGCGGATTTCCACACGGCAGCGGCGCCCGGCAAGGTGG
GCCGATTCCCACACGACAGCGGCGCCCGGCAAGGTGGGCCGATTCCCACACGACAGCGGCGCCCGGCAAGATGGGCGGAT
TCCCATATCGACATGTATGTAGCTTGTGTTATCCGTGGATTGTGCAGCTCAGCGGGTCGCTTGTCGTATGGCGTAGTGTCCCCC
GTAACCGGCCGCGTGCGGCCGCTAACGCGCAGTACGGCGCCGCGACCCGAAGGCGGGCCGCCGTTCCCGCGCGCAGGCGC
GCGGCGCCCACTGCGCACCCCCGTGGGGGACGTGCGGCAGCTGTGTGGCGGTGAGCGGGATTAGGGCTTTGCAGGGAGG
GGCTGGGTCGGGCGATACGTTCAGCATTGCGGTTTCCGGCGATTTGCGGCCGGTGCCCGTTTAACTCCGGCGTGGTCGCCTT
CCATGCCCTGACGGCATAAGAAAATAAAACCGCCATGCTGCGGTCATTCATGATTTTGTGGTGTAGCGATAAATAGTCATGCGA
GAAACGTTGAAGCGCTTAGCAACTGCACCAACTGTCATTTCAGGATCAGCAAGTAAGATTCTAATTTGTTTAACATCTTCTTCA
GAAAGTGACGGTTTTCTCCCTCCCACCGGCCCCTTGCGCGTGCAGCTGCAAGGCCTGAGCGCGTCTTCTACAGATTGCGGTC
GCGTTCAAAGCTAGAGAATATCGCCATCAGATGAGTATAGATTTCCCCGATAACTGGCGCATTTGTGTCTATTCTGTCCTTGATG
GCATGCAAGTTATTCCGCGTTTCTTCAGGTCGTCGAGTAAAGTAATGACTTGACCGAATGAACCACCGAGCCGATCTAGTGCC
CAAACTACTAGGGTATCTCCCTCGCGCAATGCTTTCAGGCAGTTCTCCAGTTCCAGCGCACCTTTTTTGTCGCGCTTTGGGCC
GCTACGTGAGGTCTGATCCTGATAGATTTGCTCACATCCAGCTTTTGTTAGTTCGTCAACCTGGTGCGCCACATCCTGAAGATG
CGTAGATTTACGTGCATAGCCGATTTTCATTCTTTTCTCGCTAATTAGTTATGGGGTTATTGTTATGTTGATACAGTAACGAGTTT
TGTTACATGAGGGGAGTCATTTTTCGGGAGAAGTCAGGACTTTTCAAGACTGTCACAAAAACCATCGTTTTTGATACATTAAT
TTAACCAATAGGTTGCAGATCAAATCGTCTGTAACAGCCTTTCTGGCTGTTTGTATATAATCATGAAAAAATGGTGAGTAGAGT
TTCAGGGTAACAGGGGATGCTTATGTCGGTTTTCCACAACTGGCTACTTGAGATCGCATGTGAGAATTACTTCGTCTACATCAA
ACGCCTTTCCGCCAACGATACCGGCGCAACAGGTGGTCACCAGGTAGGGCTTTATATCCCTTCAGGTATCGTTGAAAAACTCT
TTCCGTCTATCAACCATACCCGTGAACTGAACCCTTCGGTTTTTCTCACCGCACATGTGTCATCGCATGATTGCCCTGACAGCG
AAGCCCGGGCAATTTATTATAACAGCCGTCATTTTGGTAAAACCCGGAATGAAAAAAGGATTACCCGCTGGGGTAGAGGCAG
CCCACTTCAGAATCCTGAAAATACAGGGGCTCTGACGCTCCTGGCTTTCAAGCTTGATGAGCAAGGGGGGGACTGTAAGGA
AGTAAATATTTGGGTATGCGCCAGCACTGATGAAGAGGACGTCATTGAGACCGCTATTGGTGAAGTTATACCCGGAGCGCTTA
TATCCGGCCCCGCAGGACAGATTCTAGGCGGACTATCTCTACAGCAAGCGCCAGTAAATCATAAATATATTCTACCTGAAGACT
GGCACCTGCGCTTTCCGTCGGGAAGTGAAATTATTCAGTATGCAGCCAGCCATTATGTGAAAAATTCCCTTGATCCGGATGAG
CAACTTCTTGACCGCCGGCGCGTGGAGTACGACATATTTCTATTGGTTGAGGAACTGCATGTTCTGGATATCATCCGGAAAGG
ATTTGGCTCTGTGGATGAATTTATTGGGCACTGTTGCAAATAGTCGGTGGTGATAAACTTATCATCCCCTTTTGCTGATGGAGC
TGCACATGAACCCATTCAAAGGCCGGCATTTTCAGCGTGACATCATTCTGTGGGCCGTACGCTGGTACTGCAAATACGGCATC

AGTTACCGTGAGCTGCAGGAGATGCTGGCTGAACGCGGAGTGAATGTCGATCACTCCACGATTTACCGCTGGGTTCAGCGTT
ATGCGCCTGAAATGGAAAAACGGCTGCGCTGGTACTGGCGTAACCCTTCCGATCTTTGCCCGTGGCACATGGATGAAACCTA
CGTGAAGGTCAATGGCCGCTGGGCGTATCTGTACCGGGCCGTCGACAGCCGGGGCCGCACTGTCGATTTTTATCTCTCCTCCC
GTCGTAACAGCAAAGCTGCATACCGGTTTCTGGGTAAAATCCTCAACAACGTGAAGAAGTGGCAGATCCCGCGATTCATCAA
CACGGATAAAGCGCCCGCCTATGGTCGCGCGCTTGCTCTGCTCAAACGCGAAGGCCGGTGCCCGTCTGACGTTGAACACCGA
CAGATTAAGTACCGGAACAACGTGATTGAATGCGATCATGGCAAACTGAAACGGATAATCGGCGCCACGCTGGGATTTAAATC
CATGAAGACGGCTTACGCCACCATCAAAGGTATTGAGGTGATGCGTGCACTACGCAAAGGCCAGGCCTCAGCATTTTATTATG
GTGATCCCCTGGGCGAAATGCGCCTGGTAAGCAGAGTTTTTGAAATGTAAGGCCTTTGAATAAGACAAAAGGCTGCCTCATC
GCTAACTTTGCAACAGTGCCGTACCGACGGTGATATGGGGCAAATGGTGGTCACCATCCTGTCGGCTGTGGCACAGGCTGAA
CGCCGGAGGATCCTAGAACGCACGAATGAGGGCCGACAGGAAGCAAAGCTGAAAGGAATCAAATTTGGCCGCAGGCGTAC
CGTGGACAGGAACGTCGTGCTGACGCTTCATCAGAAGGGCACTGGTGCAACGGAAATTGCTCATCAGCTCAGTATTGCCCGC
TCCACGGTTTATAAAATTCTTGAAGACGAAAGGGCCTCGTGATACGCCTATTTTTATAGGTTAATGTCATGATAATAATGGTTTC
TTAGACGTCAGGTGGCACTTTTCGGGGAAATGTGCGCGGAACCCCTATTTGTTTATTTTTCTAAATACATTCAAATATGTATCCG
CTCATGAGACAATAACCCTGGTAAATGCTTCAATAATATTGAAAAAGGAAGAGTATGAGTATTCAACATTTCCGTGTCGCCCTTA
TTCCCTTTTTTGCGGCATTTTGCCTTCCTGTTTTTGCTCACCCAGAAACGCTGGTGAAAGTAAAAGATGCTGAAGATCAGTTG
GGTGCACGAGTGGGTTACATCGAACTGGATCTCAACAGCGGTAAGATCCTTGAGAGTTTTCGCCCCGAAGAACGTTTTCCAA
TGATGAGCACTTTTAAAGTTCTGCTATGTGGTGCGGTATTATCCCGTGTTGACGCCGGGCAAGAGCAACTCGGTCGCCGCATA
CACTATTCTCAGAATGACTTGGTTGAGTACTCACCAGTCACAGAAAAGCATCTTACGGATGGCATGACAGTAAGAGAATTATG
CAGTGCTGCCATAACCATGAGTGATAACACTGCTGCCAACTTACTTCTGACAACGATCGGAGGACCGAAGGAGCTAACCGCTT
TTTTGCACAACATGGGGGATCATGTAACTCGCCTTGATCGTTGGGAACCGGAGCTGAATGAAGCCATACCAAACGACGAGCGT
GACACCACGATGCCTGCAGCAATGGCAACAACGTTGCGCAAACTATTAACTGGCGAACTACTTACTCTAGCTTCCCGGCAACA
ATTAATAGACTGGATGGAGGCGGATAAAGTTGCAGGACCACTTCTGCGCTCGGCCCTTCCGGCTGGCTGGTTTATTGCTGATA
AATCTGGAGCCGGTGAGCGTGGGTCTCGCGGTATCATTGCAGCACTGGGGCCAGATGGTAAGCCCTCCCGTATCGTAGTTATC
TACACGACGGGGAGTCAGGCAACTATGGATGAACGAAATAGACAGATCGCTGAGATAGGTGCCTCACTGATTAAGCATTGGT
AACTGTCAGACCAAGTTTACTCATATATACTTTAGATTGATTTAAAACTTCATTTTTAATTTAAAAGGATCTAGGTGAAGATCCTT
TTTGATAATCTCATGACCAAAATCCCTTAACGTGAGTTTTCGTTCCACTGAGCGTCAGACCCCGTGTTGACACCGGCGTACCCT
CGGTGCTATCTTCGCGCCCCAATAGTCGGGGCTTGGCCAGGACTTCCTGAGGCCGTCCGTAACTCCACCTTCAAACAAGGAA
TATTGTTGATGTCACTGTATCGCCGTCTAGTTCTGCTGTCTTGTCTCTCATGGCCGCTGGCTGGCTTTTCTGCCACCGCGCTGAC
CAACCTCGTCGCGGAACCATTCGCTAAACTCGAACAGGACTTTGGCGGCTCCATCGGTGTGTACGCGATGGATACCGGCTCA
GGCGCAACTGTAAGTTACCGCGCTGAGGAGCGCTTCCCACTGTGCAGCTCATTCAAGGGCTTTCTTGCTGCCGCTGTGCTGG
CTCGCAGCCAGCAGCAGGCCGGCTTGCTGGACACACCCATCCGTTACGGCAAAAATGCGCTGGTTCCGTGGTCACCCATCTC
GGAAAAATATCTGACAACAGGCATGACGGTGGCGGAGCTGTCCGCGGCCGCCGTGCAATACAGTGATAACGCCGCCGCCAA
TTTGTTGCTGAAGGAGTTGGGCGGCCCGGCCGGGCTGACGGCCTTCATGCGCTCTATCGGCGATACCACGTTCCGTCTGGAC
CGCTGGGAGCTGGAGCTGAACTCCGCCATCCGGCGGCGATGCGCGCGATACCTCATCGCCGCGCGCCGTGACGGAAAGCTT
ACAAAAACTGACACTGGGCTCTGCACTGGCTGCGCCGCAGCGGCAGCAGTTTGTTGATTGGCTAAAGGGAAACACGACCGG
CAACCACCGCATCCGCGCGGCGGTGCCGGCAGACTGGGCAGTCGGAGACAAAACCGGAACCTGCGGAGTGTATGGCACGG
CAAATGACTATGCCGTCGTCTGGCCCACTGGGCGCGCACCTATTGTGTTGGCCGTCTACACCCGGGCGCCTAACAAGGATGAC
AAGCACAGCGAGGCCGTCATCGCCGCTGCGGCTAGACTCGCGCTCGAGGGATTGGGCGTCAACGGGCAGTAAGGCTCTGA
AATCATCTATTGGCCCACCACCGCCGCCCTTGCGGGCGGCATGGATTACCAACCACTGTCACATTTAGGCTAGGAGTCTGCGC
GGCAGAGCCGTGTGACCGGTTTTCTGTAGAGCACTGACGATGGCGGCGGCGCTCTCTGCAATTGGCAAGGCGTCGGCGCCA
AGGATACCAATCTTGCGGCGCGCGGCGTGTTATGACGACTGGGGTGCATTTGAGCCGCCCCATTTAACCTTCGCCCTCACAGA
TACGCCATTCGCCTCAGATTTAGCGCCATGCAGACGAGCTTCCACTCGGCTTGCACCTTGTCCAGGCCCCTCATGCTGAACTG
ACGCAATCCCATCACCGCCTTGATCCAACGATTCGGAGCCTCCACGATCGACTTGCGCCGGCGGTAAGCTGCATCGCCTTGCT
CCGTTTTCAATTTCGCCGCAATCGCCGCCGTATGCGGATGGGTCTTGGCATTGACCTTGGCATCTTCACGTCCCTCGCGGCCG
AGGGCAACGATGACATCGCCGTGGTGATCGGCGACCTTTGCCAGAACAGCCTCACTACGGAATCCCGCATCCGCCAGCGTCT
GGGCCGGCATTTCTCCGGTGTTGGCCTGAACTGCTGCCAGCATGCCCAGCAGCGCCTGACTGTCCGCGGCGCAGTTGGTCA

ACTCCGCCGCCACGATGATCTGGTGCTCGGCATCGACCGCTGTGTACCCGTTGTAGCTCTGCTCGGAGCCACCACCGGCGTGT
TTCATGATCCGGCTGTCCGGATCGGTGAAGCTTTCCTGATCACGGTCATCCGGCACACCAAACTCGCGTTTGTACGAGCCACC
GCCCTTGTCCGAGCCATCCGGATGGCGAGGCCGGCGGCCATCGTCTTCGCTGCGCCCCCGGGCCTGGTCCGCTTCACGCTGG
CGCGCTTCCAGGGGTCGGTTCCGGCTGAGGGCGAAATGACACCCTAAGCGTTAGCTCTGTGTCGTTGCACGATGTCAGCGAC
GGTATTCTTGCTGATACCGAGCTCGCGTGCGATCCAGCGATAGCTGCGTCCCTCGGCCCTCATCGCAACCACCTTAGGCAAAA
GTCGGTCTGATTTTGGTCGCACTCCGGCCTGACGACCAAGCCTCTTACCACGTGCCTTCGCAACAGCAAGGCCTGACTTGAC
CCGCTCGCTGATGAGATCCCGCTCAAACTCCGCAATGCCGGAAAGAAACGTCGCCAGCATTCGTCCATACGGCGACGAAAGA
TCGAACGCCATTCCATTCATGGCTATCACGGAAACCTTCCAGTTCTCCAGTTCACGTAGCGTATTGAGCAGATCGAGCGTCGA
GCGCCCCCACCGGGAAAGCTCAGTGACCAGGATTGCATCAATTTGTCTGGACTGGGCAAGCGCCAGGACTTTCTTTCGCTCG
GCCCGGTCGAGTTTAGTTCCTGAACCTGTTTCCTTAAATATTCCCACCACGTCGTAGCCGGCACGGCCGGCGAAGGCTCGCA
GATCAAATTCCTGGCGTTCACAAGACTGATCCGCTGTTGAAACCCGGCAGTAAATGGCGGCACGATGTCCCAATTGAACCCTC
CTGGATTTTTGTATCGGAACGCCCTGATTTATATGGGCTGGCTGTTGTCCAAAACAGACTATACTTCAAAAGGGACGAATTTGT
ATGTCACGACGCCATATTTTCACCGAACGGCAGCGAGCAGCGCTGTTCGATCTGCCCACGGACGAACTGTCGCTACTGAAGT
TCTACACGCTGGGCGATGATGACCTGGAAAACATTAGGCAGCGCCGCAGACCGGAAAACAGGATTGGCTTTGCCCTGCAAC
TTTGTGCCTTACGATATCCGGGCCGTGCACTGGCTCCTGGTGAGATGATCCCGCGTGAAGTCCTTTCCTTCGTCGGTGCTCAG
CTTGGAGTTCCGGCTGATGCGCTTCTCACTTATGCCACACGGCGCCAAACCCGTCAGCAGCACATGGACACGCTGCGCGAAA
TTTACGGCTACAAGACCTTCACGGGCCGTGGTGCCCGTGATCTGCGGGAGTGGACTTTCGGCCAGGCCGAAGATGCCAGAT
CAAACGAGGATCTTGCTCATCGTTTTATTGTGCGGTGTCGGGAAACTTCCACCATTCTGCCCGCAGTATCGACAATCGAGCGC
TTGTGCGCGGATGCTCTGGTCGCCGCTGAGCGGCGGATTGAAACGCGGATTGTGGAAAATTTAACAGCGGATGTTCGCGATC
ACCTGGACAAACTTCTGAGTGAAATGCTCGCCGGCAATATCAGTCGTTTCATCTGGCTTCGCAACTTCGAGGTTGGTAACAAC
TCGGCTGCTGCTAACCGTTTGCTCGACAGGCTCGAATTTCTGCGTACCCTGAATATCAATCATAGTGCTTTGGCCAGCATACCT
GCCCATCGCATTGCCCGGCTGCGTCGGCAGGGTGAACGCTACTTCACCGACGGTTTGCGTGACATCACTTCGGACCGCCGCT
GGGCGATCCTTGCCGTCTGTGTTGTGGAGTGGGAAGCGGCGATTGCTGATGCCATAGTCGAAACCCATGACAGGATCGTAGG
AAAAACCTGGCGGGAAGCGAAGCGCCAGCATGACGAAACAATTTCCGGCTCTAAAGCCACACTCACGGATACGATCCGTAC
CTTCACCGCGCTGGGAGCTTCGTTGCTTGAGGCCCGCAGTGACGGAACCCCGCTGGAGATGGCTGTCGCCAGTTCGGTTGC
ATGGGACCGGCTCGCTCAACTGGTAGCGACAGGGACTCAACTCAGCAACACGCTAGCCGATGAGCCTCTTGCATATGTCGGG
CAGGGATACCATCGCTTTCGTCGTTATGCGCCCCGCATGTTGCGCTGTCTGAAGCTCGAAGCCGCGCCGGTCGCCGGACCATT
GGTAGCAGCAGCTTTGTCGATCGGAGAGATGAAAGGTGTTGCATCGCCAGAAAGGCGTTTCCTGCGGCCCAGCTCCAAATG
GAACCGTCATTTACGAGCTCAGGAAAAAGGAGATACCCGTCTTTGGGAAGTGGCGGTACTCTTTCACCTCCGGGATGCTTTT
CGTTCCGGAGATGTCTGGCTCGCTCATTCGCGCCGCTATGGTGACCTCAAGCAGGTACTGGTGCCGATGATCGCGGCGCAGG
AAAATGCAAAACTGGCCGTGCCTTCCAACCCACAGGATTGGCTGGCAGACAGAAAGGCGCGACTCACGATCGCTCTTAAGC
GGCTGGCCCGGGCTGCCCGTAACGGCACTATTCCGCACGGTAGCATAGAAGATGGAACGTTGCGGATCGACAGGTTGACAG
CAGACGTGCCGGATGGTGCCGAGGCACTCATACTGGATCTGTATCGCCGAATGCCGTCCGTTCGGATTACCGACATGCTGCTT
GAAGTTGATGCAGCCCTTGGTTTCACAGATGCGTTTACCCATCTGAGAACCGGGGCTCCATGTCGCGACCGGATCGGTCTGC
TCAACGTCCTGCTCGCTGAAGGGCTCAATCTGGGCCTGCGTAAGATGGCGGAAGCTACAAACACGCATGATTACTGGCAGCT
CTCACGCCTTGCCCGCTGGCATGTTGAAAGCGAAGCCATGAACCAGGCATTGGCAATTGTGGTGGCCGCGCAGGGTAAACT
GCCGATGTCACGCGTCTGGGGGATGGGCACGTCAGCATCGAGCGATGGTCAGTTTTTCCCGACAGCGCGGCATGGCGAAGC
CATGAACATGGTCAATGCCAAATATGGTTCTGTTCCCGGCCTCAAAGCGTATACTCACGTAAGCGACCAGTTCGCGCCATTCGC
TTGTCAGTCGATCCCGGCGACCGTGAGCGAGGCACCGTATATTCTCGATGGACTACTGATGAACGAGGTCGGTCGCCATGTTC
GCGAACAGTATGCCGATACAGCAGGATTCACCGACCATTTGTTCGGAGCCAGTAGCCTGCTCGGCTACAATCTCGTTCTGCGA
ATCAGGGATCTGCCATCGAAGCGGTTGTACGTATTTAATCCCGATACGACCCCCAGGGAGTTACGCAAGTTGGTAGGTGGAA
AAGCCCGGGAGGATCTTATCGTTGCGAACTGGCCTGATATTTTCCGTTGTGCCGCGACGATGACCGCTGGCAAAATCAGGCC
CAGCCAACTCCTGCGCAAGCTCGCTTCTTACCCACGACAAAACAACCTTGCAGTTGCGCTTCGTGAAGTTGGTCGTATTGAAC
GGACCCTTTTCATTATTGAGTGGATCCTGGATACGGACATGCAGCGGCGTGCTCAGATCGGTCTTAACAAGGGAGAGGCCCA
CCATGCGCTCAAAAATGCGCTCCGTATCGGGAGGCAGGGGGAAATTCGCGATCGCACGACAGAGGGGCAGCACTACCGAAT
CGCTGGGCTCAATTTATTGACTGCGGTGATCATTTACTGGAATACCGTCCATCTTGGTCATGCCGTCACGGAGCGGCGGAACG

AAGGGTTGGATGTTCCCCCTGAATTTCTTCCCCACATATCCCCATTGGGCTGGGCGCACATTCTACTGACTGGCGAATATCTTT
GGCCCAAGGAACCGAAAGCTTAGGGTGTCATTTCGCCCTCAGCCGGAACCGACCCCTTCCAGGCGCGCCTTTGCCGCCTGG
ATCGCCTCCAGGCGCTTCTCGCGGCGAGAAATCTCGGCAGGAATGTCCAGCTCCGGCTCGTTACGCTCCTGGTCGTCGGTAG
CCTTGGCGCGATCAAGCAGCGCCTTGATCTCGCAATGCAATTCGTCCTCGGCCGGCTTCATGCGCTTATAGCTCATCGCCTTGT
GGCGGCTGGCGTTGGCTTTTACCTTGGTGCCGTCGACCGCGATCGTGCCAAGCTTCACCAGCCCACATTCGCGCGCCAGTTG
CACCACCTGAACGAACAAATTCTCAAGCTCGGTCAGGTGTAGGGCACGGAAGTCACTCAGCGTGCGGTGGGCCGGGAAGTT
TCCAGCGGCCAGCACACGCAACGCGACATCCTCGTACAGCTTCCTGGCTAGTTTGCGCGAAGAGAAGACGCCGCTCGCGTA
ACCATAGATCAGCACTTTGACCATCATCGCCGGATGAAACGGCTGATTGCCCGGACCACCGCCGGCATACCGGGCGTGGAAT
GCGCTCAAGTCCAGCGTATCGACAGTCTCGCTGATGAAGTAGGCAAGATGCCCTTCAGGTAGCCACTCTCCCAGAGAAGGGG
GCAGCAGATAGGATTGGTCGGGTCGGTAAGGAAGGTAACTGGCAGCCATCCCCGTATCGTCCTCGATCTTCCGCCGATTTGG
CTTCTGCCGCGCAGGCTCCTAGGCCACCGTAGCTAAACATACGAGAAAAACCGCTTCTTGAGCGCTCTCTGAGACCACCTTGC
CGACAACGCAGAAACGGTTGCGGAAAAATCGGCGTTTCCGGCGTACGCCACCCGACCTGGACGAACTATTGCGGACCTGGA
ACTGGAGTAGCGGAATTCCCCGGGGTTTCAGCGTCGCGCCACAGCGCCTCAGATAGATGCGGTAGCCTTTGGCGGGTCGTC
ATGCCGCGGACCACCCCCCCAAAAAATTCCACAATGCGGAATGGCGGAATTTTCTGGGGTTCCCGCTTACCCCCCCTTCAGGC
GAGTAGCCAACCTGAACGGATGGTTCCACCGCAAAGGCGGGCAGGGCGGCTGTCGTAAATGCTGCGGCCAGAAGCCAGGT
TGCTAAGTTTTTCATTACAGGCTCCCGTTCACACACTGCATTTTATATTCAGGGTCAAAATAAATTTCTTTGATTTTCCGCTTTTC
CATATGCACCACAACGTCAATGGTGGAGTAGAGCATTCGCATAATATCGCTCATATCGAGCATACGGCCGATAGGGGTCGCCTT
GATAAGCAGCCCAATACGGTTAAAGGCATCGCGCGCAGAGTTAGCGTGCGTTGACATAACACCGCCTGGATGGCCGGTATTA
AGTGCTTTAAGATAATCCCACGCAGCATCATCCCTAAGCTCAGTCATGATGATACGGCCCGGTGTCAGACGCATACAGGCTCGC
AGGGCATCAGTGGCGCTGACGCGGCCGATCTTTCCTGCATCGCCGTACATCATATAAACGGCTTCTACAACGTGATCGACCGT
GACTTCGTGAACGTCCTCTAAAATAATTACACGCTCGTCTTTATGTAGCGATTTTAACAGCGCGCGCGTGAGTACCGTTTTCCC
CGACCCGGTTTCACCGCAGATCACGATAGTGCGTTTCTTCTCAACGGCGGTTTGCAGGAATGCGGGCCATTTTTCGCTGCTGT
GCAGCTCTTTAAGGAAAAAATCATCATCCGTTAGGCTTTGCTTGCTGCCGGTAATCTTCCGGCAGTCACTGAAAATCCCCTCGC
GGGTCAGCTGCTCCAGATTTTTATCGGCCGCCAAATCCTTACGAAACGCTACGGCCGTTGTACCGTCAATCACCGCAGGGGG
CAGACAGATAACGCCCCTGATCCCGCCAGGCAGGATCACGTCATTAATGGCCTGCATGGTCAGCTTGTTGCTGCTCACCAACG
ATTTAGCAAGGTTCCTAATAAAATCTGCCGTAATTGCCGCGTTCTGCACAACCCTGCGGCCGCTGAACGTATCACAGATAACTT
CCTGAAAGCAGTTAATGCGAATTTCAAAAACAGTAGGATCTTCTAAATACTCGCGCAGTGGGCCAAGTTGATAGAAAGCTGC
ATCAGTCATGATTACTCCTGAAGAAAAGCGGGCGCTAAGCGCCCACTTTTTTAGTTGTCTGCGAGCGTATAAACGCCGCTGAA
ATCGAGGTCGCGGGCAACAAAAATGCTCACCGCATCACCCTGCTGATCGTAGAGGGTAGGGGGGATAGACATGTAAGAGCG
GAGTGCTTCAGACGCCAGCTGCTCACCGCTGTTTTCTGTGCTGTTGTACTGAATGTTATTACTCTGCGTCTGGTTAACCAGCGC
CGTTAAGGTGTCAGAGAACAACGAAATCATGATCGCACCACGCAGACGCTCCCACATATGGGTATCCACCTGGCCCGGAATCC
CCGCGCTGCCGAGTGAGTTCGTTCCGGCACTGTCAATATTAACGATTGTCCCGTCCTGGTCATTGCGGATACGCTCCCAGAGA
ACAAACACGCGCGCCTGGCCGTCTTTGATACCACCGGTAATCTGCCCGTCAACCCATGAGCCTTTATCAATCAGCCTAACGAGT
CCATCAGCTGAGTAAACGTCCTGTGAAACCCGGCAGGAAACCTGACCCGGAACAGTGGTATCCAGCTCGGTGCCGGTACCAC
AGGGGATCATTTTGCCTTTCGGAACAGTCAGGCTGGGATTAGCCATGACTCCAGCGCGGCTAGCCTTCAGCCTTGCAGGAGT
CAGGTTTTTAGCGAGTGCTGAACTACCTTCGCTTGTTTCGTTGTCCTGGGGCTGCACTCCGGGACTATTGCTTGTAGCCGCCT
GATTAGTCTGGGCCAGCTCGCCGCCCAGACGACGCTGCATAGCCAGTTCTTCAGGCGAAGGTTCTTTACGCTTATTAGAGGTA
CGCGCGGCGGTATTGCTGCTGCCCGCATCTGCATCTGCCTGTGCGGCAGCCTGCACAGCACGGGCATCAGTGGCGCTATTCT
GCGCAGTTGCAGGTTTATTAACATCAGGATCGCTGTTAAAGCTGTAGTTTGGCAGTGTATTGGCCTGTTGCGCTTTACCACCGT
CTTTATCAGCTTCAGCTTTAGCCGGGGTGCGAATTTTACCCATGACCGTAATCCCGATGAATACCAAAGCAAGCAGCGCCATCA
GTATGACAAAGGCTTTCATACCAGGAGCCGAACGGCGGTTACTGCCTTTAAATCCGCCACGCTCGCTTTCGAATTCACCGTCT
CCGGTGTTTTCATCGAGTTCCTGATCTACATCGACACTTTTACGGGCCATCAGTTATCCTCCCCAATTTGAACCCTGCGCACATC
CGGGGAAGCCGTACCGGTTGCTACCGCACCGGCGCCCGGCGCGAAATTATTATTACGAACGCCAACGACTTTATCGCCCAGA
CGAATACGCCACTCTTTAGCGACGGTTTCCACCTCGATGATGTTGCGGTTCTCACCCACAACATGAGAGTTAGGCAGCGTTTC
TTTGCCACTGGCCGAAATCATGTAGACCTGCGGTAACTCCGCATTGGCCGGAAACTCAAACCGGGTAAAGCGGTAGTTATCCC
AGACGTGAACCGGCTGGATGCTGCGCATTTCAGGCTGTTCGCTCATTACGTACTGATAGTTCTTCGCCCCCGCAAAAGCCGTC

TGCTTCAGCTTCTGCGTAATGCGTTTTTTATCAGCCGCGCTTTTGGCTTTTTCCTGCTGCTCAAACGGATATTCATAGGTCAGCT
GAAGAACGGCCTGGCGCACAGCCCACGGCGTTTCAATAAAGGATTTTGATACCGTACCGTCTGCATTTTTCTTCGTTTCTTCA
CCGATGAAATGGAGGACGATGTTATAGGTGCGCTTATCGGTGACGATCACCAGGTTGGTATCACTCATGGCCTGTTTCGGCTT
CACAAAAAAATGGTTCATTTTGTGCGCAAACGTCCAGCTTTCAGAATCGCCAAAAGCATGAGTGATATAGGTTTCGTCAGGCG
CGACAACAATGTGGGTAGCCACACCGGCGATAGCGTCAATTTTGACCACATTAACAGGGTTATAAACAACGCTTTTAATGCGA
TAGTCATAAGGAGAATTGCGGCCAACCTCAAGCGCCATAACGTTAGTGGCCGCGCCTCCCAGGACTGACAAAACGACTGCTG
AAAGAAGTAGTTTTTTCATGGGGCAGCCCTCAGTTAACTTCAGGGTTGACGCGATAACTCGTCACGCGGAAACCCAGCGGGT
TGACATAACGCTGCTCAGCATTCATCGCCAGCGATTTATATTCATACCCCATAATGGCAATCCAGCGCTGCGGCTGATCATCAAC
GGGATTGCTGCGCACGCGGCGAACCGTAGTAAAGCGTATCGTTGCTACGCCGTGCGGTTTATCGAGGATCACAGAGTTAATC
TTCACGCGGGTCGTTTCACTGTCGCCCAGAACCTTATCAAGACCGTTGCGGCCCTTGAACTTGCTCTGGTAAGACTCTGCCAC
GTTCGGCGTGGACATTAAGCCAACGGCCGTATAGTCGACCTGAACTGAATAGAAGTCATAGCTCTCACGGTGAATGACATATT
GTGTCAGCCAGAACTTATCAATTTCGTCACCATAAGAGGTCTGGTCGCGGGTCAGCTTGACCTGCTGTACTTCGTGAGTGGCC
TCGTTGAGCGTTAGCAGATGTGCGGGGATTGGCTGGCTGTACTTATGCACCACGTAACCAACTAAAGAGAGTGCAAAAACAG
TTACCACCGCTGAACCGGTGGCAACCATCCAGGCGGTACGCCGGGACTTCAGCACTTCATCCATCAGATCAACTTCAAGCCCT
TTACGGCTTTCGTTGAACTCTTTAATGGCTTCACGTGTAAGCCCTGTTTTTTTATTAGCTTTCATTTGCACCACCTTGCGTATCAA
CCGGGATTGTTTTGTTTACTGGAACGGTGTTGCTCCAGTCCGGCTCCGGTGGCGGTTTATGCCCGCTGGAACAGGCGCTAAT
CAGAAGAACTCCCATAAGCAATAAGGGGTCGGTTCCGGCTGAGGGCGAAATGACACCCTAAGCTTTCGGTTCCTTGGGCCA
AAGATATTCGCCAGTCAGTAGAATGTGCGCCCAGCCCAATGGGGATATGTGGGGAAGAAATTCAGGGGGAACATCCAACCCT
TCGTTCCGCCGCTCCGTGACGGCATGACCAAGATGGACGGTATTCCAGTAAATGATCACCGCAGTCAATAAATTGAGCCCAGC
GATTCGGTAGTGCTGCCCCTCTGTCGTGCGATCGCGAATTTCCCCCTGCCTCCCGATACGGAGCGCATTTTTGAGCGCATGGT
GGGCCTCTCCCTTGTTAAGACCGATCTGAGCACGCCGCTGCATGTCCGTATCCAGGATCCACTCAATAATGAAAAGGGTCCGT
TCAATACGACCAACTTCACGAAGCGCAACTGCAAGGTTGTTTTGTCGTGGGTAAGAAGCGAGCTTGCGCAGGAGTTGGCTG
GGCCTGATTTTGCCAGCGGTCATCGTCGCGGCACAACGGAAAATATCAGGCCAGTTCGCAACGATAAGATCCTCCCGGGCTT
TTCCACCTACCAACTTGCGTAACTCCCTGGGGGTCGTATCGGGATTAAATACGTACAACCGCTTCGATGGCAGATCCCTGATTC
GCAGAACGAGATTGTAGCCGAGCAGGCTACTGGCTCCGAACAAATGGTCGGTGAATCCTGCTGTATCGGCATACTGTTCGCG
AACATGGCGACCGACCTCGTTCATCAGTAGTCCATCGAGAATATACGGTGCCTCGCTCACGGTCGCCGGGATCGACTGACAAG
CGAATGGCGCGAACTGGTCGCTTACGTGAGTATACGCTTTGAGGCCGGGAACAGAACCATATTTGGCATTGACCATGTTCATG
GCTTCGCCATGCCGCGCTGTCGGGAAAAACTGACCATCGCTCGATGCTGACGTGCCCATCCCCCAGACGCGTGACATCGGCA
GTTTACCCTGCGCGGCCACCACAATTGCCAATGCCTGGTTCATGGCTTCGCTTTCAACATGCCAGCGGGCAAGGCGTGAGAG
CTGCCAGTAATCATGCGTGTTTGTAGCTTCCGCCATCTTACGCAGGCCCAGATTGAGCCCTTCAGCGAGCAGGACGTTGAGCA
GACCGATCCGGTCGCGACATGGAGCCCCGGTTCTCAGATGGGTAAACGCATCTGTGAAACCAAGGGCTGCATCAACTTCAAG
CAGCATGTCGGTAATCCGAACGGACGGCATTCGGCGATACAGATCCAGTATGAGTGCCTCGGCACCATCCGGCACGTCTGCTG
TCAACCTGTCGATCCGCAACGTTCCATCTTCTATGCTACCGTGCGGAATAGTGCCGTTACGGGCAGCCCGGGCCAGCCGCTTA
AGAGCGATCGTGAGTCGCGCCTTTCTGTCTGCCAGCCAATCCTGTGGGTTGGAAGGCACGGCCAGTTTTGCATTTTCCTGCG
CCGCGATCATCGGCACCAGTACCTGCTTGAGGTCACCATAGCGGCGCGAATGAGCGAGCCAGACATCTCCGGAACGAAAAG
CATCCCGGAGGTGAAAGAGTACCGCCACTTCCCAAAGACGGGTATCTCCTTTTTCCTGAGCTCGTAAATGACGGTTCCATTTG
GAGCTGGGCCGCAGGAAACGCCTTTCTGGCGATGCAACACCTTTCATCTCTCCGATCGACAAAGCTGCTGCTACCAATGGTCC
GGCGACCGGCGCGGCTTCGAGCTTCAGACAGCGCAACATGCGGGGCGCATAACGACGAAAGCGATGGTATCCCTGCCCGAC
ATATGCAAGAGGCTCATCGGCTAGCGTGTTGCTGAGTTGAGTCCCTGTCGCTACCAGTTGAGCGAGCCGGTCCCATGCAACC
GAACTGGCGACAGCCATCTCCAGCGGGGTTCCGTCACTGCGGGCCTCAAGCAACGAAGCTCCCAGCGCGGTGAAGGTACG
GATCGTATCCGTGAGTGTGGCTTTAGAGCCGGAAATTGTTTCGTCATGCTGGCGCTTCGCTTCCCGCCAGGTTTTTCCTACGAT
CCTGTCATGGGTTTCGACTATGGCATCAGCAATCGCCGCTTCCCACTCCACAACACAGACGGCAAGGATCGCCCAGCGGCGG
TCCGAAGTGATGTCACGCAAACCGTCGGTGAAGTAGCGTTCACCCTGCCGACGCAGCCGGGCAATGCGATGGGCAGGTATG
CTGGCCAAAGCACTATGATTGATATTCAGGGTACGCAGAAATTCGAGCCTGTCGAGCAAACGGTTAGCAGCAGCCGAGTTGT
TACCAACCTCGAAGTTGCGAAGCCAGATGAAACGACTGATATTGCCGGCGAGCATTTCACTCAGAAGTTTGTCCAGGTGATC
GCGAACATCCGCTGTTAAATTTTCCACAATCCGCGTTTCAATCCGCCGCTCAGCGGCGACCAGAGCATCCGCGCACAAGCGCT

CGATTGTCGATACTGCGGGCAGAATGGTGGAAGTTTCCCGACACCGCACAATAAAACGATGAGCAAGATCCTCGTTTGATCT
GGCATCTTCGGCCTGGCCGAAAGTCCACTCCCGCAGATCACGGGCACCACGGCCCGTGAAGGTCTTGTAGCCGTAAATTTCG
CGCAGCGTGTCCATGTGCTGCTGACGGGTTTGGCGCCGTGTGGCATAAGTGAGAAGCGCATCAGCCGGAACTCCAAGCTGA
GCACCGACGAAGGAAAGGACTTCACGCGGGATCATCTCACCAGGAGCCAGTGCACGGCCCGGATATCGTAAGGCACAAAGT
TGCAGGGCAAAGCCAATCCTGTTTTCCGGTCTGCGGCGCTGCCTAATGTTTTCCAGGTCATCATCGCCCAGCGTGTAGAACTT
CAGTAGCGACAGTTCGTCCGTGGGCAGATCGAACAGCGCTGCTCGCTGCCGTTCGGTGAAAATATGGCGTCGTGACATACAA
ATTCGTCCCTTTTGAAGTATAGTCTGTTTTGGACAACAGCCAGCCCATATAAATCAGGGCGTTCCGATACAAAAATCCAGGAG
GGTTCAATTGGGACATCGTGCCGCCATTTACTGCCGGGTTTCAACAGCGGATCAGTCTTGTGAACGCCAGGAATTTGATCTGC
GAGCCTTCGCCGGCCGTGCCGGCTACGACGTGGTGGGAATATTTAAGGAAACAGGTTCAGGAACTAAACTCGACCGGGCCG
AGCGAAAGAAAGTCCTGGCGCTTGCCCAGTCCAGACAAATTGATGCAATCCTGGTCACTGAGCTTTCCCGGTGGGGGCGCT
CGACGCTCGATCTGCTCAATACGCTACGTGAACTGGAGAACTGGAAGGTTTCCGTGATAGCCATGAATGGAATGGCGTTCGAT
CTTTCGTCGCCGTATGGACGAATGCTGGCGACGTTTCTTTCCGGCATTGCGGAGTTTGAGCGGGATCTCATCAGCGAGCGGG
TCAAGTCAGGCCTTGCTGTTGCGAAGGCACGTGGTAAGAGGCTTGGTCGTCAGGCCGGAGTGCGACCAAAATCAGACCGAC
TTTTGCCTAAGGTGGTTGCGATGAGGGCCGAGGGACGCAGCTATCGCTGGATCGCACGCGAGCTCGGTATCAGCAAGAATAC
CGTCGCTGACATCGTGCAACGACACAGAGCTAACGCTTAGGGTGTCATTTCGCCCTCAGCCGGAACCGACCCCAATAACACC
GCATTACGGTATCCATTATCGGTTGTTGAACGTACTAATTATAATAAGTCGTCGAAATTGCAATTTCAACGACTTTTGTTTTAATT
TTATGCAGCCTTCTTCCCGCGACTGCCCCTTGCCTTACCACTTAAATTAGAACCACTGCTTCCTCCGCTGTTGCTGCCACTTCCT
CCACCTGCCGATTGCTGGCCGCCGCCACTGTTACCGCCTCTGAACGCATTACCGCCAAACATGCCGTGGCTGCCCATATTGCC
AGCGCCTGCATCGAAGAACCCGTAGAGCGTCGCGGCGTCAGCAACTCCGGCGCTGATACCGCTACCCCAGCTGGCGGCAAT
TTGCGGAATCTGGAACAGAACGAAACGGAATGACCGTCAGTAATAGGCGGAGATAGAACCAGTGATTGATGAGTAAGCGGC
ATCAGAGTTCATCGAGGACAGGAGGTTGTCGAACATCCGCATGATGAAACCAAACACGAGCGCAAGAATGACGACGACAAG
GCCATAGTTAATGACCGACGCCAGCCAGCGAGCAAAGATGTTTTTTGTCGCTCCCCACAGCAGGCAGAAGATCGCAATCGGG
CCAAAACAAAGCGTAACGGCCAGAAGGATCTTAGCCATGATCACAAAGCCCGCACCGAGGCCGCCCAGCACAACGGTAGCA
ATCATCATAATGCCGCCAATGGCATACGCGGCCAGGCCGCTCGATGAAAACACGTCTGCGGCTTCCCATGCGGTGTTGACGAT
CTTGATACCTTTTTCAATACCGCTATCAATAATCGCCGGTACGCCACCTTGCACCGACTTTATTAGGGGCAGACAGTATCCCGGC
AAATCATCCGGCAGTGAAGCGCCACGTTGACCAGTCCTCTGTTGATACCAGCCGCCCGCCGTTGCAAAGCGCAGGATAAGGG
CTATGGAAAGATACTCTTTAATCAGCGAACTCAGGCTGTCGCCCGCCCCCGGATTAAACGCTGAGTACATCCCCTGAACCATCA
GCTTGATTGTCAGACAGGTGGCAATCAGAGGCGTTACATCAGAGATAATGGTGGCAACATTGGCGCTCACCATTGACGTAATC
GCCCCGTCTACTTTTGCGAAAATGTCTGCGACTAGGGTGAATGCCATATTGCCTCCTTACTCCTAAATTTTCGGTGAATTGATTT
CGCCGCATCTTTGCCGAACACAAACAGCTGACGATCGGCTTCGCGCGCGTTTTTGCAATCAGGTTTCTGAACTTCATCCGCGT
CTTTCTTGCATTCCTGAATTGTTGCCTTGCGCTCTTTCCATGTTTTTTCGTACCACTCCACGTCATGCGAGGCATCGCAGGCCAC
TAGGAGGAAAGGGATAACAAGCAGTAGTTTTTTCATAATTACCTCCAGTTAATTGAAGGTGACGCGGTAACGTCCCCGCCGG
TTCCAAAAACAAAATTGTTGGTGGCACGTTCTTTCTGCGCACGTAATAGCTTGTCCTGTGACTGCTGCAACATGTTCATCAGAT
TCAGCTTCGCCTGCTCACCCTGAATAGCACCCTGTGACGTCTGGATACGGGCCTGTAAGTCAGCAATCGATTTCAGGTCTGGA
GTCGTTTTAATCTGCTCCGTCAGCTCCTGCATATCGGTTAGTTCCTGCATCTGGTTGTTGTAGGCTTTTTCTGCCATAACACGGT
CATAAGCGCCTTTTTCAGCCAGCTTTTTGTTCATGTAGGTAATTGCTTCGCTGGGCGTCATGTCGTCAACTTCCGCATTAAACT
GGCCCATCATGCTGTTAACTGAAGGCGTGACGGAAGAACTGGAGTTCATGGCGTCGCTGTAGATCTCCTTCCAGTTGTCCGG
CAAATTATTTGCCAGCGTGCTGGTAGACGTCCCCAGCAGATCGCCCAGGTTGGTTGTCTTTGCCATCGACTCATACATATTTTT
CTGCGTTTGCAGCTGGCTTTTAAGCTGCTCCAGCTGCTGCGCCATTTGCTGAAGCTGCTCGACCTGTTTAGCCAGCTCAGTAG
GGTTGGTCACGATGATGCCTGCGGAAGCGCTTTGCGCGCCTCCCAGTATCAGGCCGGTGGTCAGCAATACTGCCGTCAGTGT
TTTTTTCATGGTGTTTTGCCTCGTTGTTAAGCCGTCAGGCGCCAGTATTCTTTGAGCCATACTTCAGGGTCATTACCGAGCCGT
TCAACCAGCTCATGCGCAATTTCGGCGTTTTGTGGTTCACCGGACAACACGCTAAGAACGTTGTCCATTGTCTTAATATCTGCA
TCAATATCGTCGCTGTTGCGAGGGTAGAGATTGAAAGAAGCAATCGCAGACTGTTGCCCTTGTTTAACCAGGAACTGTCTGG
AGTGTTCGGTAATCGACATCAGCGCGTCGTATTCAGCGTCAGTAAGGAAGGCATAATCCTCTCGGATAGCTTCCGGATCGCGC
AGGCAGATTTTTGTGACGGTCTGCGACATGATCGTGCGGCCAATACGGCTGGACAGCGCATCGTTCGGCTCCTGCGTGGCAA
ACACATAGATAGCGTCTTTCTTACGGTCAGTTTTGATACCACGCTTAACTTCACGCTCGATAACCGGATCGTCAAGGTAGGCGT

GGAACTCGTCAAAGCACTGAATGACGCGGCGTTTGCCGTCGATGGAGTCACGTACCCGGTACAGAAGGTACATCATGAGCG
GAGTACGGGCCGGGCTGGATACTTCCTCTTTGGCTGCGATAAACTCGGATAAATCGAAGCCAAAAATATCATTCGCGCTGAGA
TCCAGGCTGTCTTTATCATTGTCAAACAGCCAGCCATACTGGCCTTCGCGCGTCCATTCGCGCAGCAGCCCTTTCAGTGATACG
CCATTTTCCACAACTTCATTCACGTACCCGTCCAGGATAGTAACGGTGCGCGCCTCGCGTGGAATAAGTGAGCCTTCCCCCATA
ACCGCATCAACGCCTTCAGCCAGTTCGGTAGCCATCGTTGCGCTGATAGGCCCGTTATTGGTGGTTTCCACACAAATGCGGAA
CAGGTTTTTAATGAGGGCAATATTGCGTTTGGTCGGTTCAATCTGAAGCGGGGCAAACGCGACGGCATACCCTGTTGCAGAA
CTTTATAGTAGCCACCAACGCTTCGAATGAACGGCTCCATACCGCGGTCACGGTCATAAACAAAAAGCCGCGGGTTGTACTTC
ATTGACTGCGCCAGCAGGAAGTTAAGCAGCGTGGTTTTACCTTCCCCCGACATACCCGTTATTAACGCATGGCCCAGCGGGC
GTTTACCGTAGGAAAGTTCTTCAAGCGGGGTCGCATGGAAATTAAAATAGAGTGGCGTACCGCTGATCGTGCGGAACATGGT
CAGCGCTGGCCCCCACGGGTTATTGTCAGGCTTGCCACGCATAAAATTGTGGAACGGGCTGAAGTGCAGGAAGTTCCATGA
GTTTATCGGAACCGGGCGCGGCGCCCATTTCTGGTTGCCAGGCAGTCTCGCATAATATGCAGCCTCAGAGGCCAGGCTGAGA
GTCCCGCCAACCACGCCACAGCCGGTTAGCGTAACCTTCACACGACGCGCTTTGCGCTGTACCGCGTTTTGGTCATTATCCCA
GACATGCACGGTTCCATGATGGTAGCCCATCACGAACTCTCTGGACGTCAGCATATCGAGCGCGGTACCAAGCTGTGCCAGCT
GGCTTTGCGCACGGTCGCGCGTTTCCTGCAAAGATTTTTCCTGATGCGTCAGAAACGTTTTAGCTGAAGATTCAGAGAGGCA
AGAAAAACTCTGCGTCAGAAGGTATTCAAAATCGGCTTCTTTAAGCATGTTAAGCTGGCCTGGCTCTGTATCTTCTTCGTATTC
ACGAAATTCAATGCCGGTGGTATAGAAGTTGTGATCGTCCCGTTCTGATCTGGACAACATCCCCCCACAGTGAGCTAACAGGG
CGGTTGTCCATGATGTACTCACGGATACGATCACGGCAAACAGGCACGATGGCCCATTCCATATTTGCGAGGAAATAAAGGAA
CTCCAGCGCTTTTGAATAAGCGTGAGCCTGCGAAGGTTCAGGCTCGTTGCGTTCGATAACAATGGCTTCGTCAAAAATGTCTG
ATTCATCGACTTCAGCAAGTTCTTCACGTTCTTTTTTATGGCGCAGGAATTTCAACACCGCGTTTGTCACGATAATATGATACCC
AACTGCTGAATGCCATACGGCTTCATTGCTTCCAGGATTTGTTCAGAAATATCTTCCAGACCTTCAAGCGCCTCATTCTGCATTC
GCTGAATTTCGTCACGAGTCGGCTTTTCAAATTTCGCCAGAACTTCTGTGTTTTATCCCTACCTGTTTGTAAATAACGGTCAGAT
AAAGGTCATTAATCAGCTGCTTGGAATCACCGTGCAGCTTACGGTTATATTGATCAACATAAGCAGGGAAAAAATGGTCATACT
CACCATCCGGGTACTCTTTAGCCTCATGGTGATATTCATGCGTCCACAGCTCTACATGGTCTGTTCCGAAGCTCTTGACCAGCG
TGATTAAGGTCTTTATGCCAGGTGACCAGTTCCCGATCTGATGCGCAGTCATGTGTGCGGCCATCCAGCTTGAAAAAAGCCGA
TCAGATCGCCGTTTTCCATCGAAATCACGTAATCATTGAGGTGATAAGAATAGGGCAAATACTTTTTATTTACTGATGGCTCCTT
ACGGTAGGCATCAATTTTTTTTGGCTTCGTAGCGGTGGCAGCTCTCATTATTTTAAACCCTTACGTTTGTAGTCAACAGAGGAA
TAAGACGATCCTCCCCACTGCTTAAACGGAGAATCAAACCAATTACTGAATTTGGTTTTCAACCACAGGCCCATAATTCGAAAC
ATGCGGTCGTCATATTTTGTTAATGCAGCTGAAGGAATCCACAAAAAGACGAACACCAGGATCGCCCACATATGAATAATCATA
AAAAGCGAGGCAGAAATCATGAATATCATCATAGCTACATTACGTGGTACACCCAGCGCGCGAGGTAAGCGAGTCGCACCTTT
GAAAAGCGGTCTTTTCCCGTCAACGAACATCTTTGTAGTCCTCACTTTGGGGGACAAAAGTCCCCCCCTTTTCGCGTTGTTCA
GCTTCCTACGCCCGTCAGGGAAACCAGAAATGATGCAGAACCAATACCAATCAGCGAGATTACGATACGAGGAATAAAGCTG
GCTGGGATTACGTGAAGCATCCACATAAAGCAACTAACCATGATCGCAATAGCACAACCAATTGGAATCCATGTGCTCAACCA
CGTCTGGATTGATGTAGCGGTTGATTCACCAGTATCGGTGCCAGCGGCCAGCGCAATTTGCGGCAGGGCAATGGACAAAACG
CCCATAACTACCGCAGGGCCATACTTCTTAAACAACGTGGTCATACTTTCTCCTTACTCACTATCTTCTGTTGATTGCAGATTTAA
CCGGCATTCTTCAACGCCACACTTATCTTTCAAAAACTCTGCCAGTAGCCTTAAATCAGCCCAGGTTCGTAGTTCATGCTCTTTG
TTAACCCGAATGGAACATGCGATCTGGCCCAACTGTTCATCGTCTATCTGAAAAACGGGATACCACGTCTTTAATGAAACCCGT
TTTATCGTTACCTCCTTCACTTTTCCTGTTTCAAATAGCTCCAGGATCTGTGATTGCAATACAAGGTACAAACGTTCCATCCATAA
CCTCACTCCCCCTTCGCTGTTTCCTGTTTGGTTAAAAAGGCATCCGTATTATTTCGGCTGAAGGCATCGCCATCACCCGAACCA
AAAACATCTTGTTCACCGTCATACTGCGTGGCCGTACTTTTTGCCTGCTGAGGCTCAGTCGCGGTGCTGTCCTCACTGGTCTG
GCCATCAGGTAGCAGCGTAGGGATTTTCAAATCAGTTGATTGACGCGCCACGTTGATAACTTTCGTGACATACCCGTTAGAAA
TCCCGTTTATGAGTGAGCCGGTGTTGTAGCAGGAAAGCGCGTGTCTCAGCGCAACCTGCCCGGCTGGATAGGATTTCAGGGC
GCTATCATAACAGGCTTTAAGGATGGTCTGGCTCGCCCGCAGGTTGATGCAGGGCTTGAAAATATCGTCAACCGAAAGACCC
AGGCCCACTAAATTATTTGAGTTAATTTGTGCAAGGCCCATATCAAAACTTTTATTATCCTTCAGCAGAACTTTCGAAACGCTGA
CGGCCTCAGCTTCAGTACGTGGTTGCTGTTTTAACTGGGTACTACCACCGTTAATATTGATCCTGTACGGCCCATTTGAGGACT
CATGGCCGACGATGTACGCCATTGTTAAGGGTGAAACATCAGGCGCACATCTCTGCGCAAGCCTGGCAACTTCATCTGAGGC
AGGCGCAGCACTGGCACGGCCAGCACAAGCAAGGCAGGCCAGAGCGAGAACCAGAAGTTTTGGATGTTTACTCATTTACGT

TTCACCGCGTAATTGTAATTTCGACGACTTATGTTTATGATAAGGGCTAATGAGATCGTGTCAAGAAGAAACGAAAAGAAAAC
CTCTGAAGGAAGGAAGGTAATGCTGGTTCCCCTGAAATCAGAAAAACGCCCCAAAGGTGAACCCATGTATCGTGACCCGGAT
AACCCTTTTAATACGTGGACTGGTATAGGGAAGCGCCCGGCCTGGCTAACTGCAAAATTGGACGCTGGCATTAGCCTGGAAG
CCATGAAAATGCAGGGCGTTGCCAACCCCAGAGAACATAGACAAGTAAAATACCGCGACCCCAGGAACGCAGAAAATACCT
GGTCCGGGACTGGCCGCCGACCAACATGGCTCAAAGAGCTGCTTGATAGTGGTTTATCACTTGATGATCTAAAGATATAACCG
GAGAGTATAAAAATGGGCGATGTTATTGATTTTGCGGAAAAGCAAAAAGGCAGAAGGAAGAAAAAGGCATTTTCCATTCCG
CCCATTTTAAGAAAATTCCGAGTCCATGCTATCAGGTTACTCGCCAGCATCATCAAATCCGGCTCTTATTCAGTTGCTTATATCGT
TAAGAAAATTACAGGAAAGTTAATTAAGTTGTTCGCAATGTTAACGATTTTCGTTTTTATTGTCGAATATATCGCAGGCGATATA
GGTTACAAATCTATTTATAACTCTGCGTTATTATTAATATTACTTACCGTTATCAACATTCTGGCGGGTGTATATCTGAACAAACTG
TTAAGGACAAAACAATGAAGAAACTCTTAATCCCTCTGATAGCAGCTGGTAGTCTGCTTTATCTTCCTGCCAGCCATGCTGAAG
ATCCCTGCAAAGTTATTATGTGCATGGCGGGCAAGCTCACCGGCGATAGCGGCGGAAGCGAGTGTAACAGTGCTGAAGCTGC
TTTCTTCAATATCGTTAAAAAGAACAAGCACGGCTTTTTACCCAACCACACGAGGGATGCCAGGAAGGCTTTTCTTAATGAAT
GCCCGGATAATGGCGAAGGTGGAAGTAACCAGTCGATGATAAGCCAGATCATAAGTAAATACGGGAAAGTTCGCTTATAGGC
GGGCTAGGAATAATCTCAATTTAAGGAGCCAACGTGAAAAAAATTATATTAAGTGCATTAGCCTGTACAGCTCTCTTAACTGGT
TGCGTCAGCCAAGATAAAGGTAATGCGATGCAGAGCCAGATGAATAACCAGCAACGCCAGATTAACGAATTATCCGTTCGTTT
GCAGTCTGCGGAGTCCCGGCTATCAAAGCAGGAAGAAAAGCTGCGCAACGAACTGCTGCAATCCAGCGGCTATTGCTATCTG
AATGGCGCCCGCTACTCGACCGGCACCGTACTTTACGGGCGGATTTGCCAAAATCAGTCAGGCAGCGCTTCGTGGCAGGTTT
ACAGCCGTCGCTAAACACCAGGGCGGTTTAACCGCCCTTTTCTTTACCCACAGAAAGCACACTATCACCATGTTCCCCTTCCCC
ACTACAGAAAACCTTATTCTATGGGCTTGCAGCGCCATCGCACTGTTTGCCGTTGTATTCTTCCGGCGTTCAGTACGCAACCGA
CGACACAAAAGGAAGCAGCAAAGTGCGCGGCGGGTGCTGGAGCGCATAAAGACGTTGCCGGGCTTCCCACAAAAAATTAA
CTACCTGAGGAAAATTGATCCTTTTGTGTTTGAAGAACTGTTGCTGGAAGGATTTGAAGCGCATGGCGGCTTTGTTGAATAAA
TCAGATTTCGGGTAAAGTCTCCCCCGTAGCGGGTTGTGTTTTCAGGCAATACGCACGCTTTCAGGCATACCTGCTTTCGTCATT
TTGTTCAGCGCTCGTACCAGGGCCATAGCCTCTGCAACCTGACCATCGTAGTCACGCAGTGTCAGTGAACCTCCGAACAGCTG
TTTTACCCGGTACATCGCCGTTTCCGCTATCGAGCGACGGTTATAATCTGTTGTCCATTTCCACCGCGCATTACTCCCGGTCAGC
CGCTGATTCGCAACAGCACGGTTACGGTCTGCATATTCACCGGGCCAGTAACCCGCGCCTTTTCGGGGCGGGATAAGCGCGC
TGATTTTCTTACGCCGCAGTTCATCGTGACAGAGCCGGGTGTCGTAAGCGCCGTCTGCCGATGCTGCCCTGATTTTTCTGTGA
GTCTGCCGGATAAGACCCGGGAAGGCTTCTGAGTCCGTCACATTGTTCAGCGACAGGTCTGCACAGATGATTTCATGTGTGTT
GCTGTCAACGGCCAGATGCAACTTTCGCCATATACGACGGCGTTCTTTGCCGTGTTTTTTGACTTTCCATTCGCCTTCACCAAA
GACCTTCAGCCCGGTGGAATCAATCACCAGATGCGCGATTTCACCCCGGGTGAACGTTTTGAAACTGACATTAACCGACTTTG
CGCGCTTGCTGACACTGGTGTAATCCGGGCAGCGCAACGGAACATTCATCAGTGTAAAAATGGAATCAATAAAACCCTGTGC
AGCCCGCAGGGTCAGCCTGAACACGCGTTTAATGACCAGAACGGTGGTGATGGCGAGATCAGAATAGCGCTGAGGTCTTCC
CCGTGATGAAGGCGTTGCCGACTCATACCAGGCCTGAATAGCTTCATCATCCAGCCAGAAAGTTATGGAGCCACGGTTGATGA
GGGCTTTATTGTAGGTGGGCCAGTTGGTGATTTTGAACTTTTGCTTTGCCACGGAACGGTCTGCGTTGTCGGGAAGATGCGT
GATCTGATCCTTCAACTCAGCAAAAGTTCGATTTATTCAACAAAGCCGCGCATGGCTTCAGAACCATCAGAAACAAACGCTAT
ACCGGCGATGGAGGCATTGACGGCCAGGTAATAATAGGAAAATATCGCTATCTTATTCAGGCTAAACGCTATCGCGGCCATATT
GCTTTACAGCACGTACAGGAGTTCGAGAAGTTGCTTAAACGTCATAACTGTCGCGGTCTGTTTTGCCATACCGGGAAAACCG
GCGCAGGTTCAAAATCTGTCAGTATTGCCAGTGAACGGATGGAGATTATCAGCGGCCAGCGCCTGATAGATTTGCTCACGCCC
GGCAGCTCCTTCACTATCGCAACCGCCCCGCAGACGATGATGAAGCGTACCGCAGCAACACTAGAAACGAGCACCATTGTTA
AAGATGCCGGTAAAGAAAATCGATACCATGAGAGTTAATTAAATGAAGTCAGTAACTATAGAAGCAAAAACATTTGCTGAAAT
GTTAGGAATAACAGAAGGTGAATTAATCTTTGCCATTAAGAAAACTGGCACATTCAAAAACAAGACCATCCCACAACCTCATG
AGCCACATAAATCAAATAATAGATTTTTATATTCAGACGTAATGAGGTTTATAGAATCACTAAAAGACAAAGAGAACCGGTAAT
GACTCCAACTTACTGATAGTGTTTTATGTTCAGATAATGCCCGATGACCTTGTCATGCAGCTCCACCGATTTTGAGAACGACAG
TGACTTCCGTCCCAGCCTTGCCAGATGTTGTCTCAGATTCAGGTTATGTCGCTCAATGCGCTGAGTGTAACGCTTGCTGATAAC
GTGCAGCTTTCCCTTCAGGCGGGATTCATACAGCGGCCAGCCATCCGTCATCCATACCACGACCTCAAAGGCCGACAGCAGG
CCCAGAAGACGCTCCAGCGTGGCCAACGTGCGTTACCTAACAATAAACCTGTTTAAATATCCAGATAAAAACATTCAATCTGG
GTCAAATGAGTGATACAGTTTCACCCATAAGACCCAATGGAGGCAATATGTCTGAATTTGAATTACTGGCGCAGGATCTGCTTG

AGAAAGCAGAAGCGGAAGAACAACTGCGACAGGAAAATTATAAAAAGCTGCTCGGGCAGGTGCTGGAAATCTATGACCAG
AAGTACGTGGCTGAACTGCTTAGAAAAGTTGGTAAAAATGAGTGGAGTCGCGAGACTCTTAATCGCTGGATTAATGGTAAGT
GCTCACCTAAGACGCTGACGTTAGCCGAAGAGGAACTTCTACGAAAAATGCTTCCGGAAGCGCCTGCACATCACCCTGACTA
TGCCTTCCGGTTTATTGACCTGTTTGCTGGGATTGGAGGTATACGGAAGGGCTTCGAAACCATCGGTGGCCAGTGCGTTTTTA
CCAGTGAATGGAATAAAGAGGCTGTGCGCACATATAAAGCTAACTGGTTTAACGATGCTCAGGAACACACTTTCAATCTCGAT
ATTCGGGAAGTCACGCTCAGTGATAAACCTGAAGTACCTGAAAACGATGCCTATGCTTACATTAATGAGCATGTGCCGGATCAT
GATGTACTTCTAGCAGGTTTCCCCTGTCAACCGTTCAGCCTTGCGGGCGTAAGCAAGAAAAACTCGCTCGGGCGCGCGCATG
GTTTCGAATGTGAGGCTCAGGGAACGCTTTTCTTCGATGTGGCGCGTATTATACGCGCAAAAAAACCTGCCATCTTTGTTCTT
GAAAACGTTAAAAACCTGAAGAGCCATGACAAGGGTAAAACCTTTAAAGTCATCATGGATACCCTCGACGAACTGGGCTATG
AAGTTGCGGATGCAGCTGAGATGGGCAAAAACGATCCTAAAGTTATCGACGGAAAGCACTTTTTACCTCAGCACCGAGAACG
TATCGTTTTGGTCGGTTTCCGTCGTGATCTGAACATTCACCAGGGCTTTACCCTGCGCGATATTAGTCGTTTTTATCCGGAACAG
CGTCCGTCATTTGGCGAACTGCTGGAACCCGTGGTTGACAGCAAATATATACTGACGCCGAAACTCTGGGAGTATCTCTATAAC
TACGCCAAAAAGCACGCAGCTAAGGGTAACGGATTCGGTTTTGGCCTCGTTAATCCTGAAAATAAAGAAAGCATTGCCCGTA
CGCTTTCTGCTCGCTATCACAAAGACGGGTCTGAAATTCTGATAGACCGTGGCTGGGATATGGCCACAGGTGAAACAGACTTC
GCGAACGAAGAAAATCAGGCGCATCGGCCCCGCAGGCTGACTCCACGAGAGTGCGCGCGCCTTATGGGTTTTGAAAAAGTA
GATGGCAGGCCTTTTCGCATTCCTGTGTCAGACACTCAGTCGTACAGGCAGTTCGGTAACTCCGTAGTGGTGCCCGTGTTTGA
AGCCGTAGCCAAACTGCTTGAACCTTATATCCTGAAAGCGGTTAATGCCGATTCGTGCAAGGTTGAACGAATCTGATCGCTCC
TCCCGGTATTTATGCCGGAGATAATCTATGGAATATCTGCGTAAAGCCCTGTCAGCTCAGCAATAAACGCACCTAGCGTCATTAG
CTCAGCTCTCACCGCCTCCGGGTATTTTTTGTGCAGCGATGATGGCACGACCAATCTGACACCCGACTCCCGCATCTCCCGATA
TTGAGCCAGAGAAACTCCCTCTTGGAGTGTAAACAGATGCACCTGATGAATTTTATCGGCCTCATTCAGTATCTGACGCCAGC
GATCCTTACAGGTAGTCTTGACTGCCAGCATGCGCAGATTTTCTACGGGAAACTCAGTATCGTGGTAAGCCCCTGCGGAAGG
GAAAAGGAAATCGGGTTTTTTATTACCTTCTGTGATGGCCTGCGTCGCAAAGTGTCGCAGGCCGTGCTCAATGAATAGATGCT
CCAGGTGCAGTTCCAGAGACTTCCCGGCTCTGGATTTACGGCGATTGCTGACAGAATTGGCCAGCGCAATAAATGGCACTGT
TGCAAAGTTAGCGATGAGGCAGCCTTTTGTCTTATTCAAAGGCCTTACATTTCAAAAACTCTGCTTACCAGGCGCATTTCGCCC
AGGGGATCACCATAATAAAATGCTGAGGCCTGGCCTTTGCGTAGTGCACGCATCACCTCAATACCTTTGATGGTGGCGTAAGC
CGTCTTCATGGATTTAAATCCCAGCGTGGCGCCGATTATCCGTTTCAGTTTGCCATGATCGCATTCAATCACGTTGTTCCGGTAC
TTAATCTGTCGGTGTTCAACGTCAGACGGGCACCGGCCTTCGCGTTTGAGCAGAGCAAGCGCGCGACCATAGGCGGGCGCT
TTATCCGTGTTGATGAATCGCGGGATCTGCCACTTCTTCACGTTGTTGAGGATTTTACCCAGAAACCGGTATGCAGCTTTGCTG
TTACGACGGGAGGAGAGATAAAAATCGACAGTGCGGCCCCGGCTGTCGACGGCCCGGTACAGATACGCCCAGCGGCCATTG
ACCTTCACGTAGGTTTCATCCATGTGCCACGGGCAAAGATCGGAAGGGTTACGCCAGTACCAGCGCAGCCGTTTTTCCATTTC
AGGCGCATAACGCTGAACCCAGCGGTAAATCGTGGAGTGATCGACATTCACTCCGCGTTCAGCCAGCATCTCCTGCAGCTCAC
GGTAACTGATGCCGTATTTGCAGTACCAGCGTACGGCCCACAGAATGATGTCACGCTGAAAATGCCGGCCTTTGAATGGGTTC
ATGTGCAGCTCCATCAGCAAAAGGGGATGATAAGTTTATCACCACCGACTATTTGCAACAGTGCCTTATTTCGAGCATTTTCTT
GCGAAAAAGCCGACCGGGAAAATTGACATGCGTACCATTCGCCTGGGGTGTAATAAGGCGATAGACACCGGTACCTACACAT
TTACCTTCGCGGACAAATCAACGGTTTCAGCCCGCTACACATTCACTTATGCATGGGACGGTAAAGAGTGGAAAATTTCCACA
CACCACTCTTCAGCGATGCCTGAAGGGTAAAAAGCTGTGCAGGGGGGCGGAATATTCACCGCTCCCTGAAGACTAGCCAATAA
TCGCGATGCCAAGACGTTCCATGAGCAACGATGCCTGGTAGTTGTCCAACTTAACGCCTTGTAAATCAACGCGCCGAATATCTA
AGTCACCCAACTCCGAATTGGTCAGATCGCAATGTGTGAAGTTTGCTGCTCGCCAGTCGAAAGTCGAAAACTCGCCGCCGGA
GAGATCTGAACCACTGAACGTCGCGCCCAGTACCTGGGCCCCCATCCAACGGTTTTCCCACAGCTCACACTTTTCCAACACGA
CTTTCGAAAAATTGGCGTAGCTTAGATTTGTGTTAGTGATATATGCACTGCAAAACCAGGTGCGAGTAGTGATCATATTCATAA
AACTTGCGCCGCGAAAATCTGCGCCTTGCGCACGACAGTGGCGAATTTCAATGCCAAGCGCACTGGCATTGCGAAAATCCGC
CATGGATAAATCACAGCTTTTAAAAATGGCATCTTTCAGCATCGCACGACTAAAATTGCACCCTTTCTGGCTTTCACGATCATAG
AACTGACAGCCGATAAATTCAGTGCCGCTCAGGTCGGCACCTGAAAAATCACAGTTAAAAAATGTACTATTTTCAATTTTCTCA
CCGGTGAAGCGGTTTCTGTTAATTTTTTCGCCAACGAGTGCCAGAGCCATATTTTGTGCCTGTTTTTTTATACAGTAATGGCGT
CATGGTAAACCCTGATGAGGTTATGCGTCAAATCCGCCAATATAACATCTGCAAATGTGCGTTAAATCTGGTGTTTTTTCAGCAA
AGCGCGAAGCTGATGGTAAGTCAGACCCAGTAATTCAGCGGCTTTTTTCTGGTTAAATTTTGCCTGCTGTAAGCTGGTTTGTA

GAAAGTCTTTCTCTTGCTGCTGCTGGAATTCACGCAGATCCAGCGGTAACCCTACAGACATCGGTTTAGTTTCCGGCGCCTGC
GGCTGCGTCTGGTTCTGAAATCCATCCCTGTCGGTGTTGCTTATGCAGTCTGGTCGGGACTCGGCGTCGTCATAATTACAGCCA
TTGCCTGGTTGCTTCATGGGCAAAAGCTTGATGCGTGGGGCTTTGTAGGTATGGGGCTCATAATTGCTGCCTTTTTGCTCGCC
CGATCCCCATCGTGGAAGTCGCTGCGGAGGCCGACGCCATGGTGACGGTGTTCGGCATTCTGAATCTCACCGAGGACTCCTT
CTTCGATGAGAGCCGGCGGCTAGACCCCGCCGGCGCTGTCACCGCGGCGATCGAAATGCTGCGAGTCGGATCAGACGTCGT
GGATGTCGGACCGGCCGCCAGCCATCCGGACGCGAGGCCTGTATCGCCGGCCGATGAGATCAGACGTATTGCGCCGCTCTTA
GACGCCCTGTCCGATCAGATGCACCGTGTTTCAATCGACAGCTTCCAACCGGAAACCCAGCGCTATGCGCTCAAGCGCGGCG
TGGGCTACCTGAACGATATCCAAGGATTTCCTGACCCTGCGCTCTATCCCGATATTGCTGAGGCGGACTGCAGGCTGGTGGTT
ATGCACTCAGCGCAGCGGGATGGCATCGCCACCCGCACCGGTCACCTTCGACCCGAAGACGCGCTCGACGAGATTGTGCGG
TTCTTCGAGGCGCGGGTTTCCGCCTTGCGACGGAGCGGGGTCGCTGCCGACCGGCTCATCCTCGATCCGGGGATGGGATTT
TTCTTGAGCCCCGCACCGGAAACATCGCTGCACGTGCTGTCGAACCTTCAAAAGCTGAAGTCGGCGTTGGGGCTTCCGCTAT
TGGTCTCGGTGTCGCGGAAATCCTTCTTGGGCGCCACCGTTGGCCTTCCTGTAAAGGATCTGGGTCCAGCGAGCCTTGCGGC
GGAACTTCACGCGATCGGCAATGGCGCTGACTACGTCCGCACCCACGCGCCTGGAGATCTGCGAAGCGCAATCACCTTCTCG
GAAACCCTCGCGAAATTTCGCAGTCGCGACGCCAGAGACCGAGGGTTAGATCATGCCTAGCATTCACCTTCCGGCCGCCCGC
TAAATATCTCCTTTTGGGTTGTTAATAAAACATCCAATAAGTTGACTGTGCGTGAAAAAGAAAGTTTTGTGTGATGGCGTTGAA
GATCGCACCGTTAAGCTCTTATGTGGGATGGTGCAGAGCTCGACGACTACCGATAAAACGCAACCGCCGCAAACAGACAAGA
AAAAGCCCCAACTGATAACAGTTGGGGCTTCAGTATTGTGATTGGTGGAGCAATAGCACCCTGAACCCAAAACCTTCTCGCTC
AACCGGTAGTGGCTGATAACAACTCGTGAGGGCTATTGCGGGTTAAGCATTTAGCGATGTCTAGGGCCAGACTGGACGTCTG
AACGCAAGCCGCTGATACTGTACATAACCACAGTATCAGCGGAGGATACCCATGTCGCTGGCAAGGAACGCCACGGCGAGTC
AATCGCCCACTCAAACAAACGGTTACGAACGCCACCAACCCGACCAGACGCTGCTCTACCAGCTGGTTGAGCAGCACTACCC
AGCCTTCAAAGCCTCACTCGAAGCCCAAGGTCAACACCTGCCTCGCTACATCCAACAAGAATTCAACGACCTCCTCCAATGTG
GCCGTCTGGAGTATGGTTTCATGCGGGTTCGCTGCGAGGATTGTCATCACGAGCGTCTGGTCGCCTTCAGCTGTAAACGACG
CGGCTTTTGCCCTAGCTGCGGTGCCCGCCGGATGGCCGAGAGTGCGGCGCTGCTGATAGACGAAGTCTTCCCCAAGGAGCC
CATTCGCCAGTGGGTGCTCAGCTTTCCTTTCCAGCTACGCTTTTTGCTGGCTCGCCATCCCCAGCTGATGGGCCAGGTCTTGA
GTATCGTCTATCGTACACTCTCAACTCATCTGATCAAAAAAGCCGGTTACACCAAAGCCTCTGCACAAACTGGCTCAGTGACTC
TTATCCAACGCTTTGGCTCCGCGCTAAATCTCAATGTCCACTACCACATGCTGTTTCTCGATGGTGTCTATGCCGAAGATGACTA
TGGCAAGCAACGCTTCCATCGTGTCAAGGCACCCACTTACGATGAGCTGAATACGCTCGCTCACACCCTCAGCCATCGCATCG
CTCGCTGCATGGAAAAGCGTGGGATTTTGGAGCGTGATGCCGAGAATACGTGGTTGACACTGGAAGAGGGCGAAGACGATA
CGCTGACTCAATTACATGGTGCTTCGGTTACGTATCGCATTGCCGTCGGCCCCCAGCAAGGGCGCAAAGTCTTCACCCTGCAA
ACCTTGCCAGGGCGTGAGGATAAAGCCGACTCAAGCAGTCGAGTAGCCAACCATGCTGGTTTCTCGCTACACGCCGGTGTGA
TGGCCGAAGCGCATCAGCGGGATAAGCTTGAGCGCTTGTGTCGCTACATTAGTCGGCCAGCGGTTTCAGAAAAACGTCTGGC
ATTAACCGCCAATGGGCAGGTGCGTTACGAGCTCAAAACTCCGTACCGCAATGGCACCACCCATGTGATCTTCGAGCCGCTG
GACTTCATCGCCAAACTCGCTGCGTTGGTACCTAAGCCGCGAGTCAACCTCACACGCTTCCACGGCGTCTTTGCACCGAACA
GCAAACACCGAGTTCAAGTAACACCCGCCAAGCGGGGCAAGAAGCCCGACAAATCGGAAGGTCTCGATACTAACTGGCGTG
ACAAGAGTCCTGCAGAGCGCCACCGCGCCATGACCTGGATGCAACGCCTCAAGCGAGTCTTCAATATTGATATTGAAGTCTGC
GAACACTGCGGCGGTCACGTCAAAGTGATTGCCAGCATCGAAGATCCGAAGGTCATTGAGCAGATTCTCAAGCATCTGAAAC
AGAAAACAGCCAAGGCGAATGCCGCCAAGCAGCGTGAGCTGCCACCAGAACGAGCGCCGCCACTGACTCCCAGCCTGTTC
GATCCATCACAGAGTCGTCTCTTTGACTGACGACCCCAAATCCAACACTGCTCAACACTGCCAACTTTTAAACGGGGCGGTGG
GGCAGTTTGTATCTCTCGAGCTATCAGGCTAGAGATTTTACCGCCAAATCGAACCTTATTAGAGCGGTTTAGGCTGGACCGGC
AGTTAAAATTGGGGCTTGAGCGGTAAACGAGTGAGGGAATTTCAGGTAAGATACTTCGGATGAGGAGCAAAAAGGTGGTTT
ATACTTCCTATACCCAGTAGTGTTCCCCTGTCGGTTTGGTTGGGGTCGATGCCCCACTTTGGGCGCGGCGTTGGGCGCCGCAA
GATGAAAGCTCTGTGTGAAGGCTTGGATATTAAGACCTTGGATGATTTCAATGCCATCACCGAAGAGCAGATCGTTAGCGTCG
ACAAATTCAGCTACAAAACTGCCGCAAAAGTGCTTGCAGGTATGGCTGAACATGCCGAGCTATTCAACAAGATTCAATCAATC
ATCGGGTTCAAACAGCAGGTCACTTCGGGGATCGTTTTACCGGTGAGAAGATCGTGATCACCGGTTTTCGTGACGCGGCCTT
GGAGAAGCTGATTGAAGCTGAGGGTGGTGAAGTGCAATCATCAGTCTCGTCGAAGACCACCATGGTGATCGCGGCATCCACT
TCCGGATCTTCCGGCAAATTGAAGAAAGTGCACGATCTCAACAACAGCGGAAAGGCAAATATCAAACTGATTGATCTGGCCA

CCTTCCGTAAGCAATATCTGGAACAACCAGCATCGACTGGTTTGGAGTTTTAATGAGTCACCCACAACTTGAGCTAATAGTCGC
TGTGGATTCTAAGTTGGGATTCGGGAAAGGCGGCAAGATTCCATGGAAATGCAAAGAAGACATGGCGCGATTTACGCGGAT
TTCTAAAGAGATCCGCGTGTGCGTTATGGGGAAACACACGTATACTGACATGCGTGACATGCAGTTAGAAAAGGATGGCGCC
GAGGAGCGAATCAAGGAGAAAGGAATTCTCCCCGAACGCGAATCGTTCGTGATCTCCTCGACGTTAAAACAAGAAGATGTC
ATAGGCGCTACTGTCGTTCCTGATCTTCGTGCTGTGATCAACCTGTATGAGAATACCGATCAACGCATTGCTGTCATTGGTGGG
GAGAAGTTGTACATTCAAGCTCTTTCATCAGCAACGAAACTGCACATGACCATAATTCCAAGAGAGTTCGACTGTGATCGATT
TATTCCTGTTGATCCGATCCAGAACAATTTTCACATTGATTCCAGTGCCAGCGAGACTGTGGAGGCAACCGTTGATGAGACTC
AAGAGCGCATTCACTTTGCTACTTACGTGCGTAACAATCAGTAACGCGCTGGCAGTGGAAGAACAAGAACCAGAAACGAAG
CAGACGGTGTTGGCCGTCTGCTTCGAAACATCATTATTGGCTCGAGAACTGATCGAGAATACCATTCTTGCGGACGACATCCA
AAACAATACAGAAAAGATAGCTTTGCAGTTGTCGGAGCCCCTCCCCCCAACTTATAATGGGATTATTGCTGGGCTAAACAATG
CTGTCGCCATGCGTGGGACAGCAAACGTTGAATCGCCATCAAGAGCATCCACAGATTTACGTGCAGCCGTCATCGGTTTATGG
TGTATAAATGACACCATAGCCGCCCTGAATCAGACTGATAATGCAGTCTTACAGGACGGCGAGTTGCAACAAACACTTGTCGA
TTATTACAACAACTTATTGGCTGACATCGTTTGTCGTTTTCAGAAGACGGCTGCACTGAACGTCAGAAGCCGACTGCACTATA
GCAGCGGAGGGGTTGGATCCATCAGGCAACGACGGGCTGCTGCCGGCCATCAGCGGACGCAGGGAGGACTTTCCGCAACC
GGCCGTTCGATGCGGCACCGATGGCCTTCGCGCAGGGGTAGTGAATCCGCCAGGATTGACTTGCGCTGCCCTACCTCTCACT
AGTGAGGGGCGGCAGCGCATCAAGCGGTGAGCGCACTCCGGCACCGCCAACTTTCAGCACATGCGTGTAAATCATCGTCGT
AGAGACGTCGGAATGGCCGAGCAGATCCTGCACGGTTCGAATGTCGTAACCGCTGCGGAGCAAGGCCGTCGCGAACGAGT
GGCGGAGGGTGTGCGGTGTGGCGGGCTTCGTGATGCCTGCTTGTTCTACGGCACGTTTGAAGGCGCGCTGAAAGGTCTGGT
CATACATGTGATGGCGACGCACGACACCGCTCCGTGGATCGGTCGAATGCGTGTGCTGCGCAAAAACCCAGAACCACGGCCA
GGAATGCCCGGCGCGCGGATACTTCCGCTCAAGGGCGTCGGGAAGCGCAACGCCGCTGCGGCCCTCGGCCTGGTCCTTCAG
CCACCATGCCCGTGCACGCGACAGCTGCTCGCGCAGGCTGGGTGCCAAGCTCTCGGGTAACATCAAGGCCCGATCCTTGGA
GCCCTTGCCCTCCCGCACGATGATCGTGCCGTGATCGAAATCCAGATCCTTGACCCGCAGTTGCAAACCCTCACTGATCCGCAT
GCCCGTTCCATACAGAAGCTGGGCGAACAAACGATGCTCGCCTTCCAGAAAACCGAGGATGCGAACCACTTCATCCGGGGT
CAGCACCACCGGCAAGCGCCGCGACGGCCGAGGTCTTCCGATCTCCTGAAGCCAGGGCAGATCCGTGCACAGCACCTTGCC
GTAGAAGAACAGCAAGGCCGCCAATGCCTGACGATGCGTGGAGACCGAAACCTTGCGCTCGTTCGCCAGCCAGGACAGAA
ATGCCTCGACTTCGCTGCTGCCCAAGGTTGCCGGGTGACGCACACCGTGGAAACGGATGAAGGCACGAACCCAGTGGACAT
AAGCCTGTTCGGTTCGTAAGCTGTAATGCAAGTAGCGTATGCGCTCACGCAACTGGTCCAGAACCTTGACCGAACGCAGCGG
TGGTAACGGCGCAGTGGCGGTTTTCATGGCTTGTTATGACTGTTTTTTTGTACAGTCTATGCCTCGGGCATCAAGGCGAGCTC
AGAGACCATGGAAAGCATGTTCTCGGACTTACGTAGCAACTCGTTTCTTTTCGCAGGTTGAGCCACCTCCGCGCTTCATCAGA
AAACTGAAGGAACCTCCATTGAATCGAACTAATATTTTTTTTGGTGAATCGCATTCTGACTGGTTGCCTGTCAGAGGCGGAGA
ATCTGGTGATTTTGTTTTTCGACGTGGTGACGGGCATGCCTTCGCGAAAATCGCACCTGCTTCCCGCCGCGGTGAGCTCGCTG
GAGAGCGTGACCGCCTCATTTGGCTCAAAGGTCGAGGTGTGGCTTGCCCCGAGGTGATCAACTGGCAGGAGGAACAGGAG
GGTGCATGCTTGGTGATAACGGCAATTCCGGGAGTACCGGCGGCTGATCTGTCTGGAGCGGATTTGCTCAAAGCGTGGCCGT
CAATGGGGCAGCAACTTGGCGCTGTTCACAGCCTATCGGTTGATCAATGTCCGTTTGAGCGCAGGCTGTCGCGAATGTTCGG
ACGCGCCGTTGATGTGGTGTCCCGCAATGCCGTCAATCCCGACTTCTTACCGGACGAGGACAAGAGTACGCCGCAGCTCGAT
CTTTTGGCTCGTGTCGAACGAGAGCTACCGGTGCGGCTCGACCAAGAGCGCACCGATATGGTTGTTTGCCATGGTGATCCCT
GCATGCCGAACTTCATGGTGGACCCTAAAACTCTTCAATGCACGGGTCTGATCGACCTTGGGCGGCTCGGAACAGCAGATCG
CTATGCCGATTTGGCACTCATGATTGCTAACGCCGAAGAGAACTGGGCAGCGCCAGATGAAGCAGAGCGCGCCTTCGCTGTC
CTATTCAATGTATTGGGGATCGAAGCCCCCGACCGCGAACGCCTTGCCTTCTATCTGCGATTGGACCCTCTGACTTGGGGTTG
ATGTTCATGCCGCCTGTTTTTCCTGCTCATTGGCACGTTTCGCAACCTGTTCTCATTGCGGACACCTTTTCCAGCCTCGTTTGGA
AAGTTTCATTGCCAGACGGGACTCCTGCAATCGTCAAGGGATTGAAACCTATAGAAGACATTGCTGATGAACTGCGCGGGGC
CGACTATCTGGTATGGCGCAATGGGAGGGGAGCAGTCCGGTTGCTCGGTCGTGAGAACAATCTGATGTTGCTCGAATATGCC
GGGGAGCGAATGCTCTCTCACATCGTTGCCGAGCACGGCGACTACCAGGCGACCGAAATTGCAGCGGAACTAATGGCGAAG
CTGTATGCCGCATCTGAGGAACCCCTGCCTTCTGCCCTTCTCCCGATCCGGGATCGCTTTGCAGCTTTGTTTCAGCGGGCGCG
CGATGATCAAAACGCAGGTTGTCAAACTGACTACGTCCACGCGGCGATTATAGCCGATCAAATGATGAGCAATGCCTCGGAAC
TGCGTGGGCTACATGGCGATCTGCATCATGAAAACATCATGTTCTCCAGTCGCGGCTGGCTGGTGATAGATCCCGTCGGTCTG

GTCGGTGAAGTGGGCTTTGGCGCCGCCAATATGTTCTACGATCCGGCTGACAGAGACGACCTTTGTCTCGATCCTAGACGCAT
TGCACAGATGGCGGACGCATTCTCTCGTGCGCTGGACGTCGATCCGCGTCGCCTGCTCGACCAGGCGTACGCTTATGGGTGC
CTTTCCGCAGCTTGGAACGCGGATGGAGAAGAGGAGCAACGCGATCTAGCTATCGCGGCCGCGATCAAGCAGGTGCGACA
GACGTCATACTAGATATCAAGCGACTTCTCCTATCCCCTGGGAACACATCAATCTTACCGGAGAATATCGTTGGCCAAAGCCTT
AGCGTAGGATTTCGCCCTCTCCCGCAAACGACCCCTAAAAGCCGTTTCCTCTGTATAAAAGATCAGCTAAATTATGTGTATTGC
ACAATACATATATGTGAGGTTAGCAGTGAATTTGCCTACGCCCGAAACCTACGATGAACTTCAGAGAGCCTACGATTTTTTCAA
TGAGAAGCTATTCAGCAACGAGCTGCCGCCATGCCTGATAACGTTGCAGCGTGAGAAGCGAACGTATGGCTATTGTTCCTTTA
AGCGTTTCGTCGGCCGTGAGAGTGGGTACACGGTAGACGAGATCGCTATGAATCCGGTGTATTTCTCGATCAGAACCATAAAG
GCCACGCTTTCAACACTGGTGCATGAGATGGTTCATCAGTGGCAATTCCATTTTGGCGAGCCTGGCCGCCGTGGCTATCACAA
CAAACAGTGGGCGGCCCGGATGGAACGGGTAGGACTAATGCCTTCTGATACCGGCGAACCGGGAGGCAGGAAAGTGGGCC
AGAGCATGACCCATTATATTATTGCCGGTGGCCCTTTCGATATGGCCTGTGATGAACTGCTGACAGGCCATTTCCGGCTTTCCT
GGATGGACAGGTTTCCGCCTTACCAGCCTAAGCCTGGCGCTGTGCTAAGCCCTACAGGAAAAGGCTATATTGACGACGAGGA
AGATGATAGCGAACACGAACAGGAGGTGGAGGAAGGGCGCGACCCGGTTGAACTCGACGACGAGATCATAGAGGCCATGC
GATTTGTAACCCCACCGCCTGAAGCACCGGTGAACAAAACAAACCGGGAAAAGTACAGCTGCCCGGTGTGTCATATCAATCT
CTGGGGTAAACCGGGGATAGTGGTTTACTGTGGTGGCGAGCACTGTAATAAAGCCGCGTTAGTAGTCTTAAAATAAAGTCCTT
TCGGACTTTATTTTTTTTCCATTTCCGAGGTCGTGATGTTATTAATGCTGTACTTCGCGGCTTCTTTTAAAACAGTTTCAGCAAG
GCTTGCTGGTATCCAGACCTGAACTAATTTTAATGGTTCGCCGTTCTCGGCTTTAAGAGTGGTGTTCTGGTACAAATCCCAGAT
TCGCTTAACGGTGCTGGAAATGTTTTGCTTGGAACGGCCTACTCGCGTGGCTACGTCTGATGATTTCTCACCTTTGACAAGCA
CGGAATAGCCAATATCTGTTGTGATGTGTGCAAAGGAAGCCATTTGCGGCAGCAGCTGTTTCCATTCTGTTTCTGAAATTCTGT
TTTTCTGAGCCATCTGTGGCGCCTCCGTAGTTTTGGTTACAGAAAGGATATACTCAGAATAAACAGGGGTCAATACAAGTACG
ATTTTTATAAACTTTATTTTATTTGAGGGTGAGGCCCGGTGCGGCAGCAGCGCGGGCCTCGATGGTGCCGCGAAGGTGCTGG
CGCCATGCTCGGATTAAAACATGAACCGTGAAGAACTGCGAAACTTGTTTTCGCGGTTCTGAGGGGTTGACCGAGCCGCGA
AGCGGCGCTGGTAAGCGATGATATGCACATATCCACAGGCATATTTTTAAAAGGGGCACTGTTGCAAAGTTAGCGATGAGGC
AGCCTTTTGTCTTATTCAAAGGCCTTACATTTCAAAAACTCTGCTTACCAGGCGCATTTCGCCCAGGGGATCACCATAATAAAA
TGCTGAGGCCTGGCCTTTGCGTAGTGCACGCATCACCTCAATACCTTTGATGGTGGCGTAAGCCGTCTTCATGGATTTAAATCC
CAGCGTGGCGCCGATTATCCGTTTCAGTTTGCCATGATCGCATTCAATCACGTTGTTCCGGTACTTAATCTGTCGGTGTTCAAC
GTCAGACGGGCACCGGCCTTCGCGTTTGAGCAGAGCAAGCGCGCGACCATAGGCGGGCGCTTTATCCGTGTTGATGAATCG
CGGGATCTGCCACTTCTTCACGTTGTTGAGGATTTTACCCAGAAACCGGTATGCAGCTTTGCTGTTACGACGGGAGGAGAGA
TAAAAATCGACAGTGCGGCCCCGGCTGTCGACGGCCCGGTACAGATACGCCCAGCGGCCATTGACCTTCACGTAGGTTTCAT
CCATGTGCCACGGGCAAAGATCGGAAGGGTTACGCCAGTACCAGCGCAGCCGTTTTTCCATTTCAGGCGCATAACGCTGAAC
CCAGCGGTAAATCGTGGAGTGATCGACATTCACTCCGCGTTCAGCCAGCATCTCCTGCAGCTCACGGTAACTGATGCCGTATT
TGCAGTACCAGCGTACGGCCCACAGAATGATGTCACGCTGAAAATGCCGGCCTTTGAATGGGTTCATGTGCAGCTCCATCAGC
AAAAGGGGATGATAAGTTTATCACCACCGACTATTTGCAACAGTGCCGGTCGCCGGGAGTCAGCAGATCGACGTCAACGCCG
AGCAGCGATTTCAGTTCTTCTTCCAAATCGCCCAAGTCCAACAACGTGGCACCGGGCAGCGCATCGACCAACAGGTCGAGGT
CGCTGCCATCCCGGTCGGTGCCATGCAGCACCGAGCCGAAGACGCGCGGGTTCGCGGCGCGAAAGCGGCCTACCGCTTCAC
GCACTGCGCTTCGCTTCATGTCAAGCACAACAGACGGTCGCATGCGCATCCTTTCTTATCGAAACTCGTTGAGATGATATGCAA
TCAAGAATAGAATTTCAAGAACTCACAAAGTAACGCGGTGGTTAATATCCTGTACCCACGGATTGCCCTTAGCGCTGCCTATAT
CGGCTAAAGCACTCCGGTAGCTTGATTCACCCCACGGCCACGGCAGGATCTTGGCCGTCGCAAGCGCCAGGGGAAAATCTT
CAGCTGCAAGCCTGAGTGATTTCATGTGCGTGTAATCCATCGCCCAGATGATTTTTGTGAAGAAGAACTCGCGCTCGTTCATG
TCCGGGCGCGCGTCCTGGCCCTCCGTGCCAACGGCCAGCAAGTAATCGGCCTGAATTGGCAGTATCAGCGCGCGTAGTAAGT
CATGGATCGCCGGTTGCGGCAGTCTGCGCGCCAGATTAATTACCCGGTCGAACTTCAGTTTATCCTGCTTGCGCTTGTCGGTCT
GCTCCATCAGATTTCATGGCCCCCTTCTTCATGCTCATGCTCATGGGTGTGTTCTTTTCCGGTATGGCTCTGTTCCGCCTGAGAC
GTCTGCGGCATGGCGTAATCGTCGTAAATGCTGCTGTCAAAGTCGTAGTCTGATGCTTCGGCATAATGCTCGTAATCGGCATAC
TCCTGCGCGCTCCACTGCTGATCGTCGGCAGCAGCATAATCATGGGCCAGCTCTGCATCATTTTGCTGTGCTTCATGACGCCGC
AGGCCAACGGAATCATCCATAGGGTTCTGCTTAAGATGAAAGGCGTCCTCTGCGTTGCTCACCGGCTGATAATCAGTGCCGGT
TGTCATGTTATGTTCATCGGGTTTCTGGTTAAACGCCATGCTTTCCCCCGTGGCTTCTGGCAGACCTTTTTCAGCTGATCGGGT

TTCTAAACTGGTATCGCGGCCAATATCCTTAAACCTGGCCTCAAGCCCAAAGAAACGGTCAATTTCTGCGGCCGTGGTTTTCG
GGCTGTCGCGGCTCACGCTCGATGCCAAAGATTTTTTATCGTCGGTAAAAATTTCCACCTCATGACGCGCACGCGAAATACCA
ACATAAAAAACGTCCTTAGAAGTGGTAAGCGATTTGGTATCTATGTTGAACAACACGCGATCACAGGTAAGCCCTTGGGATTT
GTGGACGGTGGTTGCATAAGCATAGGAAAGATAAGAAGCCTGTTTTTTGTCCAGCTCAACCGTGCGCCCTTTTTTGTCCTCAA
GCGTCAGTTTTTCACCCTCCACGGTTTTCACCGTGAAGCGGTCGCCGTTGGCAACGTCCAGCGTTTTATCGTTACGCGTTACC
ATAACCTTATCGCCCGGCGCCAGTTCGGCGCTGACTGCCTGGTATACAGACAGCTTGGTGTGTGTACGCGGGCTGAAAGCGA
TCTGCTCACCGCTGCTGCTTTCAACCGTCAATTTGTTGCCCGGCCCGGTATCAAGAACCTGGTAAGACTCGCCCCGCTTCATAC
CATTTTTGTAATCCTGTTCGGGGATAATGATTTGCCCTTTACTGAAATAACGGCTGTCGCGGCGTTCCGCCTGTGTCGAATCCA
CGCGGTCAAGTAGCGTGAACGTTTCGCCGGTTCCGGCAAGCCCCAGATTGCCCCGGATGTAGTCATTGAGGGTTTTGCGTGA
GGCGTTCGTACCAGAGATTATCAGGGTGGCATCCTGTTGTTCTGAGGACAGAGACAGGTAGCGATCGGCAAGTTGAGCGAG
TCGGGGCGCTTCTTCCTTCAGTTCGTTCACGCCGGTGATATTTTTCAGGGCGCGCGCGGCATTACCTTCAGCGGCATACTTAA
CCGCCTCAAGCAAAACTTCATTCTTCTGTCGCTGAATGTCTTTCATGTAGCTGGTCTGCATATCTGCTTTAATCAGCTGCTCAAA
AGGCTTACCGGCTTCTACCGCTTTCGTCTGTGACGTATCCCCCAGGAATACCGCGCGAGCGTTATGCTTCTCGATCACCTCCAT
CAGCTGTTTCATCTGTCGGGCGGGTATAACCCCGGCTTCATCAATGAATACGACTGATTTTTCATCCAGCTTTTTATCCTTCGCT
TTGAGGAAAGCGGCAACGGTGCGGGCCGGTAATCCATCATCTTCAAGCGCTTTTTTCTGTGTCCCATAGGGGGCCAGCGCCG
TGACCTTCAGCCCTTGTGACTCCAGCAGCTCTTTAGCGGCCATCGTCATATAGCTTTTACCGGTACCGGCGTAACCATGTGCGG
CCACAAACCGATCTTTGCTCGTCACAATTTCTGTAACCGCGCGCATCTGCTCCTTCTTGAGGGTTTTCCCGGCAAGCAGCTGG
CCTGCAATCTCTGCGGTCAGCTGTCGCGGCATCTGCCCCCGGCCGCGTGATTCGATAGTCAGAATGGAACGCTCAAGGCGAA
TACCCTCCACGGTAGTGACGCGGTGGCTGGTCTTTTTAAGCCTGCCGTTTTTAATACCATCATCTACCGCAAAACGGGCTTTAT
CCGCACGCATCCCGCTATTCGTCAGCGAGTCGATCCACTCTTTGCGCGTCAGAGTTTCGGCCATAACTGAAGCACCGACCTTC
AGAGTTGATTGATACCGGGCTTCGCCCTCGATGATGGCGCCCTTCTGTACCGCCTTCAGGTACGCTTTTTCAACATCGGCTATT
GTGGCATGGCCCAGCACCTGCTTATTAGCGATTTGAATCAGCTTCTGGCGTTCAAAGCTGGCATCGCGCTCTGACAGCGACTT
AACTGCAAACTGGATAGCCCGGTCAGCTTTAACCTCCGGGCTGGTAAAATCCGGGGCCATGTTGCGCGCTATATCAGCCTCCA
GAGGTTTACCGTGTCCCTGCCATTCACGGTTATCAAAATCAATGCCGAGCGTTTTGGCGCGGCTGGCCCATTCCTGGTGAATT
TCTTCACGGGAATGCTCTGTTTTCTTTTCACGCGTAGCCATCGAGACGCGGCTTTTCGTCTGAGCATCGGCGGTTTCCCGCGT
CAGACCCATTGCAGCGAGTCCCTTTTCAATTTGCTCCGACCGGCGGGAAAAAGCGCGAATCTGTTCATCTGAAAAATGGGCC
ATATCGAACGTGTTATTTTTGCTGTTGTAACGCAGCTCATAACCGGCTTTGGTCAACTCCAACGCCAGCTCCTGTTTGTAAACAT
CGCCCAGGTGCATTTTGTTACGCATCAGCTCATCATTTTTGAGCGCGCGCCACTGGCCGTCCTCGCGCTGGGTCATGTTCATG
ACAAAAGCGTGTGTGTGCAAATCAGGATCTAGCGCCCTGGAAGTTTCGTGGCGGAAAGTAGCGACGACAAGGTTATTGGTAT
TCTGGGTTACTGATTTCCCCTGGCGAGTCGTCCGGGCCTGCGCGAGTTTTTCAGCTTCACGCACAGCAGCGGCAACAGCTTT
TTCATGAGCCTCGATAATGGTTTTATCGCCGTGTATCAGCGCCTGCATGGATACCCCTTTAGGCGCTGAAAACGTCAGGTCGTA
GCCCAGACGCTCTTTTTTGGCATCACCCACGTGTCGCTGCATATGCGTGAAGGTATCTATCTCTCCGACAAGCAGCTCTTTAAA
CCGGGCTGATTCAACGTCCCCGGATAAGCCGAGGGCTTCAGCTCCGGTTCCCTGCCAGGACGTGAATGATGAATCCTTACTGT
AGTAATCATCCTTTGCATCAGAGTAGTAGCCCACAACGCTAGTGACGTTCTGGCGGGTAATCGTGGTTATATCAAGCATCAGAT
CTCCCTCAGTTCAATGCCAGGAACAGGGTTTTTGCGATGGTATTTAACGTGTTTAGCCTTGAACTTAGCGACGGGCATATCACC
AGGCAACGCCAGATAGCCGGTGAGGTTTGGCAACATTGATATTTCGGTAGGCGTTACGGCACGAACAACTTTAACGTCGCGG
CGTTTACGGACAATCCAGGGCTTCTGAGGATCGGATTCTTTACGCTCAACTTCGCCTTCTATCTCACCGAGTGAGCGCGACATT
TGATCCAACGTTTCATCACCGAGACGGCTGCCGCCCAGCACGATGTTAGAACGCATGTTAGCCAGAATTGTCTGAGCCATATC
CCGACCATAAACCTTAACCAGCTGAGAATAGGTTTGATAGCCAGCATAAACACACAGACCGCTTTTACGCCCTTTGGTCAGTG
CATCGTTGAGGTTTGGCAGAAACTGGAGTGATTCCAGCTCGTCAATAAATACATTAATGCGGCTTTCTTTTTCACCCATACCCA
GCACGATAGAAAAAATCGAATCCAGCCAGCAGGAAATTAGCGGATTAAGTGACCTTTTCATTTCTTCCTGCCAGGTGATAAAC
AGGGTTCCCGGCTTTCCATCATCAAGCCAGTCACGCAGGGAAAAATTACCTTCCGGCATTTTCAAATGTGGGGCAAGATTCTT
ACTGAGAACAAATCGCGCGCTTCCAACTGCTTTTTCAGACCCGGAAAAAATAGCTTCGGCAGGCGTCCCCATTAAAAATTCTT
TTAATTTTTCTGGTCAACGTTACAGGCCCAGTGAATAACTTCTTCCATAGTTACTGTGCTGTATAGGCTGTGAAGTTTTTTCGA
AACTTCACTAAAAATAAGACGGCCATAGCCGAACCATTCTTCAGTAGCCATATCAGGGCTTTCCTGAACAATAGAGTTCACTAA
ACGCTCGTAATCATATGAACGGCGAATTTCATTGAAAAACACCCAGCCTTCAGTGCGTTTATCATAGGCGTTTAAAATAACATC

GCCGGGACGATAGAAATTCTTTAAGAACCCCCCATTTGGATCTAAAGCAATATTTTTGCCGCCTCTAATGATGCTCTTAAATAAC
AGTTCATTGAAAATTGTGGTTTTACCAGTACCGGTTGTACCGGCAATCGAAAAATGCAAGTTCTCAGCGTATGTAGGTATGGG
GATATTAGCCACGGTTAACTGGTTGACACCTCTTTCGCGTGTTTTATCAGCGAGTGTTCTGGCGCGAACAAGCTCTGTACCAC
GATAAATCTTTTTGAATCTTTCGCCTTTAAACACGCGTGATTTATCATAAATGATAAAAGCGATCAGACCGCCAACACCAATAAA
CCAGCCAGCAATTAAAGCTGACCATAAAGGCCATAGCGAAAAAGTATTCTTAACCAGATACGGAATCAGGTATTTAGCCGTGG
ATGGATCAATACCGTAGGTAAATTTTGCAACTAGAAACCATACCATCACTGGAGGCAAAGTAATTGCAAATAAAAATGCTAAGC
CTCTTTCTCTATCGTCCATTTCAGCGCTCCTTTTTTGGTTCCCAGACTTTGTAGCCGTTACGTTCAACCTCTGCTTTTGCCGCTTT
GGTTTTGCCCGGTTCTGCTATCGAGCGCAGGAGGATTAGCGTTTCAATAGCGAGTGATTCATGCAACATCATCTGTCCTGCCG
GTGGGAATTTTACGCCAGATAGCGTTTCGGTTATTGCCTTCAGCTCATCGCGCAGTGGGCCAAAATCCGCATCTGAAGCACGG
TCAAAAAGATAATCCAGTTTGCGATTTACGTCGCTCAGCCGGTCGGCAACTATTTTCAACCCGGACTCCCGATCACCTGGGCC
AGCTTCAATGCAGCGCCGCAGATAATCTGACCGATTACCTCCTGAAACCAGGTCTATATAGGCCAAAAGTTCATCTGATACTTT
TGCGGTTATTATTGGCATTCAGTCCTCACATTGTGCATTTCTTAAACAAAAAATTGGGATCTAACAAGCTGAAATCTTAGTATTA
CCAAAGTAATAAAGCAAACTCATTATAAAACAATGGGTTATTGGGTGTTTTTAATACCTAATTATTACCGAATATTGACGCTATTT
ATTTTTTTATCTTTTAAATCAGTACGATAGCGTGATTTATCGCGCTGCGTTAGGTGTATAGCAGGTTAAGGAAAAAAAATCATCT
TTTTTGGTAGGAGCGACCTCCGTAGGTTAAGGGTCATTTGGCTAAAAAGCGTCCTATTCTTTGATGGTCATGCTTGCATGACCA
TCTGAGCAACCAAAAACTACAGATAAACTACAGAGAAACTACAGATAAACTACAAAAAACGATTTACCTTAGCGTTGTCAGAC
TACTAATAGACTACAAGGAAACTACAAAGAAACTACAAAGAAACTACAAAGAAACTACAAATAGACTACTAAAACCGTGGCA
GACTACTAATAGACTACAAGAAAACTACAAATAAACTACAAAACTGGATTGACCCCTTCTTACGAGTGTTGTAGAGTCATCTTC
ATACAACGGAGGGGGTTATGAATAAACCAGCAGATTCTGAAACCCCGCAACTTATCGGCTGCTGTCAGATTGCGCCTAAATGA
AATCGAGAACTGGCTGGACAGAGGGCTAACGCGGCATGAAATTGCTGAAATCCTCGACAGCGAATACAGCTTTTCGGTAACA
GCCAAAGGGCTTGAGATGGCACTGTATAGAACGCGGCAAAACCGAAAAAATGTATTGCACAATACACATGATAAGAGTAGCG
CGAAGGGTGCAGCGGAAAGTGTATTGCACAATACACAACCGTCTGAGCCTGAAGCGCAGGAAAGTGAAAAAGCAGAGAGT
CCCGGCATTATTGATAAAGAGTTCTTCAATAAAATCGGTGAGGATTTCAACCCTAAGAAGTTCAACAAAAAATTCTGAGGTGA
TTTATGAAAGTAGCGGTAATTAATTACAGTGGCAGTGTTGGTAAAACATTAATTTCATCTTACCTGTTAGCCCCGCGCCTGACTG
GTGCAAAGTTCTATGCGGTAGAGACTATCAACCAGTCTGCTTCCGATCTGGGTATTGAAAATGTGACCAGTTTTAAAGGTGAC
GACTTCTCACGTTTGATTGAGGATATTGTTTTTGAAGATGCAGGCATTATTGATATTGGCGCGTCAAACGTTGAAGCGTTCCTG
ATGGCTATGTCTCGCTTTGACAGTGGCGCGAACGAATTTGATAAATATGTAATCCCGGTGACGCCGGATAATAAGGCGATTGAT
GAAAGCCTGAAAACGGCACACACGTTAAGTAAAGCGGGCGTGAGCAGCAAGAAAATTATCTTTGTTCCAAACCGTATTAGTC
CAGACAGTGAAGTAGAAGATGTGCTGGCGCCGGTGTTTGAGTTTGTCAAAGAAACGAAGATTGGCAAAATAAGCAAGAAG
GCTGTTATTTATAACAGTGAGGTTTTCGAATATCTGGCGTTTCACCGTATCTCATTCGAAGTATTGACCGCTGAAGATCCAGAA
GAATTCAAATCCCGTGCAAAACAAACAACCGATGCTGACGAGCGCAAAAAACTGGCACGCCGTTATACATACATGAAACAGG
CGATTCCGGTAAAAGCTAATCTCGATAAAGCATATGCGGCTTTAATGGGAGAATAAAATGGAAAAGCAGCCGGATAAATTAGA
AGTTCTGATGGACTGGTTTTTAGGTGACGCGAAGGAAATCACCGCAACTCAGAAAGAAATGACGCAGAAACTTTCTGAGCTT
TCGGAAAAGCTGGCAAAAGACACCGAAAGTTTAGGAGAGACGGCAGACTCTTTTAAACGGGCTTTAGTAGAAAACCAGCG
TTCAATTAGCCTGGCAATTAGTGATGATGCTAAGGCGCGCGAGGAATTTCTAACTAAATTCCGCCGCGCGCAGGCGTCCAGTG
CTGAGACGTTTACCCGTCAGATCCTTTTTATTACAGCTGGCTGCACCATCGTGGGCGCCGCAGTAGGCGCCGCGATAGCGATA
CTTTTACTGAGATAAAGCAAACCGGGCGTGTCCCGGTTTTTTTGTCAAGCGGAGCGCGGAGGCCGAAGGCCGGAGGCATTA
GTGGCCGCCGCCCGCGTAAGCGGGGCGAGACGGGAACCGGCTCGAAGCGCAGCACGGCAGAACGGCCCCGCAGGGGCA
ATGCCCGTTTTAATTCATCGTGACAGTCGCGCGTGACCATCACGGGGAGAAAAATAATGAATGACCGACAGCGAGAACTGGC
CCGTATACGCCAGGCCCGCCGCCGCGCGCGGCTCAAGGAAGAAGGCACAAGCGTGACAGTCACGCTAACAAAACAGGAAG
AAGCAATGTTGCAGGAGCTGTGCCGGGTTCGCCGTCCTGGACGAACGCCTTATTCAACGAACGAATTTTTCCAGCTGCTGCT
TATCCGCAACTGGCAGCAGTGGCAGGAGCAGAAGGCACAGCTGGGAAAATGCCAGGCTTGCGGAAAGCTGAAAGCGGAG
GGGGGGTGCGAGGGTGAACGGAAAGGCGAAACCTTTAACTGCTGGCTTGCCGTCGAAGCCAATGAACTAAATTTGTAGTGT
ATTGTGCAATACACATTTACACAGAAACAAAAACCACCGGCAATTCCTGGAACCGGATACCTACGGCTATTCCTGGGTGAACG
GTACTTTTTGCACCTGGGTGCGCTGAAAAAGCTGAATATGCAGGGTGACGTTCGCGTGCTGTTCTGCTTTGTAGACTGAATGC
GCCAGCTATACGCCTGACTGCTTAAACCTGGTAAAGTTCTGCAACCGGCACTGACCGGAAAGCAAGGCAGGGAAGACCTAA

GCCAGAAACCTTGACTGCTCCCCGCCCTTCAGGGCGGGGATTGCGGATCATGTTCTTCTCTTTCAGGGATTCAACGCAGACA
AGAAAGGCTTTCAATTTCCTATACGTGAACGGCCGCGCAGCGGAAGAAACAAGCCCGGTCAATCCGGGCTTGTTTCTTTAGG
CGGCTCAGAAATCGCCTAAAGGCCCGGCTTGGCCGGGCAGTCAGTGCTATTTAGTTTGTTGCAGCAGCTGGCTTAATTTTGC
CGCCAGTGCATACGTCGATTGGAAAGCGCCTTGCAGGCGCTGATTAGGCAGCTGGTTGAACGCTTCGAGGCAGGCGCGCAG
CAATAATTCTTTCTGAGATTCGACTTCTTTTTTCAGTTGGGAAGGGGTGGTAACTGTAGTCATGCTTGCTCCTTAGTGAGCCGA
TATCGGCAATTTTTCGGGTGGCGGTGTTGCCTCCCGATGATTTAATTATCGGTGATTATGCTTTTAAAGTCAATACAGGTACGG
AATTTATTTACCTGTTTTTATGCCCGTCAGGGCATGGAAGGCGACCGCGCCGGACTCCACCGGACACCGGCCGCAAATCGCC
GGAAACTGCGGGACTGACCGGAGCAACAGGCCAACCCCCCTCCCTGCTAAGCCATAACCCAGCCCGCCGCCACGCAGCTGC
CGCACGTCCCCCACGGGGGTGCGCAGTGGGCGCCGCGCGCCTGCGCGCGGGTACGGCGGCCCGCCTGCGGGTCGCGGCG
CCGTACTGCGAGTTAGCGGCCGCCGCGCGGCCGGTTACGGGGGACACCGCACAGTCACGGCCAGTGCCCCGCTGAGCTGC
ACAATCCACGGATAACACAATAGCGCACTGGCAAAGGATGCCGACGCCTGAAGGGCGTGGGCACCCCGAAGGGGCGGGGC
GGCCGCTTGCGGCCGGGCGAGTCCGGCGCAGGGTGTGGCCTGCCAAGCGGAGCGCGGAGGCCGAAGGCCGGAGGCGTT
AGCGGCCGCTGCCCGCGTAAGCGGGGCGAGACGGGAACCGGCTCGATGCGCAGCACAGCAGAGCGACCCCGAAGGGGTA
ACGCCCGGTGTGGCATCAGGATTTAGTGCAATGGCAGAACATGAGCTGGAGAGATCACCGGCAAGCAGCAGCAAAGGGGC
GGCACAGCCGCCCCGATGGCTGTTTGCCGATACCGGCGATTAATTAGAGCGGTGTTTAATATCCCCCGCGTTGCGGGGGACTA
GGTTTCAGCAAGTCATGTTAAATACGTGTCCATCATGTAAACTGAAATCCCCAATAAACAGATCCCGCGCATAGGCTACGATGT
CAAAATATCGGGCTACGGATTCCGGAATATCATTCAGTAGACCGCTATCATTCAGGTATTCCTCTGCAAAAGTTTCTTCGTCCTT
AGCTTCGCCCATATAGGCATCTCTAAACAGGTCGAAATCAGTGCTATTAAACAGATCAACAAAGGCCACAAACGCCGCTTCGT
TACCTTCCTCGCGGGCTTGTTTAAAGCCGTTAATAAAATCCCAGTTGATATGGCACTCTGACGCCATACCAGACGGAATACCCT
CCCAATCTTGGAACATAAATTCTGGATCAGCCTCATTTGCGTGTAACTCGCGGCAGCGCTCGTAAAACTCCTCTGAGCTATCAA
AATCGGTCAGATCGAGCCAGGCTCCCGCAATGCTTCCGCAGTTGTATTTATGGTAAGTGCCAACATAAACAGAAGGGGTCGTA
ATATCAGTCATGGTGTACTCCTTAAAGCGCCGATACCGGCAATTTTTCGGGCGGCGGTATTGCCTCCCGATGATTTAATTATCGT
TGATTATGCTTTTAAAGTCAATACAGATACGGAATTTATTTACCTGTTTTTATGCCCGTCAGGGCATGGAAGGCGACCGCGCCG
GACTCCACCGGACACCGGCCGCAAATCGCCGGAAACTGCGGGACTGACCGGAGCAACAGGCCAACCCCCCTCCCTGCTAAG
CCATAACCCAGCCCGCCGCCACGCAGCTGCCGCACGTCCCCCACGGGGGTGCGCAGTGGGCGCCGCGCGCCTGCGCGCGG
GTACGGCGGCCCGCCTGCGGGTCGCGGCGCCGTACTGCGAGTTAGCGGCCGCCGCGCGGCCGGTTACGGGGGACACCGCA
CCGTCACGGCCAGCGCCCCGCTGAGCTGCACAATCCACGGATAACACAATAGCGCACTGGCAAAGGATGCCGACGCCTGAA
GGGCGTTGGCACCCCGAAGGGGCGGGGCGGCCGCTTGCGGCCGGGCGAGTCCGGCGCAGGGTGTGGCCTGCCAAGCGG
AGCGCGGAGGCCGAAGGCCGGAGGCGTTAGCGGCCGCTGCCCGCGTAAGCGGGGCGAGACGGGAACCGGCTCGATGCGC
AGCACAGCAGAGCGGCCCCGAAGGGGTAACGCCCTGTGTGGCATCAGGATTTAGCACAATGTCAGAACATAAACTGGAGAG
ATCACCGGCAAGCAGCAGCAAAGGGGCGGCACAGCCGCCCCGATGGCTGTTACTTGTCTTTGTCGCGTAGCACTTTGATTAG
GCCGGTTACGGCCGTAATCAGAGCGGCCAGCGAGGTGATGATTTGCGGTAGGTTTTCGAGGATGGTAGAGGTCATATAGCAC
CTGTAGAGAAGTTGGCGGGGTGTCGTTTCCGACGGCCGCACTGTAACCGGGCGAATAAGGCAGGTTGTCAACAGCTTGAGC
GAAGCGTCTGTTGACAACCTGCCGCGCCCGGTTTCACTGCGGTCATAGGCGGAACGACCCACGCCAACGGAACGGCTTTAT
GACCGGGCAGCTGAGATACCGGCGAACCTGGCTGGCGGCTGACGCCAGCCGCCAAGCGCCAGCGCGGAGGGCAAAGCCC
GGAGGCCAAGCGGAGCGCGGAGGCCGAAGGCCGGAGGCCGGAGGCGTTAGCGGCCGCTGCCCGCGTAAGCGGGGCGAG
ACGGGAACCGGCTCGATGCGCAGCACAGCAGAGCGGCCCCGAAGGGGTAACGCCCGGAGTCTGCCGCTGTTTATCTCTCGT
TCCATCTGAAATCGGCGGTAAGGCCATTAAAAGGGTCAGTTTATCAGGGAGGCGTTAGCCCCCCATGTTGTTAATCATCAGGC
AATATCGTCTTTGTAGCAGGCATAACCGAAGCTAAGTCTCTGTTTTCATATAGTGGCGGGCAAAGTCCCAGGCATCGTGGCCG
AAGTCCTCATAATCTGCCAGGACGATTTCGCGGGCTTTGTGCCATTGCTGTACGGAAGAAAGCGGCAGAACAGGGCAGGGG
TGCGACCAGTCAGTGACGGTGTTGCAGATCATATCTGCCAGACGCTCCAGGGAGCCGTAAACCAGCTCTGTGCGCAGATTCG
CCACCATGCGTTGTTTACGCAGTGATGCCAGATAATCAATCTCTTTGGTTATATCAGAATTTAAGCGGGTCTGGTAATCCATGAT
GTACTCCTTTGCGCGCCGATACCGGCAATTTTGCGGGCGACGGTGTTGCCTCCCGATGATTTAATTATCGGTGATTATGCCCTC
AAAGTCAATATAAGTACGGAATATGCATGCATAATTTTATATCTTGCAAAGCGTTCATAGAGTGCCTGAATCGCTTTCTGACAGC
CTCAATAAAAAAAGGCGGGGATTCCCGCCTTTTTTCTTACAGCTGCTTACGTGGCTTTTTACGCGTCATATACAACGGTATCGC
GCAGTCTACCGCGTACAAAAAGCACGCCAGCGCGCCGCAACCGTACAGAAACGCAAGCGGCTTATTATCGAAGTAGCTGAA

AACCCCTGTCGCCGCACACAGGCCCAGAACAGAGACGCAGGCGCAGGTGATCTGCACCAGATCCCTGTATCCCGCACGAAC
GATAAACCAGGGCAGGGCAAGCGCAGCGGCGCTAATAATTAATGCGAGAGGGACAAAAACGAGATAGTGATACATGTGAAC
TCCTTGATGGTTGCCGATACCGGCGATTGTTCGGGCGGCGGTATTGCCACCCGATGATTTAATTAGAGGTTTTGCGCGTCCAG
GAGATTGACCTGAGCCGGGGTAACGTGAAACTTTTCCCCTTTATGGATCACGTTATGCGGGGCGCTAATTTCATCACTGATAA
AGCTAACCGGGTAACGTTTTTTACCGCAAATCCGCTCGCTAAACCATGCCACTTTTGCCGCTGGCCGATCCACTGGATGAACA
ATCACACCGGCCATGCTGCAACCCGTTGCGGGTTCGTCCAGCGTAATGCTTACCGGGACTGTATCCCGGTAAAAAAACTTAAC
CGGCGGCACACCTGCCTGCGTAGCGGCTGCGACAACTGCAAGCCCGATAACCGCTATCCGATTAATAAGCATTTATTCCCCTT
ACTCATGCTGATATCACCTTGCCAGCTGTTACCAGTTTACGAAATTCACTTTCATGAATTTCACGCCCATGCTCAAGCGTTGAAT
ACACGTTGCCGATAAGCCAGTTACCAGCCGTTTTTGTCTCGGTATACCACCATGCTTCTGTCAGAGTAAGCAGCGGTTCGCTAT
CCTCGCCCTTCTCATAGATCCAGATTTTAGTGACGTCTTTACCCGTTTCGTTGCTGCCCTGAATGGTAGGGTCAAAGGTATGTTC
AATCTCTATATCGAAGTAACGCTGAAGGAAGTTAGTAAAGTGCATGACGACTCCTGTAAGCGCCGATACCGGCAATTTTTCGG
GTGGCGGTGTCGCCTCCCGATGATTTAATTATCGTTGATTATGCTTTTAAAGTCAATACAGGTACGGAATTTATTTACCTGTTTTT
ATGCCCGTCAGGGCATGGAAGGCGACCGCGCCGGACTCCACCGGACACCGGCCGCAAATCGCCGGAAACTGCGGGACTGA
CCGGAGCAACAGGCCAACCCCCCTCCCTGCTAAGCCATAACCCAGCCCGCCGCCACGCAGCTGCCGCACGTCCCCCACGGG
GGTGCGCAGTGGGCGCCGCGCGCCTGCGCGCGGGTACGGCGGCCCGCCTGCGGGTCGCGGCGCCGTACTGCGAGTTAGCG
GCCGCCGCGCGGCCGGTTACGGGGGACACCGCACCGTCACGGCCAGCGCCCCACTGAGCTGCACAATCCACGGATAATGCA
GGAGACGAATCATGATAGGAGGCTGAAGGGGAAATGAGCGGCAGCAGGGGAAGGGGTTGCCAAGCGGAGCGCGGAGGC
CGCAGGCCGGAGGCGTCAGTGGCAGCTGCCCGCGTGAGCGGGGCGAGACGCGTAGCGGCTCGATGCGCAGCACAGCAGA
ACGGCCCCGGAGGGGTGACGTCCGGGGGTTCGCTTTTAAAGATTTTCGACCACATCAGTAAATCGTAGTGACACCATGAAGC
AAAAGTATCGTGCAGACCAGAATCAACAACAGTAACAGCAGACCTTTTTTTCGACGTAATAAAACCGGCCCCAAAGCGCCCG
CAAGAAGCACACTCAACACGATTTCCGTTATACTAATCGTATTCATAATCTCTCACGTTTCCCTTTTTAGAACTCTGCCACACAC
AGATAAAACCTTATAACAAGCTACAAAACCCGTTATTCAGACGCGGTAATGCCTAGTTTTTTTGCCAGTATTTCAGATGACAAA
GAAACCCTTATCCACTTCCCGGTTACAAGGTGAATGATTGTGGCTTCATGCCCTTTAACAGAGTGGGATATTAACTCACGCTGA
ATGATATGCGTCTCTCCATCATTTCCCGTCACGGCAATTAGGCCCTCTGTAGAACAGGGGTTGATACTACTAATGTTGGCGTTGT
TTTCGGTTTTGTTCAGCATCGCTGATCCTCAAATATCGGTTTGTGTTACGTCTGCCGCTTTGCGCTGGATAAGCGACTTAAAGA
AATCCGACGCCTTCAGAATATCGCTATCCTGGAAGTCGGGAAATGTCGCTTCTGTCAGCTCCTGCGCCGTACCTGCCCGTATAA
CGGTTTCATCGCGGATCATCGTTCGCTTAACGCCATCAGCCTGTTTTTTGCTGAAGGAGTGCCGGAAAATAATCGCGTATTGG
CCGCCGCTATCCTGATGAACAGAAGTATCCCAGCGATGATTACCCTGTTTACGATAGTGGTGATAGATTTGCTGGCCGTAGCGC
TCCTGATCGGAATTACGGTAATCGAACATTAACAGTTCCAAAAGCATTAACCGATCTGGCAATTGCCAGGCGGTAGGGTGTTG
AAGTGTCGCTAACATAGTTTCCCCTGAGCGTGACAGTCACGATAAGGCGGGCTTTGCCCGCCTGGTTATCAGTTAATCAATGG
CACGATAAATACGATTCTGGCTTTCGTTCTCCAGCGTATTAACGTACTCCCGCAACAGGTGATACCGGTTAGCCATCGTTTCGTT
CAGTTCGGCTTTGCCTTCTTCATATGCCAGGCCGCAAAAGTAACTGTAGGCATACAGACAAACAATAATCCCTACTTCGCGCGC
GCTGCATTCACCTTCAAAATAGTTAGGTAACGAGAGCCAAAGAGGTTGGGGCGCTTCCATAAAAAACGTGCCATTGCTGGCC
TGAAGGTATTCCCAATACCCTCCCTGGTAGTCTTTAGCGTAACGATTCAGAAAGGACTGAATGAAGTGATCTGCGCTGAAGAA
AGCGCCACGAAATGCCGCAGGCATGAAGTTCATGCGGGCGTTTTCAGAAATGTAGCGGGCGGTGATTTCGATAGTTTCCATG
ATACTTCCTCTTAAAGCCGATACCGGCGATGGTTAAGCGGCAGGCACATCACCTGCCACTTTTTAATTATCGTACAATGGGGCG
TTAAAGTCAATATAAGTACGGATTATATTTACCTAATTTTATGCCCGTCAGAGCATGGAAGGCGACCTCGCCGGACTCCACCGG
ACACCGGGGGCAAATCGCCGGAAACTGCGGGACTGACCGGAGCGACAGGCCACCCCCCCTCCCTGCTAGCCCGCCGCCAC
GCGGCCGGTTACAGGGGACACTGAGAAACAGAAAGCCAACAAACACTATATATAGCGTTCGTTGGCAGCTGAAGCAGCACT
ACATATAGTAGAGTACCTGTAAAACTTGCCAACCTGACCATAACAGCGATACTGTATAAGTAAACAGTGATTTGGAAGATCGCT
ATGAAGGTCGATATTTTTGAAAGCTCCGGCGCCAGCCGGGTACACAGCATCCCTTTTTATCTGCAAAGAATTTCTGCGGGGTT
CCCCAGCCCGGCCCAGGGCTATGAAAAGCAGGAGTTAAACCTGCATGAGTATTGTGTTCGTCACCCTTCAGCAACTTACTTCC
TACGGGTTTCTGGCTCGTCAATGGAAGATGGCCGCATCCATGATGGTGACGTACTGGTTGTGGATCGCTCGCTGACGGCCAG
CCACGGCTCAATCGTAGTCGCCTGCATCCATAATGAATTTACCGTGAAGCGGCTACTGCTGAGGCCCAGACCCTGCCTGATGC
CGATGAACAAAGATTTTCCTGTGTACTACATTGACCCGGATAATGAGAGCGTTGAAATCTGGGGAGTGGTTACGCATTCCCTT
ATCGAGCATCCGGTATGTTTGCGCTGATTGATGTCAATGGCATGTACGCCAGCTGTGAGCAGGCATTTAGGCCAGATCTGGCA

AACCGAGCAGTGGCCGTTTTATCCAACAATGACGGCAACATTGTGGCCCGTAATTACCTGGCGAAGAAAGCGGGCCTGAAA
ATGGGCGATCCGTACTTCAAAGTCAGACCCATAATCGAGCGTCATAACATCGCTATTTTTAGCTCTAATTACACTCTCTATGCCTC
CATGTCGGCCCGGTTCGCGGCCGTAGTTGAGTCCCTTGCAAGCCACGTCGAACAGTATTCAATCGACGAGCTTTTTGTTGACT
GCAAAGGGATAACGGCCGCCATGAGCCTTGACGCTTTCGGGCGCCAACTGCGCGAGGAAGTCAGGCGACACACAACGCTG
GTATGCGGGGTCGGTATTGCCCGTACTAAGACGCTGGCGAAGCTGTGTAACCACGCTGCAAAAACATGGCCCGCTACTGGCG
GGGTGGTTGCTCTGGACGATGGCGCCAGACTGAAGAAATTAATGAGCATCCTGCCGGTTGCGGAAGTCTGGGGCGTCGGCC
ATCGTACAGAGAAAGCACTCGCCACAATGGGGATCAAAACGGTGCTGGATTTAGCCAGGGCAGATACGCGCCTAATCCGTAA
AACATTCGGCGTTGTGCTTGAAAGAACGGTACGGGAGTTGCGCGGCGAGGCTTGCTTCAGCCTGGAAGAAAACCCTCCTGC
GAAGCAGCAGATTGTTGTGTCGCGCTCATTCGGCCAACGCGTAGAAACCCTGACGGACATGCAGCAGGCTGTCACCGGATTT
GCAGCGCGCGCAGCTGAAAAACTGCGTAATGAGAGGCAATACTGCCGCGTCATAAGCGTCTTTATCCGTACCAGTCCTTATTC
AGTGCGTGATACACAGTATGCCAATCAGGCAACCGAAAAACTGACGGTGGCAACCCAGGACAGCCGCACGATAATTCAGGC
AGCACAAGCCGCGCTGGCGCGGATCTGGCGGGAAGATATTGCGTATGCAAAAGCAGGGGTCATGCTGGCAGATTTTAGCGG
GAAGGAGGCCCAGCTTGATTTATTCGACTCTGCTACGCCTTCAGCTGGCAGCGAGGCTTTAATGGCTGTTCTTGATGGTATAA
ACCGGCGTGGAAAGAGCCAGCTTTTTTTTGCAGGCCAGGGCATCGATAACTCCTTTGCCATGCGTCGTCAGATGTTGTCACCT
GATTACACGACAGACTGGCGCTCAATACCAATAGCCACCATCAAATAATTACCGGCGCCGTACACGTGCCGGTTAACCCCTCA
ACCGGCCGAAACAAGTTTCGGCACGGTTTCGCGGTTTTCGGTAAAAGCCGTTTCCTCTGTATAAAAGATCAGCTAAATTATGT
GTATTGCACAATACATATATGTGAGGTTAGCAGTGAATTTGCCTACGCCCGAAACCTACGATGAACTTCAGAGAGCCTACGATT
TTTTCAATGATAAGCTATTCAGCAACGAGCTGCCGCCATGCCTGATAACGTTGCAGCGTGAGAAGCGAACGTATGGCTATTGT
TCCTTTAAGCGTTTCGTCGGCCGTGAGAGTGGGTACACGGTAGACGAGATCGCTATGAATCCGGTGTATTTCTCGATCAGAAC
CATAAAGGCCACGCTTTCAACACTGGTGCATGAGATGGTTCATCAGTGGCAATTCCATTTTGGCGAGCCTGGCCGCCGTGGCT
ATCACAACAAACAGTGGGCGGCCCGGATGGAACGGGTAGGACTAATGCCTTCTGATACCGGCGAACCGGGAGGCAGGAAA
GTGGGCCAGAGCATGACCCATTATATTATTGCCGGTGGCCCTTTCGATATGGCCTGTGATGAACTGCTGACAGGCCATTTCCGG
CTTTCCTGGATGGACAGGTTTCCGCCTTACCAGCCTAAGCCTGGCGCTGTGCTAAGCCCTACAGGAAAAGGCTATATTGACGA
CGAGGAAGATGATAGCGAACACGAACAGGAGGTGGAGGAAGGGCGCGACCCGGTTGAACTCGACGACGAGATCATAGAG
GCCATGCGATTTGTAACCCCACCGCCTGAAGCACCGGTGAACAAAACAAACCGGGAAAAGTAGCTGCCCGGTGTGTCATATC
AATCTCTGGGGTAAACCGGGGATAGTGGTTTACTGTGGTGGCGAGCACTGTAATAAAGCCGCGTTAGTAATCTTAAAATAAAG
TCCTTTCGGACTTTATTTTTTTTCCATTTCCGAGGTCGTGATGTTATTAATGCTGTACTTCGCGGCTTCTTTTAAAACAGTTTCAG
CAAGGCTTGCTGGTATCCAGACCTGAACTAATTTTAATGGTTCGCCGTTCTCGGCTTTAAGAGTGGTGTTCTGGTACAAATCCC
AGATTCGCTTAACGGTGCTGGAAATGTTTTGCTTGGAACGGCCTACTCGCATGGCTACGTCTGATGATTTCTCACCTTTGACAA
GCACGGAATAGCCAATATCTGTTGTGATGTGTGCAAAGGAAGCCATTTGCGGCAGCAGCTGTTTCCATTCTGTTTCTGAAATT
CTGTTTTTCTGAGCCATCTGTGGCGCCTCCGTAGTTTTGGTTACAGAAAGGATATACTCAGAATAAACAGGGGTCAATACAAG
TACGATTTTTATAAACTTTATTTTATTTGAGGGTGAGGCCCGGTGCGGCAGCAGCGCGGGCCTCGATGGTGCCGCGAAGGTG
CTGGCGCCATGCTCGGATTAAAACATCAACCGTGAAGAACTGCGAAACTTGTTTTCGCGGTTCTGAGGGGTTGACCGAGCCG
CGAAGCGGCGCTGGTAAGCGATGATATGCACATATCCACAGGCATATTTTTAAAGGTATTTTATAGATTTTTTATCTTTTTAAAG
TCTTTTAGAGCTATATAACTCATTGATTTAAAATCATAAATAAGTGTTATCTCTGGGAATCCGCCCACCTTGTTATGGGAATTGGC
CCACCTTACTATGGGAAACAGCCCACCTTACTATGGGAATTAGCCCACCTTGTTATG
